# Patient-reported measurement of time to diagnosis in cancer: development of the Cancer Symptom Interval Measure (C-SIM) and randomised controlled trial of method of delivery

**DOI:** 10.1186/1472-6963-14-3

**Published:** 2014-01-03

**Authors:** Richard D Neal, Sadia Nafees, Diana Pasterfield, Kerenza Hood, Maggie Hendry, Simon Gollins, Matthew Makin, Nick Stuart, Jim Turner, Ben Carter, Clare Wilkinson, Nefyn Williams, Mike Robling

**Affiliations:** 1 North Wales Centre for Primary Care Research, Bangor University, Gwenfro Unit 5, Wrexham Technology Park, Wrexham LL13 7YP, UK; 2 School of Medicine, Cardiff University, Neuadd Meirionnydd, Heath Park, Cardiff CF14 4YS, UK; 3 Betsi Cadwaladr University Health Board, Ysbyty Gwynedd, Penrhosgarnedd, Bangor, Gwynedd LL57 2PW, UK

**Keywords:** Cancer symptoms, Patient intervals, Appraisal, Primary care intervals, Diagnosis, Randomised controlled trial, Tool development

## Abstract

**Background:**

The duration between first symptom and a cancer diagnosis is important because, if shortened, may lead to earlier stage diagnosis and improved cancer outcomes. We have previously developed a tool to measure this duration in newly-diagnosed patients. In this two-phase study, we aimed further improve our tool and to conduct a trial comparing levels of anxiety between two modes of delivery: self-completed versus researcher-administered.

**Methods:**

In phase 1, ten patients completed the modified tool and participated in cognitive debrief interviews. In phase 2, we undertook a Randomised Controlled Trial (RCT) of the revised tool (Cancer Symptom Interval Measure (C-SIM)) in three hospitals for 11 different cancers. Respondents were invited to provide either exact or estimated dates of first noticing symptoms and presenting them to primary care. The primary outcome was anxiety related to delivery mode, with completeness of recording as a secondary outcome. Dates from a subset of patients were compared with GP records.

**Results:**

After analysis of phase 1 interviews, the wording and format were improved. In phase 2, 201 patients were randomised (93 self-complete and 108 researcher-complete). Anxiety scores were significantly lower in the researcher-completed group, with a mean rank of 83.5; compared with the self-completed group, with a mean rank of 104.0 (Mann-Whitney U = 3152, p = 0.007). Completeness of data was significantly better in the researcher-completed group, with no statistically significant difference in time taken to complete the tool between the two groups. When comparing the dates in the patient questionnaires with those in the GP records, there was evidence in the records of a consultation on the same date or within a proscribed time window for 32/37 (86%) consultations; for estimated dates there was evidence for 23/37 consultations (62%).

**Conclusions:**

We have developed and tested a tool for collecting patient-reported data relating to appraisal intervals, help-seeking intervals, and diagnostic intervals in the cancer diagnostic pathway for 11 separate cancers, and provided evidence of its acceptability, feasibility and validity. This is a useful tool to use in descriptive and epidemiological studies of cancer diagnostic journeys, and causes less anxiety if administered by a researcher.

**Trial registration:**

ISRCTN04475865

## Background

Mortality from cancer is worse in the UK than most other European countries [1,2]. The reasons for this are multi-factorial, but diagnostic delays and consequent later stage diagnoses are likely to be major contributory factors [3]. Interventions to reduce diagnostic delays, which result in a less advanced stage at diagnosis, have the potential to improve cancer survival [4], although tumour biology is also important. Interventions need to account for lead-time bias whereby more timely diagnosis may improve survival by bringing forward the diagnosis rather than delaying mortality. Diagnostic delays (perhaps better referred to as ‘time-intervals’ since there is not always a ‘delay’) may occur throughout the cancer diagnostic pathway. Whilst a minority of patients are diagnosed through screening (in some cancers), and some present as an emergency to A&E or via inter-specialty referral (without consulting in primary care), the majority of diagnoses are made for patients who follow the ‘typical’ cancer journey involving symptomatic presentation through primary care [5-8]. In the UK, and elsewhere, there has been a drive in policy towards early, and more timely diagnosis of cancer; for example the National Awareness and Early Diagnosis Initiative (England), the Detect Cancer Early Initiative (Scotland) and the International Cancer Benchmarking Partnership (several countries).

The duration between first symptom and cancer diagnosis is important because, if shortened, it may lead to earlier stage diagnosis and improved cancer outcomes [9,10]. Measurement is complex because some symptoms are simply present or absent (e.g. a breast lump or rectal bleeding), whilst others are not instantly noticeable (e.g. tiredness or weight loss). Most studies reporting both ‘appraisal intervals’ (‘time taken to interpret bodily changes/symptoms’) and ‘help seeking’ intervals (time taken to act on those interpretations and seek help’) [11] use tools that ignore existing models of patient behaviour [12,13], are poorly or inadequately validated, and are open to bias. There is a well-recognised need to develop valid instruments for measuring ‘delay’ [11], and this is one of the recommendations of the Aarhus checklist on the design and reporting of early cancer diagnosis studies that has recently been published [14].

We previously reported the first phase of the pilot work to develop and pilot a postal version of such a tool (the ‘DELAYS’ tool) [15]. This questionnaire was tailored to individual cancers and asked patients to recall the dates of the onset of symptoms (based on referral guidance symptoms) and their presentation to primary care, in addition to socio-demographic and health questions. One issue that arose (predominantly from phone calls from potential respondents to the research team) was the potential anxiety that may be generated by use of the tool (for example asking patients to recall when they first experienced symptoms may cause upset if they feel that their diagnosis was unduly delayed). The other main issue was that the response rate to the postal questionnaire was only moderate (46.2%).

Hence, the aim of this paper is to report the further development of the ‘DELAYS’ tool, now renamed the Cancer Symptom Interval Measure (C-SIM), through in–depth cognitive testing, and its testing in a randomised controlled trial (RCT) comparing different methods of delivery (on the premise that anxiety may be less in the presence of a researcher). The primary objective of this RCT was to compare the level of patients’ anxiety between two methods of delivery (self-completed and researcher-completed) of administering a tool, which measures time from first symptom to cancer diagnosis. Secondary objectives of this trial were: to compare the difference in completion rates between two methods of delivery; to describe the process, and difficulties associated with either method of delivery; to compare the information about symptoms from the patient tool with that obtained from primary care records. Hence, we gathered further information in the trial about the quality of the tool, primarily *acceptability* (does it provoke undue anxiety; completion rates, completion time) and *feasibility* (problems with administration). Focused qualitative evaluation presented in the paper (cognitive interviews) adds to the evidence for the measure’s *face validity*, and the comparison of self-report against records adds to the measure’s *criterion validity*[16,17].

## Methods

Phase 1. Further development of the tool and cognitive debriefing interviews with cancer patients.

The aim of Phase 1 was to improve the previously reported DELAYS tool [15], to make it suitable for use in the trial, building on the previous work and making it more acceptable, more likely to be completed, and less likely to cause anxiety.This further development was informed by the cognitive aspects of survey methodology approach [18-20] and utilised the findings from systematic review evidence regarding survey response [21-23]. We recruited ten patients to complete the modified tool and participate in cognitive debriefing interviews. They were either allocated to self-completed or researcher-completed mode. In addition to asking patients questions about symptoms related to their own cancer, we also asked about the wording of questions relating to symptoms of other cancers. Post completion, the participants underwent a cognitive debriefing interview. Areas identified as problematic in the initial piloting [15] were specifically addressed.

Key interview probes were used that directly and intentionally match the steps of the standard cognitive model for survey response (comprehension, retrieval, judgement, response formulation) [19]. These probes were:

• What the respondent believed the question to be asking.

• What specific words and phrases meant to the respondent.

• The type of information the respondent needed to recall in order to answer the question.

• What types of strategy the respondent used to retrieve information.

• Whether the respondent devoted sufficient mental effort to answer accurately and thoughtfully.

• Whether the respondent wanted to tell the truth, or whether the respondent wanted to say something that made him/her look better (i.e. social desirability).

• Whether the respondent matched his or her internally generated answer to the response categories given by the survey question.

The data were analysed to identify dominant trends across interviews and to identify ‘discoveries’ (unexpected problems that were only present in one interview, but were important). Findings from these interviews informed further re-wording of the tool. Lastly, we worked with three patient groups within the North East Wales NHS Trust (now part of Betsi Cadwaladr University Health Board) to ensure that the tool captured the diagnostic journey in a way that was acceptable and easy to understand.

### The final tool

This revised tool (C-SIM), in 11 separate versions (lung, colorectal, breast, pancreatic, gastric/oesophageal, renal/bladder, endometrial/cervical, haematological, ovarian, prostate and testicular cancers), was then tested in the RCT. It comprised questions concerning the timing of the onset and presentation of symptoms. Respondents were invited to provide either exact or estimated dates of first noticing symptoms and presenting them to primary care. These covered cancer site specific symptom questions (variable between cancers); and four general cancer symptom questions (loss of appetite, weight loss, fatigue and ‘feeling different’). One question asked whether they had been sent for any GP-initiated tests. Further questions covered employment status, educational qualifications, ethnicity, whether they lived alone, co-morbidity, smoking status, family risk of cancer. Finally, as part of the RCT a measure of anxiety, the State-Trait Anxiety Inventory (STAI) was added as the main outcome measure [24]. The tool was produced on double sided A4 paper and was 11 pages long (see Additional file 1–the colorectal tool, and Additional file 2–the cancer specific questions for the other 10 versions). The final version of the tool built upon that previously reported [15] in that we:

• Removed the calendar landmarking tool, because participants reported that they didn’t find it useful

• Added a personal letter from the Principal. Investigator inside the front cover, to try to improve the response rate [22].

• Improved the format of the boxes for the questions regarding symptom duration.

• Added more tests (CT scan & ultrasound scan), that were missing from the original.

• Made multiple changes to the wording to make it more patient-centred and easier to understand.

Phase 2. Randomised controlled trial of method of delivery of the tool.

### Setting and participants

The trial took place in the three district general hospitals in North Wales (Wrexham Maelor Hospital, Ysbyty Gwynedd, and Ysbyty Glan Clywd), now all part of Betsi Cadwaladr University Health Board. Fieldwork was undertaken by research officers/nurses from the NISCHR Clinical Research Collaboration. The research officers/nurses attended MDT meetings for the following cancers: breast, colorectal, lung, gynaecological, urological, upper GI, and haematological. At these meetings all patients with new primary diagnoses were alerted to the researcher, who then liaised with the clinical nurse specialists and other members of the clinical teams in order to assess eligibility for inclusion. Patients were eligible if they had a new diagnosis of one of the 11 cancers, were aged 18 years or above (with no upper age limit), were neither too ill nor close to death at diagnosis, were not detected by screening (some cervix, breast and colorectal), and were mentally and linguistically competent to complete the tool.

Eligible patients were then sent a letter from the clinical team inviting participation, enclosing a patient information sheet, a return FREEPOST envelope and a contact form. The researchers then contacted patients who responded positively and arranged to meet them in order to obtain consent (for data collection and access to primary care records), randomisation and tool completion. Reminders letters were sent after two weeks.

Participants randomised to the self-completion arm were handed the final tool (see above) and asked to complete it. For those randomised to the researcher-completion arm, the researcher sat with the participant in a quiet place and went through the tool with them, reading out the questions and entering their answers. Identical tools were used for both groups. All of the researchers received training to ensure that the delivery of the tool was as standardised as possible. Standard prompts in response to questions for both groups were permitted.

The trial was conducted from February to December 2009. Data were entered using Cardiff TeleForm (optical character recognition system); 22 questionnaires (two for each cancer site) were manually checked.

### Randomisation

Patients were randomised to either the researcher-completed or the self-completed mode. A computer generated random number sequence based on random permuted blocks of sizes 4, 6 and 8 was used. Allocation concealment was done by the controlled use of sequentially numbered opaque envelopes, stratified by cancer site and by hospital, except for: pancreatic, testicular and ovarian cancer where the expected prevalence was too small to stratify by hospital and a block size of 4 was used to minimise the risk of chance imbalance; and haematological, endometrial/cervix that were stratified by hospital but were also randomised using a block size of 4.

### Outcome measures

#### ***Measuring anxiety***

We used the six-item State-Trait Anxiety Inventory (STAI) to determine whether respondents had anxiety related to questionnaire completion [24]. This has a low-burden, and was designed to assess short-term fluctuations in anxiety. Each item was scored 1-4, with a total score out of 24, with higher scores indicating higher anxiety.

#### ***Process evaluation***

After completion of the tool, the researcher completed a brief sheet for each patient comprising the following questions (with answer options):

• How long did it take the patient to compete the questionnaire? (<5 minutes, between 5 and 10. minutes, between 11 and 15 minutes, >15 minutes)

• Where did the patient complete the questionnaire? (separate room, open area, while receiving treatment).

• The researcher answered one further question for the self-completed group only: ‘Did the patient ask for any help?’ (Y/N). This was to capture data relating to help sought when it had not been offered. The researcher answered two further questions for the researcher-completed group only. These were a subjective report of difficulty and anxiety observed whilst the researcher was completing the tool with each patient:

• Did the patient find any of the questions difficult? (Y/N).

Was the patient made anxious? (Y/N).

### Data analysis

#### ***Analysis of primary outcome***

The primary outcome when comparing the method of delivery was anxiety, with the analysis carried out blind to treatment allocation. We aimed to transform the data to produce an approximately normal distribution and undertake parametric tests; if not possible then non-parametric testing was undertaken. Missing items were imputed (see findings).

#### ***Analysis of secondary outcomes***

##### Completeness of questionnaire

The difference in completeness between the two methods of delivery were compared (z statistic) on questions common to all of the respondents. Hence we analysed data for:

• The four generic cancer questions. If the respondent ticked the ‘yes’ box for having the particular symptom, they were then assessed as to whether the rest of the question was entered completely. If they entered dates for first noticing and first reporting (whether exact or estimated), or they reported that they didn’t tell their GP or nurse, it was regarded as ‘complete’. Any less than this was regarded as ‘not complete’.

• The demographic questions.

##### Process and difficulties

From the post-completion sheets, we determined the process of and difficulties associated with both methods of delivery. This was descriptive other than *X*^2^ for comparison of tool completion between modes of delivery.

##### Comparison of recall of date of first presentation against GP records

We sought to obtain evidence of criterion validity by comparing the dates of consultation recorded by the measure from up to 60 patients against their GP records. A protocol was developed to identify 40-50 consecutive completed questionnaires, ensuring that there were at least 10 patients from each centre and at least three from each cancer site. Patients were excluded if they had the reported symptoms for more than two years (because unlikely to represent the first presentation of cancer). With patient consent we contacted their GP, and asked them to complete a template. We validated the date of reported first presentation of each symptom, for both exact dates and estimated dates. For exact dates, we asked GPs whether there was a consultation on that particular day, or within a two-week window either side. For estimated dates, we developed a protocol for calculating a ‘pseudo-exact’ time-window from the estimated date (see Additional file 3), and provided the GP with this time window for searching. We also asked the date of the actual consultation according to the records. For simplicity, we only analysed data for one symptom per consultation (we chose the first one that the patient listed on their questionnaire).

##### Time intervals in the diagnostic journey

Whilst the actual time intervals in the diagnostic journey are not reported in this paper, we developed, a protocol for changing reported estimated dates into pseudo-exact dates that permit the calculation of time intervals based upon the responses obtained (Additional file 4).

### Sample size

The primary aim of this study was to detect a difference in anxiety between the two methods of delivery of the tool, with the completion rates as a secondary outcome. 150 patients per group (total 300), with a 95% CI (with a comparative completion rate of 75%) would allow us to detect a difference between groups on response rates assuming a difference of 10% (95% CI +/-9%). An overall completion rate of 75% would give 225 questionnaires for analysis of anxiety. This would give a 95% CI for the difference in log anxiety for the two modes of total width 0.16 (0.08 from the mean to the limit). This is based on a SD of log anxiety of 0.3 (based on previous studies).

## Results

**Figure 1:**
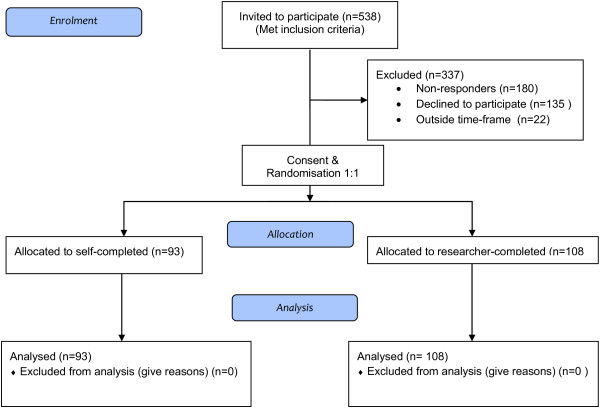
CONSORT 2010 flow diagram.

Consent was obtained from 201 patients, all were randomised and completed questionnaires were received from all 201 participants (93 from self-completed and 108 from the researcher-completed) (see Figure 1: CONSORT flow diagram). A further 22 patients initially expressed an interest in taking part in the trial, but were not contacted further, because we anticipated reaching our target sample size.

1 Group similarity at baseline

The comparison between the two groups at baseline is shown in Table 1 which shows that there were no important differences between them.

2 Analysis of primary outcome

The STAI scores were not normally distributed and could not be transformed into a normal distribution by either log, exponential or square root transformation. Consequently non-parametric analysis was undertaken, with and without imputation of individual data if the STAI score was missing (median values imputed). 18 (9%) missing values were replaced with the median value seven; this was carried out to allow us to assess the effect of missing values. Without imputation of missing data for the researcher-completed group (n = 107) the mean rank was 83.5; for the self-completed group (n = 76) the mean rank was 104.0 (Mann-Whitney U = 3152, p = 0.007). The median STAI score before replacing missing values were: researcher completed group 7.0 (IQR 3); self-completed group 8 (IQR 7). With imputation of missing data for the researcher-completed group (n = 108) the mean rank was 91.9; for the self-completed group (n = 93) the mean rank was 111.5 (Mann-Whitney U = 4042, p = 0.014). Hence STAI scores were significantly higher in the self-completed group. The median STAI score after replacing missing values with the median were: researcher completed group 7.0 (IQR 3); self-completed group 7 (IQR 5.5).

3 Analysis of secondary outcome

Completeness of recording

The completeness of recording for the four generic cancer questions is shown in Table 2. Although numbers were small, the completeness of recording was significantly better in the researcher-completed group for one of the four generic symptoms (decrease in appetite). Missing data for the demographic and STAI items are shown in Table 3.

4 Process of administering the tool

Duration of completion is shown in Table 4. This showed no significant difference between the groups (*X*^2^ = 2.23, p = 0.33). A greater proportion of participants in the researcher-completed group took >15 minutes. Help was provided to 46/93 (49.5%) of the self-complete group. The researchers reported that only 10/108 (9.3%) of the researcher-completed group found the questions difficult. The researchers administering the tool did not report that any participants in the researcher-completed group were made anxious. The tool was completed in a separate room for 122 (60.1%), in an open area for 53 (26.4%), and whilst receiving treatment for eight (4.0%). Data were missing for 18 (9.0%).

5 Comparison of patient questionnaires against GP records

**Figure 2:**
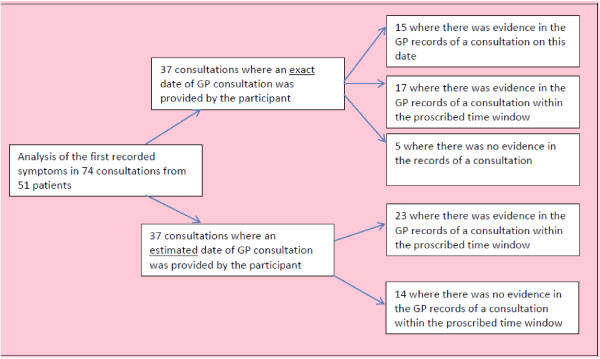
Validation of patient questionnaires against GP records.

Data from 51 patients were compared against their GP records (30 from the researcher-completed group, and 21 from the self-completed group). Of these: 16 had breast cancer; six colorectal; six lung; four endometrial; three each for haematological, pancreatic, prostate, and renal/bladder; and two testicular. The 51 patients had 74 consultations available for comparison with the GP records. 28 patients had one consultation available, 13 had two, five had three, three had four, and two had five. The findings are shown in Figure 2. This shows that there was greater evidence of consultations being recorded in the GP records for participants who reported an exact date rather than for those who reported an estimated date. For exact dates there was evidence in the GP records of a consultation on the same date or within the proscribed time window for 32/37 (86%) consultations. For estimated dates there was evidence for 23/37 (62%) consultations within the proscribed time window.

**Table 1:** Baseline comparison between groups

	**Self-completed**	**Researcher-completed**
	**n = 93**	**n = 108**
*Cancer site*		
Breast	28 (30.1%)	28 (25.9%)
Colorectal	15 (16.1%)	15 (13.9%)
Upper GI	5 (5.4%)	4 (3.7%)
Haematological	10 (10.8%)	10 (9.3%)
Pancreas	2 (2.2%)	3 (2.8%)
Testicle	1 (1.1%)	1 (0.9%)
Lung	11 (11.8%)	15 (13.9%)
Ovary	1 (1.1%)	3 (2.8%)
Prostate	15 16.1%)	21 (19.4%)
Renal/bladder	2 (2.2%)	3 (2.8%)
Endometrium/cervix	3 (3.2%)	5 (4.6%)
*Age* (years)		
Mean (SD)	65.3 (11.3)	63.6 (12.2)
*Gender*		
Male	45 (48.4%)	56 (51.9%)
Female	48 (51.6%)	52 (48.1%)
*Employment*		
Employed full time	23 (24.7%)	17 (15.7%)
Employed part time	5 (5.4%)	12 (11.1%)
Self employed full time	6 (6.5%)	2 (1.9%)
Self employed part time	2 (2.2%)	8 (7.4%)
Unemployed	1 (1.1%)	2 (1.9%)
Retired	44 (47.3%)	59 (54.6%)
Sick/disabled	3 (3.2%)	3 (2.8%)
Looking after family/home	4 (4.3%)	2 (1.9%)
Other	2 (2.2%)	3 (2.8%)
Missing	3 (3.2%)	0 (0.0%)
*Educational level*		
GCSE/O Level	19 (20.4%)	16 (14.8%)
A Level	5 (5.4%)	5 (4.6%)
Diploma	12 (12.9%)	12 (11.1%)
Degree/Higher degree	15 (16.1%)	20 (18.5%)
Other	11 (11.8%)	23 (21.3%)
None	26 (28.0%)	29 (26.9%)
Missing	5 (5.4%)	3 (2.8%)
*Ethnicity*		
White British	79 (84.9%)	85 (78.7%)
White	11 (11.8%)	13 (12.0%)
White Irish	0 (0.0%)	1 (0.9%)
Other white	1 (1.1%)	8 (7.4%)
Indian	0 (0.0%)	1 (0.9%)
Missing	2 (2.2%)	0 (0.0%)
*Domestic status*		
Alone	20 (21.5%)	21 (19.4%)
Spouse/partner (+/- others)	63 (67.7%)	81 (75.0%)
Child/ren (+/- grandchildren)	5 (5.4%)	3 (2.8%)
Sibling/s	0 (0.0%)	1 (0.9%)
Parents	1 (1.1%)	0 (0.0%)
Friends	1 (1.1%	0 (0.0%)
Other	1 (1.1%)	1 (0.9%)
Missing	2 (2.2%)	1 (0.9%)
*Co-morbidities*		
Previous cancer	6 (6.5%)	8 (7.4%)
Diabetes	9 (9.7%)	6 (5.6%)
COPD	3 (3.2%)	7 (6.5%)
Asthma	7 (7.5%)	16 (14.8%)
Other lung disease	1 (1.1%)	2 (1.9%)
Heart disease	11 (11.8%)	18 (16.7%)
Arthritis	16 (17.2%)	18 (16.7%)
Peptic ulcer	0 (0.0%)	3 (2.8%)
Irritable bowel syndrome	8 (8.6%)	9 (8.3%)
Inflammatory bowel disease	6 (6.5%)	5 (4.6%)
Anxiety or depression	19 (20.4%)	23 (21.3%)
*Smoking*		
Current smoker	12 (12.9%)	16 (14.8%)
Ex-smoker	43 (46.2%)	55 (50.9%)
Never smoked	36 (38.7%)	37 (34.3%)
Missing	2 (2.2%)	0 (0.0%)
*At risk of cancer because of family history*		
Yes	26 (28.0%)	23 (21.3%)
No	65 (65.9%)	84 (77.8%)
Missing	2 (2.2%)	1 (0.9%)
*Time between diagnosis and completion* (days)		
Mean (SD)	85.5 (54.4)	102.4 (62.3)

**Table 2:** Completeness of recording for the four generic cancer symptoms

**Number of patients reporting this symptoms**	**Complete data**	**Incomplete data**	**z statistic**	**p**
**Fatigue or tiredness**				
Researcher-completed (n = 55)	53	2		
Self-completed (n = 37)	34	3	-0.99	0.32
**Unexplained weight loss**				
Researcher-completed (n = 29)	28	1		
Self-completed (n = 24)	23	1	-0.14	0.89
**Decrease in appetite**				
Researcher-completed (n = 25)	25	0		
Self-completed (n = 23)	18	5	-2.80	0.005
**Feeling different in yourself**				
Researcher-completed (n = 35)	35	0		
Self-completed (n = 29)	27	2	-1.64	0.10

**Table 3:** Missing data regarding the demographic questions and STAI items

	**Self-completed**	**Researcher-completed n = 108**	**z statistic**	**P**
	**n = 93**			
**Demographic questions**				
Employment	3 (3.2%)	0 (0.0%)	1.88	0.06
Educational level	5 (5.4%)	3 (0.9%)	0.94	0.35
Ethnicity	2 (2.2%)	0 (0.0%)	1.53	0.13
Domestic status	2 (2.2%)	1 (0.9%)	0.71	0.48
Smoking	2 (2.2%)	0 (0.0%)	1.53	0.13
At risk of cancer (family history)	2 (2.2%)	1 (0.9%)	0.71	0.48
**6-item STAI**				
STAI item 1	7 (7.5%)	0 (0.0%)	2.90	0.004
STAI item 2	14 (15.1%)	0 (0.0%)	4.18	0.000
STAI item 3	17 (18.3%	0 (0.0%)	4.64	0.000
STAI item 4	10 (10.8%)	0 (0.0%)	4.64	0.000
STAI item 5	13 (14.0%)	1 (0.9%)	3.63	0.0003
STAI item 6	14 (15.1%)	0 (0.0%)	4.18	0.000

**Table 4:** How long did it take the patient to compete the questionnaire?

	**Self-completed**	**Researcher-completed n = 108**	***X***^**2**^	**P**
	**n = 93**			
5-10 minutes	16 (17.2%)	11 (10.2%)	2.23	0.33
11-15 minutes	35 (37.6%)	43 (39.8%)		
>15 minutes	38 (40.9%)	50 (46.3%)		
Missing	4 (4.3%)	4 (3.7%)		

## Discussion

### Summary of main findings

We have developed the Cancer Symptom Interval Measure (C-SIM) for collecting patient-reported data relating to appraisal intervals, help-seeking intervals, and diagnostic intervals in the cancer diagnostic pathway for 11 separate cancers. This tool is easy to use and quick to complete. It has evidence for acceptability, feasibility, face validity and criterion validity. The main findings from the RCT were that there were lower anxiety levels in the researcher-completed group. It is not known whether the higher level of anxiety in the self-completed group was a result of the cancer diagnosis, or the completion of the questionnaire. Data completeness was also greater for the researcher-completed group for some of the items.

### Discussion of the findings within the context of the literature

There are very few reports of the development of measures to measure diagnostic times in cancer [14]. We are not aware of any other studies that specifically measure anxiety within this context. Hence it is difficult to know whether the relatively elevated anxiety in the self-complete group is specific to this tool. A recent review demonstrated that there is a small but significant difference in subjective outcomes reported either through self-completed or interviewer-completed modes [23]. So the validity of response is partly driven by that feature of data collection mode. Because of that it may be that some of the difference in anxiety found between forms of completion in this study could be attributable not only to the calming (or similar) effect of the researcher, but also due to associated response bias. For example, this could be that patients giving answers to researchers do not want to show their feelings whilst those self-completing forms are being more ‘honest’–a form of social desirability bias. Unger-Saldana *et al* have recently reported the development and validation of a questionnaire to assess treatment delays in breast cancer [25]. Their researcher-administered tool was aimed at lower socio-economic groups, and included many domains about the diagnostic process; hence is very different from our tool. Corner *et al* reported similar levels of reporting of the date of consultation between patients’ recall and evidence from general practice records (Kendall’s tau-b = 0.65) [26].

### Strengths and weaknesses

This was an adequately powered RCT with an overall completion rate of 75%, and hence with a low risk of bias [27]. As anticipated, there was a higher degree of missing data in the anxiety domain from the self-completed group. We did not assess anxiety prior to administration of the tool, hence there is the possibility that the anxiety levels in the groups were different at baseline, Whilst we have assumed that the missing data were missing completely at random, these individuals did exhibit a greater degree of anxiety and there could be a theoretical association between anxiety and completion. The generalisability of the findings is limited with regard to ethnicity, as the sample was almost exclusively white.

### Implications for policy, practice and research

Given the lack of evidence of validation of tools in this area, we are happy for the Cancer Symptom Interval Measure (C-SIM) to be used in future descriptive and epidemiological studies of cancer diagnostic journeys, and to assess the functioning of current diagnostic service provision. Given our findings of less anxiety and more complete data collection in the researcher-completed group, we would recommend the researcher-administered approach where feasible. However, this clearly has resource implications, and limits its use in population-based studies. The agreement between patient questionnaires and GP records was moderate and whilst there was greater evidence of consultations being recorded in the GP records, for participants who reported an exact date rather than an estimated date, for many consultations the participants were unable to report an exact date, hence keeping both is probably necessary. Further work is needed to refine the tool in the light of the emergent findings, as some questions are easier to complete than others. Further research could also look at what may serve to reduce anxiety by researcher-administered forms. This could include having a professional and supportive presence when completing the form.

## Conclusion

We have developed and tested a tool for collecting patient-reported data relating to appraisal intervals, help-seeking intervals, and diagnostic intervals in the cancer diagnostic pathway for 11 separate cancers, and provided evidence of its acceptability, feasibility and validity. This is a useful tool to use in descriptive and epidemiological studies of cancer diagnostic journeys, and causes less anxiety if administered by a researcher.

## Competing interests

The authors declare that they have no competing interest in the conduct of this study.

## Authors’ contributions

RDN, DP, KH, MH, MM, CW, and MR designed the study and obtained the funding. BC conducted the randomisation. SN and DP conducted the fieldwork, with input from RDN, MH, NW, SG, MM and NS. BC, JT and KH undertook the analysis. RDN wrote the first draft of the paper, and all co-authors have approved the final draft of the manuscript. RDN will act as guarantor. All authors read and approved the final manuscript.

## Pre-publication history

The pre-publication history for this paper can be accessed here:

http://www.biomedcentral.com/1472-6963/14/3/prepub

## Supplementary Material

Additional file 1 The colorectal tool. Click here for file

Additional file 2 Cancer specific questions for all cancers. Click here for file

Additional file 3 Protocol for calculating ‘pseudo-exact’ time windows from estimated dates for the validation. Click here for file

Additional file 4 Protocol for calculating ‘pseudo-exact’ dates from estimated dates to calculate time intervals. Click here for file
