# Supplementary material for: Patient-reported measurement of time to diagnosis in cancer: development of the Cancer Symptom Interval Measure (C-SIM) and randomised controlled trial of method of delivery
Source: BMC Health Serv Res. 2014 Jan 3;14:3. doi: 10.1186/1472-6963-14-3 (PMC3922822; doi:10.1186/1472-6963-14-3)
Supplement: Additional file 2 — Cancer specific questions for all cancers. [file 1472-6963-14-3-S2.docx]

**Cancer Symptom Interval Measure (C-SIM)**

**Protocol for calculating ‘pseudo-exact’ dates from estimated dates for the validation**

| **When patient has given:** | **Action:** |
| --- | --- |
| Exact date | Search 14 days either side of the exact date |
| Seasons | Spring – search March to May  Summer – search June to August  Autumn – search September to November  Winter – search December to February |
| Month | Search 14 days before month beginning and 14 days after month end |
| ‘early (month)’ = day 1–10 | Search 14 days either side days 1-10 inclusive |
| ‘mid (month)’ = day 11–20 | Search 14 days either side days 11–20 inclusive |
| ‘late (month)’ = day 21–30/31 | Search 14 days either side days 21 – 30/31 inclusive |
| ‘last week, last month’ | Count back one and treat as week or month, and search 14 days either side (unless in future) |
| ‘x months/weeks ago’ | Count x months/weeks back from date questionnaire completed, then search 2 weeks either side of that month/week  (n.b. ‘about 3 weeks ago’ will be treated as 3 weeks ago) |
