# Supplementary material for: Patient-reported measurement of time to diagnosis in cancer: development of the Cancer Symptom Interval Measure (C-SIM) and randomised controlled trial of method of delivery
Source: BMC Health Serv Res. 2014 Jan 3;14:3. doi: 10.1186/1472-6963-14-3 (PMC3922822; doi:10.1186/1472-6963-14-3)
Supplement: Additional file 4 — Protocol for calculating ‘pseudo-exact’ dates from estimated dates to calculate time intervals. [file 1472-6963-14-3-S4.doc]

**Cancer Symptom Interval Measure (C-SIM)**

**Protocol for calculating ‘pseudo-exact’ dates from estimated dates to calculate time intervals**

| **Text for estimated date** | **Rule** | **Example text** | **Example recode** |
| --- | --- | --- | --- |
| ‘x days/weeks/months/years ago’ |  | *‘4 months ago’* | Count back 4 months from completion date |
| **Few / about / approx / range etc** |  |  |  |
| ‘Range of months or years ago’ | Take midpoint of range | *‘2-3 years ago’*  *‘Jan-Mar 08’* | Count back 2.5 years from completion date  15.02.10 |
| ‘about (or approx) x days/weeks/months/years ago’ | Ignore ‘about’ or ‘approx’ | *‘About 4 months ago’* | Count back 4 months from completion date |
| ‘Two days’ | Take first date | *‘3/4 Feb 2008’* | 03.02.2008 |
| ‘Early Feb/Late Jan 09’ (or similar) | Take 1st Feb 09 | *‘Early Feb/Late Jan 09’* | 01.02.09 |
| Few weeks/months etc ago | Few=3 | *‘Few months ago’* | Count back 3 months from completion dates |
| **Incomplete month/year** |  |  |  |
| ‘Year’ (only) | 01.07.xxxx | *‘2008’* | 01.07.2008 |
| ‘Date/Month but no year’ | Take first one | *‘15^th^ July’* | The first 15.07 prior to completion date |
| ‘Month’ (no date) | Take 15^th^ of the month | *January 2007* | 15.01.2007 |
| **Early/late/mid etc** |  |  |  |
| ‘Early year’ (only) | 15.02.xxxx | *‘Early 2008’* | 15.02.2008 |
| ‘End of year’ | Take last date in year | *‘End of 2009’* | 31.12.2009 |
| ‘Mid month’ | Take 15^th^ month | *‘Mid-January 2007’* | 15.01.2007 |
| ‘Late month’ | Take 23^rd^ month | *‘Late-January 2007’* | 23.01.2007 |
| ‘End of month’ | Take last date of the month | *‘End Jan 09’* | 31.01.2009 |
| ‘2^nd^ week in the month’ | Take 10^th^ month | *‘2^nd^ week in Jan 07’* | 10.01.2009 |
| ‘Beginning of month’ | 1st date of the given month | *Beg Feb 08* | 01.02.2008 |
| ‘Beginning of month’ (no year given) | 1st date of the given month | *Beg Feb* | The first 01.02 counting back from completion date |
| **Seasons and holidays** |  |  |  |
| ‘Spring’ | 15.04.xxxx | *‘Spring 2007’* | 15.04.2007 |
| ‘Early Spring’ | 15.03.xxxx | *‘Early Spring 2007’* | 15.03.2007 |
| ‘Late spring’ | 15.05.xxxx | *‘Late spring 2007’* | 15.05.2007 |
| ‘Summer’ | 15.07.xxxx | *‘Summer 2007’* | 15.07.2007 |
| ‘Early Summer’ | 15.06.xxxx | *‘Early Summer 2007’* | 15.06.2007 |
| ‘Late Summer’ | 15.08.xxxx | *‘Late Summer 2007’* | 15.08.2007 |
| ‘Autumn’ | 15.10.xxxx | *‘Autumn 2007’* | 15.10.2007 |
| ‘Early Autumn’ | 15.09.xxxx | *‘Early Autumn 2007’* | 15.09.2007 |
| ‘Late Autumn’ | 15.11.xxxx | *‘Late Autumn 2007’* | 15.11.2007 |
| ‘Winter’ | 15.01.xxxx | *‘Winter 2007’* | 15.01.2007 |
| ‘Early Winter’ | 15.12.xxxx | *‘Early Winter 2007’* | 15.12.2007 |
| ‘Late Winter’ | 15.02.xxxx | *‘Late Winter 2007’* | 15.02.2007 |
| ‘Good Friday’ | Recode to exact date | *‘Good Friday 2009’* | 10.04.2009 |
| Easter | Recode to exact date | *‘Easter 2009’* | 12.04.2009 |
| Xmas | Recode to exact date | *‘Xmas 2009’* | 25.12.2009 |
| Before Xmas | 15.12.xxxx | *Before Xmas 2009* | 15.12.2009 |
| After Xmas | 31.12.xxxx | *After Xmas 2009* | 31.12.2009 |
| **Other** |  |  |  |
| ‘Week beginning…’ | Code as that date | *‘Week beginning 15.02.07’* | 15.02.2007 |
| ‘Unclassified’ |  | *e.g. illegible* | 99/99/9999 |
