# Supplementary material for: Patient-reported measurement of time to diagnosis in cancer: development of the Cancer Symptom Interval Measure (C-SIM) and randomised controlled trial of method of delivery
Source: BMC Health Serv Res. 2014 Jan 3;14:3. doi: 10.1186/1472-6963-14-3 (PMC3922822; doi:10.1186/1472-6963-14-3)
Supplement: Additional file 1 — The colorectal tool. [file 1472-6963-14-3-S1.pdf]

57727

PID - 1

Ymddiriedolaeth GIG Gogledd Orllewin Cymru
North West Wales NHS Trust

NHS WALES GIG CYMRU
Ymddiriedolaeth GIG Gogledd Cymru
North Wales NHS Trust

☐ GLAN CLWYD

CID - ☐ GWYNEDD

☐ MAELOR

Confidential Questionnaire

Looking At Your Symptoms Study

This Study has been funded by Cancer Research UK

Cardiff University / Prifysgol Caerdydd

Ysgol Glinigol Gogledd Cymru / North Wales Clinical School

Cancer Research UK

sewtu — South East Wales Trials Unit / Uned Ymchwil De-ddwyrain Cymru

Dear Patient

I am very grateful to you for agreeing to help us with this study.

I hope the information you supply will help us give people a diagnosis earlier.

If you feel you would like to talk to someone about this questionnaire at a later stage, please speak to the doctor or nurse who is looking after you, or be in touch with the research team, whose contact details are on your patient information sheet.

I can assure you that anything you tell us will be treated in the utmost confidence.

Yours sincerely

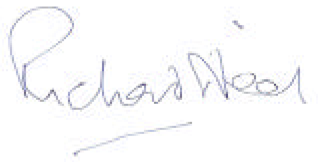

Dr Richard Neal
Senior Clinical Lecturer
Cardiff University
Chief Investigator, Symptoms Study

About your symptoms

These questions are about symptoms you noticed before your diagnosis. We are interested in symptoms which you think are related to your cancer.

Please put a cross in the appropriate box, or write an answer as required.

Looking back...

1. Did you have any symptoms that you feel were due to your cancer before you were diagnosed?

Yes ☐ **Please continue with Question 2**

No ☐ **Please go to Section 2 on Page 5**

2. What was the first symptom that made you think something might be wrong?

☐

Please go to Question 3

The following questions are about when you first noticed a symptom and when you first told your GP or nurse about it.

Please give an exact date if you can. Otherwise give your best estimate. (For example approximately how long ago, the month or season). You wish to refer to your diary or calendar if you have it with you.

Here is an example question:

Decrease in appetite

Did you have this symptom?

Yes ☒ **Please complete A then B or C below**

No ☐ **Please go to Section 2**

A When did you first notice this? d d / m m / y y **OR** Estimate: '3 months ago' or 'June'

B When did you first tell your GP or nurse? 2 3 / 1 2 / 0 7 **OR** Estimate

OR

C Put a cross here if you didn't tell your GP or nurse ☐

3. Change in bowel habit

Did you have this symptom?

Yes ☐ **Please complete A then B or C below**

No ☐ **Please go to Question 4**

A When did you first notice this? ☐☐ / ☐☐ / ☐☐ **OR** ☐

B When did you first tell your GP or nurse? ☐☐ / ☐☐ / ☐☐ **OR** ☐

OR

C Put a cross here if you didn't tell your GP or nurse ☐

4. Bleeding from back passage

Did you have this symptom?

Yes ☐ **Please complete A then B or C below**

No ☐ **Please go to Section 2**

A When did you first notice this? ☐☐ / ☐☐ / ☐☐ **OR** ☐

B When did you first tell your GP or nurse? ☐☐ / ☐☐ / ☐☐ **OR** ☐

OR

C Put a cross here if you didn't tell your GP or nurse ☐

Section 2

Please give an <u>exact</u> date if you can. Otherwise give your best <u>estimate</u> (for example approximately how long ago, the month or the season). You may wish to refer to your diary or calendar if you have it with you.

Did you have any of the following?

1. Decrease in appetite

Did you have this?

Yes ☐ **Please complete A then B or C below**

No ☐ **Please go to Question 2**

A When did you <u>first notice</u> this? ☐☐ / ☐☐ / ☐☐ **OR** ☐

B When did you <u>first tell</u> your GP or nurse? ☐☐ / ☐☐ / ☐☐ **OR** ☐

OR

C Put a cross here if you <u>didn't tell</u> your GP or nurse ☐

2. Unexplained weight loss

Did you have this?

Yes ☐ **Please complete A then B or C below**

No ☐ **Please go to Question 3**

A When did you <u>first notice</u> this? ☐☐ / ☐☐ / ☐☐ **OR** ☐

B When did you <u>first tell</u> your GP or nurse? ☐☐ / ☐☐ / ☐☐ **OR** ☐

OR

C Put a cross here if you <u>didn't tell</u> your GP or nurse ☐

3. Fatigue or tiredness that is unusual for you

Did you have this?

Yes ☐ **Please complete A then B or C below**

No ☐ **Please go to Question 4**

A When did you first notice this? ☐☐ / ☐☐ / ☐☐ **OR** ☐

B When did you first tell your GP or nurse? ☐☐ / ☐☐ / ☐☐ **OR** ☐

OR

C Put a cross here if you didn't tell your GP or nurse ☐

4. Feeling different 'in yourself' from usual

Did you have this?

Yes ☐ **Please complete A then B or C below**

No ☐ **Please go to Question 5**

A When did you first notice this? ☐☐ / ☐☐ / ☐☐ **OR** ☐

B When did you first tell your GP or nurse? ☐☐ / ☐☐ / ☐☐ **OR** ☐

OR

C Put a cross here if you didn't tell your GP or nurse ☐

Please try and describe what the feeling was

If you had any further symptoms which you feel are relevant to your cancer diagnosis, please list them below. If not, please go to Question 7.

Please describe the symptom here and complete A then B or C below

5.

**A**	When did you first notice this?	__ __ / __ __ / __ __ **OR**
**B**	When did you first tell your GP or nurse?	__ __ / __ __ / __ __ **OR**
	**OR**	
**C**	Put a cross here if you didn't tell your GP or nurse	☐

If you had another symptom you feel is relevant, please write it below, otherwise go to Question 7.

Please describe the symptom here and complete A then B or C below

6.

**A**	When did you first notice this?	__ __ / __ __ / __ __ **OR**
**B**	When did you first tell your GP or nurse?	__ __ / __ __ / __ __ **OR**
	**OR**	
**C**	Put a cross here if you didn't tell your GP or nurse	☐

If you had further symptoms you would like to tell us about, there is space at the end of the Questionnaire for you to write them.

Did your GP send you for any tests?

Thinking back to the time immediately before you diagnosis, did your GP send you for any of the following tests?

Please cross the appropriate boxes

7. Were you sent for any of these?

	Yes	Not sure	No
Blood test(s)	☐	☐	☐
CT Scan	☐	☐	☐
Ultrasound scan	☐	☐	☐
Barium Enema	☐	☐	☐
Sigmoidoscopy or colonoscopy (Looking at bowel with internal camera)	☐	☐	☐
X-ray	☐	☐	☐

Please go to Question 8

About you

The next questions relate to general information about you. It would help us to know more about the background of the people completing the questionnaire.

8. Which best describes your employment status?

Please cross one box only

- Employed full-time ☐
- Employed part-time ☐
- Self employed full-time ☐
- Self employed part-time ☐
- Unemployed (seeking work) ☐
- Unemployed (not seeking work) ☐
- Retired ☐
- Student ☐
- Permanently sick/disabled ☐
- Temporarily sick/disabled ☐
- Looking after family/home ☐
- Other, please describe ________

9. What is your highest level of qualification?

Please cross one box only

- Degree (or equivalent) ☐
- Diploma (or equivalent) ☐
- 'A' level ☐
- GCSE / 'O' level ☐
- None ☐
- Other, please specify ________

10. How would you describe your ethnicity

Please cross one box only

White ☐
or
- White British ☐
- White Irish ☐
- Other White background ☐

Mixed ☐
or
- White & Black Carribean ☐
- White & Black African ☐
- White & Asian ☐
- Other mixed background ☐

Black or Black British ☐
or
- Carribean ☐
- African ☐
- Other Black background ☐

Asian or Asian British ☐
or
- Indian ☐
- Pakistani ☐
- Bangladeshi ☐
- Other Asian background ☐

Chinese or other ethnic group ☐
or
- Chinese ☐
- Other ethnic group ☐

11.

Do you live alone?

Yes ☐ **Please go to Question 12**

No ☐ **Please say who you live with below**

Who do you live with?
(e.g. wife, husband, partner, family member)

12. Are you suffering from, or have you suffered from, any of the following in the past 2 years?

Please cross any that are applicable to you

- Asthma ☐
- Chronic Obstructive Pulmonary Disease (COPD) ☐
- Other lung disease (e.g. fibrosis, bronchiectatis etc) ☐
- Heart disease ☐
- Anxiety or depression ☐
- Inflammatory bowel disease ☐
- Irritable bowel syndrome ☐
- Peptic ulcer ☐
- Previous cancer ☐
- Diabetes ☐
- Arthritis ☐

13. About smoking

Please cross the appropriate statement

- Are you a current smoker? ☐
- Are you an ex-smoker? ☐
- Are you a non-smoker (never smoked)? ☐

14. Do you think you were more at risk of getting cancer because of your family history?

Yes ☐
No ☐

15. **The following questions are about how completing the questionnaire made you feel.**

A number of statements which people have used to describe themselves are given below. Read each statement and then cross the most appropriate box to indicate how you feel right now, at the moment. There are no right or wrong answers. Do not spend too much time on any one statement but give the answer which seems to describe your present feeling best.

		Not at all	Somewhat	Moderately	Very much
1.	I feel calm	☐	☐	☐	☐
2.	I am tense	☐	☐	☐	☐
3.	I feel upset	☐	☐	☐	☐
4.	I am relaxed	☐	☐	☐	☐
5.	I feel content	☐	☐	☐	☐
6.	I am worried	☐	☐	☐	☐

Please make sure you have answered all the questions.

If you have any other comments or there is anything else you would like to tell us about when you first noticed your symptoms, please do so using the space below.

If you feel you would like to talk to someone about this questionnaire at a later stage, please speak to the doctor or nurse who is looking after you, or be in touch with the research team, whose contact details are on your patient information sheet

Thank you for completing the questionnaire

Please give it to the research nurse

The next page is for the Researcher to complete

Researcher - Post-completion Sheet **Researcher initials:** ☐☐☐

Date questionnaire completed: Date ☐☐ / ☐☐ / ☐☐ Time ☐☐:☐☐

If not completed, give reason ☐

How long is it since the patient was told of his/her diagnosis? ☐

How long did it take the patient to complete the questionnaire?

- Less than 5 minutes ☐
- Between 5 and 10 minutes ☐
- Between 11 and 15 minutes ☐
- Longer than 15 minutes ☐

For the patient selected to complete the questionnaire on their own

Did the patient ask for any help? ☐ Yes ☐ No

If yes,

please specify what help was requested

____

____

please specify what help was given

____

____

For the patient selected for the researcher-administered questionnaire

Did the patient find any of the questions difficult? ☐ Yes ☐ No

If yes,

please specify which questions and what the difficulty was

____

____

____

____

Was the patient made anxious? ☐ Yes ☐ No

If yes,

please give details

____

____

____

____

Where did the patient complete the questionnaire?
(Please specify whether this was in a separate room alone, in a corner of the clinic, the ward or other

____

Thank you. Please now send the questionnaire and patient information sheet and consent form back to Cardiff University in the freepost envelope provided.

57727

PID - | | | 2 |

Ymddiriedolaeth GIG Gogledd Orllewin Cymru
North West Wales NHS Trust

NHS WALES GIG CYMRU
Ymddiriedolaeth GIG Gogledd Cymru
North Wales NHS Trust

CID - ☐ GLAN CLWYD
☐ GWYNEDD
☐ MAELOR

Confidential Questionnaire

Looking At Your Symptoms Study

This Study has been funded by Cancer Research UK

Cardiff University / Prifysgol Caerdydd

Ysgol Glinigol Gogledd Cymru / North Wales Clinical School

Cancer Research UK

sewtu South East Wales Trials Unit / Uned Ymchwil De-ddwyrain Cymru

Dear Patient

I am very grateful to you for agreeing to help us with this study.

I hope the information you supply will help us give people a diagnosis earlier.

If you feel you would like to talk to someone about this questionnaire at a later stage, please speak to the doctor or nurse who is looking after you, or be in touch with the research team, whose contact details are on your patient information sheet.

I can assure you that anything you tell us will be treated in the utmost confidence.

Yours sincerely

Dr Richard Neal
Senior Clinical Lecturer
Cardiff University
Chief Investigator, Symptoms Study

About your symptoms

These questions are about symptoms you noticed before your diagnosis. We are interested in symptoms which you think are related to your cancer.

Please put a cross in the appropriate box, or write an answer as required.

Looking back...

1. Did you have any symptoms that you feel were due to your cancer before you were diagnosed?

- Yes ☐ **Please continue with Question 2**
- No ☐ **Please go to Section 2 on Page 5**

2. What was the first symptom that made you think something might be wrong?

[ ]

Please go to Question 3

The following questions are about when you first noticed a symptom and when you first told your GP or nurse about it.

Please give an exact date if you can. Otherwise give your best estimate. (For example approximately how long ago, the month or season). You wish to refer to your diary or calendar if you have it with you.

Here is an example question:

Decrease in appetite

Did you have this symptom?

- Yes ☒ **Please complete A then B or C below**
- No ☐ **Please go to Section 2**

A When did you first notice this? d d / m m / y y **OR** Estimate '3 months ago' or 'June'

B When did you first tell your GP or nurse? 2 3 / 1 2 / 0 7 **OR** Estimate

OR

C Put a cross here if you didn't tell your GP or nurse ☐

3. Change in bowel habit

Did you have this symptom?

Yes ☐ **Please complete A then B or C below**

No ☐ **Please go to Question 4**

A When did you <u>first notice</u> this? __ __ / __ __ / __ __ **OR** ________

B When did you <u>first tell</u> your GP or nurse? __ __ / __ __ / __ __ **OR** ________

OR

C Put a cross here if you <u>didn't tell</u> your GP or nurse ☐

4. Bleeding from back passage

Did you have this symptom?

Yes ☐ **Please complete A then B or C below**

No ☐ **Please go to Section 2**

A When did you <u>first notice</u> this? __ __ / __ __ / __ __ **OR** ________

B When did you <u>first tell</u> your GP or nurse? __ __ / __ __ / __ __ **OR** ________

OR

C Put a cross here if you <u>didn't tell</u> your GP or nurse ☐

Section 2

Please give an exact date if you can. Otherwise give your best estimate (for example approximately how long ago, the month or the season). You may wish to refer to your diary or calendar if you have it with you.

Did you have any of the following?

1. Decrease in appetite

Did you have this?

Yes ☐ **Please complete A then B or C below**

No ☐ **Please go to Question 2**

A When did you first notice this? ☐☐ / ☐☐ / ☐☐ **OR** ☐

B When did you first tell your GP or nurse? ☐☐ / ☐☐ / ☐☐ **OR** ☐

OR

C Put a cross here if you didn't tell your GP or nurse ☐

2. Unexplained weight loss

Did you have this?

Yes ☐ **Please complete A then B or C below**

No ☐ **Please go to Question 3**

A When did you first notice this? ☐☐ / ☐☐ / ☐☐ **OR** ☐

B When did you first tell your GP or nurse? ☐☐ / ☐☐ / ☐☐ **OR** ☐

OR

C Put a cross here if you didn't tell your GP or nurse ☐

3. **Fatigue or tiredness that is unusual for you**

Did you have this?

Yes ☐ **Please complete A then B or C below**

No ☐ **Please go to Question 4**

A When did you first notice this? ☐☐ / ☐☐ / ☐☐ **OR** ☐

B When did you first tell your GP or nurse? ☐☐ / ☐☐ / ☐☐ **OR** ☐

OR

C Put a cross here if you didn't tell your GP or nurse ☐

4. **Feeling different 'in yourself' from usual**

Did you have this?

Yes ☐ **Please complete A then B or C below**

No ☐ **Please go to Question 5**

A When did you first notice this? ☐☐ / ☐☐ / ☐☐ **OR** ☐

B When did you first tell your GP or nurse? ☐☐ / ☐☐ / ☐☐ **OR** ☐

OR

C Put a cross here if you didn't tell your GP or nurse ☐

Please try and describe what the feeling was

If you had any further symptoms which you feel are relevant to your cancer diagnosis, please list them below. If not, please go to Question 7.

Please describe the symptom here and complete A then B or C below

5.

**A**	When did you first notice this?	__ __ / __ __ / __ __ **OR**
**B**	When did you first tell your GP or nurse?	__ __ / __ __ / __ __ **OR**
	**OR**	
**C**	Put a cross here if you didn't tell your GP or nurse	☐

If you had another symptom you feel is relevant, please write it below, otherwise go to Question 7.

Please describe the symptom here and complete A then B or C below

6.

**A**	When did you first notice this?	__ __ / __ __ / __ __ **OR**
**B**	When did you first tell your GP or nurse?	__ __ / __ __ / __ __ **OR**
	**OR**	
**C**	Put a cross here if you didn't tell your GP or nurse	☐

If you had further symptoms you would like to tell us about, there is space at the end of the Questionnaire for you to write them.

Did your GP send you for any tests?

Thinking back to the time immediately before you diagnosis, did your GP send you for any of the following tests?

Please cross the appropriate boxes

7. Were you sent for any of these?

	Yes	Not sure	No
Blood test(s)	☐	☐	☐
CT Scan	☐	☐	☐
Ultrasound scan	☐	☐	☐
Barium Enema	☐	☐	☐
Sigmoidoscopy or colonoscopy (Looking at bowel with internal camera)	☐	☐	☐
X-ray	☐	☐	☐

Please go to Question 8

About you

The next questions relate to general information about you. It would help us to know more about the background of the people completing the questionnaire.

8. Which best describes your employment status?

Please cross one box only

- Employed full-time ☐
- Employed part-time ☐
- Self employed full-time ☐
- Self employed part-time ☐
- Unemployed (seeking work) ☐
- Unemployed (not seeking work) ☐
- Retired ☐
- Student ☐
- Permanently sick/disabled ☐
- Temporarily sick/disabled ☐
- Looking after family/home ☐
- Other, please describe ____

9. What is your highest level of qualification?

Please cross one box only

- Degree (or equivalent) ☐
- Diploma (or equivalent) ☐
- 'A' level ☐
- GCSE / 'O' level ☐
- None ☐
- Other, please specify ____

10. How would you describe your ethnicity

Please cross one box only

White ☐
or
- White British ☐
- White Irish ☐
- Other White background ☐

Mixed ☐
or
- White & Black Carribean ☐
- White & Black African ☐
- White & Asian ☐
- Other mixed background ☐

Black or Black British ☐
or
- Carribean ☐
- African ☐
- Other Black background ☐

Asian or Asian British ☐
or
- Indian ☐
- Pakistani ☐
- Bangladeshi ☐
- Other Asian background ☐

Chinese or other ethnic group ☐
or
- Chinese ☐
- Other ethnic group ☐

11.

Do you live alone?

Yes ☐ **Please go to Question 12**

No ☐ **Please say who you live with below**

Who do you live with?
(e.g. wife, husband, partner, family member)

12. Are you suffering from, or have you suffered from, any of the following in the past 2 years?

Please cross any that are applicable to you

- Asthma ☐
- Chronic Obstructive Pulmonary Disease (COPD) ☐
- Other lung disease (e.g. fibrosis, bronchiectatis etc) ☐
- Heart disease ☐
- Anxiety or depression ☐
- Inflammatory bowel disease ☐
- Irritable bowel syndrome ☐
- Peptic ulcer ☐
- Previous cancer ☐
- Diabetes ☐
- Arthritis ☐

13. About smoking

Please cross the appropriate statement

- Are you a current smoker? ☐
- Are you an ex-smoker? ☐
- Are you a non-smoker (never smoked)? ☐

14. Do you think you were more at risk of getting cancer because of your family history?

Yes ☐

No ☐

15. The following questions are about how completing the questionnaire made you feel.

A number of statements which people have used to describe themselves are given below. Read each statement and then cross the most appropriate box to indicate how you feel right now, at the moment. There are no right or wrong answers. Do not spend too much time on any one statement but give the answer which seems to describe your present feeling best.

		Not at all	Somewhat	Moderately	Very much
1.	I feel calm	☐	☐	☐	☐
2.	I am tense	☐	☐	☐	☐
3.	I feel upset	☐	☐	☐	☐
4.	I am relaxed	☐	☐	☐	☐
5.	I feel content	☐	☐	☐	☐
6.	I am worried	☐	☐	☐	☐

Please make sure you have answered all the questions.

If you have any other comments or there is anything else you would like to tell us about when you first noticed your symptoms, please do so using the space below.

If you feel you would like to talk to someone about this questionnaire at a later stage, please speak to the doctor or nurse who is looking after you, or be in touch with the research team, whose contact details are on your patient information sheet

Thank you for completing the questionnaire

Please give it to the research nurse

The next page is for the Researcher to complete

Researcher - Post-completion Sheet

Researcher initials: ☐☐☐

Date questionnaire completed: Date ☐☐ / ☐☐ / ☐☐ Time ☐☐:☐☐

If not completed, give reason ☐

How long is it since the patient was told of his/her diagnosis? ☐

How long did it take the patient to complete the questionnaire?

- Less than 5 minutes ☐
- Between 5 and 10 minutes ☐
- Between 11 and 15 minutes ☐
- Longer than 15 minutes ☐

For the patient selected to complete the questionnaire on their own

Did the patient ask for any help? ☐ Yes ☐ No

If yes,

please specify what help was requested

please specify what help was given

For the patient selected for the researcher-administered questionnaire

Did the patient find any of the questions difficult? ☐ Yes ☐ No

If yes,

please specify which questions and what the difficulty was

Was the patient made anxious? ☐ Yes ☐ No

If yes,

please give details

Where did the patient complete the questionnaire?

(Please specify whether this was in a separate room alone, in a corner of the clinic, the ward or other

Thank you. Please now send the questionnaire and patient information sheet and consent form back to Cardiff University in the freepost envelope provided.

57727

PID - | | | 3 |

Ymddiriedolaeth GIG Gogledd Orllewin Cymru
North West Wales NHS Trust

NHS WALES GIG CYMRU
Ymddiriedolaeth GIG Gogledd Cymru
North Wales NHS Trust

CID -

☐ GLAN CLWYD

☐ GWYNEDD

☐ MAELOR

Confidential Questionnaire

Looking At Your Symptoms Study

This Study has been funded by Cancer Research UK

Cardiff University / Prifysgol Caerdydd

Ysgol Glinigol Gogledd Cymru
North Wales Clinical School

Cancer Research UK

sewtu
South East Wales Trials Unit
Uned Ymchwil De-ddwyrain Cymru

Dear Patient

I am very grateful to you for agreeing to help us with this study.

I hope the information you supply will help us give people a diagnosis earlier.

If you feel you would like to talk to someone about this questionnaire at a later stage, please speak to the doctor or nurse who is looking after you, or be in touch with the research team, whose contact details are on your patient information sheet.

I can assure you that anything you tell us will be treated in the utmost confidence.

Yours sincerely

Dr Richard Neal
Senior Clinical Lecturer
Cardiff University
Chief Investigator, Symptoms Study

About your symptoms

These questions are about symptoms you noticed before your diagnosis. We are interested in symptoms which you think are related to your cancer.

Please put a cross in the appropriate box, or write an answer as required.

Looking back...

1. Did you have any symptoms that you feel were due to your cancer before you were diagnosed?

Yes ☐ **Please continue with Question 2**

No ☐ **Please go to Section 2 on Page 5**

2. What was the first symptom that made you think something might be wrong?

☐

Please go to Question 3

The following questions are about when you first noticed a symptom and when you first told your GP or nurse about it.

Please give an exact date if you can. Otherwise give your best estimate. (For example approximately how long ago, the month or season). You wish to refer to your diary or calendar if you have it with you.

Here is an example question:

Decrease in appetite

Did you have this symptom?

Yes ☒ **Please complete A then B or C below**

No ☐ **Please go to Section 2**

A When did you first notice this? d d / m m / y y **OR** Estimate: '3 months ago' or 'June'

B When did you first tell your GP or nurse? 2 3 / 1 2 / 0 7 **OR** Estimate

OR

C Put a cross here if you didn't tell your GP or nurse ☐

3. Change in bowel habit

Did you have this symptom?

Yes ☐ **Please complete A then B or C below**

No ☐ **Please go to Question 4**

A When did you first notice this? ☐☐ / ☐☐ / ☐☐ **OR** ☐

B When did you first tell your GP or nurse? ☐☐ / ☐☐ / ☐☐ **OR** ☐

OR

C Put a cross here if you didn't tell your GP or nurse ☐

4. Bleeding from back passage

Did you have this symptom?

Yes ☐ **Please complete A then B or C below**

No ☐ **Please go to Section 2**

A When did you first notice this? ☐☐ / ☐☐ / ☐☐ **OR** ☐

B When did you first tell your GP or nurse? ☐☐ / ☐☐ / ☐☐ **OR** ☐

OR

C Put a cross here if you didn't tell your GP or nurse ☐

Section 2

Please give an <u>exact</u> date if you can. Otherwise give your best <u>estimate</u> (for example approximately how long ago, the month or the season). You may wish to refer to your diary or calendar if you have it with you.

Did you have any of the following?

1. Decrease in appetite

Did you have this?

Yes ☐ **Please complete A then B or C below**

No ☐ **Please go to Question 2**

A When did you first notice this? ☐☐ / ☐☐ / ☐☐ **OR** ☐

B When did you first tell your GP or nurse? ☐☐ / ☐☐ / ☐☐ **OR** ☐

OR

C Put a cross here if you didn't tell your GP or nurse ☐

2. Unexplained weight loss

Did you have this?

Yes ☐ **Please complete A then B or C below**

No ☐ **Please go to Question 3**

A When did you first notice this? ☐☐ / ☐☐ / ☐☐ **OR** ☐

B When did you first tell your GP or nurse? ☐☐ / ☐☐ / ☐☐ **OR** ☐

OR

C Put a cross here if you didn't tell your GP or nurse ☐

3. **Fatigue or tiredness that is unusual for you**

Did you have this?

Yes ☐ **Please complete A then B or C below**

No ☐ **Please go to Question 4**

A When did you first notice this? ☐☐ / ☐☐ / ☐☐ **OR** ☐

B When did you first tell your GP or nurse? ☐☐ / ☐☐ / ☐☐ **OR** ☐

OR

C Put a cross here if you didn't tell your GP or nurse ☐

4. **Feeling different 'in yourself' from usual**

Did you have this?

Yes ☐ **Please complete A then B or C below**

No ☐ **Please go to Question 5**

A When did you first notice this? ☐☐ / ☐☐ / ☐☐ **OR** ☐

B When did you first tell your GP or nurse? ☐☐ / ☐☐ / ☐☐ **OR** ☐

OR

C Put a cross here if you didn't tell your GP or nurse ☐

Please try and describe what the feeling was

If you had any further symptoms which you feel are relevant to your cancer diagnosis, please list them below. If not, please go to Question 7.

Please describe the symptom here and complete A then B or C below

5.

**A**	When did you first notice this?	__ __ / __ __ / __ __ **OR**
**B**	When did you first tell your GP or nurse?	__ __ / __ __ / __ __ **OR**
	**OR**	
**C**	Put a cross here if you didn't tell your GP or nurse	☐

If you had another symptom you feel is relevant, please write it below, otherwise go to Question 7.

Please describe the symptom here and complete A then B or C below

6.

**A**	When did you first notice this?	__ __ / __ __ / __ __ **OR**
**B**	When did you first tell your GP or nurse?	__ __ / __ __ / __ __ **OR**
	**OR**	
**C**	Put a cross here if you didn't tell your GP or nurse	☐

If you had further symptoms you would like to tell us about, there is space at the end of the Questionnaire for you to write them.

Did your GP send you for any tests?

Thinking back to the time immediately before you diagnosis, did your GP send you for any of the following tests?

Please cross the appropriate boxes

7. Were you sent for any of these?

	Yes	Not sure	No
Blood test(s)	☐	☐	☐
CT Scan	☐	☐	☐
Ultrasound scan	☐	☐	☐
Barium Enema	☐	☐	☐
Sigmoidoscopy or colonoscopy (Looking at bowel with internal camera)	☐	☐	☐
X-ray	☐	☐	☐

Please go to Question 8

About you

The next questions relate to general information about you. It would help us to know more about the background of the people completing the questionnaire.

8. Which best describes your employment status?

Please cross one box only

- Employed full-time ☐
- Employed part-time ☐
- Self employed full-time ☐
- Self employed part-time ☐
- Unemployed (seeking work) ☐
- Unemployed (not seeking work) ☐
- Retired ☐
- Student ☐
- Permanently sick/disabled ☐
- Temporarily sick/disabled ☐
- Looking after family/home ☐
- Other, please describe ______

9. What is your highest level of qualification?

Please cross one box only

- Degree (or equivalent) ☐
- Diploma (or equivalent) ☐
- 'A' level ☐
- GCSE / 'O' level ☐
- None ☐
- Other, please specify ______

10. How would you describe your ethnicity

Please cross one box only

White ☐
or
- White British ☐
- White Irish ☐
- Other White background ☐

Mixed ☐
or
- White & Black Carribean ☐
- White & Black African ☐
- White & Asian ☐
- Other mixed background ☐

Black or Black British ☐
or
- Carribean ☐
- African ☐
- Other Black background ☐

Asian or Asian British ☐
or
- Indian ☐
- Pakistani ☐
- Bangladeshi ☐
- Other Asian background ☐

Chinese or other ethnic group ☐
or
- Chinese ☐
- Other ethnic group ☐

11.

Do you live alone?

Yes ☐ **Please go to Question 12**

No ☐ **Please say who you live with below**

Who do you live with?
(e.g. wife, husband, partner, family member)

12. **Are you suffering from, or have you suffered from, any of the following in the past 2 years?**

Please cross any that are applicable to you

- Asthma ☐
- Chronic Obstructive Pulmonary Disease (COPD) ☐
- Other lung disease (e.g. fibrosis, bronchiectatis etc) ☐
- Heart disease ☐
- Anxiety or depression ☐
- Inflammatory bowel disease ☐
- Irritable bowel syndrome ☐
- Peptic ulcer ☐
- Previous cancer ☐
- Diabetes ☐
- Arthritis ☐

13. **About smoking**

Please cross the appropriate statement

- Are you a current smoker? ☐
- Are you an ex-smoker? ☐
- Are you a non-smoker (never smoked)? ☐

14. **Do you think you were more at risk of getting cancer because of your family history?**

Yes ☐
No ☐

15. The following questions are about how completing the questionnaire made you feel.

A number of statements which people have used to describe themselves are given below. Read each statement and then cross the most appropriate box to indicate how you feel right now, at the moment. There are no right or wrong answers. Do not spend too much time on any one statement but give the answer which seems to describe your present feeling best.

		Not at all	Somewhat	Moderately	Very much
1.	I feel calm	☐	☐	☐	☐
2.	I am tense	☐	☐	☐	☐
3.	I feel upset	☐	☐	☐	☐
4.	I am relaxed	☐	☐	☐	☐
5.	I feel content	☐	☐	☐	☐
6.	I am worried	☐	☐	☐	☐

Please make sure you have answered all the questions.

If you have any other comments or there is anything else you would like to tell us about when you first noticed your symptoms, please do so using the space below.

If you feel you would like to talk to someone about this questionnaire at a later stage, please speak to the doctor or nurse who is looking after you, or be in touch with the research team, whose contact details are on your patient information sheet

Thank you for completing the questionnaire

Please give it to the research nurse

The next page is for the Researcher to complete

57727

3

Researcher - Post-completion Sheet

Researcher initials: ☐☐☐

Date questionnaire completed: Date ☐☐ / ☐☐ / ☐☐ Time ☐☐:☐☐

If not completed, give reason ☐

How long is it since the patient was told of his/her diagnosis? ☐

How long did it take the patient to complete the questionnaire?

- Less than 5 minutes ☐
- Between 5 and 10 minutes ☐
- Between 11 and 15 minutes ☐
- Longer than 15 minutes ☐

For the patient selected to <u>complete the questionnaire on their own</u>

Did the patient ask for any help? ☐ Yes ☐ No

If yes,

please specify what help was requested

____

____

please specify what help was given

____

____

For the patient selected for <u>the researcher-administered questionnaire</u>

Did the patient find any of the questions difficult? ☐ Yes ☐ No

If yes,

please specify which questions and what the difficulty was

____

____

____

____

Was the patient made anxious? ☐ Yes ☐ No

If yes,

please give details

____

____

____

____

Where did the patient complete the questionnaire?
(Please specify whether this was in a separate room alone, in a corner of the clinic, the ward or other

____

Thank you. Please now send the questionnaire and patient information sheet and consent form back to Cardiff University in the freepost envelope provided.

57727

PID - | | | 4 |

Ymddiriedolaeth GIG Gogledd Orllewin Cymru
North West Wales NHS Trust

NHS WALES GIG CYMRU
Ymddiriedolaeth GIG Gogledd Cymru
North Wales NHS Trust

CID - ☐ GLAN CLWYD
☐ GWYNEDD
☐ MAELOR

Confidential Questionnaire

Looking At Your Symptoms Study

This Study has been funded by Cancer Research UK

Cardiff University Prifysgol Caerdydd
Ysgol Glinigol Gogledd Cymru North Wales Clinical School
Cancer Research UK
sewtu South East Wales Trials Unit Uned Ymchwil De-ddwyrain Cymru

Dear Patient

I am very grateful to you for agreeing to help us with this study.

I hope the information you supply will help us give people a diagnosis earlier.

If you feel you would like to talk to someone about this questionnaire at a later stage, please speak to the doctor or nurse who is looking after you, or be in touch with the research team, whose contact details are on your patient information sheet.

I can assure you that anything you tell us will be treated in the utmost confidence.

Yours sincerely

Richard Neal

Dr Richard Neal
Senior Clinical Lecturer
Cardiff University
Chief Investigator, Symptoms Study

About your symptoms

These questions are about symptoms you noticed before your diagnosis. We are interested in symptoms which you think are related to your cancer.

Please put a cross in the appropriate box, or write an answer as required.

Looking back...

1. Did you have any symptoms that you feel were due to your cancer before you were diagnosed?

Yes ☐ **Please continue with Question 2**

No ☐ **Please go to Section 2 on Page 5**

2. What was the first symptom that made you think something might be wrong?

☐

Please go to Question 3

The following questions are about when you first noticed a symptom and when you first told your GP or nurse about it.

Please give an exact date if you can. Otherwise give your best estimate. (For example approximately how long ago, the month or season). You wish to refer to your diary or calendar if you have it with you.

Here is an example question:

Decrease in appetite

Did you have this symptom?

Yes ☒ **Please complete A then B or C below**

No ☐ **Please go to Section 2**

A When did you first notice this? d d / m m / y y **OR** Estimate '3 months ago' or 'June'

B When did you first tell your GP or nurse? 2 3 / 1 2 / 0 7 **OR** Estimate

OR

C Put a cross here if you didn't tell your GP or nurse ☐

3. Change in bowel habit

Did you have this symptom?

Yes ☐ **Please complete A then B or C below**

No ☐ **Please go to Question 4**

A When did you first notice this? ☐☐ / ☐☐ / ☐☐ **OR** ☐

B When did you first tell your GP or nurse? ☐☐ / ☐☐ / ☐☐ **OR** ☐

OR

C Put a cross here if you didn't tell your GP or nurse ☐

4. Bleeding from back passage

Did you have this symptom?

Yes ☐ **Please complete A then B or C below**

No ☐ **Please go to Section 2**

A When did you first notice this? ☐☐ / ☐☐ / ☐☐ **OR** ☐

B When did you first tell your GP or nurse? ☐☐ / ☐☐ / ☐☐ **OR** ☐

OR

C Put a cross here if you didn't tell your GP or nurse ☐

Section 2

Please give an exact date if you can. Otherwise give your best estimate (for example approximately how long ago, the month or the season). You may wish to refer to your diary or calendar if you have it with you.

Did you have any of the following?

1. Decrease in appetite

Did you have this?

Yes ☐ **Please complete A then B or C below**

No ☐ **Please go to Question 2**

A When did you first notice this? ☐☐ / ☐☐ / ☐☐ **OR** ☐

B When did you first tell your GP or nurse? ☐☐ / ☐☐ / ☐☐ **OR** ☐

OR

C Put a cross here if you didn't tell your GP or nurse ☐

2. Unexplained weight loss

Did you have this?

Yes ☐ **Please complete A then B or C below**

No ☐ **Please go to Question 3**

A When did you first notice this? ☐☐ / ☐☐ / ☐☐ **OR** ☐

B When did you first tell your GP or nurse? ☐☐ / ☐☐ / ☐☐ **OR** ☐

OR

C Put a cross here if you didn't tell your GP or nurse ☐

3. **Fatigue or tiredness that is unusual for you**

Did you have this?

Yes ☐ **Please complete A then B or C below**

No ☐ **Please go to Question 4**

A When did you first notice this? ☐☐ / ☐☐ / ☐☐ **OR** ☐

B When did you first tell your GP or nurse? ☐☐ / ☐☐ / ☐☐ **OR** ☐

OR

C Put a cross here if you didn't tell your GP or nurse ☐

4. **Feeling different 'in yourself' from usual**

Did you have this?

Yes ☐ **Please complete A then B or C below**

No ☐ **Please go to Question 5**

A When did you first notice this? ☐☐ / ☐☐ / ☐☐ **OR** ☐

B When did you first tell your GP or nurse? ☐☐ / ☐☐ / ☐☐ **OR** ☐

OR

C Put a cross here if you didn't tell your GP or nurse ☐

Please try and describe what the feeling was

If you had any further symptoms which you feel are relevant to your cancer diagnosis, please list them below. If not, please go to Question 7.

Please describe the symptom here and complete A then B or C below

5.

**A**	When did you first notice this?	__ __ / __ __ / __ __ **OR**
**B**	When did you first tell your GP or nurse?	__ __ / __ __ / __ __ **OR**
	**OR**	
**C**	Put a cross here if you didn't tell your GP or nurse	☐

If you had another symptom you feel is relevant, please write it below, otherwise go to Question 7.

Please describe the symptom here and complete A then B or C below

6.

**A**	When did you first notice this?	__ __ / __ __ / __ __ **OR**
**B**	When did you first tell your GP or nurse?	__ __ / __ __ / __ __ **OR**
	**OR**	
**C**	Put a cross here if you didn't tell your GP or nurse	☐

If you had further symptoms you would like to tell us about, there is space at the end of the Questionnaire for you to write them.

Did your GP send you for any tests?

Thinking back to the time immediately before you diagnosis, did your GP send you for any of the following tests?

Please cross the appropriate boxes

7. Were you sent for any of these?

	Yes	Not sure	No
Blood test(s)	☐	☐	☐
CT Scan	☐	☐	☐
Ultrasound scan	☐	☐	☐
Barium Enema	☐	☐	☐
Sigmoidoscopy or colonoscopy (Looking at bowel with internal camera)	☐	☐	☐
X-ray	☐	☐	☐

Please go to Question 8

About you

The next questions relate to general information about you. It would help us to know more about the background of the people completing the questionnaire.

8. Which best describes your employment status?

Please cross one box only

- Employed full-time ☐
- Employed part-time ☐
- Self employed full-time ☐
- Self employed part-time ☐
- Unemployed (seeking work) ☐
- Unemployed (not seeking work) ☐
- Retired ☐
- Student ☐
- Permanently sick/disabled ☐
- Temporarily sick/disabled ☐
- Looking after family/home ☐

Other, please describe ______

9. What is your highest level of qualification?

Please cross one box only

- Degree (or equivalent) ☐
- Diploma (or equivalent) ☐
- 'A' level ☐
- GCSE / 'O' level ☐
- None ☐

Other, please specify ______

10. **How would you describe your ethnicity**

Please cross one box only

White ☐
or
White British ☐
White Irish ☐
Other White background ☐

Mixed ☐
or
White & Black Carribean ☐
White & Black African ☐
White & Asian ☐
Other mixed background ☐

Black or Black British ☐
or
Carribean ☐
African ☐
Other Black background ☐

Asian or Asian British ☐
or
Indian ☐
Pakistani ☐
Bangladeshi ☐
Other Asian background ☐

Chinese or other ethnic group ☐
or
Chinese ☐
Other ethnic group ☐

11.

Do you live alone?

Yes ☐ **Please go to Question 12**

No ☐ **Please say who you live with below**

Who do you live with?
(e.g. wife, husband, partner, family member)

12. **Are you suffering from, or have you suffered from, any of the following in the past 2 years?**

Please cross any that are applicable to you

- Asthma ☐
- Chronic Obstructive Pulmonary Disease (COPD) ☐
- Other lung disease (e.g. fibrosis, bronchiectatis etc) ☐
- Heart disease ☐
- Anxiety or depression ☐
- Inflammatory bowel disease ☐
- Irritable bowel syndrome ☐
- Peptic ulcer ☐
- Previous cancer ☐
- Diabetes ☐
- Arthritis ☐

13. **About smoking**

Please cross the appropriate statement

- Are you a current smoker? ☐
- Are you an ex-smoker? ☐
- Are you a non-smoker (never smoked)? ☐

14. **Do you think you were more at risk of getting cancer because of your family history?**

Yes ☐
No ☐

15. The following questions are about how completing the questionnaire made you feel.

A number of statements which people have used to describe themselves are given below. Read each statement and then cross the most appropriate box to indicate how you feel right now, at the moment. There are no right or wrong answers. Do not spend too much time on any one statement but give the answer which seems to describe your present feeling best.

		Not at all	Somewhat	Moderately	Very much
1.	I feel calm	☐	☐	☐	☐
2.	I am tense	☐	☐	☐	☐
3.	I feel upset	☐	☐	☐	☐
4.	I am relaxed	☐	☐	☐	☐
5.	I feel content	☐	☐	☐	☐
6.	I am worried	☐	☐	☐	☐

Please make sure you have answered all the questions.

If you have any other comments or there is anything else you would like to tell us about when you first noticed your symptoms, please do so using the space below.

If you feel you would like to talk to someone about this questionnaire at a later stage, please speak to the doctor or nurse who is looking after you, or be in touch with the research team, whose contact details are on your patient information sheet

Thank you for completing the questionnaire

Please give it to the research nurse

The next page is for the Researcher to complete

4

Researcher - Post-completion Sheet

Researcher initials: ☐☐☐

Date questionnaire completed: Date ☐☐ / ☐☐ / ☐☐ Time ☐☐ : ☐☐

If not completed, give reason ☐

How long is it since the patient was told of his/her diagnosis? ☐

How long did it take the patient to complete the questionnaire?

- Less than 5 minutes ☐
- Between 5 and 10 minutes ☐
- Between 11 and 15 minutes ☐
- Longer than 15 minutes ☐

For the patient selected to <u>complete the questionnaire on their own</u>

Did the patient ask for any help? ☐ Yes ☐ No

If yes,

please specify what help was requested

____

____

please specify what help was given

____

____

For the patient selected for <u>the researcher-administered questionnaire</u>

Did the patient find any of the questions difficult? ☐ Yes ☐ No

If yes,

please specify which questions and what the difficulty was

____

____

____

____

Was the patient made anxious? ☐ Yes ☐ No

If yes,

please give details

____

____

____

____

Where did the patient complete the questionnaire?
(Please specify whether this was in a separate room alone, in a corner of the clinic, the ward or other

____

Thank you. Please now send the questionnaire and patient information sheet and consent form back to Cardiff University in the freepost envelope provided.

57727

PID - | | | 5 |

Ymddiriedolaeth GIG Gogledd Orllewin Cymru
North West Wales NHS Trust

NHS WALES GIG CYMRU
Ymddiriedolaeth GIG Gogledd Cymru
North Wales NHS Trust

☐ GLAN CLWYD

CID - ☐ GWYNEDD

☐ MAELOR

Confidential Questionnaire

Looking At Your Symptoms Study

This Study has been funded by Cancer Research UK

Cardiff University Prifysgol Caerdydd

Ysgol Glinigol Gogledd Cymru
North Wales Clinical School

Cancer Research UK

sewtu South East Wales Trials Unit
Uned Ymchwil De-ddwyrain Cymru

Dear Patient

I am very grateful to you for agreeing to help us with this study.

I hope the information you supply will help us give people a diagnosis earlier.

If you feel you would like to talk to someone about this questionnaire at a later stage, please speak to the doctor or nurse who is looking after you, or be in touch with the research team, whose contact details are on your patient information sheet.

I can assure you that anything you tell us will be treated in the utmost confidence.

Yours sincerely

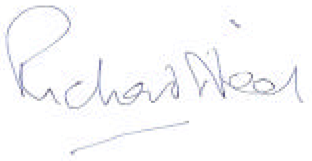

Dr Richard Neal
Senior Clinical Lecturer
Cardiff University
Chief Investigator, Symptoms Study

About your symptoms

These questions are about symptoms you noticed before your diagnosis. We are interested in symptoms which you think are related to your cancer.

Please put a cross in the appropriate box, or write an answer as required.

Looking back...

1. Did you have any symptoms that you feel were due to your cancer before you were diagnosed?

- Yes ☐ **Please continue with Question 2**
- No ☐ **Please go to Section 2 on Page 5**

2. What was the first symptom that made you think something might be wrong?

[ ]

Please go to Question 3

The following questions are about when you first noticed a symptom and when you first told your GP or nurse about it.

Please give an exact date if you can. Otherwise give your best estimate. (For example approximately how long ago, the month or season). You wish to refer to your diary or calendar if you have it with you.

Here is an example question:

Decrease in appetite

Did you have this symptom?

- Yes ☒ **Please complete A then B or C below**
- No ☐ **Please go to Section 2**

A When did you first notice this? d d / m m / y y **OR** Estimate '3 months ago' or 'June'

B When did you first tell your GP or nurse? 2 3 / 1 2 / 0 7 **OR** Estimate

OR

C Put a cross here if you didn't tell your GP or nurse ☐

3. Change in bowel habit

Did you have this symptom?

Yes ☐ **Please complete A then B or C below**

No ☐ **Please go to Question 4**

A When did you first notice this? ☐☐ / ☐☐ / ☐☐ **OR** ☐

B When did you first tell your GP or nurse? ☐☐ / ☐☐ / ☐☐ **OR** ☐

OR

C Put a cross here if you didn't tell your GP or nurse ☐

4. Bleeding from back passage

Did you have this symptom?

Yes ☐ **Please complete A then B or C below**

No ☐ **Please go to Section 2**

A When did you first notice this? ☐☐ / ☐☐ / ☐☐ **OR** ☐

B When did you first tell your GP or nurse? ☐☐ / ☐☐ / ☐☐ **OR** ☐

OR

C Put a cross here if you didn't tell your GP or nurse ☐

Section 2

Please give an exact date if you can. Otherwise give your best estimate (for example approximately how long ago, the month or the season). You may wish to refer to your diary or calendar if you have it with you.

Did you have any of the following?

1. Decrease in appetite

Did you have this?

Yes ☐ **Please complete A then B or C below**

No ☐ **Please go to Question 2**

A When did you first notice this? ☐☐ / ☐☐ / ☐☐ **OR** ☐

B When did you first tell your GP or nurse? ☐☐ / ☐☐ / ☐☐ **OR** ☐

OR

C Put a cross here if you didn't tell your GP or nurse ☐

2. Unexplained weight loss

Did you have this?

Yes ☐ **Please complete A then B or C below**

No ☐ **Please go to Question 3**

A When did you first notice this? ☐☐ / ☐☐ / ☐☐ **OR** ☐

B When did you first tell your GP or nurse? ☐☐ / ☐☐ / ☐☐ **OR** ☐

OR

C Put a cross here if you didn't tell your GP or nurse ☐

3. **Fatigue or tiredness that is unusual for you**

Did you have this?

Yes ☐ **Please complete A then B or C below**

No ☐ **Please go to Question 4**

A When did you <u>first notice</u> this? ☐☐ / ☐☐ / ☐☐ **OR** ☐

B When did you <u>first tell</u> your GP or nurse? ☐☐ / ☐☐ / ☐☐ **OR** ☐

OR

C Put a cross here if you <u>didn't tell</u> your GP or nurse ☐

4. **Feeling different 'in yourself' from usual**

Did you have this?

Yes ☐ **Please complete A then B or C below**

No ☐ **Please go to Question 5**

A When did you <u>first notice</u> this? ☐☐ / ☐☐ / ☐☐ **OR** ☐

B When did you <u>first tell</u> your GP or nurse? ☐☐ / ☐☐ / ☐☐ **OR** ☐

OR

C Put a cross here if you <u>didn't tell</u> your GP or nurse ☐

Please try and describe what the feeling was

If you had any further symptoms which you feel are relevant to your cancer diagnosis, please list them below. If not, please go to Question 7.

Please describe the symptom here and complete A then B or C below

5.

**A**	When did you first notice this?	__ / __ / __ **OR**
**B**	When did you first tell your GP or nurse?	__ / __ / __ **OR**
	**OR**	
**C**	Put a cross here if you didn't tell your GP or nurse	☐

If you had another symptom you feel is relevant, please write it below, otherwise go to Question 7.

Please describe the symptom here and complete A then B or C below

6.

**A**	When did you first notice this?	__ / __ / __ **OR**
**B**	When did you first tell your GP or nurse?	__ / __ / __ **OR**
	**OR**	
**C**	Put a cross here if you didn't tell your GP or nurse	☐

If you had further symptoms you would like to tell us about, there is space at the end of the Questionnaire for you to write them.

Did your GP send you for any tests?

Thinking back to the time immediately before you diagnosis, did your GP send you for any of the following tests?

Please cross the appropriate boxes

7. Were you sent for any of these?

	Yes	Not sure	No
Blood test(s)	☐	☐	☐
CT Scan	☐	☐	☐
Ultrasound scan	☐	☐	☐
Barium Enema	☐	☐	☐
Sigmoidoscopy or colonoscopy (Looking at bowel with internal camera)	☐	☐	☐
X-ray	☐	☐	☐

Please go to Question 8

About you

The next questions relate to general information about you. It would help us to know more about the background of the people completing the questionnaire.

8. Which best describes your employment status?

Please cross one box only

- Employed full-time ☐
- Employed part-time ☐
- Self employed full-time ☐
- Self employed part-time ☐
- Unemployed (seeking work) ☐
- Unemployed (not seeking work) ☐
- Retired ☐
- Student ☐
- Permanently sick/disabled ☐
- Temporarily sick/disabled ☐
- Looking after family/home ☐
- Other, please describe

9. What is your highest level of qualification?

Please cross one box only

- Degree (or equivalent) ☐
- Diploma (or equivalent) ☐
- 'A' level ☐
- GCSE / 'O' level ☐
- None ☐
- Other, please specify

10. **How would you describe your ethnicity**

Please cross one box only

White ☐
or
- White British ☐
- White Irish ☐
- Other White background ☐

Mixed ☐
or
- White & Black Carribean ☐
- White & Black African ☐
- White & Asian ☐
- Other mixed background ☐

Black or Black British ☐
or
- Carribean ☐
- African ☐
- Other Black background ☐

Asian or Asian British ☐
or
- Indian ☐
- Pakistani ☐
- Bangladeshi ☐
- Other Asian background ☐

Chinese or other ethnic group ☐
or
- Chinese ☐
- Other ethnic group ☐

11.

Do you live alone?

Yes ☐ **Please go to Question 12**

No ☐ **Please say who you live with below**

Who do you live with?
(e.g. wife, husband, partner, family member)

12. Are you suffering from, or have you suffered from, any of the following in the past 2 years?

Please cross any that are applicable to you

- Asthma ☐
- Chronic Obstructive Pulmonary Disease (COPD) ☐
- Other lung disease (e.g. fibrosis, bronchiectatis etc) ☐
- Heart disease ☐
- Anxiety or depression ☐
- Inflammatory bowel disease ☐
- Irritable bowel syndrome ☐
- Peptic ulcer ☐
- Previous cancer ☐
- Diabetes ☐
- Arthritis ☐

13. About smoking

Please cross the appropriate statement

- Are you a current smoker? ☐
- Are you an ex-smoker? ☐
- Are you a non-smoker (never smoked)? ☐

14. Do you think you were more at risk of getting cancer because of your family history?

Yes ☐

No ☐

15. The following questions are about how completing the questionnaire made you feel.

A number of statements which people have used to describe themselves are given below. Read each statement and then cross the most appropriate box to indicate how you feel right now, at the moment. There are no right or wrong answers. Do not spend too much time on any one statement but give the answer which seems to describe your present feeling best.

		Not at all	Somewhat	Moderately	Very much
1.	I feel calm	☐	☐	☐	☐
2.	I am tense	☐	☐	☐	☐
3.	I feel upset	☐	☐	☐	☐
4.	I am relaxed	☐	☐	☐	☐
5.	I feel content	☐	☐	☐	☐
6.	I am worried	☐	☐	☐	☐

Please make sure you have answered all the questions.

If you have any other comments or there is anything else you would like to tell us about when you first noticed your symptoms, please do so using the space below.

If you feel you would like to talk to someone about this questionnaire at a later stage, please speak to the doctor or nurse who is looking after you, or be in touch with the research team, whose contact details are on your patient information sheet

Thank you for completing the questionnaire

Please give it to the research nurse

The next page is for the Researcher to complete

57727

5

Researcher - Post-completion Sheet

Researcher initials: ☐☐☐

Date questionnaire completed: Date ☐☐ / ☐☐ / ☐☐ Time ☐☐:☐☐

If not completed, give reason ☐

How long is it since the patient was told of his/her diagnosis? ☐

How long did it take the patient to complete the questionnaire?

- Less than 5 minutes ☐
- Between 5 and 10 minutes ☐
- Between 11 and 15 minutes ☐
- Longer than 15 minutes ☐

For the patient selected to complete the questionnaire on their own

Did the patient ask for any help? ☐ Yes ☐ No

If yes,

please specify what help was requested

____

____

please specify what help was given

____

____

For the patient selected for the researcher-administered questionnaire

Did the patient find any of the questions difficult? ☐ Yes ☐ No

If yes,

please specify which questions and what the difficulty was

____

____

____

____

Was the patient made anxious? ☐ Yes ☐ No

If yes,

please give details

____

____

____

____

Where did the patient complete the questionnaire?

(Please specify whether this was in a separate room alone, in a corner of the clinic, the ward or other

____

Thank you. Please now send the questionnaire and patient information sheet and consent form back to Cardiff University in the freepost envelope provided.

57727

PID - | | | 6 |

Ymddiriedolaeth GIG Gogledd Orllewin Cymru
North West Wales NHS Trust

NHS WALES GIG CYMRU
Ymddiriedolaeth GIG Gogledd Cymru
North Wales NHS Trust

CID -
☐ GLAN CLWYD
☐ GWYNEDD
☐ MAELOR

Confidential Questionnaire

Looking At Your Symptoms Study

This Study has been funded by Cancer Research UK

Cardiff University Prifysgol Caerdydd
Ysgol Glinigol Gogledd Cymru North Wales Clinical School
Cancer Research UK
sewtu South East Wales Trials Unit Uned Ymchwil De-ddwyrain Cymru

Dear Patient

I am very grateful to you for agreeing to help us with this study.

I hope the information you supply will help us give people a diagnosis earlier.

If you feel you would like to talk to someone about this questionnaire at a later stage, please speak to the doctor or nurse who is looking after you, or be in touch with the research team, whose contact details are on your patient information sheet.

I can assure you that anything you tell us will be treated in the utmost confidence.

Yours sincerely

Dr Richard Neal
Senior Clinical Lecturer
Cardiff University
Chief Investigator, Symptoms Study

About your symptoms

These questions are about symptoms you noticed before your diagnosis. We are interested in symptoms which you think are related to your cancer.

Please put a cross in the appropriate box, or write an answer as required.

Looking back...

1. Did you have any symptoms that you feel were due to your cancer before you were diagnosed?

Yes ☐ **Please continue with Question 2**

No ☐ **Please go to Section 2 on Page 5**

2. What was the first symptom that made you think something might be wrong?

☐

Please go to Question 3

The following questions are about when you first noticed a symptom and when you first told your GP or nurse about it.

Please give an exact date if you can. Otherwise give your best estimate. (For example approximately how long ago, the month or season). You wish to refer to your diary or calendar if you have it with you.

Here is an example question:

Decrease in appetite

Did you have this symptom?

Yes ☒ **Please complete A then B or C below**

No ☐ **Please go to Section 2**

A When did you first notice this? d d / m m / y y **OR** Estimate '3 months ago' or 'June'

B When did you first tell your GP or nurse? 2 3 / 1 2 / 0 7 **OR** Estimate

OR

C Put a cross here if you didn't tell your GP or nurse ☐

3. Change in bowel habit

Did you have this symptom?

Yes ☐ **Please complete A then B or C below**

No ☐ **Please go to Question 4**

A When did you first notice this? ☐☐ / ☐☐ / ☐☐ **OR** ☐

B When did you first tell your GP or nurse? ☐☐ / ☐☐ / ☐☐ **OR** ☐

OR

C Put a cross here if you didn't tell your GP or nurse ☐

4. Bleeding from back passage

Did you have this symptom?

Yes ☐ **Please complete A then B or C below**

No ☐ **Please go to Section 2**

A When did you first notice this? ☐☐ / ☐☐ / ☐☐ **OR** ☐

B When did you first tell your GP or nurse? ☐☐ / ☐☐ / ☐☐ **OR** ☐

OR

C Put a cross here if you didn't tell your GP or nurse ☐

Section 2

Please give an exact date if you can. Otherwise give your best estimate (for example approximately how long ago, the month or the season). You may wish to refer to your diary or calendar if you have it with you.

Did you have any of the following?

1. Decrease in appetite

Did you have this?

Yes ☐ **Please complete A then B or C below**

No ☐ **Please go to Question 2**

A When did you first notice this? ☐☐ / ☐☐ / ☐☐ **OR** ☐

B When did you first tell your GP or nurse? ☐☐ / ☐☐ / ☐☐ **OR** ☐

OR

C Put a cross here if you didn't tell your GP or nurse ☐

2. Unexplained weight loss

Did you have this?

Yes ☐ **Please complete A then B or C below**

No ☐ **Please go to Question 3**

A When did you first notice this? ☐☐ / ☐☐ / ☐☐ **OR** ☐

B When did you first tell your GP or nurse? ☐☐ / ☐☐ / ☐☐ **OR** ☐

OR

C Put a cross here if you didn't tell your GP or nurse ☐

3. **Fatigue or tiredness that is unusual for you**

Did you have this?

Yes ☐ **Please complete A then B or C below**

No ☐ **Please go to Question 4**

A When did you <u>first notice</u> this? ☐☐ / ☐☐ / ☐☐ **OR** ☐

B When did you <u>first tell</u> your GP or nurse? ☐☐ / ☐☐ / ☐☐ **OR** ☐

OR

C Put a cross here if you <u>didn't tell</u> your GP or nurse ☐

4. **Feeling different 'in yourself' from usual**

Did you have this?

Yes ☐ **Please complete A then B or C below**

No ☐ **Please go to Question 5**

A When did you <u>first notice</u> this? ☐☐ / ☐☐ / ☐☐ **OR** ☐

B When did you <u>first tell</u> your GP or nurse? ☐☐ / ☐☐ / ☐☐ **OR** ☐

OR

C Put a cross here if you <u>didn't tell</u> your GP or nurse ☐

Please try and describe what the feeling was

If you had any further symptoms which you feel are relevant to your cancer diagnosis, please list them below. If not, please go to Question 7.

Please describe the symptom here and complete A then B or C below

5.

**A**	When did you first notice this?	☐☐ / ☐☐ / ☐☐ **OR** ☐
**B**	When did you first tell your GP or nurse?	☐☐ / ☐☐ / ☐☐ **OR** ☐
	**OR**	
**C**	Put a cross here if you didn't tell your GP or nurse	☐

If you had another symptom you feel is relevant, please write it below, otherwise go to Question 7.

Please describe the symptom here and complete A then B or C below

6.

**A**	When did you first notice this?	☐☐ / ☐☐ / ☐☐ **OR** ☐
**B**	When did you first tell your GP or nurse?	☐☐ / ☐☐ / ☐☐ **OR** ☐
	**OR**	
**C**	Put a cross here if you didn't tell your GP or nurse	☐

If you had further symptoms you would like to tell us about, there is space at the end of the Questionnaire for you to write them.

Did your GP send you for any tests?

Thinking back to the time immediately before you diagnosis, did your GP send you for any of the following tests?

Please cross the appropriate boxes

7. Were you sent for any of these?

	Yes	Not sure	No
Blood test(s)	☐	☐	☐
CT Scan	☐	☐	☐
Ultrasound scan	☐	☐	☐
Barium Enema	☐	☐	☐
Sigmoidoscopy or colonoscopy (Looking at bowel with internal camera)	☐	☐	☐
X-ray	☐	☐	☐

Please go to Question 8

About you

The next questions relate to general information about you. It would help us to know more about the background of the people completing the questionnaire.

8. **Which best describes your employment status?**

Please cross one box only

- Employed full-time ☐
- Employed part-time ☐
- Self employed full-time ☐
- Self employed part-time ☐
- Unemployed (seeking work) ☐
- Unemployed (not seeking work) ☐
- Retired ☐
- Student ☐
- Permanently sick/disabled ☐
- Temporarily sick/disabled ☐
- Looking after family/home ☐
- Other, please describe ____________

9. **What is your highest level of qualification?**

Please cross one box only

- Degree (or equivalent) ☐
- Diploma (or equivalent) ☐
- 'A' level ☐
- GCSE / 'O' level ☐
- None ☐
- Other, please specify ____________

10. **How would you describe your ethnicity**

Please cross one box only

White ☐
or
White British ☐
White Irish ☐
Other White background ☐

Mixed ☐
or
White & Black Carribean ☐
White & Black African ☐
White & Asian ☐
Other mixed background ☐

Black or Black British ☐
or
Carribean ☐
African ☐
Other Black background ☐

Asian or Asian British ☐
or
Indian ☐
Pakistani ☐
Bangladeshi ☐
Other Asian background ☐

Chinese or other ethnic group ☐
or
Chinese ☐
Other ethnic group ☐

11.

Do you live alone?

Yes ☐ **Please go to Question 12**

No ☐ **Please say who you live with below**

Who do you live with?
(e.g. wife, husband, partner, family member)

☐

12. Are you suffering from, or have you suffered from, any of the following in the past 2 years?

Please cross any that are applicable to you

- Asthma ☐
- Chronic Obstructive Pulmonary Disease (COPD) ☐
- Other lung disease (e.g. fibrosis, bronchiectatis etc) ☐
- Heart disease ☐
- Anxiety or depression ☐
- Inflammatory bowel disease ☐
- Irritable bowel syndrome ☐
- Peptic ulcer ☐
- Previous cancer ☐
- Diabetes ☐
- Arthritis ☐

13. About smoking

Please cross the appropriate statement

- Are you a current smoker? ☐
- Are you an ex-smoker? ☐
- Are you a non-smoker (never smoked)? ☐

14. Do you think you were more at risk of getting cancer because of your family history?

Yes ☐

No ☐

15. The following questions are about how completing the questionnaire made you feel.

A number of statements which people have used to describe themselves are given below. Read each statement and then cross the most appropriate box to indicate how you feel right now, at the moment. There are no right or wrong answers. Do not spend too much time on any one statement but give the answer which seems to describe your present feeling best.

		Not at all	Somewhat	Moderately	Very much
1.	I feel calm	☐	☐	☐	☐
2.	I am tense	☐	☐	☐	☐
3.	I feel upset	☐	☐	☐	☐
4.	I am relaxed	☐	☐	☐	☐
5.	I feel content	☐	☐	☐	☐
6.	I am worried	☐	☐	☐	☐

Please make sure you have answered all the questions.

If you have any other comments or there is anything else you would like to tell us about when you first noticed your symptoms, please do so using the space below.

If you feel you would like to talk to someone about this questionnaire at a later stage, please speak to the doctor or nurse who is looking after you, or be in touch with the research team, whose contact details are on your patient information sheet

Thank you for completing the questionnaire

Please give it to the research nurse

The next page is for the Researcher to complete

Researcher - Post-completion Sheet

Researcher initials: ☐☐☐

Date questionnaire completed: Date ☐☐ / ☐☐ / ☐☐ Time ☐☐:☐☐

If not completed, give reason ☐

How long is it since the patient was told of his/her diagnosis? ☐

How long did it take the patient to complete the questionnaire?

- Less than 5 minutes ☐
- Between 5 and 10 minutes ☐
- Between 11 and 15 minutes ☐
- Longer than 15 minutes ☐

For the patient selected to complete the questionnaire on their own

Did the patient ask for any help? ☐ Yes ☐ No

If yes,

please specify what help was requested

____

____

please specify what help was given

____

____

For the patient selected for the researcher-administered questionnaire

Did the patient find any of the questions difficult? ☐ Yes ☐ No

If yes,

please specify which questions and what the difficulty was

____

____

____

____

Was the patient made anxious? ☐ Yes ☐ No

If yes,

please give details

____

____

____

____

Where did the patient complete the questionnaire?

(Please specify whether this was in a separate room alone, in a corner of the clinic, the ward or other

____

Thank you. Please now send the questionnaire and patient information sheet and consent form back to Cardiff University in the freepost envelope provided.

57727

PID - | | | 7 |

Ymddiriedolaeth GIG Gogledd Orllewin Cymru
North West Wales NHS Trust

NHS WALES GIG CYMRU
Ymddiriedolaeth GIG Gogledd Cymru
North Wales NHS Trust

☐ GLAN CLWYD

CID - ☐ GWYNEDD

☐ MAELOR

Confidential Questionnaire

Looking At Your Symptoms Study

This Study has been funded by Cancer Research UK

CARDIFF UNIVERSITY PRIFYSGOL CAERDYDD
Ysgol Glinigol Gogledd Cymru North Wales Clinical School
CANCER RESEARCH UK
sewtu South East Wales Trials Unit Uned Ymchwil De-ddwyrain Cymru

Dear Patient

I am very grateful to you for agreeing to help us with this study.

I hope the information you supply will help us give people a diagnosis earlier.

If you feel you would like to talk to someone about this questionnaire at a later stage, please speak to the doctor or nurse who is looking after you, or be in touch with the research team, whose contact details are on your patient information sheet.

I can assure you that anything you tell us will be treated in the utmost confidence.

Yours sincerely

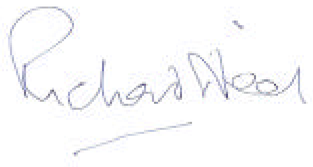

Dr Richard Neal
Senior Clinical Lecturer
Cardiff University
Chief Investigator, Symptoms Study

About your symptoms

These questions are about symptoms you noticed before your diagnosis. We are interested in symptoms which you think are related to your cancer.

Please put a cross in the appropriate box, or write an answer as required.

Looking back...

1. Did you have any symptoms that you feel were due to your cancer before you were diagnosed?

Yes ☐ **Please continue with Question 2**

No ☐ **Please go to Section 2 on Page 5**

2. What was the first symptom that made you think something might be wrong?

☐

Please go to Question 3

The following questions are about when you first noticed a symptom and when you first told your GP or nurse about it.

Please give an exact date if you can. Otherwise give your best estimate. (For example approximately how long ago, the month or season). You wish to refer to your diary or calendar if you have it with you.

Here is an example question:

Decrease in appetite

Did you have this symptom?

Yes ☒ **Please complete A then B or C below**

No ☐ **Please go to Section 2**

A When did you first notice this? d d / m m / y y **OR** Estimate '3 months ago' or 'June'

B When did you first tell your GP or nurse? 2 3 / 1 2 / 0 7 **OR** Estimate

OR

C Put a cross here if you didn't tell your GP or nurse ☐

3. Change in bowel habit

Did you have this symptom?

Yes ☐ **Please complete A then B or C below**

No ☐ **Please go to Question 4**

A When did you first notice this? ☐☐ / ☐☐ / ☐☐ **OR** ☐

B When did you first tell your GP or nurse? ☐☐ / ☐☐ / ☐☐ **OR** ☐

OR

C Put a cross here if you didn't tell your GP or nurse ☐

4. Bleeding from back passage

Did you have this symptom?

Yes ☐ **Please complete A then B or C below**

No ☐ **Please go to Section 2**

A When did you first notice this? ☐☐ / ☐☐ / ☐☐ **OR** ☐

B When did you first tell your GP or nurse? ☐☐ / ☐☐ / ☐☐ **OR** ☐

OR

C Put a cross here if you didn't tell your GP or nurse ☐

Please give an exact date if you can. Otherwise give your best estimate (for example approximately how long ago, the month or the season). You may wish to refer to your diary or calendar if you have it with you.

Did you have any of the following?

1. Decrease in appetite

Did you have this?

Yes ☐ **Please complete A then B or C below**

No ☐ **Please go to Question 2**

A When did you first notice this? ☐☐ / ☐☐ / ☐☐ **OR** ☐

B When did you first tell your GP or nurse? ☐☐ / ☐☐ / ☐☐ **OR** ☐

OR

C Put a cross here if you didn't tell your GP or nurse ☐

2. Unexplained weight loss

Did you have this?

Yes ☐ **Please complete A then B or C below**

No ☐ **Please go to Question 3**

A When did you first notice this? ☐☐ / ☐☐ / ☐☐ **OR** ☐

B When did you first tell your GP or nurse? ☐☐ / ☐☐ / ☐☐ **OR** ☐

OR

C Put a cross here if you didn't tell your GP or nurse ☐

3. Fatigue or tiredness that is unusual for you

Did you have this?

Yes ☐ **Please complete A then B or C below**

No ☐ **Please go to Question 4**

A When did you first notice this? ☐☐ / ☐☐ / ☐☐ **OR** ☐

B When did you first tell your GP or nurse? ☐☐ / ☐☐ / ☐☐ **OR** ☐

OR

C Put a cross here if you didn't tell your GP or nurse ☐

4. Feeling different 'in yourself' from usual

Did you have this?

Yes ☐ **Please complete A then B or C below**

No ☐ **Please go to Question 5**

A When did you first notice this? ☐☐ / ☐☐ / ☐☐ **OR** ☐

B When did you first tell your GP or nurse? ☐☐ / ☐☐ / ☐☐ **OR** ☐

OR

C Put a cross here if you didn't tell your GP or nurse ☐

Please try and describe what the feeling was

If you had any further symptoms which you feel are relevant to your cancer diagnosis, please list them below. If not, please go to Question 7.

Please describe the symptom here and complete A then B or C below

5.

**A** When did you first notice this?	□□ / □□ / □□ **OR**
**B** When did you first tell your GP or nurse?	□□ / □□ / □□ **OR**
**OR**	
**C** Put a cross here if you didn't tell your GP or nurse	□

If you had another symptom you feel is relevant, please write it below, otherwise go to Question 7.

Please describe the symptom here and complete A then B or C below

6.

**A** When did you first notice this?	□□ / □□ / □□ **OR**
**B** When did you first tell your GP or nurse?	□□ / □□ / □□ **OR**
**OR**	
**C** Put a cross here if you didn't tell your GP or nurse	□

If you had further symptoms you would like to tell us about, there is space at the end of the Questionnaire for you to write them.

Did your GP send you for any tests?

Thinking back to the time immediately before you diagnosis, did your GP send you for any of the following tests?

Please cross the appropriate boxes

7. Were you sent for any of these?

	Yes	Not sure	No
Blood test(s)	☐	☐	☐
CT Scan	☐	☐	☐
Ultrasound scan	☐	☐	☐
Barium Enema	☐	☐	☐
Sigmoidoscopy or colonoscopy (Looking at bowel with internal camera)	☐	☐	☐
X-ray	☐	☐	☐

Please go to Question 8

About you

The next questions relate to general information about you. It would help us to know more about the background of the people completing the questionnaire.

8. Which best describes your employment status?

Please cross one box only

- Employed full-time ☐
- Employed part-time ☐
- Self employed full-time ☐
- Self employed part-time ☐
- Unemployed (seeking work) ☐
- Unemployed (not seeking work) ☐
- Retired ☐
- Student ☐
- Permanently sick/disabled ☐
- Temporarily sick/disabled ☐
- Looking after family/home ☐
- Other, please describe ____

9. What is your highest level of qualification?

Please cross one box only

- Degree (or equivalent) ☐
- Diploma (or equivalent) ☐
- 'A' level ☐
- GCSE / 'O' level ☐
- None ☐
- Other, please specify ____

10. **How would you describe your ethnicity**

Please cross one box only

White ☐
or
- White British ☐
- White Irish ☐
- Other White background ☐

Mixed ☐
or
- White & Black Carribean ☐
- White & Black African ☐
- White & Asian ☐
- Other mixed background ☐

Black or Black British ☐
or
- Carribean ☐
- African ☐
- Other Black background ☐

Asian or Asian British ☐
or
- Indian ☐
- Pakistani ☐
- Bangladeshi ☐
- Other Asian background ☐

Chinese or other ethnic group ☐
or
- Chinese ☐
- Other ethnic group ☐

11. **Do you live alone?**

Yes ☐ **Please go to Question 12**

No ☐ **Please say who you live with below**

Who do you live with?
(e.g. wife, husband, partner, family member)

12. **Are you suffering from, or have you suffered from, any of the following in the past 2 years?**

Please cross any that are applicable to you

- Asthma ☐
- Chronic Obstructive Pulmonary Disease (COPD) ☐
- Other lung disease (e.g. fibrosis, bronchiectatis etc) ☐
- Heart disease ☐
- Anxiety or depression ☐
- Inflammatory bowel disease ☐
- Irritable bowel syndrome ☐
- Peptic ulcer ☐
- Previous cancer ☐
- Diabetes ☐
- Arthritis ☐

13. **About smoking**

Please cross the appropriate statement

- Are you a current smoker? ☐
- Are you an ex-smoker? ☐
- Are you a non-smoker (never smoked)? ☐

14. **Do you think you were more at risk of getting cancer because of your family history?**

Yes ☐
No ☐

15. **The following questions are about how completing the questionnaire made you feel.**

A number of statements which people have used to describe themselves are given below. Read each statement and then cross the most appropriate box to indicate how you feel right now, at the moment. There are no right or wrong answers. Do not spend too much time on any one statement but give the answer which seems to describe your present feeling best.

		Not at all	Somewhat	Moderately	Very much
1.	I feel calm	☐	☐	☐	☐
2.	I am tense	☐	☐	☐	☐
3.	I feel upset	☐	☐	☐	☐
4.	I am relaxed	☐	☐	☐	☐
5.	I feel content	☐	☐	☐	☐
6.	I am worried	☐	☐	☐	☐

Please make sure you have answered all the questions.

If you have any other comments or there is anything else you would like to tell us about when you first noticed your symptoms, please do so using the space below.

If you feel you would like to talk to someone about this questionnaire at a later stage, please speak to the doctor or nurse who is looking after you, or be in touch with the research team, whose contact details are on your patient information sheet

Thank you for completing the questionnaire

Please give it to the research nurse

The next page is for the Researcher to complete

57727

7

Researcher - Post-completion Sheet

Researcher initials: ☐☐☐

Date questionnaire completed: Date ☐☐ / ☐☐ / ☐☐ Time ☐☐:☐☐

If not completed, give reason ☐

How long is it since the patient was told of his/her diagnosis? ☐

How long did it take the patient to complete the questionnaire?

- Less than 5 minutes ☐
- Between 5 and 10 minutes ☐
- Between 11 and 15 minutes ☐
- Longer than 15 minutes ☐

For the patient selected to <u>complete the questionnaire on their own</u>

Did the patient ask for any help? ☐ Yes ☐ No

If yes,

please specify what help was requested

____

____

please specify what help was given

____

____

For the patient selected for <u>the researcher-administered questionnaire</u>

Did the patient find any of the questions difficult? ☐ Yes ☐ No

If yes,

please specify which questions and what the difficulty was

____

____

____

____

Was the patient made anxious? ☐ Yes ☐ No

If yes,

please give details

____

____

____

____

Where did the patient complete the questionnaire?

(Please specify whether this was in a separate room alone, in a corner of the clinic, the ward or other

____

Thank you. Please now send the questionnaire and patient information sheet and consent form back to Cardiff University in the freepost envelope provided.

57727

PID - | | | 8 |

Ymddiriedolaeth GIG Gogledd Orllewin Cymru
North West Wales NHS Trust

NHS WALES GIG CYMRU
Ymddiriedolaeth GIG Gogledd Cymru
North Wales NHS Trust

☐ GLAN CLWYD

CID - ☐ GWYNEDD

☐ MAELOR

Confidential Questionnaire

Looking At Your Symptoms Study

This Study has been funded by Cancer Research UK

Cardiff University / Prifysgol Caerdydd

Ysgol Glinigol Gogledd Cymru / North Wales Clinical School

Cancer Research UK

sewtu — South East Wales Trials Unit / Uned Ymchwil De-ddwyrain Cymru

Dear Patient

I am very grateful to you for agreeing to help us with this study.

I hope the information you supply will help us give people a diagnosis earlier.

If you feel you would like to talk to someone about this questionnaire at a later stage, please speak to the doctor or nurse who is looking after you, or be in touch with the research team, whose contact details are on your patient information sheet.

I can assure you that anything you tell us will be treated in the utmost confidence.

Yours sincerely

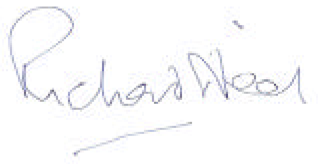

Dr Richard Neal
Senior Clinical Lecturer
Cardiff University
Chief Investigator, Symptoms Study

About your symptoms

These questions are about symptoms you noticed before your diagnosis. We are interested in symptoms which you think are related to your cancer.

Please put a cross in the appropriate box, or write an answer as required.

Looking back...

1. Did you have any symptoms that you feel were due to your cancer before you were diagnosed?

Yes ☐ **Please continue with Question 2**

No ☐ **Please go to Section 2 on Page 5**

2. What was the first symptom that made you think something might be wrong?

☐

Please go to Question 3

The following questions are about when you first noticed a symptom and when you first told your GP or nurse about it.

Please give an exact date if you can. Otherwise give your best estimate. (For example approximately how long ago, the month or season). You wish to refer to your diary or calendar if you have it with you.

Here is an example question:

Decrease in appetite

Did you have this symptom?

Yes ☒ **Please complete A then B or C below**

No ☐ **Please go to Section 2**

A When did you first notice this? d d / m m / y y **OR** Estimate '3 months ago' or 'June'

B When did you first tell your GP or nurse? 2 3 / 1 2 / 0 7 **OR** Estimate

OR

C Put a cross here if you didn't tell your GP or nurse ☐

3. Change in bowel habit

Did you have this symptom?

Yes ☐ **Please complete A then B or C below**

No ☐ **Please go to Question 4**

A When did you first notice this? ☐☐ / ☐☐ / ☐☐ **OR** ☐

B When did you first tell your GP or nurse? ☐☐ / ☐☐ / ☐☐ **OR** ☐

OR

C Put a cross here if you didn't tell your GP or nurse ☐

4. Bleeding from back passage

Did you have this symptom?

Yes ☐ **Please complete A then B or C below**

No ☐ **Please go to Section 2**

A When did you first notice this? ☐☐ / ☐☐ / ☐☐ **OR** ☐

B When did you first tell your GP or nurse? ☐☐ / ☐☐ / ☐☐ **OR** ☐

OR

C Put a cross here if you didn't tell your GP or nurse ☐

Section 2

Please give an exact date if you can. Otherwise give your best estimate (for example approximately how long ago, the month or the season). You may wish to refer to your diary or calendar if you have it with you.

Did you have any of the following?

1. Decrease in appetite

Did you have this?

Yes ☐ **Please complete A then B or C below**

No ☐ **Please go to Question 2**

A When did you first notice this? ☐☐ / ☐☐ / ☐☐ **OR** ☐

B When did you first tell your GP or nurse? ☐☐ / ☐☐ / ☐☐ **OR** ☐

OR

C Put a cross here if you didn't tell your GP or nurse ☐

2. Unexplained weight loss

Did you have this?

Yes ☐ **Please complete A then B or C below**

No ☐ **Please go to Question 3**

A When did you first notice this? ☐☐ / ☐☐ / ☐☐ **OR** ☐

B When did you first tell your GP or nurse? ☐☐ / ☐☐ / ☐☐ **OR** ☐

OR

C Put a cross here if you didn't tell your GP or nurse ☐

3. **Fatigue or tiredness that is unusual for you**

Did you have this?

Yes ☐ **Please complete A then B or C below**

No ☐ **Please go to Question 4**

A When did you first notice this? ☐☐ / ☐☐ / ☐☐ **OR** ☐

B When did you first tell your GP or nurse? ☐☐ / ☐☐ / ☐☐ **OR** ☐

OR

C Put a cross here if you didn't tell your GP or nurse ☐

4. **Feeling different 'in yourself' from usual**

Did you have this?

Yes ☐ **Please complete A then B or C below**

No ☐ **Please go to Question 5**

A When did you first notice this? ☐☐ / ☐☐ / ☐☐ **OR** ☐

B When did you first tell your GP or nurse? ☐☐ / ☐☐ / ☐☐ **OR** ☐

OR

C Put a cross here if you didn't tell your GP or nurse ☐

Please try and describe what the feeling was

If you had any further symptoms which you feel are relevant to your cancer diagnosis, please list them below. If not, please go to Question 7.

Please describe the symptom here and complete A then B or C below

5.

**A**	When did you first notice this?	☐☐ / ☐☐ / ☐☐ **OR** ☐
**B**	When did you first tell your GP or nurse?	☐☐ / ☐☐ / ☐☐ **OR** ☐
	**OR**	
**C**	Put a cross here if you didn't tell your GP or nurse	☐

If you had another symptom you feel is relevant, please write it below, otherwise go to Question 7.

Please describe the symptom here and complete A then B or C below

6.

**A**	When did you first notice this?	☐☐ / ☐☐ / ☐☐ **OR** ☐
**B**	When did you first tell your GP or nurse?	☐☐ / ☐☐ / ☐☐ **OR** ☐
	**OR**	
**C**	Put a cross here if you didn't tell your GP or nurse	☐

If you had further symptoms you would like to tell us about, there is space at the end of the Questionnaire for you to write them.

Did your GP send you for any tests?

Thinking back to the time immediately before you diagnosis, did your GP send you for any of the following tests?

Please cross the appropriate boxes

7. Were you sent for any of these?

	Yes	Not sure	No
Blood test(s)	☐	☐	☐
CT Scan	☐	☐	☐
Ultrasound scan	☐	☐	☐
Barium Enema	☐	☐	☐
Sigmoidoscopy or colonoscopy (Looking at bowel with internal camera)	☐	☐	☐
X-ray	☐	☐	☐

Please go to Question 8

About you

The next questions relate to general information about you. It would help us to know more about the background of the people completing the questionnaire.

8. Which best describes your employment status?

Please cross one box only

- Employed full-time ☐
- Employed part-time ☐
- Self employed full-time ☐
- Self employed part-time ☐
- Unemployed (seeking work) ☐
- Unemployed (not seeking work) ☐
- Retired ☐
- Student ☐
- Permanently sick/disabled ☐
- Temporarily sick/disabled ☐
- Looking after family/home ☐
- Other, please describe ______

9. What is your highest level of qualification?

Please cross one box only

- Degree (or equivalent) ☐
- Diploma (or equivalent) ☐
- 'A' level ☐
- GCSE / 'O' level ☐
- None ☐
- Other, please specify ______

10. **How would you describe your ethnicity**

Please cross one box only

White ☐
or
White British ☐
White Irish ☐
Other White background ☐

Mixed ☐
or
White & Black Carribean ☐
White & Black African ☐
White & Asian ☐
Other mixed background ☐

Black or Black British ☐
or
Carribean ☐
African ☐
Other Black background ☐

Asian or Asian British ☐
or
Indian ☐
Pakistani ☐
Bangladeshi ☐
Other Asian background ☐

Chinese or other ethnic group ☐
or
Chinese ☐
Other ethnic group ☐

11.

Do you live alone?

Yes ☐ **Please go to Question 12**

No ☐ **Please say who you live with below**

Who do you live with?
(e.g. wife, husband, partner, family member)

12. **Are you suffering from, or have you suffered from, any of the following in the past 2 years?**

Please cross any that are applicable to you

- Asthma ☐
- Chronic Obstructive Pulmonary Disease (COPD) ☐
- Other lung disease (e.g. fibrosis, bronchiectatis etc) ☐
- Heart disease ☐
- Anxiety or depression ☐
- Inflammatory bowel disease ☐
- Irritable bowel syndrome ☐
- Peptic ulcer ☐
- Previous cancer ☐
- Diabetes ☐
- Arthritis ☐

13. **About smoking**

Please cross the appropriate statement

- Are you a current smoker? ☐
- Are you an ex-smoker? ☐
- Are you a non-smoker (never smoked)? ☐

14. **Do you think you were more at risk of getting cancer because of your family history?**

Yes ☐
No ☐

15. **The following questions are about how completing the questionnaire made you feel.**

A number of statements which people have used to describe themselves are given below. Read each statement and then cross the most appropriate box to indicate how you feel right now, at the moment. There are no right or wrong answers. Do not spend too much time on any one statement but give the answer which seems to describe your present feeling best.

		Not at all	Somewhat	Moderately	Very much
1.	I feel calm	☐	☐	☐	☐
2.	I am tense	☐	☐	☐	☐
3.	I feel upset	☐	☐	☐	☐
4.	I am relaxed	☐	☐	☐	☐
5.	I feel content	☐	☐	☐	☐
6.	I am worried	☐	☐	☐	☐

Please make sure you have answered all the questions.

57727

8

If you have any other comments or there is anything else you would like to tell us about when you first noticed your symptoms, please do so using the space below.

If you feel you would like to talk to someone about this questionnaire at a later stage, please speak to the doctor or nurse who is looking after you, or be in touch with the research team, whose contact details are on your patient information sheet

Thank you for completing the questionnaire

Please give it to the research nurse

The next page is for the Researcher to complete

Researcher - Post-completion Sheet **Researcher initials:** ☐☐☐

Date questionnaire completed: Date ☐☐ / ☐☐ / ☐☐ Time ☐☐:☐☐

If not completed, give reason ☐

How long is it since the patient was told of his/her diagnosis? ☐

How long did it take the patient to complete the questionnaire?

- Less than 5 minutes ☐
- Between 5 and 10 minutes ☐
- Between 11 and 15 minutes ☐
- Longer than 15 minutes ☐

For the patient selected to complete the questionnaire on their own

Did the patient ask for any help? ☐ Yes ☐ No

If yes,

please specify what help was requested

____

____

please specify what help was given

____

____

For the patient selected for the researcher-administered questionnaire

Did the patient find any of the questions difficult? ☐ Yes ☐ No

If yes,

please specify which questions and what the difficulty was

____

____

____

____

Was the patient made anxious? ☐ Yes ☐ No

If yes,

please give details

____

____

____

____

Where did the patient complete the questionnaire?

(Please specify whether this was in a separate room alone, in a corner of the clinic, the ward or other

____

Thank you. Please now send the questionnaire and patient information sheet and consent form back to Cardiff University in the freepost envelope provided.

57727

PID - | | | 9 |

Ymddiriedolaeth GIG Gogledd Orllewin Cymru
North West Wales NHS Trust

NHS WALES GIG CYMRU
Ymddiriedolaeth GIG Gogledd Cymru
North Wales NHS Trust

☐ GLAN CLWYD

CID - ☐ GWYNEDD

☐ MAELOR

Confidential Questionnaire

Looking At Your Symptoms Study

This Study has been funded by Cancer Research UK

Cardiff University / Prifysgol Caerdydd — Ysgol Glinigol Gogledd Cymru / North Wales Clinical School — Cancer Research UK — sewtu South East Wales Trials Unit / Uned Ymchwil De-ddwyrain Cymru

Dear Patient

I am very grateful to you for agreeing to help us with this study.

I hope the information you supply will help us give people a diagnosis earlier.

If you feel you would like to talk to someone about this questionnaire at a later stage, please speak to the doctor or nurse who is looking after you, or be in touch with the research team, whose contact details are on your patient information sheet.

I can assure you that anything you tell us will be treated in the utmost confidence.

Yours sincerely

Richard Neal

Dr Richard Neal
Senior Clinical Lecturer
Cardiff University
Chief Investigator, Symptoms Study

About your symptoms

These questions are about symptoms you noticed before your diagnosis. We are interested in symptoms which you think are related to your cancer.

Please put a cross in the appropriate box, or write an answer as required.

Looking back...

1. Did you have any symptoms that you feel were due to your cancer before you were diagnosed?

- Yes ☐ **Please continue with Question 2**
- No ☐ **Please go to Section 2 on Page 5**

2. What was the first symptom that made you think something might be wrong?

[ ]

Please go to Question 3

The following questions are about when you first noticed a symptom and when you first told your GP or nurse about it.

Please give an exact date if you can. Otherwise give your best estimate. (For example approximately how long ago, the month or season). You wish to refer to your diary or calendar if you have it with you.

Here is an example question:

Decrease in appetite

Did you have this symptom?

- Yes ☒ **Please complete A then B or C below**
- No ☐ **Please go to Section 2**

A When did you first notice this? d d / m m / y y **OR** Estimate '3 months ago' or 'June'

B When did you first tell your GP or nurse? 2 3 / 1 2 / 0 7 **OR** Estimate

OR

C Put a cross here if you didn't tell your GP or nurse ☐

3. Change in bowel habit

Did you have this symptom?

Yes ☐ **Please complete A then B or C below**

No ☐ **Please go to Question 4**

A When did you first notice this? ☐☐ / ☐☐ / ☐☐ **OR** ☐

B When did you first tell your GP or nurse? ☐☐ / ☐☐ / ☐☐ **OR** ☐

OR

C Put a cross here if you didn't tell your GP or nurse ☐

4. Bleeding from back passage

Did you have this symptom?

Yes ☐ **Please complete A then B or C below**

No ☐ **Please go to Section 2**

A When did you first notice this? ☐☐ / ☐☐ / ☐☐ **OR** ☐

B When did you first tell your GP or nurse? ☐☐ / ☐☐ / ☐☐ **OR** ☐

OR

C Put a cross here if you didn't tell your GP or nurse ☐

Section 2

Please give an exact date if you can. Otherwise give your best estimate (for example approximately how long ago, the month or the season). You may wish to refer to your diary or calendar if you have it with you.

Did you have any of the following?

1. Decrease in appetite

Did you have this?

Yes ☐ **Please complete A then B or C below**

No ☐ **Please go to Question 2**

A When did you first notice this? ☐☐ / ☐☐ / ☐☐ **OR** ☐

B When did you first tell your GP or nurse? ☐☐ / ☐☐ / ☐☐ **OR** ☐

OR

C Put a cross here if you didn't tell your GP or nurse ☐

2. Unexplained weight loss

Did you have this?

Yes ☐ **Please complete A then B or C below**

No ☐ **Please go to Question 3**

A When did you first notice this? ☐☐ / ☐☐ / ☐☐ **OR** ☐

B When did you first tell your GP or nurse? ☐☐ / ☐☐ / ☐☐ **OR** ☐

OR

C Put a cross here if you didn't tell your GP or nurse ☐

3. Fatigue or tiredness that is unusual for you

Did you have this?

Yes ☐ **Please complete A then B or C below**

No ☐ **Please go to Question 4**

A When did you first notice this? ☐☐ / ☐☐ / ☐☐ **OR** ☐

B When did you first tell your GP or nurse? ☐☐ / ☐☐ / ☐☐ **OR** ☐

OR

C Put a cross here if you didn't tell your GP or nurse ☐

4. Feeling different 'in yourself' from usual

Did you have this?

Yes ☐ **Please complete A then B or C below**

No ☐ **Please go to Question 5**

A When did you first notice this? ☐☐ / ☐☐ / ☐☐ **OR** ☐

B When did you first tell your GP or nurse? ☐☐ / ☐☐ / ☐☐ **OR** ☐

OR

C Put a cross here if you didn't tell your GP or nurse ☐

Please try and describe what the feeling was

If you had any further symptoms which you feel are relevant to your cancer diagnosis, please list them below. If not, please go to Question 7.

Please describe the symptom here and complete A then B or C below

5.

**A** When did you <u>first notice</u> this?	_ _ / _ _ / _ _ **OR**
**B** When did you <u>first tell</u> your GP or nurse?	_ _ / _ _ / _ _ **OR**
**OR**	
**C** Put a cross here if you <u>didn't tell</u> your GP or nurse	☐

If you had another symptom you feel is relevant, please write it below, otherwise go to Question 7.

Please describe the symptom here and complete A then B or C below

6.

**A** When did you <u>first notice</u> this?	_ _ / _ _ / _ _ **OR**
**B** When did you <u>first tell</u> your GP or nurse?	_ _ / _ _ / _ _ **OR**
**OR**	
**C** Put a cross here if you <u>didn't tell</u> your GP or nurse	☐

If you had further symptoms you would like to tell us about, there is space at the end of the Questionnaire for you to write them.

Did your GP send you for any tests?

Thinking back to the time immediately before you diagnosis, did your GP send you for any of the following tests?

Please cross the appropriate boxes

7. Were you sent for any of these?

	Yes	Not sure	No
Blood test(s)	☐	☐	☐
CT Scan	☐	☐	☐
Ultrasound scan	☐	☐	☐
Barium Enema	☐	☐	☐
Sigmoidoscopy or colonoscopy (Looking at bowel with internal camera)	☐	☐	☐
X-ray	☐	☐	☐

Please go to Question 8

About you

The next questions relate to general information about you. It would help us to know more about the background of the people completing the questionnaire.

8. Which best describes your employment status?

Please cross one box only

- Employed full-time ☐
- Employed part-time ☐
- Self employed full-time ☐
- Self employed part-time ☐
- Unemployed (seeking work) ☐
- Unemployed (not seeking work) ☐
- Retired ☐
- Student ☐
- Permanently sick/disabled ☐
- Temporarily sick/disabled ☐
- Looking after family/home ☐

Other, please describe ______

9. What is your highest level of qualification?

Please cross one box only

- Degree (or equivalent) ☐
- Diploma (or equivalent) ☐
- 'A' level ☐
- GCSE / 'O' level ☐
- None ☐

Other, please specify ______

10. **How would you describe your ethnicity**

Please cross one box only

White ☐
or
- White British ☐
- White Irish ☐
- Other White background ☐

Mixed ☐
or
- White & Black Carribean ☐
- White & Black African ☐
- White & Asian ☐
- Other mixed background ☐

Black or Black British ☐
or
- Carribean ☐
- African ☐
- Other Black background ☐

Asian or Asian British ☐
or
- Indian ☐
- Pakistani ☐
- Bangladeshi ☐
- Other Asian background ☐

Chinese or other ethnic group ☐
or
- Chinese ☐
- Other ethnic group ☐

11.

Do you live alone?

Yes ☐ **Please go to Question 12**

No ☐ **Please say who you live with below**

Who do you live with?
(e.g. wife, husband, partner, family member)

12. Are you suffering from, or have you suffered from, any of the following in the past 2 years?

Please cross any that are applicable to you

- Asthma ☐
- Chronic Obstructive Pulmonary Disease (COPD) ☐
- Other lung disease (e.g. fibrosis, bronchiectatis etc) ☐
- Heart disease ☐
- Anxiety or depression ☐
- Inflammatory bowel disease ☐
- Irritable bowel syndrome ☐
- Peptic ulcer ☐
- Previous cancer ☐
- Diabetes ☐
- Arthritis ☐

13. About smoking

Please cross the appropriate statement

- Are you a current smoker? ☐
- Are you an ex-smoker? ☐
- Are you a non-smoker (never smoked)? ☐

14. Do you think you were more at risk of getting cancer because of your family history?

Yes ☐
No ☐

15. The following questions are about how completing the questionnaire made you feel.

A number of statements which people have used to describe themselves are given below. Read each statement and then cross the most appropriate box to indicate how you feel right now, at the moment. There are no right or wrong answers. Do not spend too much time on any one statement but give the answer which seems to describe your present feeling best.

		Not at all	Somewhat	Moderately	Very much
1.	I feel calm	☐	☐	☐	☐
2.	I am tense	☐	☐	☐	☐
3.	I feel upset	☐	☐	☐	☐
4.	I am relaxed	☐	☐	☐	☐
5.	I feel content	☐	☐	☐	☐
6.	I am worried	☐	☐	☐	☐

Please make sure you have answered all the questions.

If you have any other comments or there is anything else you would like to tell us about when you first noticed your symptoms, please do so using the space below.

If you feel you would like to talk to someone about this questionnaire at a later stage, please speak to the doctor or nurse who is looking after you, or be in touch with the research team, whose contact details are on your patient information sheet

Thank you for completing the questionnaire

Please give it to the research nurse

The next page is for the Researcher to complete

Researcher - Post-completion Sheet

Researcher initials: ☐☐☐

Date questionnaire completed: Date ☐☐ / ☐☐ / ☐☐ Time ☐☐:☐☐

If not completed, give reason ☐

How long is it since the patient was told of his/her diagnosis? ☐

How long did it take the patient to complete the questionnaire?

- Less than 5 minutes ☐
- Between 5 and 10 minutes ☐
- Between 11 and 15 minutes ☐
- Longer than 15 minutes ☐

For the patient selected to complete the questionnaire on their own

Did the patient ask for any help? ☐ Yes ☐ No

If yes,

please specify what help was requested

please specify what help was given

For the patient selected for the researcher-administered questionnaire

Did the patient find any of the questions difficult? ☐ Yes ☐ No

If yes,

please specify which questions and what the difficulty was

Was the patient made anxious? ☐ Yes ☐ No

If yes,

please give details

Where did the patient complete the questionnaire?

(Please specify whether this was in a separate room alone, in a corner of the clinic, the ward or other

Thank you. Please now send the questionnaire and patient information sheet and consent form back to Cardiff University in the freepost envelope provided.

57727

PID - | | 1 | 0 |

Ymddiriedolaeth GIG Gogledd Orllewin Cymru
North West Wales NHS Trust

NHS WALES GIG CYMRU
Ymddiriedolaeth GIG Gogledd Cymru
North Wales NHS Trust

☐ GLAN CLWYD

CID - ☐ GWYNEDD

☐ MAELOR

Confidential Questionnaire

Looking At Your Symptoms Study

This Study has been funded by Cancer Research UK

Cardiff University / Prifysgol Caerdydd
Ysgol Glinigol Gogledd Cymru / North Wales Clinical School
Cancer Research UK
sewtu South East Wales Trials Unit / Uned Ymchwil De-ddwyrain Cymru

Dear Patient

I am very grateful to you for agreeing to help us with this study.

I hope the information you supply will help us give people a diagnosis earlier.

If you feel you would like to talk to someone about this questionnaire at a later stage, please speak to the doctor or nurse who is looking after you, or be in touch with the research team, whose contact details are on your patient information sheet.

I can assure you that anything you tell us will be treated in the utmost confidence.

Yours sincerely

Dr Richard Neal
Senior Clinical Lecturer
Cardiff University
Chief Investigator, Symptoms Study

About your symptoms

These questions are about symptoms you noticed before your diagnosis. We are interested in symptoms which you think are related to your cancer.

Please put a cross in the appropriate box, or write an answer as required.

Looking back...

1. Did you have any symptoms that you feel were due to your cancer before you were diagnosed?

Yes ☐ **Please continue with Question 2**

No ☐ **Please go to Section 2 on Page 5**

2. What was the first symptom that made you think something might be wrong?

☐

Please go to Question 3

The following questions are about when you first noticed a symptom and when you first told your GP or nurse about it.

Please give an exact date if you can. Otherwise give your best estimate. (For example approximately how long ago, the month or season). You wish to refer to your diary or calendar if you have it with you.

Here is an example question:

Decrease in appetite

Did you have this symptom?

Yes ☒ **Please complete A then B or C below**

No ☐ **Please go to Section 2**

A When did you first notice this? d d / m m / y y **OR** Estimate '3 months ago' or 'June'

B When did you first tell your GP or nurse? 2 3 / 1 2 / 0 7 **OR** Estimate

OR

C Put a cross here if you didn't tell your GP or nurse ☐

3. Change in bowel habit

Did you have this symptom?

Yes ☐ **Please complete A then B or C below**

No ☐ **Please go to Question 4**

A When did you first notice this? ☐☐ / ☐☐ / ☐☐ **OR** ☐

B When did you first tell your GP or nurse? ☐☐ / ☐☐ / ☐☐ **OR** ☐

OR

C Put a cross here if you didn't tell your GP or nurse ☐

4. Bleeding from back passage

Did you have this symptom?

Yes ☐ **Please complete A then B or C below**

No ☐ **Please go to Section 2**

A When did you first notice this? ☐☐ / ☐☐ / ☐☐ **OR** ☐

B When did you first tell your GP or nurse? ☐☐ / ☐☐ / ☐☐ **OR** ☐

OR

C Put a cross here if you didn't tell your GP or nurse ☐

Please give an exact date if you can. Otherwise give your best estimate (for example approximately how long ago, the month or the season). You may wish to refer to your diary or calendar if you have it with you.

Did you have any of the following?

1. Decrease in appetite

Did you have this?

Yes ☐ **Please complete A then B or C below**

No ☐ **Please go to Question 2**

A When did you first notice this? ☐☐ / ☐☐ / ☐☐ **OR** ☐

B When did you first tell your GP or nurse? ☐☐ / ☐☐ / ☐☐ **OR** ☐

OR

C Put a cross here if you didn't tell your GP or nurse ☐

2. Unexplained weight loss

Did you have this?

Yes ☐ **Please complete A then B or C below**

No ☐ **Please go to Question 3**

A When did you first notice this? ☐☐ / ☐☐ / ☐☐ **OR** ☐

B When did you first tell your GP or nurse? ☐☐ / ☐☐ / ☐☐ **OR** ☐

OR

C Put a cross here if you didn't tell your GP or nurse ☐

3. **Fatigue or tiredness that is unusual for you**

Did you have this?

Yes ☐ **Please complete A then B or C below**

No ☐ **Please go to Question 4**

A When did you first notice this? __ / __ / __ **OR** ____

B When did you first tell your GP or nurse? __ / __ / __ **OR** ____

OR

C Put a cross here if you didn't tell your GP or nurse ☐

4. **Feeling different 'in yourself' from usual**

Did you have this?

Yes ☐ **Please complete A then B or C below**

No ☐ **Please go to Question 5**

A When did you first notice this? __ / __ / __ **OR** ____

B When did you first tell your GP or nurse? __ / __ / __ **OR** ____

OR

C Put a cross here if you didn't tell your GP or nurse ☐

Please try and describe what the feeling was

If you had any further symptoms which you feel are relevant to your cancer diagnosis, please list them below. If not, please go to Question 7.

Please describe the symptom here and complete A then B or C below

5.

**A**	When did you first notice this?	☐☐ / ☐☐ / ☐☐ **OR** ☐
**B**	When did you first tell your GP or nurse?	☐☐ / ☐☐ / ☐☐ **OR** ☐
	**OR**	
**C**	Put a cross here if you didn't tell your GP or nurse	☐

If you had another symptom you feel is relevant, please write it below, otherwise go to Question 7.

Please describe the symptom here and complete A then B or C below

6.

**A**	When did you first notice this?	☐☐ / ☐☐ / ☐☐ **OR** ☐
**B**	When did you first tell your GP or nurse?	☐☐ / ☐☐ / ☐☐ **OR** ☐
	**OR**	
**C**	Put a cross here if you didn't tell your GP or nurse	☐

If you had further symptoms you would like to tell us about, there is space at the end of the Questionnaire for you to write them.

Did your GP send you for any tests?

Thinking back to the time immediately before you diagnosis, did your GP send you for any of the following tests?

Please cross the appropriate boxes

7. Were you sent for any of these?

	Yes	Not sure	No
Blood test(s)	☐	☐	☐
CT Scan	☐	☐	☐
Ultrasound scan	☐	☐	☐
Barium Enema	☐	☐	☐
Sigmoidoscopy or colonoscopy (Looking at bowel with internal camera)	☐	☐	☐
X-ray	☐	☐	☐

Please go to Question 8

About you

The next questions relate to general information about you. It would help us to know more about the background of the people completing the questionnaire.

8. Which best describes your employment status?

Please cross one box only

- Employed full-time ☐
- Employed part-time ☐
- Self employed full-time ☐
- Self employed part-time ☐
- Unemployed (seeking work) ☐
- Unemployed (not seeking work) ☐
- Retired ☐
- Student ☐
- Permanently sick/disabled ☐
- Temporarily sick/disabled ☐
- Looking after family/home ☐

Other, please describe

9. What is your highest level of qualification?

Please cross one box only

- Degree (or equivalent) ☐
- Diploma (or equivalent) ☐
- 'A' level ☐
- GCSE / 'O' level ☐
- None ☐

Other, please specify

10. **How would you describe your ethnicity**

Please cross one box only

White ☐
or
White British ☐
White Irish ☐
Other White background ☐

Mixed ☐
or
White & Black Carribean ☐
White & Black African ☐
White & Asian ☐
Other mixed background ☐

Black or Black British ☐
or
Carribean ☐
African ☐
Other Black background ☐

Asian or Asian British ☐
or
Indian ☐
Pakistani ☐
Bangladeshi ☐
Other Asian background ☐

Chinese or other ethnic group ☐
or
Chinese ☐
Other ethnic group ☐

11.

Do you live alone?

Yes ☐ **Please go to Question 12**

No ☐ **Please say who you live with below**

Who do you live with?
(e.g. wife, husband, partner, family member)

12. **Are you suffering from, or have you suffered from, any of the following in the past 2 years?**

Please cross any that are applicable to you

- Asthma ☐
- Chronic Obstructive Pulmonary Disease (COPD) ☐
- Other lung disease (e.g. fibrosis, bronchiectatis etc) ☐
- Heart disease ☐
- Anxiety or depression ☐
- Inflammatory bowel disease ☐
- Irritable bowel syndrome ☐
- Peptic ulcer ☐
- Previous cancer ☐
- Diabetes ☐
- Arthritis ☐

13. **About smoking**

Please cross the appropriate statement

- Are you a current smoker? ☐
- Are you an ex-smoker? ☐
- Are you a non-smoker (never smoked)? ☐

14. **Do you think you were more at risk of getting cancer because of your family history?**

Yes ☐
No ☐

57727

15. The following questions are about how completing the questionnaire made you feel.

A number of statements which people have used to describe themselves are given below. Read each statement and then cross the most appropriate box to indicate how you feel <u>right now, at the moment.</u> There are no right or wrong answers. Do not spend too much time on any one statement but give the answer which seems to describe your present feeling best.

		Not at all	Somewhat	Moderately	Very much
1.	I feel calm	☐	☐	☐	☐
2.	I am tense	☐	☐	☐	☐
3.	I feel upset	☐	☐	☐	☐
4.	I am relaxed	☐	☐	☐	☐
5.	I feel content	☐	☐	☐	☐
6.	I am worried	☐	☐	☐	☐

Please make sure you have answered all the questions.

If you have any other comments or there is anything else you would like to tell us about when you first noticed your symptoms, please do so using the space below.

If you feel you would like to talk to someone about this questionnaire at a later stage, please speak to the doctor or nurse who is looking after you, or be in touch with the research team, whose contact details are on your patient information sheet

Thank you for completing the questionnaire

Please give it to the research nurse

The next page is for the Researcher to complete

57727

| | | 1 | 0 |

Researcher - Post-completion Sheet

Researcher initials: ☐☐☐

Date questionnaire completed: Date ☐☐ / ☐☐ / ☐☐ Time ☐☐:☐☐

If not completed, give reason ☐

How long is it since the patient was told of his/her diagnosis? ☐

How long did it take the patient to complete the questionnaire?

- Less than 5 minutes ☐
- Between 5 and 10 minutes ☐
- Between 11 and 15 minutes ☐
- Longer than 15 minutes ☐

For the patient selected to complete the questionnaire on their own

Did the patient ask for any help? ☐ Yes ☐ No

If yes,

please specify what help was requested

____

____

please specify what help was given

____

____

For the patient selected for the researcher-administered questionnaire

Did the patient find any of the questions difficult? ☐ Yes ☐ No

If yes,

please specify which questions and what the difficulty was

____

____

____

____

Was the patient made anxious? ☐ Yes ☐ No

If yes,

please give details

____

____

____

____

Where did the patient complete the questionnaire?
(Please specify whether this was in a separate room alone, in a corner of the clinic, the ward or other

____

Thank you. Please now send the questionnaire and patient information sheet and consent form back to Cardiff University in the freepost envelope provided.

57727

PID - | | 1 | 1 |

Ymddiriedolaeth GIG Gogledd Orllewin Cymru
North West Wales NHS Trust

NHS WALES GIG CYMRU
Ymddiriedolaeth GIG Gogledd Cymru
North Wales NHS Trust

CID -
☐ GLAN CLWYD
☐ GWYNEDD
☐ MAELOR

Confidential Questionnaire

Looking At Your Symptoms Study

This Study has been funded by Cancer Research UK

Dear Patient

I am very grateful to you for agreeing to help us with this study.

I hope the information you supply will help us give people a diagnosis earlier.

If you feel you would like to talk to someone about this questionnaire at a later stage, please speak to the doctor or nurse who is looking after you, or be in touch with the research team, whose contact details are on your patient information sheet.

I can assure you that anything you tell us will be treated in the utmost confidence.

Yours sincerely

Dr Richard Neal
Senior Clinical Lecturer
Cardiff University
Chief Investigator, Symptoms Study

About your symptoms

These questions are about symptoms you noticed before your diagnosis. We are interested in symptoms which you think are related to your cancer.

Please put a cross in the appropriate box, or write an answer as required.

Looking back...

1. Did you have any symptoms that you feel were due to your cancer before you were diagnosed?

Yes ☐ **Please continue with Question 2**

No ☐ **Please go to Section 2 on Page 5**

2. What was the first symptom that made you think something might be wrong?

Please go to Question 3

The following questions are about when you first noticed a symptom and when you first told your GP or nurse about it.

Please give an exact date if you can. Otherwise give your best estimate. (For example approximately how long ago, the month or season). You wish to refer to your diary or calendar if you have it with you.

Here is an example question:

Decrease in appetite

Did you have this symptom?

Yes ☒ **Please complete A then B or C below**

No ☐ **Please go to Section 2**

A When did you first notice this? d d / m m / y y **OR** Estimate '3 months ago' or 'June'

B When did you first tell your GP or nurse? 2 3 / 1 2 / 0 7 **OR** Estimate

OR

C Put a cross here if you didn't tell your GP or nurse ☐

3. Change in bowel habit

Did you have this symptom?

Yes ☐ **Please complete A then B or C below**

No ☐ **Please go to Question 4**

A When did you first notice this? ☐☐ / ☐☐ / ☐☐ **OR** ☐

B When did you first tell your GP or nurse? ☐☐ / ☐☐ / ☐☐ **OR** ☐

OR

C Put a cross here if you didn't tell your GP or nurse ☐

4. Bleeding from back passage

Did you have this symptom?

Yes ☐ **Please complete A then B or C below**

No ☐ **Please go to Section 2**

A When did you first notice this? ☐☐ / ☐☐ / ☐☐ **OR** ☐

B When did you first tell your GP or nurse? ☐☐ / ☐☐ / ☐☐ **OR** ☐

OR

C Put a cross here if you didn't tell your GP or nurse ☐

Section 2

Please give an <u>exact</u> date if you can. Otherwise give your best <u>estimate</u> (for example approximately how long ago, the month or the season). You may wish to refer to your diary or calendar if you have it with you.

Did you have any of the following?

1. Decrease in appetite

Did you have this?

Yes ☐ **Please complete A then B or C below**

No ☐ **Please go to Question 2**

A When did you <u>first notice</u> this? ☐☐ / ☐☐ / ☐☐ **OR** ☐

B When did you <u>first tell</u> your GP or nurse? ☐☐ / ☐☐ / ☐☐ **OR** ☐

OR

C Put a cross here if you <u>didn't tell</u> your GP or nurse ☐

2. Unexplained weight loss

Did you have this?

Yes ☐ **Please complete A then B or C below**

No ☐ **Please go to Question 3**

A When did you <u>first notice</u> this? ☐☐ / ☐☐ / ☐☐ **OR** ☐

B When did you <u>first tell</u> your GP or nurse? ☐☐ / ☐☐ / ☐☐ **OR** ☐

OR

C Put a cross here if you <u>didn't tell</u> your GP or nurse ☐

3. **Fatigue or tiredness that is unusual for you**

Did you have this?

Yes ☐ **Please complete A then B or C below**

No ☐ **Please go to Question 4**

A When did you first notice this? ☐☐ / ☐☐ / ☐☐ **OR** ☐

B When did you first tell your GP or nurse? ☐☐ / ☐☐ / ☐☐ **OR** ☐

OR

C Put a cross here if you didn't tell your GP or nurse ☐

4. **Feeling different 'in yourself' from usual**

Did you have this?

Yes ☐ **Please complete A then B or C below**

No ☐ **Please go to Question 5**

A When did you first notice this? ☐☐ / ☐☐ / ☐☐ **OR** ☐

B When did you first tell your GP or nurse? ☐☐ / ☐☐ / ☐☐ **OR** ☐

OR

C Put a cross here if you didn't tell your GP or nurse ☐

Please try and describe what the feeling was

If you had any further symptoms which you feel are relevant to your cancer diagnosis, please list them below. If not, please go to Question 7.

Please describe the symptom here and complete A then B or C below

5.

**A**	When did you first notice this?	☐☐ / ☐☐ / ☐☐ **OR** ☐
**B**	When did you first tell your GP or nurse?	☐☐ / ☐☐ / ☐☐ **OR** ☐
	**OR**	
**C**	Put a cross here if you didn't tell your GP or nurse	☐

If you had another symptom you feel is relevant, please write it below, otherwise go to Question 7.

Please describe the symptom here and complete A then B or C below

6.

**A**	When did you first notice this?	☐☐ / ☐☐ / ☐☐ **OR** ☐
**B**	When did you first tell your GP or nurse?	☐☐ / ☐☐ / ☐☐ **OR** ☐
	**OR**	
**C**	Put a cross here if you didn't tell your GP or nurse	☐

If you had further symptoms you would like to tell us about, there is space at the end of the Questionnaire for you to write them.

Did your GP send you for any tests?

Thinking back to the time immediately before you diagnosis, did your GP send you for any of the following tests?

Please cross the appropriate boxes

7. Were you sent for any of these?

	Yes	Not sure	No
Blood test(s)	☐	☐	☐
CT Scan	☐	☐	☐
Ultrasound scan	☐	☐	☐
Barium Enema	☐	☐	☐
Sigmoidoscopy or colonoscopy (Looking at bowel with internal camera)	☐	☐	☐
X-ray	☐	☐	☐

Please go to Question 8

About you

The next questions relate to general information about you. It would help us to know more about the background of the people completing the questionnaire.

8. Which best describes your employment status?

Please cross one box only

- Employed full-time ☐
- Employed part-time ☐
- Self employed full-time ☐
- Self employed part-time ☐
- Unemployed (seeking work) ☐
- Unemployed (not seeking work) ☐
- Retired ☐
- Student ☐
- Permanently sick/disabled ☐
- Temporarily sick/disabled ☐
- Looking after family/home ☐
- Other, please describe ______

9. What is your highest level of qualification?

Please cross one box only

- Degree (or equivalent) ☐
- Diploma (or equivalent) ☐
- 'A' level ☐
- GCSE / 'O' level ☐
- None ☐
- Other, please specify ______

10. **How would you describe your ethnicity**

Please cross one box only

White ☐
or
White British ☐
White Irish ☐
Other White background ☐

Mixed ☐
or
White & Black Carribean ☐
White & Black African ☐
White & Asian ☐
Other mixed background ☐

Black or Black British ☐
or
Carribean ☐
African ☐
Other Black background ☐

Asian or Asian British ☐
or
Indian ☐
Pakistani ☐
Bangladeshi ☐
Other Asian background ☐

Chinese or other ethnic group ☐
or
Chinese ☐
Other ethnic group ☐

11.

Do you live alone?

Yes ☐ **Please go to Question 12**

No ☐ **Please say who you live with below**

Who do you live with?
(e.g. wife, husband, partner, family member)

12. **Are you suffering from, or have you suffered from, any of the following in the past 2 years?**

Please cross any that are applicable to you

- Asthma ☐
- Chronic Obstructive Pulmonary Disease (COPD) ☐
- Other lung disease (e.g. fibrosis, bronchiectatis etc) ☐
- Heart disease ☐
- Anxiety or depression ☐
- Inflammatory bowel disease ☐
- Irritable bowel syndrome ☐
- Peptic ulcer ☐
- Previous cancer ☐
- Diabetes ☐
- Arthritis ☐

13. **About smoking**

Please cross the appropriate statement

- Are you a current smoker? ☐
- Are you an ex-smoker? ☐
- Are you a non-smoker (never smoked)? ☐

14. **Do you think you were more at risk of getting cancer because of your family history?**

Yes ☐

No ☐

15. **The following questions are about how completing the questionnaire made you feel.**

A number of statements which people have used to describe themselves are given below. Read each statement and then cross the most appropriate box to indicate how you feel right now, at the moment. There are no right or wrong answers. Do not spend too much time on any one statement but give the answer which seems to describe your present feeling best.

		Not at all	Somewhat	Moderately	Very much
1.	I feel calm	☐	☐	☐	☐
2.	I am tense	☐	☐	☐	☐
3.	I feel upset	☐	☐	☐	☐
4.	I am relaxed	☐	☐	☐	☐
5.	I feel content	☐	☐	☐	☐
6.	I am worried	☐	☐	☐	☐

Please make sure you have answered all the questions.

If you have any other comments or there is anything else you would like to tell us about when you first noticed your symptoms, please do so using the space below.

If you feel you would like to talk to someone about this questionnaire at a later stage, please speak to the doctor or nurse who is looking after you, or be in touch with the research team, whose contact details are on your patient information sheet

Thank you for completing the questionnaire

Please give it to the research nurse

The next page is for the Researcher to complete

Researcher - Post-completion Sheet

Researcher initials: ☐☐☐

Date questionnaire completed: Date ☐☐ / ☐☐ / ☐☐ Time ☐☐:☐☐

If not completed, give reason ☐

How long is it since the patient was told of his/her diagnosis? ☐

How long did it take the patient to complete the questionnaire?

- Less than 5 minutes ☐
- Between 5 and 10 minutes ☐
- Between 11 and 15 minutes ☐
- Longer than 15 minutes ☐

For the patient selected to complete the questionnaire on their own

Did the patient ask for any help? ☐ Yes ☐ No

If yes,

please specify what help was requested

____

____

please specify what help was given

____

____

For the patient selected for the researcher-administered questionnaire

Did the patient find any of the questions difficult? ☐ Yes ☐ No

If yes,

please specify which questions and what the difficulty was

____

____

____

____

Was the patient made anxious? ☐ Yes ☐ No

If yes,

please give details

____

____

____

____

Where did the patient complete the questionnaire?
(Please specify whether this was in a separate room alone, in a corner of the clinic, the ward or other

____

Thank you. Please now send the questionnaire and patient information sheet and consent form back to Cardiff University in the freepost envelope provided.

57727

PID - | | 1 | 2 |

☐ GLAN CLWYD

CID - ☐ GWYNEDD

☐ MAELOR

Confidential Questionnaire

Looking At Your Symptoms Study

This Study has been funded by Cancer Research UK

Dear Patient

I am very grateful to you for agreeing to help us with this study.

I hope the information you supply will help us give people a diagnosis earlier.

If you feel you would like to talk to someone about this questionnaire at a later stage, please speak to the doctor or nurse who is looking after you, or be in touch with the research team, whose contact details are on your patient information sheet.

I can assure you that anything you tell us will be treated in the utmost confidence.

Yours sincerely

Dr Richard Neal
Senior Clinical Lecturer
Cardiff University
Chief Investigator, Symptoms Study

About your symptoms

These questions are about symptoms you noticed before your diagnosis. We are interested in symptoms which you think are related to your cancer.

Please put a cross in the appropriate box, or write an answer as required.

Looking back...

1. Did you have any symptoms that you feel were due to your cancer before you were diagnosed?

Yes ☐ **Please continue with Question 2**

No ☐ **Please go to Section 2 on Page 5**

2. What was the first symptom that made you think something might be wrong?

☐

Please go to Question 3

The following questions are about when you first noticed a symptom and when you first told your GP or nurse about it.

Please give an exact date if you can. Otherwise give your best estimate. (For example approximately how long ago, the month or season). You wish to refer to your diary or calendar if you have it with you.

Here is an example question:

Decrease in appetite

Did you have this symptom?

Yes ☒ **Please complete A then B or C below**

No ☐ **Please go to Section 2**

A When did you first notice this? d d / m m / y y **OR** Estimate '3 months ago' or 'June'

B When did you first tell your GP or nurse? 2 3 / 1 2 / 0 7 **OR** Estimate

OR

C Put a cross here if you didn't tell your GP or nurse ☐

3. Change in bowel habit

Did you have this symptom?

Yes ☐ **Please complete A then B or C below**

No ☐ **Please go to Question 4**

A When did you first notice this? ☐☐ / ☐☐ / ☐☐ **OR** ☐

B When did you first tell your GP or nurse? ☐☐ / ☐☐ / ☐☐ **OR** ☐

OR

C Put a cross here if you didn't tell your GP or nurse ☐

4. Bleeding from back passage

Did you have this symptom?

Yes ☐ **Please complete A then B or C below**

No ☐ **Please go to Section 2**

A When did you first notice this? ☐☐ / ☐☐ / ☐☐ **OR** ☐

B When did you first tell your GP or nurse? ☐☐ / ☐☐ / ☐☐ **OR** ☐

OR

C Put a cross here if you didn't tell your GP or nurse ☐

Please give an exact date if you can. Otherwise give your best estimate (for example approximately how long ago, the month or the season). You may wish to refer to your diary or calendar if you have it with you.

Did you have any of the following?

1. Decrease in appetite

Did you have this?

Yes ☐ **Please complete A then B or C below**

No ☐ **Please go to Question 2**

A When did you first notice this? ☐☐ / ☐☐ / ☐☐ **OR** ☐

B When did you first tell your GP or nurse? ☐☐ / ☐☐ / ☐☐ **OR** ☐

OR

C Put a cross here if you didn't tell your GP or nurse ☐

2. Unexplained weight loss

Did you have this?

Yes ☐ **Please complete A then B or C below**

No ☐ **Please go to Question 3**

A When did you first notice this? ☐☐ / ☐☐ / ☐☐ **OR** ☐

B When did you first tell your GP or nurse? ☐☐ / ☐☐ / ☐☐ **OR** ☐

OR

C Put a cross here if you didn't tell your GP or nurse ☐

3. **Fatigue or tiredness that is unusual for you**

Did you have this?

Yes ☐ **Please complete A then B or C below**

No ☐ **Please go to Question 4**

A When did you first notice this? ☐☐ / ☐☐ / ☐☐ **OR** ☐

B When did you first tell your GP or nurse? ☐☐ / ☐☐ / ☐☐ **OR** ☐

OR

C Put a cross here if you didn't tell your GP or nurse ☐

4. **Feeling different 'in yourself' from usual**

Did you have this?

Yes ☐ **Please complete A then B or C below**

No ☐ **Please go to Question 5**

A When did you first notice this? ☐☐ / ☐☐ / ☐☐ **OR** ☐

B When did you first tell your GP or nurse? ☐☐ / ☐☐ / ☐☐ **OR** ☐

OR

C Put a cross here if you didn't tell your GP or nurse ☐

Please try and describe what the feeling was

If you had any further symptoms which you feel are relevant to your cancer diagnosis, please list them below. If not, please go to Question 7.

Please describe the symptom here and complete A then B or C below

5.

**A**	When did you first notice this?	_ _ / _ _ / _ _ **OR**
**B**	When did you first tell your GP or nurse?	_ _ / _ _ / _ _ **OR**
	**OR**	
**C**	Put a cross here if you didn't tell your GP or nurse	☐

If you had another symptom you feel is relevant, please write it below, otherwise go to Question 7.

Please describe the symptom here and complete A then B or C below

6.

**A**	When did you first notice this?	_ _ / _ _ / _ _ **OR**
**B**	When did you first tell your GP or nurse?	_ _ / _ _ / _ _ **OR**
	**OR**	
**C**	Put a cross here if you didn't tell your GP or nurse	☐

If you had further symptoms you would like to tell us about, there is space at the end of the Questionnaire for you to write them.

Did your GP send you for any tests?

Thinking back to the time immediately before you diagnosis, did your GP send you for any of the following tests?

Please cross the appropriate boxes

7. Were you sent for any of these?

	Yes	Not sure	No
Blood test(s)	☐	☐	☐
CT Scan	☐	☐	☐
Ultrasound scan	☐	☐	☐
Barium Enema	☐	☐	☐
Sigmoidoscopy or colonoscopy (Looking at bowel with internal camera)	☐	☐	☐
X-ray	☐	☐	☐

Please go to Question 8

About you

The next questions relate to general information about you. It would help us to know more about the background of the people completing the questionnaire.

8. **Which best describes your employment status?**

Please cross one box only

- Employed full-time ☐
- Employed part-time ☐
- Self employed full-time ☐
- Self employed part-time ☐
- Unemployed (seeking work) ☐
- Unemployed (not seeking work) ☐
- Retired ☐
- Student ☐
- Permanently sick/disabled ☐
- Temporarily sick/disabled ☐
- Looking after family/home ☐
- Other, please describe ________

9. **What is your highest level of qualification?**

Please cross one box only

- Degree (or equivalent) ☐
- Diploma (or equivalent) ☐
- 'A' level ☐
- GCSE / 'O' level ☐
- None ☐
- Other, please specify ________

10. How would you describe your ethnicity

Please cross one box only

White ☐
or
- White British ☐
- White Irish ☐
- Other White background ☐

Mixed ☐
or
- White & Black Carribean ☐
- White & Black African ☐
- White & Asian ☐
- Other mixed background ☐

Black or Black British ☐
or
- Carribean ☐
- African ☐
- Other Black background ☐

Asian or Asian British ☐
or
- Indian ☐
- Pakistani ☐
- Bangladeshi ☐
- Other Asian background ☐

Chinese or other ethnic group ☐
or
- Chinese ☐
- Other ethnic group ☐

11.

Do you live alone?

Yes ☐ **Please go to Question 12**

No ☐ **Please say who you live with below**

Who do you live with?
(e.g. wife, husband, partner, family member)

12. **Are you suffering from, or have you suffered from, any of the following in the past 2 years?**

Please cross any that are applicable to you

- Asthma ☐
- Chronic Obstructive Pulmonary Disease (COPD) ☐
- Other lung disease (e.g. fibrosis, bronchiectatis etc) ☐
- Heart disease ☐
- Anxiety or depression ☐
- Inflammatory bowel disease ☐
- Irritable bowel syndrome ☐
- Peptic ulcer ☐
- Previous cancer ☐
- Diabetes ☐
- Arthritis ☐

13. **About smoking**

Please cross the appropriate statement

- Are you a current smoker? ☐
- Are you an ex-smoker? ☐
- Are you a non-smoker (never smoked)? ☐

14. **Do you think you were more at risk of getting cancer because of your family history?**

Yes ☐

No ☐

15. The following questions are about how completing the questionnaire made you feel.

A number of statements which people have used to describe themselves are given below. Read each statement and then cross the most appropriate box to indicate how you feel right now, at the moment. There are no right or wrong answers. Do not spend too much time on any one statement but give the answer which seems to describe your present feeling best.

		Not at all	Somewhat	Moderately	Very much
1.	I feel calm	☐	☐	☐	☐
2.	I am tense	☐	☐	☐	☐
3.	I feel upset	☐	☐	☐	☐
4.	I am relaxed	☐	☐	☐	☐
5.	I feel content	☐	☐	☐	☐
6.	I am worried	☐	☐	☐	☐

Please make sure you have answered all the questions.

If you have any other comments or there is anything else you would like to tell us about when you first noticed your symptoms, please do so using the space below.

If you feel you would like to talk to someone about this questionnaire at a later stage, please speak to the doctor or nurse who is looking after you, or be in touch with the research team, whose contact details are on your patient information sheet

Thank you for completing the questionnaire

Please give it to the research nurse

The next page is for the Researcher to complete

Researcher - Post-completion Sheet

Researcher initials: ☐☐☐

Date questionnaire completed: Date ☐☐ / ☐☐ / ☐☐ Time ☐☐ : ☐☐

If not completed, give reason ☐

How long is it since the patient was told of his/her diagnosis? ☐

How long did it take the patient to complete the questionnaire?

- Less than 5 minutes ☐
- Between 5 and 10 minutes ☐
- Between 11 and 15 minutes ☐
- Longer than 15 minutes ☐

For the patient selected to complete the questionnaire on their own

Did the patient ask for any help? ☐ Yes ☐ No

If yes,

please specify what help was requested

____

____

please specify what help was given

____

____

For the patient selected for the researcher-administered questionnaire

Did the patient find any of the questions difficult? ☐ Yes ☐ No

If yes,

please specify which questions and what the difficulty was

____

____

____

____

Was the patient made anxious? ☐ Yes ☐ No

If yes,

please give details

____

____

____

____

Where did the patient complete the questionnaire?
(Please specify whether this was in a separate room alone, in a corner of the clinic, the ward or other

____

Thank you. Please now send the questionnaire and patient information sheet and consent form back to Cardiff University in the freepost envelope provided.

57727

PID - | | 1 | 3 |

Ymddiriedolaeth GIG Gogledd Orllewin Cymru
North West Wales NHS Trust

NHS WALES GIG CYMRU
Ymddiriedolaeth GIG Gogledd Cymru
North Wales NHS Trust

☐ GLAN CLWYD

CID - ☐ GWYNEDD

☐ MAELOR

Confidential Questionnaire

Looking At Your Symptoms Study

This Study has been funded by Cancer Research UK

Cardiff University / Prifysgol Caerdydd
Ysgol Glinigol Gogledd Cymru / North Wales Clinical School
Cancer Research UK
sewtu South East Wales Trials Unit / Uned Ymchwil De-ddwyrain Cymru

Dear Patient

I am very grateful to you for agreeing to help us with this study.

I hope the information you supply will help us give people a diagnosis earlier.

If you feel you would like to talk to someone about this questionnaire at a later stage, please speak to the doctor or nurse who is looking after you, or be in touch with the research team, whose contact details are on your patient information sheet.

I can assure you that anything you tell us will be treated in the utmost confidence.

Yours sincerely

Dr Richard Neal
Senior Clinical Lecturer
Cardiff University
Chief Investigator, Symptoms Study

About your symptoms

These questions are about symptoms you noticed before your diagnosis. We are interested in symptoms which you think are related to your cancer.

Please put a cross in the appropriate box, or write an answer as required.

Looking back...

1. Did you have any symptoms that you feel were due to your cancer before you were diagnosed?

Yes ☐ **Please continue with Question 2**

No ☐ **Please go to Section 2 on Page 5**

2. What was the first symptom that made you think something might be wrong?

☐

Please go to Question 3

The following questions are about when you first noticed a symptom and when you first told your GP or nurse about it.

Please give an exact date if you can. Otherwise give your best estimate. (For example approximately how long ago, the month or season). You wish to refer to your diary or calendar if you have it with you.

Here is an example question:

Decrease in appetite

Did you have this symptom?

Yes ☒ **Please complete A then B or C below**

No ☐ **Please go to Section 2**

A When did you first notice this? d d / m m / y y **OR** Estimate '3 months ago' or 'June'

B When did you first tell your GP or nurse? 2 3 / 1 2 / 0 7 **OR** Estimate

OR

C Put a cross here if you didn't tell your GP or nurse ☐

3. Change in bowel habit

Did you have this symptom?

Yes ☐ **Please complete A then B or C below**

No ☐ **Please go to Question 4**

A When did you first notice this? ☐☐ / ☐☐ / ☐☐ **OR** ☐

B When did you first tell your GP or nurse? ☐☐ / ☐☐ / ☐☐ **OR** ☐

OR

C Put a cross here if you didn't tell your GP or nurse ☐

4. Bleeding from back passage

Did you have this symptom?

Yes ☐ **Please complete A then B or C below**

No ☐ **Please go to Section 2**

A When did you first notice this? ☐☐ / ☐☐ / ☐☐ **OR** ☐

B When did you first tell your GP or nurse? ☐☐ / ☐☐ / ☐☐ **OR** ☐

OR

C Put a cross here if you didn't tell your GP or nurse ☐

Section 2

Please give an exact date if you can. Otherwise give your best estimate (for example approximately how long ago, the month or the season). You may wish to refer to your diary or calendar if you have it with you.

Did you have any of the following?

1. Decrease in appetite

Did you have this?

Yes ☐ **Please complete A then B or C below**

No ☐ **Please go to Question 2**

A When did you first notice this? ☐☐ / ☐☐ / ☐☐ **OR** ☐

B When did you first tell your GP or nurse? ☐☐ / ☐☐ / ☐☐ **OR** ☐

OR

C Put a cross here if you didn't tell your GP or nurse ☐

2. Unexplained weight loss

Did you have this?

Yes ☐ **Please complete A then B or C below**

No ☐ **Please go to Question 3**

A When did you first notice this? ☐☐ / ☐☐ / ☐☐ **OR** ☐

B When did you first tell your GP or nurse? ☐☐ / ☐☐ / ☐☐ **OR** ☐

OR

C Put a cross here if you didn't tell your GP or nurse ☐

3. **Fatigue or tiredness that is unusual for you**

Did you have this?

Yes ☐ **Please complete A then B or C below**

No ☐ **Please go to Question 4**

A When did you first notice this? ☐☐ / ☐☐ / ☐☐ **OR** ☐

B When did you first tell your GP or nurse? ☐☐ / ☐☐ / ☐☐ **OR** ☐

OR

C Put a cross here if you didn't tell your GP or nurse ☐

4. **Feeling different 'in yourself' from usual**

Did you have this?

Yes ☐ **Please complete A then B or C below**

No ☐ **Please go to Question 5**

A When did you first notice this? ☐☐ / ☐☐ / ☐☐ **OR** ☐

B When did you first tell your GP or nurse? ☐☐ / ☐☐ / ☐☐ **OR** ☐

OR

C Put a cross here if you didn't tell your GP or nurse ☐

Please try and describe what the feeling was

If you had any further symptoms which you feel are relevant to your cancer diagnosis, please list them below. If not, please go to Question 7.

Please describe the symptom here and complete A then B or C below

5.

**A**	When did you first notice this?	☐☐ / ☐☐ / ☐☐ **OR** ☐
**B**	When did you first tell your GP or nurse?	☐☐ / ☐☐ / ☐☐ **OR** ☐
	**OR**	
**C**	Put a cross here if you didn't tell your GP or nurse	☐

If you had another symptom you feel is relevant, please write it below, otherwise go to Question 7.

Please describe the symptom here and complete A then B or C below

6.

**A**	When did you first notice this?	☐☐ / ☐☐ / ☐☐ **OR** ☐
**B**	When did you first tell your GP or nurse?	☐☐ / ☐☐ / ☐☐ **OR** ☐
	**OR**	
**C**	Put a cross here if you didn't tell your GP or nurse	☐

If you had further symptoms you would like to tell us about, there is space at the end of the Questionnaire for you to write them.

Did your GP send you for any tests?

Thinking back to the time immediately before you diagnosis, did your GP send you for any of the following tests?

Please cross the appropriate boxes

7. Were you sent for any of these?

	Yes	Not sure	No
Blood test(s)	☐	☐	☐
CT Scan	☐	☐	☐
Ultrasound scan	☐	☐	☐
Barium Enema	☐	☐	☐
Sigmoidoscopy or colonoscopy (Looking at bowel with internal camera)	☐	☐	☐
X-ray	☐	☐	☐

Please go to Question 8

About you

The next questions relate to general information about you. It would help us to know more about the background of the people completing the questionnaire.

8. Which best describes your employment status?

Please cross one box only

- Employed full-time ☐
- Employed part-time ☐
- Self employed full-time ☐
- Self employed part-time ☐
- Unemployed (seeking work) ☐
- Unemployed (not seeking work) ☐
- Retired ☐
- Student ☐
- Permanently sick/disabled ☐
- Temporarily sick/disabled ☐
- Looking after family/home ☐

Other, please describe

9. What is your highest level of qualification?

Please cross one box only

- Degree (or equivalent) ☐
- Diploma (or equivalent) ☐
- 'A' level ☐
- GCSE / 'O' level ☐
- None ☐

Other, please specify

10. **How would you describe your ethnicity**

Please cross one box only

White ☐
or
- White British ☐
- White Irish ☐
- Other White background ☐

Mixed ☐
or
- White & Black Carribean ☐
- White & Black African ☐
- White & Asian ☐
- Other mixed background ☐

Black or Black British ☐
or
- Carribean ☐
- African ☐
- Other Black background ☐

Asian or Asian British ☐
or
- Indian ☐
- Pakistani ☐
- Bangladeshi ☐
- Other Asian background ☐

Chinese or other ethnic group ☐
or
- Chinese ☐
- Other ethnic group ☐

11.

Do you live alone?

Yes ☐ **Please go to Question 12**

No ☐ **Please say who you live with below**

Who do you live with?
(e.g. wife, husband, partner, family member)

12. Are you suffering from, or have you suffered from, any of the following in the past 2 years?

Please cross any that are applicable to you

- Asthma ☐
- Chronic Obstructive Pulmonary Disease (COPD) ☐
- Other lung disease (e.g. fibrosis, bronchiectatis etc) ☐
- Heart disease ☐
- Anxiety or depression ☐
- Inflammatory bowel disease ☐
- Irritable bowel syndrome ☐
- Peptic ulcer ☐
- Previous cancer ☐
- Diabetes ☐
- Arthritis ☐

13. About smoking

Please cross the appropriate statement

- Are you a current smoker? ☐
- Are you an ex-smoker? ☐
- Are you a non-smoker (never smoked)? ☐

14. Do you think you were more at risk of getting cancer because of your family history?

Yes ☐

No ☐

15. **The following questions are about how completing the questionnaire made you feel.**

A number of statements which people have used to describe themselves are given below. Read each statement and then cross the most appropriate box to indicate how you feel right now, at the moment. There are no right or wrong answers. Do not spend too much time on any one statement but give the answer which seems to describe your present feeling best.

		Not at all	Somewhat	Moderately	Very much
1.	I feel calm	☐	☐	☐	☐
2.	I am tense	☐	☐	☐	☐
3.	I feel upset	☐	☐	☐	☐
4.	I am relaxed	☐	☐	☐	☐
5.	I feel content	☐	☐	☐	☐
6.	I am worried	☐	☐	☐	☐

Please make sure you have answered all the questions.

If you have any other comments or there is anything else you would like to tell us about when you first noticed your symptoms, please do so using the space below.

If you feel you would like to talk to someone about this questionnaire at a later stage, please speak to the doctor or nurse who is looking after you, or be in touch with the research team, whose contact details are on your patient information sheet

Thank you for completing the questionnaire

Please give it to the research nurse

The next page is for the Researcher to complete

Researcher - Post-completion Sheet

Researcher initials: ☐☐☐

Date questionnaire completed: Date ☐☐ / ☐☐ / ☐☐ Time ☐☐:☐☐

If not completed, give reason ☐

How long is it since the patient was told of his/her diagnosis? ☐

How long did it take the patient to complete the questionnaire?

- Less than 5 minutes ☐
- Between 5 and 10 minutes ☐
- Between 11 and 15 minutes ☐
- Longer than 15 minutes ☐

For the patient selected to complete the questionnaire on their own

Did the patient ask for any help? ☐ Yes ☐ No

If yes,

please specify what help was requested

____

____

please specify what help was given

____

____

For the patient selected for the researcher-administered questionnaire

Did the patient find any of the questions difficult? ☐ Yes ☐ No

If yes,

please specify which questions and what the difficulty was

____

____

____

____

Was the patient made anxious? ☐ Yes ☐ No

If yes,

please give details

____

____

____

____

Where did the patient complete the questionnaire?
(Please specify whether this was in a separate room alone, in a corner of the clinic, the ward or other

____

Thank you. Please now send the questionnaire and patient information sheet and consent form back to Cardiff University in the freepost envelope provided.

57727

PID - | | 1 | 4 |

Ymddiriedolaeth GIG Gogledd Orllewin Cymru
North West Wales NHS Trust

NHS WALES GIG CYMRU
Ymddiriedolaeth GIG Gogledd Cymru
North Wales NHS Trust

CID -
☐ GLAN CLWYD
☐ GWYNEDD
☐ MAELOR

Confidential Questionnaire

Looking At Your Symptoms Study

This Study has been funded by Cancer Research UK

CARDIFF UNIVERSITY PRIFYSGOL CAERDYDD
Ysgol Glinigol Gogledd Cymru North Wales Clinical School
CANCER RESEARCH UK
sewtu South East Wales Trials Unit Uned Ymchwil De-ddwyrain Cymru

Dear Patient

I am very grateful to you for agreeing to help us with this study.

I hope the information you supply will help us give people a diagnosis earlier.

If you feel you would like to talk to someone about this questionnaire at a later stage, please speak to the doctor or nurse who is looking after you, or be in touch with the research team, whose contact details are on your patient information sheet.

I can assure you that anything you tell us will be treated in the utmost confidence.

Yours sincerely

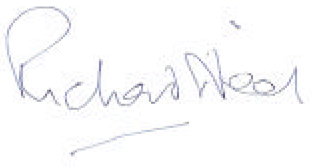

Dr Richard Neal
Senior Clinical Lecturer
Cardiff University
Chief Investigator, Symptoms Study

About your symptoms

These questions are about symptoms you noticed before your diagnosis. We are interested in symptoms which you think are related to your cancer.

Please put a cross in the appropriate box, or write an answer as required.

Looking back...

1. Did you have any symptoms that you feel were due to your cancer before you were diagnosed?

Yes ☐ **Please continue with Question 2**

No ☐ **Please go to Section 2 on Page 5**

2. What was the first symptom that made you think something might be wrong?

☐

Please go to Question 3

The following questions are about when you first noticed a symptom and when you first told your GP or nurse about it.

Please give an exact date if you can. Otherwise give your best estimate. (For example approximately how long ago, the month or season). You wish to refer to your diary or calendar if you have it with you.

Here is an example question:

Decrease in appetite

Did you have this symptom?

Yes ☒ **Please complete A then B or C below**

No ☐ **Please go to Section 2**

A When did you first notice this? d d / m m / y y **OR** Estimate '3 months ago' or 'June'

B When did you first tell your GP or nurse? 2 3 / 1 2 / 0 7 **OR** Estimate

OR

C Put a cross here if you didn't tell your GP or nurse ☐

3. Change in bowel habit

Did you have this symptom?

Yes ☐ **Please complete A then B or C below**

No ☐ **Please go to Question 4**

A When did you first notice this? ☐☐ / ☐☐ / ☐☐ **OR** ☐

B When did you first tell your GP or nurse? ☐☐ / ☐☐ / ☐☐ **OR** ☐

OR

C Put a cross here if you didn't tell your GP or nurse ☐

4. Bleeding from back passage

Did you have this symptom?

Yes ☐ **Please complete A then B or C below**

No ☐ **Please go to Section 2**

A When did you first notice this? ☐☐ / ☐☐ / ☐☐ **OR** ☐

B When did you first tell your GP or nurse? ☐☐ / ☐☐ / ☐☐ **OR** ☐

OR

C Put a cross here if you didn't tell your GP or nurse ☐

Please give an exact date if you can. Otherwise give your best estimate (for example approximately how long ago, the month or the season). You may wish to refer to your diary or calendar if you have it with you.

Did you have any of the following?

1. Decrease in appetite

Did you have this?

Yes ☐ **Please complete A then B or C below**

No ☐ **Please go to Question 2**

A When did you first notice this? ☐☐ / ☐☐ / ☐☐ **OR** ☐

B When did you first tell your GP or nurse? ☐☐ / ☐☐ / ☐☐ **OR** ☐

OR

C Put a cross here if you didn't tell your GP or nurse ☐

2. Unexplained weight loss

Did you have this?

Yes ☐ **Please complete A then B or C below**

No ☐ **Please go to Question 3**

A When did you first notice this? ☐☐ / ☐☐ / ☐☐ **OR** ☐

B When did you first tell your GP or nurse? ☐☐ / ☐☐ / ☐☐ **OR** ☐

OR

C Put a cross here if you didn't tell your GP or nurse ☐

3. **Fatigue or tiredness that is unusual for you**

Did you have this?

Yes ☐ **Please complete A then B or C below**

No ☐ **Please go to Question 4**

A When did you first notice this? ☐☐ / ☐☐ / ☐☐ **OR** ☐

B When did you first tell your GP or nurse? ☐☐ / ☐☐ / ☐☐ **OR** ☐

OR

C Put a cross here if you didn't tell your GP or nurse ☐

4. **Feeling different 'in yourself' from usual**

Did you have this?

Yes ☐ **Please complete A then B or C below**

No ☐ **Please go to Question 5**

A When did you first notice this? ☐☐ / ☐☐ / ☐☐ **OR** ☐

B When did you first tell your GP or nurse? ☐☐ / ☐☐ / ☐☐ **OR** ☐

OR

C Put a cross here if you didn't tell your GP or nurse ☐

Please try and describe what the feeling was

If you had any further symptoms which you feel are relevant to your cancer diagnosis, please list them below. If not, please go to Question 7.

Please describe the symptom here and complete A then B or C below

5.

**A**	When did you first notice this?	__ / __ / __ **OR**
**B**	When did you first tell your GP or nurse?	__ / __ / __ **OR**
	**OR**	
**C**	Put a cross here if you didn't tell your GP or nurse	☐

If you had another symptom you feel is relevant, please write it below, otherwise go to Question 7.

Please describe the symptom here and complete A then B or C below

6.

**A**	When did you first notice this?	__ / __ / __ **OR**
**B**	When did you first tell your GP or nurse?	__ / __ / __ **OR**
	**OR**	
**C**	Put a cross here if you didn't tell your GP or nurse	☐

If you had further symptoms you would like to tell us about, there is space at the end of the Questionnaire for you to write them.

Did your GP send you for any tests?

Thinking back to the time immediately before you diagnosis, did your GP send you for any of the following tests?

Please cross the appropriate boxes

7. Were you sent for any of these?

	Yes	Not sure	No
Blood test(s)	☐	☐	☐
CT Scan	☐	☐	☐
Ultrasound scan	☐	☐	☐
Barium Enema	☐	☐	☐
Sigmoidoscopy or colonoscopy (Looking at bowel with internal camera)	☐	☐	☐
X-ray	☐	☐	☐

Please go to Question 8

About you

The next questions relate to general information about you. It would help us to know more about the background of the people completing the questionnaire.

8. Which best describes your employment status?

Please cross one box only

- Employed full-time ☐
- Employed part-time ☐
- Self employed full-time ☐
- Self employed part-time ☐
- Unemployed (seeking work) ☐
- Unemployed (not seeking work) ☐
- Retired ☐
- Student ☐
- Permanently sick/disabled ☐
- Temporarily sick/disabled ☐
- Looking after family/home ☐
- Other, please describe ____

9. What is your highest level of qualification?

Please cross one box only

- Degree (or equivalent) ☐
- Diploma (or equivalent) ☐
- 'A' level ☐
- GCSE / 'O' level ☐
- None ☐
- Other, please specify ____

10. How would you describe your ethnicity

Please cross one box only

White ☐
or
- White British ☐
- White Irish ☐
- Other White background ☐

Mixed ☐
or
- White & Black Carribean ☐
- White & Black African ☐
- White & Asian ☐
- Other mixed background ☐

Black or Black British ☐
or
- Carribean ☐
- African ☐
- Other Black background ☐

Asian or Asian British ☐
or
- Indian ☐
- Pakistani ☐
- Bangladeshi ☐
- Other Asian background ☐

Chinese or other ethnic group ☐
or
- Chinese ☐
- Other ethnic group ☐

11.

Do you live alone?

Yes ☐ **Please go to Question 12**

No ☐ **Please say who you live with below**

Who do you live with?
(e.g. wife, husband, partner, family member)

12. **Are you suffering from, or have you suffered from, any of the following in the past 2 years?**

Please cross any that are applicable to you

- Asthma ☐
- Chronic Obstructive Pulmonary Disease (COPD) ☐
- Other lung disease (e.g. fibrosis, bronchiectatis etc) ☐
- Heart disease ☐
- Anxiety or depression ☐
- Inflammatory bowel disease ☐
- Irritable bowel syndrome ☐
- Peptic ulcer ☐
- Previous cancer ☐
- Diabetes ☐
- Arthritis ☐

13. **About smoking**

Please cross the appropriate statement

- Are you a current smoker? ☐
- Are you an ex-smoker? ☐
- Are you a non-smoker (never smoked)? ☐

14. **Do you think you were more at risk of getting cancer because of your family history?**

Yes ☐

No ☐

15. **The following questions are about how completing the questionnaire made you feel.**

A number of statements which people have used to describe themselves are given below. Read each statement and then cross the most appropriate box to indicate how you feel right now, at the moment. There are no right or wrong answers. Do not spend too much time on any one statement but give the answer which seems to describe your present feeling best.

		Not at all	Somewhat	Moderately	Very much
1.	I feel calm	☐	☐	☐	☐
2.	I am tense	☐	☐	☐	☐
3.	I feel upset	☐	☐	☐	☐
4.	I am relaxed	☐	☐	☐	☐
5.	I feel content	☐	☐	☐	☐
6.	I am worried	☐	☐	☐	☐

Please make sure you have answered all the questions.

If you have any other comments or there is anything else you would like to tell us about when you first noticed your symptoms, please do so using the space below.

If you feel you would like to talk to someone about this questionnaire at a later stage, please speak to the doctor or nurse who is looking after you, or be in touch with the research team, whose contact details are on your patient information sheet

Thank you for completing the questionnaire

Please give it to the research nurse

The next page is for the Researcher to complete

57727

Researcher - Post-completion Sheet

Researcher initials: ☐☐☐

Date questionnaire completed: Date ☐☐ / ☐☐ / ☐☐ Time ☐☐:☐☐

If not completed, give reason ☐

How long is it since the patient was told of his/her diagnosis? ☐

How long did it take the patient to complete the questionnaire?

- Less than 5 minutes ☐
- Between 5 and 10 minutes ☐
- Between 11 and 15 minutes ☐
- Longer than 15 minutes ☐

For the patient selected to <u>complete the questionnaire on their own</u>

Did the patient ask for any help? ☐ Yes ☐ No

If yes,

please specify what help was requested

____

____

please specify what help was given

____

____

For the patient selected for <u>the researcher-administered questionnaire</u>

Did the patient find any of the questions difficult? ☐ Yes ☐ No

If yes,

please specify which questions and what the difficulty was

____

____

____

____

Was the patient made anxious? ☐ Yes ☐ No

If yes,

please give details

____

____

____

____

Where did the patient complete the questionnaire?
(Please specify whether this was in a separate room alone, in a corner of the clinic, the ward or other

____

Thank you. Please now send the questionnaire and patient information sheet and consent form back to Cardiff University in the freepost envelope provided.

57727

PID - | | 1 | 5 |

Ymddiriedolaeth GIG Gogledd Orllewin Cymru
North West Wales NHS Trust

NHS WALES GIG CYMRU
Ymddiriedolaeth GIG Gogledd Cymru
North Wales NHS Trust

☐ GLAN CLWYD

CID - ☐ GWYNEDD

☐ MAELOR

Confidential Questionnaire

Looking At Your Symptoms Study

This Study has been funded by Cancer Research UK

Cardiff University Prifysgol Caerdydd

Ysgol Glinigol Gogledd Cymru
North Wales Clinical School

Cancer Research UK

sewtu South East Wales Trials Unit
Uned Ymchwil De-ddwyrain Cymru

Dear Patient

I am very grateful to you for agreeing to help us with this study.

I hope the information you supply will help us give people a diagnosis earlier.

If you feel you would like to talk to someone about this questionnaire at a later stage, please speak to the doctor or nurse who is looking after you, or be in touch with the research team, whose contact details are on your patient information sheet.

I can assure you that anything you tell us will be treated in the utmost confidence.

Yours sincerely

Dr Richard Neal
Senior Clinical Lecturer
Cardiff University
Chief Investigator, Symptoms Study

About your symptoms

These questions are about symptoms you noticed before your diagnosis. We are interested in symptoms which you think are related to your cancer.

Please put a cross in the appropriate box, or write an answer as required.

Looking back...

1. Did you have any symptoms that you feel were due to your cancer before you were diagnosed?

Yes ☐ **Please continue with Question 2**

No ☐ **Please go to Section 2 on Page 5**

2. What was the first symptom that made you think something might be wrong?

☐

Please go to Question 3

The following questions are about when you first noticed a symptom and when you first told your GP or nurse about it.

Please give an exact date if you can. Otherwise give your best estimate. (For example approximately how long ago, the month or season). You wish to refer to your diary or calendar if you have it with you.

Here is an example question:

Decrease in appetite

Did you have this symptom?

Yes ☒ **Please complete A then B or C below**

No ☐ **Please go to Section 2**

A When did you first notice this? d d / m m / y y **OR** Estimate '3 months ago' or 'June'

B When did you first tell your GP or nurse? 2 3 / 1 2 / 0 7 **OR** Estimate

OR

C Put a cross here if you didn't tell your GP or nurse ☐

3. Change in bowel habit

Did you have this symptom?

Yes ☐ **Please complete A then B or C below**

No ☐ **Please go to Question 4**

A When did you <u>first notice</u> this? ☐☐ / ☐☐ / ☐☐ **OR** ☐

B When did you <u>first tell</u> your GP or nurse? ☐☐ / ☐☐ / ☐☐ **OR** ☐

OR

C Put a cross here if you <u>didn't tell</u> your GP or nurse ☐

4. Bleeding from back passage

Did you have this symptom?

Yes ☐ **Please complete A then B or C below**

No ☐ **Please go to Section 2**

A When did you <u>first notice</u> this? ☐☐ / ☐☐ / ☐☐ **OR** ☐

B When did you <u>first tell</u> your GP or nurse? ☐☐ / ☐☐ / ☐☐ **OR** ☐

OR

C Put a cross here if you <u>didn't tell</u> your GP or nurse ☐

Section 2

Please give an <u>exact</u> date if you can. Otherwise give your best <u>estimate</u> (for example approximately how long ago, the month or the season). You may wish to refer to your diary or calendar if you have it with you.

Did you have any of the following?

1. Decrease in appetite

Did you have this?

Yes ☐ **Please complete A then B or C below**

No ☐ **Please go to Question 2**

A When did you first notice this? ☐☐ / ☐☐ / ☐☐ **OR** ☐

B When did you first tell your GP or nurse? ☐☐ / ☐☐ / ☐☐ **OR** ☐

OR

C Put a cross here if you didn't tell your GP or nurse ☐

2. Unexplained weight loss

Did you have this?

Yes ☐ **Please complete A then B or C below**

No ☐ **Please go to Question 3**

A When did you first notice this? ☐☐ / ☐☐ / ☐☐ **OR** ☐

B When did you first tell your GP or nurse? ☐☐ / ☐☐ / ☐☐ **OR** ☐

OR

C Put a cross here if you didn't tell your GP or nurse ☐

3. **Fatigue or tiredness that is unusual for you**

Did you have this?

Yes ☐ **Please complete A then B or C below**

No ☐ **Please go to Question 4**

A When did you <u>first notice</u> this? ☐☐ / ☐☐ / ☐☐ **OR** ☐

B When did you <u>first tell</u> your GP or nurse? ☐☐ / ☐☐ / ☐☐ **OR** ☐

OR

C Put a cross here if you <u>didn't tell</u> your GP or nurse ☐

4. **Feeling different 'in yourself' from usual**

Did you have this?

Yes ☐ **Please complete A then B or C below**

No ☐ **Please go to Question 5**

A When did you <u>first notice</u> this? ☐☐ / ☐☐ / ☐☐ **OR** ☐

B When did you <u>first tell</u> your GP or nurse? ☐☐ / ☐☐ / ☐☐ **OR** ☐

OR

C Put a cross here if you <u>didn't tell</u> your GP or nurse ☐

Please try and describe what the feeling was

If you had any further symptoms which you feel are relevant to your cancer diagnosis, please list them below. If not, please go to Question 7.

Please describe the symptom here and complete A then B or C below

5.

**A**	When did you first notice this?	☐☐ / ☐☐ / ☐☐ **OR** ☐
**B**	When did you first tell your GP or nurse?	☐☐ / ☐☐ / ☐☐ **OR** ☐
	**OR**	
**C**	Put a cross here if you didn't tell your GP or nurse	☐

If you had another symptom you feel is relevant, please write it below, otherwise go to Question 7.

Please describe the symptom here and complete A then B or C below

6.

**A**	When did you first notice this?	☐☐ / ☐☐ / ☐☐ **OR** ☐
**B**	When did you first tell your GP or nurse?	☐☐ / ☐☐ / ☐☐ **OR** ☐
	**OR**	
**C**	Put a cross here if you didn't tell your GP or nurse	☐

If you had further symptoms you would like to tell us about, there is space at the end of the Questionnaire for you to write them.

Did your GP send you for any tests?

Thinking back to the time immediately before you diagnosis, did your GP send you for any of the following tests?

Please cross the appropriate boxes

7. Were you sent for any of these?

	Yes	Not sure	No
Blood test(s)	☐	☐	☐
CT Scan	☐	☐	☐
Ultrasound scan	☐	☐	☐
Barium Enema	☐	☐	☐
Sigmoidoscopy or colonoscopy (Looking at bowel with internal camera)	☐	☐	☐
X-ray	☐	☐	☐

Please go to Question 8

About you

The next questions relate to general information about you. It would help us to know more about the background of the people completing the questionnaire.

8. **Which best describes your employment status?**

Please cross one box only

- Employed full-time ☐
- Employed part-time ☐
- Self employed full-time ☐
- Self employed part-time ☐
- Unemployed (seeking work) ☐
- Unemployed (not seeking work) ☐
- Retired ☐
- Student ☐
- Permanently sick/disabled ☐
- Temporarily sick/disabled ☐
- Looking after family/home ☐

Other, please describe ____________

9. **What is your highest level of qualification?**

Please cross one box only

- Degree (or equivalent) ☐
- Diploma (or equivalent) ☐
- 'A' level ☐
- GCSE / 'O' level ☐
- None ☐

Other, please specify ____________

10. **How would you describe your ethnicity**

Please cross one box only

White ☐
or
- White British ☐
- White Irish ☐
- Other White background ☐

Mixed ☐
or
- White & Black Carribean ☐
- White & Black African ☐
- White & Asian ☐
- Other mixed background ☐

Black or Black British ☐
or
- Carribean ☐
- African ☐
- Other Black background ☐

Asian or Asian British ☐
or
- Indian ☐
- Pakistani ☐
- Bangladeshi ☐
- Other Asian background ☐

Chinese or other ethnic group ☐
or
- Chinese ☐
- Other ethnic group ☐

11.

Do you live alone?

Yes ☐ **Please go to Question 12**

No ☐ **Please say who you live with below**

Who do you live with?
(e.g. wife, husband, partner, family member)

12. **Are you suffering from, or have you suffered from, any of the following in the past 2 years?**

Please cross any that are applicable to you

- Asthma ☐
- Chronic Obstructive Pulmonary Disease (COPD) ☐
- Other lung disease (e.g. fibrosis, bronchiectatis etc) ☐
- Heart disease ☐
- Anxiety or depression ☐
- Inflammatory bowel disease ☐
- Irritable bowel syndrome ☐
- Peptic ulcer ☐
- Previous cancer ☐
- Diabetes ☐
- Arthritis ☐

13. **About smoking**

Please cross the appropriate statement

- Are you a current smoker? ☐
- Are you an ex-smoker? ☐
- Are you a non-smoker (never smoked)? ☐

14. **Do you think you were more at risk of getting cancer because of your family history?**

Yes ☐

No ☐

15. The following questions are about how completing the questionnaire made you feel.

A number of statements which people have used to describe themselves are given below. Read each statement and then cross the most appropriate box to indicate how you feel right now, at the moment. There are no right or wrong answers. Do not spend too much time on any one statement but give the answer which seems to describe your present feeling best.

		Not at all	Somewhat	Moderately	Very much
1.	I feel calm	☐	☐	☐	☐
2.	I am tense	☐	☐	☐	☐
3.	I feel upset	☐	☐	☐	☐
4.	I am relaxed	☐	☐	☐	☐
5.	I feel content	☐	☐	☐	☐
6.	I am worried	☐	☐	☐	☐

Please make sure you have answered all the questions.

If you have any other comments or there is anything else you would like to tell us about when you first noticed your symptoms, please do so using the space below.

If you feel you would like to talk to someone about this questionnaire at a later stage, please speak to the doctor or nurse who is looking after you, or be in touch with the research team, whose contact details are on your patient information sheet

Thank you for completing the questionnaire

Please give it to the research nurse

The next page is for the Researcher to complete

57727

1 5

Researcher - Post-completion Sheet

Researcher initials: ☐☐☐

Date questionnaire completed: Date ☐☐ / ☐☐ / ☐☐ Time ☐☐:☐☐

If not completed, give reason ☐

How long is it since the patient was told of his/her diagnosis? ☐

How long did it take the patient to complete the questionnaire?

- Less than 5 minutes ☐
- Between 5 and 10 minutes ☐
- Between 11 and 15 minutes ☐
- Longer than 15 minutes ☐

For the patient selected to complete the questionnaire on their own

Did the patient ask for any help? ☐ Yes ☐ No

If yes,

please specify what help was requested

____

____

please specify what help was given

____

____

For the patient selected for the researcher-administered questionnaire

Did the patient find any of the questions difficult? ☐ Yes ☐ No

If yes,

please specify which questions and what the difficulty was

____

____

____

____

Was the patient made anxious? ☐ Yes ☐ No

If yes,

please give details

____

____

____

____

Where did the patient complete the questionnaire?
(Please specify whether this was in a separate room alone, in a corner of the clinic, the ward or other

____

Thank you. Please now send the questionnaire and patient information sheet and consent form back to Cardiff University in the freepost envelope provided.

57727

PID - | 1 | 6

Ymddiriedolaeth GIG Gogledd Orllewin Cymru
North West Wales NHS Trust

NHS WALES GIG CYMRU
Ymddiriedolaeth GIG Gogledd Cymru
North Wales NHS Trust

☐ GLAN CLWYD

CID - ☐ GWYNEDD

☐ MAELOR

Confidential Questionnaire

Looking At Your Symptoms Study

This Study has been funded by Cancer Research UK

Cardiff University Prifysgol Caerdydd

Ysgol Glinigol Gogledd Cymru North Wales Clinical School

Cancer Research UK

sewtu South East Wales Trials Unit Uned Ymchwil De-ddwyrain Cymru

Dear Patient

I am very grateful to you for agreeing to help us with this study.

I hope the information you supply will help us give people a diagnosis earlier.

If you feel you would like to talk to someone about this questionnaire at a later stage, please speak to the doctor or nurse who is looking after you, or be in touch with the research team, whose contact details are on your patient information sheet.

I can assure you that anything you tell us will be treated in the utmost confidence.

Yours sincerely

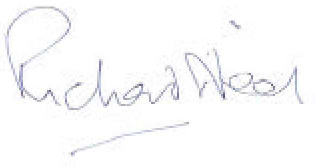

Dr Richard Neal
Senior Clinical Lecturer
Cardiff University
Chief Investigator, Symptoms Study

About your symptoms

These questions are about symptoms you noticed before your diagnosis. We are interested in symptoms which you think are related to your cancer.

Please put a cross in the appropriate box, or write an answer as required.

Looking back...

1. Did you have any symptoms that you feel were due to your cancer before you were diagnosed?

Yes ☐ **Please continue with Question 2**

No ☐ **Please go to Section 2 on Page 5**

2. What was the first symptom that made you think something might be wrong?

[ ]

Please go to Question 3

The following questions are about when you first noticed a symptom and when you first told your GP or nurse about it.

Please give an exact date if you can. Otherwise give your best estimate. (For example approximately how long ago, the month or season). You wish to refer to your diary or calendar if you have it with you.

Here is an example question:

Decrease in appetite

Did you have this symptom?

Yes ☒ **Please complete A then B or C below**

No ☐ **Please go to Section 2**

A When did you first notice this? d d / m m / y y **OR** Estimate: '3 months ago' or 'June'

B When did you first tell your GP or nurse? 2 3 / 1 2 / 0 7 **OR** Estimate

OR

C Put a cross here if you didn't tell your GP or nurse ☐

3. Change in bowel habit

Did you have this symptom?

Yes ☐ **Please complete A then B or C below**

No ☐ **Please go to Question 4**

A When did you first notice this? ☐☐ / ☐☐ / ☐☐ **OR** ☐

B When did you first tell your GP or nurse? ☐☐ / ☐☐ / ☐☐ **OR** ☐

OR

C Put a cross here if you didn't tell your GP or nurse ☐

4. Bleeding from back passage

Did you have this symptom?

Yes ☐ **Please complete A then B or C below**

No ☐ **Please go to Section 2**

A When did you first notice this? ☐☐ / ☐☐ / ☐☐ **OR** ☐

B When did you first tell your GP or nurse? ☐☐ / ☐☐ / ☐☐ **OR** ☐

OR

C Put a cross here if you didn't tell your GP or nurse ☐

Please give an exact date if you can. Otherwise give your best estimate (for example approximately how long ago, the month or the season). You may wish to refer to your diary or calendar if you have it with you.

Did you have any of the following?

1. Decrease in appetite

Did you have this?

Yes ☐ **Please complete A then B or C below**

No ☐ **Please go to Question 2**

A When did you first notice this? ☐☐ / ☐☐ / ☐☐ **OR** ☐

B When did you first tell your GP or nurse? ☐☐ / ☐☐ / ☐☐ **OR** ☐

OR

C Put a cross here if you didn't tell your GP or nurse ☐

2. Unexplained weight loss

Did you have this?

Yes ☐ **Please complete A then B or C below**

No ☐ **Please go to Question 3**

A When did you first notice this? ☐☐ / ☐☐ / ☐☐ **OR** ☐

B When did you first tell your GP or nurse? ☐☐ / ☐☐ / ☐☐ **OR** ☐

OR

C Put a cross here if you didn't tell your GP or nurse ☐

3. **Fatigue or tiredness that is unusual for you**

Did you have this?

Yes ☐ **Please complete A then B or C below**

No ☐ **Please go to Question 4**

A When did you first notice this? ☐☐ / ☐☐ / ☐☐ **OR** ☐

B When did you first tell your GP or nurse? ☐☐ / ☐☐ / ☐☐ **OR** ☐

OR

C Put a cross here if you didn't tell your GP or nurse ☐

4. **Feeling different 'in yourself' from usual**

Did you have this?

Yes ☐ **Please complete A then B or C below**

No ☐ **Please go to Question 5**

A When did you first notice this? ☐☐ / ☐☐ / ☐☐ **OR** ☐

B When did you first tell your GP or nurse? ☐☐ / ☐☐ / ☐☐ **OR** ☐

OR

C Put a cross here if you didn't tell your GP or nurse ☐

Please try and describe what the feeling was

If you had any further symptoms which you feel are relevant to your cancer diagnosis, please list them below. If not, please go to Question 7.

Please describe the symptom here and complete A then B or C below

5.

A When did you first notice this? ☐☐ / ☐☐ / ☐☐ **OR** ☐

B When did you first tell your GP or nurse? ☐☐ / ☐☐ / ☐☐ **OR** ☐

OR

C Put a cross here if you didn't tell your GP or nurse ☐

If you had another symptom you feel is relevant, please write it below, otherwise go to Question 7.

Please describe the symptom here and complete A then B or C below

6.

A When did you first notice this? ☐☐ / ☐☐ / ☐☐ **OR** ☐

B When did you first tell your GP or nurse? ☐☐ / ☐☐ / ☐☐ **OR** ☐

OR

C Put a cross here if you didn't tell your GP or nurse ☐

If you had further symptoms you would like to tell us about, there is space at the end of the Questionnaire for you to write them.

Did your GP send you for any tests?

Thinking back to the time immediately before you diagnosis, did your GP send you for any of the following tests?

Please cross the appropriate boxes

7. Were you sent for any of these?

	Yes	Not sure	No
Blood test(s)	☐	☐	☐
CT Scan	☐	☐	☐
Ultrasound scan	☐	☐	☐
Barium Enema	☐	☐	☐
Sigmoidoscopy or colonoscopy (Looking at bowel with internal camera)	☐	☐	☐
X-ray	☐	☐	☐

Please go to Question 8

About you

The next questions relate to general information about you. It would help us to know more about the background of the people completing the questionnaire.

8. Which best describes your employment status?

Please cross one box only

- Employed full-time ☐
- Employed part-time ☐
- Self employed full-time ☐
- Self employed part-time ☐
- Unemployed (seeking work) ☐
- Unemployed (not seeking work) ☐
- Retired ☐
- Student ☐
- Permanently sick/disabled ☐
- Temporarily sick/disabled ☐
- Looking after family/home ☐
- Other, please describe ______

9. What is your highest level of qualification?

Please cross one box only

- Degree (or equivalent) ☐
- Diploma (or equivalent) ☐
- 'A' level ☐
- GCSE / 'O' level ☐
- None ☐
- Other, please specify ______

10. How would you describe your ethnicity

Please cross one box only

White ☐
or
- White British ☐
- White Irish ☐
- Other White background ☐

Mixed ☐
or
- White & Black Carribean ☐
- White & Black African ☐
- White & Asian ☐
- Other mixed background ☐

Black or Black British ☐
or
- Carribean ☐
- African ☐
- Other Black background ☐

Asian or Asian British ☐
or
- Indian ☐
- Pakistani ☐
- Bangladeshi ☐
- Other Asian background ☐

Chinese or other ethnic group ☐
or
- Chinese ☐
- Other ethnic group ☐

11.

Do you live alone?

Yes ☐ **Please go to Question 12**

No ☐ **Please say who you live with below**

Who do you live with?
(e.g. wife, husband, partner, family member)

12. **Are you suffering from, or have you suffered from, any of the following in the past 2 years?**

Please cross any that are applicable to you

- Asthma ☐
- Chronic Obstructive Pulmonary Disease (COPD) ☐
- Other lung disease (e.g. fibrosis, bronchiectatis etc) ☐
- Heart disease ☐
- Anxiety or depression ☐
- Inflammatory bowel disease ☐
- Irritable bowel syndrome ☐
- Peptic ulcer ☐
- Previous cancer ☐
- Diabetes ☐
- Arthritis ☐

13. **About smoking**

Please cross the appropriate statement

- Are you a current smoker? ☐
- Are you an ex-smoker? ☐
- Are you a non-smoker (never smoked)? ☐

14. **Do you think you were more at risk of getting cancer because of your family history?**

Yes ☐

No ☐

15. **The following questions are about how completing the questionnaire made you feel.**

A number of statements which people have used to describe themselves are given below. Read each statement and then cross the most appropriate box to indicate how you feel <u>right now, at the moment.</u> There are no right or wrong answers. Do not spend too much time on any one statement but give the answer which seems to describe your present feeling best.

		Not at all	Somewhat	Moderately	Very much
1.	I feel calm	☐	☐	☐	☐
2.	I am tense	☐	☐	☐	☐
3.	I feel upset	☐	☐	☐	☐
4.	I am relaxed	☐	☐	☐	☐
5.	I feel content	☐	☐	☐	☐
6.	I am worried	☐	☐	☐	☐

Please make sure you have answered all the questions.

If you have any other comments or there is anything else you would like to tell us about when you first noticed your symptoms, please do so using the space below.

If you feel you would like to talk to someone about this questionnaire at a later stage, please speak to the doctor or nurse who is looking after you, or be in touch with the research team, whose contact details are on your patient information sheet

Thank you for completing the questionnaire

Please give it to the research nurse

The next page is for the Researcher to complete

57727

1 6

Researcher - Post-completion Sheet

Researcher initials: ☐☐☐

Date questionnaire completed: Date ☐☐ / ☐☐ / ☐☐ Time ☐☐:☐☐

If not completed, give reason ☐

How long is it since the patient was told of his/her diagnosis? ☐

How long did it take the patient to complete the questionnaire?

- Less than 5 minutes ☐
- Between 5 and 10 minutes ☐
- Between 11 and 15 minutes ☐
- Longer than 15 minutes ☐

For the patient selected to complete the questionnaire on their own

Did the patient ask for any help? ☐ Yes ☐ No

If yes,

please specify what help was requested

____

____

please specify what help was given

____

____

For the patient selected for the researcher-administered questionnaire

Did the patient find any of the questions difficult? ☐ Yes ☐ No

If yes,

please specify which questions and what the difficulty was

____

____

____

____

Was the patient made anxious? ☐ Yes ☐ No

If yes,

please give details

____

____

____

____

Where did the patient complete the questionnaire?

(Please specify whether this was in a separate room alone, in a corner of the clinic, the ward or other

____

Thank you. Please now send the questionnaire and patient information sheet and consent form back to Cardiff University in the freepost envelope provided.

57727

PID - 1 7

Ymddiriedolaeth GIG Gogledd Orllewin Cymru
North West Wales NHS Trust

NHS WALES GIG CYMRU
Ymddiriedolaeth GIG Gogledd Cymru
North Wales NHS Trust

☐ GLAN CLWYD

CID - ☐ GWYNEDD

☐ MAELOR

Confidential Questionnaire

Looking At Your Symptoms Study

This Study has been funded by Cancer Research UK

Cardiff University / Prifysgol Caerdydd — Ysgol Glinigol Gogledd Cymru / North Wales Clinical School — Cancer Research UK — sewtu South East Wales Trials Unit / Uned Ymchwil De-ddwyrain Cymru

Dear Patient

I am very grateful to you for agreeing to help us with this study.

I hope the information you supply will help us give people a diagnosis earlier.

If you feel you would like to talk to someone about this questionnaire at a later stage, please speak to the doctor or nurse who is looking after you, or be in touch with the research team, whose contact details are on your patient information sheet.

I can assure you that anything you tell us will be treated in the utmost confidence.

Yours sincerely

Dr Richard Neal
Senior Clinical Lecturer
Cardiff University
Chief Investigator, Symptoms Study

About your symptoms

These questions are about symptoms you noticed before your diagnosis. We are interested in symptoms which you think are related to your cancer.

Please put a cross in the appropriate box, or write an answer as required.

Looking back...

1. Did you have any symptoms that you feel were due to your cancer before you were diagnosed?

Yes ☐ **Please continue with Question 2**

No ☐ **Please go to Section 2 on Page 5**

2. What was the first symptom that made you think something might be wrong?

☐

Please go to Question 3

The following questions are about when you first noticed a symptom and when you first told your GP or nurse about it.

Please give an exact date if you can. Otherwise give your best estimate. (For example approximately how long ago, the month or season). You wish to refer to your diary or calendar if you have it with you.

Here is an example question:

Decrease in appetite

Did you have this symptom?

Yes ☒ **Please complete A then B or C below**

No ☐ **Please go to Section 2**

A When did you first notice this? d d / m m / y y **OR** Estimate '3 months ago' or 'June'

B When did you first tell your GP or nurse? 2 3 / 1 2 / 0 7 **OR** Estimate

OR

C Put a cross here if you didn't tell your GP or nurse ☐

3. Change in bowel habit

Did you have this symptom?

Yes ☐ **Please complete A then B or C below**

No ☐ **Please go to Question 4**

A When did you first notice this? ☐☐ / ☐☐ / ☐☐ **OR** ☐

B When did you first tell your GP or nurse? ☐☐ / ☐☐ / ☐☐ **OR** ☐

OR

C Put a cross here if you didn't tell your GP or nurse ☐

4. Bleeding from back passage

Did you have this symptom?

Yes ☐ **Please complete A then B or C below**

No ☐ **Please go to Section 2**

A When did you first notice this? ☐☐ / ☐☐ / ☐☐ **OR** ☐

B When did you first tell your GP or nurse? ☐☐ / ☐☐ / ☐☐ **OR** ☐

OR

C Put a cross here if you didn't tell your GP or nurse ☐

Section 2

Please give an exact date if you can. Otherwise give your best estimate (for example approximately how long ago, the month or the season). You may wish to refer to your diary or calendar if you have it with you.

Did you have any of the following?

1. Decrease in appetite

Did you have this?

Yes ☐ **Please complete A then B or C below**

No ☐ **Please go to Question 2**

A When did you first notice this? ☐☐ / ☐☐ / ☐☐ **OR** ☐

B When did you first tell your GP or nurse? ☐☐ / ☐☐ / ☐☐ **OR** ☐

OR

C Put a cross here if you didn't tell your GP or nurse ☐

2. Unexplained weight loss

Did you have this?

Yes ☐ **Please complete A then B or C below**

No ☐ **Please go to Question 3**

A When did you first notice this? ☐☐ / ☐☐ / ☐☐ **OR** ☐

B When did you first tell your GP or nurse? ☐☐ / ☐☐ / ☐☐ **OR** ☐

OR

C Put a cross here if you didn't tell your GP or nurse ☐

3. **Fatigue or tiredness that is unusual for you**

Did you have this?

Yes ☐ **Please complete A then B or C below**

No ☐ **Please go to Question 4**

A When did you first notice this? ☐☐ / ☐☐ / ☐☐ **OR** ☐

B When did you first tell your GP or nurse? ☐☐ / ☐☐ / ☐☐ **OR** ☐

OR

C Put a cross here if you didn't tell your GP or nurse ☐

4. **Feeling different 'in yourself' from usual**

Did you have this?

Yes ☐ **Please complete A then B or C below**

No ☐ **Please go to Question 5**

A When did you first notice this? ☐☐ / ☐☐ / ☐☐ **OR** ☐

B When did you first tell your GP or nurse? ☐☐ / ☐☐ / ☐☐ **OR** ☐

OR

C Put a cross here if you didn't tell your GP or nurse ☐

Please try and describe what the feeling was

If you had any further symptoms which you feel are relevant to your cancer diagnosis, please list them below. If not, please go to Question 7.

Please describe the symptom here and complete A then B or C below

5.

**A**	When did you first notice this?	__ __ / __ __ / __ __ **OR**
**B**	When did you first tell your GP or nurse?	__ __ / __ __ / __ __ **OR**
	**OR**	
**C**	Put a cross here if you didn't tell your GP or nurse	☐

If you had another symptom you feel is relevant, please write it below, otherwise go to Question 7.

Please describe the symptom here and complete A then B or C below

6.

**A**	When did you first notice this?	__ __ / __ __ / __ __ **OR**
**B**	When did you first tell your GP or nurse?	__ __ / __ __ / __ __ **OR**
	**OR**	
**C**	Put a cross here if you didn't tell your GP or nurse	☐

If you had further symptoms you would like to tell us about, there is space at the end of the Questionnaire for you to write them.

Did your GP send you for any tests?

Thinking back to the time immediately before you diagnosis, did your GP send you for any of the following tests?

Please cross the appropriate boxes

7. Were you sent for any of these?

	Yes	Not sure	No
Blood test(s)	☐	☐	☐
CT Scan	☐	☐	☐
Ultrasound scan	☐	☐	☐
Barium Enema	☐	☐	☐
Sigmoidoscopy or colonoscopy (Looking at bowel with internal camera)	☐	☐	☐
X-ray	☐	☐	☐

Please go to Question 8

About you

The next questions relate to general information about you. It would help us to know more about the background of the people completing the questionnaire.

8. Which best describes your employment status?

Please cross one box only

- Employed full-time ☐
- Employed part-time ☐
- Self employed full-time ☐
- Self employed part-time ☐
- Unemployed (seeking work) ☐
- Unemployed (not seeking work) ☐
- Retired ☐
- Student ☐
- Permanently sick/disabled ☐
- Temporarily sick/disabled ☐
- Looking after family/home ☐
- Other, please describe ____

9. What is your highest level of qualification?

Please cross one box only

- Degree (or equivalent) ☐
- Diploma (or equivalent) ☐
- 'A' level ☐
- GCSE / 'O' level ☐
- None ☐
- Other, please specify ____

10. **How would you describe your ethnicity**

Please cross one box only

White ☐
or
- White British ☐
- White Irish ☐
- Other White background ☐

Mixed ☐
or
- White & Black Carribean ☐
- White & Black African ☐
- White & Asian ☐
- Other mixed background ☐

Black or Black British ☐
or
- Carribean ☐
- African ☐
- Other Black background ☐

Asian or Asian British ☐
or
- Indian ☐
- Pakistani ☐
- Bangladeshi ☐
- Other Asian background ☐

Chinese or other ethnic group ☐
or
- Chinese ☐
- Other ethnic group ☐

11.

Do you live alone?

Yes ☐ **Please go to Question 12**

No ☐ **Please say who you live with below**

Who do you live with?
(e.g. wife, husband, partner, family member)

12. Are you suffering from, or have you suffered from, any of the following in the past 2 years?

Please cross any that are applicable to you

- Asthma ☐
- Chronic Obstructive Pulmonary Disease (COPD) ☐
- Other lung disease (e.g. fibrosis, bronchiectatis etc) ☐
- Heart disease ☐
- Anxiety or depression ☐
- Inflammatory bowel disease ☐
- Irritable bowel syndrome ☐
- Peptic ulcer ☐
- Previous cancer ☐
- Diabetes ☐
- Arthritis ☐

13. About smoking

Please cross the appropriate statement

- Are you a current smoker? ☐
- Are you an ex-smoker? ☐
- Are you a non-smoker (never smoked)? ☐

14. Do you think you were more at risk of getting cancer because of your family history?

Yes ☐
No ☐

15. The following questions are about how completing the questionnaire made you feel.

A number of statements which people have used to describe themselves are given below. Read each statement and then cross the most appropriate box to indicate how you feel right now, at the moment. There are no right or wrong answers. Do not spend too much time on any one statement but give the answer which seems to describe your present feeling best.

		Not at all	Somewhat	Moderately	Very much
1.	I feel calm	☐	☐	☐	☐
2.	I am tense	☐	☐	☐	☐
3.	I feel upset	☐	☐	☐	☐
4.	I am relaxed	☐	☐	☐	☐
5.	I feel content	☐	☐	☐	☐
6.	I am worried	☐	☐	☐	☐

Please make sure you have answered all the questions.

If you have any other comments or there is anything else you would like to tell us about when you first noticed your symptoms, please do so using the space below.

If you feel you would like to talk to someone about this questionnaire at a later stage, please speak to the doctor or nurse who is looking after you, or be in touch with the research team, whose contact details are on your patient information sheet

Thank you for completing the questionnaire

Please give it to the research nurse

The next page is for the Researcher to complete

Researcher - Post-completion Sheet

Researcher initials: ☐☐☐

Date questionnaire completed: Date ☐☐ / ☐☐ / ☐☐ Time ☐☐:☐☐

If not completed, give reason ☐

How long is it since the patient was told of his/her diagnosis? ☐

How long did it take the patient to complete the questionnaire?

- Less than 5 minutes ☐
- Between 5 and 10 minutes ☐
- Between 11 and 15 minutes ☐
- Longer than 15 minutes ☐

For the patient selected to <u>complete the questionnaire on their own</u>

Did the patient ask for any help? ☐ Yes ☐ No

If yes,

please specify what help was requested

____

____

please specify what help was given

____

____

For the patient selected for <u>the researcher-administered questionnaire</u>

Did the patient find any of the questions difficult? ☐ Yes ☐ No

If yes,

please specify which questions and what the difficulty was

____

____

____

____

Was the patient made anxious? ☐ Yes ☐ No

If yes,

please give details

____

____

____

____

Where did the patient complete the questionnaire?
(Please specify whether this was in a separate room alone, in a corner of the clinic, the ward or other

____

Thank you. Please now send the questionnaire and patient information sheet and consent form back to Cardiff University in the freepost envelope provided.

57727

PID - | 1 | 8 |

Ymddiriedolaeth GIG Gogledd Orllewin Cymru
North West Wales NHS Trust

NHS WALES GIG CYMRU
Ymddiriedolaeth GIG Gogledd Cymru
North Wales NHS Trust

CID -
☐ GLAN CLWYD
☐ GWYNEDD
☐ MAELOR

Confidential Questionnaire

Looking At Your Symptoms Study

This Study has been funded by Cancer Research UK

Cardiff University Prifysgol Caerdydd
Ysgol Glinigol Gogledd Cymru North Wales Clinical School
Cancer Research UK
sewtu South East Wales Trials Unit Uned Ymchwil De-ddwyrain Cymru

Dear Patient

I am very grateful to you for agreeing to help us with this study.

I hope the information you supply will help us give people a diagnosis earlier.

If you feel you would like to talk to someone about this questionnaire at a later stage, please speak to the doctor or nurse who is looking after you, or be in touch with the research team, whose contact details are on your patient information sheet.

I can assure you that anything you tell us will be treated in the utmost confidence.

Yours sincerely

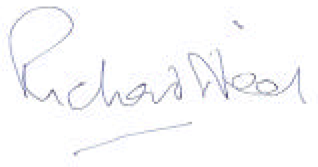

Dr Richard Neal
Senior Clinical Lecturer
Cardiff University
Chief Investigator, Symptoms Study

About your symptoms

These questions are about symptoms you noticed before your diagnosis. We are interested in symptoms which you think are related to your cancer.

Please put a cross in the appropriate box, or write an answer as required.

Looking back...

1. Did you have any symptoms that you feel were due to your cancer before you were diagnosed?

Yes ☐ **Please continue with Question 2**

No ☐ **Please go to Section 2 on Page 5**

2. What was the first symptom that made you think something might be wrong?

☐

Please go to Question 3

The following questions are about when you first noticed a symptom and when you first told your GP or nurse about it.

Please give an exact date if you can. Otherwise give your best estimate. (For example approximately how long ago, the month or season). You wish to refer to your diary or calendar if you have it with you.

Here is an example question:

Decrease in appetite

Did you have this symptom?

Yes ☒ **Please complete A then B or C below**

No ☐ **Please go to Section 2**

A When did you first notice this? d d / m m / y y **OR** Estimate: '3 months ago' or 'June'

B When did you first tell your GP or nurse? 2 3 / 1 2 / 0 7 **OR** Estimate

OR

C Put a cross here if you didn't tell your GP or nurse ☐

3. Change in bowel habit

Did you have this symptom?

Yes ☐ **Please complete A then B or C below**

No ☐ **Please go to Question 4**

A When did you <u>first notice</u> this? ☐☐ / ☐☐ / ☐☐ **OR** ☐

B When did you <u>first tell</u> your GP or nurse? ☐☐ / ☐☐ / ☐☐ **OR** ☐

OR

C Put a cross here if you <u>didn't tell</u> your GP or nurse ☐

4. Bleeding from back passage

Did you have this symptom?

Yes ☐ **Please complete A then B or C below**

No ☐ **Please go to Section 2**

A When did you <u>first notice</u> this? ☐☐ / ☐☐ / ☐☐ **OR** ☐

B When did you <u>first tell</u> your GP or nurse? ☐☐ / ☐☐ / ☐☐ **OR** ☐

OR

C Put a cross here if you <u>didn't tell</u> your GP or nurse ☐

Please give an exact date if you can. Otherwise give your best estimate (for example approximately how long ago, the month or the season). You may wish to refer to your diary or calendar if you have it with you.

Did you have any of the following?

1. Decrease in appetite

Did you have this?

Yes ☐ **Please complete A then B or C below**

No ☐ **Please go to Question 2**

A When did you first notice this? ☐☐ / ☐☐ / ☐☐ **OR** ☐

B When did you first tell your GP or nurse? ☐☐ / ☐☐ / ☐☐ **OR** ☐

OR

C Put a cross here if you didn't tell your GP or nurse ☐

2. Unexplained weight loss

Did you have this?

Yes ☐ **Please complete A then B or C below**

No ☐ **Please go to Question 3**

A When did you first notice this? ☐☐ / ☐☐ / ☐☐ **OR** ☐

B When did you first tell your GP or nurse? ☐☐ / ☐☐ / ☐☐ **OR** ☐

OR

C Put a cross here if you didn't tell your GP or nurse ☐

3. **Fatigue or tiredness that is unusual for you**

Did you have this?

Yes ☐ **Please complete A then B or C below**

No ☐ **Please go to Question 4**

A When did you first notice this? ☐☐ / ☐☐ / ☐☐ **OR** ☐

B When did you first tell your GP or nurse? ☐☐ / ☐☐ / ☐☐ **OR** ☐

OR

C Put a cross here if you didn't tell your GP or nurse ☐

4. **Feeling different 'in yourself' from usual**

Did you have this?

Yes ☐ **Please complete A then B or C below**

No ☐ **Please go to Question 5**

A When did you first notice this? ☐☐ / ☐☐ / ☐☐ **OR** ☐

B When did you first tell your GP or nurse? ☐☐ / ☐☐ / ☐☐ **OR** ☐

OR

C Put a cross here if you didn't tell your GP or nurse ☐

Please try and describe what the feeling was

If you had any further symptoms which you feel are relevant to your cancer diagnosis, please list them below. If not, please go to Question 7.

Please describe the symptom here and complete A then B or C below

5.

**A**	When did you first notice this?	__ __ / __ __ / __ __ **OR**
**B**	When did you first tell your GP or nurse?	__ __ / __ __ / __ __ **OR**
	**OR**	
**C**	Put a cross here if you didn't tell your GP or nurse	☐

If you had another symptom you feel is relevant, please write it below, otherwise go to Question 7.

Please describe the symptom here and complete A then B or C below

6.

**A**	When did you first notice this?	__ __ / __ __ / __ __ **OR**
**B**	When did you first tell your GP or nurse?	__ __ / __ __ / __ __ **OR**
	**OR**	
**C**	Put a cross here if you didn't tell your GP or nurse	☐

If you had further symptoms you would like to tell us about, there is space at the end of the Questionnaire for you to write them.

Did your GP send you for any tests?

Thinking back to the time immediately before you diagnosis, did your GP send you for any of the following tests?

Please cross the appropriate boxes

7. Were you sent for any of these?

	Yes	Not sure	No
Blood test(s)	☐	☐	☐
CT Scan	☐	☐	☐
Ultrasound scan	☐	☐	☐
Barium Enema	☐	☐	☐
Sigmoidoscopy or colonoscopy (Looking at bowel with internal camera)	☐	☐	☐
X-ray	☐	☐	☐

Please go to Question 8

About you

The next questions relate to general information about you. It would help us to know more about the background of the people completing the questionnaire.

8. Which best describes your employment status?

Please cross one box only

- Employed full-time ☐
- Employed part-time ☐
- Self employed full-time ☐
- Self employed part-time ☐
- Unemployed (seeking work) ☐
- Unemployed (not seeking work) ☐
- Retired ☐
- Student ☐
- Permanently sick/disabled ☐
- Temporarily sick/disabled ☐
- Looking after family/home ☐
- Other, please describe

9. What is your highest level of qualification?

Please cross one box only

- Degree (or equivalent) ☐
- Diploma (or equivalent) ☐
- 'A' level ☐
- GCSE / 'O' level ☐
- None ☐
- Other, please specify

10. How would you describe your ethnicity

Please cross one box only

White ☐
or
- White British ☐
- White Irish ☐
- Other White background ☐

Mixed ☐
or
- White & Black Carribean ☐
- White & Black African ☐
- White & Asian ☐
- Other mixed background ☐

Black or Black British ☐
or
- Carribean ☐
- African ☐
- Other Black background ☐

Asian or Asian British ☐
or
- Indian ☐
- Pakistani ☐
- Bangladeshi ☐
- Other Asian background ☐

Chinese or other ethnic group ☐
or
- Chinese ☐
- Other ethnic group ☐

11.

Do you live alone?

Yes ☐ **Please go to Question 12**

No ☐ **Please say who you live with below**

Who do you live with?
(e.g. wife, husband, partner, family member)

12. **Are you suffering from, or have you suffered from, any of the following in the past 2 years?**

Please cross any that are applicable to you

- Asthma ☐
- Chronic Obstructive Pulmonary Disease (COPD) ☐
- Other lung disease (e.g. fibrosis, bronchiectatis etc) ☐
- Heart disease ☐
- Anxiety or depression ☐
- Inflammatory bowel disease ☐
- Irritable bowel syndrome ☐
- Peptic ulcer ☐
- Previous cancer ☐
- Diabetes ☐
- Arthritis ☐

13. **About smoking**

Please cross the appropriate statement

- Are you a current smoker? ☐
- Are you an ex-smoker? ☐
- Are you a non-smoker (never smoked)? ☐

14. **Do you think you were more at risk of getting cancer because of your family history?**

Yes ☐
No ☐

15. **The following questions are about how completing the questionnaire made you feel.**

A number of statements which people have used to describe themselves are given below. Read each statement and then cross the most appropriate box to indicate how you feel right now, at the moment. There are no right or wrong answers. Do not spend too much time on any one statement but give the answer which seems to describe your present feeling best.

		Not at all	Somewhat	Moderately	Very much
1.	I feel calm	☐	☐	☐	☐
2.	I am tense	☐	☐	☐	☐
3.	I feel upset	☐	☐	☐	☐
4.	I am relaxed	☐	☐	☐	☐
5.	I feel content	☐	☐	☐	☐
6.	I am worried	☐	☐	☐	☐

Please make sure you have answered all the questions.

If you have any other comments or there is anything else you would like to tell us about when you first noticed your symptoms, please do so using the space below.

If you feel you would like to talk to someone about this questionnaire at a later stage, please speak to the doctor or nurse who is looking after you, or be in touch with the research team, whose contact details are on your patient information sheet

Thank you for completing the questionnaire

Please give it to the research nurse

The next page is for the Researcher to complete

57727

1 8

Researcher - Post-completion Sheet

Researcher initials: ☐☐☐

Date questionnaire completed: Date ☐☐ / ☐☐ / ☐☐ Time ☐☐:☐☐

If not completed, give reason ☐

How long is it since the patient was told of his/her diagnosis? ☐

How long did it take the patient to complete the questionnaire?

- Less than 5 minutes ☐
- Between 5 and 10 minutes ☐
- Between 11 and 15 minutes ☐
- Longer than 15 minutes ☐

For the patient selected to <u>complete the questionnaire on their own</u>

Did the patient ask for any help? ☐ Yes ☐ No

If yes,

please specify what help was requested

____

____

please specify what help was given

____

____

For the patient selected for <u>the researcher-administered questionnaire</u>

Did the patient find any of the questions difficult? ☐ Yes ☐ No

If yes,

please specify which questions and what the difficulty was

____

____

____

____

Was the patient made anxious? ☐ Yes ☐ No

If yes,

please give details

____

____

____

____

Where did the patient complete the questionnaire?
(Please specify whether this was in a separate room alone, in a corner of the clinic, the ward or other

____

Thank you. Please now send the questionnaire and patient information sheet and consent form back to Cardiff University in the freepost envelope provided.

57727

PID - | 1 | 9

Ymddiriedolaeth GIG Gogledd Orllewin Cymru
North West Wales NHS Trust

NHS WALES GIG CYMRU
Ymddiriedolaeth GIG Gogledd Cymru
North Wales NHS Trust

CID -
☐ GLAN CLWYD
☐ GWYNEDD
☐ MAELOR

Confidential Questionnaire

Looking At Your Symptoms Study

This Study has been funded by Cancer Research UK

Cardiff University / Prifysgol Caerdydd
Ysgol Glinigol Gogledd Cymru / North Wales Clinical School
Cancer Research UK
sewtu — South East Wales Trials Unit / Uned Ymchwil De-ddwyrain Cymru

Dear Patient

I am very grateful to you for agreeing to help us with this study.

I hope the information you supply will help us give people a diagnosis earlier.

If you feel you would like to talk to someone about this questionnaire at a later stage, please speak to the doctor or nurse who is looking after you, or be in touch with the research team, whose contact details are on your patient information sheet.

I can assure you that anything you tell us will be treated in the utmost confidence.

Yours sincerely

Richard Neal

Dr Richard Neal
Senior Clinical Lecturer
Cardiff University
Chief Investigator, Symptoms Study

About your symptoms

These questions are about symptoms you noticed before your diagnosis. We are interested in symptoms which you think are related to your cancer.

Please put a cross in the appropriate box, or write an answer as required.

Looking back...

1. Did you have any symptoms that you feel were due to your cancer before you were diagnosed?

Yes ☐ **Please continue with Question 2**

No ☐ **Please go to Section 2 on Page 5**

2. What was the first symptom that made you think something might be wrong?

☐

Please go to Question 3

The following questions are about when you first noticed a symptom and when you first told your GP or nurse about it.

Please give an exact date if you can. Otherwise give your best estimate. (For example approximately how long ago, the month or season). You wish to refer to your diary or calendar if you have it with you.

Here is an example question:

Decrease in appetite

Did you have this symptom?

Yes ☒ **Please complete A then B or C below**

No ☐ **Please go to Section 2**

A When did you first notice this? d d / m m / y y **OR** Estimate '3 months ago' or 'June'

B When did you first tell your GP or nurse? 2 3 / 1 2 / 0 7 **OR** Estimate

OR

C Put a cross here if you didn't tell your GP or nurse ☐

3. Change in bowel habit

Did you have this symptom?

Yes ☐ **Please complete A then B or C below**

No ☐ **Please go to Question 4**

A When did you first notice this? ☐☐ / ☐☐ / ☐☐ **OR** ☐

B When did you first tell your GP or nurse? ☐☐ / ☐☐ / ☐☐ **OR** ☐

OR

C Put a cross here if you didn't tell your GP or nurse ☐

4. Bleeding from back passage

Did you have this symptom?

Yes ☐ **Please complete A then B or C below**

No ☐ **Please go to Section 2**

A When did you first notice this? ☐☐ / ☐☐ / ☐☐ **OR** ☐

B When did you first tell your GP or nurse? ☐☐ / ☐☐ / ☐☐ **OR** ☐

OR

C Put a cross here if you didn't tell your GP or nurse ☐

Please give an exact date if you can. Otherwise give your best estimate (for example approximately how long ago, the month or the season). You may wish to refer to your diary or calendar if you have it with you.

Did you have any of the following?

1. Decrease in appetite

Did you have this?

Yes ☐ **Please complete A then B or C below**

No ☐ **Please go to Question 2**

A When did you first notice this? ☐☐ / ☐☐ / ☐☐ **OR** ☐

B When did you first tell your GP or nurse? ☐☐ / ☐☐ / ☐☐ **OR** ☐

OR

C Put a cross here if you didn't tell your GP or nurse ☐

2. Unexplained weight loss

Did you have this?

Yes ☐ **Please complete A then B or C below**

No ☐ **Please go to Question 3**

A When did you first notice this? ☐☐ / ☐☐ / ☐☐ **OR** ☐

B When did you first tell your GP or nurse? ☐☐ / ☐☐ / ☐☐ **OR** ☐

OR

C Put a cross here if you didn't tell your GP or nurse ☐

3. **Fatigue or tiredness that is unusual for you**

Did you have this?

Yes ☐ **Please complete A then B or C below**

No ☐ **Please go to Question 4**

A When did you first notice this? ☐☐ / ☐☐ / ☐☐ **OR** ☐

B When did you first tell your GP or nurse? ☐☐ / ☐☐ / ☐☐ **OR** ☐

OR

C Put a cross here if you didn't tell your GP or nurse ☐

4. **Feeling different 'in yourself' from usual**

Did you have this?

Yes ☐ **Please complete A then B or C below**

No ☐ **Please go to Question 5**

A When did you first notice this? ☐☐ / ☐☐ / ☐☐ **OR** ☐

B When did you first tell your GP or nurse? ☐☐ / ☐☐ / ☐☐ **OR** ☐

OR

C Put a cross here if you didn't tell your GP or nurse ☐

Please try and describe what the feeling was

If you had any further symptoms which you feel are relevant to your cancer diagnosis, please list them below. If not, please go to Question 7.

Please describe the symptom here and complete A then B or C below

5.

**A**	When did you first notice this?	☐☐ / ☐☐ / ☐☐ **OR** ☐
**B**	When did you first tell your GP or nurse?	☐☐ / ☐☐ / ☐☐ **OR** ☐
	**OR**	
**C**	Put a cross here if you didn't tell your GP or nurse	☐

If you had another symptom you feel is relevant, please write it below, otherwise go to Question 7.

Please describe the symptom here and complete A then B or C below

6.

**A**	When did you first notice this?	☐☐ / ☐☐ / ☐☐ **OR** ☐
**B**	When did you first tell your GP or nurse?	☐☐ / ☐☐ / ☐☐ **OR** ☐
	**OR**	
**C**	Put a cross here if you didn't tell your GP or nurse	☐

If you had further symptoms you would like to tell us about, there is space at the end of the Questionnaire for you to write them.

Did your GP send you for any tests?

Thinking back to the time immediately before you diagnosis, did your GP send you for any of the following tests?

Please cross the appropriate boxes

7. Were you sent for any of these?

	Yes	Not sure	No
Blood test(s)	☐	☐	☐
CT Scan	☐	☐	☐
Ultrasound scan	☐	☐	☐
Barium Enema	☐	☐	☐
Sigmoidoscopy or colonoscopy (Looking at bowel with internal camera)	☐	☐	☐
X-ray	☐	☐	☐

Please go to Question 8

About you

The next questions relate to general information about you. It would help us to know more about the background of the people completing the questionnaire.

8. Which best describes your employment status?

Please cross one box only

- Employed full-time ☐
- Employed part-time ☐
- Self employed full-time ☐
- Self employed part-time ☐
- Unemployed (seeking work) ☐
- Unemployed (not seeking work) ☐
- Retired ☐
- Student ☐
- Permanently sick/disabled ☐
- Temporarily sick/disabled ☐
- Looking after family/home ☐
- Other, please describe ______

9. What is your highest level of qualification?

Please cross one box only

- Degree (or equivalent) ☐
- Diploma (or equivalent) ☐
- 'A' level ☐
- GCSE / 'O' level ☐
- None ☐
- Other, please specify ______

10. How would you describe your ethnicity

Please cross one box only

White ☐
or
- White British ☐
- White Irish ☐
- Other White background ☐

Mixed ☐
or
- White & Black Carribean ☐
- White & Black African ☐
- White & Asian ☐
- Other mixed background ☐

Black or Black British ☐
or
- Carribean ☐
- African ☐
- Other Black background ☐

Asian or Asian British ☐
or
- Indian ☐
- Pakistani ☐
- Bangladeshi ☐
- Other Asian background ☐

Chinese or other ethnic group ☐
or
- Chinese ☐
- Other ethnic group ☐

11.

Do you live alone?

Yes ☐ **Please go to Question 12**

No ☐ **Please say who you live with below**

Who do you live with?
(e.g. wife, husband, partner, family member)

☐

12. Are you suffering from, or have you suffered from, any of the following in the past 2 years?

Please cross any that are applicable to you

- Asthma ☐
- Chronic Obstructive Pulmonary Disease (COPD) ☐
- Other lung disease (e.g. fibrosis, bronchiectatis etc) ☐
- Heart disease ☐
- Anxiety or depression ☐
- Inflammatory bowel disease ☐
- Irritable bowel syndrome ☐
- Peptic ulcer ☐
- Previous cancer ☐
- Diabetes ☐
- Arthritis ☐

13. About smoking

Please cross the appropriate statement

- Are you a current smoker? ☐
- Are you an ex-smoker? ☐
- Are you a non-smoker (never smoked)? ☐

14. Do you think you were more at risk of getting cancer because of your family history?

Yes ☐

No ☐

15. The following questions are about how completing the questionnaire made you feel.

A number of statements which people have used to describe themselves are given below. Read each statement and then cross the most appropriate box to indicate how you feel right now, at the moment. There are no right or wrong answers. Do not spend too much time on any one statement but give the answer which seems to describe your present feeling best.

		Not at all	Somewhat	Moderately	Very much
1.	I feel calm	☐	☐	☐	☐
2.	I am tense	☐	☐	☐	☐
3.	I feel upset	☐	☐	☐	☐
4.	I am relaxed	☐	☐	☐	☐
5.	I feel content	☐	☐	☐	☐
6.	I am worried	☐	☐	☐	☐

Please make sure you have answered all the questions.

If you have any other comments or there is anything else you would like to tell us about when you first noticed your symptoms, please do so using the space below.

If you feel you would like to talk to someone about this questionnaire at a later stage, please speak to the doctor or nurse who is looking after you, or be in touch with the research team, whose contact details are on your patient information sheet

Thank you for completing the questionnaire

Please give it to the research nurse

The next page is for the Researcher to complete

Researcher - Post-completion Sheet **Researcher initials:** ☐☐☐

Date questionnaire completed: Date ☐☐ / ☐☐ / ☐☐ Time ☐☐:☐☐

If not completed, give reason ☐

How long is it since the patient was told of his/her diagnosis? ☐

How long did it take the patient to complete the questionnaire?

- Less than 5 minutes ☐
- Between 5 and 10 minutes ☐
- Between 11 and 15 minutes ☐
- Longer than 15 minutes ☐

For the patient selected to complete the questionnaire on their own

Did the patient ask for any help? ☐ Yes ☐ No

If yes,

please specify what help was requested

____

____

please specify what help was given

____

____

For the patient selected for the researcher-administered questionnaire

Did the patient find any of the questions difficult? ☐ Yes ☐ No

If yes,

please specify which questions and what the difficulty was

____

____

____

____

Was the patient made anxious? ☐ Yes ☐ No

If yes,

please give details

____

____

____

____

Where did the patient complete the questionnaire?

(Please specify whether this was in a separate room alone, in a corner of the clinic, the ward or other

____

Thank you. Please now send the questionnaire and patient information sheet and consent form back to Cardiff University in the freepost envelope provided.

57727

PID - 2 0

Ymddiriedolaeth GIG Gogledd Orllewin Cymru
North West Wales NHS Trust

NHS WALES GIG CYMRU
Ymddiriedolaeth GIG Gogledd Cymru
North Wales NHS Trust

☐ GLAN CLWYD

CID - ☐ GWYNEDD

☐ MAELOR

Confidential Questionnaire

Looking At Your Symptoms Study

This Study has been funded by Cancer Research UK

Cardiff University / Prifysgol Caerdydd
Ysgol Glinigol Gogledd Cymru / North Wales Clinical School
Cancer Research UK
sewtu South East Wales Trials Unit / Uned Ymchwil De-ddwyrain Cymru

Dear Patient

I am very grateful to you for agreeing to help us with this study.

I hope the information you supply will help us give people a diagnosis earlier.

If you feel you would like to talk to someone about this questionnaire at a later stage, please speak to the doctor or nurse who is looking after you, or be in touch with the research team, whose contact details are on your patient information sheet.

I can assure you that anything you tell us will be treated in the utmost confidence.

Yours sincerely

Dr Richard Neal
Senior Clinical Lecturer
Cardiff University
Chief Investigator, Symptoms Study

About your symptoms

These questions are about symptoms you noticed before your diagnosis. We are interested in symptoms which you think are related to your cancer.

Please put a cross in the appropriate box, or write an answer as required.

Looking back...

1. Did you have any symptoms that you feel were due to your cancer before you were diagnosed?

Yes ☐ **Please continue with Question 2**

No ☐ **Please go to Section 2 on Page 5**

2. What was the first symptom that made you think something might be wrong?

[ ]

Please go to Question 3

The following questions are about when you first noticed a symptom and when you first told your GP or nurse about it.

Please give an exact date if you can. Otherwise give your best estimate. (For example approximately how long ago, the month or season). You wish to refer to your diary or calendar if you have it with you.

Here is an example question:

Decrease in appetite

Did you have this symptom?

Yes ☒ **Please complete A then B or C below**

No ☐ **Please go to Section 2**

A When did you first notice this? d d / m m / y y **OR** Estimate '3 months ago' or 'June'

B When did you first tell your GP or nurse? 2 3 / 1 2 / 0 7 **OR** Estimate

OR

C Put a cross here if you didn't tell your GP or nurse ☐

3. Change in bowel habit

Did you have this symptom?

Yes ☐ **Please complete A then B or C below**

No ☐ **Please go to Question 4**

A When did you first notice this? ☐☐ / ☐☐ / ☐☐ **OR** ☐

B When did you first tell your GP or nurse? ☐☐ / ☐☐ / ☐☐ **OR** ☐

OR

C Put a cross here if you didn't tell your GP or nurse ☐

4. Bleeding from back passage

Did you have this symptom?

Yes ☐ **Please complete A then B or C below**

No ☐ **Please go to Section 2**

A When did you first notice this? ☐☐ / ☐☐ / ☐☐ **OR** ☐

B When did you first tell your GP or nurse? ☐☐ / ☐☐ / ☐☐ **OR** ☐

OR

C Put a cross here if you didn't tell your GP or nurse ☐

Please give an exact date if you can. Otherwise give your best estimate (for example approximately how long ago, the month or the season). You may wish to refer to your diary or calendar if you have it with you.

Did you have any of the following?

1. Decrease in appetite

Did you have this?

Yes ☐ **Please complete A then B or C below**

No ☐ **Please go to Question 2**

A When did you first notice this? ☐☐ / ☐☐ / ☐☐ **OR** ☐

B When did you first tell your GP or nurse? ☐☐ / ☐☐ / ☐☐ **OR** ☐

OR

C Put a cross here if you didn't tell your GP or nurse ☐

2. Unexplained weight loss

Did you have this?

Yes ☐ **Please complete A then B or C below**

No ☐ **Please go to Question 3**

A When did you first notice this? ☐☐ / ☐☐ / ☐☐ **OR** ☐

B When did you first tell your GP or nurse? ☐☐ / ☐☐ / ☐☐ **OR** ☐

OR

C Put a cross here if you didn't tell your GP or nurse ☐

3. **Fatigue or tiredness that is unusual for you**

Did you have this?

Yes ☐ **Please complete A then B or C below**

No ☐ **Please go to Question 4**

A When did you first notice this? ☐☐ / ☐☐ / ☐☐ **OR** ☐

B When did you first tell your GP or nurse? ☐☐ / ☐☐ / ☐☐ **OR** ☐

OR

C Put a cross here if you didn't tell your GP or nurse ☐

4. **Feeling different 'in yourself' from usual**

Did you have this?

Yes ☐ **Please complete A then B or C below**

No ☐ **Please go to Question 5**

A When did you first notice this? ☐☐ / ☐☐ / ☐☐ **OR** ☐

B When did you first tell your GP or nurse? ☐☐ / ☐☐ / ☐☐ **OR** ☐

OR

C Put a cross here if you didn't tell your GP or nurse ☐

Please try and describe what the feeling was

If you had any further symptoms which you feel are relevant to your cancer diagnosis, please list them below. If not, please go to Question 7.

Please describe the symptom here and complete A then B or C below

5.

**A**	When did you first notice this?	☐☐ / ☐☐ / ☐☐ **OR** ☐
**B**	When did you first tell your GP or nurse?	☐☐ / ☐☐ / ☐☐ **OR** ☐
	**OR**	
**C**	Put a cross here if you didn't tell your GP or nurse	☐

If you had another symptom you feel is relevant, please write it below, otherwise go to Question 7.

Please describe the symptom here and complete A then B or C below

6.

**A**	When did you first notice this?	☐☐ / ☐☐ / ☐☐ **OR** ☐
**B**	When did you first tell your GP or nurse?	☐☐ / ☐☐ / ☐☐ **OR** ☐
	**OR**	
**C**	Put a cross here if you didn't tell your GP or nurse	☐

If you had further symptoms you would like to tell us about, there is space at the end of the Questionnaire for you to write them.

Did your GP send you for any tests?

Thinking back to the time immediately before you diagnosis, did your GP send you for any of the following tests?

Please cross the appropriate boxes

7. Were you sent for any of these?

	Yes	Not sure	No
Blood test(s)	☐	☐	☐
CT Scan	☐	☐	☐
Ultrasound scan	☐	☐	☐
Barium Enema	☐	☐	☐
Sigmoidoscopy or colonoscopy (Looking at bowel with internal camera)	☐	☐	☐
X-ray	☐	☐	☐

Please go to Question 8

About you

The next questions relate to general information about you. It would help us to know more about the background of the people completing the questionnaire.

8. Which best describes your employment status?

Please cross one box only

- Employed full-time ☐
- Employed part-time ☐
- Self employed full-time ☐
- Self employed part-time ☐
- Unemployed (seeking work) ☐
- Unemployed (not seeking work) ☐
- Retired ☐
- Student ☐
- Permanently sick/disabled ☐
- Temporarily sick/disabled ☐
- Looking after family/home ☐
- Other, please describe ______

9. What is your highest level of qualification?

Please cross one box only

- Degree (or equivalent) ☐
- Diploma (or equivalent) ☐
- 'A' level ☐
- GCSE / 'O' level ☐
- None ☐
- Other, please specify ______

10. **How would you describe your ethnicity**

Please cross one box only

White ☐
or
- White British ☐
- White Irish ☐
- Other White background ☐

Mixed ☐
or
- White & Black Carribean ☐
- White & Black African ☐
- White & Asian ☐
- Other mixed background ☐

Black or Black British ☐
or
- Carribean ☐
- African ☐
- Other Black background ☐

Asian or Asian British ☐
or
- Indian ☐
- Pakistani ☐
- Bangladeshi ☐
- Other Asian background ☐

Chinese or other ethnic group ☐
or
- Chinese ☐
- Other ethnic group ☐

11.

Do you live alone?

Yes ☐ **Please go to Question 12**

No ☐ **Please say who you live with below**

Who do you live with?
(e.g. wife, husband, partner, family member)

12. Are you suffering from, or have you suffered from, any of the following in the past 2 years?

Please cross any that are applicable to you

- Asthma ☐
- Chronic Obstructive Pulmonary Disease (COPD) ☐
- Other lung disease (e.g. fibrosis, bronchiectatis etc) ☐
- Heart disease ☐
- Anxiety or depression ☐
- Inflammatory bowel disease ☐
- Irritable bowel syndrome ☐
- Peptic ulcer ☐
- Previous cancer ☐
- Diabetes ☐
- Arthritis ☐

13. About smoking

Please cross the appropriate statement

- Are you a current smoker? ☐
- Are you an ex-smoker? ☐
- Are you a non-smoker (never smoked)? ☐

14. Do you think you were more at risk of getting cancer because of your family history?

Yes ☐
No ☐

15. The following questions are about how completing the questionnaire made you feel.

A number of statements which people have used to describe themselves are given below. Read each statement and then cross the most appropriate box to indicate how you feel right now, at the moment. There are no right or wrong answers. Do not spend too much time on any one statement but give the answer which seems to describe your present feeling best.

		Not at all	Somewhat	Moderately	Very much
1.	I feel calm	☐	☐	☐	☐
2.	I am tense	☐	☐	☐	☐
3.	I feel upset	☐	☐	☐	☐
4.	I am relaxed	☐	☐	☐	☐
5.	I feel content	☐	☐	☐	☐
6.	I am worried	☐	☐	☐	☐

Please make sure you have answered all the questions.

If you have any other comments or there is anything else you would like to tell us about when you first noticed your symptoms, please do so using the space below.

If you feel you would like to talk to someone about this questionnaire at a later stage, please speak to the doctor or nurse who is looking after you, or be in touch with the research team, whose contact details are on your patient information sheet

Thank you for completing the questionnaire

Please give it to the research nurse

The next page is for the Researcher to complete

Researcher - Post-completion Sheet

Researcher initials: ☐☐☐

Date questionnaire completed: Date ☐☐ / ☐☐ / ☐☐ Time ☐☐:☐☐

If not completed, give reason ☐

How long is it since the patient was told of his/her diagnosis? ☐

How long did it take the patient to complete the questionnaire?

- Less than 5 minutes ☐
- Between 5 and 10 minutes ☐
- Between 11 and 15 minutes ☐
- Longer than 15 minutes ☐

For the patient selected to <u>complete the questionnaire on their own</u>

Did the patient ask for any help? ☐ Yes ☐ No

If yes,

please specify what help was requested

____

____

please specify what help was given

____

____

For the patient selected for <u>the researcher-administered questionnaire</u>

Did the patient find any of the questions difficult? ☐ Yes ☐ No

If yes,

please specify which questions and what the difficulty was

____

____

____

____

Was the patient made anxious? ☐ Yes ☐ No

If yes,

please give details

____

____

____

____

Where did the patient complete the questionnaire?

(Please specify whether this was in a separate room alone, in a corner of the clinic, the ward or other

____

Thank you. Please now send the questionnaire and patient information sheet and consent form back to Cardiff University in the freepost envelope provided.

57727

PID - | 2 | 1

Ymddiriedolaeth GIG Gogledd Orllewin Cymru
North West Wales NHS Trust

NHS WALES GIG CYMRU
Ymddiriedolaeth GIG Gogledd Cymru
North Wales NHS Trust

☐ GLAN CLWYD

CID - ☐ GWYNEDD

☐ MAELOR

Confidential Questionnaire

Looking At Your Symptoms Study

This Study has been funded by Cancer Research UK

Cardiff University / Prifysgol Caerdydd — Ysgol Glinigol Gogledd Cymru / North Wales Clinical School — Cancer Research UK — sewtu: South East Wales Trials Unit / Uned Ymchwil De-ddwyrain Cymru

Dear Patient

I am very grateful to you for agreeing to help us with this study.

I hope the information you supply will help us give people a diagnosis earlier.

If you feel you would like to talk to someone about this questionnaire at a later stage, please speak to the doctor or nurse who is looking after you, or be in touch with the research team, whose contact details are on your patient information sheet.

I can assure you that anything you tell us will be treated in the utmost confidence.

Yours sincerely

Richard Neal

Dr Richard Neal
Senior Clinical Lecturer
Cardiff University
Chief Investigator, Symptoms Study

About your symptoms

These questions are about symptoms you noticed before your diagnosis. We are interested in symptoms which you think are related to your cancer.

Please put a cross in the appropriate box, or write an answer as required.

Looking back...

1. Did you have any symptoms that you feel were due to your cancer before you were diagnosed?

Yes ☐ **Please continue with Question 2**

No ☐ **Please go to Section 2 on Page 5**

2. What was the first symptom that made you think something might be wrong?

☐

Please go to Question 3

The following questions are about when you first noticed a symptom and when you first told your GP or nurse about it.

Please give an exact date if you can. Otherwise give your best estimate. (For example approximately how long ago, the month or season). You wish to refer to your diary or calendar if you have it with you.

Here is an example question:

Decrease in appetite

Did you have this symptom?

Yes ☒ **Please complete A then B or C below**

No ☐ **Please go to Section 2**

A When did you first notice this? d d / m m / y y **OR** Estimate: '3 months ago' or 'June'

B When did you first tell your GP or nurse? 2 3 / 1 2 / 0 7 **OR** Estimate

OR

C Put a cross here if you didn't tell your GP or nurse ☐

3. Change in bowel habit

Did you have this symptom?

Yes ☐ **Please complete A then B or C below**

No ☐ **Please go to Question 4**

A When did you first notice this? ☐☐ / ☐☐ / ☐☐ **OR** ☐

B When did you first tell your GP or nurse? ☐☐ / ☐☐ / ☐☐ **OR** ☐

OR

C Put a cross here if you didn't tell your GP or nurse ☐

4. Bleeding from back passage

Did you have this symptom?

Yes ☐ **Please complete A then B or C below**

No ☐ **Please go to Section 2**

A When did you first notice this? ☐☐ / ☐☐ / ☐☐ **OR** ☐

B When did you first tell your GP or nurse? ☐☐ / ☐☐ / ☐☐ **OR** ☐

OR

C Put a cross here if you didn't tell your GP or nurse ☐

Section 2

Please give an exact date if you can. Otherwise give your best estimate (for example approximately how long ago, the month or the season). You may wish to refer to your diary or calendar if you have it with you.

Did you have any of the following?

1. Decrease in appetite

Did you have this?

Yes ☐ **Please complete A then B or C below**

No ☐ **Please go to Question 2**

A When did you first notice this? ☐☐ / ☐☐ / ☐☐ **OR** ☐

B When did you first tell your GP or nurse? ☐☐ / ☐☐ / ☐☐ **OR** ☐

OR

C Put a cross here if you didn't tell your GP or nurse ☐

2. Unexplained weight loss

Did you have this?

Yes ☐ **Please complete A then B or C below**

No ☐ **Please go to Question 3**

A When did you first notice this? ☐☐ / ☐☐ / ☐☐ **OR** ☐

B When did you first tell your GP or nurse? ☐☐ / ☐☐ / ☐☐ **OR** ☐

OR

C Put a cross here if you didn't tell your GP or nurse ☐

3. Fatigue or tiredness that is unusual for you

Did you have this?

Yes ☐ **Please complete A then B or C below**

No ☐ **Please go to Question 4**

A When did you first notice this? ☐☐ / ☐☐ / ☐☐ **OR** ☐

B When did you first tell your GP or nurse? ☐☐ / ☐☐ / ☐☐ **OR** ☐

OR

C Put a cross here if you didn't tell your GP or nurse ☐

4. Feeling different 'in yourself' from usual

Did you have this?

Yes ☐ **Please complete A then B or C below**

No ☐ **Please go to Question 5**

A When did you first notice this? ☐☐ / ☐☐ / ☐☐ **OR** ☐

B When did you first tell your GP or nurse? ☐☐ / ☐☐ / ☐☐ **OR** ☐

OR

C Put a cross here if you didn't tell your GP or nurse ☐

Please try and describe what the feeling was

If you had any further symptoms which you feel are relevant to your cancer diagnosis, please list them below. If not, please go to Question 7.

Please describe the symptom here and complete A then B or C below

5.

**A**	When did you first notice this?	☐☐ / ☐☐ / ☐☐ **OR** ☐
**B**	When did you first tell your GP or nurse?	☐☐ / ☐☐ / ☐☐ **OR** ☐
	**OR**	
**C**	Put a cross here if you didn't tell your GP or nurse	☐

If you had another symptom you feel is relevant, please write it below, otherwise go to Question 7.

Please describe the symptom here and complete A then B or C below

6.

**A**	When did you first notice this?	☐☐ / ☐☐ / ☐☐ **OR** ☐
**B**	When did you first tell your GP or nurse?	☐☐ / ☐☐ / ☐☐ **OR** ☐
	**OR**	
**C**	Put a cross here if you didn't tell your GP or nurse	☐

If you had further symptoms you would like to tell us about, there is space at the end of the Questionnaire for you to write them.

Did your GP send you for any tests?

Thinking back to the time immediately before you diagnosis, did your GP send you for any of the following tests?

Please cross the appropriate boxes

7. Were you sent for any of these?

	Yes	Not sure	No
Blood test(s)	☐	☐	☐
CT Scan	☐	☐	☐
Ultrasound scan	☐	☐	☐
Barium Enema	☐	☐	☐
Sigmoidoscopy or colonoscopy (Looking at bowel with internal camera)	☐	☐	☐
X-ray	☐	☐	☐

Please go to Question 8

About you

The next questions relate to general information about you. It would help us to know more about the background of the people completing the questionnaire.

8. Which best describes your employment status?

Please cross one box only

- Employed full-time ☐
- Employed part-time ☐
- Self employed full-time ☐
- Self employed part-time ☐
- Unemployed (seeking work) ☐
- Unemployed (not seeking work) ☐
- Retired ☐
- Student ☐
- Permanently sick/disabled ☐
- Temporarily sick/disabled ☐
- Looking after family/home ☐
- Other, please describe ______

9. What is your highest level of qualification?

Please cross one box only

- Degree (or equivalent) ☐
- Diploma (or equivalent) ☐
- 'A' level ☐
- GCSE / 'O' level ☐
- None ☐
- Other, please specify ______

10. **How would you describe your ethnicity**

Please cross one box only

White ☐
or
White British ☐
White Irish ☐
Other White background ☐

Mixed ☐
or
White & Black Carribean ☐
White & Black African ☐
White & Asian ☐
Other mixed background ☐

Black or Black British ☐
or
Carribean ☐
African ☐
Other Black background ☐

Asian or Asian British ☐
or
Indian ☐
Pakistani ☐
Bangladeshi ☐
Other Asian background ☐

Chinese or other ethnic group ☐
or
Chinese ☐
Other ethnic group ☐

11.

Do you live alone?

Yes ☐ **Please go to Question 12**

No ☐ **Please say who you live with below**

Who do you live with?
(e.g. wife, husband, partner, family member)

12. **Are you suffering from, or have you suffered from, any of the following in the past 2 years?**

Please cross any that are applicable to you

- Asthma ☐
- Chronic Obstructive Pulmonary Disease (COPD) ☐
- Other lung disease (e.g. fibrosis, bronchiectatis etc) ☐
- Heart disease ☐
- Anxiety or depression ☐
- Inflammatory bowel disease ☐
- Irritable bowel syndrome ☐
- Peptic ulcer ☐
- Previous cancer ☐
- Diabetes ☐
- Arthritis ☐

13. **About smoking**

Please cross the appropriate statement

- Are you a current smoker? ☐
- Are you an ex-smoker? ☐
- Are you a non-smoker (never smoked)? ☐

14. **Do you think you were more at risk of getting cancer because of your family history?**

Yes ☐

No ☐

15. The following questions are about how completing the questionnaire made you feel.

A number of statements which people have used to describe themselves are given below. Read each statement and then cross the most appropriate box to indicate how you feel right now, at the moment. There are no right or wrong answers. Do not spend too much time on any one statement but give the answer which seems to describe your present feeling best.

		Not at all	Somewhat	Moderately	Very much
1.	I feel calm	☐	☐	☐	☐
2.	I am tense	☐	☐	☐	☐
3.	I feel upset	☐	☐	☐	☐
4.	I am relaxed	☐	☐	☐	☐
5.	I feel content	☐	☐	☐	☐
6.	I am worried	☐	☐	☐	☐

Please make sure you have answered all the questions.

If you have any other comments or there is anything else you would like to tell us about when you first noticed your symptoms, please do so using the space below.

If you feel you would like to talk to someone about this questionnaire at a later stage, please speak to the doctor or nurse who is looking after you, or be in touch with the research team, whose contact details are on your patient information sheet

Thank you for completing the questionnaire

Please give it to the research nurse

The next page is for the Researcher to complete

57727

2 1

Researcher - Post-completion Sheet

Researcher initials: ☐☐☐

Date questionnaire completed: Date ☐☐ / ☐☐ / ☐☐ Time ☐☐:☐☐

If not completed, give reason ☐

How long is it since the patient was told of his/her diagnosis? ☐

How long did it take the patient to complete the questionnaire?

- Less than 5 minutes ☐
- Between 5 and 10 minutes ☐
- Between 11 and 15 minutes ☐
- Longer than 15 minutes ☐

For the patient selected to complete the questionnaire on their own

Did the patient ask for any help? ☐ Yes ☐ No

If yes,

please specify what help was requested

____

____

please specify what help was given

____

____

For the patient selected for the researcher-administered questionnaire

Did the patient find any of the questions difficult? ☐ Yes ☐ No

If yes,

please specify which questions and what the difficulty was

____

____

____

____

Was the patient made anxious? ☐ Yes ☐ No

If yes,

please give details

____

____

____

____

Where did the patient complete the questionnaire?

(Please specify whether this was in a separate room alone, in a corner of the clinic, the ward or other

____

Thank you. Please now send the questionnaire and patient information sheet and consent form back to Cardiff University in the freepost envelope provided.

57727

PID - | | 2 | 2 |

Ymddiriedolaeth GIG Gogledd Orllewin Cymru
North West Wales NHS Trust

NHS Wales GIG Cymru
Ymddiriedolaeth GIG Gogledd Cymru
North Wales NHS Trust

☐ GLAN CLWYD

CID - ☐ GWYNEDD

☐ MAELOR

Confidential Questionnaire

Looking At Your Symptoms Study

This Study has been funded by Cancer Research UK

Cardiff University Prifysgol Caerdydd
Ysgol Glinigol Gogledd Cymru North Wales Clinical School
Cancer Research UK
sewtu South East Wales Trials Unit Uned Ymchwil De-ddwyrain Cymru

Dear Patient

I am very grateful to you for agreeing to help us with this study.

I hope the information you supply will help us give people a diagnosis earlier.

If you feel you would like to talk to someone about this questionnaire at a later stage, please speak to the doctor or nurse who is looking after you, or be in touch with the research team, whose contact details are on your patient information sheet.

I can assure you that anything you tell us will be treated in the utmost confidence.

Yours sincerely

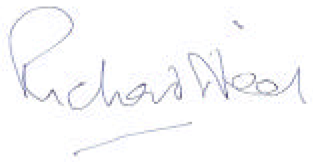

Dr Richard Neal
Senior Clinical Lecturer
Cardiff University
Chief Investigator, Symptoms Study

About your symptoms

These questions are about symptoms you noticed before your diagnosis. We are interested in symptoms which you think are related to your cancer.

Please put a cross in the appropriate box, or write an answer as required.

Looking back...

1. Did you have any symptoms that you feel were due to your cancer before you were diagnosed?

Yes ☐ **Please continue with Question 2**

No ☐ **Please go to Section 2 on Page 5**

2. What was the first symptom that made you think something might be wrong?

☐

Please go to Question 3

The following questions are about when you first noticed a symptom and when you first told your GP or nurse about it.

Please give an exact date if you can. Otherwise give your best estimate. (For example approximately how long ago, the month or season). You wish to refer to your diary or calendar if you have it with you.

Here is an example question:

Decrease in appetite

Did you have this symptom?

Yes ☒ **Please complete A then B or C below**

No ☐ **Please go to Section 2**

A When did you first notice this? d d / m m / y y **OR** Estimate '3 months ago' or 'June'

B When did you first tell your GP or nurse? 2 3 / 1 2 / 0 7 **OR** Estimate

OR

C Put a cross here if you didn't tell your GP or nurse ☐

3. Change in bowel habit

Did you have this symptom?

Yes ☐ **Please complete A then B or C below**

No ☐ **Please go to Question 4**

A When did you <u>first notice</u> this? ☐☐ / ☐☐ / ☐☐ **OR** ☐

B When did you <u>first tell</u> your GP or nurse? ☐☐ / ☐☐ / ☐☐ **OR** ☐

OR

C Put a cross here if you <u>didn't tell</u> your GP or nurse ☐

4. Bleeding from back passage

Did you have this symptom?

Yes ☐ **Please complete A then B or C below**

No ☐ **Please go to Section 2**

A When did you <u>first notice</u> this? ☐☐ / ☐☐ / ☐☐ **OR** ☐

B When did you <u>first tell</u> your GP or nurse? ☐☐ / ☐☐ / ☐☐ **OR** ☐

OR

C Put a cross here if you <u>didn't tell</u> your GP or nurse ☐

Please give an exact date if you can. Otherwise give your best estimate (for example approximately how long ago, the month or the season). You may wish to refer to your diary or calendar if you have it with you.

Did you have any of the following?

1. Decrease in appetite

Did you have this?

Yes ☐ **Please complete A then B or C below**

No ☐ **Please go to Question 2**

A When did you first notice this? ☐☐ / ☐☐ / ☐☐ **OR** ☐

B When did you first tell your GP or nurse? ☐☐ / ☐☐ / ☐☐ **OR** ☐

OR

C Put a cross here if you didn't tell your GP or nurse ☐

2. Unexplained weight loss

Did you have this?

Yes ☐ **Please complete A then B or C below**

No ☐ **Please go to Question 3**

A When did you first notice this? ☐☐ / ☐☐ / ☐☐ **OR** ☐

B When did you first tell your GP or nurse? ☐☐ / ☐☐ / ☐☐ **OR** ☐

OR

C Put a cross here if you didn't tell your GP or nurse ☐

3. Fatigue or tiredness that is unusual for you

Did you have this?

Yes ☐ **Please complete A then B or C below**

No ☐ **Please go to Question 4**

A When did you first notice this? ☐☐ / ☐☐ / ☐☐ **OR** ☐

B When did you first tell your GP or nurse? ☐☐ / ☐☐ / ☐☐ **OR** ☐

OR

C Put a cross here if you didn't tell your GP or nurse ☐

4. Feeling different 'in yourself' from usual

Did you have this?

Yes ☐ **Please complete A then B or C below**

No ☐ **Please go to Question 5**

A When did you first notice this? ☐☐ / ☐☐ / ☐☐ **OR** ☐

B When did you first tell your GP or nurse? ☐☐ / ☐☐ / ☐☐ **OR** ☐

OR

C Put a cross here if you didn't tell your GP or nurse ☐

Please try and describe what the feeling was

If you had any further symptoms which you feel are relevant to your cancer diagnosis, please list them below. If not, please go to Question 7.

Please describe the symptom here and complete A then B or C below

5.

**A**	When did you first notice this?	__ __ / __ __ / __ __ **OR** ______
**B**	When did you first tell your GP or nurse?	__ __ / __ __ / __ __ **OR** ______
	**OR**	
**C**	Put a cross here if you didn't tell your GP or nurse	☐

If you had another symptom you feel is relevant, please write it below, otherwise go to Question 7.

Please describe the symptom here and complete A then B or C below

6.

**A**	When did you first notice this?	__ __ / __ __ / __ __ **OR** ______
**B**	When did you first tell your GP or nurse?	__ __ / __ __ / __ __ **OR** ______
	**OR**	
**C**	Put a cross here if you didn't tell your GP or nurse	☐

If you had further symptoms you would like to tell us about, there is space at the end of the Questionnaire for you to write them.

Did your GP send you for any tests?

Thinking back to the time immediately before you diagnosis, did your GP send you for any of the following tests?

Please cross the appropriate boxes

7. Were you sent for any of these?

	Yes	Not sure	No
Blood test(s)	☐	☐	☐
CT Scan	☐	☐	☐
Ultrasound scan	☐	☐	☐
Barium Enema	☐	☐	☐
Sigmoidoscopy or colonoscopy (Looking at bowel with internal camera)	☐	☐	☐
X-ray	☐	☐	☐

Please go to Question 8

About you

The next questions relate to general information about you. It would help us to know more about the background of the people completing the questionnaire.

8. **Which best describes your employment status?**

Please cross one box only

- Employed full-time ☐
- Employed part-time ☐
- Self employed full-time ☐
- Self employed part-time ☐
- Unemployed (seeking work) ☐
- Unemployed (not seeking work) ☐
- Retired ☐
- Student ☐
- Permanently sick/disabled ☐
- Temporarily sick/disabled ☐
- Looking after family/home ☐
- Other, please describe ____________

9. **What is your highest level of qualification?**

Please cross one box only

- Degree (or equivalent) ☐
- Diploma (or equivalent) ☐
- 'A' level ☐
- GCSE / 'O' level ☐
- None ☐
- Other, please specify ____________

10. How would you describe your ethnicity

Please cross one box only

White ☐
or
- White British ☐
- White Irish ☐
- Other White background ☐

Mixed ☐
or
- White & Black Carribean ☐
- White & Black African ☐
- White & Asian ☐
- Other mixed background ☐

Black or Black British ☐
or
- Carribean ☐
- African ☐
- Other Black background ☐

Asian or Asian British ☐
or
- Indian ☐
- Pakistani ☐
- Bangladeshi ☐
- Other Asian background ☐

Chinese or other ethnic group ☐
or
- Chinese ☐
- Other ethnic group ☐

11.

Do you live alone?

Yes ☐ **Please go to Question 12**

No ☐ **Please say who you live with below**

Who do you live with?
(e.g. wife, husband, partner, family member)

12. **Are you suffering from, or have you suffered from, any of the following in the past 2 years?**

Please cross any that are applicable to you

- Asthma ☐
- Chronic Obstructive Pulmonary Disease (COPD) ☐
- Other lung disease (e.g. fibrosis, bronchiectatis etc) ☐
- Heart disease ☐
- Anxiety or depression ☐
- Inflammatory bowel disease ☐
- Irritable bowel syndrome ☐
- Peptic ulcer ☐
- Previous cancer ☐
- Diabetes ☐
- Arthritis ☐

13. **About smoking**

Please cross the appropriate statement

- Are you a current smoker? ☐
- Are you an ex-smoker? ☐
- Are you a non-smoker (never smoked)? ☐

14. **Do you think you were more at risk of getting cancer because of your family history?**

Yes ☐

No ☐

15. The following questions are about how completing the questionnaire made you feel.

A number of statements which people have used to describe themselves are given below. Read each statement and then cross the most appropriate box to indicate how you feel <u>right now, at the moment.</u> There are no right or wrong answers. Do not spend too much time on any one statement but give the answer which seems to describe your present feeling best.

		Not at all	Somewhat	Moderately	Very much
1.	I feel calm	☐	☐	☐	☐
2.	I am tense	☐	☐	☐	☐
3.	I feel upset	☐	☐	☐	☐
4.	I am relaxed	☐	☐	☐	☐
5.	I feel content	☐	☐	☐	☐
6.	I am worried	☐	☐	☐	☐

Please make sure you have answered all the questions.

If you have any other comments or there is anything else you would like to tell us about when you first noticed your symptoms, please do so using the space below.

If you feel you would like to talk to someone about this questionnaire at a later stage, please speak to the doctor or nurse who is looking after you, or be in touch with the research team, whose contact details are on your patient information sheet

Thank you for completing the questionnaire

Please give it to the research nurse

The next page is for the Researcher to complete

Researcher - Post-completion Sheet

Researcher initials: ☐☐☐

Date questionnaire completed: Date ☐☐ / ☐☐ / ☐☐ Time ☐☐ : ☐☐

If not completed, give reason ☐

How long is it since the patient was told of his/her diagnosis? ☐

How long did it take the patient to complete the questionnaire?

- Less than 5 minutes ☐
- Between 5 and 10 minutes ☐
- Between 11 and 15 minutes ☐
- Longer than 15 minutes ☐

For the patient selected to complete the questionnaire on their own

Did the patient ask for any help? ☐ Yes ☐ No

If yes,

please specify what help was requested

____

____

please specify what help was given

____

____

For the patient selected for the researcher-administered questionnaire

Did the patient find any of the questions difficult? ☐ Yes ☐ No

If yes,

please specify which questions and what the difficulty was

____

____

____

____

Was the patient made anxious? ☐ Yes ☐ No

If yes,

please give details

____

____

____

____

Where did the patient complete the questionnaire?

(Please specify whether this was in a separate room alone, in a corner of the clinic, the ward or other

____

Thank you. Please now send the questionnaire and patient information sheet and consent form back to Cardiff University in the freepost envelope provided.

57727

PID - | | 2 | 3 |

Ymddiriedolaeth GIG Gogledd Orllewin Cymru
North West Wales NHS Trust

NHS WALES GIG CYMRU
Ymddiriedolaeth GIG Gogledd Cymru
North Wales NHS Trust

☐ GLAN CLWYD

CID - ☐ GWYNEDD

☐ MAELOR

Confidential Questionnaire

Looking At Your Symptoms Study

This Study has been funded by Cancer Research UK

CARDIFF UNIVERSITY PRIFYSGOL CAERDYDD

Ysgol Glinigol Gogledd Cymru
North Wales Clinical School

CANCER RESEARCH UK

sewtu
South East Wales Trials Unit
Uned Ymchwil De-ddwyrain Cymru

Dear Patient

I am very grateful to you for agreeing to help us with this study.

I hope the information you supply will help us give people a diagnosis earlier.

If you feel you would like to talk to someone about this questionnaire at a later stage, please speak to the doctor or nurse who is looking after you, or be in touch with the research team, whose contact details are on your patient information sheet.

I can assure you that anything you tell us will be treated in the utmost confidence.

Yours sincerely

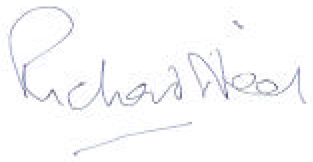

Dr Richard Neal
Senior Clinical Lecturer
Cardiff University
Chief Investigator, Symptoms Study

About your symptoms

These questions are about symptoms you noticed before your diagnosis. We are interested in symptoms which you think are related to your cancer.

Please put a cross in the appropriate box, or write an answer as required.

Looking back...

1. Did you have any symptoms that you feel were due to your cancer before you were diagnosed?

Yes ☐ **Please continue with Question 2**

No ☐ **Please go to Section 2 on Page 5**

2. What was the first symptom that made you think something might be wrong?

☐

Please go to Question 3

The following questions are about when you first noticed a symptom and when you first told your GP or nurse about it.

Please give an exact date if you can. Otherwise give your best estimate. (For example approximately how long ago, the month or season). You wish to refer to your diary or calendar if you have it with you.

Here is an example question:

Decrease in appetite

Did you have this symptom?

Yes ☒ **Please complete A then B or C below**

No ☐ **Please go to Section 2**

A When did you first notice this? d d / m m / y y **OR** Estimate: '3 months ago' or 'June'

B When did you first tell your GP or nurse? 2 3 / 1 2 / 0 7 **OR** Estimate

OR

C Put a cross here if you didn't tell your GP or nurse ☐

3. Change in bowel habit

Did you have this symptom?

Yes ☐ **Please complete A then B or C below**

No ☐ **Please go to Question 4**

A When did you first notice this? __ __ / __ __ / __ __ **OR** ☐

B When did you first tell your GP or nurse? __ __ / __ __ / __ __ **OR** ☐

OR

C Put a cross here if you didn't tell your GP or nurse ☐

4. Bleeding from back passage

Did you have this symptom?

Yes ☐ **Please complete A then B or C below**

No ☐ **Please go to Section 2**

A When did you first notice this? __ __ / __ __ / __ __ **OR** ☐

B When did you first tell your GP or nurse? __ __ / __ __ / __ __ **OR** ☐

OR

C Put a cross here if you didn't tell your GP or nurse ☐

Please give an exact date if you can. Otherwise give your best estimate (for example approximately how long ago, the month or the season). You may wish to refer to your diary or calendar if you have it with you.

Did you have any of the following?

1. Decrease in appetite

Did you have this?

Yes ☐ **Please complete A then B or C below**

No ☐ **Please go to Question 2**

A When did you first notice this? ☐☐ / ☐☐ / ☐☐ **OR** ☐

B When did you first tell your GP or nurse? ☐☐ / ☐☐ / ☐☐ **OR** ☐

OR

C Put a cross here if you didn't tell your GP or nurse ☐

2. Unexplained weight loss

Did you have this?

Yes ☐ **Please complete A then B or C below**

No ☐ **Please go to Question 3**

A When did you first notice this? ☐☐ / ☐☐ / ☐☐ **OR** ☐

B When did you first tell your GP or nurse? ☐☐ / ☐☐ / ☐☐ **OR** ☐

OR

C Put a cross here if you didn't tell your GP or nurse ☐

3. **Fatigue or tiredness that is unusual for you**

Did you have this?

Yes ☐ **Please complete A then B or C below**

No ☐ **Please go to Question 4**

A When did you first notice this? ☐☐ / ☐☐ / ☐☐ **OR** ☐

B When did you first tell your GP or nurse? ☐☐ / ☐☐ / ☐☐ **OR** ☐

OR

C Put a cross here if you didn't tell your GP or nurse ☐

4. **Feeling different 'in yourself' from usual**

Did you have this?

Yes ☐ **Please complete A then B or C below**

No ☐ **Please go to Question 5**

A When did you first notice this? ☐☐ / ☐☐ / ☐☐ **OR** ☐

B When did you first tell your GP or nurse? ☐☐ / ☐☐ / ☐☐ **OR** ☐

OR

C Put a cross here if you didn't tell your GP or nurse ☐

Please try and describe what the feeling was

If you had any further symptoms which you feel are relevant to your cancer diagnosis, please list them below. If not, please go to Question 7.

Please describe the symptom here and complete A then B or C below

5.

**A**	When did you first notice this?	☐☐ / ☐☐ / ☐☐ **OR** ☐
**B**	When did you first tell your GP or nurse?	☐☐ / ☐☐ / ☐☐ **OR** ☐
	**OR**	
**C**	Put a cross here if you didn't tell your GP or nurse	☐

If you had another symptom you feel is relevant, please write it below, otherwise go to Question 7.

Please describe the symptom here and complete A then B or C below

6.

**A**	When did you first notice this?	☐☐ / ☐☐ / ☐☐ **OR** ☐
**B**	When did you first tell your GP or nurse?	☐☐ / ☐☐ / ☐☐ **OR** ☐
	**OR**	
**C**	Put a cross here if you didn't tell your GP or nurse	☐

If you had further symptoms you would like to tell us about, there is space at the end of the Questionnaire for you to write them.

Did your GP send you for any tests?

Thinking back to the time immediately before you diagnosis, did your GP send you for any of the following tests?

Please cross the appropriate boxes

7. Were you sent for any of these?

	Yes	Not sure	No
Blood test(s)	☐	☐	☐
CT Scan	☐	☐	☐
Ultrasound scan	☐	☐	☐
Barium Enema	☐	☐	☐
Sigmoidoscopy or colonoscopy (Looking at bowel with internal camera)	☐	☐	☐
X-ray	☐	☐	☐

Please go to Question 8

About you

The next questions relate to general information about you. It would help us to know more about the background of the people completing the questionnaire.

8. Which best describes your employment status?

Please cross one box only

- Employed full-time ☐
- Employed part-time ☐
- Self employed full-time ☐
- Self employed part-time ☐
- Unemployed (seeking work) ☐
- Unemployed (not seeking work) ☐
- Retired ☐
- Student ☐
- Permanently sick/disabled ☐
- Temporarily sick/disabled ☐
- Looking after family/home ☐
- Other, please describe ____

9. What is your highest level of qualification?

Please cross one box only

- Degree (or equivalent) ☐
- Diploma (or equivalent) ☐
- 'A' level ☐
- GCSE / 'O' level ☐
- None ☐
- Other, please specify ____

10. How would you describe your ethnicity

Please cross one box only

White ☐
or
- White British ☐
- White Irish ☐
- Other White background ☐

Mixed ☐
or
- White & Black Carribean ☐
- White & Black African ☐
- White & Asian ☐
- Other mixed background ☐

Black or Black British ☐
or
- Carribean ☐
- African ☐
- Other Black background ☐

Asian or Asian British ☐
or
- Indian ☐
- Pakistani ☐
- Bangladeshi ☐
- Other Asian background ☐

Chinese or other ethnic group ☐
or
- Chinese ☐
- Other ethnic group ☐

11.

Do you live alone?

Yes ☐ **Please go to Question 12**

No ☐ **Please say who you live with below**

Who do you live with?
(e.g. wife, husband, partner, family member)

12. Are you suffering from, or have you suffered from, any of the following in the past 2 years?

Please cross any that are applicable to you

- Asthma ☐
- Chronic Obstructive Pulmonary Disease (COPD) ☐
- Other lung disease (e.g. fibrosis, bronchiectatis etc) ☐
- Heart disease ☐
- Anxiety or depression ☐
- Inflammatory bowel disease ☐
- Irritable bowel syndrome ☐
- Peptic ulcer ☐
- Previous cancer ☐
- Diabetes ☐
- Arthritis ☐

13. About smoking

Please cross the appropriate statement

- Are you a current smoker? ☐
- Are you an ex-smoker? ☐
- Are you a non-smoker (never smoked)? ☐

14. Do you think you were more at risk of getting cancer because of your family history?

Yes ☐

No ☐

15. The following questions are about how completing the questionnaire made you feel.

A number of statements which people have used to describe themselves are given below. Read each statement and then cross the most appropriate box to indicate how you feel right now, at the moment. There are no right or wrong answers. Do not spend too much time on any one statement but give the answer which seems to describe your present feeling best.

		Not at all	Somewhat	Moderately	Very much
1.	I feel calm	☐	☐	☐	☐
2.	I am tense	☐	☐	☐	☐
3.	I feel upset	☐	☐	☐	☐
4.	I am relaxed	☐	☐	☐	☐
5.	I feel content	☐	☐	☐	☐
6.	I am worried	☐	☐	☐	☐

Please make sure you have answered all the questions.

If you have any other comments or there is anything else you would like to tell us about when you first noticed your symptoms, please do so using the space below.

If you feel you would like to talk to someone about this questionnaire at a later stage, please speak to the doctor or nurse who is looking after you, or be in touch with the research team, whose contact details are on your patient information sheet

Thank you for completing the questionnaire

Please give it to the research nurse

The next page is for the Researcher to complete

57727

2 3

<u>Researcher - Post-completion Sheet</u>

Researcher initials: ☐☐☐

Date questionnaire completed: Date ☐☐ / ☐☐ / ☐☐ Time ☐☐:☐☐

If not completed, give reason ☐

How long is it since the patient was told of his/her diagnosis? ☐

How long did it take the patient to complete the questionnaire?

- Less than 5 minutes ☐
- Between 5 and 10 minutes ☐
- Between 11 and 15 minutes ☐
- Longer than 15 minutes ☐

For the patient selected to <u>complete the questionnaire on their own</u>

Did the patient ask for any help? ☐ Yes ☐ No

If yes,

please specify what help was requested

____

____

please specify what help was given

____

____

For the patient selected for <u>the researcher-administered questionnaire</u>

Did the patient find any of the questions difficult? ☐ Yes ☐ No

If yes,

please specify which questions and what the difficulty was

____

____

____

____

Was the patient made anxious? ☐ Yes ☐ No

If yes,

please give details

____

____

____

____

Where did the patient complete the questionnaire?

(Please specify whether this was in a separate room alone, in a corner of the clinic, the ward or other

____

Thank you. Please now send the questionnaire and patient information sheet and consent form back to Cardiff University in the freepost envelope provided.

57727

PID - | | 2 | 4 |

Ymddiriedolaeth GIG Gogledd Orllewin Cymru
North West Wales NHS Trust

NHS WALES GIG CYMRU
Ymddiriedolaeth GIG Gogledd Cymru
North Wales NHS Trust

CID -
☐ GLAN CLWYD
☐ GWYNEDD
☐ MAELOR

Confidential Questionnaire

Looking At Your Symptoms Study

This Study has been funded by Cancer Research UK

Cardiff University / Prifysgol Caerdydd
Ysgol Glinigol Gogledd Cymru / North Wales Clinical School
Cancer Research UK
sewtu South East Wales Trials Unit / Uned Ymchwil De-ddwyrain Cymru

Dear Patient

I am very grateful to you for agreeing to help us with this study.

I hope the information you supply will help us give people a diagnosis earlier.

If you feel you would like to talk to someone about this questionnaire at a later stage, please speak to the doctor or nurse who is looking after you, or be in touch with the research team, whose contact details are on your patient information sheet.

I can assure you that anything you tell us will be treated in the utmost confidence.

Yours sincerely

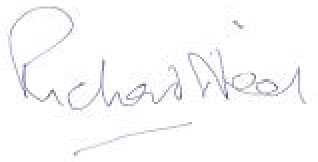

Dr Richard Neal
Senior Clinical Lecturer
Cardiff University
Chief Investigator, Symptoms Study

About your symptoms

These questions are about symptoms you noticed before your diagnosis. We are interested in symptoms which you think are related to your cancer.

Please put a cross in the appropriate box, or write an answer as required.

Looking back...

1. Did you have any symptoms that you feel were due to your cancer before you were diagnosed?

Yes ☐ **Please continue with Question 2**

No ☐ **Please go to Section 2 on Page 5**

2. What was the first symptom that made you think something might be wrong?

☐

Please go to Question 3

The following questions are about when you first noticed a symptom and when you first told your GP or nurse about it.

Please give an exact date if you can. Otherwise give your best estimate. (For example approximately how long ago, the month or season). You wish to refer to your diary or calendar if you have it with you.

Here is an example question:

Decrease in appetite

Did you have this symptom?

Yes ☒ **Please complete A then B or C below**

No ☐ **Please go to Section 2**

A When did you first notice this? d d / m m / y y **OR** Estimate: '3 months ago' or 'June'

B When did you first tell your GP or nurse? 2 3 / 1 2 / 0 7 **OR** Estimate

OR

C Put a cross here if you didn't tell your GP or nurse ☐

3. Change in bowel habit

Did you have this symptom?

Yes ☐ **Please complete A then B or C below**

No ☐ **Please go to Question 4**

A When did you first notice this? ☐☐ / ☐☐ / ☐☐ **OR** ☐

B When did you first tell your GP or nurse? ☐☐ / ☐☐ / ☐☐ **OR** ☐

OR

C Put a cross here if you didn't tell your GP or nurse ☐

4. Bleeding from back passage

Did you have this symptom?

Yes ☐ **Please complete A then B or C below**

No ☐ **Please go to Section 2**

A When did you first notice this? ☐☐ / ☐☐ / ☐☐ **OR** ☐

B When did you first tell your GP or nurse? ☐☐ / ☐☐ / ☐☐ **OR** ☐

OR

C Put a cross here if you didn't tell your GP or nurse ☐

Please give an <u>exact</u> date if you can. Otherwise give your best <u>estimate</u> (for example approximately how long ago, the month or the season). You may wish to refer to your diary or calendar if you have it with you.

Did you have any of the following?

1. Decrease in appetite

Did you have this?

Yes ☐ **Please complete A then B or C below**

No ☐ **Please go to Question 2**

A When did you <u>first notice</u> this? ☐☐ / ☐☐ / ☐☐ **OR** ☐

B When did you <u>first tell</u> your GP or nurse? ☐☐ / ☐☐ / ☐☐ **OR** ☐

OR

C Put a cross here if you <u>didn't tell</u> your GP or nurse ☐

2. Unexplained weight loss

Did you have this?

Yes ☐ **Please complete A then B or C below**

No ☐ **Please go to Question 3**

A When did you <u>first notice</u> this? ☐☐ / ☐☐ / ☐☐ **OR** ☐

B When did you <u>first tell</u> your GP or nurse? ☐☐ / ☐☐ / ☐☐ **OR** ☐

OR

C Put a cross here if you <u>didn't tell</u> your GP or nurse ☐

3. **Fatigue or tiredness that is unusual for you**

Did you have this?

Yes ☐ **Please complete A then B or C below**

No ☐ **Please go to Question 4**

A When did you first notice this? ☐☐ / ☐☐ / ☐☐ **OR** ☐

B When did you first tell your GP or nurse? ☐☐ / ☐☐ / ☐☐ **OR** ☐

OR

C Put a cross here if you didn't tell your GP or nurse ☐

4. **Feeling different 'in yourself' from usual**

Did you have this?

Yes ☐ **Please complete A then B or C below**

No ☐ **Please go to Question 5**

A When did you first notice this? ☐☐ / ☐☐ / ☐☐ **OR** ☐

B When did you first tell your GP or nurse? ☐☐ / ☐☐ / ☐☐ **OR** ☐

OR

C Put a cross here if you didn't tell your GP or nurse ☐

Please try and describe what the feeling was

If you had any further symptoms which you feel are relevant to your cancer diagnosis, please list them below. If not, please go to Question 7.

Please describe the symptom here and complete A then B or C below

5.

**A**	When did you first notice this?	☐☐ / ☐☐ / ☐☐ **OR** ☐
**B**	When did you first tell your GP or nurse?	☐☐ / ☐☐ / ☐☐ **OR** ☐
	**OR**	
**C**	Put a cross here if you didn't tell your GP or nurse	☐

If you had another symptom you feel is relevant, please write it below, otherwise go to Question 7.

Please describe the symptom here and complete A then B or C below

6.

**A**	When did you first notice this?	☐☐ / ☐☐ / ☐☐ **OR** ☐
**B**	When did you first tell your GP or nurse?	☐☐ / ☐☐ / ☐☐ **OR** ☐
	**OR**	
**C**	Put a cross here if you didn't tell your GP or nurse	☐

If you had further symptoms you would like to tell us about, there is space at the end of the Questionnaire for you to write them.

Did your GP send you for any tests?

Thinking back to the time immediately before you diagnosis, did your GP send you for any of the following tests?

Please cross the appropriate boxes

7. Were you sent for any of these?

	Yes	Not sure	No
Blood test(s)	☐	☐	☐
CT Scan	☐	☐	☐
Ultrasound scan	☐	☐	☐
Barium Enema	☐	☐	☐
Sigmoidoscopy or colonoscopy (Looking at bowel with internal camera)	☐	☐	☐
X-ray	☐	☐	☐

Please go to Question 8

About you

The next questions relate to general information about you. It would help us to know more about the background of the people completing the questionnaire.

8. Which best describes your employment status?

Please cross one box only

- Employed full-time ☐
- Employed part-time ☐
- Self employed full-time ☐
- Self employed part-time ☐
- Unemployed (seeking work) ☐
- Unemployed (not seeking work) ☐
- Retired ☐
- Student ☐
- Permanently sick/disabled ☐
- Temporarily sick/disabled ☐
- Looking after family/home ☐
- Other, please describe

9. What is your highest level of qualification?

Please cross one box only

- Degree (or equivalent) ☐
- Diploma (or equivalent) ☐
- 'A' level ☐
- GCSE / 'O' level ☐
- None ☐
- Other, please specify

10. How would you describe your ethnicity

Please cross one box only

White ☐
or
White British ☐
White Irish ☐
Other White background ☐

Mixed ☐
or
White & Black Carribean ☐
White & Black African ☐
White & Asian ☐
Other mixed background ☐

Black or Black British ☐
or
Carribean ☐
African ☐
Other Black background ☐

Asian or Asian British ☐
or
Indian ☐
Pakistani ☐
Bangladeshi ☐
Other Asian background ☐

Chinese or other ethnic group ☐
or
Chinese ☐
Other ethnic group ☐

11.

Do you live alone?

Yes ☐ **Please go to Question 12**

No ☐ **Please say who you live with below**

Who do you live with?
(e.g. wife, husband, partner, family member)

12. **Are you suffering from, or have you suffered from, any of the following in the past 2 years?**

Please cross any that are applicable to you

- Asthma ☐
- Chronic Obstructive Pulmonary Disease (COPD) ☐
- Other lung disease (e.g. fibrosis, bronchiectatis etc) ☐
- Heart disease ☐
- Anxiety or depression ☐
- Inflammatory bowel disease ☐
- Irritable bowel syndrome ☐
- Peptic ulcer ☐
- Previous cancer ☐
- Diabetes ☐
- Arthritis ☐

13. **About smoking**

Please cross the appropriate statement

- Are you a current smoker? ☐
- Are you an ex-smoker? ☐
- Are you a non-smoker (never smoked)? ☐

14. **Do you think you were more at risk of getting cancer because of your family history?**

Yes ☐
No ☐

15. **The following questions are about how completing the questionnaire made you feel.**

A number of statements which people have used to describe themselves are given below. Read each statement and then cross the most appropriate box to indicate how you feel right now, at the moment. There are no right or wrong answers. Do not spend too much time on any one statement but give the answer which seems to describe your present feeling best.

		Not at all	Somewhat	Moderately	Very much
1.	I feel calm	☐	☐	☐	☐
2.	I am tense	☐	☐	☐	☐
3.	I feel upset	☐	☐	☐	☐
4.	I am relaxed	☐	☐	☐	☐
5.	I feel content	☐	☐	☐	☐
6.	I am worried	☐	☐	☐	☐

Please make sure you have answered all the questions.

If you have any other comments or there is anything else you would like to tell us about when you first noticed your symptoms, please do so using the space below.

If you feel you would like to talk to someone about this questionnaire at a later stage, please speak to the doctor or nurse who is looking after you, or be in touch with the research team, whose contact details are on your patient information sheet

Thank you for completing the questionnaire

Please give it to the research nurse

The next page is for the Researcher to complete

57727

2 4

Researcher - Post-completion Sheet

Researcher initials: ☐☐☐

Date questionnaire completed: Date ☐☐ / ☐☐ / ☐☐ Time ☐☐:☐☐

If not completed, give reason ☐

How long is it since the patient was told of his/her diagnosis? ☐

How long did it take the patient to complete the questionnaire?

- Less than 5 minutes ☐
- Between 5 and 10 minutes ☐
- Between 11 and 15 minutes ☐
- Longer than 15 minutes ☐

For the patient selected to complete the questionnaire on their own

Did the patient ask for any help? ☐ Yes ☐ No

If yes,

please specify what help was requested

____

____

please specify what help was given

____

____

For the patient selected for the researcher-administered questionnaire

Did the patient find any of the questions difficult? ☐ Yes ☐ No

If yes,

please specify which questions and what the difficulty was

____

____

____

____

Was the patient made anxious? ☐ Yes ☐ No

If yes,

please give details

____

____

____

____

Where did the patient complete the questionnaire?
(Please specify whether this was in a separate room alone, in a corner of the clinic, the ward or other

____

Thank you. Please now send the questionnaire and patient information sheet and consent form back to Cardiff University in the freepost envelope provided.

57727

PID - | | 2 | 5 |

Ymddiriedolaeth GIG Gogledd Orllewin Cymru
North West Wales NHS Trust

NHS WALES GIG CYMRU
Ymddiriedolaeth GIG Gogledd Cymru
North Wales NHS Trust

☐ GLAN CLWYD

CID - ☐ GWYNEDD

☐ MAELOR

Confidential Questionnaire

Looking At Your Symptoms Study

This Study has been funded by Cancer Research UK

Cardiff University / Prifysgol Caerdydd
Ysgol Glinigol Gogledd Cymru / North Wales Clinical School
Cancer Research UK
sewtu South East Wales Trials Unit / Uned Ymchwil De-ddwyrain Cymru

Dear Patient

I am very grateful to you for agreeing to help us with this study.

I hope the information you supply will help us give people a diagnosis earlier.

If you feel you would like to talk to someone about this questionnaire at a later stage, please speak to the doctor or nurse who is looking after you, or be in touch with the research team, whose contact details are on your patient information sheet.

I can assure you that anything you tell us will be treated in the utmost confidence.

Yours sincerely

Dr Richard Neal
Senior Clinical Lecturer
Cardiff University
Chief Investigator, Symptoms Study

About your symptoms

These questions are about symptoms you noticed before your diagnosis. We are interested in symptoms which you think are related to your cancer.

Please put a cross in the appropriate box, or write an answer as required.

Looking back...

1. Did you have any symptoms that you feel were due to your cancer before you were diagnosed?

Yes ☐ **Please continue with Question 2**

No ☐ **Please go to Section 2 on Page 5**

2. What was the first symptom that made you think something might be wrong?

☐

Please go to Question 3

The following questions are about when you first noticed a symptom and when you first told your GP or nurse about it.

Please give an exact date if you can. Otherwise give your best estimate. (For example approximately how long ago, the month or season). You wish to refer to your diary or calendar if you have it with you.

Here is an example question:

Decrease in appetite

Did you have this symptom?

Yes ☒ **Please complete A then B or C below**

No ☐ **Please go to Section 2**

A When did you first notice this? d d / m m / y y **OR** Estimate '3 months ago' or 'June'

B When did you first tell your GP or nurse? 2 3 / 1 2 / 0 7 **OR** Estimate

OR

C Put a cross here if you didn't tell your GP or nurse ☐

3. Change in bowel habit

Did you have this symptom?

Yes ☐ **Please complete A then B or C below**

No ☐ **Please go to Question 4**

A When did you first notice this? ☐☐ / ☐☐ / ☐☐ **OR** ☐

B When did you first tell your GP or nurse? ☐☐ / ☐☐ / ☐☐ **OR** ☐

OR

C Put a cross here if you didn't tell your GP or nurse ☐

4. Bleeding from back passage

Did you have this symptom?

Yes ☐ **Please complete A then B or C below**

No ☐ **Please go to Section 2**

A When did you first notice this? ☐☐ / ☐☐ / ☐☐ **OR** ☐

B When did you first tell your GP or nurse? ☐☐ / ☐☐ / ☐☐ **OR** ☐

OR

C Put a cross here if you didn't tell your GP or nurse ☐

Section 2

Please give an <u>exact</u> date if you can. Otherwise give your best <u>estimate</u> (for example approximately how long ago, the month or the season). You may wish to refer to your diary or calendar if you have it with you.

Did you have any of the following?

1. Decrease in appetite

Did you have this?

Yes ☐ **Please complete A then B or C below**

No ☐ **Please go to Question 2**

A When did you <u>first notice</u> this? ☐☐ / ☐☐ / ☐☐ **OR** ☐

B When did you <u>first tell</u> your GP or nurse? ☐☐ / ☐☐ / ☐☐ **OR** ☐

OR

C Put a cross here if you <u>didn't tell</u> your GP or nurse ☐

2. Unexplained weight loss

Did you have this?

Yes ☐ **Please complete A then B or C below**

No ☐ **Please go to Question 3**

A When did you <u>first notice</u> this? ☐☐ / ☐☐ / ☐☐ **OR** ☐

B When did you <u>first tell</u> your GP or nurse? ☐☐ / ☐☐ / ☐☐ **OR** ☐

OR

C Put a cross here if you <u>didn't tell</u> your GP or nurse ☐

3. **Fatigue or tiredness that is unusual for you**

Did you have this?

Yes ☐ **Please complete A then B or C below**

No ☐ **Please go to Question 4**

A When did you <u>first notice</u> this? ☐☐ / ☐☐ / ☐☐ **OR** ☐

B When did you <u>first tell</u> your GP or nurse? ☐☐ / ☐☐ / ☐☐ **OR** ☐

OR

C Put a cross here if you <u>didn't tell</u> your GP or nurse ☐

4. **Feeling different 'in yourself' from usual**

Did you have this?

Yes ☐ **Please complete A then B or C below**

No ☐ **Please go to Question 5**

A When did you <u>first notice</u> this? ☐☐ / ☐☐ / ☐☐ **OR** ☐

B When did you <u>first tell</u> your GP or nurse? ☐☐ / ☐☐ / ☐☐ **OR** ☐

OR

C Put a cross here if you <u>didn't tell</u> your GP or nurse ☐

Please try and describe what the feeling was

If you had any further symptoms which you feel are relevant to your cancer diagnosis, please list them below. If not, please go to Question 7.

Please describe the symptom here and complete A then B or C below

5.

**A**	When did you first notice this?	☐☐ / ☐☐ / ☐☐ **OR** ☐
**B**	When did you first tell your GP or nurse?	☐☐ / ☐☐ / ☐☐ **OR** ☐
	**OR**	
**C**	Put a cross here if you didn't tell your GP or nurse	☐

If you had another symptom you feel is relevant, please write it below, otherwise go to Question 7.

Please describe the symptom here and complete A then B or C below

6.

**A**	When did you first notice this?	☐☐ / ☐☐ / ☐☐ **OR** ☐
**B**	When did you first tell your GP or nurse?	☐☐ / ☐☐ / ☐☐ **OR** ☐
	**OR**	
**C**	Put a cross here if you didn't tell your GP or nurse	☐

If you had further symptoms you would like to tell us about, there is space at the end of the Questionnaire for you to write them.

Did your GP send you for any tests?

Thinking back to the time immediately before you diagnosis, did your GP send you for any of the following tests?

Please cross the appropriate boxes

7. Were you sent for any of these?

	Yes	Not sure	No
Blood test(s)	☐	☐	☐
CT Scan	☐	☐	☐
Ultrasound scan	☐	☐	☐
Barium Enema	☐	☐	☐
Sigmoidoscopy or colonoscopy (Looking at bowel with internal camera)	☐	☐	☐
X-ray	☐	☐	☐

Please go to Question 8

About you

The next questions relate to general information about you. It would help us to know more about the background of the people completing the questionnaire.

8. Which best describes your employment status?

Please cross one box only

- Employed full-time ☐
- Employed part-time ☐
- Self employed full-time ☐
- Self employed part-time ☐
- Unemployed (seeking work) ☐
- Unemployed (not seeking work) ☐
- Retired ☐
- Student ☐
- Permanently sick/disabled ☐
- Temporarily sick/disabled ☐
- Looking after family/home ☐

Other, please describe

9. What is your highest level of qualification?

Please cross one box only

- Degree (or equivalent) ☐
- Diploma (or equivalent) ☐
- 'A' level ☐
- GCSE / 'O' level ☐
- None ☐

Other, please specify

10. **How would you describe your ethnicity**

Please cross one box only

White ☐
or
- White British ☐
- White Irish ☐
- Other White background ☐

Mixed ☐
or
- White & Black Carribean ☐
- White & Black African ☐
- White & Asian ☐
- Other mixed background ☐

Black or Black British ☐
or
- Carribean ☐
- African ☐
- Other Black background ☐

Asian or Asian British ☐
or
- Indian ☐
- Pakistani ☐
- Bangladeshi ☐
- Other Asian background ☐

Chinese or other ethnic group ☐
or
- Chinese ☐
- Other ethnic group ☐

11.

Do you live alone?

Yes ☐ **Please go to Question 12**

No ☐ **Please say who you live with below**

Who do you live with?
(e.g. wife, husband, partner, family member)

12. **Are you suffering from, or have you suffered from, any of the following in the past 2 years?**

Please cross any that are applicable to you

- Asthma ☐
- Chronic Obstructive Pulmonary Disease (COPD) ☐
- Other lung disease (e.g. fibrosis, bronchiectatis etc) ☐
- Heart disease ☐
- Anxiety or depression ☐
- Inflammatory bowel disease ☐
- Irritable bowel syndrome ☐
- Peptic ulcer ☐
- Previous cancer ☐
- Diabetes ☐
- Arthritis ☐

13. **About smoking**

Please cross the appropriate statement

- Are you a current smoker? ☐
- Are you an ex-smoker? ☐
- Are you a non-smoker (never smoked)? ☐

14. **Do you think you were more at risk of getting cancer because of your family history?**

Yes ☐

No ☐

15. The following questions are about how completing the questionnaire made you feel.

A number of statements which people have used to describe themselves are given below. Read each statement and then cross the most appropriate box to indicate how you feel <u>right now, at the moment.</u> There are no right or wrong answers. Do not spend too much time on any one statement but give the answer which seems to describe your present feeling best.

		Not at all	Somewhat	Moderately	Very much
1.	I feel calm	☐	☐	☐	☐
2.	I am tense	☐	☐	☐	☐
3.	I feel upset	☐	☐	☐	☐
4.	I am relaxed	☐	☐	☐	☐
5.	I feel content	☐	☐	☐	☐
6.	I am worried	☐	☐	☐	☐

Please make sure you have answered all the questions.

If you have any other comments or there is anything else you would like to tell us about when you first noticed your symptoms, please do so using the space below.

If you feel you would like to talk to someone about this questionnaire at a later stage, please speak to the doctor or nurse who is looking after you, or be in touch with the research team, whose contact details are on your patient information sheet

Thank you for completing the questionnaire

Please give it to the research nurse

The next page is for the Researcher to complete

Researcher - Post-completion Sheet

Researcher initials: ☐☐☐

Date questionnaire completed: Date ☐☐ / ☐☐ / ☐☐ Time ☐☐:☐☐

If not completed, give reason ☐

How long is it since the patient was told of his/her diagnosis? ☐

How long did it take the patient to complete the questionnaire?

- Less than 5 minutes ☐
- Between 5 and 10 minutes ☐
- Between 11 and 15 minutes ☐
- Longer than 15 minutes ☐

For the patient selected to complete the questionnaire on their own

Did the patient ask for any help? ☐ Yes ☐ No

If yes,

please specify what help was requested

____

____

please specify what help was given

____

____

For the patient selected for the researcher-administered questionnaire

Did the patient find any of the questions difficult? ☐ Yes ☐ No

If yes,

please specify which questions and what the difficulty was

____

____

____

____

Was the patient made anxious? ☐ Yes ☐ No

If yes,

please give details

____

____

____

____

Where did the patient complete the questionnaire?

(Please specify whether this was in a separate room alone, in a corner of the clinic, the ward or other

____

Thank you. Please now send the questionnaire and patient information sheet and consent form back to Cardiff University in the freepost envelope provided.

57727

PID - 2 6

Ymddiriedolaeth GIG Gogledd Orllewin Cymru
North West Wales NHS Trust

NHS WALES GIG CYMRU
Ymddiriedolaeth GIG Gogledd Cymru
North Wales NHS Trust

CID - ☐ GLAN CLWYD
☐ GWYNEDD
☐ MAELOR

Confidential Questionnaire

Looking At Your Symptoms Study

This Study has been funded by Cancer Research UK

Cardiff University Prifysgol Caerdydd
Ysgol Glinigol Gogledd Cymru North Wales Clinical School
Cancer Research UK
sewtu South East Wales Trials Unit Uned Ymchwil De-ddwyrain Cymru

Dear Patient

I am very grateful to you for agreeing to help us with this study.

I hope the information you supply will help us give people a diagnosis earlier.

If you feel you would like to talk to someone about this questionnaire at a later stage, please speak to the doctor or nurse who is looking after you, or be in touch with the research team, whose contact details are on your patient information sheet.

I can assure you that anything you tell us will be treated in the utmost confidence.

Yours sincerely

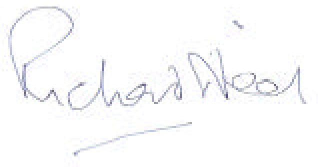

Dr Richard Neal
Senior Clinical Lecturer
Cardiff University
Chief Investigator, Symptoms Study

About your symptoms

These questions are about symptoms you noticed before your diagnosis. We are interested in symptoms which you think are related to your cancer.

Please put a cross in the appropriate box, or write an answer as required.

Looking back...

1. Did you have any symptoms that you feel were due to your cancer before you were diagnosed?

Yes ☐ **Please continue with Question 2**

No ☐ **Please go to Section 2 on Page 5**

2. What was the first symptom that made you think something might be wrong?

[ ]

Please go to Question 3

The following questions are about when you first noticed a symptom and when you first told your GP or nurse about it.

Please give an exact date if you can. Otherwise give your best estimate. (For example approximately how long ago, the month or season). You wish to refer to your diary or calendar if you have it with you.

Here is an example question:

Decrease in appetite

Did you have this symptom?

Yes ☒ **Please complete A then B or C below**

No ☐ **Please go to Section 2**

A When did you first notice this? d d / m m / y y **OR** Estimate '3 months ago' or 'June'

B When did you first tell your GP or nurse? 2 3 / 1 2 / 0 7 **OR** Estimate

OR

C Put a cross here if you didn't tell your GP or nurse ☐

3. Change in bowel habit

Did you have this symptom?

Yes ☐ **Please complete A then B or C below**

No ☐ **Please go to Question 4**

A When did you first notice this? ☐☐ / ☐☐ / ☐☐ **OR** ☐

B When did you first tell your GP or nurse? ☐☐ / ☐☐ / ☐☐ **OR** ☐

OR

C Put a cross here if you didn't tell your GP or nurse ☐

4. Bleeding from back passage

Did you have this symptom?

Yes ☐ **Please complete A then B or C below**

No ☐ **Please go to Section 2**

A When did you first notice this? ☐☐ / ☐☐ / ☐☐ **OR** ☐

B When did you first tell your GP or nurse? ☐☐ / ☐☐ / ☐☐ **OR** ☐

OR

C Put a cross here if you didn't tell your GP or nurse ☐

Section 2

Please give an exact date if you can. Otherwise give your best estimate (for example approximately how long ago, the month or the season). You may wish to refer to your diary or calendar if you have it with you.

Did you have any of the following?

1. Decrease in appetite

Did you have this?

Yes ☐ **Please complete A then B or C below**

No ☐ **Please go to Question 2**

A When did you first notice this? ☐☐ / ☐☐ / ☐☐ **OR** ☐

B When did you first tell your GP or nurse? ☐☐ / ☐☐ / ☐☐ **OR** ☐

OR

C Put a cross here if you didn't tell your GP or nurse ☐

2. Unexplained weight loss

Did you have this?

Yes ☐ **Please complete A then B or C below**

No ☐ **Please go to Question 3**

A When did you first notice this? ☐☐ / ☐☐ / ☐☐ **OR** ☐

B When did you first tell your GP or nurse? ☐☐ / ☐☐ / ☐☐ **OR** ☐

OR

C Put a cross here if you didn't tell your GP or nurse ☐

3. **Fatigue or tiredness that is unusual for you**

Did you have this?

Yes ☐ **Please complete A then B or C below**

No ☐ **Please go to Question 4**

A When did you <u>first notice</u> this? ☐☐ / ☐☐ / ☐☐ **OR** ☐

B When did you <u>first tell</u> your GP or nurse? ☐☐ / ☐☐ / ☐☐ **OR** ☐

OR

C Put a cross here if you <u>didn't tell</u> your GP or nurse ☐

4. **Feeling different 'in yourself' from usual**

Did you have this?

Yes ☐ **Please complete A then B or C below**

No ☐ **Please go to Question 5**

A When did you <u>first notice</u> this? ☐☐ / ☐☐ / ☐☐ **OR** ☐

B When did you <u>first tell</u> your GP or nurse? ☐☐ / ☐☐ / ☐☐ **OR** ☐

OR

C Put a cross here if you <u>didn't tell</u> your GP or nurse ☐

Please try and describe what the feeling was

If you had any further symptoms which you feel are relevant to your cancer diagnosis, please list them below. If not, please go to Question 7.

Please describe the symptom here and complete A then B or C below

5.

**A** When did you <u>first notice</u> this?	__ __ / __ __ / __ __ **OR**
**B** When did you <u>first tell</u> your GP or nurse?	__ __ / __ __ / __ __ **OR**
**OR**	
**C** Put a cross here if you <u>didn't tell</u> your GP or nurse	☐

If you had another symptom you feel is relevant, please write it below, otherwise go to Question 7.

Please describe the symptom here and complete A then B or C below

6.

**A** When did you <u>first notice</u> this?	__ __ / __ __ / __ __ **OR**
**B** When did you <u>first tell</u> your GP or nurse?	__ __ / __ __ / __ __ **OR**
**OR**	
**C** Put a cross here if you <u>didn't tell</u> your GP or nurse	☐

If you had further symptoms you would like to tell us about, there is space at the end of the Questionnaire for you to write them.

Did your GP send you for any tests?

Thinking back to the time immediately before you diagnosis, did your GP send you for any of the following tests?

Please cross the appropriate boxes

7. Were you sent for any of these?

	Yes	Not sure	No
Blood test(s)	☐	☐	☐
CT Scan	☐	☐	☐
Ultrasound scan	☐	☐	☐
Barium Enema	☐	☐	☐
Sigmoidoscopy or colonoscopy (Looking at bowel with internal camera)	☐	☐	☐
X-ray	☐	☐	☐

Please go to Question 8

About you

The next questions relate to general information about you. It would help us to know more about the background of the people completing the questionnaire.

8. Which best describes your employment status?

Please cross one box only

- Employed full-time ☐
- Employed part-time ☐
- Self employed full-time ☐
- Self employed part-time ☐
- Unemployed (seeking work) ☐
- Unemployed (not seeking work) ☐
- Retired ☐
- Student ☐
- Permanently sick/disabled ☐
- Temporarily sick/disabled ☐
- Looking after family/home ☐
- Other, please describe ☐

9. What is your highest level of qualification?

Please cross one box only

- Degree (or equivalent) ☐
- Diploma (or equivalent) ☐
- 'A' level ☐
- GCSE / 'O' level ☐
- None ☐
- Other, please specify ☐

10. **How would you describe your ethnicity**

Please cross one box only

White ☐
or
White British ☐
White Irish ☐
Other White background ☐

Mixed ☐
or
White & Black Carribean ☐
White & Black African ☐
White & Asian ☐
Other mixed background ☐

Black or Black British ☐
or
Carribean ☐
African ☐
Other Black background ☐

Asian or Asian British ☐
or
Indian ☐
Pakistani ☐
Bangladeshi ☐
Other Asian background ☐

Chinese or other ethnic group ☐
or
Chinese ☐
Other ethnic group ☐

11.

Do you live alone?

Yes ☐ **Please go to Question 12**

No ☐ **Please say who you live with below**

Who do you live with?
(e.g. wife, husband, partner, family member)

12. **Are you suffering from, or have you suffered from, any of the following in the past 2 years?**

Please cross any that are applicable to you

- Asthma ☐
- Chronic Obstructive Pulmonary Disease (COPD) ☐
- Other lung disease (e.g. fibrosis, bronchiectatis etc) ☐
- Heart disease ☐
- Anxiety or depression ☐
- Inflammatory bowel disease ☐
- Irritable bowel syndrome ☐
- Peptic ulcer ☐
- Previous cancer ☐
- Diabetes ☐
- Arthritis ☐

13. **About smoking**

Please cross the appropriate statement

- Are you a current smoker? ☐
- Are you an ex-smoker? ☐
- Are you a non-smoker (never smoked)? ☐

14. **Do you think you were more at risk of getting cancer because of your family history?**

Yes ☐

No ☐

15. The following questions are about how completing the questionnaire made you feel.

A number of statements which people have used to describe themselves are given below. Read each statement and then cross the most appropriate box to indicate how you feel <u>right now, at the moment.</u> There are no right or wrong answers. Do not spend too much time on any one statement but give the answer which seems to describe your present feeling best.

		Not at all	Somewhat	Moderately	Very much
1.	I feel calm	☐	☐	☐	☐
2.	I am tense	☐	☐	☐	☐
3.	I feel upset	☐	☐	☐	☐
4.	I am relaxed	☐	☐	☐	☐
5.	I feel content	☐	☐	☐	☐
6.	I am worried	☐	☐	☐	☐

Please make sure you have answered all the questions.

If you have any other comments or there is anything else you would like to tell us about when you first noticed your symptoms, please do so using the space below.

If you feel you would like to talk to someone about this questionnaire at a later stage, please speak to the doctor or nurse who is looking after you, or be in touch with the research team, whose contact details are on your patient information sheet

Thank you for completing the questionnaire

Please give it to the research nurse

The next page is for the Researcher to complete

57727

2 6

Researcher - Post-completion Sheet

Researcher initials: ☐☐☐

Date questionnaire completed: Date ☐☐ / ☐☐ / ☐☐ Time ☐☐:☐☐

If not completed, give reason ☐

How long is it since the patient was told of his/her diagnosis? ☐

How long did it take the patient to complete the questionnaire?

- Less than 5 minutes ☐
- Between 5 and 10 minutes ☐
- Between 11 and 15 minutes ☐
- Longer than 15 minutes ☐

For the patient selected to complete the questionnaire on their own

Did the patient ask for any help? ☐ Yes ☐ No

If yes,

please specify what help was requested

____

____

please specify what help was given

____

____

For the patient selected for the researcher-administered questionnaire

Did the patient find any of the questions difficult? ☐ Yes ☐ No

If yes,

please specify which questions and what the difficulty was

____

____

____

____

Was the patient made anxious? ☐ Yes ☐ No

If yes,

please give details

____

____

____

____

Where did the patient complete the questionnaire?
(Please specify whether this was in a separate room alone, in a corner of the clinic, the ward or other

____

Thank you. Please now send the questionnaire and patient information sheet and consent form back to Cardiff University in the freepost envelope provided.

57727

PID - 2 7

Ymddiriedolaeth GIG Gogledd Orllewin Cymru
North West Wales NHS Trust

NHS WALES GIG CYMRU
Ymddiriedolaeth GIG Gogledd Cymru
North Wales NHS Trust

CID - ☐ GLAN CLWYD

☐ GWYNEDD

☐ MAELOR

Confidential Questionnaire

Looking At Your Symptoms Study

This Study has been funded by Cancer Research UK

Cardiff University / Prifysgol Caerdydd

Ysgol Glinigol Gogledd Cymru / North Wales Clinical School

Cancer Research UK

sewtu — South East Wales Trials Unit / Uned Ymchwil De-ddwyrain Cymru

Dear Patient

I am very grateful to you for agreeing to help us with this study.

I hope the information you supply will help us give people a diagnosis earlier.

If you feel you would like to talk to someone about this questionnaire at a later stage, please speak to the doctor or nurse who is looking after you, or be in touch with the research team, whose contact details are on your patient information sheet.

I can assure you that anything you tell us will be treated in the utmost confidence.

Yours sincerely

Dr Richard Neal
Senior Clinical Lecturer
Cardiff University
Chief Investigator, Symptoms Study

About your symptoms

These questions are about symptoms you noticed before your diagnosis. We are interested in symptoms which you think are related to your cancer.

Please put a cross in the appropriate box, or write an answer as required.

Looking back...

1. Did you have any symptoms that you feel were due to your cancer before you were diagnosed?

Yes ☐ **Please continue with Question 2**

No ☐ **Please go to Section 2 on Page 5**

2. What was the first symptom that made you think something might be wrong?

☐

Please go to Question 3

The following questions are about when you first noticed a symptom and when you first told your GP or nurse about it.

Please give an exact date if you can. Otherwise give your best estimate. (For example approximately how long ago, the month or season). You wish to refer to your diary or calendar if you have it with you.

Here is an example question:

Decrease in appetite

Did you have this symptom?

Yes ☒ **Please complete A then B or C below**

No ☐ **Please go to Section 2**

A When did you first notice this? d d / m m / y y **OR** Estimate '3 months ago' or 'June'

B When did you first tell your GP or nurse? 2 3 / 1 2 / 0 7 **OR** Estimate

OR

C Put a cross here if you didn't tell your GP or nurse ☐

3. Change in bowel habit

Did you have this symptom?

Yes ☐ **Please complete A then B or C below**

No ☐ **Please go to Question 4**

A When did you first notice this? ☐☐ / ☐☐ / ☐☐ **OR** ☐

B When did you first tell your GP or nurse? ☐☐ / ☐☐ / ☐☐ **OR** ☐

OR

C Put a cross here if you didn't tell your GP or nurse ☐

4. Bleeding from back passage

Did you have this symptom?

Yes ☐ **Please complete A then B or C below**

No ☐ **Please go to Section 2**

A When did you first notice this? ☐☐ / ☐☐ / ☐☐ **OR** ☐

B When did you first tell your GP or nurse? ☐☐ / ☐☐ / ☐☐ **OR** ☐

OR

C Put a cross here if you didn't tell your GP or nurse ☐

Section 2

Please give an <u>exact</u> date if you can. Otherwise give your best <u>estimate</u> (for example approximately how long ago, the month or the season). You may wish to refer to your diary or calendar if you have it with you.

Did you have any of the following?

1. Decrease in appetite

Did you have this?

Yes ☐ **Please complete A then B or C below**

No ☐ **Please go to Question 2**

A When did you first notice this? ☐☐ / ☐☐ / ☐☐ **OR** ☐

B When did you first tell your GP or nurse? ☐☐ / ☐☐ / ☐☐ **OR** ☐

OR

C Put a cross here if you didn't tell your GP or nurse ☐

2. Unexplained weight loss

Did you have this?

Yes ☐ **Please complete A then B or C below**

No ☐ **Please go to Question 3**

A When did you first notice this? ☐☐ / ☐☐ / ☐☐ **OR** ☐

B When did you first tell your GP or nurse? ☐☐ / ☐☐ / ☐☐ **OR** ☐

OR

C Put a cross here if you didn't tell your GP or nurse ☐

3. **Fatigue or tiredness that is unusual for you**

Did you have this?

Yes ☐ **Please complete A then B or C below**

No ☐ **Please go to Question 4**

A When did you first notice this? ☐☐ / ☐☐ / ☐☐ **OR** ☐

B When did you first tell your GP or nurse? ☐☐ / ☐☐ / ☐☐ **OR** ☐

OR

C Put a cross here if you didn't tell your GP or nurse ☐

4. **Feeling different 'in yourself' from usual**

Did you have this?

Yes ☐ **Please complete A then B or C below**

No ☐ **Please go to Question 5**

A When did you first notice this? ☐☐ / ☐☐ / ☐☐ **OR** ☐

B When did you first tell your GP or nurse? ☐☐ / ☐☐ / ☐☐ **OR** ☐

OR

C Put a cross here if you didn't tell your GP or nurse ☐

Please try and describe what the feeling was

If you had any further symptoms which you feel are relevant to your cancer diagnosis, please list them below. If not, please go to Question 7.

Please describe the symptom here and complete A then B or C below

5.

**A**	When did you first notice this?	__ __ / __ __ / __ __ **OR** ______
**B**	When did you first tell your GP or nurse?	__ __ / __ __ / __ __ **OR** ______
	**OR**	
**C**	Put a cross here if you didn't tell your GP or nurse	☐

If you had another symptom you feel is relevant, please write it below, otherwise go to Question 7.

Please describe the symptom here and complete A then B or C below

6.

**A**	When did you first notice this?	__ __ / __ __ / __ __ **OR** ______
**B**	When did you first tell your GP or nurse?	__ __ / __ __ / __ __ **OR** ______
	**OR**	
**C**	Put a cross here if you didn't tell your GP or nurse	☐

If you had further symptoms you would like to tell us about, there is space at the end of the Questionnaire for you to write them.

Did your GP send you for any tests?

Thinking back to the time immediately before you diagnosis, did your GP send you for any of the following tests?

Please cross the appropriate boxes

7. Were you sent for any of these?

	Yes	Not sure	No
Blood test(s)	☐	☐	☐
CT Scan	☐	☐	☐
Ultrasound scan	☐	☐	☐
Barium Enema	☐	☐	☐
Sigmoidoscopy or colonoscopy (Looking at bowel with internal camera)	☐	☐	☐
X-ray	☐	☐	☐

Please go to Question 8

About you

The next questions relate to general information about you. It would help us to know more about the background of the people completing the questionnaire.

8. Which best describes your employment status?

Please cross one box only

- Employed full-time ☐
- Employed part-time ☐
- Self employed full-time ☐
- Self employed part-time ☐
- Unemployed (seeking work) ☐
- Unemployed (not seeking work) ☐
- Retired ☐
- Student ☐
- Permanently sick/disabled ☐
- Temporarily sick/disabled ☐
- Looking after family/home ☐
- Other, please describe ________

9. What is your highest level of qualification?

Please cross one box only

- Degree (or equivalent) ☐
- Diploma (or equivalent) ☐
- 'A' level ☐
- GCSE / 'O' level ☐
- None ☐
- Other, please specify ________

10. How would you describe your ethnicity

Please cross one box only

White ☐
or
- White British ☐
- White Irish ☐
- Other White background ☐

Mixed ☐
or
- White & Black Carribean ☐
- White & Black African ☐
- White & Asian ☐
- Other mixed background ☐

Black or Black British ☐
or
- Carribean ☐
- African ☐
- Other Black background ☐

Asian or Asian British ☐
or
- Indian ☐
- Pakistani ☐
- Bangladeshi ☐
- Other Asian background ☐

Chinese or other ethnic group ☐
or
- Chinese ☐
- Other ethnic group ☐

11.

Do you live alone?

Yes ☐ **Please go to Question 12**

No ☐ **Please say who you live with below**

Who do you live with?
(e.g. wife, husband, partner, family member)

☐

12. **Are you suffering from, or have you suffered from, any of the following in the past 2 years?**

Please cross any that are applicable to you

- Asthma ☐
- Chronic Obstructive Pulmonary Disease (COPD) ☐
- Other lung disease (e.g. fibrosis, bronchiectatis etc) ☐
- Heart disease ☐
- Anxiety or depression ☐
- Inflammatory bowel disease ☐
- Irritable bowel syndrome ☐
- Peptic ulcer ☐
- Previous cancer ☐
- Diabetes ☐
- Arthritis ☐

13. **About smoking**

Please cross the appropriate statement

- Are you a current smoker? ☐
- Are you an ex-smoker? ☐
- Are you a non-smoker (never smoked)? ☐

14. **Do you think you were more at risk of getting cancer because of your family history?**

Yes ☐
No ☐

15. The following questions are about how completing the questionnaire made you feel.

A number of statements which people have used to describe themselves are given below. Read each statement and then cross the most appropriate box to indicate how you feel right now, at the moment. There are no right or wrong answers. Do not spend too much time on any one statement but give the answer which seems to describe your present feeling best.

		Not at all	Somewhat	Moderately	Very much
1.	I feel calm	☐	☐	☐	☐
2.	I am tense	☐	☐	☐	☐
3.	I feel upset	☐	☐	☐	☐
4.	I am relaxed	☐	☐	☐	☐
5.	I feel content	☐	☐	☐	☐
6.	I am worried	☐	☐	☐	☐

Please make sure you have answered all the questions.

If you have any other comments or there is anything else you would like to tell us about when you first noticed your symptoms, please do so using the space below.

If you feel you would like to talk to someone about this questionnaire at a later stage, please speak to the doctor or nurse who is looking after you, or be in touch with the research team, whose contact details are on your patient information sheet

Thank you for completing the questionnaire

Please give it to the research nurse

The next page is for the Researcher to complete

Researcher - Post-completion Sheet

Researcher initials: ☐☐☐

Date questionnaire completed: Date ☐☐ / ☐☐ / ☐☐ Time ☐☐:☐☐

If not completed, give reason ☐

How long is it since the patient was told of his/her diagnosis? ☐

How long did it take the patient to complete the questionnaire?

- Less than 5 minutes ☐
- Between 5 and 10 minutes ☐
- Between 11 and 15 minutes ☐
- Longer than 15 minutes ☐

For the patient selected to complete the questionnaire on their own

Did the patient ask for any help? ☐ Yes ☐ No

If yes,

please specify what help was requested

____

____

please specify what help was given

____

____

For the patient selected for the researcher-administered questionnaire

Did the patient find any of the questions difficult? ☐ Yes ☐ No

If yes,

please specify which questions and what the difficulty was

____

____

____

____

Was the patient made anxious? ☐ Yes ☐ No

If yes,

please give details

____

____

____

____

Where did the patient complete the questionnaire?

(Please specify whether this was in a separate room alone, in a corner of the clinic, the ward or other

____

Thank you. Please now send the questionnaire and patient information sheet and consent form back to Cardiff University in the freepost envelope provided.

57727

PID - | 2 | 8

Ymddiriedolaeth GIG Gogledd Orllewin Cymru
North West Wales NHS Trust

NHS WALES GIG CYMRU
Ymddiriedolaeth GIG Gogledd Cymru
North Wales NHS Trust

CID -
☐ GLAN CLWYD
☐ GWYNEDD
☐ MAELOR

Confidential Questionnaire

Looking At Your Symptoms Study

This Study has been funded by Cancer Research UK

Cardiff University / Prifysgol Caerdydd
Ysgol Glinigol Gogledd Cymru / North Wales Clinical School
Cancer Research UK
sewtu — South East Wales Trials Unit / Uned Ymchwil De-ddwyrain Cymru

Dear Patient

I am very grateful to you for agreeing to help us with this study.

I hope the information you supply will help us give people a diagnosis earlier.

If you feel you would like to talk to someone about this questionnaire at a later stage, please speak to the doctor or nurse who is looking after you, or be in touch with the research team, whose contact details are on your patient information sheet.

I can assure you that anything you tell us will be treated in the utmost confidence.

Yours sincerely

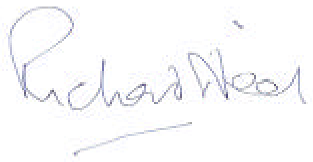

Dr Richard Neal
Senior Clinical Lecturer
Cardiff University
Chief Investigator, Symptoms Study

About your symptoms

These questions are about symptoms you noticed before your diagnosis. We are interested in symptoms which you think are related to your cancer.

Please put a cross in the appropriate box, or write an answer as required.

Looking back...

1. Did you have any symptoms that you feel were due to your cancer before you were diagnosed?

Yes ☐ **Please continue with Question 2**

No ☐ **Please go to Section 2 on Page 5**

2. What was the first symptom that made you think something might be wrong?

☐

Please go to Question 3

The following questions are about when you first noticed a symptom and when you first told your GP or nurse about it.

Please give an exact date if you can. Otherwise give your best estimate. (For example approximately how long ago, the month or season). You wish to refer to your diary or calendar if you have it with you.

Here is an example question:

Decrease in appetite

Did you have this symptom?

Yes ☒ **Please complete A then B or C below**

No ☐ **Please go to Section 2**

A When did you first notice this? d d / m m / y y **OR** Estimate '3 months ago' or 'June'

B When did you first tell your GP or nurse? 2 3 / 1 2 / 0 7 **OR** Estimate

OR

C Put a cross here if you didn't tell your GP or nurse ☐

3. Change in bowel habit

Did you have this symptom?

Yes ☐ **Please complete A then B or C below**

No ☐ **Please go to Question 4**

A When did you first notice this? ☐☐ / ☐☐ / ☐☐ **OR** ☐

B When did you first tell your GP or nurse? ☐☐ / ☐☐ / ☐☐ **OR** ☐

OR

C Put a cross here if you didn't tell your GP or nurse ☐

4. Bleeding from back passage

Did you have this symptom?

Yes ☐ **Please complete A then B or C below**

No ☐ **Please go to Section 2**

A When did you first notice this? ☐☐ / ☐☐ / ☐☐ **OR** ☐

B When did you first tell your GP or nurse? ☐☐ / ☐☐ / ☐☐ **OR** ☐

OR

C Put a cross here if you didn't tell your GP or nurse ☐

Section 2

Please give an exact date if you can. Otherwise give your best estimate (for example approximately how long ago, the month or the season). You may wish to refer to your diary or calendar if you have it with you.

Did you have any of the following?

1. Decrease in appetite

Did you have this?

Yes ☐ **Please complete A then B or C below**

No ☐ **Please go to Question 2**

A When did you first notice this? ☐☐ / ☐☐ / ☐☐ **OR** ☐

B When did you first tell your GP or nurse? ☐☐ / ☐☐ / ☐☐ **OR** ☐

OR

C Put a cross here if you didn't tell your GP or nurse ☐

2. Unexplained weight loss

Did you have this?

Yes ☐ **Please complete A then B or C below**

No ☐ **Please go to Question 3**

A When did you first notice this? ☐☐ / ☐☐ / ☐☐ **OR** ☐

B When did you first tell your GP or nurse? ☐☐ / ☐☐ / ☐☐ **OR** ☐

OR

C Put a cross here if you didn't tell your GP or nurse ☐

3. **Fatigue or tiredness that is unusual for you**

Did you have this?

Yes ☐ **Please complete A then B or C below**

No ☐ **Please go to Question 4**

A When did you first notice this? __ __ / __ __ / __ __ **OR** ______

B When did you first tell your GP or nurse? __ __ / __ __ / __ __ **OR** ______

OR

C Put a cross here if you didn't tell your GP or nurse ☐

4. **Feeling different 'in yourself' from usual**

Did you have this?

Yes ☐ **Please complete A then B or C below**

No ☐ **Please go to Question 5**

A When did you first notice this? __ __ / __ __ / __ __ **OR** ______

B When did you first tell your GP or nurse? __ __ / __ __ / __ __ **OR** ______

OR

C Put a cross here if you didn't tell your GP or nurse ☐

Please try and describe what the feeling was

If you had any further symptoms which you feel are relevant to your cancer diagnosis, please list them below. If not, please go to Question 7.

Please describe the symptom here and complete A then B or C below

5.

**A**	When did you first notice this?	__/__/__ **OR**
**B**	When did you first tell your GP or nurse?	__/__/__ **OR**
	**OR**	
**C**	Put a cross here if you didn't tell your GP or nurse	☐

If you had another symptom you feel is relevant, please write it below, otherwise go to Question 7.

Please describe the symptom here and complete A then B or C below

6.

**A**	When did you first notice this?	__/__/__ **OR**
**B**	When did you first tell your GP or nurse?	__/__/__ **OR**
	**OR**	
**C**	Put a cross here if you didn't tell your GP or nurse	☐

If you had further symptoms you would like to tell us about, there is space at the end of the Questionnaire for you to write them.

Did your GP send you for any tests?

Thinking back to the time immediately before you diagnosis, did your GP send you for any of the following tests?

Please cross the appropriate boxes

7. Were you sent for any of these?

	Yes	Not sure	No
Blood test(s)	☐	☐	☐
CT Scan	☐	☐	☐
Ultrasound scan	☐	☐	☐
Barium Enema	☐	☐	☐
Sigmoidoscopy or colonoscopy (Looking at bowel with internal camera)	☐	☐	☐
X-ray	☐	☐	☐

Please go to Question 8

About you

The next questions relate to general information about you. It would help us to know more about the background of the people completing the questionnaire.

8. Which best describes your employment status?

Please cross one box only

- Employed full-time ☐
- Employed part-time ☐
- Self employed full-time ☐
- Self employed part-time ☐
- Unemployed (seeking work) ☐
- Unemployed (not seeking work) ☐
- Retired ☐
- Student ☐
- Permanently sick/disabled ☐
- Temporarily sick/disabled ☐
- Looking after family/home ☐
- Other, please describe ____________

9. What is your highest level of qualification?

Please cross one box only

- Degree (or equivalent) ☐
- Diploma (or equivalent) ☐
- 'A' level ☐
- GCSE / 'O' level ☐
- None ☐
- Other, please specify ____________

10. How would you describe your ethnicity

Please cross one box only

White ☐
or
- White British ☐
- White Irish ☐
- Other White background ☐

Mixed ☐
or
- White & Black Carribean ☐
- White & Black African ☐
- White & Asian ☐
- Other mixed background ☐

Black or Black British ☐
or
- Carribean ☐
- African ☐
- Other Black background ☐

Asian or Asian British ☐
or
- Indian ☐
- Pakistani ☐
- Bangladeshi ☐
- Other Asian background ☐

Chinese or other ethnic group ☐
or
- Chinese ☐
- Other ethnic group ☐

11.

Do you live alone?

Yes ☐ **Please go to Question 12**

No ☐ **Please say who you live with below**

Who do you live with?
(e.g. wife, husband, partner, family member)

12. **Are you suffering from, or have you suffered from, any of the following in the past 2 years?**

Please cross any that are applicable to you

- Asthma ☐
- Chronic Obstructive Pulmonary Disease (COPD) ☐
- Other lung disease (e.g. fibrosis, bronchiectatis etc) ☐
- Heart disease ☐
- Anxiety or depression ☐
- Inflammatory bowel disease ☐
- Irritable bowel syndrome ☐
- Peptic ulcer ☐
- Previous cancer ☐
- Diabetes ☐
- Arthritis ☐

13. **About smoking**

Please cross the appropriate statement

- Are you a current smoker? ☐
- Are you an ex-smoker? ☐
- Are you a non-smoker (never smoked)? ☐

14. **Do you think you were more at risk of getting cancer because of your family history?**

Yes ☐
No ☐

15. The following questions are about how completing the questionnaire made you feel.

A number of statements which people have used to describe themselves are given below. Read each statement and then cross the most appropriate box to indicate how you feel right now, at the moment. There are no right or wrong answers. Do not spend too much time on any one statement but give the answer which seems to describe your present feeling best.

		Not at all	Somewhat	Moderately	Very much
1.	I feel calm	☐	☐	☐	☐
2.	I am tense	☐	☐	☐	☐
3.	I feel upset	☐	☐	☐	☐
4.	I am relaxed	☐	☐	☐	☐
5.	I feel content	☐	☐	☐	☐
6.	I am worried	☐	☐	☐	☐

Please make sure you have answered all the questions.

If you have any other comments or there is anything else you would like to tell us about when you first noticed your symptoms, please do so using the space below.

If you feel you would like to talk to someone about this questionnaire at a later stage, please speak to the doctor or nurse who is looking after you, or be in touch with the research team, whose contact details are on your patient information sheet

Thank you for completing the questionnaire

Please give it to the research nurse

The next page is for the Researcher to complete

Researcher - Post-completion Sheet

Researcher initials: ☐☐☐

Date questionnaire completed: Date ☐☐ / ☐☐ / ☐☐ Time ☐☐:☐☐

If not completed, give reason ☐

How long is it since the patient was told of his/her diagnosis? ☐

How long did it take the patient to complete the questionnaire?

- Less than 5 minutes ☐
- Between 5 and 10 minutes ☐
- Between 11 and 15 minutes ☐
- Longer than 15 minutes ☐

For the patient selected to complete the questionnaire on their own

Did the patient ask for any help? ☐ Yes ☐ No

If yes,

please specify what help was requested

____

____

please specify what help was given

____

____

For the patient selected for the researcher-administered questionnaire

Did the patient find any of the questions difficult? ☐ Yes ☐ No

If yes,

please specify which questions and what the difficulty was

____

____

____

____

Was the patient made anxious? ☐ Yes ☐ No

If yes,

please give details

____

____

____

____

Where did the patient complete the questionnaire?

(Please specify whether this was in a separate room alone, in a corner of the clinic, the ward or other

____

Thank you. Please now send the questionnaire and patient information sheet and consent form back to Cardiff University in the freepost envelope provided.

57727

PID - | 2 | 9 |

Ymddiriedolaeth GIG Gogledd Orllewin Cymru
North West Wales NHS Trust

NHS WALES GIG CYMRU
Ymddiriedolaeth GIG Gogledd Cymru
North Wales NHS Trust

CID - ☐ GLAN CLWYD
☐ GWYNEDD
☐ MAELOR

Confidential Questionnaire

Looking At Your Symptoms Study

This Study has been funded by Cancer Research UK

Cardiff University Prifysgol Caerdydd

Ysgol Glinigol Gogledd Cymru
North Wales Clinical School

Cancer Research UK

sewtu
South East Wales Trials Unit
Uned Ymchwil De-ddwyrain Cymru

Dear Patient

I am very grateful to you for agreeing to help us with this study.

I hope the information you supply will help us give people a diagnosis earlier.

If you feel you would like to talk to someone about this questionnaire at a later stage, please speak to the doctor or nurse who is looking after you, or be in touch with the research team, whose contact details are on your patient information sheet.

I can assure you that anything you tell us will be treated in the utmost confidence.

Yours sincerely

Richard Neal

Dr Richard Neal
Senior Clinical Lecturer
Cardiff University
Chief Investigator, Symptoms Study

About your symptoms

These questions are about symptoms you noticed before your diagnosis. We are interested in symptoms which you think are related to your cancer.

Please put a cross in the appropriate box, or write an answer as required.

Looking back...

1. Did you have any symptoms that you feel were due to your cancer before you were diagnosed?

Yes ☐ **Please continue with Question 2**

No ☐ **Please go to Section 2 on Page 5**

2. What was the first symptom that made you think something might be wrong?

[ ]

Please go to Question 3

The following questions are about when you first noticed a symptom and when you first told your GP or nurse about it.

Please give an exact date if you can. Otherwise give your best estimate. (For example approximately how long ago, the month or season). You wish to refer to your diary or calendar if you have it with you.

Here is an example question:

Decrease in appetite

Did you have this symptom?

Yes ☒ **Please complete A then B or C below**

No ☐ **Please go to Section 2**

A When did you first notice this? d d / m m / y y **OR** Estimate: '3 months ago' or 'June'

B When did you first tell your GP or nurse? 2 3 / 1 2 / 0 7 **OR** Estimate

OR

C Put a cross here if you didn't tell your GP or nurse ☐

3. Change in bowel habit

Did you have this symptom?

Yes ☐ **Please complete A then B or C below**

No ☐ **Please go to Question 4**

A When did you first notice this? ☐☐ / ☐☐ / ☐☐ **OR** ☐

B When did you first tell your GP or nurse? ☐☐ / ☐☐ / ☐☐ **OR** ☐

OR

C Put a cross here if you didn't tell your GP or nurse ☐

4. Bleeding from back passage

Did you have this symptom?

Yes ☐ **Please complete A then B or C below**

No ☐ **Please go to Section 2**

A When did you first notice this? ☐☐ / ☐☐ / ☐☐ **OR** ☐

B When did you first tell your GP or nurse? ☐☐ / ☐☐ / ☐☐ **OR** ☐

OR

C Put a cross here if you didn't tell your GP or nurse ☐

Please give an exact date if you can. Otherwise give your best estimate (for example approximately how long ago, the month or the season). You may wish to refer to your diary or calendar if you have it with you.

Did you have any of the following?

1. Decrease in appetite

Did you have this?

Yes ☐ **Please complete A then B or C below**

No ☐ **Please go to Question 2**

A When did you first notice this? ☐☐ / ☐☐ / ☐☐ **OR** ☐

B When did you first tell your GP or nurse? ☐☐ / ☐☐ / ☐☐ **OR** ☐

OR

C Put a cross here if you didn't tell your GP or nurse ☐

2. Unexplained weight loss

Did you have this?

Yes ☐ **Please complete A then B or C below**

No ☐ **Please go to Question 3**

A When did you first notice this? ☐☐ / ☐☐ / ☐☐ **OR** ☐

B When did you first tell your GP or nurse? ☐☐ / ☐☐ / ☐☐ **OR** ☐

OR

C Put a cross here if you didn't tell your GP or nurse ☐

3. **Fatigue or tiredness that is unusual for you**

Did you have this?

Yes ☐ **Please complete A then B or C below**

No ☐ **Please go to Question 4**

A When did you first notice this?

☐☐ / ☐☐ / ☐☐ **OR** ☐

B When did you first tell your GP or nurse?

☐☐ / ☐☐ / ☐☐ **OR** ☐

OR

C Put a cross here if you didn't tell your GP or nurse ☐

4. **Feeling different 'in yourself' from usual**

Did you have this?

Yes ☐ **Please complete A then B or C below**

No ☐ **Please go to Question 5**

A When did you first notice this?

☐☐ / ☐☐ / ☐☐ **OR** ☐

B When did you first tell your GP or nurse?

☐☐ / ☐☐ / ☐☐ **OR** ☐

OR

C Put a cross here if you didn't tell your GP or nurse ☐

Please try and describe what the feeling was

If you had any further symptoms which you feel are relevant to your cancer diagnosis, please list them below. If not, please go to Question 7.

Please describe the symptom here and complete A then B or C below

5.

**A**	When did you <u>first notice</u> this?	☐☐ / ☐☐ / ☐☐	**OR** ☐
**B**	When did you <u>first tell</u> your GP or nurse?	☐☐ / ☐☐ / ☐☐	**OR** ☐
	**OR**		
**C**	Put a cross here if you <u>didn't tell</u> your GP or nurse	☐	

If you had another symptom you feel is relevant, please write it below, otherwise go to Question 7.

Please describe the symptom here and complete A then B or C below

6.

**A**	When did you <u>first notice</u> this?	☐☐ / ☐☐ / ☐☐	**OR** ☐
**B**	When did you <u>first tell</u> your GP or nurse?	☐☐ / ☐☐ / ☐☐	**OR** ☐
	**OR**		
**C**	Put a cross here if you <u>didn't tell</u> your GP or nurse	☐	

If you had further symptoms you would like to tell us about, there is space at the end of the Questionnaire for you to write them.

Did your GP send you for any tests?

Thinking back to the time immediately before you diagnosis, did your GP send you for any of the following tests?

Please cross the appropriate boxes

7. Were you sent for any of these?

	Yes	Not sure	No
Blood test(s)	☐	☐	☐
CT Scan	☐	☐	☐
Ultrasound scan	☐	☐	☐
Barium Enema	☐	☐	☐
Sigmoidoscopy or colonoscopy (Looking at bowel with internal camera)	☐	☐	☐
X-ray	☐	☐	☐

Please go to Question 8

About you

The next questions relate to general information about you. It would help us to know more about the background of the people completing the questionnaire.

8. Which best describes your employment status?

Please cross one box only

- Employed full-time ☐
- Employed part-time ☐
- Self employed full-time ☐
- Self employed part-time ☐
- Unemployed (seeking work) ☐
- Unemployed (not seeking work) ☐
- Retired ☐
- Student ☐
- Permanently sick/disabled ☐
- Temporarily sick/disabled ☐
- Looking after family/home ☐
- Other, please describe ______

9. What is your highest level of qualification?

Please cross one box only

- Degree (or equivalent) ☐
- Diploma (or equivalent) ☐
- 'A' level ☐
- GCSE / 'O' level ☐
- None ☐
- Other, please specify ______

10. How would you describe your ethnicity

Please cross one box only

White ☐
or
- White British ☐
- White Irish ☐
- Other White background ☐

Mixed ☐
or
- White & Black Carribean ☐
- White & Black African ☐
- White & Asian ☐
- Other mixed background ☐

Black or Black British ☐
or
- Carribean ☐
- African ☐
- Other Black background ☐

Asian or Asian British ☐
or
- Indian ☐
- Pakistani ☐
- Bangladeshi ☐
- Other Asian background ☐

Chinese or other ethnic group ☐
or
- Chinese ☐
- Other ethnic group ☐

11.

Do you live alone?

Yes ☐ **Please go to Question 12**

No ☐ **Please say who you live with below**

Who do you live with?
(e.g. wife, husband, partner, family member)

12. Are you suffering from, or have you suffered from, any of the following in the past 2 years?

Please cross any that are applicable to you

- Asthma ☐
- Chronic Obstructive Pulmonary Disease (COPD) ☐
- Other lung disease (e.g. fibrosis, bronchiectatis etc) ☐
- Heart disease ☐
- Anxiety or depression ☐
- Inflammatory bowel disease ☐
- Irritable bowel syndrome ☐
- Peptic ulcer ☐
- Previous cancer ☐
- Diabetes ☐
- Arthritis ☐

13. About smoking

Please cross the appropriate statement

- Are you a current smoker? ☐
- Are you an ex-smoker? ☐
- Are you a non-smoker (never smoked)? ☐

14. Do you think you were more at risk of getting cancer because of your family history?

Yes ☐

No ☐

15. The following questions are about how completing the questionnaire made you feel.

A number of statements which people have used to describe themselves are given below. Read each statement and then cross the most appropriate box to indicate how you feel right now, at the moment. There are no right or wrong answers. Do not spend too much time on any one statement but give the answer which seems to describe your present feeling best.

		Not at all	Somewhat	Moderately	Very much
1.	I feel calm	☐	☐	☐	☐
2.	I am tense	☐	☐	☐	☐
3.	I feel upset	☐	☐	☐	☐
4.	I am relaxed	☐	☐	☐	☐
5.	I feel content	☐	☐	☐	☐
6.	I am worried	☐	☐	☐	☐

Please make sure you have answered all the questions.

If you have any other comments or there is anything else you would like to tell us about when you first noticed your symptoms, please do so using the space below.

If you feel you would like to talk to someone about this questionnaire at a later stage, please speak to the doctor or nurse who is looking after you, or be in touch with the research team, whose contact details are on your patient information sheet

Thank you for completing the questionnaire

Please give it to the research nurse

The next page is for the Researcher to complete

57727

29

Researcher - Post-completion Sheet

Researcher initials: ☐☐☐

Date questionnaire completed: Date ☐☐ / ☐☐ / ☐☐ Time ☐☐:☐☐

If not completed, give reason ☐

How long is it since the patient was told of his/her diagnosis? ☐

How long did it take the patient to complete the questionnaire?

- Less than 5 minutes ☐
- Between 5 and 10 minutes ☐
- Between 11 and 15 minutes ☐
- Longer than 15 minutes ☐

For the patient selected to complete the questionnaire on their own

Did the patient ask for any help? ☐ Yes ☐ No

If yes,

please specify what help was requested

____

____

please specify what help was given

____

____

For the patient selected for the researcher-administered questionnaire

Did the patient find any of the questions difficult? ☐ Yes ☐ No

If yes,

please specify which questions and what the difficulty was

____

____

____

____

Was the patient made anxious? ☐ Yes ☐ No

If yes,

please give details

____

____

____

____

Where did the patient complete the questionnaire?

(Please specify whether this was in a separate room alone, in a corner of the clinic, the ward or other

____

Thank you. Please now send the questionnaire and patient information sheet and consent form back to Cardiff University in the freepost envelope provided.

57727

PID - | | 3 | 0 |

Ymddiriedolaeth GIG Gogledd Orllewin Cymru
North West Wales NHS Trust

NHS WALES GIG CYMRU
Ymddiriedolaeth GIG Gogledd Cymru
North Wales NHS Trust

☐ GLAN CLWYD

CID - ☐ GWYNEDD

☐ MAELOR

Confidential Questionnaire

Looking At Your Symptoms Study

This Study has been funded by Cancer Research UK

Cardiff University / Prifysgol Caerdydd

Ysgol Glinigol Gogledd Cymru / North Wales Clinical School

Cancer Research UK

sewtu — South East Wales Trials Unit / Uned Ymchwil De-ddwyrain Cymru

Dear Patient

I am very grateful to you for agreeing to help us with this study.

I hope the information you supply will help us give people a diagnosis earlier.

If you feel you would like to talk to someone about this questionnaire at a later stage, please speak to the doctor or nurse who is looking after you, or be in touch with the research team, whose contact details are on your patient information sheet.

I can assure you that anything you tell us will be treated in the utmost confidence.

Yours sincerely

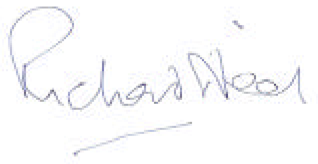

Dr Richard Neal
Senior Clinical Lecturer
Cardiff University
Chief Investigator, Symptoms Study

About your symptoms

These questions are about symptoms you noticed before your diagnosis. We are interested in symptoms which you think are related to your cancer.

Please put a cross in the appropriate box, or write an answer as required.

Looking back...

1. Did you have any symptoms that you feel were due to your cancer before you were diagnosed?

Yes ☐ **Please continue with Question 2**

No ☐ **Please go to Section 2 on Page 5**

2. What was the first symptom that made you think something might be wrong?

☐

Please go to Question 3

The following questions are about when you first noticed a symptom and when you first told your GP or nurse about it.

Please give an exact date if you can. Otherwise give your best estimate. (For example approximately how long ago, the month or season). You wish to refer to your diary or calendar if you have it with you.

Here is an example question:

Decrease in appetite

Did you have this symptom?

Yes ☒ **Please complete A then B or C below**

No ☐ **Please go to Section 2**

A When did you first notice this? d d / m m / y y **OR** Estimate '3 months ago' or 'June'

B When did you first tell your GP or nurse? 2 3 / 1 2 / 0 7 **OR** Estimate

OR

C Put a cross here if you didn't tell your GP or nurse ☐

3. Change in bowel habit

Did you have this symptom?

Yes ☐ **Please complete A then B or C below**

No ☐ **Please go to Question 4**

A When did you first notice this? __/__/__ **OR** ______

B When did you first tell your GP or nurse? __/__/__ **OR** ______

OR

C Put a cross here if you didn't tell your GP or nurse ☐

4. Bleeding from back passage

Did you have this symptom?

Yes ☐ **Please complete A then B or C below**

No ☐ **Please go to Section 2**

A When did you first notice this? __/__/__ **OR** ______

B When did you first tell your GP or nurse? __/__/__ **OR** ______

OR

C Put a cross here if you didn't tell your GP or nurse ☐

Section 2

Please give an exact date if you can. Otherwise give your best estimate (for example approximately how long ago, the month or the season). You may wish to refer to your diary or calendar if you have it with you.

Did you have any of the following?

1. Decrease in appetite

Did you have this?

Yes ☐ **Please complete A then B or C below**

No ☐ **Please go to Question 2**

A When did you first notice this? ☐☐ / ☐☐ / ☐☐ **OR** ☐

B When did you first tell your GP or nurse? ☐☐ / ☐☐ / ☐☐ **OR** ☐

OR

C Put a cross here if you didn't tell your GP or nurse ☐

2. Unexplained weight loss

Did you have this?

Yes ☐ **Please complete A then B or C below**

No ☐ **Please go to Question 3**

A When did you first notice this? ☐☐ / ☐☐ / ☐☐ **OR** ☐

B When did you first tell your GP or nurse? ☐☐ / ☐☐ / ☐☐ **OR** ☐

OR

C Put a cross here if you didn't tell your GP or nurse ☐

3. Fatigue or tiredness that is unusual for you

Did you have this?

Yes ☐ **Please complete A then B or C below**

No ☐ **Please go to Question 4**

A When did you first notice this? ☐☐ / ☐☐ / ☐☐ **OR** ☐

B When did you first tell your GP or nurse? ☐☐ / ☐☐ / ☐☐ **OR** ☐

OR

C Put a cross here if you didn't tell your GP or nurse ☐

4. Feeling different 'in yourself' from usual

Did you have this?

Yes ☐ **Please complete A then B or C below**

No ☐ **Please go to Question 5**

A When did you first notice this? ☐☐ / ☐☐ / ☐☐ **OR** ☐

B When did you first tell your GP or nurse? ☐☐ / ☐☐ / ☐☐ **OR** ☐

OR

C Put a cross here if you didn't tell your GP or nurse ☐

Please try and describe what the feeling was

If you had any further symptoms which you feel are relevant to your cancer diagnosis, please list them below. If not, please go to Question 7.

Please describe the symptom here and complete A then B or C below

5.

**A**	When did you first notice this?	__ __ / __ __ / __ __ **OR**
**B**	When did you first tell your GP or nurse?	__ __ / __ __ / __ __ **OR**
	**OR**	
**C**	Put a cross here if you didn't tell your GP or nurse	☐

If you had another symptom you feel is relevant, please write it below, otherwise go to Question 7.

Please describe the symptom here and complete A then B or C below

6.

**A**	When did you first notice this?	__ __ / __ __ / __ __ **OR**
**B**	When did you first tell your GP or nurse?	__ __ / __ __ / __ __ **OR**
	**OR**	
**C**	Put a cross here if you didn't tell your GP or nurse	☐

If you had further symptoms you would like to tell us about, there is space at the end of the Questionnaire for you to write them.

Did your GP send you for any tests?

Thinking back to the time immediately before you diagnosis, did your GP send you for any of the following tests?

Please cross the appropriate boxes

7. Were you sent for any of these?

	Yes	Not sure	No
Blood test(s)	☐	☐	☐
CT Scan	☐	☐	☐
Ultrasound scan	☐	☐	☐
Barium Enema	☐	☐	☐
Sigmoidoscopy or colonoscopy (Looking at bowel with internal camera)	☐	☐	☐
X-ray	☐	☐	☐

Please go to Question 8

About you

The next questions relate to general information about you. It would help us to know more about the background of the people completing the questionnaire.

8. **Which best describes your employment status?**

Please cross one box only

- Employed full-time ☐
- Employed part-time ☐
- Self employed full-time ☐
- Self employed part-time ☐
- Unemployed (seeking work) ☐
- Unemployed (not seeking work) ☐
- Retired ☐
- Student ☐
- Permanently sick/disabled ☐
- Temporarily sick/disabled ☐
- Looking after family/home ☐
- Other, please describe ______

9. **What is your highest level of qualification?**

Please cross one box only

- Degree (or equivalent) ☐
- Diploma (or equivalent) ☐
- 'A' level ☐
- GCSE / 'O' level ☐
- None ☐
- Other, please specify ______

10. How would you describe your ethnicity

Please cross one box only

White ☐
or
- White British ☐
- White Irish ☐
- Other White background ☐

Mixed ☐
or
- White & Black Carribean ☐
- White & Black African ☐
- White & Asian ☐
- Other mixed background ☐

Black or Black British ☐
or
- Carribean ☐
- African ☐
- Other Black background ☐

Asian or Asian British ☐
or
- Indian ☐
- Pakistani ☐
- Bangladeshi ☐
- Other Asian background ☐

Chinese or other ethnic group ☐
or
- Chinese ☐
- Other ethnic group ☐

11.

Do you live alone?

Yes ☐ **Please go to Question 12**

No ☐ **Please say who you live with below**

Who do you live with?
(e.g. wife, husband, partner, family member)

12. **Are you suffering from, or have you suffered from, any of the following in the past 2 years?**

Please cross any that are applicable to you

- Asthma ☐
- Chronic Obstructive Pulmonary Disease (COPD) ☐
- Other lung disease (e.g. fibrosis, bronchiectatis etc) ☐
- Heart disease ☐
- Anxiety or depression ☐
- Inflammatory bowel disease ☐
- Irritable bowel syndrome ☐
- Peptic ulcer ☐
- Previous cancer ☐
- Diabetes ☐
- Arthritis ☐

13. **About smoking**

Please cross the appropriate statement

- Are you a current smoker? ☐
- Are you an ex-smoker? ☐
- Are you a non-smoker (never smoked)? ☐

14. **Do you think you were more at risk of getting cancer because of your family history?**

Yes ☐

No ☐

15. **The following questions are about how completing the questionnaire made you feel.**

A number of statements which people have used to describe themselves are given below. Read each statement and then cross the most appropriate box to indicate how you feel right now, at the moment. There are no right or wrong answers. Do not spend too much time on any one statement but give the answer which seems to describe your present feeling best.

		Not at all	Somewhat	Moderately	Very much
1.	I feel calm	☐	☐	☐	☐
2.	I am tense	☐	☐	☐	☐
3.	I feel upset	☐	☐	☐	☐
4.	I am relaxed	☐	☐	☐	☐
5.	I feel content	☐	☐	☐	☐
6.	I am worried	☐	☐	☐	☐

Please make sure you have answered all the questions.

If you have any other comments or there is anything else you would like to tell us about when you first noticed your symptoms, please do so using the space below.

If you feel you would like to talk to someone about this questionnaire at a later stage, please speak to the doctor or nurse who is looking after you, or be in touch with the research team, whose contact details are on your patient information sheet

Thank you for completing the questionnaire

Please give it to the research nurse

The next page is for the Researcher to complete

57727

30

Researcher - Post-completion Sheet

Researcher initials: ☐☐☐

Date questionnaire completed: Date ☐☐ / ☐☐ / ☐☐ Time ☐☐:☐☐

If not completed, give reason ☐

How long is it since the patient was told of his/her diagnosis? ☐

How long did it take the patient to complete the questionnaire?

- Less than 5 minutes ☐
- Between 5 and 10 minutes ☐
- Between 11 and 15 minutes ☐
- Longer than 15 minutes ☐

For the patient selected to complete the questionnaire on their own

Did the patient ask for any help? ☐ Yes ☐ No

If yes,

please specify what help was requested

___

___

please specify what help was given

___

___

For the patient selected for the researcher-administered questionnaire

Did the patient find any of the questions difficult? ☐ Yes ☐ No

If yes,

please specify which questions and what the difficulty was

___

___

___

___

Was the patient made anxious? ☐ Yes ☐ No

If yes,

please give details

___

___

___

___

Where did the patient complete the questionnaire?
(Please specify whether this was in a separate room alone, in a corner of the clinic, the ward or other

___

Thank you. Please now send the questionnaire and patient information sheet and consent form back to Cardiff University in the freepost envelope provided.

57727

PID - | | 3 | 1 |

Ymddiriedolaeth GIG Gogledd Orllewin Cymru
North West Wales NHS Trust

NHS WALES GIG CYMRU
Ymddiriedolaeth GIG Gogledd Cymru
North Wales NHS Trust

☐ GLAN CLWYD

CID - ☐ GWYNEDD

☐ MAELOR

Confidential Questionnaire

Looking At Your Symptoms Study

This Study has been funded by Cancer Research UK

Cardiff University / Prifysgol Caerdydd

Ysgol Glinigol Gogledd Cymru / North Wales Clinical School

Cancer Research UK

sewtu South East Wales Trials Unit / Uned Ymchwil De-ddwyrain Cymru

Dear Patient

I am very grateful to you for agreeing to help us with this study.

I hope the information you supply will help us give people a diagnosis earlier.

If you feel you would like to talk to someone about this questionnaire at a later stage, please speak to the doctor or nurse who is looking after you, or be in touch with the research team, whose contact details are on your patient information sheet.

I can assure you that anything you tell us will be treated in the utmost confidence.

Yours sincerely

Dr Richard Neal
Senior Clinical Lecturer
Cardiff University
Chief Investigator, Symptoms Study

About your symptoms

These questions are about symptoms you noticed before your diagnosis. We are interested in symptoms which you think are related to your cancer.

Please put a cross in the appropriate box, or write an answer as required.

Looking back...

1. Did you have any symptoms that you feel were due to your cancer before you were diagnosed?

Yes ☐ **Please continue with Question 2**

No ☐ **Please go to Section 2 on Page 5**

2. What was the first symptom that made you think something might be wrong?

☐

Please go to Question 3

The following questions are about when you first noticed a symptom and when you first told your GP or nurse about it.

Please give an exact date if you can. Otherwise give your best estimate. (For example approximately how long ago, the month or season). You wish to refer to your diary or calendar if you have it with you.

Here is an example question:

Decrease in appetite

Did you have this symptom?

Yes ☒ **Please complete A then B or C below**

No ☐ **Please go to Section 2**

A When did you first notice this? d d / m m / y y **OR** Estimate '3 months ago' or 'June'

B When did you first tell your GP or nurse? 2 3 / 1 2 / 0 7 **OR** Estimate

OR

C Put a cross here if you didn't tell your GP or nurse ☐

3. Change in bowel habit

Did you have this symptom?

Yes ☐ **Please complete A then B or C below**

No ☐ **Please go to Question 4**

A When did you first notice this? ☐☐ / ☐☐ / ☐☐ **OR** ☐

B When did you first tell your GP or nurse? ☐☐ / ☐☐ / ☐☐ **OR** ☐

OR

C Put a cross here if you didn't tell your GP or nurse ☐

4. Bleeding from back passage

Did you have this symptom?

Yes ☐ **Please complete A then B or C below**

No ☐ **Please go to Section 2**

A When did you first notice this? ☐☐ / ☐☐ / ☐☐ **OR** ☐

B When did you first tell your GP or nurse? ☐☐ / ☐☐ / ☐☐ **OR** ☐

OR

C Put a cross here if you didn't tell your GP or nurse ☐

Section 2

Please give an exact date if you can. Otherwise give your best estimate (for example approximately how long ago, the month or the season). You may wish to refer to your diary or calendar if you have it with you.

Did you have any of the following?

1. Decrease in appetite

Did you have this?

Yes ☐ **Please complete A then B or C below**

No ☐ **Please go to Question 2**

A When did you first notice this? ☐☐ / ☐☐ / ☐☐ **OR** ☐

B When did you first tell your GP or nurse? ☐☐ / ☐☐ / ☐☐ **OR** ☐

OR

C Put a cross here if you didn't tell your GP or nurse ☐

2. Unexplained weight loss

Did you have this?

Yes ☐ **Please complete A then B or C below**

No ☐ **Please go to Question 3**

A When did you first notice this? ☐☐ / ☐☐ / ☐☐ **OR** ☐

B When did you first tell your GP or nurse? ☐☐ / ☐☐ / ☐☐ **OR** ☐

OR

C Put a cross here if you didn't tell your GP or nurse ☐

3. Fatigue or tiredness that is unusual for you

Did you have this?

Yes ☐ **Please complete A then B or C below**

No ☐ **Please go to Question 4**

A When did you first notice this? ☐☐ / ☐☐ / ☐☐ **OR** ☐

B When did you first tell your GP or nurse? ☐☐ / ☐☐ / ☐☐ **OR** ☐

OR

C Put a cross here if you didn't tell your GP or nurse ☐

4. Feeling different 'in yourself' from usual

Did you have this?

Yes ☐ **Please complete A then B or C below**

No ☐ **Please go to Question 5**

A When did you first notice this? ☐☐ / ☐☐ / ☐☐ **OR** ☐

B When did you first tell your GP or nurse? ☐☐ / ☐☐ / ☐☐ **OR** ☐

OR

C Put a cross here if you didn't tell your GP or nurse ☐

Please try and describe what the feeling was

If you had any further symptoms which you feel are relevant to your cancer diagnosis, please list them below. If not, please go to Question 7.

Please describe the symptom here and complete A then B or C below

5.

**A**	When did you first notice this?	__ __ / __ __ / __ __ **OR**
**B**	When did you first tell your GP or nurse?	__ __ / __ __ / __ __ **OR**
	**OR**	
**C**	Put a cross here if you didn't tell your GP or nurse	☐

If you had another symptom you feel is relevant, please write it below, otherwise go to Question 7.

Please describe the symptom here and complete A then B or C below

6.

**A**	When did you first notice this?	__ __ / __ __ / __ __ **OR**
**B**	When did you first tell your GP or nurse?	__ __ / __ __ / __ __ **OR**
	**OR**	
**C**	Put a cross here if you didn't tell your GP or nurse	☐

If you had further symptoms you would like to tell us about, there is space at the end of the Questionnaire for you to write them.

Did your GP send you for any tests?

Thinking back to the time immediately before you diagnosis, did your GP send you for any of the following tests?

Please cross the appropriate boxes

7. Were you sent for any of these?

	Yes	Not sure	No
Blood test(s)	☐	☐	☐
CT Scan	☐	☐	☐
Ultrasound scan	☐	☐	☐
Barium Enema	☐	☐	☐
Sigmoidoscopy or colonoscopy (Looking at bowel with internal camera)	☐	☐	☐
X-ray	☐	☐	☐

Please go to Question 8

About you

The next questions relate to general information about you. It would help us to know more about the background of the people completing the questionnaire.

8. Which best describes your employment status?

Please cross one box only

- Employed full-time ☐
- Employed part-time ☐
- Self employed full-time ☐
- Self employed part-time ☐
- Unemployed (seeking work) ☐
- Unemployed (not seeking work) ☐
- Retired ☐
- Student ☐
- Permanently sick/disabled ☐
- Temporarily sick/disabled ☐
- Looking after family/home ☐
- Other, please describe ____________

9. What is your highest level of qualification?

Please cross one box only

- Degree (or equivalent) ☐
- Diploma (or equivalent) ☐
- 'A' level ☐
- GCSE / 'O' level ☐
- None ☐
- Other, please specify ____________

10. **How would you describe your ethnicity**

Please cross one box only

White ☐
or
- White British ☐
- White Irish ☐
- Other White background ☐

Mixed ☐
or
- White & Black Carribean ☐
- White & Black African ☐
- White & Asian ☐
- Other mixed background ☐

Black or Black British ☐
or
- Carribean ☐
- African ☐
- Other Black background ☐

Asian or Asian British ☐
or
- Indian ☐
- Pakistani ☐
- Bangladeshi ☐
- Other Asian background ☐

Chinese or other ethnic group ☐
or
- Chinese ☐
- Other ethnic group ☐

11.

Do you live alone?

Yes ☐ **Please go to Question 12**

No ☐ **Please say who you live with below**

Who do you live with?
(e.g. wife, husband, partner, family member)

12. Are you suffering from, or have you suffered from, any of the following in the past 2 years?

Please cross any that are applicable to you

Asthma ☐

Chronic Obstructive Pulmonary Disease (COPD) ☐

Other lung disease (e.g. fibrosis, bronchiectatis etc) ☐

Heart disease ☐

Anxiety or depression ☐

Inflammatory bowel disease ☐

Irritable bowel syndrome ☐

Peptic ulcer ☐

Previous cancer ☐

Diabetes ☐

Arthritis ☐

13. About smoking

Please cross the appropriate statement

Are you a current smoker? ☐

Are you an ex-smoker? ☐

Are you a non-smoker (never smoked)? ☐

14. Do you think you were more at risk of getting cancer because of your family history?

Yes ☐

No ☐

15. **The following questions are about how completing the questionnaire made you feel.**

A number of statements which people have used to describe themselves are given below. Read each statement and then cross the most appropriate box to indicate how you feel right now, at the moment. There are no right or wrong answers. Do not spend too much time on any one statement but give the answer which seems to describe your present feeling best.

		Not at all	Somewhat	Moderately	Very much
1.	I feel calm	☐	☐	☐	☐
2.	I am tense	☐	☐	☐	☐
3.	I feel upset	☐	☐	☐	☐
4.	I am relaxed	☐	☐	☐	☐
5.	I feel content	☐	☐	☐	☐
6.	I am worried	☐	☐	☐	☐

Please make sure you have answered all the questions.

If you have any other comments or there is anything else you would like to tell us about when you first noticed your symptoms, please do so using the space below.

If you feel you would like to talk to someone about this questionnaire at a later stage, please speak to the doctor or nurse who is looking after you, or be in touch with the research team, whose contact details are on your patient information sheet

Thank you for completing the questionnaire

Please give it to the research nurse

The next page is for the Researcher to complete

Researcher - Post-completion Sheet **Researcher initials:** ☐☐☐

Date questionnaire completed: Date ☐☐ / ☐☐ / ☐☐ Time ☐☐:☐☐

If not completed, give reason ☐

How long is it since the patient was told of his/her diagnosis? ☐

How long did it take the patient to complete the questionnaire?

- Less than 5 minutes ☐
- Between 5 and 10 minutes ☐
- Between 11 and 15 minutes ☐
- Longer than 15 minutes ☐

For the patient selected to complete the questionnaire on their own

Did the patient ask for any help? ☐ Yes ☐ No

If yes,

please specify what help was requested

____

____

please specify what help was given

____

____

For the patient selected for the researcher-administered questionnaire

Did the patient find any of the questions difficult? ☐ Yes ☐ No

If yes,

please specify which questions and what the difficulty was

____

____

____

____

Was the patient made anxious? ☐ Yes ☐ No

If yes,

please give details

____

____

____

____

Where did the patient complete the questionnaire?

(Please specify whether this was in a separate room alone, in a corner of the clinic, the ward or other

____

Thank you. Please now send the questionnaire and patient information sheet and consent form back to Cardiff University in the freepost envelope provided.

57727

PID - | | 3 | 2 |

Ymddiriedolaeth GIG Gogledd Orllewin Cymru
North West Wales NHS Trust

NHS WALES GIG CYMRU
Ymddiriedolaeth GIG Gogledd Cymru
North Wales NHS Trust

CID - ☐ GLAN CLWYD
☐ GWYNEDD
☐ MAELOR

Confidential Questionnaire

Looking At Your Symptoms Study

This Study has been funded by Cancer Research UK

CARDIFF UNIVERSITY PRIFYSGOL CAERDYDD
Ysgol Glinigol Gogledd Cymru North Wales Clinical School
CANCER RESEARCH UK
sewtu South East Wales Trials Unit Uned Ymchwil De-ddwyrain Cymru

Dear Patient

I am very grateful to you for agreeing to help us with this study.

I hope the information you supply will help us give people a diagnosis earlier.

If you feel you would like to talk to someone about this questionnaire at a later stage, please speak to the doctor or nurse who is looking after you, or be in touch with the research team, whose contact details are on your patient information sheet.

I can assure you that anything you tell us will be treated in the utmost confidence.

Yours sincerely

Dr Richard Neal
Senior Clinical Lecturer
Cardiff University
Chief Investigator, Symptoms Study

About your symptoms

These questions are about symptoms you noticed before your diagnosis. We are interested in symptoms which you think are related to your cancer.

Please put a cross in the appropriate box, or write an answer as required.

Looking back...

1. Did you have any symptoms that you feel were due to your cancer before you were diagnosed?

Yes ☐ **Please continue with Question 2**

No ☐ **Please go to Section 2 on Page 5**

2. What was the first symptom that made you think something might be wrong?

[ ]

Please go to Question 3

The following questions are about when you first noticed a symptom and when you first told your GP or nurse about it.

Please give an exact date if you can. Otherwise give your best estimate. (For example approximately how long ago, the month or season). You wish to refer to your diary or calendar if you have it with you.

Here is an example question:

Decrease in appetite

Did you have this symptom?

Yes ☒ **Please complete A then B or C below**

No ☐ **Please go to Section 2**

A When did you first notice this? d d / m m / y y **OR** Estimate '3 months ago' or 'June'

B When did you first tell your GP or nurse? 2 3 / 1 2 / 0 7 **OR** Estimate

OR

C Put a cross here if you didn't tell your GP or nurse ☐

3. Change in bowel habit

Did you have this symptom?

Yes ☐ **Please complete A then B or C below**

No ☐ **Please go to Question 4**

A When did you first notice this? ☐☐ / ☐☐ / ☐☐ **OR** ☐

B When did you first tell your GP or nurse? ☐☐ / ☐☐ / ☐☐ **OR** ☐

OR

C Put a cross here if you didn't tell your GP or nurse ☐

4. Bleeding from back passage

Did you have this symptom?

Yes ☐ **Please complete A then B or C below**

No ☐ **Please go to Section 2**

A When did you first notice this? ☐☐ / ☐☐ / ☐☐ **OR** ☐

B When did you first tell your GP or nurse? ☐☐ / ☐☐ / ☐☐ **OR** ☐

OR

C Put a cross here if you didn't tell your GP or nurse ☐

Section 2

Please give an exact date if you can. Otherwise give your best estimate (for example approximately how long ago, the month or the season). You may wish to refer to your diary or calendar if you have it with you.

Did you have any of the following?

1. Decrease in appetite

Did you have this?

Yes ☐ **Please complete A then B or C below**

No ☐ **Please go to Question 2**

A When did you first notice this? ☐☐ / ☐☐ / ☐☐ **OR** ☐

B When did you first tell your GP or nurse? ☐☐ / ☐☐ / ☐☐ **OR** ☐

OR

C Put a cross here if you didn't tell your GP or nurse ☐

2. Unexplained weight loss

Did you have this?

Yes ☐ **Please complete A then B or C below**

No ☐ **Please go to Question 3**

A When did you first notice this? ☐☐ / ☐☐ / ☐☐ **OR** ☐

B When did you first tell your GP or nurse? ☐☐ / ☐☐ / ☐☐ **OR** ☐

OR

C Put a cross here if you didn't tell your GP or nurse ☐

3. **Fatigue or tiredness that is unusual for you**

Did you have this?

Yes ☐ **Please complete A then B or C below**

No ☐ **Please go to Question 4**

A When did you <u>first notice</u> this? ☐☐ / ☐☐ / ☐☐ **OR** ☐

B When did you <u>first tell</u> your GP or nurse? ☐☐ / ☐☐ / ☐☐ **OR** ☐

OR

C Put a cross here if you <u>didn't tell</u> your GP or nurse ☐

4. **Feeling different 'in yourself' from usual**

Did you have this?

Yes ☐ **Please complete A then B or C below**

No ☐ **Please go to Question 5**

A When did you <u>first notice</u> this? ☐☐ / ☐☐ / ☐☐ **OR** ☐

B When did you <u>first tell</u> your GP or nurse? ☐☐ / ☐☐ / ☐☐ **OR** ☐

OR

C Put a cross here if you <u>didn't tell</u> your GP or nurse ☐

Please try and describe what the feeling was

If you had any further symptoms which you feel are relevant to your cancer diagnosis, please list them below. If not, please go to Question 7.

Please describe the symptom here and complete A then B or C below

5.

**A**	When did you first notice this?	☐☐ / ☐☐ / ☐☐ **OR** ☐
**B**	When did you first tell your GP or nurse?	☐☐ / ☐☐ / ☐☐ **OR** ☐
	**OR**	
**C**	Put a cross here if you didn't tell your GP or nurse	☐

If you had another symptom you feel is relevant, please write it below, otherwise go to Question 7.

Please describe the symptom here and complete A then B or C below

6.

**A**	When did you first notice this?	☐☐ / ☐☐ / ☐☐ **OR** ☐
**B**	When did you first tell your GP or nurse?	☐☐ / ☐☐ / ☐☐ **OR** ☐
	**OR**	
**C**	Put a cross here if you didn't tell your GP or nurse	☐

If you had further symptoms you would like to tell us about, there is space at the end of the Questionnaire for you to write them.

Did your GP send you for any tests?

Thinking back to the time immediately before you diagnosis, did your GP send you for any of the following tests?

Please cross the appropriate boxes

7. Were you sent for any of these?

	Yes	Not sure	No
Blood test(s)	☐	☐	☐
CT Scan	☐	☐	☐
Ultrasound scan	☐	☐	☐
Barium Enema	☐	☐	☐
Sigmoidoscopy or colonoscopy (Looking at bowel with internal camera)	☐	☐	☐
X-ray	☐	☐	☐

Please go to Question 8

About you

The next questions relate to general information about you. It would help us to know more about the background of the people completing the questionnaire.

8. Which best describes your employment status?

Please cross one box only

- Employed full-time ☐
- Employed part-time ☐
- Self employed full-time ☐
- Self employed part-time ☐
- Unemployed (seeking work) ☐
- Unemployed (not seeking work) ☐
- Retired ☐
- Student ☐
- Permanently sick/disabled ☐
- Temporarily sick/disabled ☐
- Looking after family/home ☐

Other, please describe

9. What is your highest level of qualification?

Please cross one box only

- Degree (or equivalent) ☐
- Diploma (or equivalent) ☐
- 'A' level ☐
- GCSE / 'O' level ☐
- None ☐

Other, please specify

10. How would you describe your ethnicity

Please cross one box only

White ☐
or
- White British ☐
- White Irish ☐
- Other White background ☐

Mixed ☐
or
- White & Black Carribean ☐
- White & Black African ☐
- White & Asian ☐
- Other mixed background ☐

Black or Black British ☐
or
- Carribean ☐
- African ☐
- Other Black background ☐

Asian or Asian British ☐
or
- Indian ☐
- Pakistani ☐
- Bangladeshi ☐
- Other Asian background ☐

Chinese or other ethnic group ☐
or
- Chinese ☐
- Other ethnic group ☐

11.

Do you live alone?

Yes ☐ **Please go to Question 12**

No ☐ **Please say who you live with below**

Who do you live with?
(e.g. wife, husband, partner, family member)

12. **Are you suffering from, or have you suffered from, any of the following in the past 2 years?**

Please cross any that are applicable to you

- Asthma ☐
- Chronic Obstructive Pulmonary Disease (COPD) ☐
- Other lung disease (e.g. fibrosis, bronchiectatis etc) ☐
- Heart disease ☐
- Anxiety or depression ☐
- Inflammatory bowel disease ☐
- Irritable bowel syndrome ☐
- Peptic ulcer ☐
- Previous cancer ☐
- Diabetes ☐
- Arthritis ☐

13. **About smoking**

Please cross the appropriate statement

- Are you a current smoker? ☐
- Are you an ex-smoker? ☐
- Are you a non-smoker (never smoked)? ☐

14. **Do you think you were more at risk of getting cancer because of your family history?**

Yes ☐

No ☐

15. **The following questions are about how completing the questionnaire made you feel.**

A number of statements which people have used to describe themselves are given below. Read each statement and then cross the most appropriate box to indicate how you feel right now, at the moment. There are no right or wrong answers. Do not spend too much time on any one statement but give the answer which seems to describe your present feeling best.

		Not at all	Somewhat	Moderately	Very much
1.	I feel calm	☐	☐	☐	☐
2.	I am tense	☐	☐	☐	☐
3.	I feel upset	☐	☐	☐	☐
4.	I am relaxed	☐	☐	☐	☐
5.	I feel content	☐	☐	☐	☐
6.	I am worried	☐	☐	☐	☐

Please make sure you have answered all the questions.

If you have any other comments or there is anything else you would like to tell us about when you first noticed your symptoms, please do so using the space below.

If you feel you would like to talk to someone about this questionnaire at a later stage, please speak to the doctor or nurse who is looking after you, or be in touch with the research team, whose contact details are on your patient information sheet

Thank you for completing the questionnaire

Please give it to the research nurse

The next page is for the Researcher to complete

57727

3 2

Researcher - Post-completion Sheet

Researcher initials: ☐☐☐

Date questionnaire completed: Date ☐☐ / ☐☐ / ☐☐ Time ☐☐:☐☐

If not completed, give reason ☐

How long is it since the patient was told of his/her diagnosis? ☐

How long did it take the patient to complete the questionnaire?

- Less than 5 minutes ☐
- Between 5 and 10 minutes ☐
- Between 11 and 15 minutes ☐
- Longer than 15 minutes ☐

For the patient selected to complete the questionnaire on their own

Did the patient ask for any help? ☐ Yes ☐ No

If yes,

please specify what help was requested

___

___

please specify what help was given

___

___

For the patient selected for the researcher-administered questionnaire

Did the patient find any of the questions difficult? ☐ Yes ☐ No

If yes,

please specify which questions and what the difficulty was

___

___

___

___

Was the patient made anxious? ☐ Yes ☐ No

If yes,

please give details

___

___

___

___

Where did the patient complete the questionnaire?

(Please specify whether this was in a separate room alone, in a corner of the clinic, the ward or other

___

Thank you. Please now send the questionnaire and patient information sheet and consent form back to Cardiff University in the freepost envelope provided.

57727

PID - | | 3 | 3 |

Ymddiriedolaeth GIG Gogledd Orllewin Cymru
North West Wales NHS Trust

NHS WALES GIG CYMRU
Ymddiriedolaeth GIG Gogledd Cymru
North Wales NHS Trust

☐ GLAN CLWYD

CID - ☐ GWYNEDD

☐ MAELOR

Confidential Questionnaire

Looking At Your Symptoms Study

This Study has been funded by Cancer Research UK

Cardiff University / Prifysgol Caerdydd

Ysgol Glinigol Gogledd Cymru / North Wales Clinical School

Cancer Research UK

sewtu — South East Wales Trials Unit / Uned Ymchwil De-ddwyrain Cymru

Dear Patient

I am very grateful to you for agreeing to help us with this study.

I hope the information you supply will help us give people a diagnosis earlier.

If you feel you would like to talk to someone about this questionnaire at a later stage, please speak to the doctor or nurse who is looking after you, or be in touch with the research team, whose contact details are on your patient information sheet.

I can assure you that anything you tell us will be treated in the utmost confidence.

Yours sincerely

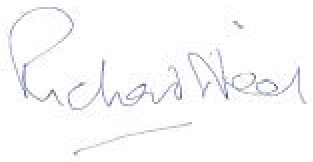

Dr Richard Neal
Senior Clinical Lecturer
Cardiff University
Chief Investigator, Symptoms Study

About your symptoms

These questions are about symptoms you noticed before your diagnosis. We are interested in symptoms which you think are related to your cancer.

Please put a cross in the appropriate box, or write an answer as required.

Looking back...

1. Did you have any symptoms that you feel were due to your cancer before you were diagnosed?

Yes ☐ **Please continue with Question 2**

No ☐ **Please go to Section 2 on Page 5**

2. What was the first symptom that made you think something might be wrong?

☐

Please go to Question 3

The following questions are about when you first noticed a symptom and when you first told your GP or nurse about it.

Please give an exact date if you can. Otherwise give your best estimate. (For example approximately how long ago, the month or season). You wish to refer to your diary or calendar if you have it with you.

Here is an example question:

Decrease in appetite

Did you have this symptom?

Yes ☒ **Please complete A then B or C below**

No ☐ **Please go to Section 2**

A When did you first notice this? d d / m m / y y **OR** Estimate '3 months ago' or 'June'

B When did you first tell your GP or nurse? 2 3 / 1 2 / 0 7 **OR** Estimate

OR

C Put a cross here if you didn't tell your GP or nurse ☐

3. Change in bowel habit

Did you have this symptom?

Yes ☐ **Please complete A then B or C below**

No ☐ **Please go to Question 4**

A When did you first notice this?

☐☐ / ☐☐ / ☐☐ **OR** ☐

B When did you first tell your GP or nurse?

☐☐ / ☐☐ / ☐☐ **OR** ☐

OR

C Put a cross here if you didn't tell your GP or nurse

☐

4. Bleeding from back passage

Did you have this symptom?

Yes ☐ **Please complete A then B or C below**

No ☐ **Please go to Section 2**

A When did you first notice this?

☐☐ / ☐☐ / ☐☐ **OR** ☐

B When did you first tell your GP or nurse?

☐☐ / ☐☐ / ☐☐ **OR** ☐

OR

C Put a cross here if you didn't tell your GP or nurse

☐

Section 2

Please give an exact date if you can. Otherwise give your best estimate (for example approximately how long ago, the month or the season). You may wish to refer to your diary or calendar if you have it with you.

Did you have any of the following?

1. Decrease in appetite

Did you have this?

Yes ☐ **Please complete A then B or C below**

No ☐ **Please go to Question 2**

A When did you first notice this? ☐☐ / ☐☐ / ☐☐ **OR** ☐

B When did you first tell your GP or nurse? ☐☐ / ☐☐ / ☐☐ **OR** ☐

OR

C Put a cross here if you didn't tell your GP or nurse ☐

2. Unexplained weight loss

Did you have this?

Yes ☐ **Please complete A then B or C below**

No ☐ **Please go to Question 3**

A When did you first notice this? ☐☐ / ☐☐ / ☐☐ **OR** ☐

B When did you first tell your GP or nurse? ☐☐ / ☐☐ / ☐☐ **OR** ☐

OR

C Put a cross here if you didn't tell your GP or nurse ☐

3. **Fatigue or tiredness that is unusual for you**

Did you have this?

Yes ☐ **Please complete A then B or C below**

No ☐ **Please go to Question 4**

A When did you first notice this? ☐☐ / ☐☐ / ☐☐ **OR** ☐

B When did you first tell your GP or nurse? ☐☐ / ☐☐ / ☐☐ **OR** ☐

OR

C Put a cross here if you didn't tell your GP or nurse ☐

4. **Feeling different 'in yourself' from usual**

Did you have this?

Yes ☐ **Please complete A then B or C below**

No ☐ **Please go to Question 5**

A When did you first notice this? ☐☐ / ☐☐ / ☐☐ **OR** ☐

B When did you first tell your GP or nurse? ☐☐ / ☐☐ / ☐☐ **OR** ☐

OR

C Put a cross here if you didn't tell your GP or nurse ☐

Please try and describe what the feeling was

If you had any further symptoms which you feel are relevant to your cancer diagnosis, please list them below. If not, please go to Question 7.

Please describe the symptom here and complete A then B or C below

5.

A When did you first notice this? ☐☐ / ☐☐ / ☐☐ **OR**

B When did you first tell your GP or nurse? ☐☐ / ☐☐ / ☐☐ **OR**

OR

C Put a cross here if you didn't tell your GP or nurse ☐

If you had another symptom you feel is relevant, please write it below, otherwise go to Question 7.

Please describe the symptom here and complete A then B or C below

6.

A When did you first notice this? ☐☐ / ☐☐ / ☐☐ **OR**

B When did you first tell your GP or nurse? ☐☐ / ☐☐ / ☐☐ **OR**

OR

C Put a cross here if you didn't tell your GP or nurse ☐

If you had further symptoms you would like to tell us about, there is space at the end of the Questionnaire for you to write them.

Did your GP send you for any tests?

Thinking back to the time immediately before you diagnosis, did your GP send you for any of the following tests?

Please cross the appropriate boxes

7. Were you sent for any of these?

	Yes	Not sure	No
Blood test(s)	☐	☐	☐
CT Scan	☐	☐	☐
Ultrasound scan	☐	☐	☐
Barium Enema	☐	☐	☐
Sigmoidoscopy or colonoscopy (Looking at bowel with internal camera)	☐	☐	☐
X-ray	☐	☐	☐

Please go to Question 8

About you

The next questions relate to general information about you. It would help us to know more about the background of the people completing the questionnaire.

8. Which best describes your employment status?

Please cross one box only

- Employed full-time ☐
- Employed part-time ☐
- Self employed full-time ☐
- Self employed part-time ☐
- Unemployed (seeking work) ☐
- Unemployed (not seeking work) ☐
- Retired ☐
- Student ☐
- Permanently sick/disabled ☐
- Temporarily sick/disabled ☐
- Looking after family/home ☐
- Other, please describe

9. What is your highest level of qualification?

Please cross one box only

- Degree (or equivalent) ☐
- Diploma (or equivalent) ☐
- 'A' level ☐
- GCSE / 'O' level ☐
- None ☐
- Other, please specify

10. **How would you describe your ethnicity**

Please cross one box only

White ☐
or
White British ☐
White Irish ☐
Other White background ☐

Mixed ☐
or
White & Black Carribean ☐
White & Black African ☐
White & Asian ☐
Other mixed background ☐

Black or Black British ☐
or
Carribean ☐
African ☐
Other Black background ☐

Asian or Asian British ☐
or
Indian ☐
Pakistani ☐
Bangladeshi ☐
Other Asian background ☐

Chinese or other ethnic group ☐
or
Chinese ☐
Other ethnic group ☐

11.

Do you live alone?

Yes ☐ **Please go to Question 12**

No ☐ **Please say who you live with below**

Who do you live with?
(e.g. wife, husband, partner, family member)

☐

12. **Are you suffering from, or have you suffered from, any of the following in the past 2 years?**

Please cross any that are applicable to you

- Asthma ☐
- Chronic Obstructive Pulmonary Disease (COPD) ☐
- Other lung disease (e.g. fibrosis, bronchiectatis etc) ☐
- Heart disease ☐
- Anxiety or depression ☐
- Inflammatory bowel disease ☐
- Irritable bowel syndrome ☐
- Peptic ulcer ☐
- Previous cancer ☐
- Diabetes ☐
- Arthritis ☐

13. **About smoking**

Please cross the appropriate statement

- Are you a current smoker? ☐
- Are you an ex-smoker? ☐
- Are you a non-smoker (never smoked)? ☐

14. **Do you think you were more at risk of getting cancer because of your family history?**

Yes ☐
No ☐

15. **The following questions are about how completing the questionnaire made you feel.**

A number of statements which people have used to describe themselves are given below. Read each statement and then cross the most appropriate box to indicate how you feel right now, at the moment. There are no right or wrong answers. Do not spend too much time on any one statement but give the answer which seems to describe your present feeling best.

		Not at all	Somewhat	Moderately	Very much
1.	I feel calm	☐	☐	☐	☐
2.	I am tense	☐	☐	☐	☐
3.	I feel upset	☐	☐	☐	☐
4.	I am relaxed	☐	☐	☐	☐
5.	I feel content	☐	☐	☐	☐
6.	I am worried	☐	☐	☐	☐

Please make sure you have answered all the questions.

If you have any other comments or there is anything else you would like to tell us about when you first noticed your symptoms, please do so using the space below.

If you feel you would like to talk to someone about this questionnaire at a later stage, please speak to the doctor or nurse who is looking after you, or be in touch with the research team, whose contact details are on your patient information sheet

Thank you for completing the questionnaire

Please give it to the research nurse

The next page is for the Researcher to complete

57727

3 3

Researcher - Post-completion Sheet

Researcher initials: ☐☐☐

Date questionnaire completed: Date ☐☐ / ☐☐ / ☐☐ Time ☐☐:☐☐

If not completed, give reason ☐

How long is it since the patient was told of his/her diagnosis? ☐

How long did it take the patient to complete the questionnaire?

- Less than 5 minutes ☐
- Between 5 and 10 minutes ☐
- Between 11 and 15 minutes ☐
- Longer than 15 minutes ☐

For the patient selected to complete the questionnaire on their own

Did the patient ask for any help? ☐ Yes ☐ No

If yes,

please specify what help was requested

____

____

please specify what help was given

____

____

For the patient selected for the researcher-administered questionnaire

Did the patient find any of the questions difficult? ☐ Yes ☐ No

If yes,

please specify which questions and what the difficulty was

____

____

____

____

Was the patient made anxious? ☐ Yes ☐ No

If yes,

please give details

____

____

____

____

Where did the patient complete the questionnaire?

(Please specify whether this was in a separate room alone, in a corner of the clinic, the ward or other

____

Thank you. Please now send the questionnaire and patient information sheet and consent form back to Cardiff University in the freepost envelope provided.

57727

PID - | | 3 | 4 |

Ymddiriedolaeth GIG Gogledd Orllewin Cymru
North West Wales NHS Trust

NHS WALES GIG CYMRU
Ymddiriedolaeth GIG Gogledd Cymru
North Wales NHS Trust

☐ GLAN CLWYD

CID - ☐ GWYNEDD

☐ MAELOR

Confidential Questionnaire

Looking At Your Symptoms Study

This Study has been funded by Cancer Research UK

Cardiff University / Prifysgol Caerdydd

Ysgol Glinigol Gogledd Cymru / North Wales Clinical School

Cancer Research UK

sewtu — South East Wales Trials Unit / Uned Ymchwil De-ddwyrain Cymru

Dear Patient

I am very grateful to you for agreeing to help us with this study.

I hope the information you supply will help us give people a diagnosis earlier.

If you feel you would like to talk to someone about this questionnaire at a later stage, please speak to the doctor or nurse who is looking after you, or be in touch with the research team, whose contact details are on your patient information sheet.

I can assure you that anything you tell us will be treated in the utmost confidence.

Yours sincerely

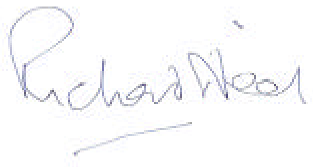

Dr Richard Neal
Senior Clinical Lecturer
Cardiff University
Chief Investigator, Symptoms Study

About your symptoms

These questions are about symptoms you noticed before your diagnosis. We are interested in symptoms which you think are related to your cancer.

Please put a cross in the appropriate box, or write an answer as required.

Looking back...

1. Did you have any symptoms that you feel were due to your cancer before you were diagnosed?

Yes ☐ **Please continue with Question 2**

No ☐ **Please go to Section 2 on Page 5**

2. What was the first symptom that made you think something might be wrong?

☐

Please go to Question 3

The following questions are about when you first noticed a symptom and when you first told your GP or nurse about it.

Please give an exact date if you can. Otherwise give your best estimate. (For example approximately how long ago, the month or season). You wish to refer to your diary or calendar if you have it with you.

Here is an example question:

Decrease in appetite

Did you have this symptom?

Yes ☒ **Please complete A then B or C below**

No ☐ **Please go to Section 2**

A When did you first notice this? d d / m m / y y **OR** Estimate '3 months ago' or 'June'

B When did you first tell your GP or nurse? 2 3 / 1 2 / 0 7 **OR** Estimate

OR

C Put a cross here if you didn't tell your GP or nurse ☐

3. Change in bowel habit

Did you have this symptom?

Yes ☐ **Please complete A then B or C below**

No ☐ **Please go to Question 4**

A When did you first notice this? ☐☐ / ☐☐ / ☐☐ **OR** ☐

B When did you first tell your GP or nurse? ☐☐ / ☐☐ / ☐☐ **OR** ☐

OR

C Put a cross here if you didn't tell your GP or nurse ☐

4. Bleeding from back passage

Did you have this symptom?

Yes ☐ **Please complete A then B or C below**

No ☐ **Please go to Section 2**

A When did you first notice this? ☐☐ / ☐☐ / ☐☐ **OR** ☐

B When did you first tell your GP or nurse? ☐☐ / ☐☐ / ☐☐ **OR** ☐

OR

C Put a cross here if you didn't tell your GP or nurse ☐

Section 2

Please give an exact date if you can. Otherwise give your best estimate (for example approximately how long ago, the month or the season). You may wish to refer to your diary or calendar if you have it with you.

Did you have any of the following?

1. Decrease in appetite

Did you have this?

Yes ☐ **Please complete A then B or C below**

No ☐ **Please go to Question 2**

A When did you first notice this? ☐☐ / ☐☐ / ☐☐ **OR** ☐

B When did you first tell your GP or nurse? ☐☐ / ☐☐ / ☐☐ **OR** ☐

OR

C Put a cross here if you didn't tell your GP or nurse ☐

2. Unexplained weight loss

Did you have this?

Yes ☐ **Please complete A then B or C below**

No ☐ **Please go to Question 3**

A When did you first notice this? ☐☐ / ☐☐ / ☐☐ **OR** ☐

B When did you first tell your GP or nurse? ☐☐ / ☐☐ / ☐☐ **OR** ☐

OR

C Put a cross here if you didn't tell your GP or nurse ☐

3. Fatigue or tiredness that is unusual for you

Did you have this?

Yes ☐ **Please complete A then B or C below**

No ☐ **Please go to Question 4**

A When did you <u>first notice</u> this? ☐☐ / ☐☐ / ☐☐ **OR** ☐

B When did you <u>first tell</u> your GP or nurse? ☐☐ / ☐☐ / ☐☐ **OR** ☐

OR

C Put a cross here if you <u>didn't tell</u> your GP or nurse ☐

4. Feeling different 'in yourself' from usual

Did you have this?

Yes ☐ **Please complete A then B or C below**

No ☐ **Please go to Question 5**

A When did you <u>first notice</u> this? ☐☐ / ☐☐ / ☐☐ **OR** ☐

B When did you <u>first tell</u> your GP or nurse? ☐☐ / ☐☐ / ☐☐ **OR** ☐

OR

C Put a cross here if you <u>didn't tell</u> your GP or nurse ☐

Please try and describe what the feeling was

If you had any further symptoms which you feel are relevant to your cancer diagnosis, please list them below. If not, please go to Question 7.

Please describe the symptom here and complete A then B or C below

5. ______________________

A When did you first notice this? __ __ / __ __ / __ __ **OR** ______

B When did you first tell your GP or nurse? __ __ / __ __ / __ __ **OR** ______

OR

C Put a cross here if you didn't tell your GP or nurse ☐

If you had another symptom you feel is relevant, please write it below, otherwise go to Question 7.

Please describe the symptom here and complete A then B or C below

6. ______________________

A When did you first notice this? __ __ / __ __ / __ __ **OR** ______

B When did you first tell your GP or nurse? __ __ / __ __ / __ __ **OR** ______

OR

C Put a cross here if you didn't tell your GP or nurse ☐

If you had further symptoms you would like to tell us about, there is space at the end of the Questionnaire for you to write them.

Did your GP send you for any tests?

Thinking back to the time immediately before you diagnosis, did your GP send you for any of the following tests?

Please cross the appropriate boxes

7. Were you sent for any of these?

	Yes	Not sure	No
Blood test(s)	☐	☐	☐
CT Scan	☐	☐	☐
Ultrasound scan	☐	☐	☐
Barium Enema	☐	☐	☐
Sigmoidoscopy or colonoscopy (Looking at bowel with internal camera)	☐	☐	☐
X-ray	☐	☐	☐

Please go to Question 8

About you

The next questions relate to general information about you. It would help us to know more about the background of the people completing the questionnaire.

8. Which best describes your employment status?

Please cross one box only

- Employed full-time ☐
- Employed part-time ☐
- Self employed full-time ☐
- Self employed part-time ☐
- Unemployed (seeking work) ☐
- Unemployed (not seeking work) ☐
- Retired ☐
- Student ☐
- Permanently sick/disabled ☐
- Temporarily sick/disabled ☐
- Looking after family/home ☐
- Other, please describe ____

9. What is your highest level of qualification?

Please cross one box only

- Degree (or equivalent) ☐
- Diploma (or equivalent) ☐
- 'A' level ☐
- GCSE / 'O' level ☐
- None ☐
- Other, please specify ____

10. **How would you describe your ethnicity**

Please cross one box only

White ☐
or
- White British ☐
- White Irish ☐
- Other White background ☐

Mixed ☐
or
- White & Black Carribean ☐
- White & Black African ☐
- White & Asian ☐
- Other mixed background ☐

Black or Black British ☐
or
- Carribean ☐
- African ☐
- Other Black background ☐

Asian or Asian British ☐
or
- Indian ☐
- Pakistani ☐
- Bangladeshi ☐
- Other Asian background ☐

Chinese or other ethnic group ☐
or
- Chinese ☐
- Other ethnic group ☐

11.

Do you live alone?

Yes ☐ **Please go to Question 12**

No ☐ **Please say who you live with below**

Who do you live with?
(e.g. wife, husband, partner, family member)

12. **Are you suffering from, or have you suffered from, any of the following in the past 2 years?**

Please cross any that are applicable to you

- Asthma ☐
- Chronic Obstructive Pulmonary Disease (COPD) ☐
- Other lung disease (e.g. fibrosis, bronchiectatis etc) ☐
- Heart disease ☐
- Anxiety or depression ☐
- Inflammatory bowel disease ☐
- Irritable bowel syndrome ☐
- Peptic ulcer ☐
- Previous cancer ☐
- Diabetes ☐
- Arthritis ☐

13. **About smoking**

Please cross the appropriate statement

- Are you a current smoker? ☐
- Are you an ex-smoker? ☐
- Are you a non-smoker (never smoked)? ☐

14. **Do you think you were more at risk of getting cancer because of your family history?**

Yes ☐
No ☐

15. The following questions are about how completing the questionnaire made you feel.

A number of statements which people have used to describe themselves are given below. Read each statement and then cross the most appropriate box to indicate how you feel <u>right now, at the moment.</u> There are no right or wrong answers. Do not spend too much time on any one statement but give the answer which seems to describe your present feeling best.

		Not at all	Somewhat	Moderately	Very much
1.	I feel calm	☐	☐	☐	☐
2.	I am tense	☐	☐	☐	☐
3.	I feel upset	☐	☐	☐	☐
4.	I am relaxed	☐	☐	☐	☐
5.	I feel content	☐	☐	☐	☐
6.	I am worried	☐	☐	☐	☐

Please make sure you have answered all the questions.

If you have any other comments or there is anything else you would like to tell us about when you first noticed your symptoms, please do so using the space below.

If you feel you would like to talk to someone about this questionnaire at a later stage, please speak to the doctor or nurse who is looking after you, or be in touch with the research team, whose contact details are on your patient information sheet

Thank you for completing the questionnaire

Please give it to the research nurse

The next page is for the Researcher to complete

Researcher - Post-completion Sheet **Researcher initials:** ☐☐☐

Date questionnaire completed: Date ☐☐ / ☐☐ / ☐☐ Time ☐☐:☐☐

If not completed, give reason ☐

How long is it since the patient was told of his/her diagnosis? ☐

How long did it take the patient to complete the questionnaire?

- Less than 5 minutes ☐
- Between 5 and 10 minutes ☐
- Between 11 and 15 minutes ☐
- Longer than 15 minutes ☐

For the patient selected to complete the questionnaire on their own

Did the patient ask for any help? ☐ Yes ☐ No

If yes,

please specify what help was requested

____

____

please specify what help was given

____

____

For the patient selected for the researcher-administered questionnaire

Did the patient find any of the questions difficult? ☐ Yes ☐ No

If yes,

please specify which questions and what the difficulty was

____

____

____

____

Was the patient made anxious? ☐ Yes ☐ No

If yes,

please give details

____

____

____

____

Where did the patient complete the questionnaire?
(Please specify whether this was in a separate room alone, in a corner of the clinic, the ward or other

____

Thank you. Please now send the questionnaire and patient information sheet and consent form back to Cardiff University in the freepost envelope provided.

57727

PID - | | 3 | 5 |

Ymddiriedolaeth GIG Gogledd Orllewin Cymru
North West Wales NHS Trust

NHS WALES GIG CYMRU
Ymddiriedolaeth GIG Gogledd Cymru
North Wales NHS Trust

CID -
☐ GLAN CLWYD
☐ GWYNEDD
☐ MAELOR

Confidential Questionnaire

Looking At Your Symptoms Study

This Study has been funded by Cancer Research UK

Cardiff University / Prifysgol Caerdydd
Ysgol Glinigol Gogledd Cymru / North Wales Clinical School
Cancer Research UK
sewtu South East Wales Trials Unit / Uned Ymchwil De-ddwyrain Cymru

Dear Patient

I am very grateful to you for agreeing to help us with this study.

I hope the information you supply will help us give people a diagnosis earlier.

If you feel you would like to talk to someone about this questionnaire at a later stage, please speak to the doctor or nurse who is looking after you, or be in touch with the research team, whose contact details are on your patient information sheet.

I can assure you that anything you tell us will be treated in the utmost confidence.

Yours sincerely

Dr Richard Neal
Senior Clinical Lecturer
Cardiff University
Chief Investigator, Symptoms Study

About your symptoms

These questions are about symptoms you noticed before your diagnosis. We are interested in symptoms which you think are related to your cancer.

Please put a cross in the appropriate box, or write an answer as required.

Looking back...

1. Did you have any symptoms that you feel were due to your cancer before you were diagnosed?

Yes ☐ **Please continue with Question 2**

No ☐ **Please go to Section 2 on Page 5**

2. What was the first symptom that made you think something might be wrong?

☐

Please go to Question 3

The following questions are about when you first noticed a symptom and when you first told your GP or nurse about it.

Please give an exact date if you can. Otherwise give your best estimate. (For example approximately how long ago, the month or season). You wish to refer to your diary or calendar if you have it with you.

Here is an example question:

Decrease in appetite

Did you have this symptom?

Yes ☒ **Please complete A then B or C below**

No ☐ **Please go to Section 2**

A When did you first notice this? d d / m m / y y **OR** Estimate '3 months ago' or 'June'

B When did you first tell your GP or nurse? 2 3 / 1 2 / 0 7 **OR** Estimate

OR

C Put a cross here if you didn't tell your GP or nurse ☐

3. Change in bowel habit

Did you have this symptom?

Yes ☐ **Please complete A then B or C below**

No ☐ **Please go to Question 4**

A When did you first notice this? ☐☐ / ☐☐ / ☐☐ **OR** ☐

B When did you first tell your GP or nurse? ☐☐ / ☐☐ / ☐☐ **OR** ☐

OR

C Put a cross here if you didn't tell your GP or nurse ☐

4. Bleeding from back passage

Did you have this symptom?

Yes ☐ **Please complete A then B or C below**

No ☐ **Please go to Section 2**

A When did you first notice this? ☐☐ / ☐☐ / ☐☐ **OR** ☐

B When did you first tell your GP or nurse? ☐☐ / ☐☐ / ☐☐ **OR** ☐

OR

C Put a cross here if you didn't tell your GP or nurse ☐

Section 2

Please give an exact date if you can. Otherwise give your best estimate (for example approximately how long ago, the month or the season). You may wish to refer to your diary or calendar if you have it with you.

Did you have any of the following?

1. Decrease in appetite

Did you have this?

Yes ☐ **Please complete A then B or C below**

No ☐ **Please go to Question 2**

A When did you first notice this? ☐☐ / ☐☐ / ☐☐ **OR** ☐

B When did you first tell your GP or nurse? ☐☐ / ☐☐ / ☐☐ **OR** ☐

OR

C Put a cross here if you didn't tell your GP or nurse ☐

2. Unexplained weight loss

Did you have this?

Yes ☐ **Please complete A then B or C below**

No ☐ **Please go to Question 3**

A When did you first notice this? ☐☐ / ☐☐ / ☐☐ **OR** ☐

B When did you first tell your GP or nurse? ☐☐ / ☐☐ / ☐☐ **OR** ☐

OR

C Put a cross here if you didn't tell your GP or nurse ☐

3. Fatigue or tiredness that is unusual for you

Did you have this?

Yes ☐ **Please complete A then B or C below**

No ☐ **Please go to Question 4**

A When did you first notice this? ☐☐ / ☐☐ / ☐☐ **OR** ☐

B When did you first tell your GP or nurse? ☐☐ / ☐☐ / ☐☐ **OR** ☐

OR

C Put a cross here if you didn't tell your GP or nurse ☐

4. Feeling different 'in yourself' from usual

Did you have this?

Yes ☐ **Please complete A then B or C below**

No ☐ **Please go to Question 5**

A When did you first notice this? ☐☐ / ☐☐ / ☐☐ **OR** ☐

B When did you first tell your GP or nurse? ☐☐ / ☐☐ / ☐☐ **OR** ☐

OR

C Put a cross here if you didn't tell your GP or nurse ☐

Please try and describe what the feeling was

If you had any further symptoms which you feel are relevant to your cancer diagnosis, please list them below. If not, please go to Question 7.

Please describe the symptom here and complete A then B or C below

5.

**A**	When did you first notice this?	☐☐ / ☐☐ / ☐☐ **OR** ☐
**B**	When did you first tell your GP or nurse?	☐☐ / ☐☐ / ☐☐ **OR** ☐
	**OR**	
**C**	Put a cross here if you didn't tell your GP or nurse	☐

If you had another symptom you feel is relevant, please write it below, otherwise go to Question 7.

Please describe the symptom here and complete A then B or C below

6.

**A**	When did you first notice this?	☐☐ / ☐☐ / ☐☐ **OR** ☐
**B**	When did you first tell your GP or nurse?	☐☐ / ☐☐ / ☐☐ **OR** ☐
	**OR**	
**C**	Put a cross here if you didn't tell your GP or nurse	☐

If you had further symptoms you would like to tell us about, there is space at the end of the Questionnaire for you to write them.

Did your GP send you for any tests?

Thinking back to the time immediately before you diagnosis, did your GP send you for any of the following tests?

Please cross the appropriate boxes

7. Were you sent for any of these?

	Yes	Not sure	No
Blood test(s)	☐	☐	☐
CT Scan	☐	☐	☐
Ultrasound scan	☐	☐	☐
Barium Enema	☐	☐	☐
Sigmoidoscopy or colonoscopy (Looking at bowel with internal camera)	☐	☐	☐
X-ray	☐	☐	☐

Please go to Question 8

About you

The next questions relate to general information about you. It would help us to know more about the background of the people completing the questionnaire.

8. Which best describes your employment status?

Please cross one box only

- Employed full-time ☐
- Employed part-time ☐
- Self employed full-time ☐
- Self employed part-time ☐
- Unemployed (seeking work) ☐
- Unemployed (not seeking work) ☐
- Retired ☐
- Student ☐
- Permanently sick/disabled ☐
- Temporarily sick/disabled ☐
- Looking after family/home ☐
- Other, please describe ____________

9. What is your highest level of qualification?

Please cross one box only

- Degree (or equivalent) ☐
- Diploma (or equivalent) ☐
- 'A' level ☐
- GCSE / 'O' level ☐
- None ☐
- Other, please specify ____________

10. How would you describe your ethnicity

Please cross one box only

White ☐
or
- White British ☐
- White Irish ☐
- Other White background ☐

Mixed ☐
or
- White & Black Carribean ☐
- White & Black African ☐
- White & Asian ☐
- Other mixed background ☐

Black or Black British ☐
or
- Carribean ☐
- African ☐
- Other Black background ☐

Asian or Asian British ☐
or
- Indian ☐
- Pakistani ☐
- Bangladeshi ☐
- Other Asian background ☐

Chinese or other ethnic group ☐
or
- Chinese ☐
- Other ethnic group ☐

11.

Do you live alone?

Yes ☐ **Please go to Question 12**

No ☐ **Please say who you live with below**

Who do you live with?
(e.g. wife, husband, partner, family member)

☐

12. **Are you suffering from, or have you suffered from, any of the following in the past 2 years?**

Please cross any that are applicable to you

- Asthma ☐
- Chronic Obstructive Pulmonary Disease (COPD) ☐
- Other lung disease (e.g. fibrosis, bronchiectatis etc) ☐
- Heart disease ☐
- Anxiety or depression ☐
- Inflammatory bowel disease ☐
- Irritable bowel syndrome ☐
- Peptic ulcer ☐
- Previous cancer ☐
- Diabetes ☐
- Arthritis ☐

13. **About smoking**

Please cross the appropriate statement

- Are you a current smoker? ☐
- Are you an ex-smoker? ☐
- Are you a non-smoker (never smoked)? ☐

14. **Do you think you were more at risk of getting cancer because of your family history?**

Yes ☐
No ☐

15. **The following questions are about how completing the questionnaire made you feel.**

A number of statements which people have used to describe themselves are given below. Read each statement and then cross the most appropriate box to indicate how you feel right now, at the moment. There are no right or wrong answers. Do not spend too much time on any one statement but give the answer which seems to describe your present feeling best.

		Not at all	Somewhat	Moderately	Very much
1.	I feel calm	☐	☐	☐	☐
2.	I am tense	☐	☐	☐	☐
3.	I feel upset	☐	☐	☐	☐
4.	I am relaxed	☐	☐	☐	☐
5.	I feel content	☐	☐	☐	☐
6.	I am worried	☐	☐	☐	☐

Please make sure you have answered all the questions.

If you have any other comments or there is anything else you would like to tell us about when you first noticed your symptoms, please do so using the space below.

If you feel you would like to talk to someone about this questionnaire at a later stage, please speak to the doctor or nurse who is looking after you, or be in touch with the research team, whose contact details are on your patient information sheet

Thank you for completing the questionnaire

Please give it to the research nurse

The next page is for the Researcher to complete

Researcher - Post-completion Sheet **Researcher initials:** ☐☐☐

Date questionnaire completed: Date ☐☐ / ☐☐ / ☐☐ Time ☐☐:☐☐

If not completed, give reason ☐

How long is it since the patient was told of his/her diagnosis? ☐

How long did it take the patient to complete the questionnaire?

- Less than 5 minutes ☐
- Between 5 and 10 minutes ☐
- Between 11 and 15 minutes ☐
- Longer than 15 minutes ☐

For the patient selected to complete the questionnaire on their own

Did the patient ask for any help? ☐ Yes ☐ No

If yes,

please specify what help was requested

____

____

please specify what help was given

____

____

For the patient selected for the researcher-administered questionnaire

Did the patient find any of the questions difficult? ☐ Yes ☐ No

If yes,

please specify which questions and what the difficulty was

____

____

____

____

Was the patient made anxious? ☐ Yes ☐ No

If yes,

please give details

____

____

____

____

Where did the patient complete the questionnaire?

(Please specify whether this was in a separate room alone, in a corner of the clinic, the ward or other

____

Thank you. Please now send the questionnaire and patient information sheet and consent form back to Cardiff University in the freepost envelope provided.

57727

PID - | 3 | 6 |

Ymddiriedolaeth GIG Gogledd Orllewin Cymru
North West Wales NHS Trust

NHS WALES GIG CYMRU
Ymddiriedolaeth GIG Gogledd Cymru
North Wales NHS Trust

☐ GLAN CLWYD

CID - ☐ GWYNEDD

☐ MAELOR

Confidential Questionnaire

Looking At Your Symptoms Study

This Study has been funded by Cancer Research UK

Cardiff University Prifysgol Caerdydd

Ysgol Glinigol Gogledd Cymru
North Wales Clinical School

Cancer Research UK

sewtu
South East Wales Trials Unit
Uned Ymchwil De-ddwyrain Cymru

Dear Patient

I am very grateful to you for agreeing to help us with this study.

I hope the information you supply will help us give people a diagnosis earlier.

If you feel you would like to talk to someone about this questionnaire at a later stage, please speak to the doctor or nurse who is looking after you, or be in touch with the research team, whose contact details are on your patient information sheet.

I can assure you that anything you tell us will be treated in the utmost confidence.

Yours sincerely

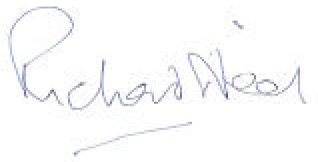

Dr Richard Neal
Senior Clinical Lecturer
Cardiff University
Chief Investigator, Symptoms Study

About your symptoms

These questions are about symptoms you noticed before your diagnosis. We are interested in symptoms which you think are related to your cancer.

Please put a cross in the appropriate box, or write an answer as required.

Looking back...

1. Did you have any symptoms that you feel were due to your cancer before you were diagnosed?

Yes ☐ **Please continue with Question 2**

No ☐ **Please go to Section 2 on Page 5**

2. What was the first symptom that made you think something might be wrong?

☐

Please go to Question 3

The following questions are about when you first noticed a symptom and when you first told your GP or nurse about it.

Please give an exact date if you can. Otherwise give your best estimate. (For example approximately how long ago, the month or season). You wish to refer to your diary or calendar if you have it with you.

Here is an example question:

Decrease in appetite

Did you have this symptom?

Yes ☒ **Please complete A then B or C below**

No ☐ **Please go to Section 2**

A When did you first notice this? d d / m m / y y **OR** Estimate: '3 months ago' or 'June'

B When did you first tell your GP or nurse? 2 3 / 1 2 / 0 7 **OR** Estimate

OR

C Put a cross here if you didn't tell your GP or nurse ☐

3. Change in bowel habit

Did you have this symptom?

Yes ☐ **Please complete A then B or C below**

No ☐ **Please go to Question 4**

A When did you first notice this? ☐☐ / ☐☐ / ☐☐ **OR** ☐

B When did you first tell your GP or nurse? ☐☐ / ☐☐ / ☐☐ **OR** ☐

OR

C Put a cross here if you didn't tell your GP or nurse ☐

4. Bleeding from back passage

Did you have this symptom?

Yes ☐ **Please complete A then B or C below**

No ☐ **Please go to Section 2**

A When did you first notice this? ☐☐ / ☐☐ / ☐☐ **OR** ☐

B When did you first tell your GP or nurse? ☐☐ / ☐☐ / ☐☐ **OR** ☐

OR

C Put a cross here if you didn't tell your GP or nurse ☐

Section 2

Please give an exact date if you can. Otherwise give your best estimate (for example approximately how long ago, the month or the season). You may wish to refer to your diary or calendar if you have it with you.

Did you have any of the following?

1. Decrease in appetite

Did you have this?

Yes ☐ **Please complete A then B or C below**

No ☐ **Please go to Question 2**

A When did you first notice this? ☐☐ / ☐☐ / ☐☐ **OR** ☐

B When did you first tell your GP or nurse? ☐☐ / ☐☐ / ☐☐ **OR** ☐

OR

C Put a cross here if you didn't tell your GP or nurse ☐

2. Unexplained weight loss

Did you have this?

Yes ☐ **Please complete A then B or C below**

No ☐ **Please go to Question 3**

A When did you first notice this? ☐☐ / ☐☐ / ☐☐ **OR** ☐

B When did you first tell your GP or nurse? ☐☐ / ☐☐ / ☐☐ **OR** ☐

OR

C Put a cross here if you didn't tell your GP or nurse ☐

3. **Fatigue or tiredness that is unusual for you**

Did you have this?

Yes ☐ **Please complete A then B or C below**

No ☐ **Please go to Question 4**

A When did you <u>first notice</u> this? ☐☐ / ☐☐ / ☐☐ **OR** ☐

B When did you <u>first tell</u> your GP or nurse? ☐☐ / ☐☐ / ☐☐ **OR** ☐

OR

C Put a cross here if you <u>didn't tell</u> your GP or nurse ☐

4. **Feeling different 'in yourself' from usual**

Did you have this?

Yes ☐ **Please complete A then B or C below**

No ☐ **Please go to Question 5**

A When did you <u>first notice</u> this? ☐☐ / ☐☐ / ☐☐ **OR** ☐

B When did you <u>first tell</u> your GP or nurse? ☐☐ / ☐☐ / ☐☐ **OR** ☐

OR

C Put a cross here if you <u>didn't tell</u> your GP or nurse ☐

Please try and describe what the feeling was

If you had any further symptoms which you feel are relevant to your cancer diagnosis, please list them below. If not, please go to Question 7.

Please describe the symptom here and complete A then B or C below

5.

**A**	When did you <u>first notice</u> this?	__ __ / __ __ / __ __ **OR** ☐
**B**	When did you <u>first tell</u> your GP or nurse?	__ __ / __ __ / __ __ **OR** ☐
	**OR**	
**C**	Put a cross here if you <u>didn't tell</u> your GP or nurse	☐

If you had another symptom you feel is relevant, please write it below, otherwise go to Question 7.

Please describe the symptom here and complete A then B or C below

6.

**A**	When did you <u>first notice</u> this?	__ __ / __ __ / __ __ **OR** ☐
**B**	When did you <u>first tell</u> your GP or nurse?	__ __ / __ __ / __ __ **OR** ☐
	**OR**	
**C**	Put a cross here if you <u>didn't tell</u> your GP or nurse	☐

If you had further symptoms you would like to tell us about, there is space at the end of the Questionnaire for you to write them.

Did your GP send you for any tests?

Thinking back to the time immediately before you diagnosis, did your GP send you for any of the following tests?

Please cross the appropriate boxes

7. Were you sent for any of these?

	Yes	Not sure	No
Blood test(s)	☐	☐	☐
CT Scan	☐	☐	☐
Ultrasound scan	☐	☐	☐
Barium Enema	☐	☐	☐
Sigmoidoscopy or colonoscopy (Looking at bowel with internal camera)	☐	☐	☐
X-ray	☐	☐	☐

Please go to Question 8

About you

The next questions relate to general information about you. It would help us to know more about the background of the people completing the questionnaire.

8. Which best describes your employment status?

Please cross one box only

- Employed full-time ☐
- Employed part-time ☐
- Self employed full-time ☐
- Self employed part-time ☐
- Unemployed (seeking work) ☐
- Unemployed (not seeking work) ☐
- Retired ☐
- Student ☐
- Permanently sick/disabled ☐
- Temporarily sick/disabled ☐
- Looking after family/home ☐
- Other, please describe ☐

9. What is your highest level of qualification?

Please cross one box only

- Degree (or equivalent) ☐
- Diploma (or equivalent) ☐
- 'A' level ☐
- GCSE / 'O' level ☐
- None ☐
- Other, please specify ☐

10. **How would you describe your ethnicity**

Please cross one box only

White ☐
or
White British ☐
White Irish ☐
Other White background ☐

Mixed ☐
or
White & Black Carribean ☐
White & Black African ☐
White & Asian ☐
Other mixed background ☐

Black or Black British ☐
or
Carribean ☐
African ☐
Other Black background ☐

Asian or Asian British ☐
or
Indian ☐
Pakistani ☐
Bangladeshi ☐
Other Asian background ☐

Chinese or other ethnic group ☐
or
Chinese ☐
Other ethnic group ☐

11.

Do you live alone?

Yes ☐ **Please go to Question 12**

No ☐ **Please say who you live with below**

Who do you live with?
(e.g. wife, husband, partner, family member)

12. **Are you suffering from, or have you suffered from, any of the following in the past 2 years?**

Please cross any that are applicable to you

- Asthma ☐
- Chronic Obstructive Pulmonary Disease (COPD) ☐
- Other lung disease (e.g. fibrosis, bronchiectatis etc) ☐
- Heart disease ☐
- Anxiety or depression ☐
- Inflammatory bowel disease ☐
- Irritable bowel syndrome ☐
- Peptic ulcer ☐
- Previous cancer ☐
- Diabetes ☐
- Arthritis ☐

13. **About smoking**

Please cross the appropriate statement

- Are you a current smoker? ☐
- Are you an ex-smoker? ☐
- Are you a non-smoker (never smoked)? ☐

14. **Do you think you were more at risk of getting cancer because of your family history?**

Yes ☐

No ☐

15. **The following questions are about how completing the questionnaire made you feel.**

A number of statements which people have used to describe themselves are given below. Read each statement and then cross the most appropriate box to indicate how you feel right now, at the moment. There are no right or wrong answers. Do not spend too much time on any one statement but give the answer which seems to describe your present feeling best.

		Not at all	Somewhat	Moderately	Very much
1.	I feel calm	☐	☐	☐	☐
2.	I am tense	☐	☐	☐	☐
3.	I feel upset	☐	☐	☐	☐
4.	I am relaxed	☐	☐	☐	☐
5.	I feel content	☐	☐	☐	☐
6.	I am worried	☐	☐	☐	☐

Please make sure you have answered all the questions.

If you have any other comments or there is anything else you would like to tell us about when you first noticed your symptoms, please do so using the space below.

If you feel you would like to talk to someone about this questionnaire at a later stage, please speak to the doctor or nurse who is looking after you, or be in touch with the research team, whose contact details are on your patient information sheet

Thank you for completing the questionnaire

Please give it to the research nurse

The next page is for the Researcher to complete

57727

3 6

Researcher - Post-completion Sheet

Researcher initials: ☐☐☐

Date questionnaire completed: Date ☐☐ / ☐☐ / ☐☐ Time ☐☐:☐☐

If not completed, give reason ☐

How long is it since the patient was told of his/her diagnosis? ☐

How long did it take the patient to complete the questionnaire?

- Less than 5 minutes ☐
- Between 5 and 10 minutes ☐
- Between 11 and 15 minutes ☐
- Longer than 15 minutes ☐

For the patient selected to complete the questionnaire on their own

Did the patient ask for any help? ☐ Yes ☐ No

If yes,

please specify what help was requested

____

____

please specify what help was given

____

____

For the patient selected for the researcher-administered questionnaire

Did the patient find any of the questions difficult? ☐ Yes ☐ No

If yes,

please specify which questions and what the difficulty was

____

____

____

____

Was the patient made anxious? ☐ Yes ☐ No

If yes,

please give details

____

____

____

____

Where did the patient complete the questionnaire?
(Please specify whether this was in a separate room alone, in a corner of the clinic, the ward or other

____

Thank you. Please now send the questionnaire and patient information sheet and consent form back to Cardiff University in the freepost envelope provided.

57727

PID - | | 3 | 7 |

Ymddiriedolaeth GIG Gogledd Orllewin Cymru
North West Wales NHS Trust

NHS WALES GIG CYMRU
Ymddiriedolaeth GIG Gogledd Cymru
North Wales NHS Trust

CID - ☐ GLAN CLWYD
☐ GWYNEDD
☐ MAELOR

Confidential Questionnaire

Looking At Your Symptoms Study

This Study has been funded by Cancer Research UK

CARDIFF UNIVERSITY PRIFYSGOL CAERDYDD

Ysgol Glinigol Gogledd Cymru
North Wales Clinical School

CANCER RESEARCH UK

sewtu South East Wales Trials Unit
Uned Ymchwil De-ddwyrain Cymru

Dear Patient

I am very grateful to you for agreeing to help us with this study.

I hope the information you supply will help us give people a diagnosis earlier.

If you feel you would like to talk to someone about this questionnaire at a later stage, please speak to the doctor or nurse who is looking after you, or be in touch with the research team, whose contact details are on your patient information sheet.

I can assure you that anything you tell us will be treated in the utmost confidence.

Yours sincerely

Dr Richard Neal
Senior Clinical Lecturer
Cardiff University
Chief Investigator, Symptoms Study

About your symptoms

These questions are about symptoms you noticed before your diagnosis. We are interested in symptoms which you think are related to your cancer.

Please put a cross in the appropriate box, or write an answer as required.

Looking back...

1. Did you have any symptoms that you feel were due to your cancer before you were diagnosed?

Yes ☐ **Please continue with Question 2**

No ☐ **Please go to Section 2 on Page 5**

2. What was the first symptom that made you think something might be wrong?

☐

Please go to Question 3

The following questions are about when you first noticed a symptom and when you first told your GP or nurse about it.

Please give an exact date if you can. Otherwise give your best estimate. (For example approximately how long ago, the month or season). You wish to refer to your diary or calendar if you have it with you.

Here is an example question:

Decrease in appetite

Did you have this symptom?

Yes ☒ **Please complete A then B or C below**

No ☐ **Please go to Section 2**

A When did you first notice this? d d / m m / y y **OR** Estimate '3 months ago' or 'June'

B When did you first tell your GP or nurse? 2 3 / 1 2 / 0 7 **OR** Estimate

OR

C Put a cross here if you didn't tell your GP or nurse ☐

3. Change in bowel habit

Did you have this symptom?

Yes ☐ **Please complete A then B or C below**

No ☐ **Please go to Question 4**

A When did you <u>first notice</u> this? __ __ / __ __ / __ __ **OR** ______

B When did you <u>first tell</u> your GP or nurse? __ __ / __ __ / __ __ **OR** ______

OR

C Put a cross here if you <u>didn't tell</u> your GP or nurse ☐

4. Bleeding from back passage

Did you have this symptom?

Yes ☐ **Please complete A then B or C below**

No ☐ **Please go to Section 2**

A When did you <u>first notice</u> this? __ __ / __ __ / __ __ **OR** ______

B When did you <u>first tell</u> your GP or nurse? __ __ / __ __ / __ __ **OR** ______

OR

C Put a cross here if you <u>didn't tell</u> your GP or nurse ☐

Please give an exact date if you can. Otherwise give your best estimate (for example approximately how long ago, the month or the season). You may wish to refer to your diary or calendar if you have it with you.

Did you have any of the following?

1. Decrease in appetite

Did you have this?

Yes ☐ **Please complete A then B or C below**

No ☐ **Please go to Question 2**

A When did you first notice this? ☐☐ / ☐☐ / ☐☐ **OR** ☐

B When did you first tell your GP or nurse? ☐☐ / ☐☐ / ☐☐ **OR** ☐

OR

C Put a cross here if you didn't tell your GP or nurse ☐

2. Unexplained weight loss

Did you have this?

Yes ☐ **Please complete A then B or C below**

No ☐ **Please go to Question 3**

A When did you first notice this? ☐☐ / ☐☐ / ☐☐ **OR** ☐

B When did you first tell your GP or nurse? ☐☐ / ☐☐ / ☐☐ **OR** ☐

OR

C Put a cross here if you didn't tell your GP or nurse ☐

3. Fatigue or tiredness that is unusual for you

Did you have this?

Yes ☐ **Please complete A then B or C below**

No ☐ **Please go to Question 4**

A When did you <u>first notice</u> this? ☐☐ / ☐☐ / ☐☐ **OR** ☐

B When did you <u>first tell</u> your GP or nurse? ☐☐ / ☐☐ / ☐☐ **OR** ☐

OR

C Put a cross here if you <u>didn't tell</u> your GP or nurse ☐

4. Feeling different 'in yourself' from usual

Did you have this?

Yes ☐ **Please complete A then B or C below**

No ☐ **Please go to Question 5**

A When did you <u>first notice</u> this? ☐☐ / ☐☐ / ☐☐ **OR** ☐

B When did you <u>first tell</u> your GP or nurse? ☐☐ / ☐☐ / ☐☐ **OR** ☐

OR

C Put a cross here if you <u>didn't tell</u> your GP or nurse ☐

Please try and describe what the feeling was

If you had any further symptoms which you feel are relevant to your cancer diagnosis, please list them below. If not, please go to Question 7.

Please describe the symptom here and complete A then B or C below

5.

**A**	When did you first notice this?	☐☐ / ☐☐ / ☐☐ **OR** ☐
**B**	When did you first tell your GP or nurse?	☐☐ / ☐☐ / ☐☐ **OR** ☐
	**OR**	
**C**	Put a cross here if you didn't tell your GP or nurse	☐

If you had another symptom you feel is relevant, please write it below, otherwise go to Question 7.

Please describe the symptom here and complete A then B or C below

6.

**A**	When did you first notice this?	☐☐ / ☐☐ / ☐☐ **OR** ☐
**B**	When did you first tell your GP or nurse?	☐☐ / ☐☐ / ☐☐ **OR** ☐
	**OR**	
**C**	Put a cross here if you didn't tell your GP or nurse	☐

If you had further symptoms you would like to tell us about, there is space at the end of the Questionnaire for you to write them.

Did your GP send you for any tests?

Thinking back to the time immediately before you diagnosis, did your GP send you for any of the following tests?

Please cross the appropriate boxes

7. Were you sent for any of these?

	Yes	Not sure	No
Blood test(s)	☐	☐	☐
CT Scan	☐	☐	☐
Ultrasound scan	☐	☐	☐
Barium Enema	☐	☐	☐
Sigmoidoscopy or colonoscopy (Looking at bowel with internal camera)	☐	☐	☐
X-ray	☐	☐	☐

Please go to Question 8

About you

The next questions relate to general information about you. It would help us to know more about the background of the people completing the questionnaire.

8. Which best describes your employment status?

Please cross one box only

- Employed full-time ☐
- Employed part-time ☐
- Self employed full-time ☐
- Self employed part-time ☐
- Unemployed (seeking work) ☐
- Unemployed (not seeking work) ☐
- Retired ☐
- Student ☐
- Permanently sick/disabled ☐
- Temporarily sick/disabled ☐
- Looking after family/home ☐
- Other, please describe ☐

9. What is your highest level of qualification?

Please cross one box only

- Degree (or equivalent) ☐
- Diploma (or equivalent) ☐
- 'A' level ☐
- GCSE / 'O' level ☐
- None ☐
- Other, please specify ☐

10. How would you describe your ethnicity

Please cross one box only

White ☐
or
- White British ☐
- White Irish ☐
- Other White background ☐

Mixed ☐
or
- White & Black Carribean ☐
- White & Black African ☐
- White & Asian ☐
- Other mixed background ☐

Black or Black British ☐
or
- Carribean ☐
- African ☐
- Other Black background ☐

Asian or Asian British ☐
or
- Indian ☐
- Pakistani ☐
- Bangladeshi ☐
- Other Asian background ☐

Chinese or other ethnic group ☐
or
- Chinese ☐
- Other ethnic group ☐

11.

Do you live alone?

Yes ☐ **Please go to Question 12**

No ☐ **Please say who you live with below**

Who do you live with?
(e.g. wife, husband, partner, family member)

☐

12. **Are you suffering from, or have you suffered from, any of the following in the past 2 years?**

Please cross any that are applicable to you

Asthma	☐
Chronic Obstructive Pulmonary Disease (COPD)	☐
Other lung disease (e.g. fibrosis, bronchiectatis etc)	☐
Heart disease	☐
Anxiety or depression	☐
Inflammatory bowel disease	☐
Irritable bowel syndrome	☐
Peptic ulcer	☐
Previous cancer	☐
Diabetes	☐
Arthritis	☐

13. **About smoking**

Please cross the appropriate statement

Are you a current smoker?	☐
Are you an ex-smoker?	☐
Are you a non-smoker (never smoked)?	☐

14. **Do you think you were more at risk of getting cancer because of your family history?**

Yes ☐

No ☐

15. **The following questions are about how completing the questionnaire made you feel.**

A number of statements which people have used to describe themselves are given below. Read each statement and then cross the most appropriate box to indicate how you feel right now, at the moment. There are no right or wrong answers. Do not spend too much time on any one statement but give the answer which seems to describe your present feeling best.

		Not at all	Somewhat	Moderately	Very much
1.	I feel calm	☐	☐	☐	☐
2.	I am tense	☐	☐	☐	☐
3.	I feel upset	☐	☐	☐	☐
4.	I am relaxed	☐	☐	☐	☐
5.	I feel content	☐	☐	☐	☐
6.	I am worried	☐	☐	☐	☐

Please make sure you have answered all the questions.

If you have any other comments or there is anything else you would like to tell us about when you first noticed your symptoms, please do so using the space below.

If you feel you would like to talk to someone about this questionnaire at a later stage, please speak to the doctor or nurse who is looking after you, or be in touch with the research team, whose contact details are on your patient information sheet

Thank you for completing the questionnaire

Please give it to the research nurse

The next page is for the Researcher to complete

Researcher - Post-completion Sheet

Researcher initials: ☐☐☐

Date questionnaire completed: Date ☐☐ / ☐☐ / ☐☐ Time ☐☐:☐☐

If not completed, give reason ☐

How long is it since the patient was told of his/her diagnosis? ☐

How long did it take the patient to complete the questionnaire?

- Less than 5 minutes ☐
- Between 5 and 10 minutes ☐
- Between 11 and 15 minutes ☐
- Longer than 15 minutes ☐

For the patient selected to <u>complete the questionnaire on their own</u>

Did the patient ask for any help? ☐ Yes ☐ No

If yes,

please specify what help was requested

____

____

please specify what help was given

____

____

For the patient selected for <u>the researcher-administered questionnaire</u>

Did the patient find any of the questions difficult? ☐ Yes ☐ No

If yes,

please specify which questions and what the difficulty was

____

____

____

____

Was the patient made anxious? ☐ Yes ☐ No

If yes,

please give details

____

____

____

____

Where did the patient complete the questionnaire?

(Please specify whether this was in a separate room alone, in a corner of the clinic, the ward or other

____

Thank you. Please now send the questionnaire and patient information sheet and consent form back to Cardiff University in the freepost envelope provided.

57727

PID - 38

Ymddiriedolaeth GIG Gogledd Orllewin Cymru
North West Wales NHS Trust

NHS WALES GIG CYMRU
Ymddiriedolaeth GIG Gogledd Cymru
North Wales NHS Trust

☐ GLAN CLWYD

CID - ☐ GWYNEDD

☐ MAELOR

Confidential Questionnaire

Looking At Your Symptoms Study

This Study has been funded by Cancer Research UK

Cardiff University / Prifysgol Caerdydd
Ysgol Glinigol Gogledd Cymru / North Wales Clinical School
Cancer Research UK
sewtu — South East Wales Trials Unit / Uned Ymchwil De-ddwyrain Cymru

Dear Patient

I am very grateful to you for agreeing to help us with this study.

I hope the information you supply will help us give people a diagnosis earlier.

If you feel you would like to talk to someone about this questionnaire at a later stage, please speak to the doctor or nurse who is looking after you, or be in touch with the research team, whose contact details are on your patient information sheet.

I can assure you that anything you tell us will be treated in the utmost confidence.

Yours sincerely

Dr Richard Neal
Senior Clinical Lecturer
Cardiff University
Chief Investigator, Symptoms Study

About your symptoms

These questions are about symptoms you noticed before your diagnosis. We are interested in symptoms which you think are related to your cancer.

Please put a cross in the appropriate box, or write an answer as required.

Looking back...

1. Did you have any symptoms that you feel were due to your cancer before you were diagnosed?

Yes ☐ **Please continue with Question 2**

No ☐ **Please go to Section 2 on Page 5**

2. What was the first symptom that made you think something might be wrong?

[ ]

Please go to Question 3

The following questions are about when you first noticed a symptom and when you first told your GP or nurse about it.

Please give an exact date if you can. Otherwise give your best estimate. (For example approximately how long ago, the month or season). You wish to refer to your diary or calendar if you have it with you.

Here is an example question:

Decrease in appetite

Did you have this symptom?

Yes ☒ **Please complete A then B or C below**

No ☐ **Please go to Section 2**

A When did you first notice this? d d / m m / y y **OR** Estimate '3 months ago' or 'June'

B When did you first tell your GP or nurse? 2 3 / 1 2 / 0 7 **OR** Estimate

OR

C Put a cross here if you didn't tell your GP or nurse ☐

3. Change in bowel habit

Did you have this symptom?

Yes ☐ **Please complete A then B or C below**

No ☐ **Please go to Question 4**

A When did you first notice this? ☐☐ / ☐☐ / ☐☐ **OR** ☐

B When did you first tell your GP or nurse? ☐☐ / ☐☐ / ☐☐ **OR** ☐

OR

C Put a cross here if you didn't tell your GP or nurse ☐

4. Bleeding from back passage

Did you have this symptom?

Yes ☐ **Please complete A then B or C below**

No ☐ **Please go to Section 2**

A When did you first notice this? ☐☐ / ☐☐ / ☐☐ **OR** ☐

B When did you first tell your GP or nurse? ☐☐ / ☐☐ / ☐☐ **OR** ☐

OR

C Put a cross here if you didn't tell your GP or nurse ☐

Section 2

Please give an exact date if you can. Otherwise give your best estimate (for example approximately how long ago, the month or the season). You may wish to refer to your diary or calendar if you have it with you.

Did you have any of the following?

1. Decrease in appetite

Did you have this?

Yes ☐ **Please complete A then B or C below**

No ☐ **Please go to Question 2**

A When did you first notice this? ☐☐ / ☐☐ / ☐☐ **OR** ☐

B When did you first tell your GP or nurse? ☐☐ / ☐☐ / ☐☐ **OR** ☐

OR

C Put a cross here if you didn't tell your GP or nurse ☐

2. Unexplained weight loss

Did you have this?

Yes ☐ **Please complete A then B or C below**

No ☐ **Please go to Question 3**

A When did you first notice this? ☐☐ / ☐☐ / ☐☐ **OR** ☐

B When did you first tell your GP or nurse? ☐☐ / ☐☐ / ☐☐ **OR** ☐

OR

C Put a cross here if you didn't tell your GP or nurse ☐

3. **Fatigue or tiredness that is unusual for you**

Did you have this?

Yes ☐ **Please complete A then B or C below**

No ☐ **Please go to Question 4**

A When did you first notice this? ☐☐ / ☐☐ / ☐☐ **OR** ☐

B When did you first tell your GP or nurse? ☐☐ / ☐☐ / ☐☐ **OR** ☐

OR

C Put a cross here if you didn't tell your GP or nurse ☐

4. **Feeling different 'in yourself' from usual**

Did you have this?

Yes ☐ **Please complete A then B or C below**

No ☐ **Please go to Question 5**

A When did you first notice this? ☐☐ / ☐☐ / ☐☐ **OR** ☐

B When did you first tell your GP or nurse? ☐☐ / ☐☐ / ☐☐ **OR** ☐

OR

C Put a cross here if you didn't tell your GP or nurse ☐

Please try and describe what the feeling was

If you had any further symptoms which you feel are relevant to your cancer diagnosis, please list them below. If not, please go to Question 7.

Please describe the symptom here and complete A then B or C below

5.

A	When did you <u>first notice</u> this?	☐☐ / ☐☐ / ☐☐ **OR** ☐
B	When did you <u>first tell</u> your GP or nurse?	☐☐ / ☐☐ / ☐☐ **OR** ☐
	**OR**	
C	Put a cross here if you <u>didn't tell</u> your GP or nurse	☐

If you had another symptom you feel is relevant, please write it below, otherwise go to Question 7.

Please describe the symptom here and complete A then B or C below

6.

A	When did you <u>first notice</u> this?	☐☐ / ☐☐ / ☐☐ **OR** ☐
B	When did you <u>first tell</u> your GP or nurse?	☐☐ / ☐☐ / ☐☐ **OR** ☐
	**OR**	
C	Put a cross here if you <u>didn't tell</u> your GP or nurse	☐

If you had further symptoms you would like to tell us about, there is space at the end of the Questionnaire for you to write them.

Did your GP send you for any tests?

Thinking back to the time immediately before you diagnosis, did your GP send you for any of the following tests?

Please cross the appropriate boxes

7. Were you sent for any of these?

	Yes	Not sure	No
Blood test(s)	☐	☐	☐
CT Scan	☐	☐	☐
Ultrasound scan	☐	☐	☐
Barium Enema	☐	☐	☐
Sigmoidoscopy or colonoscopy (Looking at bowel with internal camera)	☐	☐	☐
X-ray	☐	☐	☐

Please go to Question 8

About you

The next questions relate to general information about you. It would help us to know more about the background of the people completing the questionnaire.

8. **Which best describes your employment status?**

Please cross one box only

- Employed full-time ☐
- Employed part-time ☐
- Self employed full-time ☐
- Self employed part-time ☐
- Unemployed (seeking work) ☐
- Unemployed (not seeking work) ☐
- Retired ☐
- Student ☐
- Permanently sick/disabled ☐
- Temporarily sick/disabled ☐
- Looking after family/home ☐

Other, please describe

9. **What is your highest level of qualification?**

Please cross one box only

- Degree (or equivalent) ☐
- Diploma (or equivalent) ☐
- 'A' level ☐
- GCSE / 'O' level ☐
- None ☐

Other, please specify

10. **How would you describe your ethnicity**

Please cross one box only

White ☐
or
- White British ☐
- White Irish ☐
- Other White background ☐

Mixed ☐
or
- White & Black Carribean ☐
- White & Black African ☐
- White & Asian ☐
- Other mixed background ☐

Black or Black British ☐
or
- Carribean ☐
- African ☐
- Other Black background ☐

Asian or Asian British ☐
or
- Indian ☐
- Pakistani ☐
- Bangladeshi ☐
- Other Asian background ☐

Chinese or other ethnic group ☐
or
- Chinese ☐
- Other ethnic group ☐

11.

Do you live alone?

Yes ☐ **Please go to Question 12**

No ☐ **Please say who you live with below**

Who do you live with?
(e.g. wife, husband, partner, family member)

12. Are you suffering from, or have you suffered from, any of the following in the past 2 years?

Please cross any that are applicable to you

- Asthma ☐
- Chronic Obstructive Pulmonary Disease (COPD) ☐
- Other lung disease (e.g. fibrosis, bronchiectatis etc) ☐
- Heart disease ☐
- Anxiety or depression ☐
- Inflammatory bowel disease ☐
- Irritable bowel syndrome ☐
- Peptic ulcer ☐
- Previous cancer ☐
- Diabetes ☐
- Arthritis ☐

13. About smoking

Please cross the appropriate statement

- Are you a current smoker? ☐
- Are you an ex-smoker? ☐
- Are you a non-smoker (never smoked)? ☐

14. Do you think you were more at risk of getting cancer because of your family history?

Yes ☐

No ☐

15. The following questions are about how completing the questionnaire made you feel.

A number of statements which people have used to describe themselves are given below. Read each statement and then cross the most appropriate box to indicate how you feel right now, at the moment. There are no right or wrong answers. Do not spend too much time on any one statement but give the answer which seems to describe your present feeling best.

		Not at all	Somewhat	Moderately	Very much
1.	I feel calm	☐	☐	☐	☐
2.	I am tense	☐	☐	☐	☐
3.	I feel upset	☐	☐	☐	☐
4.	I am relaxed	☐	☐	☐	☐
5.	I feel content	☐	☐	☐	☐
6.	I am worried	☐	☐	☐	☐

Please make sure you have answered all the questions.

If you have any other comments or there is anything else you would like to tell us about when you first noticed your symptoms, please do so using the space below.

If you feel you would like to talk to someone about this questionnaire at a later stage, please speak to the doctor or nurse who is looking after you, or be in touch with the research team, whose contact details are on your patient information sheet

Thank you for completing the questionnaire

Please give it to the research nurse

The next page is for the Researcher to complete

Researcher - Post-completion Sheet

Researcher initials: ☐☐☐

Date questionnaire completed: Date ☐☐ / ☐☐ / ☐☐ Time ☐☐:☐☐

If not completed, give reason ☐

How long is it since the patient was told of his/her diagnosis? ☐

How long did it take the patient to complete the questionnaire?

- Less than 5 minutes ☐
- Between 5 and 10 minutes ☐
- Between 11 and 15 minutes ☐
- Longer than 15 minutes ☐

For the patient selected to complete the questionnaire on their own

Did the patient ask for any help? ☐ Yes ☐ No

If yes,

please specify what help was requested

___

___

please specify what help was given

___

___

For the patient selected for the researcher-administered questionnaire

Did the patient find any of the questions difficult? ☐ Yes ☐ No

If yes,

please specify which questions and what the difficulty was

___

___

___

___

Was the patient made anxious? ☐ Yes ☐ No

If yes,

please give details

___

___

___

___

Where did the patient complete the questionnaire?

(Please specify whether this was in a separate room alone, in a corner of the clinic, the ward or other

___

Thank you. Please now send the questionnaire and patient information sheet and consent form back to Cardiff University in the freepost envelope provided.

57727

PID - | | 3 | 9 |

Ymddiriedolaeth GIG Gogledd Orllewin Cymru
North West Wales NHS Trust

NHS WALES GIG CYMRU
Ymddiriedolaeth GIG Gogledd Cymru
North Wales NHS Trust

CID - ☐ GLAN CLWYD
☐ GWYNEDD
☐ MAELOR

Confidential Questionnaire

Looking At Your Symptoms Study

This Study has been funded by Cancer Research UK

CARDIFF UNIVERSITY PRIFYSGOL CAERDYDD
Ysgol Glinigol Gogledd Cymru North Wales Clinical School
CANCER RESEARCH UK
sewtu South East Wales Trials Unit Uned Ymchwil De-ddwyrain Cymru

Dear Patient

I am very grateful to you for agreeing to help us with this study.

I hope the information you supply will help us give people a diagnosis earlier.

If you feel you would like to talk to someone about this questionnaire at a later stage, please speak to the doctor or nurse who is looking after you, or be in touch with the research team, whose contact details are on your patient information sheet.

I can assure you that anything you tell us will be treated in the utmost confidence.

Yours sincerely

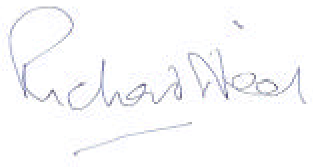

Dr Richard Neal
Senior Clinical Lecturer
Cardiff University
Chief Investigator, Symptoms Study

About your symptoms

These questions are about symptoms you noticed before your diagnosis. We are interested in symptoms which you think are related to your cancer.

Please put a cross in the appropriate box, or write an answer as required.

Looking back...

1. Did you have any symptoms that you feel were due to your cancer before you were diagnosed?

Yes ☐ **Please continue with Question 2**

No ☐ **Please go to Section 2 on Page 5**

2. What was the first symptom that made you think something might be wrong?

☐

Please go to Question 3

The following questions are about when you first noticed a symptom and when you first told your GP or nurse about it.

Please give an exact date if you can. Otherwise give your best estimate. (For example approximately how long ago, the month or season). You wish to refer to your diary or calendar if you have it with you.

Here is an example question:

Decrease in appetite

Did you have this symptom?

Yes ☒ **Please complete A then B or C below**

No ☐ **Please go to Section 2**

A When did you first notice this? d d / m m / y y **OR** Estimate '3 months ago' or 'June'

B When did you first tell your GP or nurse? 2 3 / 1 2 / 0 7 **OR** Estimate

OR

C Put a cross here if you didn't tell your GP or nurse ☐

3. Change in bowel habit

Did you have this symptom?

Yes ☐ **Please complete A then B or C below**

No ☐ **Please go to Question 4**

A When did you first notice this? ☐☐ / ☐☐ / ☐☐ **OR** ☐

B When did you first tell your GP or nurse? ☐☐ / ☐☐ / ☐☐ **OR** ☐

OR

C Put a cross here if you didn't tell your GP or nurse ☐

4. Bleeding from back passage

Did you have this symptom?

Yes ☐ **Please complete A then B or C below**

No ☐ **Please go to Section 2**

A When did you first notice this? ☐☐ / ☐☐ / ☐☐ **OR** ☐

B When did you first tell your GP or nurse? ☐☐ / ☐☐ / ☐☐ **OR** ☐

OR

C Put a cross here if you didn't tell your GP or nurse ☐

Section 2

Please give an exact date if you can. Otherwise give your best estimate (for example approximately how long ago, the month or the season). You may wish to refer to your diary or calendar if you have it with you.

Did you have any of the following?

1. Decrease in appetite

Did you have this?

Yes ☐ **Please complete A then B or C below**

No ☐ **Please go to Question 2**

A When did you first notice this? ☐☐ / ☐☐ / ☐☐ **OR** ☐

B When did you first tell your GP or nurse? ☐☐ / ☐☐ / ☐☐ **OR** ☐

OR

C Put a cross here if you didn't tell your GP or nurse ☐

2. Unexplained weight loss

Did you have this?

Yes ☐ **Please complete A then B or C below**

No ☐ **Please go to Question 3**

A When did you first notice this? ☐☐ / ☐☐ / ☐☐ **OR** ☐

B When did you first tell your GP or nurse? ☐☐ / ☐☐ / ☐☐ **OR** ☐

OR

C Put a cross here if you didn't tell your GP or nurse ☐

3. Fatigue or tiredness that is unusual for you

Did you have this?

Yes ☐ **Please complete A then B or C below**

No ☐ **Please go to Question 4**

A When did you first notice this? ☐☐ / ☐☐ / ☐☐ **OR** ☐

B When did you first tell your GP or nurse? ☐☐ / ☐☐ / ☐☐ **OR** ☐

OR

C Put a cross here if you didn't tell your GP or nurse ☐

4. Feeling different 'in yourself' from usual

Did you have this?

Yes ☐ **Please complete A then B or C below**

No ☐ **Please go to Question 5**

A When did you first notice this? ☐☐ / ☐☐ / ☐☐ **OR** ☐

B When did you first tell your GP or nurse? ☐☐ / ☐☐ / ☐☐ **OR** ☐

OR

C Put a cross here if you didn't tell your GP or nurse ☐

Please try and describe what the feeling was

If you had any further symptoms which you feel are relevant to your cancer diagnosis, please list them below. If not, please go to Question 7.

Please describe the symptom here and complete A then B or C below

5.

A When did you first notice this? ☐☐ / ☐☐ / ☐☐ OR ☐

B When did you first tell your GP or nurse? ☐☐ / ☐☐ / ☐☐ OR ☐

OR

C Put a cross here if you didn't tell your GP or nurse ☐

If you had another symptom you feel is relevant, please write it below, otherwise go to Question 7.

Please describe the symptom here and complete A then B or C below

6.

A When did you first notice this? ☐☐ / ☐☐ / ☐☐ OR ☐

B When did you first tell your GP or nurse? ☐☐ / ☐☐ / ☐☐ OR ☐

OR

C Put a cross here if you didn't tell your GP or nurse ☐

If you had further symptoms you would like to tell us about, there is space at the end of the Questionnaire for you to write them.

Did your GP send you for any tests?

Thinking back to the time immediately before you diagnosis, did your GP send you for any of the following tests?

Please cross the appropriate boxes

7. Were you sent for any of these?

	Yes	Not sure	No
Blood test(s)	☐	☐	☐
CT Scan	☐	☐	☐
Ultrasound scan	☐	☐	☐
Barium Enema	☐	☐	☐
Sigmoidoscopy or colonoscopy (Looking at bowel with internal camera)	☐	☐	☐
X-ray	☐	☐	☐

Please go to Question 8

About you

The next questions relate to general information about you. It would help us to know more about the background of the people completing the questionnaire.

8. **Which best describes your employment status?**

Please cross one box only

- Employed full-time ☐
- Employed part-time ☐
- Self employed full-time ☐
- Self employed part-time ☐
- Unemployed (seeking work) ☐
- Unemployed (not seeking work) ☐
- Retired ☐
- Student ☐
- Permanently sick/disabled ☐
- Temporarily sick/disabled ☐
- Looking after family/home ☐
- Other, please describe ____________

9. **What is your highest level of qualification?**

Please cross one box only

- Degree (or equivalent) ☐
- Diploma (or equivalent) ☐
- 'A' level ☐
- GCSE / 'O' level ☐
- None ☐
- Other, please specify ____________

10. **How would you describe your ethnicity**

Please cross one box only

White ☐
or
- White British ☐
- White Irish ☐
- Other White background ☐

Mixed ☐
or
- White & Black Carribean ☐
- White & Black African ☐
- White & Asian ☐
- Other mixed background ☐

Black or Black British ☐
or
- Carribean ☐
- African ☐
- Other Black background ☐

Asian or Asian British ☐
or
- Indian ☐
- Pakistani ☐
- Bangladeshi ☐
- Other Asian background ☐

Chinese or other ethnic group ☐
or
- Chinese ☐
- Other ethnic group ☐

11.

Do you live alone?

Yes ☐ **Please go to Question 12**

No ☐ **Please say who you live with below**

Who do you live with?
(e.g. wife, husband, partner, family member) ☐

12. **Are you suffering from, or have you suffered from, any of the following in the past 2 years?**

Please cross any that are applicable to you

- Asthma ☐
- Chronic Obstructive Pulmonary Disease (COPD) ☐
- Other lung disease (e.g. fibrosis, bronchiectatis etc) ☐
- Heart disease ☐
- Anxiety or depression ☐
- Inflammatory bowel disease ☐
- Irritable bowel syndrome ☐
- Peptic ulcer ☐
- Previous cancer ☐
- Diabetes ☐
- Arthritis ☐

13. **About smoking**

Please cross the appropriate statement

- Are you a current smoker? ☐
- Are you an ex-smoker? ☐
- Are you a non-smoker (never smoked)? ☐

14. **Do you think you were more at risk of getting cancer because of your family history?**

Yes ☐

No ☐

15. **The following questions are about how completing the questionnaire made you feel.**

A number of statements which people have used to describe themselves are given below. Read each statement and then cross the most appropriate box to indicate how you feel right now, at the moment. There are no right or wrong answers. Do not spend too much time on any one statement but give the answer which seems to describe your present feeling best.

		Not at all	Somewhat	Moderately	Very much
1.	I feel calm	☐	☐	☐	☐
2.	I am tense	☐	☐	☐	☐
3.	I feel upset	☐	☐	☐	☐
4.	I am relaxed	☐	☐	☐	☐
5.	I feel content	☐	☐	☐	☐
6.	I am worried	☐	☐	☐	☐

Please make sure you have answered all the questions.

If you have any other comments or there is anything else you would like to tell us about when you first noticed your symptoms, please do so using the space below.

If you feel you would like to talk to someone about this questionnaire at a later stage, please speak to the doctor or nurse who is looking after you, or be in touch with the research team, whose contact details are on your patient information sheet

Thank you for completing the questionnaire

Please give it to the research nurse

The next page is for the Researcher to complete

Researcher - Post-completion Sheet

Researcher initials: ☐☐☐

Date questionnaire completed: Date ☐☐ / ☐☐ / ☐☐ Time ☐☐:☐☐

If not completed, give reason ☐

How long is it since the patient was told of his/her diagnosis? ☐

How long did it take the patient to complete the questionnaire?

- Less than 5 minutes ☐
- Between 5 and 10 minutes ☐
- Between 11 and 15 minutes ☐
- Longer than 15 minutes ☐

For the patient selected to complete the questionnaire on their own

Did the patient ask for any help? ☐ Yes ☐ No

If yes,

please specify what help was requested

____

____

please specify what help was given

____

____

For the patient selected for the researcher-administered questionnaire

Did the patient find any of the questions difficult? ☐ Yes ☐ No

If yes,

please specify which questions and what the difficulty was

____

____

____

____

Was the patient made anxious? ☐ Yes ☐ No

If yes,

please give details

____

____

____

____

Where did the patient complete the questionnaire?
(Please specify whether this was in a separate room alone, in a corner of the clinic, the ward or other

____

Thank you. Please now send the questionnaire and patient information sheet and consent form back to Cardiff University in the freepost envelope provided.

57727

PID - 4 0

Ymddiriedolaeth GIG Gogledd Orllewin Cymru
North West Wales NHS Trust

NHS WALES GIG CYMRU
Ymddiriedolaeth GIG Gogledd Cymru
North Wales NHS Trust

CID - ☐ GLAN CLWYD
☐ GWYNEDD
☐ MAELOR

Confidential Questionnaire

Looking At Your Symptoms Study

This Study has been funded by Cancer Research UK

Cardiff University / Prifysgol Caerdydd
Ysgol Glinigol Gogledd Cymru / North Wales Clinical School
Cancer Research UK
sewtu South East Wales Trials Unit / Uned Ymchwil De-ddwyrain Cymru

Dear Patient

I am very grateful to you for agreeing to help us with this study.

I hope the information you supply will help us give people a diagnosis earlier.

If you feel you would like to talk to someone about this questionnaire at a later stage, please speak to the doctor or nurse who is looking after you, or be in touch with the research team, whose contact details are on your patient information sheet.

I can assure you that anything you tell us will be treated in the utmost confidence.

Yours sincerely

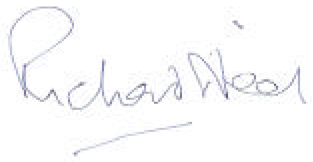

Dr Richard Neal
Senior Clinical Lecturer
Cardiff University
Chief Investigator, Symptoms Study

About your symptoms

These questions are about symptoms you noticed before your diagnosis. We are interested in symptoms which you think are related to your cancer.

Please put a cross in the appropriate box, or write an answer as required.

Looking back...

1. Did you have any symptoms that you feel were due to your cancer before you were diagnosed?

Yes ☐ **Please continue with Question 2**

No ☐ **Please go to Section 2 on Page 5**

2. What was the first symptom that made you think something might be wrong?

☐

Please go to Question 3

The following questions are about when you first noticed a symptom and when you first told your GP or nurse about it.

Please give an exact date if you can. Otherwise give your best estimate. (For example approximately how long ago, the month or season). You wish to refer to your diary or calendar if you have it with you.

Here is an example question:

Decrease in appetite

Did you have this symptom?

Yes ☒ **Please complete A then B or C below**

No ☐ **Please go to Section 2**

A When did you first notice this? d d / m m / y y **OR** Estimate '3 months ago' or 'June'

B When did you first tell your GP or nurse? 2 3 / 1 2 / 0 7 **OR** Estimate

OR

C Put a cross here if you didn't tell your GP or nurse ☐

3. Change in bowel habit

Did you have this symptom?

Yes ☐ **Please complete A then B or C below**

No ☐ **Please go to Question 4**

A When did you first notice this?

☐☐ / ☐☐ / ☐☐ **OR** ☐

B When did you first tell your GP or nurse?

☐☐ / ☐☐ / ☐☐ **OR** ☐

OR

C Put a cross here if you didn't tell your GP or nurse ☐

4. Bleeding from back passage

Did you have this symptom?

Yes ☐ **Please complete A then B or C below**

No ☐ **Please go to Section 2**

A When did you first notice this?

☐☐ / ☐☐ / ☐☐ **OR** ☐

B When did you first tell your GP or nurse?

☐☐ / ☐☐ / ☐☐ **OR** ☐

OR

C Put a cross here if you didn't tell your GP or nurse ☐

Please give an exact date if you can. Otherwise give your best estimate (for example approximately how long ago, the month or the season). You may wish to refer to your diary or calendar if you have it with you.

Did you have any of the following?

1. Decrease in appetite

Did you have this?

Yes ☐ **Please complete A then B or C below**

No ☐ **Please go to Question 2**

A When did you first notice this? ☐☐ / ☐☐ / ☐☐ **OR** ☐

B When did you first tell your GP or nurse? ☐☐ / ☐☐ / ☐☐ **OR** ☐

OR

C Put a cross here if you didn't tell your GP or nurse ☐

2. Unexplained weight loss

Did you have this?

Yes ☐ **Please complete A then B or C below**

No ☐ **Please go to Question 3**

A When did you first notice this? ☐☐ / ☐☐ / ☐☐ **OR** ☐

B When did you first tell your GP or nurse? ☐☐ / ☐☐ / ☐☐ **OR** ☐

OR

C Put a cross here if you didn't tell your GP or nurse ☐

3. **Fatigue or tiredness that is unusual for you**

Did you have this?

Yes ☐ **Please complete A then B or C below**

No ☐ **Please go to Question 4**

A When did you first notice this? ☐☐ / ☐☐ / ☐☐ **OR** ☐

B When did you first tell your GP or nurse? ☐☐ / ☐☐ / ☐☐ **OR** ☐

OR

C Put a cross here if you didn't tell your GP or nurse ☐

4. **Feeling different 'in yourself' from usual**

Did you have this?

Yes ☐ **Please complete A then B or C below**

No ☐ **Please go to Question 5**

A When did you first notice this? ☐☐ / ☐☐ / ☐☐ **OR** ☐

B When did you first tell your GP or nurse? ☐☐ / ☐☐ / ☐☐ **OR** ☐

OR

C Put a cross here if you didn't tell your GP or nurse ☐

Please try and describe what the feeling was

If you had any further symptoms which you feel are relevant to your cancer diagnosis, please list them below. If not, please go to Question 7.

Please describe the symptom here and complete A then B or C below

5.

**A**	When did you first notice this?	☐☐ / ☐☐ / ☐☐ **OR** ☐
**B**	When did you first tell your GP or nurse?	☐☐ / ☐☐ / ☐☐ **OR** ☐
	**OR**	
**C**	Put a cross here if you didn't tell your GP or nurse	☐

If you had another symptom you feel is relevant, please write it below, otherwise go to Question 7.

Please describe the symptom here and complete A then B or C below

6.

**A**	When did you first notice this?	☐☐ / ☐☐ / ☐☐ **OR** ☐
**B**	When did you first tell your GP or nurse?	☐☐ / ☐☐ / ☐☐ **OR** ☐
	**OR**	
**C**	Put a cross here if you didn't tell your GP or nurse	☐

If you had further symptoms you would like to tell us about, there is space at the end of the Questionnaire for you to write them.

Did your GP send you for any tests?

Thinking back to the time immediately before you diagnosis, did your GP send you for any of the following tests?

Please cross the appropriate boxes

7. Were you sent for any of these?

	Yes	Not sure	No
Blood test(s)	☐	☐	☐
CT Scan	☐	☐	☐
Ultrasound scan	☐	☐	☐
Barium Enema	☐	☐	☐
Sigmoidoscopy or colonoscopy (Looking at bowel with internal camera)	☐	☐	☐
X-ray	☐	☐	☐

Please go to Question 8

About you

The next questions relate to general information about you. It would help us to know more about the background of the people completing the questionnaire.

8. Which best describes your employment status?

Please cross one box only

- Employed full-time ☐
- Employed part-time ☐
- Self employed full-time ☐
- Self employed part-time ☐
- Unemployed (seeking work) ☐
- Unemployed (not seeking work) ☐
- Retired ☐
- Student ☐
- Permanently sick/disabled ☐
- Temporarily sick/disabled ☐
- Looking after family/home ☐
- Other, please describe ______

9. What is your highest level of qualification?

Please cross one box only

- Degree (or equivalent) ☐
- Diploma (or equivalent) ☐
- 'A' level ☐
- GCSE / 'O' level ☐
- None ☐
- Other, please specify ______

10. **How would you describe your ethnicity**

Please cross one box only

White ☐
or
- White British ☐
- White Irish ☐
- Other White background ☐

Mixed ☐
or
- White & Black Carribean ☐
- White & Black African ☐
- White & Asian ☐
- Other mixed background ☐

Black or Black British ☐
or
- Carribean ☐
- African ☐
- Other Black background ☐

Asian or Asian British ☐
or
- Indian ☐
- Pakistani ☐
- Bangladeshi ☐
- Other Asian background ☐

Chinese or other ethnic group ☐
or
- Chinese ☐
- Other ethnic group ☐

11.

Do you live alone?

Yes ☐ **Please go to Question 12**

No ☐ **Please say who you live with below**

Who do you live with?
(e.g. wife, husband, partner, family member)

12. **Are you suffering from, or have you suffered from, any of the following in the past 2 years?**

Please cross any that are applicable to you

- Asthma ☐
- Chronic Obstructive Pulmonary Disease (COPD) ☐
- Other lung disease (e.g. fibrosis, bronchiectatis etc) ☐
- Heart disease ☐
- Anxiety or depression ☐
- Inflammatory bowel disease ☐
- Irritable bowel syndrome ☐
- Peptic ulcer ☐
- Previous cancer ☐
- Diabetes ☐
- Arthritis ☐

13. **About smoking**

Please cross the appropriate statement

- Are you a current smoker? ☐
- Are you an ex-smoker? ☐
- Are you a non-smoker (never smoked)? ☐

14. **Do you think you were more at risk of getting cancer because of your family history?**

Yes ☐

No ☐

15. **The following questions are about how completing the questionnaire made you feel.**

A number of statements which people have used to describe themselves are given below. Read each statement and then cross the most appropriate box to indicate how you feel right now, at the moment. There are no right or wrong answers. Do not spend too much time on any one statement but give the answer which seems to describe your present feeling best.

		Not at all	Somewhat	Moderately	Very much
1.	I feel calm	☐	☐	☐	☐
2.	I am tense	☐	☐	☐	☐
3.	I feel upset	☐	☐	☐	☐
4.	I am relaxed	☐	☐	☐	☐
5.	I feel content	☐	☐	☐	☐
6.	I am worried	☐	☐	☐	☐

Please make sure you have answered all the questions.

If you have any other comments or there is anything else you would like to tell us about when you first noticed your symptoms, please do so using the space below.

If you feel you would like to talk to someone about this questionnaire at a later stage, please speak to the doctor or nurse who is looking after you, or be in touch with the research team, whose contact details are on your patient information sheet

Thank you for completing the questionnaire

Please give it to the research nurse

The next page is for the Researcher to complete

57727

4 0

Researcher - Post-completion Sheet

Researcher initials: ☐☐☐

Date questionnaire completed: Date ☐☐ / ☐☐ / ☐☐ Time ☐☐:☐☐

If not completed, give reason ☐

How long is it since the patient was told of his/her diagnosis? ☐

How long did it take the patient to complete the questionnaire?

- Less than 5 minutes ☐
- Between 5 and 10 minutes ☐
- Between 11 and 15 minutes ☐
- Longer than 15 minutes ☐

For the patient selected to complete the questionnaire on their own

Did the patient ask for any help? ☐ Yes ☐ No

If yes,

please specify what help was requested

____

____

please specify what help was given

____

____

For the patient selected for the researcher-administered questionnaire

Did the patient find any of the questions difficult? ☐ Yes ☐ No

If yes,

please specify which questions and what the difficulty was

____

____

____

____

Was the patient made anxious? ☐ Yes ☐ No

If yes,

please give details

____

____

____

____

Where did the patient complete the questionnaire?
(Please specify whether this was in a separate room alone, in a corner of the clinic, the ward or other

____

Thank you. Please now send the questionnaire and patient information sheet and consent form back to Cardiff University in the freepost envelope provided.

57727

PID - 4 1

Ymddiriedolaeth GIG Gogledd Orllewin Cymru
North West Wales NHS Trust

NHS WALES GIG CYMRU
Ymddiriedolaeth GIG Gogledd Cymru
North Wales NHS Trust

CID - ☐ GLAN CLWYD
☐ GWYNEDD
☐ MAELOR

Confidential Questionnaire

Looking At Your Symptoms Study

This Study has been funded by Cancer Research UK

Cardiff University / Prifysgol Caerdydd
Ysgol Glinigol Gogledd Cymru / North Wales Clinical School
Cancer Research UK
sewtu — South East Wales Trials Unit / Uned Ymchwil De-ddwyrain Cymru

Dear Patient

I am very grateful to you for agreeing to help us with this study.

I hope the information you supply will help us give people a diagnosis earlier.

If you feel you would like to talk to someone about this questionnaire at a later stage, please speak to the doctor or nurse who is looking after you, or be in touch with the research team, whose contact details are on your patient information sheet.

I can assure you that anything you tell us will be treated in the utmost confidence.

Yours sincerely

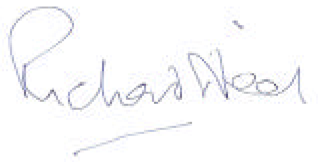

Dr Richard Neal
Senior Clinical Lecturer
Cardiff University
Chief Investigator, Symptoms Study

About your symptoms

These questions are about symptoms you noticed before your diagnosis. We are interested in symptoms which you think are related to your cancer.

Please put a cross in the appropriate box, or write an answer as required.

Looking back...

1. Did you have any symptoms that you feel were due to your cancer before you were diagnosed?

Yes ☐ **Please continue with Question 2**

No ☐ **Please go to Section 2 on Page 5**

2. What was the first symptom that made you think something might be wrong?

☐

Please go to Question 3

The following questions are about when you first noticed a symptom and when you first told your GP or nurse about it.

Please give an exact date if you can. Otherwise give your best estimate. (For example approximately how long ago, the month or season). You wish to refer to your diary or calendar if you have it with you.

Here is an example question:

Decrease in appetite

Did you have this symptom?

Yes ☒ **Please complete A then B or C below**

No ☐ **Please go to Section 2**

A When did you first notice this? d d / m m / y y **OR** Estimate '3 months ago' or 'June'

B When did you first tell your GP or nurse? 23 / 12 / 07 **OR** Estimate

OR

C Put a cross here if you didn't tell your GP or nurse ☐

3. Change in bowel habit

Did you have this symptom?

Yes ☐ **Please complete A then B or C below**

No ☐ **Please go to Question 4**

A When did you first notice this? ☐☐ / ☐☐ / ☐☐ **OR** ☐

B When did you first tell your GP or nurse? ☐☐ / ☐☐ / ☐☐ **OR** ☐

OR

C Put a cross here if you didn't tell your GP or nurse ☐

4. Bleeding from back passage

Did you have this symptom?

Yes ☐ **Please complete A then B or C below**

No ☐ **Please go to Section 2**

A When did you first notice this? ☐☐ / ☐☐ / ☐☐ **OR** ☐

B When did you first tell your GP or nurse? ☐☐ / ☐☐ / ☐☐ **OR** ☐

OR

C Put a cross here if you didn't tell your GP or nurse ☐

Section 2

Please give an exact date if you can. Otherwise give your best estimate (for example approximately how long ago, the month or the season). You may wish to refer to your diary or calendar if you have it with you.

Did you have any of the following?

1. Decrease in appetite

Did you have this?

Yes ☐ **Please complete A then B or C below**

No ☐ **Please go to Question 2**

A When did you first notice this? ☐☐ / ☐☐ / ☐☐ **OR** ☐

B When did you first tell your GP or nurse? ☐☐ / ☐☐ / ☐☐ **OR** ☐

OR

C Put a cross here if you didn't tell your GP or nurse ☐

2. Unexplained weight loss

Did you have this?

Yes ☐ **Please complete A then B or C below**

No ☐ **Please go to Question 3**

A When did you first notice this? ☐☐ / ☐☐ / ☐☐ **OR** ☐

B When did you first tell your GP or nurse? ☐☐ / ☐☐ / ☐☐ **OR** ☐

OR

C Put a cross here if you didn't tell your GP or nurse ☐

3. **Fatigue or tiredness that is unusual for you**

Did you have this?

Yes ☐ **Please complete A then B or C below**

No ☐ **Please go to Question 4**

A When did you first notice this? ☐☐ / ☐☐ / ☐☐ **OR** ☐

B When did you first tell your GP or nurse? ☐☐ / ☐☐ / ☐☐ **OR** ☐

OR

C Put a cross here if you didn't tell your GP or nurse ☐

4. **Feeling different 'in yourself' from usual**

Did you have this?

Yes ☐ **Please complete A then B or C below**

No ☐ **Please go to Question 5**

A When did you first notice this? ☐☐ / ☐☐ / ☐☐ **OR** ☐

B When did you first tell your GP or nurse? ☐☐ / ☐☐ / ☐☐ **OR** ☐

OR

C Put a cross here if you didn't tell your GP or nurse ☐

Please try and describe what the feeling was

If you had any further symptoms which you feel are relevant to your cancer diagnosis, please list them below. If not, please go to Question 7.

Please describe the symptom here and complete A then B or C below

5.

A	When did you first notice this?	__ __ / __ __ / __ __ OR
B	When did you first tell your GP or nurse?	__ __ / __ __ / __ __ OR
	**OR**	
C	Put a cross here if you didn't tell your GP or nurse	☐

If you had another symptom you feel is relevant, please write it below, otherwise go to Question 7.

Please describe the symptom here and complete A then B or C below

6.

A	When did you first notice this?	__ __ / __ __ / __ __ OR
B	When did you first tell your GP or nurse?	__ __ / __ __ / __ __ OR
	**OR**	
C	Put a cross here if you didn't tell your GP or nurse	☐

If you had further symptoms you would like to tell us about, there is space at the end of the Questionnaire for you to write them.

Did your GP send you for any tests?

Thinking back to the time immediately before you diagnosis, did your GP send you for any of the following tests?

Please cross the appropriate boxes

7. Were you sent for any of these?

	Yes	Not sure	No
Blood test(s)	☐	☐	☐
CT Scan	☐	☐	☐
Ultrasound scan	☐	☐	☐
Barium Enema	☐	☐	☐
Sigmoidoscopy or colonoscopy (Looking at bowel with internal camera)	☐	☐	☐
X-ray	☐	☐	☐

Please go to Question 8

About you

The next questions relate to general information about you. It would help us to know more about the background of the people completing the questionnaire.

8. **Which best describes your employment status?**

Please cross one box only

- Employed full-time ☐
- Employed part-time ☐
- Self employed full-time ☐
- Self employed part-time ☐
- Unemployed (seeking work) ☐
- Unemployed (not seeking work) ☐
- Retired ☐
- Student ☐
- Permanently sick/disabled ☐
- Temporarily sick/disabled ☐
- Looking after family/home ☐
- Other, please describe ______

9. **What is your highest level of qualification?**

Please cross one box only

- Degree (or equivalent) ☐
- Diploma (or equivalent) ☐
- 'A' level ☐
- GCSE / 'O' level ☐
- None ☐
- Other, please specify ______

10. How would you describe your ethnicity

Please cross one box only

White ☐
or
- White British ☐
- White Irish ☐
- Other White background ☐

Mixed ☐
or
- White & Black Carribean ☐
- White & Black African ☐
- White & Asian ☐
- Other mixed background ☐

Black or Black British ☐
or
- Carribean ☐
- African ☐
- Other Black background ☐

Asian or Asian British ☐
or
- Indian ☐
- Pakistani ☐
- Bangladeshi ☐
- Other Asian background ☐

Chinese or other ethnic group ☐
or
- Chinese ☐
- Other ethnic group ☐

11.

Do you live alone?

Yes ☐ **Please go to Question 12**

No ☐ **Please say who you live with below**

Who do you live with?
(e.g. wife, husband, partner, family member)

12. **Are you suffering from, or have you suffered from, any of the following in the past 2 years?**

Please cross any that are applicable to you

- Asthma ☐
- Chronic Obstructive Pulmonary Disease (COPD) ☐
- Other lung disease (e.g. fibrosis, bronchiectatis etc) ☐
- Heart disease ☐
- Anxiety or depression ☐
- Inflammatory bowel disease ☐
- Irritable bowel syndrome ☐
- Peptic ulcer ☐
- Previous cancer ☐
- Diabetes ☐
- Arthritis ☐

13. **About smoking**

Please cross the appropriate statement

- Are you a current smoker? ☐
- Are you an ex-smoker? ☐
- Are you a non-smoker (never smoked)? ☐

14. **Do you think you were more at risk of getting cancer because of your family history?**

Yes ☐
No ☐

15. **The following questions are about how completing the questionnaire made you feel.**

A number of statements which people have used to describe themselves are given below. Read each statement and then cross the most appropriate box to indicate how you feel <u>right now, at the moment.</u> There are no right or wrong answers. Do not spend too much time on any one statement but give the answer which seems to describe your present feeling best.

		Not at all	Somewhat	Moderately	Very much
1.	I feel calm	☐	☐	☐	☐
2.	I am tense	☐	☐	☐	☐
3.	I feel upset	☐	☐	☐	☐
4.	I am relaxed	☐	☐	☐	☐
5.	I feel content	☐	☐	☐	☐
6.	I am worried	☐	☐	☐	☐

Please make sure you have answered all the questions.

If you have any other comments or there is anything else you would like to tell us about when you first noticed your symptoms, please do so using the space below.

If you feel you would like to talk to someone about this questionnaire at a later stage, please speak to the doctor or nurse who is looking after you, or be in touch with the research team, whose contact details are on your patient information sheet

Thank you for completing the questionnaire

Please give it to the research nurse

The next page is for the Researcher to complete

Researcher - Post-completion Sheet **Researcher initials:** ☐☐☐

Date questionnaire completed: Date ☐☐ / ☐☐ / ☐☐ Time ☐☐:☐☐

If not completed, give reason ☐

How long is it since the patient was told of his/her diagnosis? ☐

How long did it take the patient to complete the questionnaire?

- Less than 5 minutes ☐
- Between 5 and 10 minutes ☐
- Between 11 and 15 minutes ☐
- Longer than 15 minutes ☐

For the patient selected to <u>complete the questionnaire on their own</u>

Did the patient ask for any help? ☐ Yes ☐ No

If yes,

please specify what help was requested

____

____

please specify what help was given

____

____

For the patient selected for <u>the researcher-administered questionnaire</u>

Did the patient find any of the questions difficult? ☐ Yes ☐ No

If yes,

please specify which questions and what the difficulty was

____

____

____

____

Was the patient made anxious? ☐ Yes ☐ No

If yes,

please give details

____

____

____

____

Where did the patient complete the questionnaire?
(Please specify whether this was in a separate room alone, in a corner of the clinic, the ward or other

____

Thank you. Please now send the questionnaire and patient information sheet and consent form back to Cardiff University in the freepost envelope provided.

57727

PID - | | 4 | 2 |

Ymddiriedolaeth GIG Gogledd Orllewin Cymru
North West Wales NHS Trust

NHS WALES GIG CYMRU
Ymddiriedolaeth GIG Gogledd Cymru
North Wales NHS Trust

CID -
- ☐ GLAN CLWYD
- ☐ GWYNEDD
- ☐ MAELOR

Confidential Questionnaire

Looking At Your Symptoms Study

This Study has been funded by Cancer Research UK

Cardiff University / Prifysgol Caerdydd
Ysgol Glinigol Gogledd Cymru / North Wales Clinical School
Cancer Research UK
sewtu South East Wales Trials Unit / Uned Ymchwil De-ddwyrain Cymru

Dear Patient

I am very grateful to you for agreeing to help us with this study.

I hope the information you supply will help us give people a diagnosis earlier.

If you feel you would like to talk to someone about this questionnaire at a later stage, please speak to the doctor or nurse who is looking after you, or be in touch with the research team, whose contact details are on your patient information sheet.

I can assure you that anything you tell us will be treated in the utmost confidence.

Yours sincerely

Dr Richard Neal
Senior Clinical Lecturer
Cardiff University
Chief Investigator, Symptoms Study

About your symptoms

These questions are about symptoms you noticed before your diagnosis. We are interested in symptoms which you think are related to your cancer.

Please put a cross in the appropriate box, or write an answer as required.

Looking back...

1. Did you have any symptoms that you feel were due to your cancer before you were diagnosed?

Yes ☐ **Please continue with Question 2**

No ☐ **Please go to Section 2 on Page 5**

2. What was the first symptom that made you think something might be wrong?

☐

Please go to Question 3

The following questions are about when you first noticed a symptom and when you first told your GP or nurse about it.

Please give an exact date if you can. Otherwise give your best estimate. (For example approximately how long ago, the month or season). You wish to refer to your diary or calendar if you have it with you.

Here is an example question:

Decrease in appetite

Did you have this symptom?

Yes ☒ **Please complete A then B or C below**

No ☐ **Please go to Section 2**

A When did you first notice this? d d / m m / y y **OR** Estimate '3 months ago' or 'June'

B When did you first tell your GP or nurse? 2 3 / 1 2 / 0 7 **OR** Estimate

OR

C Put a cross here if you didn't tell your GP or nurse ☐

3. Change in bowel habit

Did you have this symptom?

Yes ☐ **Please complete A then B or C below**
No ☐ **Please go to Question 4**

A When did you first notice this? ☐☐ / ☐☐ / ☐☐ **OR** ☐

B When did you first tell your GP or nurse? ☐☐ / ☐☐ / ☐☐ **OR** ☐

OR

C Put a cross here if you didn't tell your GP or nurse ☐

4. Bleeding from back passage

Did you have this symptom?

Yes ☐ **Please complete A then B or C below**
No ☐ **Please go to Section 2**

A When did you first notice this? ☐☐ / ☐☐ / ☐☐ **OR** ☐

B When did you first tell your GP or nurse? ☐☐ / ☐☐ / ☐☐ **OR** ☐

OR

C Put a cross here if you didn't tell your GP or nurse ☐

Please give an exact date if you can. Otherwise give your best estimate (for example approximately how long ago, the month or the season). You may wish to refer to your diary or calendar if you have it with you.

Did you have any of the following?

1. Decrease in appetite

Did you have this?

Yes ☐ **Please complete A then B or C below**

No ☐ **Please go to Question 2**

A When did you first notice this? ☐☐ / ☐☐ / ☐☐ **OR** ☐

B When did you first tell your GP or nurse? ☐☐ / ☐☐ / ☐☐ **OR** ☐

OR

C Put a cross here if you didn't tell your GP or nurse ☐

2. Unexplained weight loss

Did you have this?

Yes ☐ **Please complete A then B or C below**

No ☐ **Please go to Question 3**

A When did you first notice this? ☐☐ / ☐☐ / ☐☐ **OR** ☐

B When did you first tell your GP or nurse? ☐☐ / ☐☐ / ☐☐ **OR** ☐

OR

C Put a cross here if you didn't tell your GP or nurse ☐

3. Fatigue or tiredness that is unusual for you

Did you have this?

Yes ☐ **Please complete A then B or C below**

No ☐ **Please go to Question 4**

A When did you first notice this? ☐☐ / ☐☐ / ☐☐ **OR** ☐

B When did you first tell your GP or nurse? ☐☐ / ☐☐ / ☐☐ **OR** ☐

OR

C Put a cross here if you didn't tell your GP or nurse ☐

4. Feeling different 'in yourself' from usual

Did you have this?

Yes ☐ **Please complete A then B or C below**

No ☐ **Please go to Question 5**

A When did you first notice this? ☐☐ / ☐☐ / ☐☐ **OR** ☐

B When did you first tell your GP or nurse? ☐☐ / ☐☐ / ☐☐ **OR** ☐

OR

C Put a cross here if you didn't tell your GP or nurse ☐

Please try and describe what the feeling was

If you had any further symptoms which you feel are relevant to your cancer diagnosis, please list them below. If not, please go to Question 7.

Please describe the symptom here and complete A then B or C below

5.

**A**	When did you first notice this?	☐☐ / ☐☐ / ☐☐ **OR** ☐
**B**	When did you first tell your GP or nurse?	☐☐ / ☐☐ / ☐☐ **OR** ☐
	**OR**	
**C**	Put a cross here if you didn't tell your GP or nurse	☐

If you had another symptom you feel is relevant, please write it below, otherwise go to Question 7.

Please describe the symptom here and complete A then B or C below

6.

**A**	When did you first notice this?	☐☐ / ☐☐ / ☐☐ **OR** ☐
**B**	When did you first tell your GP or nurse?	☐☐ / ☐☐ / ☐☐ **OR** ☐
	**OR**	
**C**	Put a cross here if you didn't tell your GP or nurse	☐

If you had further symptoms you would like to tell us about, there is space at the end of the Questionnaire for you to write them.

Did your GP send you for any tests?

Thinking back to the time immediately before you diagnosis, did your GP send you for any of the following tests?

Please cross the appropriate boxes

7. Were you sent for any of these?

	Yes	Not sure	No
Blood test(s)	☐	☐	☐
CT Scan	☐	☐	☐
Ultrasound scan	☐	☐	☐
Barium Enema	☐	☐	☐
Sigmoidoscopy or colonoscopy (Looking at bowel with internal camera)	☐	☐	☐
X-ray	☐	☐	☐

Please go to Question 8

About you

The next questions relate to general information about you. It would help us to know more about the background of the people completing the questionnaire.

8. Which best describes your employment status?

Please cross one box only

- Employed full-time ☐
- Employed part-time ☐
- Self employed full-time ☐
- Self employed part-time ☐
- Unemployed (seeking work) ☐
- Unemployed (not seeking work) ☐
- Retired ☐
- Student ☐
- Permanently sick/disabled ☐
- Temporarily sick/disabled ☐
- Looking after family/home ☐
- Other, please describe ☐

9. What is your highest level of qualification?

Please cross one box only

- Degree (or equivalent) ☐
- Diploma (or equivalent) ☐
- 'A' level ☐
- GCSE / 'O' level ☐
- None ☐
- Other, please specify ☐

10. How would you describe your ethnicity

Please cross one box only

White ☐
or
- White British ☐
- White Irish ☐
- Other White background ☐

Mixed ☐
or
- White & Black Carribean ☐
- White & Black African ☐
- White & Asian ☐
- Other mixed background ☐

Black or Black British ☐
or
- Carribean ☐
- African ☐
- Other Black background ☐

Asian or Asian British ☐
or
- Indian ☐
- Pakistani ☐
- Bangladeshi ☐
- Other Asian background ☐

Chinese or other ethnic group ☐
or
- Chinese ☐
- Other ethnic group ☐

11.

Do you live alone?

Yes ☐ **Please go to Question 12**

No ☐ **Please say who you live with below**

Who do you live with?
(e.g. wife, husband, partner, family member)

12. **Are you suffering from, or have you suffered from, any of the following in the past 2 years?**

Please cross any that are applicable to you

- Asthma ☐
- Chronic Obstructive Pulmonary Disease (COPD) ☐
- Other lung disease (e.g. fibrosis, bronchiectatis etc) ☐
- Heart disease ☐
- Anxiety or depression ☐
- Inflammatory bowel disease ☐
- Irritable bowel syndrome ☐
- Peptic ulcer ☐
- Previous cancer ☐
- Diabetes ☐
- Arthritis ☐

13. **About smoking**

Please cross the appropriate statement

- Are you a current smoker? ☐
- Are you an ex-smoker? ☐
- Are you a non-smoker (never smoked)? ☐

14. **Do you think you were more at risk of getting cancer because of your family history?**

Yes ☐

No ☐

15. The following questions are about how completing the questionnaire made you feel.

A number of statements which people have used to describe themselves are given below. Read each statement and then cross the most appropriate box to indicate how you feel right now, at the moment. There are no right or wrong answers. Do not spend too much time on any one statement but give the answer which seems to describe your present feeling best.

		Not at all	Somewhat	Moderately	Very much
1.	I feel calm	☐	☐	☐	☐
2.	I am tense	☐	☐	☐	☐
3.	I feel upset	☐	☐	☐	☐
4.	I am relaxed	☐	☐	☐	☐
5.	I feel content	☐	☐	☐	☐
6.	I am worried	☐	☐	☐	☐

Please make sure you have answered all the questions.

If you have any other comments or there is anything else you would like to tell us about when you first noticed your symptoms, please do so using the space below.

If you feel you would like to talk to someone about this questionnaire at a later stage, please speak to the doctor or nurse who is looking after you, or be in touch with the research team, whose contact details are on your patient information sheet

Thank you for completing the questionnaire

Please give it to the research nurse

The next page is for the Researcher to complete

Researcher - Post-completion Sheet **Researcher initials:** ☐☐☐

Date questionnaire completed: Date ☐☐ / ☐☐ / ☐☐ Time ☐☐ : ☐☐

If not completed, give reason ☐

How long is it since the patient was told of his/her diagnosis? ☐

How long did it take the patient to complete the questionnaire?

- Less than 5 minutes ☐
- Between 5 and 10 minutes ☐
- Between 11 and 15 minutes ☐
- Longer than 15 minutes ☐

For the patient selected to complete the questionnaire on their own

Did the patient ask for any help? ☐ Yes ☐ No

If yes,

please specify what help was requested

____

____

please specify what help was given

____

____

For the patient selected for the researcher-administered questionnaire

Did the patient find any of the questions difficult? ☐ Yes ☐ No

If yes,

please specify which questions and what the difficulty was

____

____

____

____

Was the patient made anxious? ☐ Yes ☐ No

If yes,

please give details

____

____

____

____

Where did the patient complete the questionnaire?
(Please specify whether this was in a separate room alone, in a corner of the clinic, the ward or other

____

Thank you. Please now send the questionnaire and patient information sheet and consent form back to Cardiff University in the freepost envelope provided.

57727

PID - | | 4 | 3 |

Ymddiriedolaeth GIG Gogledd Orllewin Cymru
North West Wales NHS Trust

NHS WALES GIG CYMRU
Ymddiriedolaeth GIG Gogledd Cymru
North Wales NHS Trust

☐ GLAN CLWYD

CID - ☐ GWYNEDD

☐ MAELOR

Confidential Questionnaire

Looking At Your Symptoms Study

This Study has been funded by Cancer Research UK

Cardiff University / Prifysgol Caerdydd
Ysgol Glinigol Gogledd Cymru / North Wales Clinical School
Cancer Research UK
sewtu — South East Wales Trials Unit / Uned Ymchwil De-ddwyrain Cymru

Dear Patient

I am very grateful to you for agreeing to help us with this study.

I hope the information you supply will help us give people a diagnosis earlier.

If you feel you would like to talk to someone about this questionnaire at a later stage, please speak to the doctor or nurse who is looking after you, or be in touch with the research team, whose contact details are on your patient information sheet.

I can assure you that anything you tell us will be treated in the utmost confidence.

Yours sincerely

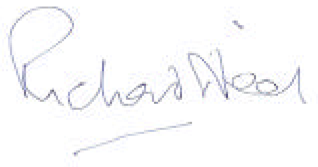

Dr Richard Neal
Senior Clinical Lecturer
Cardiff University
Chief Investigator, Symptoms Study

About your symptoms

These questions are about symptoms you noticed before your diagnosis. We are interested in symptoms which you think are related to your cancer.

Please put a cross in the appropriate box, or write an answer as required.

Looking back...

1. Did you have any symptoms that you feel were due to your cancer before you were diagnosed?

Yes ☐ **Please continue with Question 2**

No ☐ **Please go to Section 2 on Page 5**

2. What was the first symptom that made you think something might be wrong?

☐

Please go to Question 3

The following questions are about when you first noticed a symptom and when you first told your GP or nurse about it.

Please give an exact date if you can. Otherwise give your best estimate. (For example approximately how long ago, the month or season). You wish to refer to your diary or calendar if you have it with you.

Here is an example question:

Decrease in appetite

Did you have this symptom?

Yes ☒ **Please complete A then B or C below**

No ☐ **Please go to Section 2**

A When did you first notice this? d d / m m / y y **OR** Estimate '3 months ago' or 'June'

B When did you first tell your GP or nurse? 2 3 / 1 2 / 0 7 **OR** Estimate

OR

C Put a cross here if you didn't tell your GP or nurse ☐

3. Change in bowel habit

Did you have this symptom?

Yes ☐ **Please complete A then B or C below**

No ☐ **Please go to Question 4**

A When did you first notice this? ☐☐ / ☐☐ / ☐☐ **OR** ☐

B When did you first tell your GP or nurse? ☐☐ / ☐☐ / ☐☐ **OR** ☐

OR

C Put a cross here if you didn't tell your GP or nurse ☐

4. Bleeding from back passage

Did you have this symptom?

Yes ☐ **Please complete A then B or C below**

No ☐ **Please go to Section 2**

A When did you first notice this? ☐☐ / ☐☐ / ☐☐ **OR** ☐

B When did you first tell your GP or nurse? ☐☐ / ☐☐ / ☐☐ **OR** ☐

OR

C Put a cross here if you didn't tell your GP or nurse ☐

Section 2

Please give an exact date if you can. Otherwise give your best estimate (for example approximately how long ago, the month or the season). You may wish to refer to your diary or calendar if you have it with you.

Did you have any of the following?

1. Decrease in appetite

Did you have this?

Yes ☐ **Please complete A then B or C below**

No ☐ **Please go to Question 2**

A When did you first notice this? ☐☐ / ☐☐ / ☐☐ **OR** ☐

B When did you first tell your GP or nurse? ☐☐ / ☐☐ / ☐☐ **OR** ☐

OR

C Put a cross here if you didn't tell your GP or nurse ☐

2. Unexplained weight loss

Did you have this?

Yes ☐ **Please complete A then B or C below**

No ☐ **Please go to Question 3**

A When did you first notice this? ☐☐ / ☐☐ / ☐☐ **OR** ☐

B When did you first tell your GP or nurse? ☐☐ / ☐☐ / ☐☐ **OR** ☐

OR

C Put a cross here if you didn't tell your GP or nurse ☐

3. **Fatigue or tiredness that is unusual for you**

Did you have this?

Yes ☐ **Please complete A then B or C below**

No ☐ **Please go to Question 4**

A When did you first notice this? ☐☐ / ☐☐ / ☐☐ **OR** ☐

B When did you first tell your GP or nurse? ☐☐ / ☐☐ / ☐☐ **OR** ☐

OR

C Put a cross here if you didn't tell your GP or nurse ☐

4. **Feeling different 'in yourself' from usual**

Did you have this?

Yes ☐ **Please complete A then B or C below**

No ☐ **Please go to Question 5**

A When did you first notice this? ☐☐ / ☐☐ / ☐☐ **OR** ☐

B When did you first tell your GP or nurse? ☐☐ / ☐☐ / ☐☐ **OR** ☐

OR

C Put a cross here if you didn't tell your GP or nurse ☐

Please try and describe what the feeling was

If you had any further symptoms which you feel are relevant to your cancer diagnosis, please list them below. If not, please go to Question 7.

Please describe the symptom here and complete A then B or C below

5.

A When did you first notice this? __/__/__ OR

B When did you first tell your GP or nurse? __/__/__ OR

OR

C Put a cross here if you didn't tell your GP or nurse ☐

If you had another symptom you feel is relevant, please write it below, otherwise go to Question 7.

Please describe the symptom here and complete A then B or C below

6.

A When did you first notice this? __/__/__ OR

B When did you first tell your GP or nurse? __/__/__ OR

OR

C Put a cross here if you didn't tell your GP or nurse ☐

If you had further symptoms you would like to tell us about, there is space at the end of the Questionnaire for you to write them.

Did your GP send you for any tests?

Thinking back to the time immediately before you diagnosis, did your GP send you for any of the following tests?

Please cross the appropriate boxes

7. Were you sent for any of these?

	Yes	Not sure	No
Blood test(s)	☐	☐	☐
CT Scan	☐	☐	☐
Ultrasound scan	☐	☐	☐
Barium Enema	☐	☐	☐
Sigmoidoscopy or colonoscopy (Looking at bowel with internal camera)	☐	☐	☐
X-ray	☐	☐	☐

Please go to Question 8

About you

The next questions relate to general information about you. It would help us to know more about the background of the people completing the questionnaire.

8. Which best describes your employment status?

Please cross one box only

- Employed full-time ☐
- Employed part-time ☐
- Self employed full-time ☐
- Self employed part-time ☐
- Unemployed (seeking work) ☐
- Unemployed (not seeking work) ☐
- Retired ☐
- Student ☐
- Permanently sick/disabled ☐
- Temporarily sick/disabled ☐
- Looking after family/home ☐
- Other, please describe

9. What is your highest level of qualification?

Please cross one box only

- Degree (or equivalent) ☐
- Diploma (or equivalent) ☐
- 'A' level ☐
- GCSE / 'O' level ☐
- None ☐
- Other, please specify

10. **How would you describe your ethnicity**

Please cross one box only

White ☐
or
White British ☐
White Irish ☐
Other White background ☐

Mixed ☐
or
White & Black Carribean ☐
White & Black African ☐
White & Asian ☐
Other mixed background ☐

Black or Black British ☐
or
Carribean ☐
African ☐
Other Black background ☐

Asian or Asian British ☐
or
Indian ☐
Pakistani ☐
Bangladeshi ☐
Other Asian background ☐

Chinese or other ethnic group ☐
or
Chinese ☐
Other ethnic group ☐

11.

Do you live alone?

Yes ☐ **Please go to Question 12**

No ☐ **Please say who you live with below**

Who do you live with?
(e.g. wife, husband, partner, family member)

12. Are you suffering from, or have you suffered from, any of the following in the past 2 years?

Please cross any that are applicable to you

- Asthma ☐
- Chronic Obstructive Pulmonary Disease (COPD) ☐
- Other lung disease (e.g. fibrosis, bronchiectatis etc) ☐
- Heart disease ☐
- Anxiety or depression ☐
- Inflammatory bowel disease ☐
- Irritable bowel syndrome ☐
- Peptic ulcer ☐
- Previous cancer ☐
- Diabetes ☐
- Arthritis ☐

13. About smoking

Please cross the appropriate statement

- Are you a current smoker? ☐
- Are you an ex-smoker? ☐
- Are you a non-smoker (never smoked)? ☐

14. Do you think you were more at risk of getting cancer because of your family history?

Yes ☐

No ☐

15. The following questions are about how completing the questionnaire made you feel.

A number of statements which people have used to describe themselves are given below. Read each statement and then cross the most appropriate box to indicate how you feel right now, at the moment. There are no right or wrong answers. Do not spend too much time on any one statement but give the answer which seems to describe your present feeling best.

		Not at all	Somewhat	Moderately	Very much
1.	I feel calm	☐	☐	☐	☐
2.	I am tense	☐	☐	☐	☐
3.	I feel upset	☐	☐	☐	☐
4.	I am relaxed	☐	☐	☐	☐
5.	I feel content	☐	☐	☐	☐
6.	I am worried	☐	☐	☐	☐

Please make sure you have answered all the questions.

If you have any other comments or there is anything else you would like to tell us about when you first noticed your symptoms, please do so using the space below.

If you feel you would like to talk to someone about this questionnaire at a later stage, please speak to the doctor or nurse who is looking after you, or be in touch with the research team, whose contact details are on your patient information sheet

Thank you for completing the questionnaire

Please give it to the research nurse

The next page is for the Researcher to complete

57727

4 3

Researcher - Post-completion Sheet

Researcher initials: ☐☐☐

Date questionnaire completed: Date ☐☐ / ☐☐ / ☐☐ Time ☐☐:☐☐

If not completed, give reason ☐

How long is it since the patient was told of his/her diagnosis? ☐

How long did it take the patient to complete the questionnaire?

- Less than 5 minutes ☐
- Between 5 and 10 minutes ☐
- Between 11 and 15 minutes ☐
- Longer than 15 minutes ☐

For the patient selected to complete the questionnaire on their own

Did the patient ask for any help? ☐ Yes ☐ No

If yes,

please specify what help was requested

____

____

please specify what help was given

____

____

For the patient selected for the researcher-administered questionnaire

Did the patient find any of the questions difficult? ☐ Yes ☐ No

If yes,

please specify which questions and what the difficulty was

____

____

____

____

Was the patient made anxious? ☐ Yes ☐ No

If yes,

please give details

____

____

____

____

Where did the patient complete the questionnaire?

(Please specify whether this was in a separate room alone, in a corner of the clinic, the ward or other

____

Thank you. Please now send the questionnaire and patient information sheet and consent form back to Cardiff University in the freepost envelope provided.

57727

PID - | | 4 | 4 |

Ymddiriedolaeth GIG Gogledd Orllewin Cymru
North West Wales NHS Trust

NHS WALES GIG CYMRU
Ymddiriedolaeth GIG Gogledd Cymru
North Wales NHS Trust

CID - ☐ GLAN CLWYD
☐ GWYNEDD
☐ MAELOR

Confidential Questionnaire

Looking At Your Symptoms Study

This Study has been funded by Cancer Research UK

Cardiff University Prifysgol Caerdydd
Ysgol Glinigol Gogledd Cymru North Wales Clinical School
Cancer Research UK
sewtu South East Wales Trials Unit Uned Ymchwil De-ddwyrain Cymru

Dear Patient

I am very grateful to you for agreeing to help us with this study.

I hope the information you supply will help us give people a diagnosis earlier.

If you feel you would like to talk to someone about this questionnaire at a later stage, please speak to the doctor or nurse who is looking after you, or be in touch with the research team, whose contact details are on your patient information sheet.

I can assure you that anything you tell us will be treated in the utmost confidence.

Yours sincerely

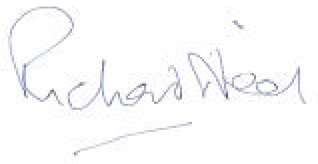

Dr Richard Neal
Senior Clinical Lecturer
Cardiff University
Chief Investigator, Symptoms Study

About your symptoms

These questions are about symptoms you noticed before your diagnosis. We are interested in symptoms which you think are related to your cancer.

Please put a cross in the appropriate box, or write an answer as required.

Looking back...

1. Did you have any symptoms that you feel were due to your cancer before you were diagnosed?

Yes ☐ **Please continue with Question 2**
No ☐ **Please go to Section 2 on Page 5**

2. What was the first symptom that made you think something might be wrong?

☐

Please go to Question 3

The following questions are about when you first noticed a symptom and when you first told your GP or nurse about it.

Please give an exact date if you can. Otherwise give your best estimate. (For example approximately how long ago, the month or season). You wish to refer to your diary or calendar if you have it with you.

Here is an example question:

Decrease in appetite

Did you have this symptom?

Yes ☒ **Please complete A then B or C below**
No ☐ **Please go to Section 2**

A When did you first notice this? d d / m m / y y **OR** Estimate: '3 months ago' or 'June'

B When did you first tell your GP or nurse? 2 3 / 1 2 / 0 7 **OR** Estimate

OR

C Put a cross here if you didn't tell your GP or nurse ☐

3. Change in bowel habit

Did you have this symptom?

Yes ☐ **Please complete A then B or C below**

No ☐ **Please go to Question 4**

A When did you first notice this? ☐☐ / ☐☐ / ☐☐ **OR** ☐

B When did you first tell your GP or nurse? ☐☐ / ☐☐ / ☐☐ **OR** ☐

OR

C Put a cross here if you didn't tell your GP or nurse ☐

4. Bleeding from back passage

Did you have this symptom?

Yes ☐ **Please complete A then B or C below**

No ☐ **Please go to Section 2**

A When did you first notice this? ☐☐ / ☐☐ / ☐☐ **OR** ☐

B When did you first tell your GP or nurse? ☐☐ / ☐☐ / ☐☐ **OR** ☐

OR

C Put a cross here if you didn't tell your GP or nurse ☐

Section 2

Please give an exact date if you can. Otherwise give your best estimate (for example approximately how long ago, the month or the season). You may wish to refer to your diary or calendar if you have it with you.

Did you have any of the following?

1. Decrease in appetite

Did you have this?

Yes ☐ **Please complete A then B or C below**

No ☐ **Please go to Question 2**

A When did you first notice this? ☐☐ / ☐☐ / ☐☐ **OR** ☐

B When did you first tell your GP or nurse? ☐☐ / ☐☐ / ☐☐ **OR** ☐

OR

C Put a cross here if you didn't tell your GP or nurse ☐

2. Unexplained weight loss

Did you have this?

Yes ☐ **Please complete A then B or C below**

No ☐ **Please go to Question 3**

A When did you first notice this? ☐☐ / ☐☐ / ☐☐ **OR** ☐

B When did you first tell your GP or nurse? ☐☐ / ☐☐ / ☐☐ **OR** ☐

OR

C Put a cross here if you didn't tell your GP or nurse ☐

3. **Fatigue or tiredness that is unusual for you**

Did you have this?

Yes ☐ **Please complete A then B or C below**

No ☐ **Please go to Question 4**

A When did you first notice this? ☐☐ / ☐☐ / ☐☐ **OR** ☐

B When did you first tell your GP or nurse? ☐☐ / ☐☐ / ☐☐ **OR** ☐

OR

C Put a cross here if you didn't tell your GP or nurse ☐

4. **Feeling different 'in yourself' from usual**

Did you have this?

Yes ☐ **Please complete A then B or C below**

No ☐ **Please go to Question 5**

A When did you first notice this? ☐☐ / ☐☐ / ☐☐ **OR** ☐

B When did you first tell your GP or nurse? ☐☐ / ☐☐ / ☐☐ **OR** ☐

OR

C Put a cross here if you didn't tell your GP or nurse ☐

Please try and describe what the feeling was

If you had any further symptoms which you feel are relevant to your cancer diagnosis, please list them below. If not, please go to Question 7.

Please describe the symptom here and complete A then B or C below

5.

A When did you <u>first notice</u> this? __ __ / __ __ / __ __ **OR**

B When did you <u>first tell</u> your GP or nurse? __ __ / __ __ / __ __ **OR**

OR

C Put a cross here if you <u>didn't tell</u> your GP or nurse ☐

If you had another symptom you feel is relevant, please write it below, otherwise go to Question 7.

Please describe the symptom here and complete A then B or C below

6.

A When did you <u>first notice</u> this? __ __ / __ __ / __ __ **OR**

B When did you <u>first tell</u> your GP or nurse? __ __ / __ __ / __ __ **OR**

OR

C Put a cross here if you <u>didn't tell</u> your GP or nurse ☐

If you had further symptoms you would like to tell us about, there is space at the end of the Questionnaire for you to write them.

Did your GP send you for any tests?

Thinking back to the time immediately before you diagnosis, did your GP send you for any of the following tests?

Please cross the appropriate boxes

7. Were you sent for any of these?

	Yes	Not sure	No
Blood test(s)	☐	☐	☐
CT Scan	☐	☐	☐
Ultrasound scan	☐	☐	☐
Barium Enema	☐	☐	☐
Sigmoidoscopy or colonoscopy (Looking at bowel with internal camera)	☐	☐	☐
X-ray	☐	☐	☐

Please go to Question 8

About you

The next questions relate to general information about you. It would help us to know more about the background of the people completing the questionnaire.

8. Which best describes your employment status?

Please cross one box only

- Employed full-time ☐
- Employed part-time ☐
- Self employed full-time ☐
- Self employed part-time ☐
- Unemployed (seeking work) ☐
- Unemployed (not seeking work) ☐
- Retired ☐
- Student ☐
- Permanently sick/disabled ☐
- Temporarily sick/disabled ☐
- Looking after family/home ☐
- Other, please describe ________

9. What is your highest level of qualification?

Please cross one box only

- Degree (or equivalent) ☐
- Diploma (or equivalent) ☐
- 'A' level ☐
- GCSE / 'O' level ☐
- None ☐
- Other, please specify ________

10. How would you describe your ethnicity

Please cross one box only

White ☐
or
- White British ☐
- White Irish ☐
- Other White background ☐

Mixed ☐
or
- White & Black Carribean ☐
- White & Black African ☐
- White & Asian ☐
- Other mixed background ☐

Black or Black British ☐
or
- Carribean ☐
- African ☐
- Other Black background ☐

Asian or Asian British ☐
or
- Indian ☐
- Pakistani ☐
- Bangladeshi ☐
- Other Asian background ☐

Chinese or other ethnic group ☐
or
- Chinese ☐
- Other ethnic group ☐

11.

Do you live alone?

Yes ☐ **Please go to Question 12**

No ☐ **Please say who you live with below**

Who do you live with?
(e.g. wife, husband, partner, family member)

☐

12. Are you suffering from, or have you suffered from, any of the following in the past 2 years?

Please cross any that are applicable to you

Asthma	☐
Chronic Obstructive Pulmonary Disease (COPD)	☐
Other lung disease (e.g. fibrosis, bronchiectatis etc)	☐
Heart disease	☐
Anxiety or depression	☐
Inflammatory bowel disease	☐
Irritable bowel syndrome	☐
Peptic ulcer	☐
Previous cancer	☐
Diabetes	☐
Arthritis	☐

13. About smoking

Please cross the appropriate statement

Are you a current smoker?	☐
Are you an ex-smoker?	☐
Are you a non-smoker (never smoked)?	☐

14. Do you think you were more at risk of getting cancer because of your family history?

Yes ☐

No ☐

15. **The following questions are about how completing the questionnaire made you feel.**

A number of statements which people have used to describe themselves are given below. Read each statement and then cross the most appropriate box to indicate how you feel right now, at the moment. There are no right or wrong answers. Do not spend too much time on any one statement but give the answer which seems to describe your present feeling best.

		Not at all	Somewhat	Moderately	Very much
1.	I feel calm	☐	☐	☐	☐
2.	I am tense	☐	☐	☐	☐
3.	I feel upset	☐	☐	☐	☐
4.	I am relaxed	☐	☐	☐	☐
5.	I feel content	☐	☐	☐	☐
6.	I am worried	☐	☐	☐	☐

Please make sure you have answered all the questions.

If you have any other comments or there is anything else you would like to tell us about when you first noticed your symptoms, please do so using the space below.

If you feel you would like to talk to someone about this questionnaire at a later stage, please speak to the doctor or nurse who is looking after you, or be in touch with the research team, whose contact details are on your patient information sheet

Thank you for completing the questionnaire

Please give it to the research nurse

The next page is for the Researcher to complete

57727

44

Researcher - Post-completion Sheet

Researcher initials: ☐☐☐

Date questionnaire completed: Date ☐☐ / ☐☐ / ☐☐ Time ☐☐:☐☐

If not completed, give reason ____________________

How long is it since the patient was told of his/her diagnosis? ____________________

How long did it take the patient to complete the questionnaire?

- Less than 5 minutes ☐
- Between 5 and 10 minutes ☐
- Between 11 and 15 minutes ☐
- Longer than 15 minutes ☐

For the patient selected to complete the questionnaire on their own

Did the patient ask for any help? ☐ Yes ☐ No

If yes,

please specify what help was requested

____

____

please specify what help was given

____

____

For the patient selected for the researcher-administered questionnaire

Did the patient find any of the questions difficult? ☐ Yes ☐ No

If yes,

please specify which questions and what the difficulty was

____

____

____

____

Was the patient made anxious? ☐ Yes ☐ No

If yes,

please give details

____

____

____

____

Where did the patient complete the questionnaire?

(Please specify whether this was in a separate room alone, in a corner of the clinic, the ward or other

____

Thank you. Please now send the questionnaire and patient information sheet and consent form back to Cardiff University in the freepost envelope provided.

57727

PID - | | 4 | 5 |

Ymddiriedolaeth GIG Gogledd Orllewin Cymru
North West Wales NHS Trust

NHS WALES GIG CYMRU
Ymddiriedolaeth GIG Gogledd Cymru
North Wales NHS Trust

CID - ☐ GLAN CLWYD

☐ GWYNEDD

☐ MAELOR

Confidential Questionnaire

Looking At Your Symptoms Study

This Study has been funded by Cancer Research UK

Cardiff University / Prifysgol Caerdydd
Ysgol Glinigol Gogledd Cymru / North Wales Clinical School
Cancer Research UK
sewtu — South East Wales Trials Unit / Uned Ymchwil De-ddwyrain Cymru

Dear Patient

I am very grateful to you for agreeing to help us with this study.

I hope the information you supply will help us give people a diagnosis earlier.

If you feel you would like to talk to someone about this questionnaire at a later stage, please speak to the doctor or nurse who is looking after you, or be in touch with the research team, whose contact details are on your patient information sheet.

I can assure you that anything you tell us will be treated in the utmost confidence.

Yours sincerely

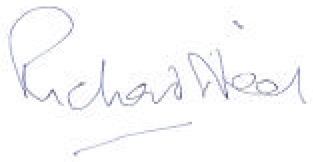

Dr Richard Neal
Senior Clinical Lecturer
Cardiff University
Chief Investigator, Symptoms Study

About your symptoms

These questions are about symptoms you noticed before your diagnosis. We are interested in symptoms which you think are related to your cancer.

Please put a cross in the appropriate box, or write an answer as required.

Looking back...

1. Did you have any symptoms that you feel were due to your cancer before you were diagnosed?

Yes ☐ **Please continue with Question 2**

No ☐ **Please go to Section 2 on Page 5**

2. What was the first symptom that made you think something might be wrong?

[ ]

Please go to Question 3

The following questions are about when you first noticed a symptom and when you first told your GP or nurse about it.

Please give an exact date if you can. Otherwise give your best estimate. (For example approximately how long ago, the month or season). You wish to refer to your diary or calendar if you have it with you.

Here is an example question:

Decrease in appetite

Did you have this symptom?

Yes ☒ **Please complete A then B or C below**

No ☐ **Please go to Section 2**

A When did you first notice this? d d / m m / y y **OR** Estimate '3 months ago' or 'June'

B When did you first tell your GP or nurse? 2 3 / 1 2 / 0 7 **OR** Estimate

OR

C Put a cross here if you didn't tell your GP or nurse ☐

3. Change in bowel habit

Did you have this symptom?

Yes ☐ **Please complete A then B or C below**

No ☐ **Please go to Question 4**

A When did you first notice this? ☐☐ / ☐☐ / ☐☐ **OR** ☐

B When did you first tell your GP or nurse? ☐☐ / ☐☐ / ☐☐ **OR** ☐

OR

C Put a cross here if you didn't tell your GP or nurse ☐

4. Bleeding from back passage

Did you have this symptom?

Yes ☐ **Please complete A then B or C below**

No ☐ **Please go to Section 2**

A When did you first notice this? ☐☐ / ☐☐ / ☐☐ **OR** ☐

B When did you first tell your GP or nurse? ☐☐ / ☐☐ / ☐☐ **OR** ☐

OR

C Put a cross here if you didn't tell your GP or nurse ☐

Section 2

Please give an exact date if you can. Otherwise give your best estimate (for example approximately how long ago, the month or the season). You may wish to refer to your diary or calendar if you have it with you.

Did you have any of the following?

1. Decrease in appetite

Did you have this?

Yes ☐ **Please complete A then B or C below**

No ☐ **Please go to Question 2**

A When did you first notice this? ☐☐ / ☐☐ / ☐☐ **OR** ☐

B When did you first tell your GP or nurse? ☐☐ / ☐☐ / ☐☐ **OR** ☐

OR

C Put a cross here if you didn't tell your GP or nurse ☐

2. Unexplained weight loss

Did you have this?

Yes ☐ **Please complete A then B or C below**

No ☐ **Please go to Question 3**

A When did you first notice this? ☐☐ / ☐☐ / ☐☐ **OR** ☐

B When did you first tell your GP or nurse? ☐☐ / ☐☐ / ☐☐ **OR** ☐

OR

C Put a cross here if you didn't tell your GP or nurse ☐

3. **Fatigue or tiredness that is unusual for you**

Did you have this?

Yes ☐ **Please complete A then B or C below**

No ☐ **Please go to Question 4**

A When did you first notice this? ☐☐ / ☐☐ / ☐☐ **OR** ☐

B When did you first tell your GP or nurse? ☐☐ / ☐☐ / ☐☐ **OR** ☐

OR

C Put a cross here if you didn't tell your GP or nurse ☐

4. **Feeling different 'in yourself' from usual**

Did you have this?

Yes ☐ **Please complete A then B or C below**

No ☐ **Please go to Question 5**

A When did you first notice this? ☐☐ / ☐☐ / ☐☐ **OR** ☐

B When did you first tell your GP or nurse? ☐☐ / ☐☐ / ☐☐ **OR** ☐

OR

C Put a cross here if you didn't tell your GP or nurse ☐

Please try and describe what the feeling was

If you had any further symptoms which you feel are relevant to your cancer diagnosis, please list them below. If not, please go to Question 7.

Please describe the symptom here and complete A then B or C below

5.

**A**	When did you first notice this?	☐☐ / ☐☐ / ☐☐ **OR** ☐
**B**	When did you first tell your GP or nurse?	☐☐ / ☐☐ / ☐☐ **OR** ☐
	**OR**	
**C**	Put a cross here if you didn't tell your GP or nurse	☐

If you had another symptom you feel is relevant, please write it below, otherwise go to Question 7.

Please describe the symptom here and complete A then B or C below

6.

**A**	When did you first notice this?	☐☐ / ☐☐ / ☐☐ **OR** ☐
**B**	When did you first tell your GP or nurse?	☐☐ / ☐☐ / ☐☐ **OR** ☐
	**OR**	
**C**	Put a cross here if you didn't tell your GP or nurse	☐

If you had further symptoms you would like to tell us about, there is space at the end of the Questionnaire for you to write them.

Did your GP send you for any tests?

Thinking back to the time immediately before you diagnosis, did your GP send you for any of the following tests?

Please cross the appropriate boxes

7. Were you sent for any of these?

	Yes	Not sure	No
Blood test(s)	☐	☐	☐
CT Scan	☐	☐	☐
Ultrasound scan	☐	☐	☐
Barium Enema	☐	☐	☐
Sigmoidoscopy or colonoscopy (Looking at bowel with internal camera)	☐	☐	☐
X-ray	☐	☐	☐

Please go to Question 8

About you

The next questions relate to general information about you. It would help us to know more about the background of the people completing the questionnaire.

8. Which best describes your employment status?

Please cross one box only

- Employed full-time ☐
- Employed part-time ☐
- Self employed full-time ☐
- Self employed part-time ☐
- Unemployed (seeking work) ☐
- Unemployed (not seeking work) ☐
- Retired ☐
- Student ☐
- Permanently sick/disabled ☐
- Temporarily sick/disabled ☐
- Looking after family/home ☐
- Other, please describe ______

9. What is your highest level of qualification?

Please cross one box only

- Degree (or equivalent) ☐
- Diploma (or equivalent) ☐
- 'A' level ☐
- GCSE / 'O' level ☐
- None ☐
- Other, please specify ______

10. **How would you describe your ethnicity**

Please cross one box only

White ☐
or
White British ☐
White Irish ☐
Other White background ☐

Mixed ☐
or
White & Black Carribean ☐
White & Black African ☐
White & Asian ☐
Other mixed background ☐

Black or Black British ☐
or
Carribean ☐
African ☐
Other Black background ☐

Asian or Asian British ☐
or
Indian ☐
Pakistani ☐
Bangladeshi ☐
Other Asian background ☐

Chinese or other ethnic group ☐
or
Chinese ☐
Other ethnic group ☐

11.

Do you live alone?

Yes ☐ **Please go to Question 12**

No ☐ **Please say who you live with below**

Who do you live with?
(e.g. wife, husband, partner, family member)

12. **Are you suffering from, or have you suffered from, any of the following in the past 2 years?**

Please cross any that are applicable to you

- Asthma ☐
- Chronic Obstructive Pulmonary Disease (COPD) ☐
- Other lung disease (e.g. fibrosis, bronchiectatis etc) ☐
- Heart disease ☐
- Anxiety or depression ☐
- Inflammatory bowel disease ☐
- Irritable bowel syndrome ☐
- Peptic ulcer ☐
- Previous cancer ☐
- Diabetes ☐
- Arthritis ☐

13. **About smoking**

Please cross the appropriate statement

- Are you a current smoker? ☐
- Are you an ex-smoker? ☐
- Are you a non-smoker (never smoked)? ☐

14. **Do you think you were more at risk of getting cancer because of your family history?**

Yes ☐

No ☐

15. **The following questions are about how completing the questionnaire made you feel.**

A number of statements which people have used to describe themselves are given below. Read each statement and then cross the most appropriate box to indicate how you feel right now, at the moment. There are no right or wrong answers. Do not spend too much time on any one statement but give the answer which seems to describe your present feeling best.

		Not at all	Somewhat	Moderately	Very much
1.	I feel calm	☐	☐	☐	☐
2.	I am tense	☐	☐	☐	☐
3.	I feel upset	☐	☐	☐	☐
4.	I am relaxed	☐	☐	☐	☐
5.	I feel content	☐	☐	☐	☐
6.	I am worried	☐	☐	☐	☐

Please make sure you have answered all the questions.

If you have any other comments or there is anything else you would like to tell us about when you first noticed your symptoms, please do so using the space below.

If you feel you would like to talk to someone about this questionnaire at a later stage, please speak to the doctor or nurse who is looking after you, or be in touch with the research team, whose contact details are on your patient information sheet

Thank you for completing the questionnaire

Please give it to the research nurse

The next page is for the Researcher to complete

57727

Researcher - Post-completion Sheet

Researcher initials: ☐☐☐

Date questionnaire completed: Date ☐☐ / ☐☐ / ☐☐ Time ☐☐ : ☐☐

If not completed, give reason ☐

How long is it since the patient was told of his/her diagnosis? ☐

How long did it take the patient to complete the questionnaire?

- Less than 5 minutes ☐
- Between 5 and 10 minutes ☐
- Between 11 and 15 minutes ☐
- Longer than 15 minutes ☐

For the patient selected to complete the questionnaire on their own

Did the patient ask for any help? ☐ Yes ☐ No

If yes,

please specify what help was requested

____

____

please specify what help was given

____

____

For the patient selected for the researcher-administered questionnaire

Did the patient find any of the questions difficult? ☐ Yes ☐ No

If yes,

please specify which questions and what the difficulty was

____

____

____

____

Was the patient made anxious? ☐ Yes ☐ No

If yes,

please give details

____

____

____

____

Where did the patient complete the questionnaire?
(Please specify whether this was in a separate room alone, in a corner of the clinic, the ward or other

____

Thank you. Please now send the questionnaire and patient information sheet and consent form back to Cardiff University in the freepost envelope provided.

57727

PID - | | 4 | 6 |

Ymddiriedolaeth GIG Gogledd Orllewin Cymru
North West Wales NHS Trust

NHS WALES GIG CYMRU
Ymddiriedolaeth GIG Gogledd Cymru
North Wales NHS Trust

CID -
☐ GLAN CLWYD
☐ GWYNEDD
☐ MAELOR

Confidential Questionnaire

Looking At Your Symptoms Study

This Study has been funded by Cancer Research UK

Cardiff University / Prifysgol Caerdydd
Ysgol Glinigol Gogledd Cymru / North Wales Clinical School
Cancer Research UK
sewtu — South East Wales Trials Unit / Uned Ymchwil De-ddwyrain Cymru

Dear Patient

I am very grateful to you for agreeing to help us with this study.

I hope the information you supply will help us give people a diagnosis earlier.

If you feel you would like to talk to someone about this questionnaire at a later stage, please speak to the doctor or nurse who is looking after you, or be in touch with the research team, whose contact details are on your patient information sheet.

I can assure you that anything you tell us will be treated in the utmost confidence.

Yours sincerely

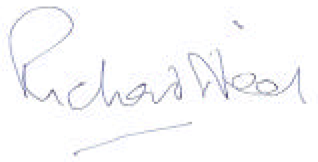

Dr Richard Neal
Senior Clinical Lecturer
Cardiff University
Chief Investigator, Symptoms Study

About your symptoms

These questions are about symptoms you noticed before your diagnosis. We are interested in symptoms which you think are related to your cancer.

Please put a cross in the appropriate box, or write an answer as required.

Looking back...

1. Did you have any symptoms that you feel were due to your cancer before you were diagnosed?

Yes ☐ **Please continue with Question 2**

No ☐ **Please go to Section 2 on Page 5**

2. What was the first symptom that made you think something might be wrong?

☐

Please go to Question 3

The following questions are about when you first noticed a symptom and when you first told your GP or nurse about it.

Please give an exact date if you can. Otherwise give your best estimate. (For example approximately how long ago, the month or season). You wish to refer to your diary or calendar if you have it with you.

Here is an example question:

Decrease in appetite

Did you have this symptom?

Yes ☒ **Please complete A then B or C below**

No ☐ **Please go to Section 2**

A When did you first notice this? d d / m m / y y **OR** Estimate '3 months ago' or 'June'

B When did you first tell your GP or nurse? 2 3 / 1 2 / 0 7 **OR** Estimate

OR

C Put a cross here if you didn't tell your GP or nurse ☐

3. Change in bowel habit

Did you have this symptom?

Yes ☐ **Please complete A then B or C below**

No ☐ **Please go to Question 4**

A When did you first notice this? ☐☐ / ☐☐ / ☐☐ **OR** ☐

B When did you first tell your GP or nurse? ☐☐ / ☐☐ / ☐☐ **OR** ☐

OR

C Put a cross here if you didn't tell your GP or nurse ☐

4. Bleeding from back passage

Did you have this symptom?

Yes ☐ **Please complete A then B or C below**

No ☐ **Please go to Section 2**

A When did you first notice this? ☐☐ / ☐☐ / ☐☐ **OR** ☐

B When did you first tell your GP or nurse? ☐☐ / ☐☐ / ☐☐ **OR** ☐

OR

C Put a cross here if you didn't tell your GP or nurse ☐

Section 2

Please give an <u>exact</u> date if you can. Otherwise give your best <u>estimate</u> (for example approximately how long ago, the month or the season). You may wish to refer to your diary or calendar if you have it with you.

Did you have any of the following?

1. Decrease in appetite

Did you have this?

Yes ☐ **Please complete A then B or C below**

No ☐ **Please go to Question 2**

A When did you first notice this? ☐☐ / ☐☐ / ☐☐ **OR** ☐

B When did you first tell your GP or nurse? ☐☐ / ☐☐ / ☐☐ **OR** ☐

OR

C Put a cross here if you didn't tell your GP or nurse ☐

2. Unexplained weight loss

Did you have this?

Yes ☐ **Please complete A then B or C below**

No ☐ **Please go to Question 3**

A When did you first notice this? ☐☐ / ☐☐ / ☐☐ **OR** ☐

B When did you first tell your GP or nurse? ☐☐ / ☐☐ / ☐☐ **OR** ☐

OR

C Put a cross here if you didn't tell your GP or nurse ☐

3. **Fatigue or tiredness that is unusual for you**

Did you have this?

Yes ☐ **Please complete A then B or C below**

No ☐ **Please go to Question 4**

A When did you <u>first notice</u> this? ☐☐ / ☐☐ / ☐☐ **OR** ☐

B When did you <u>first tell</u> your GP or nurse? ☐☐ / ☐☐ / ☐☐ **OR** ☐

OR

C Put a cross here if you <u>didn't tell</u> your GP or nurse ☐

4. **Feeling different 'in yourself' from usual**

Did you have this?

Yes ☐ **Please complete A then B or C below**

No ☐ **Please go to Question 5**

A When did you <u>first notice</u> this? ☐☐ / ☐☐ / ☐☐ **OR** ☐

B When did you <u>first tell</u> your GP or nurse? ☐☐ / ☐☐ / ☐☐ **OR** ☐

OR

C Put a cross here if you <u>didn't tell</u> your GP or nurse ☐

Please try and describe what the feeling was

If you had any further symptoms which you feel are relevant to your cancer diagnosis, please list them below. If not, please go to Question 7.

Please describe the symptom here and complete A then B or C below

5.

**A**	When did you first notice this?	☐☐ / ☐☐ / ☐☐ **OR** ☐
**B**	When did you first tell your GP or nurse?	☐☐ / ☐☐ / ☐☐ **OR** ☐
	**OR**	
**C**	Put a cross here if you didn't tell your GP or nurse	☐

If you had another symptom you feel is relevant, please write it below, otherwise go to Question 7.

Please describe the symptom here and complete A then B or C below

6.

**A**	When did you first notice this?	☐☐ / ☐☐ / ☐☐ **OR** ☐
**B**	When did you first tell your GP or nurse?	☐☐ / ☐☐ / ☐☐ **OR** ☐
	**OR**	
**C**	Put a cross here if you didn't tell your GP or nurse	☐

If you had further symptoms you would like to tell us about, there is space at the end of the Questionnaire for you to write them.

Did your GP send you for any tests?

Thinking back to the time immediately before you diagnosis, did your GP send you for any of the following tests?

Please cross the appropriate boxes

7. **Were you sent for any of these?**

	Yes	Not sure	No
Blood test(s)	☐	☐	☐
CT Scan	☐	☐	☐
Ultrasound scan	☐	☐	☐
Barium Enema	☐	☐	☐
Sigmoidoscopy or colonoscopy (Looking at bowel with internal camera)	☐	☐	☐
X-ray	☐	☐	☐

Please go to Question 8

About you

The next questions relate to general information about you. It would help us to know more about the background of the people completing the questionnaire.

8. Which best describes your employment status?

Please cross one box only

- Employed full-time ☐
- Employed part-time ☐
- Self employed full-time ☐
- Self employed part-time ☐
- Unemployed (seeking work) ☐
- Unemployed (not seeking work) ☐
- Retired ☐
- Student ☐
- Permanently sick/disabled ☐
- Temporarily sick/disabled ☐
- Looking after family/home ☐
- Other, please describe ______

9. What is your highest level of qualification?

Please cross one box only

- Degree (or equivalent) ☐
- Diploma (or equivalent) ☐
- 'A' level ☐
- GCSE / 'O' level ☐
- None ☐
- Other, please specify ______

10. How would you describe your ethnicity

Please cross one box only

White ☐
or
- White British ☐
- White Irish ☐
- Other White background ☐

Mixed ☐
or
- White & Black Carribean ☐
- White & Black African ☐
- White & Asian ☐
- Other mixed background ☐

Black or Black British ☐
or
- Carribean ☐
- African ☐
- Other Black background ☐

Asian or Asian British ☐
or
- Indian ☐
- Pakistani ☐
- Bangladeshi ☐
- Other Asian background ☐

Chinese or other ethnic group ☐
or
- Chinese ☐
- Other ethnic group ☐

11.

Do you live alone?

Yes ☐ **Please go to Question 12**

No ☐ **Please say who you live with below**

Who do you live with?
(e.g. wife, husband, partner, family member)

12. Are you suffering from, or have you suffered from, any of the following in the past 2 years?

Please cross any that are applicable to you

Asthma	☐
Chronic Obstructive Pulmonary Disease (COPD)	☐
Other lung disease (e.g. fibrosis, bronchiectatis etc)	☐
Heart disease	☐
Anxiety or depression	☐
Inflammatory bowel disease	☐
Irritable bowel syndrome	☐
Peptic ulcer	☐
Previous cancer	☐
Diabetes	☐
Arthritis	☐

13. About smoking

Please cross the appropriate statement

Are you a current smoker?	☐
Are you an ex-smoker?	☐
Are you a non-smoker (never smoked)?	☐

14. Do you think you were more at risk of getting cancer because of your family history?

Yes ☐

No ☐

15. **The following questions are about how completing the questionnaire made you feel.**

A number of statements which people have used to describe themselves are given below. Read each statement and then cross the most appropriate box to indicate how you feel right now, at the moment. There are no right or wrong answers. Do not spend too much time on any one statement but give the answer which seems to describe your present feeling best.

		Not at all	Somewhat	Moderately	Very much
1.	I feel calm	☐	☐	☐	☐
2.	I am tense	☐	☐	☐	☐
3.	I feel upset	☐	☐	☐	☐
4.	I am relaxed	☐	☐	☐	☐
5.	I feel content	☐	☐	☐	☐
6.	I am worried	☐	☐	☐	☐

Please make sure you have answered all the questions.

If you have any other comments or there is anything else you would like to tell us about when you first noticed your symptoms, please do so using the space below.

If you feel you would like to talk to someone about this questionnaire at a later stage, please speak to the doctor or nurse who is looking after you, or be in touch with the research team, whose contact details are on your patient information sheet

Thank you for completing the questionnaire

Please give it to the research nurse

The next page is for the Researcher to complete

57727

4 6

Researcher - Post-completion Sheet

Researcher initials: ☐☐☐

Date questionnaire completed: Date ☐☐ / ☐☐ / ☐☐ Time ☐☐ : ☐☐

If not completed, give reason ☐

How long is it since the patient was told of his/her diagnosis? ☐

How long did it take the patient to complete the questionnaire?

- Less than 5 minutes ☐
- Between 5 and 10 minutes ☐
- Between 11 and 15 minutes ☐
- Longer than 15 minutes ☐

For the patient selected to complete the questionnaire on their own

Did the patient ask for any help? ☐ Yes ☐ No

If yes,

please specify what help was requested

____

____

please specify what help was given

____

____

For the patient selected for the researcher-administered questionnaire

Did the patient find any of the questions difficult? ☐ Yes ☐ No

If yes,

please specify which questions and what the difficulty was

____

____

____

____

Was the patient made anxious? ☐ Yes ☐ No

If yes,

please give details

____

____

____

____

Where did the patient complete the questionnaire?
(Please specify whether this was in a separate room alone, in a corner of the clinic, the ward or other

____

Thank you. Please now send the questionnaire and patient information sheet and consent form back to Cardiff University in the freepost envelope provided.

57727

PID - | | 4 | 7 |

Ymddiriedolaeth GIG Gogledd Orllewin Cymru
North West Wales NHS Trust

NHS WALES GIG CYMRU
Ymddiriedolaeth GIG Gogledd Cymru
North Wales NHS Trust

CID - ☐ GLAN CLWYD
☐ GWYNEDD
☐ MAELOR

Confidential Questionnaire

Looking At Your Symptoms Study

This Study has been funded by Cancer Research UK

Cardiff University / Prifysgol Caerdydd · Ysgol Glinigol Gogledd Cymru / North Wales Clinical School · Cancer Research UK · sewtu South East Wales Trials Unit / Uned Ymchwil De-ddwyrain Cymru

Dear Patient

I am very grateful to you for agreeing to help us with this study.

I hope the information you supply will help us give people a diagnosis earlier.

If you feel you would like to talk to someone about this questionnaire at a later stage, please speak to the doctor or nurse who is looking after you, or be in touch with the research team, whose contact details are on your patient information sheet.

I can assure you that anything you tell us will be treated in the utmost confidence.

Yours sincerely

Richard Neal

Dr Richard Neal
Senior Clinical Lecturer
Cardiff University
Chief Investigator, Symptoms Study

About your symptoms

These questions are about symptoms you noticed before your diagnosis. We are interested in symptoms which you think are related to your cancer.

Please put a cross in the appropriate box, or write an answer as required.

Looking back...

1. Did you have any symptoms that you feel were due to your cancer before you were diagnosed?

Yes ☐ **Please continue with Question 2**

No ☐ **Please go to Section 2 on Page 5**

2. What was the first symptom that made you think something might be wrong?

[ ]

Please go to Question 3

The following questions are about when you first noticed a symptom and when you first told your GP or nurse about it.

Please give an exact date if you can. Otherwise give your best estimate. (For example approximately how long ago, the month or season). You wish to refer to your diary or calendar if you have it with you.

Here is an example question:

Decrease in appetite

Did you have this symptom?

Yes ☒ **Please complete A then B or C below**

No ☐ **Please go to Section 2**

A When did you first notice this? d d / m m / y y **OR** Estimate: '3 months ago' or 'June'

B When did you first tell your GP or nurse? 2 3 / 1 2 / 0 7 **OR** Estimate

OR

C Put a cross here if you didn't tell your GP or nurse ☐

3. Change in bowel habit

Did you have this symptom?

Yes ☐ **Please complete A then B or C below**

No ☐ **Please go to Question 4**

A When did you first notice this? ☐☐ / ☐☐ / ☐☐ **OR** ☐

B When did you first tell your GP or nurse? ☐☐ / ☐☐ / ☐☐ **OR** ☐

OR

C Put a cross here if you didn't tell your GP or nurse ☐

4. Bleeding from back passage

Did you have this symptom?

Yes ☐ **Please complete A then B or C below**

No ☐ **Please go to Section 2**

A When did you first notice this? ☐☐ / ☐☐ / ☐☐ **OR** ☐

B When did you first tell your GP or nurse? ☐☐ / ☐☐ / ☐☐ **OR** ☐

OR

C Put a cross here if you didn't tell your GP or nurse ☐

Section 2

Please give an exact date if you can. Otherwise give your best estimate (for example approximately how long ago, the month or the season). You may wish to refer to your diary or calendar if you have it with you.

Did you have any of the following?

1. Decrease in appetite

Did you have this?

Yes ☐ **Please complete A then B or C below**

No ☐ **Please go to Question 2**

A When did you first notice this? ☐☐ / ☐☐ / ☐☐ **OR** ☐

B When did you first tell your GP or nurse? ☐☐ / ☐☐ / ☐☐ **OR** ☐

OR

C Put a cross here if you didn't tell your GP or nurse ☐

2. Unexplained weight loss

Did you have this?

Yes ☐ **Please complete A then B or C below**

No ☐ **Please go to Question 3**

A When did you first notice this? ☐☐ / ☐☐ / ☐☐ **OR** ☐

B When did you first tell your GP or nurse? ☐☐ / ☐☐ / ☐☐ **OR** ☐

OR

C Put a cross here if you didn't tell your GP or nurse ☐

3. **Fatigue or tiredness that is unusual for you**

Did you have this?

Yes ☐ **Please complete A then B or C below**

No ☐ **Please go to Question 4**

A When did you first notice this? ☐☐ / ☐☐ / ☐☐ **OR** ☐

B When did you first tell your GP or nurse? ☐☐ / ☐☐ / ☐☐ **OR** ☐

OR

C Put a cross here if you didn't tell your GP or nurse ☐

4. **Feeling different 'in yourself' from usual**

Did you have this?

Yes ☐ **Please complete A then B or C below**

No ☐ **Please go to Question 5**

A When did you first notice this? ☐☐ / ☐☐ / ☐☐ **OR** ☐

B When did you first tell your GP or nurse? ☐☐ / ☐☐ / ☐☐ **OR** ☐

OR

C Put a cross here if you didn't tell your GP or nurse ☐

Please try and describe what the feeling was

If you had any further symptoms which you feel are relevant to your cancer diagnosis, please list them below. If not, please go to Question 7.

Please describe the symptom here and complete A then B or C below

5.

A	When did you first notice this?	_ _ / _ _ / _ _ **OR**
B	When did you first tell your GP or nurse?	_ _ / _ _ / _ _ **OR**
	**OR**	
C	Put a cross here if you didn't tell your GP or nurse	☐

If you had another symptom you feel is relevant, please write it below, otherwise go to Question 7.

Please describe the symptom here and complete A then B or C below

6.

A	When did you first notice this?	_ _ / _ _ / _ _ **OR**
B	When did you first tell your GP or nurse?	_ _ / _ _ / _ _ **OR**
	**OR**	
C	Put a cross here if you didn't tell your GP or nurse	☐

If you had further symptoms you would like to tell us about, there is space at the end of the Questionnaire for you to write them.

Did your GP send you for any tests?

Thinking back to the time immediately before you diagnosis, did your GP send you for any of the following tests?

Please cross the appropriate boxes

7. Were you sent for any of these?

	Yes	Not sure	No
Blood test(s)	☐	☐	☐
CT Scan	☐	☐	☐
Ultrasound scan	☐	☐	☐
Barium Enema	☐	☐	☐
Sigmoidoscopy or colonoscopy (Looking at bowel with internal camera)	☐	☐	☐
X-ray	☐	☐	☐

Please go to Question 8

About you

The next questions relate to general information about you. It would help us to know more about the background of the people completing the questionnaire.

8. Which best describes your employment status?

Please cross one box only

- Employed full-time ☐
- Employed part-time ☐
- Self employed full-time ☐
- Self employed part-time ☐
- Unemployed (seeking work) ☐
- Unemployed (not seeking work) ☐
- Retired ☐
- Student ☐
- Permanently sick/disabled ☐
- Temporarily sick/disabled ☐
- Looking after family/home ☐
- Other, please describe ________

9. What is your highest level of qualification?

Please cross one box only

- Degree (or equivalent) ☐
- Diploma (or equivalent) ☐
- 'A' level ☐
- GCSE / 'O' level ☐
- None ☐
- Other, please specify ________

10. **How would you describe your ethnicity**

Please cross one box only

White ☐
or
- White British ☐
- White Irish ☐
- Other White background ☐

Mixed ☐
or
- White & Black Carribean ☐
- White & Black African ☐
- White & Asian ☐
- Other mixed background ☐

Black or Black British ☐
or
- Carribean ☐
- African ☐
- Other Black background ☐

Asian or Asian British ☐
or
- Indian ☐
- Pakistani ☐
- Bangladeshi ☐
- Other Asian background ☐

Chinese or other ethnic group ☐
or
- Chinese ☐
- Other ethnic group ☐

11.

Do you live alone?

Yes ☐ **Please go to Question 12**

No ☐ **Please say who you live with below**

Who do you live with?
(e.g. wife, husband, partner, family member)

12. Are you suffering from, or have you suffered from, any of the following in the past 2 years?

Please cross any that are applicable to you

- Asthma ☐
- Chronic Obstructive Pulmonary Disease (COPD) ☐
- Other lung disease (e.g. fibrosis, bronchiectatis etc) ☐
- Heart disease ☐
- Anxiety or depression ☐
- Inflammatory bowel disease ☐
- Irritable bowel syndrome ☐
- Peptic ulcer ☐
- Previous cancer ☐
- Diabetes ☐
- Arthritis ☐

13. About smoking

Please cross the appropriate statement

- Are you a current smoker? ☐
- Are you an ex-smoker? ☐
- Are you a non-smoker (never smoked)? ☐

14. Do you think you were more at risk of getting cancer because of your family history?

Yes ☐

No ☐

15. The following questions are about how completing the questionnaire made you feel.

A number of statements which people have used to describe themselves are given below. Read each statement and then cross the most appropriate box to indicate how you feel right now, at the moment. There are no right or wrong answers. Do not spend too much time on any one statement but give the answer which seems to describe your present feeling best.

		Not at all	Somewhat	Moderately	Very much
1.	I feel calm	☐	☐	☐	☐
2.	I am tense	☐	☐	☐	☐
3.	I feel upset	☐	☐	☐	☐
4.	I am relaxed	☐	☐	☐	☐
5.	I feel content	☐	☐	☐	☐
6.	I am worried	☐	☐	☐	☐

Please make sure you have answered all the questions.

If you have any other comments or there is anything else you would like to tell us about when you first noticed your symptoms, please do so using the space below.

If you feel you would like to talk to someone about this questionnaire at a later stage, please speak to the doctor or nurse who is looking after you, or be in touch with the research team, whose contact details are on your patient information sheet

Thank you for completing the questionnaire

Please give it to the research nurse

The next page is for the Researcher to complete

Researcher - Post-completion Sheet

Researcher initials: ☐☐☐

Date questionnaire completed: Date ☐☐ / ☐☐ / ☐☐ Time ☐☐ : ☐☐

If not completed, give reason ☐

How long is it since the patient was told of his/her diagnosis? ☐

How long did it take the patient to complete the questionnaire?

- Less than 5 minutes ☐
- Between 5 and 10 minutes ☐
- Between 11 and 15 minutes ☐
- Longer than 15 minutes ☐

For the patient selected to complete the questionnaire on their own

Did the patient ask for any help? ☐ Yes ☐ No

If yes,

please specify what help was requested

____

____

please specify what help was given

____

____

For the patient selected for the researcher-administered questionnaire

Did the patient find any of the questions difficult? ☐ Yes ☐ No

If yes,

please specify which questions and what the difficulty was

____

____

____

____

Was the patient made anxious? ☐ Yes ☐ No

If yes,

please give details

____

____

____

____

Where did the patient complete the questionnaire?

(Please specify whether this was in a separate room alone, in a corner of the clinic, the ward or other

____

Thank you. Please now send the questionnaire and patient information sheet and consent form back to Cardiff University in the freepost envelope provided.

57727

PID - | | 4 | 8 |

Ymddiriedolaeth GIG Gogledd Orllewin Cymru
North West Wales NHS Trust

NHS WALES GIG CYMRU
Ymddiriedolaeth GIG Gogledd Cymru
North Wales NHS Trust

CID -
- ☐ GLAN CLWYD
- ☐ GWYNEDD
- ☐ MAELOR

Confidential Questionnaire

Looking At Your Symptoms Study

This Study has been funded by Cancer Research UK

Cardiff University / Prifysgol Caerdydd

Ysgol Glinigol Gogledd Cymru / North Wales Clinical School

Cancer Research UK

sewtu South East Wales Trials Unit / Uned Ymchwil De-ddwyrain Cymru

Dear Patient

I am very grateful to you for agreeing to help us with this study.

I hope the information you supply will help us give people a diagnosis earlier.

If you feel you would like to talk to someone about this questionnaire at a later stage, please speak to the doctor or nurse who is looking after you, or be in touch with the research team, whose contact details are on your patient information sheet.

I can assure you that anything you tell us will be treated in the utmost confidence.

Yours sincerely

Dr Richard Neal
Senior Clinical Lecturer
Cardiff University
Chief Investigator, Symptoms Study

About your symptoms

These questions are about symptoms you noticed before your diagnosis. We are interested in symptoms which you think are related to your cancer.

Please put a cross in the appropriate box, or write an answer as required.

Looking back...

1. Did you have any symptoms that you feel were due to your cancer before you were diagnosed?

Yes ☐ **Please continue with Question 2**

No ☐ **Please go to Section 2 on Page 5**

2. What was the first symptom that made you think something might be wrong?

☐

Please go to Question 3

The following questions are about when you first noticed a symptom and when you first told your GP or nurse about it.

Please give an exact date if you can. Otherwise give your best estimate. (For example approximately how long ago, the month or season). You wish to refer to your diary or calendar if you have it with you.

Here is an example question:

Decrease in appetite

Did you have this symptom?

Yes ☒ **Please complete A then B or C below**

No ☐ **Please go to Section 2**

A When did you first notice this? d d / m m / y y **OR** Estimate '3 months ago' or 'June'

B When did you first tell your GP or nurse? 2 3 / 1 2 / 0 7 **OR** Estimate

OR

C Put a cross here if you didn't tell your GP or nurse ☐

3. Change in bowel habit

Did you have this symptom?

Yes ☐ **Please complete A then B or C below**

No ☐ **Please go to Question 4**

A When did you first notice this? ☐☐ / ☐☐ / ☐☐ **OR** ☐

B When did you first tell your GP or nurse? ☐☐ / ☐☐ / ☐☐ **OR** ☐

OR

C Put a cross here if you didn't tell your GP or nurse ☐

4. Bleeding from back passage

Did you have this symptom?

Yes ☐ **Please complete A then B or C below**

No ☐ **Please go to Section 2**

A When did you first notice this? ☐☐ / ☐☐ / ☐☐ **OR** ☐

B When did you first tell your GP or nurse? ☐☐ / ☐☐ / ☐☐ **OR** ☐

OR

C Put a cross here if you didn't tell your GP or nurse ☐

Section 2

Please give an exact date if you can. Otherwise give your best estimate (for example approximately how long ago, the month or the season). You may wish to refer to your diary or calendar if you have it with you.

Did you have any of the following?

1. Decrease in appetite

Did you have this?

Yes ☐ **Please complete A then B or C below**

No ☐ **Please go to Question 2**

A When did you first notice this? ☐☐ / ☐☐ / ☐☐ **OR** ☐

B When did you first tell your GP or nurse? ☐☐ / ☐☐ / ☐☐ **OR** ☐

OR

C Put a cross here if you didn't tell your GP or nurse ☐

2. Unexplained weight loss

Did you have this?

Yes ☐ **Please complete A then B or C below**

No ☐ **Please go to Question 3**

A When did you first notice this? ☐☐ / ☐☐ / ☐☐ **OR** ☐

B When did you first tell your GP or nurse? ☐☐ / ☐☐ / ☐☐ **OR** ☐

OR

C Put a cross here if you didn't tell your GP or nurse ☐

3. **Fatigue or tiredness that is unusual for you**

Did you have this?

Yes ☐ **Please complete A then B or C below**

No ☐ **Please go to Question 4**

A When did you first notice this? ☐☐ / ☐☐ / ☐☐ **OR** ☐

B When did you first tell your GP or nurse? ☐☐ / ☐☐ / ☐☐ **OR** ☐

OR

C Put a cross here if you didn't tell your GP or nurse ☐

4. **Feeling different 'in yourself' from usual**

Did you have this?

Yes ☐ **Please complete A then B or C below**

No ☐ **Please go to Question 5**

A When did you first notice this? ☐☐ / ☐☐ / ☐☐ **OR** ☐

B When did you first tell your GP or nurse? ☐☐ / ☐☐ / ☐☐ **OR** ☐

OR

C Put a cross here if you didn't tell your GP or nurse ☐

Please try and describe what the feeling was

If you had any further symptoms which you feel are relevant to your cancer diagnosis, please list them below. If not, please go to Question 7.

Please describe the symptom here and complete A then B or C below

5.

**A**	When did you first notice this?	☐☐ / ☐☐ / ☐☐ **OR** ☐
**B**	When did you first tell your GP or nurse?	☐☐ / ☐☐ / ☐☐ **OR** ☐
	**OR**	
**C**	Put a cross here if you didn't tell your GP or nurse	☐

If you had another symptom you feel is relevant, please write it below, otherwise go to Question 7.

Please describe the symptom here and complete A then B or C below

6.

**A**	When did you first notice this?	☐☐ / ☐☐ / ☐☐ **OR** ☐
**B**	When did you first tell your GP or nurse?	☐☐ / ☐☐ / ☐☐ **OR** ☐
	**OR**	
**C**	Put a cross here if you didn't tell your GP or nurse	☐

If you had further symptoms you would like to tell us about, there is space at the end of the Questionnaire for you to write them.

Did your GP send you for any tests?

Thinking back to the time immediately before you diagnosis, did your GP send you for any of the following tests?

Please cross the appropriate boxes

7. Were you sent for any of these?

	Yes	Not sure	No
Blood test(s)	☐	☐	☐
CT Scan	☐	☐	☐
Ultrasound scan	☐	☐	☐
Barium Enema	☐	☐	☐
Sigmoidoscopy or colonoscopy (Looking at bowel with internal camera)	☐	☐	☐
X-ray	☐	☐	☐

Please go to Question 8

About you

The next questions relate to general information about you. It would help us to know more about the background of the people completing the questionnaire.

8. Which best describes your employment status?

Please cross one box only

- Employed full-time ☐
- Employed part-time ☐
- Self employed full-time ☐
- Self employed part-time ☐
- Unemployed (seeking work) ☐
- Unemployed (not seeking work) ☐
- Retired ☐
- Student ☐
- Permanently sick/disabled ☐
- Temporarily sick/disabled ☐
- Looking after family/home ☐
- Other, please describe ☐

9. What is your highest level of qualification?

Please cross one box only

- Degree (or equivalent) ☐
- Diploma (or equivalent) ☐
- 'A' level ☐
- GCSE / 'O' level ☐
- None ☐
- Other, please specify ☐

10. **How would you describe your ethnicity**

Please cross one box only

White ☐
or
White British ☐
White Irish ☐
Other White background ☐

Mixed ☐
or
White & Black Carribean ☐
White & Black African ☐
White & Asian ☐
Other mixed background ☐

Black or Black British ☐
or
Carribean ☐
African ☐
Other Black background ☐

Asian or Asian British ☐
or
Indian ☐
Pakistani ☐
Bangladeshi ☐
Other Asian background ☐

Chinese or other ethnic group ☐
or
Chinese ☐
Other ethnic group ☐

11.

Do you live alone?

Yes ☐ **Please go to Question 12**

No ☐ **Please say who you live with below**

Who do you live with?
(e.g. wife, husband, partner, family member)

12. **Are you suffering from, or have you suffered from, any of the following in the past 2 years?**

Please cross any that are applicable to you

- Asthma ☐
- Chronic Obstructive Pulmonary Disease (COPD) ☐
- Other lung disease (e.g. fibrosis, bronchiectatis etc) ☐
- Heart disease ☐
- Anxiety or depression ☐
- Inflammatory bowel disease ☐
- Irritable bowel syndrome ☐
- Peptic ulcer ☐
- Previous cancer ☐
- Diabetes ☐
- Arthritis ☐

13. **About smoking**

Please cross the appropriate statement

- Are you a current smoker? ☐
- Are you an ex-smoker? ☐
- Are you a non-smoker (never smoked)? ☐

14. **Do you think you were more at risk of getting cancer because of your family history?**

Yes ☐

No ☐

15. The following questions are about how completing the questionnaire made you feel.

A number of statements which people have used to describe themselves are given below. Read each statement and then cross the most appropriate box to indicate how you feel right now, at the moment. There are no right or wrong answers. Do not spend too much time on any one statement but give the answer which seems to describe your present feeling best.

		Not at all	Somewhat	Moderately	Very much
1.	I feel calm	☐	☐	☐	☐
2.	I am tense	☐	☐	☐	☐
3.	I feel upset	☐	☐	☐	☐
4.	I am relaxed	☐	☐	☐	☐
5.	I feel content	☐	☐	☐	☐
6.	I am worried	☐	☐	☐	☐

Please make sure you have answered all the questions.

If you have any other comments or there is anything else you would like to tell us about when you first noticed your symptoms, please do so using the space below.

If you feel you would like to talk to someone about this questionnaire at a later stage, please speak to the doctor or nurse who is looking after you, or be in touch with the research team, whose contact details are on your patient information sheet

Thank you for completing the questionnaire

Please give it to the research nurse

The next page is for the Researcher to complete

57727

4 8

Researcher - Post-completion Sheet

Researcher initials: ☐☐☐

Date questionnaire completed: Date ☐☐ / ☐☐ / ☐☐ Time ☐☐:☐☐

If not completed, give reason ☐

How long is it since the patient was told of his/her diagnosis? ☐

How long did it take the patient to complete the questionnaire?

- Less than 5 minutes ☐
- Between 5 and 10 minutes ☐
- Between 11 and 15 minutes ☐
- Longer than 15 minutes ☐

For the patient selected to <u>complete the questionnaire on their own</u>

Did the patient ask for any help? ☐ Yes ☐ No

If yes,

please specify what help was requested

____

____

please specify what help was given

____

____

For the patient selected for <u>the researcher-administered questionnaire</u>

Did the patient find any of the questions difficult? ☐ Yes ☐ No

If yes,

please specify which questions and what the difficulty was

____

____

____

____

Was the patient made anxious? ☐ Yes ☐ No

If yes,

please give details

____

____

____

____

Where did the patient complete the questionnaire?
(Please specify whether this was in a separate room alone, in a corner of the clinic, the ward or other

____

Thank you. Please now send the questionnaire and patient information sheet and consent form back to Cardiff University in the freepost envelope provided.

57727

PID - 4 9

Ymddiriedolaeth GIG Gogledd Orllewin Cymru
North West Wales NHS Trust

NHS WALES GIG CYMRU
Ymddiriedolaeth GIG Gogledd Cymru
North Wales NHS Trust

CID - ☐ GLAN CLWYD
☐ GWYNEDD
☐ MAELOR

Confidential Questionnaire

Looking At Your Symptoms Study

This Study has been funded by Cancer Research UK

Cardiff University / Prifysgol Caerdydd
Ysgol Glinigol Gogledd Cymru / North Wales Clinical School
Cancer Research UK
sewtu — South East Wales Trials Unit / Uned Ymchwil De-ddwyrain Cymru

Dear Patient

I am very grateful to you for agreeing to help us with this study.

I hope the information you supply will help us give people a diagnosis earlier.

If you feel you would like to talk to someone about this questionnaire at a later stage, please speak to the doctor or nurse who is looking after you, or be in touch with the research team, whose contact details are on your patient information sheet.

I can assure you that anything you tell us will be treated in the utmost confidence.

Yours sincerely

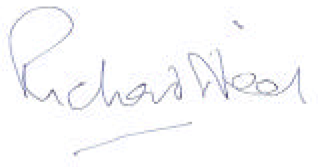

Dr Richard Neal
Senior Clinical Lecturer
Cardiff University
Chief Investigator, Symptoms Study

About your symptoms

These questions are about symptoms you noticed before your diagnosis. We are interested in symptoms which you think are related to your cancer.

Please put a cross in the appropriate box, or write an answer as required.

Looking back...

1. Did you have any symptoms that you feel were due to your cancer before you were diagnosed?

Yes ☐ **Please continue with Question 2**

No ☐ **Please go to Section 2 on Page 5**

2. What was the first symptom that made you think something might be wrong?

[ ]

Please go to Question 3

The following questions are about when you first noticed a symptom and when you first told your GP or nurse about it.

Please give an exact date if you can. Otherwise give your best estimate. (For example approximately how long ago, the month or season). You wish to refer to your diary or calendar if you have it with you.

Here is an example question:

Decrease in appetite

Did you have this symptom?

Yes ☒ **Please complete A then B or C below**

No ☐ **Please go to Section 2**

A When did you first notice this? d d / m m / y y **OR** Estimate: '3 months ago' or 'June'

B When did you first tell your GP or nurse? 2 3 / 1 2 / 0 7 **OR** Estimate

OR

C Put a cross here if you didn't tell your GP or nurse ☐

3. Change in bowel habit

Did you have this symptom?

Yes ☐ **Please complete A then B or C below**

No ☐ **Please go to Question 4**

A When did you first notice this? ☐☐ / ☐☐ / ☐☐ **OR** ☐

B When did you first tell your GP or nurse? ☐☐ / ☐☐ / ☐☐ **OR** ☐

OR

C Put a cross here if you didn't tell your GP or nurse ☐

4. Bleeding from back passage

Did you have this symptom?

Yes ☐ **Please complete A then B or C below**

No ☐ **Please go to Section 2**

A When did you first notice this? ☐☐ / ☐☐ / ☐☐ **OR** ☐

B When did you first tell your GP or nurse? ☐☐ / ☐☐ / ☐☐ **OR** ☐

OR

C Put a cross here if you didn't tell your GP or nurse ☐

Section 2

Please give an exact date if you can. Otherwise give your best estimate (for example approximately how long ago, the month or the season). You may wish to refer to your diary or calendar if you have it with you.

Did you have any of the following?

1. Decrease in appetite

Did you have this?

Yes ☐ **Please complete A then B or C below**

No ☐ **Please go to Question 2**

A When did you first notice this? ☐☐ / ☐☐ / ☐☐ **OR** ☐

B When did you first tell your GP or nurse? ☐☐ / ☐☐ / ☐☐ **OR** ☐

OR

C Put a cross here if you didn't tell your GP or nurse ☐

2. Unexplained weight loss

Did you have this?

Yes ☐ **Please complete A then B or C below**

No ☐ **Please go to Question 3**

A When did you first notice this? ☐☐ / ☐☐ / ☐☐ **OR** ☐

B When did you first tell your GP or nurse? ☐☐ / ☐☐ / ☐☐ **OR** ☐

OR

C Put a cross here if you didn't tell your GP or nurse ☐

3. **Fatigue or tiredness that is unusual for you**

Did you have this?

Yes ☐ **Please complete A then B or C below**

No ☐ **Please go to Question 4**

A When did you first notice this? ☐☐ / ☐☐ / ☐☐ **OR** ☐

B When did you first tell your GP or nurse? ☐☐ / ☐☐ / ☐☐ **OR** ☐

OR

C Put a cross here if you didn't tell your GP or nurse ☐

4. **Feeling different 'in yourself' from usual**

Did you have this?

Yes ☐ **Please complete A then B or C below**

No ☐ **Please go to Question 5**

A When did you first notice this? ☐☐ / ☐☐ / ☐☐ **OR** ☐

B When did you first tell your GP or nurse? ☐☐ / ☐☐ / ☐☐ **OR** ☐

OR

C Put a cross here if you didn't tell your GP or nurse ☐

Please try and describe what the feeling was

If you had any further symptoms which you feel are relevant to your cancer diagnosis, please list them below. If not, please go to Question 7.

Please describe the symptom here and complete A then B or C below

5.

**A**	When did you first notice this?	☐☐ / ☐☐ / ☐☐ **OR** ☐
**B**	When did you first tell your GP or nurse?	☐☐ / ☐☐ / ☐☐ **OR** ☐
	**OR**	
**C**	Put a cross here if you didn't tell your GP or nurse	☐

If you had another symptom you feel is relevant, please write it below, otherwise go to Question 7.

Please describe the symptom here and complete A then B or C below

6.

**A**	When did you first notice this?	☐☐ / ☐☐ / ☐☐ **OR** ☐
**B**	When did you first tell your GP or nurse?	☐☐ / ☐☐ / ☐☐ **OR** ☐
	**OR**	
**C**	Put a cross here if you didn't tell your GP or nurse	☐

If you had further symptoms you would like to tell us about, there is space at the end of the Questionnaire for you to write them.

Did your GP send you for any tests?

Thinking back to the time immediately before you diagnosis, did your GP send you for any of the following tests?

Please cross the appropriate boxes

7. Were you sent for any of these?

	Yes	Not sure	No
Blood test(s)	☐	☐	☐
CT Scan	☐	☐	☐
Ultrasound scan	☐	☐	☐
Barium Enema	☐	☐	☐
Sigmoidoscopy or colonoscopy (Looking at bowel with internal camera)	☐	☐	☐
X-ray	☐	☐	☐

Please go to Question 8

About you

The next questions relate to general information about you. It would help us to know more about the background of the people completing the questionnaire.

8. Which best describes your employment status?

Please cross one box only

- Employed full-time ☐
- Employed part-time ☐
- Self employed full-time ☐
- Self employed part-time ☐
- Unemployed (seeking work) ☐
- Unemployed (not seeking work) ☐
- Retired ☐
- Student ☐
- Permanently sick/disabled ☐
- Temporarily sick/disabled ☐
- Looking after family/home ☐

Other, please describe ________

9. What is your highest level of qualification?

Please cross one box only

- Degree (or equivalent) ☐
- Diploma (or equivalent) ☐
- 'A' level ☐
- GCSE / 'O' level ☐
- None ☐

Other, please specify ________

10. **How would you describe your ethnicity**

Please cross one box only

White ☐
or
- White British ☐
- White Irish ☐
- Other White background ☐

Mixed ☐
or
- White & Black Carribean ☐
- White & Black African ☐
- White & Asian ☐
- Other mixed background ☐

Black or Black British ☐
or
- Carribean ☐
- African ☐
- Other Black background ☐

Asian or Asian British ☐
or
- Indian ☐
- Pakistani ☐
- Bangladeshi ☐
- Other Asian background ☐

Chinese or other ethnic group ☐
or
- Chinese ☐
- Other ethnic group ☐

11.

Do you live alone?

Yes ☐ **Please go to Question 12**

No ☐ **Please say who you live with below**

Who do you live with?
(e.g. wife, husband, partner, family member)

12. Are you suffering from, or have you suffered from, any of the following in the past 2 years?

Please cross any that are applicable to you

- Asthma ☐
- Chronic Obstructive Pulmonary Disease (COPD) ☐
- Other lung disease (e.g. fibrosis, bronchiectatis etc) ☐
- Heart disease ☐
- Anxiety or depression ☐
- Inflammatory bowel disease ☐
- Irritable bowel syndrome ☐
- Peptic ulcer ☐
- Previous cancer ☐
- Diabetes ☐
- Arthritis ☐

13. About smoking

Please cross the appropriate statement

- Are you a current smoker? ☐
- Are you an ex-smoker? ☐
- Are you a non-smoker (never smoked)? ☐

14. Do you think you were more at risk of getting cancer because of your family history?

Yes ☐

No ☐

15. **The following questions are about how completing the questionnaire made you feel.**

A number of statements which people have used to describe themselves are given below. Read each statement and then cross the most appropriate box to indicate how you feel right now, at the moment. There are no right or wrong answers. Do not spend too much time on any one statement but give the answer which seems to describe your present feeling best.

		Not at all	Somewhat	Moderately	Very much
1.	I feel calm	☐	☐	☐	☐
2.	I am tense	☐	☐	☐	☐
3.	I feel upset	☐	☐	☐	☐
4.	I am relaxed	☐	☐	☐	☐
5.	I feel content	☐	☐	☐	☐
6.	I am worried	☐	☐	☐	☐

Please make sure you have answered all the questions.

If you have any other comments or there is anything else you would like to tell us about when you first noticed your symptoms, please do so using the space below.

If you feel you would like to talk to someone about this questionnaire at a later stage, please speak to the doctor or nurse who is looking after you, or be in touch with the research team, whose contact details are on your patient information sheet

Thank you for completing the questionnaire

Please give it to the research nurse

The next page is for the Researcher to complete

Researcher - Post-completion Sheet

Researcher initials: ☐☐☐

Date questionnaire completed: Date ☐☐ / ☐☐ / ☐☐ Time ☐☐:☐☐

If not completed, give reason ☐

How long is it since the patient was told of his/her diagnosis? ☐

How long did it take the patient to complete the questionnaire?

- Less than 5 minutes ☐
- Between 5 and 10 minutes ☐
- Between 11 and 15 minutes ☐
- Longer than 15 minutes ☐

For the patient selected to <u>complete the questionnaire on their own</u>

Did the patient ask for any help? ☐ Yes ☐ No

If yes,

please specify what help was requested

____

____

please specify what help was given

____

____

For the patient selected for <u>the researcher-administered questionnaire</u>

Did the patient find any of the questions difficult? ☐ Yes ☐ No

If yes,

please specify which questions and what the difficulty was

____

____

____

____

Was the patient made anxious? ☐ Yes ☐ No

If yes,

please give details

____

____

____

____

Where did the patient complete the questionnaire?
(Please specify whether this was in a separate room alone, in a corner of the clinic, the ward or other

____

Thank you. Please now send the questionnaire and patient information sheet and consent form back to Cardiff University in the freepost envelope provided.

57727

PID - 50

Ymddiriedolaeth GIG Gogledd Orllewin Cymru
North West Wales NHS Trust

NHS WALES GIG CYMRU
Ymddiriedolaeth GIG Gogledd Cymru
North Wales NHS Trust

☐ GLAN CLWYD

CID - ☐ GWYNEDD

☐ MAELOR

Confidential Questionnaire

Looking At Your Symptoms Study

This Study has been funded by Cancer Research UK

Cardiff University / Prifysgol Caerdydd
Ysgol Glinigol Gogledd Cymru / North Wales Clinical School
Cancer Research UK
sewtu South East Wales Trials Unit / Uned Ymchwil De-ddwyrain Cymru

Dear Patient

I am very grateful to you for agreeing to help us with this study.

I hope the information you supply will help us give people a diagnosis earlier.

If you feel you would like to talk to someone about this questionnaire at a later stage, please speak to the doctor or nurse who is looking after you, or be in touch with the research team, whose contact details are on your patient information sheet.

I can assure you that anything you tell us will be treated in the utmost confidence.

Yours sincerely

Dr Richard Neal
Senior Clinical Lecturer
Cardiff University
Chief Investigator, Symptoms Study

About your symptoms

These questions are about symptoms you noticed before your diagnosis. We are interested in symptoms which you think are related to your cancer.

Please put a cross in the appropriate box, or write an answer as required.

Looking back...

1. Did you have any symptoms that you feel were due to your cancer before you were diagnosed?

Yes ☐ **Please continue with Question 2**

No ☐ **Please go to Section 2 on Page 5**

2. What was the first symptom that made you think something might be wrong?

☐

Please go to Question 3

The following questions are about when you first noticed a symptom and when you first told your GP or nurse about it.

Please give an exact date if you can. Otherwise give your best estimate. (For example approximately how long ago, the month or season). You wish to refer to your diary or calendar if you have it with you.

Here is an example question:

Decrease in appetite

Did you have this symptom?

Yes ☒ **Please complete A then B or C below**

No ☐ **Please go to Section 2**

A When did you first notice this? d d / m m / y y **OR** Estimate '3 months ago' or 'June'

B When did you first tell your GP or nurse? 2 3 / 1 2 / 0 7 **OR** Estimate

OR

C Put a cross here if you didn't tell your GP or nurse ☐

3. Change in bowel habit

Did you have this symptom?

Yes ☐ **Please complete A then B or C below**
No ☐ **Please go to Question 4**

A When did you <u>first notice</u> this? ☐☐ / ☐☐ / ☐☐ **OR** ☐

B When did you <u>first tell</u> your GP or nurse? ☐☐ / ☐☐ / ☐☐ **OR** ☐

OR

C Put a cross here if you <u>didn't tell</u> your GP or nurse ☐

4. Bleeding from back passage

Did you have this symptom?

Yes ☐ **Please complete A then B or C below**
No ☐ **Please go to Section 2**

A When did you <u>first notice</u> this? ☐☐ / ☐☐ / ☐☐ **OR** ☐

B When did you <u>first tell</u> your GP or nurse? ☐☐ / ☐☐ / ☐☐ **OR** ☐

OR

C Put a cross here if you <u>didn't tell</u> your GP or nurse ☐

Section 2

Please give an exact date if you can. Otherwise give your best estimate (for example approximately how long ago, the month or the season). You may wish to refer to your diary or calendar if you have it with you.

Did you have any of the following?

1. Decrease in appetite

Did you have this?

Yes ☐ **Please complete A then B or C below**

No ☐ **Please go to Question 2**

A When did you first notice this? ☐☐ / ☐☐ / ☐☐ **OR** ☐

B When did you first tell your GP or nurse? ☐☐ / ☐☐ / ☐☐ **OR** ☐

OR

C Put a cross here if you didn't tell your GP or nurse ☐

2. Unexplained weight loss

Did you have this?

Yes ☐ **Please complete A then B or C below**

No ☐ **Please go to Question 3**

A When did you first notice this? ☐☐ / ☐☐ / ☐☐ **OR** ☐

B When did you first tell your GP or nurse? ☐☐ / ☐☐ / ☐☐ **OR** ☐

OR

C Put a cross here if you didn't tell your GP or nurse ☐

3. **Fatigue or tiredness that is unusual for you**

Did you have this?

Yes ☐ **Please complete A then B or C below**

No ☐ **Please go to Question 4**

A When did you first notice this? ☐☐ / ☐☐ / ☐☐ **OR** ☐

B When did you first tell your GP or nurse? ☐☐ / ☐☐ / ☐☐ **OR** ☐

OR

C Put a cross here if you didn't tell your GP or nurse ☐

4. **Feeling different 'in yourself' from usual**

Did you have this?

Yes ☐ **Please complete A then B or C below**

No ☐ **Please go to Question 5**

A When did you first notice this? ☐☐ / ☐☐ / ☐☐ **OR** ☐

B When did you first tell your GP or nurse? ☐☐ / ☐☐ / ☐☐ **OR** ☐

OR

C Put a cross here if you didn't tell your GP or nurse ☐

Please try and describe what the feeling was

If you had any further symptoms which you feel are relevant to your cancer diagnosis, please list them below. If not, please go to Question 7.

Please describe the symptom here and complete A then B or C below

5.

**A**	When did you first notice this?	__ __ / __ __ / __ __ **OR**
**B**	When did you first tell your GP or nurse?	__ __ / __ __ / __ __ **OR**
	**OR**	
**C**	Put a cross here if you didn't tell your GP or nurse	☐

If you had another symptom you feel is relevant, please write it below, otherwise go to Question 7.

Please describe the symptom here and complete A then B or C below

6.

**A**	When did you first notice this?	__ __ / __ __ / __ __ **OR**
**B**	When did you first tell your GP or nurse?	__ __ / __ __ / __ __ **OR**
	**OR**	
**C**	Put a cross here if you didn't tell your GP or nurse	☐

If you had further symptoms you would like to tell us about, there is space at the end of the Questionnaire for you to write them.

Did your GP send you for any tests?

Thinking back to the time immediately before you diagnosis, did your GP send you for any of the following tests?

Please cross the appropriate boxes

7. Were you sent for any of these?

	Yes	Not sure	No
Blood test(s)	☐	☐	☐
CT Scan	☐	☐	☐
Ultrasound scan	☐	☐	☐
Barium Enema	☐	☐	☐
Sigmoidoscopy or colonoscopy (Looking at bowel with internal camera)	☐	☐	☐
X-ray	☐	☐	☐

Please go to Question 8

About you

The next questions relate to general information about you. It would help us to know more about the background of the people completing the questionnaire.

8. **Which best describes your employment status?**

Please cross one box only

- Employed full-time ☐
- Employed part-time ☐
- Self employed full-time ☐
- Self employed part-time ☐
- Unemployed (seeking work) ☐
- Unemployed (not seeking work) ☐
- Retired ☐
- Student ☐
- Permanently sick/disabled ☐
- Temporarily sick/disabled ☐
- Looking after family/home ☐

Other, please describe

9. **What is your highest level of qualification?**

Please cross one box only

- Degree (or equivalent) ☐
- Diploma (or equivalent) ☐
- 'A' level ☐
- GCSE / 'O' level ☐
- None ☐

Other, please specify

10. How would you describe your ethnicity

Please cross one box only

White ☐
or
- White British ☐
- White Irish ☐
- Other White background ☐

Mixed ☐
or
- White & Black Carribean ☐
- White & Black African ☐
- White & Asian ☐
- Other mixed background ☐

Black or Black British ☐
or
- Carribean ☐
- African ☐
- Other Black background ☐

Asian or Asian British ☐
or
- Indian ☐
- Pakistani ☐
- Bangladeshi ☐
- Other Asian background ☐

Chinese or other ethnic group ☐
or
- Chinese ☐
- Other ethnic group ☐

11.

Do you live alone?

Yes ☐ **Please go to Question 12**

No ☐ **Please say who you live with below**

Who do you live with?
(e.g. wife, husband, partner, family member)

☐

12. Are you suffering from, or have you suffered from, any of the following in the past 2 years?

Please cross any that are applicable to you

- Asthma ☐
- Chronic Obstructive Pulmonary Disease (COPD) ☐
- Other lung disease (e.g. fibrosis, bronchiectatis etc) ☐
- Heart disease ☐
- Anxiety or depression ☐
- Inflammatory bowel disease ☐
- Irritable bowel syndrome ☐
- Peptic ulcer ☐
- Previous cancer ☐
- Diabetes ☐
- Arthritis ☐

13. About smoking

Please cross the appropriate statement

- Are you a current smoker? ☐
- Are you an ex-smoker? ☐
- Are you a non-smoker (never smoked)? ☐

14. Do you think you were more at risk of getting cancer because of your family history?

Yes ☐

No ☐

15. The following questions are about how completing the questionnaire made you feel.

A number of statements which people have used to describe themselves are given below. Read each statement and then cross the most appropriate box to indicate how you feel right now, at the moment. There are no right or wrong answers. Do not spend too much time on any one statement but give the answer which seems to describe your present feeling best.

		Not at all	Somewhat	Moderately	Very much
1.	I feel calm	☐	☐	☐	☐
2.	I am tense	☐	☐	☐	☐
3.	I feel upset	☐	☐	☐	☐
4.	I am relaxed	☐	☐	☐	☐
5.	I feel content	☐	☐	☐	☐
6.	I am worried	☐	☐	☐	☐

Please make sure you have answered all the questions.

If you have any other comments or there is anything else you would like to tell us about when you first noticed your symptoms, please do so using the space below.

If you feel you would like to talk to someone about this questionnaire at a later stage, please speak to the doctor or nurse who is looking after you, or be in touch with the research team, whose contact details are on your patient information sheet

Thank you for completing the questionnaire

Please give it to the research nurse

The next page is for the Researcher to complete

Researcher - Post-completion Sheet **Researcher initials:** ☐☐☐

Date questionnaire completed: Date ☐☐ / ☐☐ / ☐☐ Time ☐☐:☐☐

If not completed, give reason ☐

How long is it since the patient was told of his/her diagnosis? ☐

How long did it take the patient to complete the questionnaire?

- Less than 5 minutes ☐
- Between 5 and 10 minutes ☐
- Between 11 and 15 minutes ☐
- Longer than 15 minutes ☐

For the patient selected to complete the questionnaire on their own

Did the patient ask for any help? ☐ Yes ☐ No

If yes,

please specify what help was requested

____

____

please specify what help was given

____

____

For the patient selected for the researcher-administered questionnaire

Did the patient find any of the questions difficult? ☐ Yes ☐ No

If yes,

please specify which questions and what the difficulty was

____

____

____

____

Was the patient made anxious? ☐ Yes ☐ No

If yes,

please give details

____

____

____

____

Where did the patient complete the questionnaire?
(Please specify whether this was in a separate room alone, in a corner of the clinic, the ward or other

____

Thank you. Please now send the questionnaire and patient information sheet and consent form back to Cardiff University in the freepost envelope provided.

57727

PID - | | 5 | 1 |

Ymddiriedolaeth GIG Gogledd Orllewin Cymru
North West Wales NHS Trust

NHS WALES GIG CYMRU
Ymddiriedolaeth GIG Gogledd Cymru
North Wales NHS Trust

CID - ☐ GLAN CLWYD
☐ GWYNEDD
☐ MAELOR

Confidential Questionnaire

Looking At Your Symptoms Study

This Study has been funded by Cancer Research UK

Cardiff University / Prifysgol Caerdydd
Ysgol Glinigol Gogledd Cymru / North Wales Clinical School
Cancer Research UK
sewtu — South East Wales Trials Unit / Uned Ymchwil De-ddwyrain Cymru

Dear Patient

I am very grateful to you for agreeing to help us with this study.

I hope the information you supply will help us give people a diagnosis earlier.

If you feel you would like to talk to someone about this questionnaire at a later stage, please speak to the doctor or nurse who is looking after you, or be in touch with the research team, whose contact details are on your patient information sheet.

I can assure you that anything you tell us will be treated in the utmost confidence.

Yours sincerely

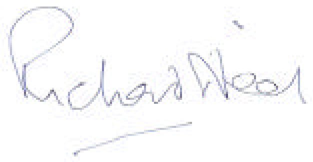

Dr Richard Neal
Senior Clinical Lecturer
Cardiff University
Chief Investigator, Symptoms Study

About your symptoms

These questions are about symptoms you noticed before your diagnosis. We are interested in symptoms which you think are related to your cancer.

Please put a cross in the appropriate box, or write an answer as required.

Looking back...

1. Did you have any symptoms that you feel were due to your cancer before you were diagnosed?

Yes ☐ **Please continue with Question 2**

No ☐ **Please go to Section 2 on Page 5**

2. What was the first symptom that made you think something might be wrong?

☐

Please go to Question 3

The following questions are about when you first noticed a symptom and when you first told your GP or nurse about it.

Please give an exact date if you can. Otherwise give your best estimate. (For example approximately how long ago, the month or season). You wish to refer to your diary or calendar if you have it with you.

Here is an example question:

Decrease in appetite

Did you have this symptom?

Yes ☒ **Please complete A then B or C below**

No ☐ **Please go to Section 2**

A When did you first notice this? d d / m m / y y **OR** Estimate: '3 months ago' or 'June'

B When did you first tell your GP or nurse? 23 / 12 / 07 **OR** Estimate

OR

C Put a cross here if you didn't tell your GP or nurse ☐

3. Change in bowel habit

Did you have this symptom?

Yes ☐ **Please complete A then B or C below**

No ☐ **Please go to Question 4**

A When did you first notice this? ☐☐ / ☐☐ / ☐☐ **OR** ☐

B When did you first tell your GP or nurse? ☐☐ / ☐☐ / ☐☐ **OR** ☐

OR

C Put a cross here if you didn't tell your GP or nurse ☐

4. Bleeding from back passage

Did you have this symptom?

Yes ☐ **Please complete A then B or C below**

No ☐ **Please go to Section 2**

A When did you first notice this? ☐☐ / ☐☐ / ☐☐ **OR** ☐

B When did you first tell your GP or nurse? ☐☐ / ☐☐ / ☐☐ **OR** ☐

OR

C Put a cross here if you didn't tell your GP or nurse ☐

Section 2

Please give an exact date if you can. Otherwise give your best estimate (for example approximately how long ago, the month or the season). You may wish to refer to your diary or calendar if you have it with you.

Did you have any of the following?

1. Decrease in appetite

Did you have this?

Yes ☐ **Please complete A then B or C below**

No ☐ **Please go to Question 2**

A When did you first notice this? ☐☐ / ☐☐ / ☐☐ **OR** ☐

B When did you first tell your GP or nurse? ☐☐ / ☐☐ / ☐☐ **OR** ☐

OR

C Put a cross here if you didn't tell your GP or nurse ☐

2. Unexplained weight loss

Did you have this?

Yes ☐ **Please complete A then B or C below**

No ☐ **Please go to Question 3**

A When did you first notice this? ☐☐ / ☐☐ / ☐☐ **OR** ☐

B When did you first tell your GP or nurse? ☐☐ / ☐☐ / ☐☐ **OR** ☐

OR

C Put a cross here if you didn't tell your GP or nurse ☐

3. Fatigue or tiredness that is unusual for you

Did you have this?

Yes ☐ **Please complete A then B or C below**

No ☐ **Please go to Question 4**

A When did you <u>first notice</u> this? ☐☐ / ☐☐ / ☐☐ **OR** ☐

B When did you <u>first tell</u> your GP or nurse? ☐☐ / ☐☐ / ☐☐ **OR** ☐

OR

C Put a cross here if you <u>didn't tell</u> your GP or nurse ☐

4. Feeling different 'in yourself' from usual

Did you have this?

Yes ☐ **Please complete A then B or C below**

No ☐ **Please go to Question 5**

A When did you <u>first notice</u> this? ☐☐ / ☐☐ / ☐☐ **OR** ☐

B When did you <u>first tell</u> your GP or nurse? ☐☐ / ☐☐ / ☐☐ **OR** ☐

OR

C Put a cross here if you <u>didn't tell</u> your GP or nurse ☐

Please try and describe what the feeling was

If you had any further symptoms which you feel are relevant to your cancer diagnosis, please list them below. If not, please go to Question 7.

Please describe the symptom here and complete A then B or C below

5.

**A**	When did you first notice this?	__ __ / __ __ / __ __ **OR**
**B**	When did you first tell your GP or nurse?	__ __ / __ __ / __ __ **OR**
	**OR**	
**C**	Put a cross here if you didn't tell your GP or nurse	☐

If you had another symptom you feel is relevant, please write it below, otherwise go to Question 7.

Please describe the symptom here and complete A then B or C below

6.

**A**	When did you first notice this?	__ __ / __ __ / __ __ **OR**
**B**	When did you first tell your GP or nurse?	__ __ / __ __ / __ __ **OR**
	**OR**	
**C**	Put a cross here if you didn't tell your GP or nurse	☐

If you had further symptoms you would like to tell us about, there is space at the end of the Questionnaire for you to write them.

Did your GP send you for any tests?

Thinking back to the time immediately before you diagnosis, did your GP send you for any of the following tests?

Please cross the appropriate boxes

7. Were you sent for any of these?

	Yes	Not sure	No
Blood test(s)	☐	☐	☐
CT Scan	☐	☐	☐
Ultrasound scan	☐	☐	☐
Barium Enema	☐	☐	☐
Sigmoidoscopy or colonoscopy (Looking at bowel with internal camera)	☐	☐	☐
X-ray	☐	☐	☐

Please go to Question 8

About you

The next questions relate to general information about you. It would help us to know more about the background of the people completing the questionnaire.

8. Which best describes your employment status?

Please cross one box only

- Employed full-time ☐
- Employed part-time ☐
- Self employed full-time ☐
- Self employed part-time ☐
- Unemployed (seeking work) ☐
- Unemployed (not seeking work) ☐
- Retired ☐
- Student ☐
- Permanently sick/disabled ☐
- Temporarily sick/disabled ☐
- Looking after family/home ☐

Other, please describe ☐

9. What is your highest level of qualification?

Please cross one box only

- Degree (or equivalent) ☐
- Diploma (or equivalent) ☐
- 'A' level ☐
- GCSE / 'O' level ☐
- None ☐

Other, please specify ☐

10. **How would you describe your ethnicity**

Please cross one box only

White ☐
or
- White British ☐
- White Irish ☐
- Other White background ☐

Mixed ☐
or
- White & Black Carribean ☐
- White & Black African ☐
- White & Asian ☐
- Other mixed background ☐

Black or Black British ☐
or
- Carribean ☐
- African ☐
- Other Black background ☐

Asian or Asian British ☐
or
- Indian ☐
- Pakistani ☐
- Bangladeshi ☐
- Other Asian background ☐

Chinese or other ethnic group ☐
or
- Chinese ☐
- Other ethnic group ☐

11.

Do you live alone?

Yes ☐ **Please go to Question 12**

No ☐ **Please say who you live with below**

Who do you live with?
(e.g. wife, husband, partner, family member)

12. **Are you suffering from, or have you suffered from, any of the following in the past 2 years?**

Please cross any that are applicable to you

- Asthma ☐
- Chronic Obstructive Pulmonary Disease (COPD) ☐
- Other lung disease (e.g. fibrosis, bronchiectatis etc) ☐
- Heart disease ☐
- Anxiety or depression ☐
- Inflammatory bowel disease ☐
- Irritable bowel syndrome ☐
- Peptic ulcer ☐
- Previous cancer ☐
- Diabetes ☐
- Arthritis ☐

13. **About smoking**

Please cross the appropriate statement

- Are you a current smoker? ☐
- Are you an ex-smoker? ☐
- Are you a non-smoker (never smoked)? ☐

14. **Do you think you were more at risk of getting cancer because of your family history?**

Yes ☐

No ☐

15. **The following questions are about how completing the questionnaire made you feel.**

A number of statements which people have used to describe themselves are given below. Read each statement and then cross the most appropriate box to indicate how you feel <u>right now, at the moment.</u> There are no right or wrong answers. Do not spend too much time on any one statement but give the answer which seems to describe your present feeling best.

		Not at all	Somewhat	Moderately	Very much
1.	I feel calm	☐	☐	☐	☐
2.	I am tense	☐	☐	☐	☐
3.	I feel upset	☐	☐	☐	☐
4.	I am relaxed	☐	☐	☐	☐
5.	I feel content	☐	☐	☐	☐
6.	I am worried	☐	☐	☐	☐

Please make sure you have answered all the questions.

If you have any other comments or there is anything else you would like to tell us about when you first noticed your symptoms, please do so using the space below.

If you feel you would like to talk to someone about this questionnaire at a later stage, please speak to the doctor or nurse who is looking after you, or be in touch with the research team, whose contact details are on your patient information sheet

Thank you for completing the questionnaire

Please give it to the research nurse

The next page is for the Researcher to complete

57727

5 1

Researcher - Post-completion Sheet

Researcher initials: ☐☐☐

Date questionnaire completed: Date ☐☐ / ☐☐ / ☐☐ Time ☐☐:☐☐

If not completed, give reason ☐

How long is it since the patient was told of his/her diagnosis? ☐

How long did it take the patient to complete the questionnaire?

- Less than 5 minutes ☐
- Between 5 and 10 minutes ☐
- Between 11 and 15 minutes ☐
- Longer than 15 minutes ☐

For the patient selected to complete the questionnaire on their own

Did the patient ask for any help? ☐ Yes ☐ No

If yes,

please specify what help was requested

____

____

please specify what help was given

____

____

For the patient selected for the researcher-administered questionnaire

Did the patient find any of the questions difficult? ☐ Yes ☐ No

If yes,

please specify which questions and what the difficulty was

____

____

____

____

Was the patient made anxious? ☐ Yes ☐ No

If yes,

please give details

____

____

____

____

Where did the patient complete the questionnaire?

(Please specify whether this was in a separate room alone, in a corner of the clinic, the ward or other

____

Thank you. Please now send the questionnaire and patient information sheet and consent form back to Cardiff University in the freepost envelope provided.

57727

PID - | | 5 | 2

Ymddiriedolaeth GIG Gogledd Orllewin Cymru
North West Wales NHS Trust

NHS WALES GIG CYMRU
Ymddiriedolaeth GIG Gogledd Cymru
North Wales NHS Trust

☐ GLAN CLWYD

CID - ☐ GWYNEDD

☐ MAELOR

Confidential Questionnaire

Looking At Your Symptoms Study

This Study has been funded by Cancer Research UK

Cardiff University / Prifysgol Caerdydd
Ysgol Glinigol Gogledd Cymru / North Wales Clinical School
Cancer Research UK
sewtu South East Wales Trials Unit / Uned Ymchwil De-ddwyrain Cymru

Dear Patient

I am very grateful to you for agreeing to help us with this study.

I hope the information you supply will help us give people a diagnosis earlier.

If you feel you would like to talk to someone about this questionnaire at a later stage, please speak to the doctor or nurse who is looking after you, or be in touch with the research team, whose contact details are on your patient information sheet.

I can assure you that anything you tell us will be treated in the utmost confidence.

Yours sincerely

Dr Richard Neal
Senior Clinical Lecturer
Cardiff University
Chief Investigator, Symptoms Study

About your symptoms

These questions are about symptoms you noticed before your diagnosis. We are interested in symptoms which you think are related to your cancer.

Please put a cross in the appropriate box, or write an answer as required.

Looking back...

1. Did you have any symptoms that you feel were due to your cancer before you were diagnosed?

Yes ☐ **Please continue with Question 2**

No ☐ **Please go to Section 2 on Page 5**

2. What was the first symptom that made you think something might be wrong?

[ ]

Please go to Question 3

The following questions are about when you first noticed a symptom and when you first told your GP or nurse about it.

Please give an exact date if you can. Otherwise give your best estimate. (For example approximately how long ago, the month or season). You wish to refer to your diary or calendar if you have it with you.

Here is an example question:

Decrease in appetite

Did you have this symptom?

Yes ☒ **Please complete A then B or C below**

No ☐ **Please go to Section 2**

A When did you first notice this? d d / m m / y y **OR** Estimate '3 months ago' or 'June'

B When did you first tell your GP or nurse? 2 3 / 1 2 / 0 7 **OR** Estimate

OR

C Put a cross here if you didn't tell your GP or nurse ☐

3. Change in bowel habit

Did you have this symptom?

Yes ☐ **Please complete A then B or C below**

No ☐ **Please go to Question 4**

A When did you first notice this? ☐☐ / ☐☐ / ☐☐ **OR** ☐

B When did you first tell your GP or nurse? ☐☐ / ☐☐ / ☐☐ **OR** ☐

OR

C Put a cross here if you didn't tell your GP or nurse ☐

4. Bleeding from back passage

Did you have this symptom?

Yes ☐ **Please complete A then B or C below**

No ☐ **Please go to Section 2**

A When did you first notice this? ☐☐ / ☐☐ / ☐☐ **OR** ☐

B When did you first tell your GP or nurse? ☐☐ / ☐☐ / ☐☐ **OR** ☐

OR

C Put a cross here if you didn't tell your GP or nurse ☐

Section 2

Please give an exact date if you can. Otherwise give your best estimate (for example approximately how long ago, the month or the season). You may wish to refer to your diary or calendar if you have it with you.

Did you have any of the following?

1. Decrease in appetite

Did you have this?

Yes ☐ **Please complete A then B or C below**

No ☐ **Please go to Question 2**

A When did you first notice this? ☐☐ / ☐☐ / ☐☐ **OR** ☐

B When did you first tell your GP or nurse? ☐☐ / ☐☐ / ☐☐ **OR** ☐

OR

C Put a cross here if you didn't tell your GP or nurse ☐

2. Unexplained weight loss

Did you have this?

Yes ☐ **Please complete A then B or C below**

No ☐ **Please go to Question 3**

A When did you first notice this? ☐☐ / ☐☐ / ☐☐ **OR** ☐

B When did you first tell your GP or nurse? ☐☐ / ☐☐ / ☐☐ **OR** ☐

OR

C Put a cross here if you didn't tell your GP or nurse ☐

3. **Fatigue or tiredness that is unusual for you**

Did you have this?

Yes ☐ **Please complete A then B or C below**

No ☐ **Please go to Question 4**

A When did you first notice this? ☐☐ / ☐☐ / ☐☐ **OR** ☐

B When did you first tell your GP or nurse? ☐☐ / ☐☐ / ☐☐ **OR** ☐

OR

C Put a cross here if you didn't tell your GP or nurse ☐

4. **Feeling different 'in yourself' from usual**

Did you have this?

Yes ☐ **Please complete A then B or C below**

No ☐ **Please go to Question 5**

A When did you first notice this? ☐☐ / ☐☐ / ☐☐ **OR** ☐

B When did you first tell your GP or nurse? ☐☐ / ☐☐ / ☐☐ **OR** ☐

OR

C Put a cross here if you didn't tell your GP or nurse ☐

Please try and describe what the feeling was

If you had any further symptoms which you feel are relevant to your cancer diagnosis, please list them below. If not, please go to Question 7.

Please describe the symptom here and complete A then B or C below

5.

A When did you first notice this? ☐☐ / ☐☐ / ☐☐ **OR** ☐

B When did you first tell your GP or nurse? ☐☐ / ☐☐ / ☐☐ **OR** ☐

OR

C Put a cross here if you didn't tell your GP or nurse ☐

If you had another symptom you feel is relevant, please write it below, otherwise go to Question 7.

Please describe the symptom here and complete A then B or C below

6.

A When did you first notice this? ☐☐ / ☐☐ / ☐☐ **OR** ☐

B When did you first tell your GP or nurse? ☐☐ / ☐☐ / ☐☐ **OR** ☐

OR

C Put a cross here if you didn't tell your GP or nurse ☐

If you had further symptoms you would like to tell us about, there is space at the end of the Questionnaire for you to write them.

Did your GP send you for any tests?

Thinking back to the time immediately before you diagnosis, did your GP send you for any of the following tests?

Please cross the appropriate boxes

7. Were you sent for any of these?

	Yes	Not sure	No
Blood test(s)	☐	☐	☐
CT Scan	☐	☐	☐
Ultrasound scan	☐	☐	☐
Barium Enema	☐	☐	☐
Sigmoidoscopy or colonoscopy (Looking at bowel with internal camera)	☐	☐	☐
X-ray	☐	☐	☐

Please go to Question 8

About you

The next questions relate to general information about you. It would help us to know more about the background of the people completing the questionnaire.

8. Which best describes your employment status?

Please cross one box only

- Employed full-time ☐
- Employed part-time ☐
- Self employed full-time ☐
- Self employed part-time ☐
- Unemployed (seeking work) ☐
- Unemployed (not seeking work) ☐
- Retired ☐
- Student ☐
- Permanently sick/disabled ☐
- Temporarily sick/disabled ☐
- Looking after family/home ☐
- Other, please describe ________

9. What is your highest level of qualification?

Please cross one box only

- Degree (or equivalent) ☐
- Diploma (or equivalent) ☐
- 'A' level ☐
- GCSE / 'O' level ☐
- None ☐
- Other, please specify ________

10. **How would you describe your ethnicity**

Please cross one box only

White ☐
or
White British ☐
White Irish ☐
Other White background ☐

Mixed ☐
or
White & Black Carribean ☐
White & Black African ☐
White & Asian ☐
Other mixed background ☐

Black or Black British ☐
or
Carribean ☐
African ☐
Other Black background ☐

Asian or Asian British ☐
or
Indian ☐
Pakistani ☐
Bangladeshi ☐
Other Asian background ☐

Chinese or other ethnic group ☐
or
Chinese ☐
Other ethnic group ☐

11.

Do you live alone?

Yes ☐ **Please go to Question 12**

No ☐ **Please say who you live with below**

Who do you live with?
(e.g. wife, husband, partner, family member)

12. **Are you suffering from, or have you suffered from, any of the following in the past 2 years?**

Please cross any that are applicable to you

- Asthma ☐
- Chronic Obstructive Pulmonary Disease (COPD) ☐
- Other lung disease (e.g. fibrosis, bronchiectatis etc) ☐
- Heart disease ☐
- Anxiety or depression ☐
- Inflammatory bowel disease ☐
- Irritable bowel syndrome ☐
- Peptic ulcer ☐
- Previous cancer ☐
- Diabetes ☐
- Arthritis ☐

13. **About smoking**

Please cross the appropriate statement

- Are you a current smoker? ☐
- Are you an ex-smoker? ☐
- Are you a non-smoker (never smoked)? ☐

14. **Do you think you were more at risk of getting cancer because of your family history?**

Yes ☐
No ☐

15. The following questions are about how completing the questionnaire made you feel.

A number of statements which people have used to describe themselves are given below. Read each statement and then cross the most appropriate box to indicate how you feel right now, at the moment. There are no right or wrong answers. Do not spend too much time on any one statement but give the answer which seems to describe your present feeling best.

		Not at all	Somewhat	Moderately	Very much
1.	I feel calm	☐	☐	☐	☐
2.	I am tense	☐	☐	☐	☐
3.	I feel upset	☐	☐	☐	☐
4.	I am relaxed	☐	☐	☐	☐
5.	I feel content	☐	☐	☐	☐
6.	I am worried	☐	☐	☐	☐

Please make sure you have answered all the questions.

If you have any other comments or there is anything else you would like to tell us about when you first noticed your symptoms, please do so using the space below.

If you feel you would like to talk to someone about this questionnaire at a later stage, please speak to the doctor or nurse who is looking after you, or be in touch with the research team, whose contact details are on your patient information sheet

Thank you for completing the questionnaire

Please give it to the research nurse

The next page is for the Researcher to complete

57727

52

Researcher - Post-completion Sheet

Researcher initials: ☐☐☐

Date questionnaire completed: Date ☐☐ / ☐☐ / ☐☐ Time ☐☐:☐☐

If not completed, give reason ☐

How long is it since the patient was told of his/her diagnosis? ☐

How long did it take the patient to complete the questionnaire?

- Less than 5 minutes ☐
- Between 5 and 10 minutes ☐
- Between 11 and 15 minutes ☐
- Longer than 15 minutes ☐

For the patient selected to <u>complete the questionnaire on their own</u>

Did the patient ask for any help? ☐ Yes ☐ No

If yes,

please specify what help was requested

____

____

please specify what help was given

____

____

For the patient selected for <u>the researcher-administered questionnaire</u>

Did the patient find any of the questions difficult? ☐ Yes ☐ No

If yes,

please specify which questions and what the difficulty was

____

____

____

____

Was the patient made anxious? ☐ Yes ☐ No

If yes,

please give details

____

____

____

____

Where did the patient complete the questionnaire?
(Please specify whether this was in a separate room alone, in a corner of the clinic, the ward or other

____

Thank you. Please now send the questionnaire and patient information sheet and consent form back to Cardiff University in the freepost envelope provided.

57727

PID - 53

GLAN CLWYD

CID - GWYNEDD

MAELOR

Confidential Questionnaire

Looking At Your Symptoms Study

This Study has been funded by Cancer Research UK

Dear Patient

I am very grateful to you for agreeing to help us with this study.

I hope the information you supply will help us give people a diagnosis earlier.

If you feel you would like to talk to someone about this questionnaire at a later stage, please speak to the doctor or nurse who is looking after you, or be in touch with the research team, whose contact details are on your patient information sheet.

I can assure you that anything you tell us will be treated in the utmost confidence.

Yours sincerely

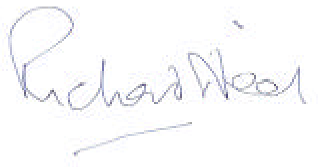

Dr Richard Neal
Senior Clinical Lecturer
Cardiff University
Chief Investigator, Symptoms Study

About your symptoms

These questions are about symptoms you noticed before your diagnosis. We are interested in symptoms which you think are related to your cancer.

Please put a cross in the appropriate box, or write an answer as required.

Looking back...

1. Did you have any symptoms that you feel were due to your cancer before you were diagnosed?

Yes ☐ **Please continue with Question 2**

No ☐ **Please go to Section 2 on Page 5**

2. What was the first symptom that made you think something might be wrong?

☐

Please go to Question 3

The following questions are about when you first noticed a symptom and when you first told your GP or nurse about it.

Please give an exact date if you can. Otherwise give your best estimate. (For example approximately how long ago, the month or season). You wish to refer to your diary or calendar if you have it with you.

Here is an example question:

Decrease in appetite

Did you have this symptom?

Yes ☒ **Please complete A then B or C below**

No ☐ **Please go to Section 2**

A When did you first notice this? d d / m m / y y **OR** Estimate '3 months ago' or 'June'

B When did you first tell your GP or nurse? 2 3 / 1 2 / 0 7 **OR** Estimate

OR

C Put a cross here if you didn't tell your GP or nurse ☐

3. Change in bowel habit

Did you have this symptom?

Yes ☐ **Please complete A then B or C below**

No ☐ **Please go to Question 4**

A When did you first notice this? ☐☐ / ☐☐ / ☐☐ **OR** ☐

B When did you first tell your GP or nurse? ☐☐ / ☐☐ / ☐☐ **OR** ☐

OR

C Put a cross here if you didn't tell your GP or nurse ☐

4. Bleeding from back passage

Did you have this symptom?

Yes ☐ **Please complete A then B or C below**

No ☐ **Please go to Section 2**

A When did you first notice this? ☐☐ / ☐☐ / ☐☐ **OR** ☐

B When did you first tell your GP or nurse? ☐☐ / ☐☐ / ☐☐ **OR** ☐

OR

C Put a cross here if you didn't tell your GP or nurse ☐

Section 2

Please give an exact date if you can. Otherwise give your best estimate (for example approximately how long ago, the month or the season). You may wish to refer to your diary or calendar if you have it with you.

Did you have any of the following?

1. Decrease in appetite

Did you have this?

Yes ☐ **Please complete A then B or C below**

No ☐ **Please go to Question 2**

A When did you first notice this? ☐☐ / ☐☐ / ☐☐ **OR** ☐

B When did you first tell your GP or nurse? ☐☐ / ☐☐ / ☐☐ **OR** ☐

OR

C Put a cross here if you didn't tell your GP or nurse ☐

2. Unexplained weight loss

Did you have this?

Yes ☐ **Please complete A then B or C below**

No ☐ **Please go to Question 3**

A When did you first notice this? ☐☐ / ☐☐ / ☐☐ **OR** ☐

B When did you first tell your GP or nurse? ☐☐ / ☐☐ / ☐☐ **OR** ☐

OR

C Put a cross here if you didn't tell your GP or nurse ☐

3. **Fatigue or tiredness that is unusual for you**

Did you have this?

Yes ☐ **Please complete A then B or C below**

No ☐ **Please go to Question 4**

A When did you <u>first notice</u> this? ☐☐ / ☐☐ / ☐☐ **OR** ☐

B When did you <u>first tell</u> your GP or nurse? ☐☐ / ☐☐ / ☐☐ **OR** ☐

OR

C Put a cross here if you <u>didn't tell</u> your GP or nurse ☐

4. **Feeling different 'in yourself' from usual**

Did you have this?

Yes ☐ **Please complete A then B or C below**

No ☐ **Please go to Question 5**

A When did you <u>first notice</u> this? ☐☐ / ☐☐ / ☐☐ **OR** ☐

B When did you <u>first tell</u> your GP or nurse? ☐☐ / ☐☐ / ☐☐ **OR** ☐

OR

C Put a cross here if you <u>didn't tell</u> your GP or nurse ☐

Please try and describe what the feeling was

If you had any further symptoms which you feel are relevant to your cancer diagnosis, please list them below. If not, please go to Question 7.

Please describe the symptom here and complete A then B or C below

5.

**A**	When did you first notice this?	__ __ / __ __ / __ __ **OR**
**B**	When did you first tell your GP or nurse?	__ __ / __ __ / __ __ **OR**
	**OR**	
**C**	Put a cross here if you didn't tell your GP or nurse	☐

If you had another symptom you feel is relevant, please write it below, otherwise go to Question 7.

Please describe the symptom here and complete A then B or C below

6.

**A**	When did you first notice this?	__ __ / __ __ / __ __ **OR**
**B**	When did you first tell your GP or nurse?	__ __ / __ __ / __ __ **OR**
	**OR**	
**C**	Put a cross here if you didn't tell your GP or nurse	☐

If you had further symptoms you would like to tell us about, there is space at the end of the Questionnaire for you to write them.

Did your GP send you for any tests?

Thinking back to the time immediately before you diagnosis, did your GP send you for any of the following tests?

Please cross the appropriate boxes

7. Were you sent for any of these?

	Yes	Not sure	No
Blood test(s)	☐	☐	☐
CT Scan	☐	☐	☐
Ultrasound scan	☐	☐	☐
Barium Enema	☐	☐	☐
Sigmoidoscopy or colonoscopy (Looking at bowel with internal camera)	☐	☐	☐
X-ray	☐	☐	☐

Please go to Question 8

About you

The next questions relate to general information about you. It would help us to know more about the background of the people completing the questionnaire.

8. Which best describes your employment status?

Please cross one box only

- Employed full-time ☐
- Employed part-time ☐
- Self employed full-time ☐
- Self employed part-time ☐
- Unemployed (seeking work) ☐
- Unemployed (not seeking work) ☐
- Retired ☐
- Student ☐
- Permanently sick/disabled ☐
- Temporarily sick/disabled ☐
- Looking after family/home ☐
- Other, please describe ________

9. What is your highest level of qualification?

Please cross one box only

- Degree (or equivalent) ☐
- Diploma (or equivalent) ☐
- 'A' level ☐
- GCSE / 'O' level ☐
- None ☐
- Other, please specify ________

10. How would you describe your ethnicity

Please cross one box only

White ☐
or
- White British ☐
- White Irish ☐
- Other White background ☐

Mixed ☐
or
- White & Black Carribean ☐
- White & Black African ☐
- White & Asian ☐
- Other mixed background ☐

Black or Black British ☐
or
- Carribean ☐
- African ☐
- Other Black background ☐

Asian or Asian British ☐
or
- Indian ☐
- Pakistani ☐
- Bangladeshi ☐
- Other Asian background ☐

Chinese or other ethnic group ☐
or
- Chinese ☐
- Other ethnic group ☐

11.

Do you live alone?

Yes ☐ **Please go to Question 12**

No ☐ **Please say who you live with below**

Who do you live with?
(e.g. wife, husband, partner, family member)

12. Are you suffering from, or have you suffered from, any of the following in the past 2 years?

Please cross any that are applicable to you

- Asthma ☐
- Chronic Obstructive Pulmonary Disease (COPD) ☐
- Other lung disease (e.g. fibrosis, bronchiectatis etc) ☐
- Heart disease ☐
- Anxiety or depression ☐
- Inflammatory bowel disease ☐
- Irritable bowel syndrome ☐
- Peptic ulcer ☐
- Previous cancer ☐
- Diabetes ☐
- Arthritis ☐

13. About smoking

Please cross the appropriate statement

- Are you a current smoker? ☐
- Are you an ex-smoker? ☐
- Are you a non-smoker (never smoked)? ☐

14. Do you think you were more at risk of getting cancer because of your family history?

Yes ☐

No ☐

15. **The following questions are about how completing the questionnaire made you feel.**

A number of statements which people have used to describe themselves are given below. Read each statement and then cross the most appropriate box to indicate how you feel right now, at the moment. There are no right or wrong answers. Do not spend too much time on any one statement but give the answer which seems to describe your present feeling best.

		Not at all	Somewhat	Moderately	Very much
1.	I feel calm	☐	☐	☐	☐
2.	I am tense	☐	☐	☐	☐
3.	I feel upset	☐	☐	☐	☐
4.	I am relaxed	☐	☐	☐	☐
5.	I feel content	☐	☐	☐	☐
6.	I am worried	☐	☐	☐	☐

Please make sure you have answered all the questions.

If you have any other comments or there is anything else you would like to tell us about when you first noticed your symptoms, please do so using the space below.

If you feel you would like to talk to someone about this questionnaire at a later stage, please speak to the doctor or nurse who is looking after you, or be in touch with the research team, whose contact details are on your patient information sheet

Thank you for completing the questionnaire

Please give it to the research nurse

The next page is for the Researcher to complete

57727

5 3

Researcher - Post-completion Sheet

Researcher initials: ☐☐☐

Date questionnaire completed: Date ☐☐ / ☐☐ / ☐☐ Time ☐☐:☐☐

If not completed, give reason ☐

How long is it since the patient was told of his/her diagnosis? ☐

How long did it take the patient to complete the questionnaire?

- Less than 5 minutes ☐
- Between 5 and 10 minutes ☐
- Between 11 and 15 minutes ☐
- Longer than 15 minutes ☐

For the patient selected to complete the questionnaire on their own

Did the patient ask for any help? ☐ Yes ☐ No

If yes,

please specify what help was requested

____

____

please specify what help was given

____

____

For the patient selected for the researcher-administered questionnaire

Did the patient find any of the questions difficult? ☐ Yes ☐ No

If yes,

please specify which questions and what the difficulty was

____

____

____

____

Was the patient made anxious? ☐ Yes ☐ No

If yes,

please give details

____

____

____

____

Where did the patient complete the questionnaire?

(Please specify whether this was in a separate room alone, in a corner of the clinic, the ward or other

____

Thank you. Please now send the questionnaire and patient information sheet and consent form back to Cardiff University in the freepost envelope provided.

57727

PID - | | 5 | 4 |

Ymddiriedolaeth GIG Gogledd Orllewin Cymru
North West Wales NHS Trust

NHS WALES GIG CYMRU
Ymddiriedolaeth GIG Gogledd Cymru
North Wales NHS Trust

☐ GLAN CLWYD

CID - ☐ GWYNEDD

☐ MAELOR

Confidential Questionnaire

Looking At Your Symptoms Study

This Study has been funded by Cancer Research UK

Cardiff University / Prifysgol Caerdydd

Ysgol Glinigol Gogledd Cymru / North Wales Clinical School

Cancer Research UK

sewtu — South East Wales Trials Unit / Uned Ymchwil De-ddwyrain Cymru

Dear Patient

I am very grateful to you for agreeing to help us with this study.

I hope the information you supply will help us give people a diagnosis earlier.

If you feel you would like to talk to someone about this questionnaire at a later stage, please speak to the doctor or nurse who is looking after you, or be in touch with the research team, whose contact details are on your patient information sheet.

I can assure you that anything you tell us will be treated in the utmost confidence.

Yours sincerely

Dr Richard Neal
Senior Clinical Lecturer
Cardiff University
Chief Investigator, Symptoms Study

About your symptoms

These questions are about symptoms you noticed before your diagnosis. We are interested in symptoms which you think are related to your cancer.

Please put a cross in the appropriate box, or write an answer as required.

Looking back...

1. Did you have any symptoms that you feel were due to your cancer before you were diagnosed?

Yes ☐ **Please continue with Question 2**

No ☐ **Please go to Section 2 on Page 5**

2. What was the first symptom that made you think something might be wrong?

[ ]

Please go to Question 3

The following questions are about when you first noticed a symptom and when you first told your GP or nurse about it.

Please give an exact date if you can. Otherwise give your best estimate. (For example approximately how long ago, the month or season). You wish to refer to your diary or calendar if you have it with you.

Here is an example question:

Decrease in appetite

Did you have this symptom?

Yes ☒ **Please complete A then B or C below**

No ☐ **Please go to Section 2**

A When did you first notice this? d d / m m / y y **OR** Estimate: '3 months ago' or 'June'

B When did you first tell your GP or nurse? 2 3 / 1 2 / 0 7 **OR** Estimate

OR

C Put a cross here if you didn't tell your GP or nurse ☐

3. Change in bowel habit

Did you have this symptom?

Yes ☐ **Please complete A then B or C below**

No ☐ **Please go to Question 4**

A When did you first notice this? ☐☐ / ☐☐ / ☐☐ **OR** ☐

B When did you first tell your GP or nurse? ☐☐ / ☐☐ / ☐☐ **OR** ☐

OR

C Put a cross here if you didn't tell your GP or nurse ☐

4. Bleeding from back passage

Did you have this symptom?

Yes ☐ **Please complete A then B or C below**

No ☐ **Please go to Section 2**

A When did you first notice this? ☐☐ / ☐☐ / ☐☐ **OR** ☐

B When did you first tell your GP or nurse? ☐☐ / ☐☐ / ☐☐ **OR** ☐

OR

C Put a cross here if you didn't tell your GP or nurse ☐

Section 2

Please give an exact date if you can. Otherwise give your best estimate (for example approximately how long ago, the month or the season). You may wish to refer to your diary or calendar if you have it with you.

Did you have any of the following?

1. Decrease in appetite

Did you have this?

Yes ☐ **Please complete A then B or C below**

No ☐ **Please go to Question 2**

A When did you first notice this? ☐☐ / ☐☐ / ☐☐ **OR** ☐

B When did you first tell your GP or nurse? ☐☐ / ☐☐ / ☐☐ **OR** ☐

OR

C Put a cross here if you didn't tell your GP or nurse ☐

2. Unexplained weight loss

Did you have this?

Yes ☐ **Please complete A then B or C below**

No ☐ **Please go to Question 3**

A When did you first notice this? ☐☐ / ☐☐ / ☐☐ **OR** ☐

B When did you first tell your GP or nurse? ☐☐ / ☐☐ / ☐☐ **OR** ☐

OR

C Put a cross here if you didn't tell your GP or nurse ☐

3. Fatigue or tiredness that is unusual for you

Did you have this?

Yes ☐ Please complete A then B or C below

No ☐ Please go to Question 4

A When did you <u>first notice</u> this? ☐☐ / ☐☐ / ☐☐ **OR** ☐

B When did you <u>first tell</u> your GP or nurse? ☐☐ / ☐☐ / ☐☐ **OR** ☐

OR

C Put a cross here if you <u>didn't tell</u> your GP or nurse ☐

4. Feeling different 'in yourself' from usual

Did you have this?

Yes ☐ Please complete A then B or C below

No ☐ Please go to Question 5

A When did you <u>first notice</u> this? ☐☐ / ☐☐ / ☐☐ **OR** ☐

B When did you <u>first tell</u> your GP or nurse? ☐☐ / ☐☐ / ☐☐ **OR** ☐

OR

C Put a cross here if you <u>didn't tell</u> your GP or nurse ☐

Please try and describe what the feeling was

If you had any further symptoms which you feel are relevant to your cancer diagnosis, please list them below. If not, please go to Question 7.

Please describe the symptom here and complete A then B or C below

5.

**A**	When did you first notice this?	☐☐ / ☐☐ / ☐☐ **OR** ☐
**B**	When did you first tell your GP or nurse?	☐☐ / ☐☐ / ☐☐ **OR** ☐
	**OR**	
**C**	Put a cross here if you didn't tell your GP or nurse	☐

If you had another symptom you feel is relevant, please write it below, otherwise go to Question 7.

Please describe the symptom here and complete A then B or C below

6.

**A**	When did you first notice this?	☐☐ / ☐☐ / ☐☐ **OR** ☐
**B**	When did you first tell your GP or nurse?	☐☐ / ☐☐ / ☐☐ **OR** ☐
	**OR**	
**C**	Put a cross here if you didn't tell your GP or nurse	☐

If you had further symptoms you would like to tell us about, there is space at the end of the Questionnaire for you to write them.

Did your GP send you for any tests?

Thinking back to the time immediately before you diagnosis, did your GP send you for any of the following tests?

Please cross the appropriate boxes

7. Were you sent for any of these?

	Yes	Not sure	No
Blood test(s)	☐	☐	☐
CT Scan	☐	☐	☐
Ultrasound scan	☐	☐	☐
Barium Enema	☐	☐	☐
Sigmoidoscopy or colonoscopy (Looking at bowel with internal camera)	☐	☐	☐
X-ray	☐	☐	☐

Please go to Question 8

About you

The next questions relate to general information about you. It would help us to know more about the background of the people completing the questionnaire.

8. Which best describes your employment status?

Please cross one box only

- Employed full-time ☐
- Employed part-time ☐
- Self employed full-time ☐
- Self employed part-time ☐
- Unemployed (seeking work) ☐
- Unemployed (not seeking work) ☐
- Retired ☐
- Student ☐
- Permanently sick/disabled ☐
- Temporarily sick/disabled ☐
- Looking after family/home ☐
- Other, please describe ______

9. What is your highest level of qualification?

Please cross one box only

- Degree (or equivalent) ☐
- Diploma (or equivalent) ☐
- 'A' level ☐
- GCSE / 'O' level ☐
- None ☐
- Other, please specify ______

10. How would you describe your ethnicity

Please cross one box only

White ☐
or
- White British ☐
- White Irish ☐
- Other White background ☐

Mixed ☐
or
- White & Black Carribean ☐
- White & Black African ☐
- White & Asian ☐
- Other mixed background ☐

Black or Black British ☐
or
- Carribean ☐
- African ☐
- Other Black background ☐

Asian or Asian British ☐
or
- Indian ☐
- Pakistani ☐
- Bangladeshi ☐
- Other Asian background ☐

Chinese or other ethnic group ☐
or
- Chinese ☐
- Other ethnic group ☐

11.

Do you live alone?

Yes ☐ **Please go to Question 12**

No ☐ **Please say who you live with below**

Who do you live with?
(e.g. wife, husband, partner, family member) ☐

12. **Are you suffering from, or have you suffered from, any of the following in the past 2 years?**

Please cross any that are applicable to you

- Asthma ☐
- Chronic Obstructive Pulmonary Disease (COPD) ☐
- Other lung disease (e.g. fibrosis, bronchiectatis etc) ☐
- Heart disease ☐
- Anxiety or depression ☐
- Inflammatory bowel disease ☐
- Irritable bowel syndrome ☐
- Peptic ulcer ☐
- Previous cancer ☐
- Diabetes ☐
- Arthritis ☐

13. **About smoking**

Please cross the appropriate statement

- Are you a current smoker? ☐
- Are you an ex-smoker? ☐
- Are you a non-smoker (never smoked)? ☐

14. **Do you think you were more at risk of getting cancer because of your family history?**

Yes ☐
No ☐

15. **The following questions are about how completing the questionnaire made you feel.**

A number of statements which people have used to describe themselves are given below. Read each statement and then cross the most appropriate box to indicate how you feel right now, at the moment. There are no right or wrong answers. Do not spend too much time on any one statement but give the answer which seems to describe your present feeling best.

		Not at all	Somewhat	Moderately	Very much
1.	I feel calm	☐	☐	☐	☐
2.	I am tense	☐	☐	☐	☐
3.	I feel upset	☐	☐	☐	☐
4.	I am relaxed	☐	☐	☐	☐
5.	I feel content	☐	☐	☐	☐
6.	I am worried	☐	☐	☐	☐

Please make sure you have answered all the questions.

If you have any other comments or there is anything else you would like to tell us about when you first noticed your symptoms, please do so using the space below.

If you feel you would like to talk to someone about this questionnaire at a later stage, please speak to the doctor or nurse who is looking after you, or be in touch with the research team, whose contact details are on your patient information sheet

Thank you for completing the questionnaire

Please give it to the research nurse

The next page is for the Researcher to complete

57727

Researcher - Post-completion Sheet

Researcher initials: ☐☐☐

Date questionnaire completed: Date ☐☐ / ☐☐ / ☐☐ Time ☐☐:☐☐

If not completed, give reason ☐

How long is it since the patient was told of his/her diagnosis? ☐

How long did it take the patient to complete the questionnaire?

- Less than 5 minutes ☐
- Between 5 and 10 minutes ☐
- Between 11 and 15 minutes ☐
- Longer than 15 minutes ☐

For the patient selected to complete the questionnaire on their own

Did the patient ask for any help? ☐ Yes ☐ No

If yes,

please specify what help was requested

____

____

please specify what help was given

____

____

For the patient selected for the researcher-administered questionnaire

Did the patient find any of the questions difficult? ☐ Yes ☐ No

If yes,

please specify which questions and what the difficulty was

____

____

____

____

Was the patient made anxious? ☐ Yes ☐ No

If yes,

please give details

____

____

____

____

Where did the patient complete the questionnaire?

(Please specify whether this was in a separate room alone, in a corner of the clinic, the ward or other

____

Thank you. Please now send the questionnaire and patient information sheet and consent form back to Cardiff University in the freepost envelope provided.

57727

PID - | | 5 | 5 |

☐ GLAN CLWYD

CID - ☐ GWYNEDD

☐ MAELOR

Confidential Questionnaire

Looking At Your Symptoms Study

This Study has been funded by Cancer Research UK

Dear Patient

I am very grateful to you for agreeing to help us with this study.

I hope the information you supply will help us give people a diagnosis earlier.

If you feel you would like to talk to someone about this questionnaire at a later stage, please speak to the doctor or nurse who is looking after you, or be in touch with the research team, whose contact details are on your patient information sheet.

I can assure you that anything you tell us will be treated in the utmost confidence.

Yours sincerely

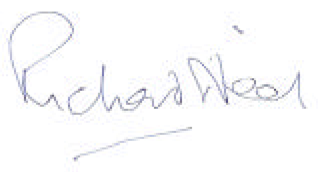

Dr Richard Neal
Senior Clinical Lecturer
Cardiff University
Chief Investigator, Symptoms Study

About your symptoms

These questions are about symptoms you noticed before your diagnosis. We are interested in symptoms which you think are related to your cancer.

Please put a cross in the appropriate box, or write an answer as required.

Looking back...

1. Did you have any symptoms that you feel were due to your cancer before you were diagnosed?

Yes ☐ **Please continue with Question 2**

No ☐ **Please go to Section 2 on Page 5**

2. What was the first symptom that made you think something might be wrong?

☐

Please go to Question 3

The following questions are about when you first noticed a symptom and when you first told your GP or nurse about it.

Please give an exact date if you can. Otherwise give your best estimate. (For example approximately how long ago, the month or season). You wish to refer to your diary or calendar if you have it with you.

Here is an example question:

Decrease in appetite

Did you have this symptom?

Yes ☒ **Please complete A then B or C below**

No ☐ **Please go to Section 2**

A When did you first notice this? d d / m m / y y **OR** Estimate '3 months ago' or 'June'

B When did you first tell your GP or nurse? 2 3 / 1 2 / 0 7 **OR** Estimate

OR

C Put a cross here if you didn't tell your GP or nurse ☐

3. Change in bowel habit

Did you have this symptom?

Yes ☐ **Please complete A then B or C below**

No ☐ **Please go to Question 4**

A When did you first notice this? ☐☐ / ☐☐ / ☐☐ **OR** ☐

B When did you first tell your GP or nurse? ☐☐ / ☐☐ / ☐☐ **OR** ☐

OR

C Put a cross here if you didn't tell your GP or nurse ☐

4. Bleeding from back passage

Did you have this symptom?

Yes ☐ **Please complete A then B or C below**

No ☐ **Please go to Section 2**

A When did you first notice this? ☐☐ / ☐☐ / ☐☐ **OR** ☐

B When did you first tell your GP or nurse? ☐☐ / ☐☐ / ☐☐ **OR** ☐

OR

C Put a cross here if you didn't tell your GP or nurse ☐

Section 2

Please give an exact date if you can. Otherwise give your best estimate (for example approximately how long ago, the month or the season). You may wish to refer to your diary or calendar if you have it with you.

Did you have any of the following?

1. Decrease in appetite

Did you have this?

Yes ☐ **Please complete A then B or C below**

No ☐ **Please go to Question 2**

A When did you first notice this? ☐☐ / ☐☐ / ☐☐ **OR** ☐

B When did you first tell your GP or nurse? ☐☐ / ☐☐ / ☐☐ **OR** ☐

OR

C Put a cross here if you didn't tell your GP or nurse ☐

2. Unexplained weight loss

Did you have this?

Yes ☐ **Please complete A then B or C below**

No ☐ **Please go to Question 3**

A When did you first notice this? ☐☐ / ☐☐ / ☐☐ **OR** ☐

B When did you first tell your GP or nurse? ☐☐ / ☐☐ / ☐☐ **OR** ☐

OR

C Put a cross here if you didn't tell your GP or nurse ☐

3. **Fatigue or tiredness that is unusual for you**

Did you have this?

Yes ☐ **Please complete A then B or C below**

No ☐ **Please go to Question 4**

A When did you first notice this? ☐☐ / ☐☐ / ☐☐ **OR** ☐

B When did you first tell your GP or nurse? ☐☐ / ☐☐ / ☐☐ **OR** ☐

OR

C Put a cross here if you didn't tell your GP or nurse ☐

4. **Feeling different 'in yourself' from usual**

Did you have this?

Yes ☐ **Please complete A then B or C below**

No ☐ **Please go to Question 5**

A When did you first notice this? ☐☐ / ☐☐ / ☐☐ **OR** ☐

B When did you first tell your GP or nurse? ☐☐ / ☐☐ / ☐☐ **OR** ☐

OR

C Put a cross here if you didn't tell your GP or nurse ☐

Please try and describe what the feeling was

If you had any further symptoms which you feel are relevant to your cancer diagnosis, please list them below. If not, please go to Question 7.

Please describe the symptom here and complete A then B or C below

5.

**A**	When did you first notice this?	☐☐ / ☐☐ / ☐☐ **OR** ☐
**B**	When did you first tell your GP or nurse?	☐☐ / ☐☐ / ☐☐ **OR** ☐
	**OR**	
**C**	Put a cross here if you didn't tell your GP or nurse	☐

If you had another symptom you feel is relevant, please write it below, otherwise go to Question 7.

Please describe the symptom here and complete A then B or C below

6.

**A**	When did you first notice this?	☐☐ / ☐☐ / ☐☐ **OR** ☐
**B**	When did you first tell your GP or nurse?	☐☐ / ☐☐ / ☐☐ **OR** ☐
	**OR**	
**C**	Put a cross here if you didn't tell your GP or nurse	☐

If you had further symptoms you would like to tell us about, there is space at the end of the Questionnaire for you to write them.

Did your GP send you for any tests?

Thinking back to the time immediately before you diagnosis, did your GP send you for any of the following tests?

Please cross the appropriate boxes

7. Were you sent for any of these?

	Yes	Not sure	No
Blood test(s)	☐	☐	☐
CT Scan	☐	☐	☐
Ultrasound scan	☐	☐	☐
Barium Enema	☐	☐	☐
Sigmoidoscopy or colonoscopy (Looking at bowel with internal camera)	☐	☐	☐
X-ray	☐	☐	☐

Please go to Question 8

About you

The next questions relate to general information about you. It would help us to know more about the background of the people completing the questionnaire.

8. Which best describes your employment status?

Please cross one box only

- Employed full-time ☐
- Employed part-time ☐
- Self employed full-time ☐
- Self employed part-time ☐
- Unemployed (seeking work) ☐
- Unemployed (not seeking work) ☐
- Retired ☐
- Student ☐
- Permanently sick/disabled ☐
- Temporarily sick/disabled ☐
- Looking after family/home ☐

Other, please describe

9. What is your highest level of qualification?

Please cross one box only

- Degree (or equivalent) ☐
- Diploma (or equivalent) ☐
- 'A' level ☐
- GCSE / 'O' level ☐
- None ☐

Other, please specify

10. How would you describe your ethnicity

Please cross one box only

White ☐
or
- White British ☐
- White Irish ☐
- Other White background ☐

Mixed ☐
or
- White & Black Carribean ☐
- White & Black African ☐
- White & Asian ☐
- Other mixed background ☐

Black or Black British ☐
or
- Carribean ☐
- African ☐
- Other Black background ☐

Asian or Asian British ☐
or
- Indian ☐
- Pakistani ☐
- Bangladeshi ☐
- Other Asian background ☐

Chinese or other ethnic group ☐
or
- Chinese ☐
- Other ethnic group ☐

11.

Do you live alone?

Yes ☐ **Please go to Question 12**

No ☐ **Please say who you live with below**

Who do you live with?
(e.g. wife, husband, partner, family member)

12. Are you suffering from, or have you suffered from, any of the following in the past 2 years?

Please cross any that are applicable to you

- Asthma ☐
- Chronic Obstructive Pulmonary Disease (COPD) ☐
- Other lung disease (e.g. fibrosis, bronchiectatis etc) ☐
- Heart disease ☐
- Anxiety or depression ☐
- Inflammatory bowel disease ☐
- Irritable bowel syndrome ☐
- Peptic ulcer ☐
- Previous cancer ☐
- Diabetes ☐
- Arthritis ☐

13. About smoking

Please cross the appropriate statement

- Are you a current smoker? ☐
- Are you an ex-smoker? ☐
- Are you a non-smoker (never smoked)? ☐

14. Do you think you were more at risk of getting cancer because of your family history?

Yes ☐

No ☐

15. The following questions are about how completing the questionnaire made you feel.

A number of statements which people have used to describe themselves are given below. Read each statement and then cross the most appropriate box to indicate how you feel right now, at the moment. There are no right or wrong answers. Do not spend too much time on any one statement but give the answer which seems to describe your present feeling best.

		Not at all	Somewhat	Moderately	Very much
1.	I feel calm	☐	☐	☐	☐
2.	I am tense	☐	☐	☐	☐
3.	I feel upset	☐	☐	☐	☐
4.	I am relaxed	☐	☐	☐	☐
5.	I feel content	☐	☐	☐	☐
6.	I am worried	☐	☐	☐	☐

Please make sure you have answered all the questions.

If you have any other comments or there is anything else you would like to tell us about when you first noticed your symptoms, please do so using the space below.

If you feel you would like to talk to someone about this questionnaire at a later stage, please speak to the doctor or nurse who is looking after you, or be in touch with the research team, whose contact details are on your patient information sheet

Thank you for completing the questionnaire

Please give it to the research nurse

The next page is for the Researcher to complete

Researcher - Post-completion Sheet

Researcher initials: ☐☐☐

Date questionnaire completed: Date ☐☐ / ☐☐ / ☐☐ Time ☐☐:☐☐

If not completed, give reason ☐

How long is it since the patient was told of his/her diagnosis? ☐

How long did it take the patient to complete the questionnaire?

- Less than 5 minutes ☐
- Between 5 and 10 minutes ☐
- Between 11 and 15 minutes ☐
- Longer than 15 minutes ☐

For the patient selected to complete the questionnaire on their own

Did the patient ask for any help? ☐ Yes ☐ No

If yes,

please specify what help was requested

___

___

please specify what help was given

___

___

For the patient selected for the researcher-administered questionnaire

Did the patient find any of the questions difficult? ☐ Yes ☐ No

If yes,

please specify which questions and what the difficulty was

___

___

___

___

Was the patient made anxious? ☐ Yes ☐ No

If yes,

please give details

___

___

___

___

Where did the patient complete the questionnaire?

(Please specify whether this was in a separate room alone, in a corner of the clinic, the ward or other

___

Thank you. Please now send the questionnaire and patient information sheet and consent form back to Cardiff University in the freepost envelope provided.

57727

PID - | | 5 | 6 |

Ymddiriedolaeth GIG Gogledd Orllewin Cymru
North West Wales NHS Trust

NHS WALES GIG CYMRU
Ymddiriedolaeth GIG Gogledd Cymru
North Wales NHS Trust

☐ GLAN CLWYD

CID - ☐ GWYNEDD

☐ MAELOR

Confidential Questionnaire

Looking At Your Symptoms Study

This Study has been funded by Cancer Research UK

CARDIFF UNIVERSITY PRIFYSGOL CAERDYDD

Ysgol Glinigol Gogledd Cymru North Wales Clinical School

CANCER RESEARCH UK

sewtu South East Wales Trials Unit Uned Ymchwil De-ddwyrain Cymru

Dear Patient

I am very grateful to you for agreeing to help us with this study.

I hope the information you supply will help us give people a diagnosis earlier.

If you feel you would like to talk to someone about this questionnaire at a later stage, please speak to the doctor or nurse who is looking after you, or be in touch with the research team, whose contact details are on your patient information sheet.

I can assure you that anything you tell us will be treated in the utmost confidence.

Yours sincerely

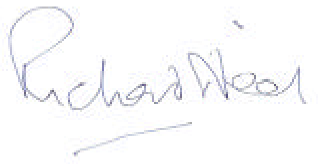

Dr Richard Neal
Senior Clinical Lecturer
Cardiff University
Chief Investigator, Symptoms Study

About your symptoms

These questions are about symptoms you noticed before your diagnosis. We are interested in symptoms which you think are related to your cancer.

Please put a cross in the appropriate box, or write an answer as required.

Looking back...

1. Did you have any symptoms that you feel were due to your cancer before you were diagnosed?

Yes ☐ **Please continue with Question 2**

No ☐ **Please go to Section 2 on Page 5**

2. What was the first symptom that made you think something might be wrong?

☐

Please go to Question 3

The following questions are about when you first noticed a symptom and when you first told your GP or nurse about it.

Please give an exact date if you can. Otherwise give your best estimate. (For example approximately how long ago, the month or season). You wish to refer to your diary or calendar if you have it with you.

Here is an example question:

Decrease in appetite

Did you have this symptom?

Yes ☒ **Please complete A then B or C below**

No ☐ **Please go to Section 2**

A When did you first notice this? d d / m m / y y **OR** Estimate '3 months ago' or 'June'

B When did you first tell your GP or nurse? 2 3 / 1 2 / 0 7 **OR** Estimate

OR

C Put a cross here if you didn't tell your GP or nurse ☐

3. Change in bowel habit

Did you have this symptom?

Yes ☐ **Please complete A then B or C below**

No ☐ **Please go to Question 4**

A When did you first notice this? ☐☐ / ☐☐ / ☐☐ **OR** ☐

B When did you first tell your GP or nurse? ☐☐ / ☐☐ / ☐☐ **OR** ☐

OR

C Put a cross here if you didn't tell your GP or nurse ☐

4. Bleeding from back passage

Did you have this symptom?

Yes ☐ **Please complete A then B or C below**

No ☐ **Please go to Section 2**

A When did you first notice this? ☐☐ / ☐☐ / ☐☐ **OR** ☐

B When did you first tell your GP or nurse? ☐☐ / ☐☐ / ☐☐ **OR** ☐

OR

C Put a cross here if you didn't tell your GP or nurse ☐

Section 2

Please give an exact date if you can. Otherwise give your best estimate (for example approximately how long ago, the month or the season). You may wish to refer to your diary or calendar if you have it with you.

Did you have any of the following?

1. Decrease in appetite

Did you have this?

Yes ☐ **Please complete A then B or C below**

No ☐ **Please go to Question 2**

A When did you first notice this? ☐☐ / ☐☐ / ☐☐ **OR** ☐

B When did you first tell your GP or nurse? ☐☐ / ☐☐ / ☐☐ **OR** ☐

OR

C Put a cross here if you didn't tell your GP or nurse ☐

2. Unexplained weight loss

Did you have this?

Yes ☐ **Please complete A then B or C below**

No ☐ **Please go to Question 3**

A When did you first notice this? ☐☐ / ☐☐ / ☐☐ **OR** ☐

B When did you first tell your GP or nurse? ☐☐ / ☐☐ / ☐☐ **OR** ☐

OR

C Put a cross here if you didn't tell your GP or nurse ☐

3. **Fatigue or tiredness that is unusual for you**

Did you have this?

Yes ☐ **Please complete A then B or C below**

No ☐ **Please go to Question 4**

A When did you first notice this? ☐☐ / ☐☐ / ☐☐ **OR** ☐

B When did you first tell your GP or nurse? ☐☐ / ☐☐ / ☐☐ **OR** ☐

OR

C Put a cross here if you didn't tell your GP or nurse ☐

4. **Feeling different 'in yourself' from usual**

Did you have this?

Yes ☐ **Please complete A then B or C below**

No ☐ **Please go to Question 5**

A When did you first notice this? ☐☐ / ☐☐ / ☐☐ **OR** ☐

B When did you first tell your GP or nurse? ☐☐ / ☐☐ / ☐☐ **OR** ☐

OR

C Put a cross here if you didn't tell your GP or nurse ☐

Please try and describe what the feeling was

If you had any further symptoms which you feel are relevant to your cancer diagnosis, please list them below. If not, please go to Question 7.

Please describe the symptom here and complete A then B or C below

5.

**A**	When did you first notice this?	☐☐ / ☐☐ / ☐☐ **OR** ☐
**B**	When did you first tell your GP or nurse?	☐☐ / ☐☐ / ☐☐ **OR** ☐
	**OR**	
**C**	Put a cross here if you didn't tell your GP or nurse	☐

If you had another symptom you feel is relevant, please write it below, otherwise go to Question 7.

Please describe the symptom here and complete A then B or C below

6.

**A**	When did you first notice this?	☐☐ / ☐☐ / ☐☐ **OR** ☐
**B**	When did you first tell your GP or nurse?	☐☐ / ☐☐ / ☐☐ **OR** ☐
	**OR**	
**C**	Put a cross here if you didn't tell your GP or nurse	☐

If you had further symptoms you would like to tell us about, there is space at the end of the Questionnaire for you to write them.

Did your GP send you for any tests?

Thinking back to the time immediately before you diagnosis, did your GP send you for any of the following tests?

Please cross the appropriate boxes

7. Were you sent for any of these?

	Yes	Not sure	No
Blood test(s)	☐	☐	☐
CT Scan	☐	☐	☐
Ultrasound scan	☐	☐	☐
Barium Enema	☐	☐	☐
Sigmoidoscopy or colonoscopy (Looking at bowel with internal camera)	☐	☐	☐
X-ray	☐	☐	☐

Please go to Question 8

About you

The next questions relate to general information about you. It would help us to know more about the background of the people completing the questionnaire.

8. Which best describes your employment status?

Please cross one box only

- Employed full-time ☐
- Employed part-time ☐
- Self employed full-time ☐
- Self employed part-time ☐
- Unemployed (seeking work) ☐
- Unemployed (not seeking work) ☐
- Retired ☐
- Student ☐
- Permanently sick/disabled ☐
- Temporarily sick/disabled ☐
- Looking after family/home ☐
- Other, please describe ____

9. What is your highest level of qualification?

Please cross one box only

- Degree (or equivalent) ☐
- Diploma (or equivalent) ☐
- 'A' level ☐
- GCSE / 'O' level ☐
- None ☐
- Other, please specify ____

10. How would you describe your ethnicity

Please cross one box only

White ☐
or
- White British ☐
- White Irish ☐
- Other White background ☐

Mixed ☐
or
- White & Black Carribean ☐
- White & Black African ☐
- White & Asian ☐
- Other mixed background ☐

Black or Black British ☐
or
- Carribean ☐
- African ☐
- Other Black background ☐

Asian or Asian British ☐
or
- Indian ☐
- Pakistani ☐
- Bangladeshi ☐
- Other Asian background ☐

Chinese or other ethnic group ☐
or
- Chinese ☐
- Other ethnic group ☐

11.

Do you live alone?

Yes ☐ **Please go to Question 12**

No ☐ **Please say who you live with below**

Who do you live with?
(e.g. wife, husband, partner, family member)

12. Are you suffering from, or have you suffered from, any of the following in the past 2 years?

Please cross any that are applicable to you

- Asthma ☐
- Chronic Obstructive Pulmonary Disease (COPD) ☐
- Other lung disease (e.g. fibrosis, bronchiectatis etc) ☐
- Heart disease ☐
- Anxiety or depression ☐
- Inflammatory bowel disease ☐
- Irritable bowel syndrome ☐
- Peptic ulcer ☐
- Previous cancer ☐
- Diabetes ☐
- Arthritis ☐

13. About smoking

Please cross the appropriate statement

- Are you a current smoker? ☐
- Are you an ex-smoker? ☐
- Are you a non-smoker (never smoked)? ☐

14. Do you think you were more at risk of getting cancer because of your family history?

Yes ☐

No ☐

15. The following questions are about how completing the questionnaire made you feel.

A number of statements which people have used to describe themselves are given below. Read each statement and then cross the most appropriate box to indicate how you feel right now, at the moment. There are no right or wrong answers. Do not spend too much time on any one statement but give the answer which seems to describe your present feeling best.

		Not at all	Somewhat	Moderately	Very much
1.	I feel calm	☐	☐	☐	☐
2.	I am tense	☐	☐	☐	☐
3.	I feel upset	☐	☐	☐	☐
4.	I am relaxed	☐	☐	☐	☐
5.	I feel content	☐	☐	☐	☐
6.	I am worried	☐	☐	☐	☐

Please make sure you have answered all the questions.

If you have any other comments or there is anything else you would like to tell us about when you first noticed your symptoms, please do so using the space below.

If you feel you would like to talk to someone about this questionnaire at a later stage, please speak to the doctor or nurse who is looking after you, or be in touch with the research team, whose contact details are on your patient information sheet

Thank you for completing the questionnaire

Please give it to the research nurse

The next page is for the Researcher to complete

57727

56

Researcher - Post-completion Sheet

Researcher initials: ☐☐☐

Date questionnaire completed: Date ☐☐ / ☐☐ / ☐☐ Time ☐☐:☐☐

If not completed, give reason ☐

How long is it since the patient was told of his/her diagnosis? ☐

How long did it take the patient to complete the questionnaire?

- Less than 5 minutes ☐
- Between 5 and 10 minutes ☐
- Between 11 and 15 minutes ☐
- Longer than 15 minutes ☐

For the patient selected to <u>complete the questionnaire on their own</u>

Did the patient ask for any help? ☐ Yes ☐ No

If yes,

please specify what help was requested

___

___

please specify what help was given

___

___

For the patient selected for <u>the researcher-administered questionnaire</u>

Did the patient find any of the questions difficult? ☐ Yes ☐ No

If yes,

please specify which questions and what the difficulty was

___

___

___

___

Was the patient made anxious? ☐ Yes ☐ No

If yes,

please give details

___

___

___

___

Where did the patient complete the questionnaire?

(Please specify whether this was in a separate room alone, in a corner of the clinic, the ward or other

___

Thank you. Please now send the questionnaire and patient information sheet and consent form back to Cardiff University in the freepost envelope provided.

57727

PID - | | 5 | 7 |

Ymddiriedolaeth GIG Gogledd Orllewin Cymru
North West Wales NHS Trust

NHS WALES GIG CYMRU
Ymddiriedolaeth GIG Gogledd Cymru
North Wales NHS Trust

☐ GLAN CLWYD

CID - ☐ GWYNEDD

☐ MAELOR

Confidential Questionnaire

Looking At Your Symptoms Study

This Study has been funded by Cancer Research UK

CARDIFF UNIVERSITY PRIFYSGOL CAERDYDD
Ysgol Glinigol Gogledd Cymru North Wales Clinical School
CANCER RESEARCH UK
sewtu South East Wales Trials Unit Uned Ymchwil De-ddwyrain Cymru

Dear Patient

I am very grateful to you for agreeing to help us with this study.

I hope the information you supply will help us give people a diagnosis earlier.

If you feel you would like to talk to someone about this questionnaire at a later stage, please speak to the doctor or nurse who is looking after you, or be in touch with the research team, whose contact details are on your patient information sheet.

I can assure you that anything you tell us will be treated in the utmost confidence.

Yours sincerely

Dr Richard Neal
Senior Clinical Lecturer
Cardiff University
Chief Investigator, Symptoms Study

About your symptoms

These questions are about symptoms you noticed before your diagnosis. We are interested in symptoms which you think are related to your cancer.

Please put a cross in the appropriate box, or write an answer as required.

Looking back...

1. Did you have any symptoms that you feel were due to your cancer before you were diagnosed?

Yes ☐ **Please continue with Question 2**

No ☐ **Please go to Section 2 on Page 5**

2. What was the first symptom that made you think something might be wrong?

☐

Please go to Question 3

The following questions are about when you first noticed a symptom and when you first told your GP or nurse about it.

Please give an exact date if you can. Otherwise give your best estimate. (For example approximately how long ago, the month or season). You wish to refer to your diary or calendar if you have it with you.

Here is an example question:

Decrease in appetite

Did you have this symptom?

Yes ☒ **Please complete A then B or C below**

No ☐ **Please go to Section 2**

A When did you first notice this? d d / m m / y y **OR** Estimate '3 months ago' or 'June'

B When did you first tell your GP or nurse? 2 3 / 1 2 / 0 7 **OR** Estimate

OR

C Put a cross here if you didn't tell your GP or nurse ☐

3. Change in bowel habit

Did you have this symptom?

Yes ☐ **Please complete A then B or C below**

No ☐ **Please go to Question 4**

A When did you first notice this? ☐☐ / ☐☐ / ☐☐ **OR** ☐

B When did you first tell your GP or nurse? ☐☐ / ☐☐ / ☐☐ **OR** ☐

OR

C Put a cross here if you didn't tell your GP or nurse ☐

4. Bleeding from back passage

Did you have this symptom?

Yes ☐ **Please complete A then B or C below**

No ☐ **Please go to Section 2**

A When did you first notice this? ☐☐ / ☐☐ / ☐☐ **OR** ☐

B When did you first tell your GP or nurse? ☐☐ / ☐☐ / ☐☐ **OR** ☐

OR

C Put a cross here if you didn't tell your GP or nurse ☐

Please give an exact date if you can. Otherwise give your best estimate (for example approximately how long ago, the month or the season). You may wish to refer to your diary or calendar if you have it with you.

Did you have any of the following?

1. Decrease in appetite

Did you have this?

Yes ☐ **Please complete A then B or C below**

No ☐ **Please go to Question 2**

A When did you first notice this? ☐☐ / ☐☐ / ☐☐ **OR** ☐

B When did you first tell your GP or nurse? ☐☐ / ☐☐ / ☐☐ **OR** ☐

OR

C Put a cross here if you didn't tell your GP or nurse ☐

2. Unexplained weight loss

Did you have this?

Yes ☐ **Please complete A then B or C below**

No ☐ **Please go to Question 3**

A When did you first notice this? ☐☐ / ☐☐ / ☐☐ **OR** ☐

B When did you first tell your GP or nurse? ☐☐ / ☐☐ / ☐☐ **OR** ☐

OR

C Put a cross here if you didn't tell your GP or nurse ☐

3. Fatigue or tiredness that is unusual for you

Did you have this?

Yes ☐ **Please complete A then B or C below**

No ☐ **Please go to Question 4**

A When did you first notice this? ☐☐ / ☐☐ / ☐☐ **OR** ☐

B When did you first tell your GP or nurse? ☐☐ / ☐☐ / ☐☐ **OR** ☐

OR

C Put a cross here if you didn't tell your GP or nurse ☐

4. Feeling different 'in yourself' from usual

Did you have this?

Yes ☐ **Please complete A then B or C below**

No ☐ **Please go to Question 5**

A When did you first notice this? ☐☐ / ☐☐ / ☐☐ **OR** ☐

B When did you first tell your GP or nurse? ☐☐ / ☐☐ / ☐☐ **OR** ☐

OR

C Put a cross here if you didn't tell your GP or nurse ☐

Please try and describe what the feeling was

If you had any further symptoms which you feel are relevant to your cancer diagnosis, please list them below. If not, please go to Question 7.

Please describe the symptom here and complete A then B or C below

5.

A When did you first notice this? ☐☐ / ☐☐ / ☐☐ **OR** ☐

B When did you first tell your GP or nurse? ☐☐ / ☐☐ / ☐☐ **OR** ☐

OR

C Put a cross here if you didn't tell your GP or nurse ☐

If you had another symptom you feel is relevant, please write it below, otherwise go to Question 7.

Please describe the symptom here and complete A then B or C below

6.

A When did you first notice this? ☐☐ / ☐☐ / ☐☐ **OR** ☐

B When did you first tell your GP or nurse? ☐☐ / ☐☐ / ☐☐ **OR** ☐

OR

C Put a cross here if you didn't tell your GP or nurse ☐

If you had further symptoms you would like to tell us about, there is space at the end of the Questionnaire for you to write them.

Did your GP send you for any tests?

Thinking back to the time immediately before you diagnosis, did your GP send you for any of the following tests?

Please cross the appropriate boxes

7. Were you sent for any of these?

	Yes	Not sure	No
Blood test(s)	☐	☐	☐
CT Scan	☐	☐	☐
Ultrasound scan	☐	☐	☐
Barium Enema	☐	☐	☐
Sigmoidoscopy or colonoscopy (Looking at bowel with internal camera)	☐	☐	☐
X-ray	☐	☐	☐

Please go to Question 8

About you

The next questions relate to general information about you. It would help us to know more about the background of the people completing the questionnaire.

8. Which best describes your employment status?

Please cross one box only

- Employed full-time ☐
- Employed part-time ☐
- Self employed full-time ☐
- Self employed part-time ☐
- Unemployed (seeking work) ☐
- Unemployed (not seeking work) ☐
- Retired ☐
- Student ☐
- Permanently sick/disabled ☐
- Temporarily sick/disabled ☐
- Looking after family/home ☐
- Other, please describe ________

9. What is your highest level of qualification?

Please cross one box only

- Degree (or equivalent) ☐
- Diploma (or equivalent) ☐
- 'A' level ☐
- GCSE / 'O' level ☐
- None ☐
- Other, please specify ________

10. **How would you describe your ethnicity**

Please cross one box only

White ☐
or
- White British ☐
- White Irish ☐
- Other White background ☐

Mixed ☐
or
- White & Black Carribean ☐
- White & Black African ☐
- White & Asian ☐
- Other mixed background ☐

Black or Black British ☐
or
- Carribean ☐
- African ☐
- Other Black background ☐

Asian or Asian British ☐
or
- Indian ☐
- Pakistani ☐
- Bangladeshi ☐
- Other Asian background ☐

Chinese or other ethnic group ☐
or
- Chinese ☐
- Other ethnic group ☐

11.

Do you live alone?

Yes ☐ **Please go to Question 12**

No ☐ **Please say who you live with below**

Who do you live with?
(e.g. wife, husband, partner, family member)

12. **Are you suffering from, or have you suffered from, any of the following in the past 2 years?**

Please cross any that are applicable to you

- Asthma ☐
- Chronic Obstructive Pulmonary Disease (COPD) ☐
- Other lung disease (e.g. fibrosis, bronchiectatis etc) ☐
- Heart disease ☐
- Anxiety or depression ☐
- Inflammatory bowel disease ☐
- Irritable bowel syndrome ☐
- Peptic ulcer ☐
- Previous cancer ☐
- Diabetes ☐
- Arthritis ☐

13. **About smoking**

Please cross the appropriate statement

- Are you a current smoker? ☐
- Are you an ex-smoker? ☐
- Are you a non-smoker (never smoked)? ☐

14. **Do you think you were more at risk of getting cancer because of your family history?**

Yes ☐
No ☐

15. The following questions are about how completing the questionnaire made you feel.

A number of statements which people have used to describe themselves are given below. Read each statement and then cross the most appropriate box to indicate how you feel right now, at the moment. There are no right or wrong answers. Do not spend too much time on any one statement but give the answer which seems to describe your present feeling best.

		Not at all	Somewhat	Moderately	Very much
1.	I feel calm	☐	☐	☐	☐
2.	I am tense	☐	☐	☐	☐
3.	I feel upset	☐	☐	☐	☐
4.	I am relaxed	☐	☐	☐	☐
5.	I feel content	☐	☐	☐	☐
6.	I am worried	☐	☐	☐	☐

Please make sure you have answered all the questions.

If you have any other comments or there is anything else you would like to tell us about when you first noticed your symptoms, please do so using the space below.

If you feel you would like to talk to someone about this questionnaire at a later stage, please speak to the doctor or nurse who is looking after you, or be in touch with the research team, whose contact details are on your patient information sheet

Thank you for completing the questionnaire

Please give it to the research nurse

The next page is for the Researcher to complete

57727

5 7

Researcher - Post-completion Sheet

Researcher initials: ☐☐☐

Date questionnaire completed: Date ☐☐ / ☐☐ / ☐☐ Time ☐☐:☐☐

If not completed, give reason ☐

How long is it since the patient was told of his/her diagnosis? ☐

How long did it take the patient to complete the questionnaire?

- Less than 5 minutes ☐
- Between 5 and 10 minutes ☐
- Between 11 and 15 minutes ☐
- Longer than 15 minutes ☐

For the patient selected to complete the questionnaire on their own

Did the patient ask for any help? ☐ Yes ☐ No

If yes,

please specify what help was requested

___

___

please specify what help was given

___

___

For the patient selected for the researcher-administered questionnaire

Did the patient find any of the questions difficult? ☐ Yes ☐ No

If yes,

please specify which questions and what the difficulty was

___

___

___

___

Was the patient made anxious? ☐ Yes ☐ No

If yes,

please give details

___

___

___

___

Where did the patient complete the questionnaire?

(Please specify whether this was in a separate room alone, in a corner of the clinic, the ward or other

___

Thank you. Please now send the questionnaire and patient information sheet and consent form back to Cardiff University in the freepost envelope provided.

57727

PID - | 5 | 8

Ymddiriedolaeth GIG Gogledd Orllewin Cymru
North West Wales NHS Trust

NHS WALES GIG CYMRU
Ymddiriedolaeth GIG Gogledd Cymru
North Wales NHS Trust

CID -
- ☐ GLAN CLWYD
- ☐ GWYNEDD
- ☐ MAELOR

Confidential Questionnaire

Looking At Your Symptoms Study

This Study has been funded by Cancer Research UK

Cardiff University / Prifysgol Caerdydd

Ysgol Glinigol Gogledd Cymru / North Wales Clinical School

Cancer Research UK

sewtu — South East Wales Trials Unit / Uned Ymchwil De-ddwyrain Cymru

Dear Patient

I am very grateful to you for agreeing to help us with this study.

I hope the information you supply will help us give people a diagnosis earlier.

If you feel you would like to talk to someone about this questionnaire at a later stage, please speak to the doctor or nurse who is looking after you, or be in touch with the research team, whose contact details are on your patient information sheet.

I can assure you that anything you tell us will be treated in the utmost confidence.

Yours sincerely

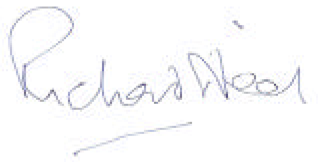

Dr Richard Neal
Senior Clinical Lecturer
Cardiff University
Chief Investigator, Symptoms Study

About your symptoms

These questions are about symptoms you noticed before your diagnosis. We are interested in symptoms which you think are related to your cancer.

Please put a cross in the appropriate box, or write an answer as required.

Looking back...

1. Did you have any symptoms that you feel were due to your cancer before you were diagnosed?

Yes ☐ **Please continue with Question 2**

No ☐ **Please go to Section 2 on Page 5**

2. What was the first symptom that made you think something might be wrong?

☐

Please go to Question 3

The following questions are about when you first noticed a symptom and when you first told your GP or nurse about it.

Please give an exact date if you can. Otherwise give your best estimate. (For example approximately how long ago, the month or season). You wish to refer to your diary or calendar if you have it with you.

Here is an example question:

Decrease in appetite

Did you have this symptom?

Yes ☒ **Please complete A then B or C below**

No ☐ **Please go to Section 2**

A When did you first notice this? d d / m m / y y **OR** Estimate '3 months ago' or 'June'

B When did you first tell your GP or nurse? 2 3 / 1 2 / 0 7 **OR** Estimate

OR

C Put a cross here if you didn't tell your GP or nurse ☐

3. Change in bowel habit

Did you have this symptom?

Yes ☐ **Please complete A then B or C below**

No ☐ **Please go to Question 4**

A When did you <u>first notice</u> this? ☐☐ / ☐☐ / ☐☐ **OR** ☐

B When did you <u>first tell</u> your GP or nurse? ☐☐ / ☐☐ / ☐☐ **OR** ☐

OR

C Put a cross here if you <u>didn't tell</u> your GP or nurse ☐

4. Bleeding from back passage

Did you have this symptom?

Yes ☐ **Please complete A then B or C below**

No ☐ **Please go to Section 2**

A When did you <u>first notice</u> this? ☐☐ / ☐☐ / ☐☐ **OR** ☐

B When did you <u>first tell</u> your GP or nurse? ☐☐ / ☐☐ / ☐☐ **OR** ☐

OR

C Put a cross here if you <u>didn't tell</u> your GP or nurse ☐

Section 2

Please give an exact date if you can. Otherwise give your best estimate (for example approximately how long ago, the month or the season). You may wish to refer to your diary or calendar if you have it with you.

Did you have any of the following?

1. Decrease in appetite

Did you have this?

Yes ☐ **Please complete A then B or C below**

No ☐ **Please go to Question 2**

A When did you first notice this? ☐☐ / ☐☐ / ☐☐ **OR** ☐

B When did you first tell your GP or nurse? ☐☐ / ☐☐ / ☐☐ **OR** ☐

OR

C Put a cross here if you didn't tell your GP or nurse ☐

2. Unexplained weight loss

Did you have this?

Yes ☐ **Please complete A then B or C below**

No ☐ **Please go to Question 3**

A When did you first notice this? ☐☐ / ☐☐ / ☐☐ **OR** ☐

B When did you first tell your GP or nurse? ☐☐ / ☐☐ / ☐☐ **OR** ☐

OR

C Put a cross here if you didn't tell your GP or nurse ☐

3. **Fatigue or tiredness that is unusual for you**

Did you have this?

Yes ☐ **Please complete A then B or C below**

No ☐ **Please go to Question 4**

A When did you <u>first notice</u> this? ☐☐ / ☐☐ / ☐☐ **OR** ☐

B When did you <u>first tell</u> your GP or nurse? ☐☐ / ☐☐ / ☐☐ **OR** ☐

OR

C Put a cross here if you <u>didn't tell</u> your GP or nurse ☐

4. **Feeling different 'in yourself' from usual**

Did you have this?

Yes ☐ **Please complete A then B or C below**

No ☐ **Please go to Question 5**

A When did you <u>first notice</u> this? ☐☐ / ☐☐ / ☐☐ **OR** ☐

B When did you <u>first tell</u> your GP or nurse? ☐☐ / ☐☐ / ☐☐ **OR** ☐

OR

C Put a cross here if you <u>didn't tell</u> your GP or nurse ☐

Please try and describe what the feeling was

If you had any further symptoms which you feel are relevant to your cancer diagnosis, please list them below. If not, please go to Question 7.

Please describe the symptom here and complete A then B or C below

5.

A When did you first notice this? __ / __ / __ **OR**

B When did you first tell your GP or nurse? __ / __ / __ **OR**

OR

C Put a cross here if you didn't tell your GP or nurse ☐

If you had another symptom you feel is relevant, please write it below, otherwise go to Question 7.

Please describe the symptom here and complete A then B or C below

6.

A When did you first notice this? __ / __ / __ **OR**

B When did you first tell your GP or nurse? __ / __ / __ **OR**

OR

C Put a cross here if you didn't tell your GP or nurse ☐

If you had further symptoms you would like to tell us about, there is space at the end of the Questionnaire for you to write them.

Did your GP send you for any tests?

Thinking back to the time immediately before you diagnosis, did your GP send you for any of the following tests?

Please cross the appropriate boxes

7. Were you sent for any of these?

	Yes	Not sure	No
Blood test(s)	☐	☐	☐
CT Scan	☐	☐	☐
Ultrasound scan	☐	☐	☐
Barium Enema	☐	☐	☐
Sigmoidoscopy or colonoscopy (Looking at bowel with internal camera)	☐	☐	☐
X-ray	☐	☐	☐

Please go to Question 8

About you

The next questions relate to general information about you. It would help us to know more about the background of the people completing the questionnaire.

8. Which best describes your employment status?

Please cross one box only

- Employed full-time ☐
- Employed part-time ☐
- Self employed full-time ☐
- Self employed part-time ☐
- Unemployed (seeking work) ☐
- Unemployed (not seeking work) ☐
- Retired ☐
- Student ☐
- Permanently sick/disabled ☐
- Temporarily sick/disabled ☐
- Looking after family/home ☐
- Other, please describe

9. What is your highest level of qualification?

Please cross one box only

- Degree (or equivalent) ☐
- Diploma (or equivalent) ☐
- 'A' level ☐
- GCSE / 'O' level ☐
- None ☐
- Other, please specify

10. How would you describe your ethnicity

Please cross one box only

White ☐
or
- White British ☐
- White Irish ☐
- Other White background ☐

Mixed ☐
or
- White & Black Carribean ☐
- White & Black African ☐
- White & Asian ☐
- Other mixed background ☐

Black or Black British ☐
or
- Carribean ☐
- African ☐
- Other Black background ☐

Asian or Asian British ☐
or
- Indian ☐
- Pakistani ☐
- Bangladeshi ☐
- Other Asian background ☐

Chinese or other ethnic group ☐
or
- Chinese ☐
- Other ethnic group ☐

11.

Do you live alone?

Yes ☐ **Please go to Question 12**

No ☐ **Please say who you live with below**

Who do you live with?
(e.g. wife, husband, partner, family member)

12. **Are you suffering from, or have you suffered from, any of the following in the past 2 years?**

Please cross any that are applicable to you

- Asthma ☐
- Chronic Obstructive Pulmonary Disease (COPD) ☐
- Other lung disease (e.g. fibrosis, bronchiectatis etc) ☐
- Heart disease ☐
- Anxiety or depression ☐
- Inflammatory bowel disease ☐
- Irritable bowel syndrome ☐
- Peptic ulcer ☐
- Previous cancer ☐
- Diabetes ☐
- Arthritis ☐

13. **About smoking**

Please cross the appropriate statement

- Are you a current smoker? ☐
- Are you an ex-smoker? ☐
- Are you a non-smoker (never smoked)? ☐

14. **Do you think you were more at risk of getting cancer because of your family history?**

Yes ☐
No ☐

15. The following questions are about how completing the questionnaire made you feel.

A number of statements which people have used to describe themselves are given below. Read each statement and then cross the most appropriate box to indicate how you feel right now, at the moment. There are no right or wrong answers. Do not spend too much time on any one statement but give the answer which seems to describe your present feeling best.

		Not at all	Somewhat	Moderately	Very much
1.	I feel calm	☐	☐	☐	☐
2.	I am tense	☐	☐	☐	☐
3.	I feel upset	☐	☐	☐	☐
4.	I am relaxed	☐	☐	☐	☐
5.	I feel content	☐	☐	☐	☐
6.	I am worried	☐	☐	☐	☐

Please make sure you have answered all the questions.

If you have any other comments or there is anything else you would like to tell us about when you first noticed your symptoms, please do so using the space below.

If you feel you would like to talk to someone about this questionnaire at a later stage, please speak to the doctor or nurse who is looking after you, or be in touch with the research team, whose contact details are on your patient information sheet

Thank you for completing the questionnaire

Please give it to the research nurse

The next page is for the Researcher to complete

Researcher - Post-completion Sheet

Researcher initials: ☐☐☐

Date questionnaire completed: Date ☐☐ / ☐☐ / ☐☐ Time ☐☐:☐☐

If not completed, give reason ☐

How long is it since the patient was told of his/her diagnosis? ☐

How long did it take the patient to complete the questionnaire?

- Less than 5 minutes ☐
- Between 5 and 10 minutes ☐
- Between 11 and 15 minutes ☐
- Longer than 15 minutes ☐

For the patient selected to <u>complete the questionnaire on their own</u>

Did the patient ask for any help? ☐ Yes ☐ No

If yes,

please specify what help was requested

____

____

please specify what help was given

____

____

For the patient selected for <u>the researcher-administered questionnaire</u>

Did the patient find any of the questions difficult? ☐ Yes ☐ No

If yes,

please specify which questions and what the difficulty was

____

____

____

____

Was the patient made anxious? ☐ Yes ☐ No

If yes,

please give details

____

____

____

____

Where did the patient complete the questionnaire?

(Please specify whether this was in a separate room alone, in a corner of the clinic, the ward or other

____

Thank you. Please now send the questionnaire and patient information sheet and consent form back to Cardiff University in the freepost envelope provided.

57727

PID - 59

Ymddiriedolaeth GIG Gogledd Orllewin Cymru
North West Wales NHS Trust

NHS WALES GIG CYMRU
Ymddiriedolaeth GIG Gogledd Cymru
North Wales NHS Trust

☐ GLAN CLWYD

CID - ☐ GWYNEDD

☐ MAELOR

Confidential Questionnaire

Looking At Your Symptoms Study

This Study has been funded by Cancer Research UK

Cardiff University / Prifysgol Caerdydd
Ysgol Glinigol Gogledd Cymru / North Wales Clinical School
Cancer Research UK
sewtu South East Wales Trials Unit / Uned Ymchwil De-ddwyrain Cymru

Dear Patient

I am very grateful to you for agreeing to help us with this study.

I hope the information you supply will help us give people a diagnosis earlier.

If you feel you would like to talk to someone about this questionnaire at a later stage, please speak to the doctor or nurse who is looking after you, or be in touch with the research team, whose contact details are on your patient information sheet.

I can assure you that anything you tell us will be treated in the utmost confidence.

Yours sincerely

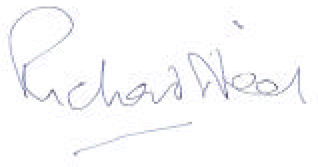

Dr Richard Neal
Senior Clinical Lecturer
Cardiff University
Chief Investigator, Symptoms Study

About your symptoms

These questions are about symptoms you noticed before your diagnosis. We are interested in symptoms which you think are related to your cancer.

Please put a cross in the appropriate box, or write an answer as required.

Looking back...

1. Did you have any symptoms that you feel were due to your cancer before you were diagnosed?

Yes ☐ **Please continue with Question 2**

No ☐ **Please go to Section 2 on Page 5**

2. What was the first symptom that made you think something might be wrong?

☐

Please go to Question 3

The following questions are about when you first noticed a symptom and when you first told your GP or nurse about it.

Please give an exact date if you can. Otherwise give your best estimate. (For example approximately how long ago, the month or season). You wish to refer to your diary or calendar if you have it with you.

Here is an example question:

Decrease in appetite

Did you have this symptom?

Yes ☒ **Please complete A then B or C below**

No ☐ **Please go to Section 2**

A When did you first notice this? d d / m m / y y **OR** Estimate '3 months ago' or 'June'

B When did you first tell your GP or nurse? 2 3 / 1 2 / 0 7 **OR** Estimate

OR

C Put a cross here if you didn't tell your GP or nurse ☐

3. Change in bowel habit

Did you have this symptom?

Yes ☐ **Please complete A then B or C below**

No ☐ **Please go to Question 4**

A When did you first notice this? ☐☐ / ☐☐ / ☐☐ **OR** ☐

B When did you first tell your GP or nurse? ☐☐ / ☐☐ / ☐☐ **OR** ☐

OR

C Put a cross here if you didn't tell your GP or nurse ☐

4. Bleeding from back passage

Did you have this symptom?

Yes ☐ **Please complete A then B or C below**

No ☐ **Please go to Section 2**

A When did you first notice this? ☐☐ / ☐☐ / ☐☐ **OR** ☐

B When did you first tell your GP or nurse? ☐☐ / ☐☐ / ☐☐ **OR** ☐

OR

C Put a cross here if you didn't tell your GP or nurse ☐

Section 2

Please give an exact date if you can. Otherwise give your best estimate (for example approximately how long ago, the month or the season). You may wish to refer to your diary or calendar if you have it with you.

Did you have any of the following?

1. Decrease in appetite

Did you have this?

Yes ☐ **Please complete A then B or C below**

No ☐ **Please go to Question 2**

A When did you first notice this? ☐☐ / ☐☐ / ☐☐ **OR** ☐

B When did you first tell your GP or nurse? ☐☐ / ☐☐ / ☐☐ **OR** ☐

OR

C Put a cross here if you didn't tell your GP or nurse ☐

2. Unexplained weight loss

Did you have this?

Yes ☐ **Please complete A then B or C below**

No ☐ **Please go to Question 3**

A When did you first notice this? ☐☐ / ☐☐ / ☐☐ **OR** ☐

B When did you first tell your GP or nurse? ☐☐ / ☐☐ / ☐☐ **OR** ☐

OR

C Put a cross here if you didn't tell your GP or nurse ☐

3. **Fatigue or tiredness that is unusual for you**

Did you have this?

Yes ☐ **Please complete A then B or C below**

No ☐ **Please go to Question 4**

A When did you first notice this? ☐☐ / ☐☐ / ☐☐ **OR** ☐

B When did you first tell your GP or nurse? ☐☐ / ☐☐ / ☐☐ **OR** ☐

OR

C Put a cross here if you didn't tell your GP or nurse ☐

4. **Feeling different 'in yourself' from usual**

Did you have this?

Yes ☐ **Please complete A then B or C below**

No ☐ **Please go to Question 5**

A When did you first notice this? ☐☐ / ☐☐ / ☐☐ **OR** ☐

B When did you first tell your GP or nurse? ☐☐ / ☐☐ / ☐☐ **OR** ☐

OR

C Put a cross here if you didn't tell your GP or nurse ☐

Please try and describe what the feeling was

If you had any further symptoms which you feel are relevant to your cancer diagnosis, please list them below. If not, please go to Question 7.

Please describe the symptom here and complete A then B or C below

5.

**A**	When did you first notice this?	☐☐ / ☐☐ / ☐☐ **OR** ☐
**B**	When did you first tell your GP or nurse?	☐☐ / ☐☐ / ☐☐ **OR** ☐
	**OR**	
**C**	Put a cross here if you didn't tell your GP or nurse	☐

If you had another symptom you feel is relevant, please write it below, otherwise go to Question 7.

Please describe the symptom here and complete A then B or C below

6.

**A**	When did you first notice this?	☐☐ / ☐☐ / ☐☐ **OR** ☐
**B**	When did you first tell your GP or nurse?	☐☐ / ☐☐ / ☐☐ **OR** ☐
	**OR**	
**C**	Put a cross here if you didn't tell your GP or nurse	☐

If you had further symptoms you would like to tell us about, there is space at the end of the Questionnaire for you to write them.

Did your GP send you for any tests?

Thinking back to the time immediately before you diagnosis, did your GP send you for any of the following tests?

Please cross the appropriate boxes

7. Were you sent for any of these?

	Yes	Not sure	No
Blood test(s)	☐	☐	☐
CT Scan	☐	☐	☐
Ultrasound scan	☐	☐	☐
Barium Enema	☐	☐	☐
Sigmoidoscopy or colonoscopy (Looking at bowel with internal camera)	☐	☐	☐
X-ray	☐	☐	☐

Please go to Question 8

About you

The next questions relate to general information about you. It would help us to know more about the background of the people completing the questionnaire.

8. Which best describes your employment status?

Please cross one box only

- Employed full-time ☐
- Employed part-time ☐
- Self employed full-time ☐
- Self employed part-time ☐
- Unemployed (seeking work) ☐
- Unemployed (not seeking work) ☐
- Retired ☐
- Student ☐
- Permanently sick/disabled ☐
- Temporarily sick/disabled ☐
- Looking after family/home ☐

Other, please describe

9. What is your highest level of qualification?

Please cross one box only

- Degree (or equivalent) ☐
- Diploma (or equivalent) ☐
- 'A' level ☐
- GCSE / 'O' level ☐
- None ☐

Other, please specify

10. How would you describe your ethnicity

Please cross one box only

White ☐
or
- White British ☐
- White Irish ☐
- Other White background ☐

Mixed ☐
or
- White & Black Carribean ☐
- White & Black African ☐
- White & Asian ☐
- Other mixed background ☐

Black or Black British ☐
or
- Carribean ☐
- African ☐
- Other Black background ☐

Asian or Asian British ☐
or
- Indian ☐
- Pakistani ☐
- Bangladeshi ☐
- Other Asian background ☐

Chinese or other ethnic group ☐
or
- Chinese ☐
- Other ethnic group ☐

11.

Do you live alone?

Yes ☐ **Please go to Question 12**

No ☐ **Please say who you live with below**

Who do you live with?
(e.g. wife, husband, partner, family member)

12. **Are you suffering from, or have you suffered from, any of the following in the past 2 years?**

Please cross any that are applicable to you

- Asthma ☐
- Chronic Obstructive Pulmonary Disease (COPD) ☐
- Other lung disease (e.g. fibrosis, bronchiectatis etc) ☐
- Heart disease ☐
- Anxiety or depression ☐
- Inflammatory bowel disease ☐
- Irritable bowel syndrome ☐
- Peptic ulcer ☐
- Previous cancer ☐
- Diabetes ☐
- Arthritis ☐

13. **About smoking**

Please cross the appropriate statement

- Are you a current smoker? ☐
- Are you an ex-smoker? ☐
- Are you a non-smoker (never smoked)? ☐

14. **Do you think you were more at risk of getting cancer because of your family history?**

Yes ☐
No ☐

15. **The following questions are about how completing the questionnaire made you feel.**

A number of statements which people have used to describe themselves are given below. Read each statement and then cross the most appropriate box to indicate how you feel right now, at the moment. There are no right or wrong answers. Do not spend too much time on any one statement but give the answer which seems to describe your present feeling best.

		Not at all	Somewhat	Moderately	Very much
1.	I feel calm	☐	☐	☐	☐
2.	I am tense	☐	☐	☐	☐
3.	I feel upset	☐	☐	☐	☐
4.	I am relaxed	☐	☐	☐	☐
5.	I feel content	☐	☐	☐	☐
6.	I am worried	☐	☐	☐	☐

Please make sure you have answered all the questions.

If you have any other comments or there is anything else you would like to tell us about when you first noticed your symptoms, please do so using the space below.

If you feel you would like to talk to someone about this questionnaire at a later stage, please speak to the doctor or nurse who is looking after you, or be in touch with the research team, whose contact details are on your patient information sheet

Thank you for completing the questionnaire

Please give it to the research nurse

The next page is for the Researcher to complete

57727

59

Researcher - Post-completion Sheet

Researcher initials: ☐☐☐

Date questionnaire completed: Date ☐☐ / ☐☐ / ☐☐ Time ☐☐:☐☐

If not completed, give reason ☐

How long is it since the patient was told of his/her diagnosis? ☐

How long did it take the patient to complete the questionnaire?

- Less than 5 minutes ☐
- Between 5 and 10 minutes ☐
- Between 11 and 15 minutes ☐
- Longer than 15 minutes ☐

For the patient selected to <u>complete the questionnaire on their own</u>

Did the patient ask for any help? ☐ Yes ☐ No

If yes,

please specify what help was requested

____

____

please specify what help was given

____

____

For the patient selected for <u>the researcher-administered questionnaire</u>

Did the patient find any of the questions difficult? ☐ Yes ☐ No

If yes,

please specify which questions and what the difficulty was

____

____

____

____

Was the patient made anxious? ☐ Yes ☐ No

If yes,

please give details

____

____

____

____

Where did the patient complete the questionnaire?

(Please specify whether this was in a separate room alone, in a corner of the clinic, the ward or other

____

Thank you. Please now send the questionnaire and patient information sheet and consent form back to Cardiff University in the freepost envelope provided.

57727

PID - 6 0

Ymddiriedolaeth GIG Gogledd Orllewin Cymru
North West Wales NHS Trust

NHS WALES GIG CYMRU
Ymddiriedolaeth GIG Gogledd Cymru
North Wales NHS Trust

☐ GLAN CLWYD

CID - ☐ GWYNEDD

☐ MAELOR

Confidential Questionnaire

Looking At Your Symptoms Study

This Study has been funded by Cancer Research UK

Cardiff University / Prifysgol Caerdydd
Ysgol Glinigol Gogledd Cymru / North Wales Clinical School
Cancer Research UK
sewtu South East Wales Trials Unit / Uned Ymchwil De-ddwyrain Cymru

Dear Patient

I am very grateful to you for agreeing to help us with this study.

I hope the information you supply will help us give people a diagnosis earlier.

If you feel you would like to talk to someone about this questionnaire at a later stage, please speak to the doctor or nurse who is looking after you, or be in touch with the research team, whose contact details are on your patient information sheet.

I can assure you that anything you tell us will be treated in the utmost confidence.

Yours sincerely

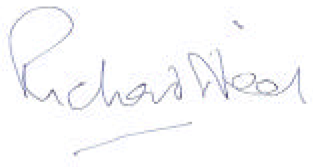

Dr Richard Neal
Senior Clinical Lecturer
Cardiff University
Chief Investigator, Symptoms Study

About your symptoms

These questions are about symptoms you noticed before your diagnosis. We are interested in symptoms which you think are related to your cancer.

Please put a cross in the appropriate box, or write an answer as required.

Looking back...

1. Did you have any symptoms that you feel were due to your cancer before you were diagnosed?

Yes ☐ **Please continue with Question 2**

No ☐ **Please go to Section 2 on Page 5**

2. What was the first symptom that made you think something might be wrong?

☐

Please go to Question 3

The following questions are about when you first noticed a symptom and when you first told your GP or nurse about it.

Please give an exact date if you can. Otherwise give your best estimate. (For example approximately how long ago, the month or season). You wish to refer to your diary or calendar if you have it with you.

Here is an example question:

Decrease in appetite

Did you have this symptom?

Yes ☒ **Please complete A then B or C below**

No ☐ **Please go to Section 2**

A When did you first notice this? d d / m m / y y **OR** Estimate '3 months ago' or 'June'

B When did you first tell your GP or nurse? 2 3 / 1 2 / 0 7 **OR** Estimate

OR

C Put a cross here if you didn't tell your GP or nurse ☐

3. Change in bowel habit

Did you have this symptom?

Yes ☐ **Please complete A then B or C below**

No ☐ **Please go to Question 4**

A When did you first notice this? ☐☐ / ☐☐ / ☐☐ **OR** ☐

B When did you first tell your GP or nurse? ☐☐ / ☐☐ / ☐☐ **OR** ☐

OR

C Put a cross here if you didn't tell your GP or nurse ☐

4. Bleeding from back passage

Did you have this symptom?

Yes ☐ **Please complete A then B or C below**

No ☐ **Please go to Section 2**

A When did you first notice this? ☐☐ / ☐☐ / ☐☐ **OR** ☐

B When did you first tell your GP or nurse? ☐☐ / ☐☐ / ☐☐ **OR** ☐

OR

C Put a cross here if you didn't tell your GP or nurse ☐

Please give an exact date if you can. Otherwise give your best estimate (for example approximately how long ago, the month or the season). You may wish to refer to your diary or calendar if you have it with you.

Did you have any of the following?

1. Decrease in appetite

Did you have this?

Yes ☐ **Please complete A then B or C below**

No ☐ **Please go to Question 2**

A When did you first notice this? ☐☐ / ☐☐ / ☐☐ **OR** ☐

B When did you first tell your GP or nurse? ☐☐ / ☐☐ / ☐☐ **OR** ☐

OR

C Put a cross here if you didn't tell your GP or nurse ☐

2. Unexplained weight loss

Did you have this?

Yes ☐ **Please complete A then B or C below**

No ☐ **Please go to Question 3**

A When did you first notice this? ☐☐ / ☐☐ / ☐☐ **OR** ☐

B When did you first tell your GP or nurse? ☐☐ / ☐☐ / ☐☐ **OR** ☐

OR

C Put a cross here if you didn't tell your GP or nurse ☐

3. Fatigue or tiredness that is unusual for you

Did you have this?

Yes ☐ **Please complete A then B or C below**

No ☐ **Please go to Question 4**

A When did you first notice this? ☐☐ / ☐☐ / ☐☐ **OR** ☐

B When did you first tell your GP or nurse? ☐☐ / ☐☐ / ☐☐ **OR** ☐

OR

C Put a cross here if you didn't tell your GP or nurse ☐

4. Feeling different 'in yourself' from usual

Did you have this?

Yes ☐ **Please complete A then B or C below**

No ☐ **Please go to Question 5**

A When did you first notice this? ☐☐ / ☐☐ / ☐☐ **OR** ☐

B When did you first tell your GP or nurse? ☐☐ / ☐☐ / ☐☐ **OR** ☐

OR

C Put a cross here if you didn't tell your GP or nurse ☐

Please try and describe what the feeling was

If you had any further symptoms which you feel are relevant to your cancer diagnosis, please list them below. If not, please go to Question 7.

Please describe the symptom here and complete A then B or C below

5.

A When did you first notice this? __/__/__ **OR**

B When did you first tell your GP or nurse? __/__/__ **OR**

OR

C Put a cross here if you didn't tell your GP or nurse ☐

If you had another symptom you feel is relevant, please write it below, otherwise go to Question 7.

Please describe the symptom here and complete A then B or C below

6.

A When did you first notice this? __/__/__ **OR**

B When did you first tell your GP or nurse? __/__/__ **OR**

OR

C Put a cross here if you didn't tell your GP or nurse ☐

If you had further symptoms you would like to tell us about, there is space at the end of the Questionnaire for you to write them.

Did your GP send you for any tests?

Thinking back to the time immediately before you diagnosis, did your GP send you for any of the following tests?

Please cross the appropriate boxes

7. Were you sent for any of these?

	Yes	Not sure	No
Blood test(s)	☐	☐	☐
CT Scan	☐	☐	☐
Ultrasound scan	☐	☐	☐
Barium Enema	☐	☐	☐
Sigmoidoscopy or colonoscopy (Looking at bowel with internal camera)	☐	☐	☐
X-ray	☐	☐	☐

Please go to Question 8

About you

The next questions relate to general information about you. It would help us to know more about the background of the people completing the questionnaire.

8. **Which best describes your employment status?**

Please cross one box only

- Employed full-time ☐
- Employed part-time ☐
- Self employed full-time ☐
- Self employed part-time ☐
- Unemployed (seeking work) ☐
- Unemployed (not seeking work) ☐
- Retired ☐
- Student ☐
- Permanently sick/disabled ☐
- Temporarily sick/disabled ☐
- Looking after family/home ☐
- Other, please describe ______

9. **What is your highest level of qualification?**

Please cross one box only

- Degree (or equivalent) ☐
- Diploma (or equivalent) ☐
- 'A' level ☐
- GCSE / 'O' level ☐
- None ☐
- Other, please specify ______

10. How would you describe your ethnicity

Please cross one box only

White ☐
or
- White British ☐
- White Irish ☐
- Other White background ☐

Mixed ☐
or
- White & Black Carribean ☐
- White & Black African ☐
- White & Asian ☐
- Other mixed background ☐

Black or Black British ☐
or
- Carribean ☐
- African ☐
- Other Black background ☐

Asian or Asian British ☐
or
- Indian ☐
- Pakistani ☐
- Bangladeshi ☐
- Other Asian background ☐

Chinese or other ethnic group ☐
or
- Chinese ☐
- Other ethnic group ☐

11.

Do you live alone?

Yes ☐ **Please go to Question 12**

No ☐ **Please say who you live with below**

Who do you live with?
(e.g. wife, husband, partner, family member)

12. **Are you suffering from, or have you suffered from, any of the following in the past 2 years?**

Please cross any that are applicable to you

- Asthma ☐
- Chronic Obstructive Pulmonary Disease (COPD) ☐
- Other lung disease (e.g. fibrosis, bronchiectatis etc) ☐
- Heart disease ☐
- Anxiety or depression ☐
- Inflammatory bowel disease ☐
- Irritable bowel syndrome ☐
- Peptic ulcer ☐
- Previous cancer ☐
- Diabetes ☐
- Arthritis ☐

13. **About smoking**

Please cross the appropriate statement

- Are you a current smoker? ☐
- Are you an ex-smoker? ☐
- Are you a non-smoker (never smoked)? ☐

14. **Do you think you were more at risk of getting cancer because of your family history?**

Yes ☐
No ☐

15. **The following questions are about how completing the questionnaire made you feel.**

A number of statements which people have used to describe themselves are given below. Read each statement and then cross the most appropriate box to indicate how you feel right now, at the moment. There are no right or wrong answers. Do not spend too much time on any one statement but give the answer which seems to describe your present feeling best.

		Not at all	Somewhat	Moderately	Very much
1.	I feel calm	☐	☐	☐	☐
2.	I am tense	☐	☐	☐	☐
3.	I feel upset	☐	☐	☐	☐
4.	I am relaxed	☐	☐	☐	☐
5.	I feel content	☐	☐	☐	☐
6.	I am worried	☐	☐	☐	☐

Please make sure you have answered all the questions.

If you have any other comments or there is anything else you would like to tell us about when you first noticed your symptoms, please do so using the space below.

If you feel you would like to talk to someone about this questionnaire at a later stage, please speak to the doctor or nurse who is looking after you, or be in touch with the research team, whose contact details are on your patient information sheet

Thank you for completing the questionnaire

Please give it to the research nurse

The next page is for the Researcher to complete

57727

6 0

Researcher - Post-completion Sheet

Researcher initials: ☐☐☐

Date questionnaire completed: Date ☐☐ / ☐☐ / ☐☐ Time ☐☐:☐☐

If not completed, give reason ☐

How long is it since the patient was told of his/her diagnosis? ☐

How long did it take the patient to complete the questionnaire?

- Less than 5 minutes ☐
- Between 5 and 10 minutes ☐
- Between 11 and 15 minutes ☐
- Longer than 15 minutes ☐

For the patient selected to <u>complete the questionnaire on their own</u>

Did the patient ask for any help? ☐ Yes ☐ No

If yes,

please specify what help was requested

___

___

please specify what help was given

___

___

For the patient selected for <u>the researcher-administered questionnaire</u>

Did the patient find any of the questions difficult? ☐ Yes ☐ No

If yes,

please specify which questions and what the difficulty was

___

___

___

___

Was the patient made anxious? ☐ Yes ☐ No

If yes,

please give details

___

___

___

___

Where did the patient complete the questionnaire?

(Please specify whether this was in a separate room alone, in a corner of the clinic, the ward or other

___

Thank you. Please now send the questionnaire and patient information sheet and consent form back to Cardiff University in the freepost envelope provided.

57727

PID - | 6 | 1 |

Ymddiriedolaeth GIG Gogledd Orllewin Cymru
North West Wales NHS Trust

NHS WALES GIG CYMRU
Ymddiriedolaeth GIG Gogledd Cymru
North Wales NHS Trust

CID -
- ☐ GLAN CLWYD
- ☐ GWYNEDD
- ☐ MAELOR

Confidential Questionnaire

Looking At Your Symptoms Study

This Study has been funded by Cancer Research UK

Cardiff University / Prifysgol Caerdydd
Ysgol Glinigol Gogledd Cymru / North Wales Clinical School
Cancer Research UK
sewtu South East Wales Trials Unit / Uned Ymchwil De-ddwyrain Cymru

Dear Patient

I am very grateful to you for agreeing to help us with this study.

I hope the information you supply will help us give people a diagnosis earlier.

If you feel you would like to talk to someone about this questionnaire at a later stage, please speak to the doctor or nurse who is looking after you, or be in touch with the research team, whose contact details are on your patient information sheet.

I can assure you that anything you tell us will be treated in the utmost confidence.

Yours sincerely

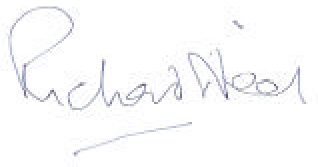

Dr Richard Neal
Senior Clinical Lecturer
Cardiff University
Chief Investigator, Symptoms Study

About your symptoms

These questions are about symptoms you noticed before your diagnosis. We are interested in symptoms which you think are related to your cancer.

Please put a cross in the appropriate box, or write an answer as required.

Looking back...

1. Did you have any symptoms that you feel were due to your cancer before you were diagnosed?

Yes ☐ **Please continue with Question 2**

No ☐ **Please go to Section 2 on Page 5**

2. What was the first symptom that made you think something might be wrong?

☐

Please go to Question 3

The following questions are about when you first noticed a symptom and when you first told your GP or nurse about it.

Please give an exact date if you can. Otherwise give your best estimate. (For example approximately how long ago, the month or season). You wish to refer to your diary or calendar if you have it with you.

Here is an example question:

Decrease in appetite

Did you have this symptom?

Yes ☒ **Please complete A then B or C below**

No ☐ **Please go to Section 2**

A When did you first notice this? d d / m m / y y **OR** Estimate '3 months ago' or 'June'

B When did you first tell your GP or nurse? 2 3 / 1 2 / 0 7 **OR** Estimate

OR

C Put a cross here if you didn't tell your GP or nurse ☐

3. Change in bowel habit

Did you have this symptom?

Yes ☐ **Please complete A then B or C below**

No ☐ **Please go to Question 4**

A When did you first notice this? ☐☐ / ☐☐ / ☐☐ **OR** ☐

B When did you first tell your GP or nurse? ☐☐ / ☐☐ / ☐☐ **OR** ☐

OR

C Put a cross here if you didn't tell your GP or nurse ☐

4. Bleeding from back passage

Did you have this symptom?

Yes ☐ **Please complete A then B or C below**

No ☐ **Please go to Section 2**

A When did you first notice this? ☐☐ / ☐☐ / ☐☐ **OR** ☐

B When did you first tell your GP or nurse? ☐☐ / ☐☐ / ☐☐ **OR** ☐

OR

C Put a cross here if you didn't tell your GP or nurse ☐

Section 2

Please give an exact date if you can. Otherwise give your best estimate (for example approximately how long ago, the month or the season). You may wish to refer to your diary or calendar if you have it with you.

Did you have any of the following?

1. Decrease in appetite

Did you have this?

Yes ☐ **Please complete A then B or C below**

No ☐ **Please go to Question 2**

A When did you first notice this? ☐☐ / ☐☐ / ☐☐ **OR** ☐

B When did you first tell your GP or nurse? ☐☐ / ☐☐ / ☐☐ **OR** ☐

OR

C Put a cross here if you didn't tell your GP or nurse ☐

2. Unexplained weight loss

Did you have this?

Yes ☐ **Please complete A then B or C below**

No ☐ **Please go to Question 3**

A When did you first notice this? ☐☐ / ☐☐ / ☐☐ **OR** ☐

B When did you first tell your GP or nurse? ☐☐ / ☐☐ / ☐☐ **OR** ☐

OR

C Put a cross here if you didn't tell your GP or nurse ☐

3. Fatigue or tiredness that is unusual for you

Did you have this?

Yes ☐ **Please complete A then B or C below**

No ☐ **Please go to Question 4**

A When did you first notice this? __ __ / __ __ / __ __ **OR** ______

B When did you first tell your GP or nurse? __ __ / __ __ / __ __ **OR** ______

OR

C Put a cross here if you didn't tell your GP or nurse ☐

4. Feeling different 'in yourself' from usual

Did you have this?

Yes ☐ **Please complete A then B or C below**

No ☐ **Please go to Question 5**

A When did you first notice this? __ __ / __ __ / __ __ **OR** ______

B When did you first tell your GP or nurse? __ __ / __ __ / __ __ **OR** ______

OR

C Put a cross here if you didn't tell your GP or nurse ☐

Please try and describe what the feeling was

If you had any further symptoms which you feel are relevant to your cancer diagnosis, please list them below. If not, please go to Question 7.

Please describe the symptom here and complete A then B or C below

5.

A When did you first notice this? | _ _ / _ _ / _ _ **OR**

B When did you first tell your GP or nurse? | _ _ / _ _ / _ _ **OR**

OR

C Put a cross here if you didn't tell your GP or nurse ☐

If you had another symptom you feel is relevant, please write it below, otherwise go to Question 7.

Please describe the symptom here and complete A then B or C below

6.

A When did you first notice this? | _ _ / _ _ / _ _ **OR**

B When did you first tell your GP or nurse? | _ _ / _ _ / _ _ **OR**

OR

C Put a cross here if you didn't tell your GP or nurse ☐

If you had further symptoms you would like to tell us about, there is space at the end of the Questionnaire for you to write them.

Did your GP send you for any tests?

Thinking back to the time immediately before you diagnosis, did your GP send you for any of the following tests?

Please cross the appropriate boxes

7. Were you sent for any of these?

	Yes	Not sure	No
Blood test(s)	☐	☐	☐
CT Scan	☐	☐	☐
Ultrasound scan	☐	☐	☐
Barium Enema	☐	☐	☐
Sigmoidoscopy or colonoscopy (Looking at bowel with internal camera)	☐	☐	☐
X-ray	☐	☐	☐

Please go to Question 8

About you

The next questions relate to general information about you. It would help us to know more about the background of the people completing the questionnaire.

8. Which best describes your employment status?

Please cross one box only

- Employed full-time ☐
- Employed part-time ☐
- Self employed full-time ☐
- Self employed part-time ☐
- Unemployed (seeking work) ☐
- Unemployed (not seeking work) ☐
- Retired ☐
- Student ☐
- Permanently sick/disabled ☐
- Temporarily sick/disabled ☐
- Looking after family/home ☐
- Other, please describe ____

9. What is your highest level of qualification?

Please cross one box only

- Degree (or equivalent) ☐
- Diploma (or equivalent) ☐
- 'A' level ☐
- GCSE / 'O' level ☐
- None ☐
- Other, please specify ____

10. **How would you describe your ethnicity**

Please cross one box only

White ☐
or
- White British ☐
- White Irish ☐
- Other White background ☐

Mixed ☐
or
- White & Black Carribean ☐
- White & Black African ☐
- White & Asian ☐
- Other mixed background ☐

Black or Black British ☐
or
- Carribean ☐
- African ☐
- Other Black background ☐

Asian or Asian British ☐
or
- Indian ☐
- Pakistani ☐
- Bangladeshi ☐
- Other Asian background ☐

Chinese or other ethnic group ☐
or
- Chinese ☐
- Other ethnic group ☐

11.

Do you live alone?

Yes ☐ **Please go to Question 12**

No ☐ **Please say who you live with below**

Who do you live with?
(e.g. wife, husband, partner, family member)

☐

12. Are you suffering from, or have you suffered from, any of the following in the past 2 years?

Please cross any that are applicable to you

- Asthma ☐
- Chronic Obstructive Pulmonary Disease (COPD) ☐
- Other lung disease (e.g. fibrosis, bronchiectatis etc) ☐
- Heart disease ☐
- Anxiety or depression ☐
- Inflammatory bowel disease ☐
- Irritable bowel syndrome ☐
- Peptic ulcer ☐
- Previous cancer ☐
- Diabetes ☐
- Arthritis ☐

13. About smoking

Please cross the appropriate statement

- Are you a current smoker? ☐
- Are you an ex-smoker? ☐
- Are you a non-smoker (never smoked)? ☐

14. Do you think you were more at risk of getting cancer because of your family history?

Yes ☐

No ☐

15. **The following questions are about how completing the questionnaire made you feel.**

A number of statements which people have used to describe themselves are given below. Read each statement and then cross the most appropriate box to indicate how you feel right now, at the moment. There are no right or wrong answers. Do not spend too much time on any one statement but give the answer which seems to describe your present feeling best.

		Not at all	Somewhat	Moderately	Very much
1.	I feel calm	☐	☐	☐	☐
2.	I am tense	☐	☐	☐	☐
3.	I feel upset	☐	☐	☐	☐
4.	I am relaxed	☐	☐	☐	☐
5.	I feel content	☐	☐	☐	☐
6.	I am worried	☐	☐	☐	☐

Please make sure you have answered all the questions.

If you have any other comments or there is anything else you would like to tell us about when you first noticed your symptoms, please do so using the space below.

If you feel you would like to talk to someone about this questionnaire at a later stage, please speak to the doctor or nurse who is looking after you, or be in touch with the research team, whose contact details are on your patient information sheet

Thank you for completing the questionnaire

Please give it to the research nurse

The next page is for the Researcher to complete

57727

6 1

Researcher - Post-completion Sheet

Researcher initials: ☐☐☐

Date questionnaire completed: Date ☐☐ / ☐☐ / ☐☐ Time ☐☐:☐☐

If not completed, give reason ☐

How long is it since the patient was told of his/her diagnosis? ☐

How long did it take the patient to complete the questionnaire?

- Less than 5 minutes ☐
- Between 5 and 10 minutes ☐
- Between 11 and 15 minutes ☐
- Longer than 15 minutes ☐

For the patient selected to complete the questionnaire on their own

Did the patient ask for any help? ☐ Yes ☐ No

If yes,

please specify what help was requested

please specify what help was given

For the patient selected for the researcher-administered questionnaire

Did the patient find any of the questions difficult? ☐ Yes ☐ No

If yes,

please specify which questions and what the difficulty was

Was the patient made anxious? ☐ Yes ☐ No

If yes,

please give details

Where did the patient complete the questionnaire?

(Please specify whether this was in a separate room alone, in a corner of the clinic, the ward or other

Thank you. Please now send the questionnaire and patient information sheet and consent form back to Cardiff University in the freepost envelope provided.

57727

PID - 62

Ymddiriedolaeth GIG Gogledd Orllewin Cymru
North West Wales NHS Trust

NHS WALES GIG CYMRU
Ymddiriedolaeth GIG Gogledd Cymru
North Wales NHS Trust

CID -
- ☐ GLAN CLWYD
- ☐ GWYNEDD
- ☐ MAELOR

Confidential Questionnaire

Looking At Your Symptoms Study

This Study has been funded by Cancer Research UK

Cardiff University Prifysgol Caerdydd
Ysgol Glinigol Gogledd Cymru North Wales Clinical School
Cancer Research UK
sewtu South East Wales Trials Unit Uned Ymchwil De-ddwyrain Cymru

Dear Patient

I am very grateful to you for agreeing to help us with this study.

I hope the information you supply will help us give people a diagnosis earlier.

If you feel you would like to talk to someone about this questionnaire at a later stage, please speak to the doctor or nurse who is looking after you, or be in touch with the research team, whose contact details are on your patient information sheet.

I can assure you that anything you tell us will be treated in the utmost confidence.

Yours sincerely

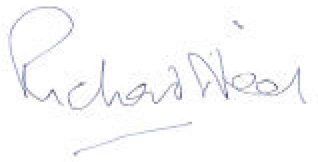

Dr Richard Neal
Senior Clinical Lecturer
Cardiff University
Chief Investigator, Symptoms Study

About your symptoms

These questions are about symptoms you noticed before your diagnosis. We are interested in symptoms which you think are related to your cancer.

Please put a cross in the appropriate box, or write an answer as required.

Looking back...

1. Did you have any symptoms that you feel were due to your cancer before you were diagnosed?

Yes ☐ **Please continue with Question 2**

No ☐ **Please go to Section 2 on Page 5**

2. What was the first symptom that made you think something might be wrong?

[ ]

Please go to Question 3

The following questions are about when you first noticed a symptom and when you first told your GP or nurse about it.

Please give an exact date if you can. Otherwise give your best estimate. (For example approximately how long ago, the month or season). You wish to refer to your diary or calendar if you have it with you.

Here is an example question:

Decrease in appetite

Did you have this symptom?

Yes ☒ **Please complete A then B or C below**

No ☐ **Please go to Section 2**

A When did you first notice this? d d / m m / y y **OR** Estimate '3 months ago' or 'June'

B When did you first tell your GP or nurse? 2 3 / 1 2 / 0 7 **OR** Estimate

OR

C Put a cross here if you didn't tell your GP or nurse ☐

3. Change in bowel habit

Did you have this symptom?

Yes ☐ **Please complete A then B or C below**

No ☐ **Please go to Question 4**

A When did you first notice this? ☐☐ / ☐☐ / ☐☐ **OR** ☐

B When did you first tell your GP or nurse? ☐☐ / ☐☐ / ☐☐ **OR** ☐

OR

C Put a cross here if you didn't tell your GP or nurse ☐

4. Bleeding from back passage

Did you have this symptom?

Yes ☐ **Please complete A then B or C below**

No ☐ **Please go to Section 2**

A When did you first notice this? ☐☐ / ☐☐ / ☐☐ **OR** ☐

B When did you first tell your GP or nurse? ☐☐ / ☐☐ / ☐☐ **OR** ☐

OR

C Put a cross here if you didn't tell your GP or nurse ☐

Please give an exact date if you can. Otherwise give your best estimate (for example approximately how long ago, the month or the season). You may wish to refer to your diary or calendar if you have it with you.

Did you have any of the following?

1. Decrease in appetite

Did you have this?

Yes ☐ **Please complete A then B or C below**

No ☐ **Please go to Question 2**

A When did you first notice this? ☐☐ / ☐☐ / ☐☐ **OR** ☐

B When did you first tell your GP or nurse? ☐☐ / ☐☐ / ☐☐ **OR** ☐

OR

C Put a cross here if you didn't tell your GP or nurse ☐

2. Unexplained weight loss

Did you have this?

Yes ☐ **Please complete A then B or C below**

No ☐ **Please go to Question 3**

A When did you first notice this? ☐☐ / ☐☐ / ☐☐ **OR** ☐

B When did you first tell your GP or nurse? ☐☐ / ☐☐ / ☐☐ **OR** ☐

OR

C Put a cross here if you didn't tell your GP or nurse ☐

3. Fatigue or tiredness that is unusual for you

Did you have this?

Yes ☐ **Please complete A then B or C below**

No ☐ **Please go to Question 4**

A When did you first notice this? ☐☐ / ☐☐ / ☐☐ **OR** ☐

B When did you first tell your GP or nurse? ☐☐ / ☐☐ / ☐☐ **OR** ☐

OR

C Put a cross here if you didn't tell your GP or nurse ☐

4. Feeling different 'in yourself' from usual

Did you have this?

Yes ☐ **Please complete A then B or C below**

No ☐ **Please go to Question 5**

A When did you first notice this? ☐☐ / ☐☐ / ☐☐ **OR** ☐

B When did you first tell your GP or nurse? ☐☐ / ☐☐ / ☐☐ **OR** ☐

OR

C Put a cross here if you didn't tell your GP or nurse ☐

Please try and describe what the feeling was

If you had any further symptoms which you feel are relevant to your cancer diagnosis, please list them below. If not, please go to Question 7.

Please describe the symptom here and complete A then B or C below

5.

A When did you <u>first notice</u> this? __ __ / __ __ / __ __ OR

B When did you <u>first tell</u> your GP or nurse? __ __ / __ __ / __ __ OR

OR

C Put a cross here if you <u>didn't tell</u> your GP or nurse ☐

If you had another symptom you feel is relevant, please write it below, otherwise go to Question 7.

Please describe the symptom here and complete A then B or C below

6.

A When did you <u>first notice</u> this? __ __ / __ __ / __ __ OR

B When did you <u>first tell</u> your GP or nurse? __ __ / __ __ / __ __ OR

OR

C Put a cross here if you <u>didn't tell</u> your GP or nurse ☐

If you had further symptoms you would like to tell us about, there is space at the end of the Questionnaire for you to write them.

Did your GP send you for any tests?

Thinking back to the time immediately before you diagnosis, did your GP send you for any of the following tests?

Please cross the appropriate boxes

7. Were you sent for any of these?

	Yes	Not sure	No
Blood test(s)	☐	☐	☐
CT Scan	☐	☐	☐
Ultrasound scan	☐	☐	☐
Barium Enema	☐	☐	☐
Sigmoidoscopy or colonoscopy (Looking at bowel with internal camera)	☐	☐	☐
X-ray	☐	☐	☐

Please go to Question 8

About you

The next questions relate to general information about you. It would help us to know more about the background of the people completing the questionnaire.

8. Which best describes your employment status?

Please cross one box only

- Employed full-time ☐
- Employed part-time ☐
- Self employed full-time ☐
- Self employed part-time ☐
- Unemployed (seeking work) ☐
- Unemployed (not seeking work) ☐
- Retired ☐
- Student ☐
- Permanently sick/disabled ☐
- Temporarily sick/disabled ☐
- Looking after family/home ☐
- Other, please describe ______

9. What is your highest level of qualification?

Please cross one box only

- Degree (or equivalent) ☐
- Diploma (or equivalent) ☐
- 'A' level ☐
- GCSE / 'O' level ☐
- None ☐
- Other, please specify ______

10. How would you describe your ethnicity

Please cross one box only

White ☐
or
- White British ☐
- White Irish ☐
- Other White background ☐

Mixed ☐
or
- White & Black Carribean ☐
- White & Black African ☐
- White & Asian ☐
- Other mixed background ☐

Black or Black British ☐
or
- Carribean ☐
- African ☐
- Other Black background ☐

Asian or Asian British ☐
or
- Indian ☐
- Pakistani ☐
- Bangladeshi ☐
- Other Asian background ☐

Chinese or other ethnic group ☐
or
- Chinese ☐
- Other ethnic group ☐

11.

Do you live alone?

Yes ☐ **Please go to Question 12**

No ☐ **Please say who you live with below**

Who do you live with?
(e.g. wife, husband, partner, family member)

☐

12. **Are you suffering from, or have you suffered from, any of the following in the past 2 years?**

Please cross any that are applicable to you

- Asthma ☐
- Chronic Obstructive Pulmonary Disease (COPD) ☐
- Other lung disease (e.g. fibrosis, bronchiectatis etc) ☐
- Heart disease ☐
- Anxiety or depression ☐
- Inflammatory bowel disease ☐
- Irritable bowel syndrome ☐
- Peptic ulcer ☐
- Previous cancer ☐
- Diabetes ☐
- Arthritis ☐

13. **About smoking**

Please cross the appropriate statement

- Are you a current smoker? ☐
- Are you an ex-smoker? ☐
- Are you a non-smoker (never smoked)? ☐

14. **Do you think you were more at risk of getting cancer because of your family history?**

Yes ☐

No ☐

15. The following questions are about how completing the questionnaire made you feel.

A number of statements which people have used to describe themselves are given below. Read each statement and then cross the most appropriate box to indicate how you feel <u>right now, at the moment.</u> There are no right or wrong answers. Do not spend too much time on any one statement but give the answer which seems to describe your present feeling best.

		Not at all	Somewhat	Moderately	Very much
1.	I feel calm	☐	☐	☐	☐
2.	I am tense	☐	☐	☐	☐
3.	I feel upset	☐	☐	☐	☐
4.	I am relaxed	☐	☐	☐	☐
5.	I feel content	☐	☐	☐	☐
6.	I am worried	☐	☐	☐	☐

Please make sure you have answered all the questions.

If you have any other comments or there is anything else you would like to tell us about when you first noticed your symptoms, please do so using the space below.

If you feel you would like to talk to someone about this questionnaire at a later stage, please speak to the doctor or nurse who is looking after you, or be in touch with the research team, whose contact details are on your patient information sheet

Thank you for completing the questionnaire

Please give it to the research nurse

The next page is for the Researcher to complete

57727

62

Researcher - Post-completion Sheet

Researcher initials: ☐☐☐

Date questionnaire completed: Date ☐☐ / ☐☐ / ☐☐ Time ☐☐:☐☐

If not completed, give reason ☐

How long is it since the patient was told of his/her diagnosis? ☐

How long did it take the patient to complete the questionnaire?

- Less than 5 minutes ☐
- Between 5 and 10 minutes ☐
- Between 11 and 15 minutes ☐
- Longer than 15 minutes ☐

For the patient selected to <u>complete the questionnaire on their own</u>

Did the patient ask for any help? ☐ Yes ☐ No

If yes,

please specify what help was requested

____

____

please specify what help was given

____

____

For the patient selected for <u>the researcher-administered questionnaire</u>

Did the patient find any of the questions difficult? ☐ Yes ☐ No

If yes,

please specify which questions and what the difficulty was

____

____

____

____

Was the patient made anxious? ☐ Yes ☐ No

If yes,

please give details

____

____

____

____

Where did the patient complete the questionnaire?
(Please specify whether this was in a separate room alone, in a corner of the clinic, the ward or other

____

Thank you. Please now send the questionnaire and patient information sheet and consent form back to Cardiff University in the freepost envelope provided.

57727

PID - 63

Ymddiriedolaeth GIG Gogledd Orllewin Cymru
North West Wales NHS Trust

NHS Wales GIG Cymru
Ymddiriedolaeth GIG Gogledd Cymru
North Wales NHS Trust

☐ GLAN CLWYD

CID - ☐ GWYNEDD

☐ MAELOR

Confidential Questionnaire

Looking At Your Symptoms Study

This Study has been funded by Cancer Research UK

Cardiff University / Prifysgol Caerdydd
Ysgol Glinigol Gogledd Cymru / North Wales Clinical School
Cancer Research UK
sewtu South East Wales Trials Unit / Uned Ymchwil De-ddwyrain Cymru

Dear Patient

I am very grateful to you for agreeing to help us with this study.

I hope the information you supply will help us give people a diagnosis earlier.

If you feel you would like to talk to someone about this questionnaire at a later stage, please speak to the doctor or nurse who is looking after you, or be in touch with the research team, whose contact details are on your patient information sheet.

I can assure you that anything you tell us will be treated in the utmost confidence.

Yours sincerely

Richard Neal

Dr Richard Neal
Senior Clinical Lecturer
Cardiff University
Chief Investigator, Symptoms Study

About your symptoms

These questions are about symptoms you noticed before your diagnosis. We are interested in symptoms which you think are related to your cancer.

Please put a cross in the appropriate box, or write an answer as required.

Looking back...

1. Did you have any symptoms that you feel were due to your cancer before you were diagnosed?

Yes ☐ **Please continue with Question 2**

No ☐ **Please go to Section 2 on Page 5**

2. What was the first symptom that made you think something might be wrong?

☐

Please go to Question 3

The following questions are about when you first noticed a symptom and when you first told your GP or nurse about it.

Please give an exact date if you can. Otherwise give your best estimate. (For example approximately how long ago, the month or season). You wish to refer to your diary or calendar if you have it with you.

Here is an example question:

Decrease in appetite

Did you have this symptom?

Yes ☒ **Please complete A then B or C below**

No ☐ **Please go to Section 2**

A When did you first notice this? d d / m m / y y **OR** Estimate '3 months ago' or 'June'

B When did you first tell your GP or nurse? 2 3 / 1 2 / 0 7 **OR** Estimate

OR

C Put a cross here if you didn't tell your GP or nurse ☐

3. Change in bowel habit

Did you have this symptom?

Yes ☐ **Please complete A then B or C below**

No ☐ **Please go to Question 4**

A When did you first notice this? __ __ / __ __ / __ __ **OR** ______

B When did you first tell your GP or nurse? __ __ / __ __ / __ __ **OR** ______

OR

C Put a cross here if you didn't tell your GP or nurse ☐

4. Bleeding from back passage

Did you have this symptom?

Yes ☐ **Please complete A then B or C below**

No ☐ **Please go to Section 2**

A When did you first notice this? __ __ / __ __ / __ __ **OR** ______

B When did you first tell your GP or nurse? __ __ / __ __ / __ __ **OR** ______

OR

C Put a cross here if you didn't tell your GP or nurse ☐

Section 2

Please give an exact date if you can. Otherwise give your best estimate (for example approximately how long ago, the month or the season). You may wish to refer to your diary or calendar if you have it with you.

Did you have any of the following?

1. Decrease in appetite

Did you have this?

Yes ☐ **Please complete A then B or C below**

No ☐ **Please go to Question 2**

A When did you first notice this? ☐☐ / ☐☐ / ☐☐ **OR** ☐

B When did you first tell your GP or nurse? ☐☐ / ☐☐ / ☐☐ **OR** ☐

OR

C Put a cross here if you didn't tell your GP or nurse ☐

2. Unexplained weight loss

Did you have this?

Yes ☐ **Please complete A then B or C below**

No ☐ **Please go to Question 3**

A When did you first notice this? ☐☐ / ☐☐ / ☐☐ **OR** ☐

B When did you first tell your GP or nurse? ☐☐ / ☐☐ / ☐☐ **OR** ☐

OR

C Put a cross here if you didn't tell your GP or nurse ☐

3. Fatigue or tiredness that is unusual for you

Did you have this?

Yes ☐ **Please complete A then B or C below**

No ☐ **Please go to Question 4**

A When did you first notice this? ☐☐ / ☐☐ / ☐☐ **OR** ☐

B When did you first tell your GP or nurse? ☐☐ / ☐☐ / ☐☐ **OR** ☐

OR

C Put a cross here if you didn't tell your GP or nurse ☐

4. Feeling different 'in yourself' from usual

Did you have this?

Yes ☐ **Please complete A then B or C below**

No ☐ **Please go to Question 5**

A When did you first notice this? ☐☐ / ☐☐ / ☐☐ **OR** ☐

B When did you first tell your GP or nurse? ☐☐ / ☐☐ / ☐☐ **OR** ☐

OR

C Put a cross here if you didn't tell your GP or nurse ☐

Please try and describe what the feeling was

If you had any further symptoms which you feel are relevant to your cancer diagnosis, please list them below. If not, please go to Question 7.

Please describe the symptom here and complete A then B or C below

5.

A When did you first notice this? __/__/__ **OR**

B When did you first tell your GP or nurse? __/__/__ **OR**

OR

C Put a cross here if you didn't tell your GP or nurse ☐

If you had another symptom you feel is relevant, please write it below, otherwise go to Question 7.

Please describe the symptom here and complete A then B or C below

6.

A When did you first notice this? __/__/__ **OR**

B When did you first tell your GP or nurse? __/__/__ **OR**

OR

C Put a cross here if you didn't tell your GP or nurse ☐

If you had further symptoms you would like to tell us about, there is space at the end of the Questionnaire for you to write them.

Did your GP send you for any tests?

Thinking back to the time immediately before you diagnosis, did your GP send you for any of the following tests?

Please cross the appropriate boxes

7. Were you sent for any of these?

	Yes	Not sure	No
Blood test(s)	☐	☐	☐
CT Scan	☐	☐	☐
Ultrasound scan	☐	☐	☐
Barium Enema	☐	☐	☐
Sigmoidoscopy or colonoscopy (Looking at bowel with internal camera)	☐	☐	☐
X-ray	☐	☐	☐

Please go to Question 8

About you

The next questions relate to general information about you. It would help us to know more about the background of the people completing the questionnaire.

8. Which best describes your employment status?

Please cross one box only

- Employed full-time ☐
- Employed part-time ☐
- Self employed full-time ☐
- Self employed part-time ☐
- Unemployed (seeking work) ☐
- Unemployed (not seeking work) ☐
- Retired ☐
- Student ☐
- Permanently sick/disabled ☐
- Temporarily sick/disabled ☐
- Looking after family/home ☐
- Other, please describe ________

9. What is your highest level of qualification?

Please cross one box only

- Degree (or equivalent) ☐
- Diploma (or equivalent) ☐
- 'A' level ☐
- GCSE / 'O' level ☐
- None ☐
- Other, please specify ________

10. **How would you describe your ethnicity**

Please cross one box only

White ☐
or
- White British ☐
- White Irish ☐
- Other White background ☐

Mixed ☐
or
- White & Black Carribean ☐
- White & Black African ☐
- White & Asian ☐
- Other mixed background ☐

Black or Black British ☐
or
- Carribean ☐
- African ☐
- Other Black background ☐

Asian or Asian British ☐
or
- Indian ☐
- Pakistani ☐
- Bangladeshi ☐
- Other Asian background ☐

Chinese or other ethnic group ☐
or
- Chinese ☐
- Other ethnic group ☐

11.

Do you live alone?

Yes ☐ **Please go to Question 12**

No ☐ **Please say who you live with below**

Who do you live with?
(e.g. wife, husband, partner, family member)

12. Are you suffering from, or have you suffered from, any of the following in the past 2 years?

Please cross any that are applicable to you

- Asthma ☐
- Chronic Obstructive Pulmonary Disease (COPD) ☐
- Other lung disease (e.g. fibrosis, bronchiectatis etc) ☐
- Heart disease ☐
- Anxiety or depression ☐
- Inflammatory bowel disease ☐
- Irritable bowel syndrome ☐
- Peptic ulcer ☐
- Previous cancer ☐
- Diabetes ☐
- Arthritis ☐

13. About smoking

Please cross the appropriate statement

- Are you a current smoker? ☐
- Are you an ex-smoker? ☐
- Are you a non-smoker (never smoked)? ☐

14. Do you think you were more at risk of getting cancer because of your family history?

Yes ☐

No ☐

15. The following questions are about how completing the questionnaire made you feel.

A number of statements which people have used to describe themselves are given below. Read each statement and then cross the most appropriate box to indicate how you feel right now, at the moment. There are no right or wrong answers. Do not spend too much time on any one statement but give the answer which seems to describe your present feeling best.

		Not at all	Somewhat	Moderately	Very much
1.	I feel calm	☐	☐	☐	☐
2.	I am tense	☐	☐	☐	☐
3.	I feel upset	☐	☐	☐	☐
4.	I am relaxed	☐	☐	☐	☐
5.	I feel content	☐	☐	☐	☐
6.	I am worried	☐	☐	☐	☐

Please make sure you have answered all the questions.

If you have any other comments or there is anything else you would like to tell us about when you first noticed your symptoms, please do so using the space below.

If you feel you would like to talk to someone about this questionnaire at a later stage, please speak to the doctor or nurse who is looking after you, or be in touch with the research team, whose contact details are on your patient information sheet

Thank you for completing the questionnaire

Please give it to the research nurse

The next page is for the Researcher to complete

Researcher - Post-completion Sheet

Researcher initials: ☐☐☐

Date questionnaire completed: Date ☐☐ / ☐☐ / ☐☐ Time ☐☐:☐☐

If not completed, give reason ☐

How long is it since the patient was told of his/her diagnosis? ☐

How long did it take the patient to complete the questionnaire?

- Less than 5 minutes ☐
- Between 5 and 10 minutes ☐
- Between 11 and 15 minutes ☐
- Longer than 15 minutes ☐

For the patient selected to <u>complete the questionnaire on their own</u>

Did the patient ask for any help? ☐ Yes ☐ No

If yes,

please specify what help was requested

____

____

please specify what help was given

____

____

For the patient selected for <u>the researcher-administered questionnaire</u>

Did the patient find any of the questions difficult? ☐ Yes ☐ No

If yes,

please specify which questions and what the difficulty was

____

____

____

____

Was the patient made anxious? ☐ Yes ☐ No

If yes,

please give details

____

____

____

____

Where did the patient complete the questionnaire?
(Please specify whether this was in a separate room alone, in a corner of the clinic, the ward or other

____

Thank you. Please now send the questionnaire and patient information sheet and consent form back to Cardiff University in the freepost envelope provided.

57727

PID - | 6 | 4 |

Ymddiriedolaeth GIG Gogledd Orllewin Cymru
North West Wales NHS Trust

NHS WALES GIG CYMRU
Ymddiriedolaeth GIG Gogledd Cymru
North Wales NHS Trust

CID -
☐ GLAN CLWYD
☐ GWYNEDD
☐ MAELOR

Confidential Questionnaire

Looking At Your Symptoms Study

This Study has been funded by Cancer Research UK

Cardiff University / Prifysgol Caerdydd
Ysgol Glinigol Gogledd Cymru / North Wales Clinical School
Cancer Research UK
sewtu South East Wales Trials Unit / Uned Ymchwil De-ddwyrain Cymru

Dear Patient

I am very grateful to you for agreeing to help us with this study.

I hope the information you supply will help us give people a diagnosis earlier.

If you feel you would like to talk to someone about this questionnaire at a later stage, please speak to the doctor or nurse who is looking after you, or be in touch with the research team, whose contact details are on your patient information sheet.

I can assure you that anything you tell us will be treated in the utmost confidence.

Yours sincerely

Richard Neal

Dr Richard Neal
Senior Clinical Lecturer
Cardiff University
Chief Investigator, Symptoms Study

About your symptoms

These questions are about symptoms you noticed before your diagnosis. We are interested in symptoms which you think are related to your cancer.

Please put a cross in the appropriate box, or write an answer as required.

Looking back...

1. Did you have any symptoms that you feel were due to your cancer before you were diagnosed?

Yes ☐ **Please continue with Question 2**

No ☐ **Please go to Section 2 on Page 5**

2. What was the first symptom that made you think something might be wrong?

[ ]

Please go to Question 3

The following questions are about when you first noticed a symptom and when you first told your GP or nurse about it.

Please give an exact date if you can. Otherwise give your best estimate. (For example approximately how long ago, the month or season). You wish to refer to your diary or calendar if you have it with you.

Here is an example question:

Decrease in appetite

Did you have this symptom?

Yes ☒ **Please complete A then B or C below**

No ☐ **Please go to Section 2**

A When did you first notice this? d d / m m / y y **OR** Estimate '3 months ago' or 'June'

B When did you first tell your GP or nurse? 2 3 / 1 2 / 0 7 **OR** Estimate

OR

C Put a cross here if you didn't tell your GP or nurse ☐

3. Change in bowel habit

Did you have this symptom?

Yes ☐ **Please complete A then B or C below**

No ☐ **Please go to Question 4**

A When did you first notice this? ☐☐ / ☐☐ / ☐☐ **OR** ☐

B When did you first tell your GP or nurse? ☐☐ / ☐☐ / ☐☐ **OR** ☐

OR

C Put a cross here if you didn't tell your GP or nurse ☐

4. Bleeding from back passage

Did you have this symptom?

Yes ☐ **Please complete A then B or C below**

No ☐ **Please go to Section 2**

A When did you first notice this? ☐☐ / ☐☐ / ☐☐ **OR** ☐

B When did you first tell your GP or nurse? ☐☐ / ☐☐ / ☐☐ **OR** ☐

OR

C Put a cross here if you didn't tell your GP or nurse ☐

Section 2

Please give an exact date if you can. Otherwise give your best estimate (for example approximately how long ago, the month or the season). You may wish to refer to your diary or calendar if you have it with you.

Did you have any of the following?

1. Decrease in appetite

Did you have this?

Yes ☐ **Please complete A then B or C below**

No ☐ **Please go to Question 2**

A When did you first notice this? ☐☐ / ☐☐ / ☐☐ **OR** ☐

B When did you first tell your GP or nurse? ☐☐ / ☐☐ / ☐☐ **OR** ☐

OR

C Put a cross here if you didn't tell your GP or nurse ☐

2. Unexplained weight loss

Did you have this?

Yes ☐ **Please complete A then B or C below**

No ☐ **Please go to Question 3**

A When did you first notice this? ☐☐ / ☐☐ / ☐☐ **OR** ☐

B When did you first tell your GP or nurse? ☐☐ / ☐☐ / ☐☐ **OR** ☐

OR

C Put a cross here if you didn't tell your GP or nurse ☐

3. **Fatigue or tiredness that is unusual for you**

Did you have this?

Yes ☐ **Please complete A then B or C below**

No ☐ **Please go to Question 4**

A When did you first notice this? ☐☐ / ☐☐ / ☐☐ **OR** ☐

B When did you first tell your GP or nurse? ☐☐ / ☐☐ / ☐☐ **OR** ☐

OR

C Put a cross here if you didn't tell your GP or nurse ☐

4. **Feeling different 'in yourself' from usual**

Did you have this?

Yes ☐ **Please complete A then B or C below**

No ☐ **Please go to Question 5**

A When did you first notice this? ☐☐ / ☐☐ / ☐☐ **OR** ☐

B When did you first tell your GP or nurse? ☐☐ / ☐☐ / ☐☐ **OR** ☐

OR

C Put a cross here if you didn't tell your GP or nurse ☐

Please try and describe what the feeling was

If you had any further symptoms which you feel are relevant to your cancer diagnosis, please list them below. If not, please go to Question 7.

Please describe the symptom here and complete A then B or C below

5.

A When did you first notice this? ☐☐ / ☐☐ / ☐☐ OR

B When did you first tell your GP or nurse? ☐☐ / ☐☐ / ☐☐ OR

OR

C Put a cross here if you didn't tell your GP or nurse ☐

If you had another symptom you feel is relevant, please write it below, otherwise go to Question 7.

Please describe the symptom here and complete A then B or C below

6.

A When did you first notice this? ☐☐ / ☐☐ / ☐☐ OR

B When did you first tell your GP or nurse? ☐☐ / ☐☐ / ☐☐ OR

OR

C Put a cross here if you didn't tell your GP or nurse ☐

If you had further symptoms you would like to tell us about, there is space at the end of the Questionnaire for you to write them.

Did your GP send you for any tests?

Thinking back to the time immediately before you diagnosis, did your GP send you for any of the following tests?

Please cross the appropriate boxes

7. **Were you sent for any of these?**

	Yes	Not sure	No
Blood test(s)	☐	☐	☐
CT Scan	☐	☐	☐
Ultrasound scan	☐	☐	☐
Barium Enema	☐	☐	☐
Sigmoidoscopy or colonoscopy (Looking at bowel with internal camera)	☐	☐	☐
X-ray	☐	☐	☐

Please go to Question 8

About you

The next questions relate to general information about you. It would help us to know more about the background of the people completing the questionnaire.

8. Which best describes your employment status?

Please cross one box only

- Employed full-time ☐
- Employed part-time ☐
- Self employed full-time ☐
- Self employed part-time ☐
- Unemployed (seeking work) ☐
- Unemployed (not seeking work) ☐
- Retired ☐
- Student ☐
- Permanently sick/disabled ☐
- Temporarily sick/disabled ☐
- Looking after family/home ☐
- Other, please describe ____

9. What is your highest level of qualification?

Please cross one box only

- Degree (or equivalent) ☐
- Diploma (or equivalent) ☐
- 'A' level ☐
- GCSE / 'O' level ☐
- None ☐
- Other, please specify ____

10. How would you describe your ethnicity

Please cross one box only

White ☐
or
- White British ☐
- White Irish ☐
- Other White background ☐

Mixed ☐
or
- White & Black Carribean ☐
- White & Black African ☐
- White & Asian ☐
- Other mixed background ☐

Black or Black British ☐
or
- Carribean ☐
- African ☐
- Other Black background ☐

Asian or Asian British ☐
or
- Indian ☐
- Pakistani ☐
- Bangladeshi ☐
- Other Asian background ☐

Chinese or other ethnic group ☐
or
- Chinese ☐
- Other ethnic group ☐

11.

Do you live alone?

Yes ☐ **Please go to Question 12**

No ☐ **Please say who you live with below**

Who do you live with?
(e.g. wife, husband, partner, family member)

☐

12. Are you suffering from, or have you suffered from, any of the following in the past 2 years?

Please cross any that are applicable to you

- Asthma ☐
- Chronic Obstructive Pulmonary Disease (COPD) ☐
- Other lung disease (e.g. fibrosis, bronchiectatis etc) ☐
- Heart disease ☐
- Anxiety or depression ☐
- Inflammatory bowel disease ☐
- Irritable bowel syndrome ☐
- Peptic ulcer ☐
- Previous cancer ☐
- Diabetes ☐
- Arthritis ☐

13. About smoking

Please cross the appropriate statement

- Are you a current smoker? ☐
- Are you an ex-smoker? ☐
- Are you a non-smoker (never smoked)? ☐

14. Do you think you were more at risk of getting cancer because of your family history?

Yes ☐
No ☐

15. **The following questions are about how completing the questionnaire made you feel.**

A number of statements which people have used to describe themselves are given below. Read each statement and then cross the most appropriate box to indicate how you feel right now, at the moment. There are no right or wrong answers. Do not spend too much time on any one statement but give the answer which seems to describe your present feeling best.

		Not at all	Somewhat	Moderately	Very much
1.	I feel calm	☐	☐	☐	☐
2.	I am tense	☐	☐	☐	☐
3.	I feel upset	☐	☐	☐	☐
4.	I am relaxed	☐	☐	☐	☐
5.	I feel content	☐	☐	☐	☐
6.	I am worried	☐	☐	☐	☐

Please make sure you have answered all the questions.

If you have any other comments or there is anything else you would like to tell us about when you first noticed your symptoms, please do so using the space below.

If you feel you would like to talk to someone about this questionnaire at a later stage, please speak to the doctor or nurse who is looking after you, or be in touch with the research team, whose contact details are on your patient information sheet

Thank you for completing the questionnaire

Please give it to the research nurse

The next page is for the Researcher to complete

Researcher - Post-completion Sheet

Researcher initials: ☐☐☐

Date questionnaire completed: Date ☐☐ / ☐☐ / ☐☐ Time ☐☐ : ☐☐

If not completed, give reason ☐

How long is it since the patient was told of his/her diagnosis? ☐

How long did it take the patient to complete the questionnaire?

- Less than 5 minutes ☐
- Between 5 and 10 minutes ☐
- Between 11 and 15 minutes ☐
- Longer than 15 minutes ☐

For the patient selected to complete the questionnaire on their own

Did the patient ask for any help? ☐ Yes ☐ No

If yes,

please specify what help was requested

____

____

please specify what help was given

____

____

For the patient selected for the researcher-administered questionnaire

Did the patient find any of the questions difficult? ☐ Yes ☐ No

If yes,

please specify which questions and what the difficulty was

____

____

____

____

Was the patient made anxious? ☐ Yes ☐ No

If yes,

please give details

____

____

____

____

Where did the patient complete the questionnaire?
(Please specify whether this was in a separate room alone, in a corner of the clinic, the ward or other

____

Thank you. Please now send the questionnaire and patient information sheet and consent form back to Cardiff University in the freepost envelope provided.

57727

PID - | 6 | 5

Ymddiriedolaeth GIG Gogledd Orllewin Cymru
North West Wales NHS Trust

NHS WALES GIG CYMRU
Ymddiriedolaeth GIG Gogledd Cymru
North Wales NHS Trust

CID -
- ☐ GLAN CLWYD
- ☐ GWYNEDD
- ☐ MAELOR

Confidential Questionnaire

Looking At Your Symptoms Study

This Study has been funded by Cancer Research UK

Cardiff University Prifysgol Caerdydd
Ysgol Glinigol Gogledd Cymru North Wales Clinical School
Cancer Research UK
sewtu South East Wales Trials Unit Uned Ymchwil De-ddwyrain Cymru

Dear Patient

I am very grateful to you for agreeing to help us with this study.

I hope the information you supply will help us give people a diagnosis earlier.

If you feel you would like to talk to someone about this questionnaire at a later stage, please speak to the doctor or nurse who is looking after you, or be in touch with the research team, whose contact details are on your patient information sheet.

I can assure you that anything you tell us will be treated in the utmost confidence.

Yours sincerely

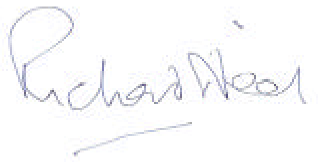

Dr Richard Neal
Senior Clinical Lecturer
Cardiff University
Chief Investigator, Symptoms Study

About your symptoms

These questions are about symptoms you noticed before your diagnosis. We are interested in symptoms which you think are related to your cancer.

Please put a cross in the appropriate box, or write an answer as required.

Looking back...

1. Did you have any symptoms that you feel were due to your cancer before you were diagnosed?

Yes ☐ **Please continue with Question 2**

No ☐ **Please go to Section 2 on Page 5**

2. What was the first symptom that made you think something might be wrong?

[ ]

Please go to Question 3

The following questions are about when you first noticed a symptom and when you first told your GP or nurse about it.

Please give an exact date if you can. Otherwise give your best estimate. (For example approximately how long ago, the month or season). You wish to refer to your diary or calendar if you have it with you.

Here is an example question:

Decrease in appetite

Did you have this symptom?

Yes ☒ **Please complete A then B or C below**

No ☐ **Please go to Section 2**

A When did you first notice this? d d / m m / y y **OR** Estimate: '3 months ago' or 'June'

B When did you first tell your GP or nurse? 2 3 / 1 2 / 0 7 **OR** Estimate

OR

C Put a cross here if you didn't tell your GP or nurse ☐

3. Change in bowel habit

Did you have this symptom?

Yes ☐ **Please complete A then B or C below**

No ☐ **Please go to Question 4**

A When did you <u>first notice</u> this? ☐☐ / ☐☐ / ☐☐ **OR** ☐

B When did you <u>first tell</u> your GP or nurse? ☐☐ / ☐☐ / ☐☐ **OR** ☐

OR

C Put a cross here if you <u>didn't tell</u> your GP or nurse ☐

4. Bleeding from back passage

Did you have this symptom?

Yes ☐ **Please complete A then B or C below**

No ☐ **Please go to Section 2**

A When did you <u>first notice</u> this? ☐☐ / ☐☐ / ☐☐ **OR** ☐

B When did you <u>first tell</u> your GP or nurse? ☐☐ / ☐☐ / ☐☐ **OR** ☐

OR

C Put a cross here if you <u>didn't tell</u> your GP or nurse ☐

Section 2

Please give an exact date if you can. Otherwise give your best estimate (for example approximately how long ago, the month or the season). You may wish to refer to your diary or calendar if you have it with you.

Did you have any of the following?

1. Decrease in appetite

Did you have this?

Yes ☐ **Please complete A then B or C below**

No ☐ **Please go to Question 2**

A When did you first notice this? ☐☐ / ☐☐ / ☐☐ **OR** ☐

B When did you first tell your GP or nurse? ☐☐ / ☐☐ / ☐☐ **OR** ☐

OR

C Put a cross here if you didn't tell your GP or nurse ☐

2. Unexplained weight loss

Did you have this?

Yes ☐ **Please complete A then B or C below**

No ☐ **Please go to Question 3**

A When did you first notice this? ☐☐ / ☐☐ / ☐☐ **OR** ☐

B When did you first tell your GP or nurse? ☐☐ / ☐☐ / ☐☐ **OR** ☐

OR

C Put a cross here if you didn't tell your GP or nurse ☐

3. **Fatigue or tiredness that is unusual for you**

Did you have this?

Yes ☐ **Please complete A then B or C below**

No ☐ **Please go to Question 4**

A When did you first notice this? ☐☐ / ☐☐ / ☐☐ **OR** ☐

B When did you first tell your GP or nurse? ☐☐ / ☐☐ / ☐☐ **OR** ☐

OR

C Put a cross here if you didn't tell your GP or nurse ☐

4. **Feeling different 'in yourself' from usual**

Did you have this?

Yes ☐ **Please complete A then B or C below**

No ☐ **Please go to Question 5**

A When did you first notice this? ☐☐ / ☐☐ / ☐☐ **OR** ☐

B When did you first tell your GP or nurse? ☐☐ / ☐☐ / ☐☐ **OR** ☐

OR

C Put a cross here if you didn't tell your GP or nurse ☐

Please try and describe what the feeling was

If you had any further symptoms which you feel are relevant to your cancer diagnosis, please list them below. If not, please go to Question 7.

Please describe the symptom here and complete A then B or C below

5.

**A**	When did you first notice this?	__ __ / __ __ / __ __ **OR**
**B**	When did you first tell your GP or nurse?	__ __ / __ __ / __ __ **OR**
	**OR**	
**C**	Put a cross here if you didn't tell your GP or nurse	☐

If you had another symptom you feel is relevant, please write it below, otherwise go to Question 7.

Please describe the symptom here and complete A then B or C below

6.

**A**	When did you first notice this?	__ __ / __ __ / __ __ **OR**
**B**	When did you first tell your GP or nurse?	__ __ / __ __ / __ __ **OR**
	**OR**	
**C**	Put a cross here if you didn't tell your GP or nurse	☐

If you had further symptoms you would like to tell us about, there is space at the end of the Questionnaire for you to write them.

Did your GP send you for any tests?

Thinking back to the time immediately before you diagnosis, did your GP send you for any of the following tests?

Please cross the appropriate boxes

7. Were you sent for any of these?

	Yes	Not sure	No
Blood test(s)	☐	☐	☐
CT Scan	☐	☐	☐
Ultrasound scan	☐	☐	☐
Barium Enema	☐	☐	☐
Sigmoidoscopy or colonoscopy (Looking at bowel with internal camera)	☐	☐	☐
X-ray	☐	☐	☐

Please go to Question 8

About you

The next questions relate to general information about you. It would help us to know more about the background of the people completing the questionnaire.

8. Which best describes your employment status?

Please cross one box only

- Employed full-time ☐
- Employed part-time ☐
- Self employed full-time ☐
- Self employed part-time ☐
- Unemployed (seeking work) ☐
- Unemployed (not seeking work) ☐
- Retired ☐
- Student ☐
- Permanently sick/disabled ☐
- Temporarily sick/disabled ☐
- Looking after family/home ☐
- Other, please describe

9. What is your highest level of qualification?

Please cross one box only

- Degree (or equivalent) ☐
- Diploma (or equivalent) ☐
- 'A' level ☐
- GCSE / 'O' level ☐
- None ☐
- Other, please specify

10. How would you describe your ethnicity

Please cross one box only

White ☐
or
- White British ☐
- White Irish ☐
- Other White background ☐

Mixed ☐
or
- White & Black Carribean ☐
- White & Black African ☐
- White & Asian ☐
- Other mixed background ☐

Black or Black British ☐
or
- Carribean ☐
- African ☐
- Other Black background ☐

Asian or Asian British ☐
or
- Indian ☐
- Pakistani ☐
- Bangladeshi ☐
- Other Asian background ☐

Chinese or other ethnic group ☐
or
- Chinese ☐
- Other ethnic group ☐

11.

Do you live alone?

Yes ☐ **Please go to Question 12**

No ☐ **Please say who you live with below**

Who do you live with?
(e.g. wife, husband, partner, family member)

12. **Are you suffering from, or have you suffered from, any of the following in the past 2 years?**

Please cross any that are applicable to you

- Asthma ☐
- Chronic Obstructive Pulmonary Disease (COPD) ☐
- Other lung disease (e.g. fibrosis, bronchiectatis etc) ☐
- Heart disease ☐
- Anxiety or depression ☐
- Inflammatory bowel disease ☐
- Irritable bowel syndrome ☐
- Peptic ulcer ☐
- Previous cancer ☐
- Diabetes ☐
- Arthritis ☐

13. **About smoking**

Please cross the appropriate statement

- Are you a current smoker? ☐
- Are you an ex-smoker? ☐
- Are you a non-smoker (never smoked)? ☐

14. **Do you think you were more at risk of getting cancer because of your family history?**

Yes ☐
No ☐

15. **The following questions are about how completing the questionnaire made you feel.**

A number of statements which people have used to describe themselves are given below. Read each statement and then cross the most appropriate box to indicate how you feel right now, at the moment. There are no right or wrong answers. Do not spend too much time on any one statement but give the answer which seems to describe your present feeling best.

		Not at all	Somewhat	Moderately	Very much
1.	I feel calm	☐	☐	☐	☐
2.	I am tense	☐	☐	☐	☐
3.	I feel upset	☐	☐	☐	☐
4.	I am relaxed	☐	☐	☐	☐
5.	I feel content	☐	☐	☐	☐
6.	I am worried	☐	☐	☐	☐

Please make sure you have answered all the questions.

If you have any other comments or there is anything else you would like to tell us about when you first noticed your symptoms, please do so using the space below.

If you feel you would like to talk to someone about this questionnaire at a later stage, please speak to the doctor or nurse who is looking after you, or be in touch with the research team, whose contact details are on your patient information sheet

Thank you for completing the questionnaire

Please give it to the research nurse

The next page is for the Researcher to complete

57727

65

Researcher - Post-completion Sheet

Researcher initials: ☐☐☐

Date questionnaire completed: Date ☐☐ / ☐☐ / ☐☐ Time ☐☐:☐☐

If not completed, give reason ☐

How long is it since the patient was told of his/her diagnosis? ☐

How long did it take the patient to complete the questionnaire?

- Less than 5 minutes ☐
- Between 5 and 10 minutes ☐
- Between 11 and 15 minutes ☐
- Longer than 15 minutes ☐

For the patient selected to complete the questionnaire on their own

Did the patient ask for any help? ☐ Yes ☐ No

If yes,

please specify what help was requested

____

____

please specify what help was given

____

____

For the patient selected for the researcher-administered questionnaire

Did the patient find any of the questions difficult? ☐ Yes ☐ No

If yes,

please specify which questions and what the difficulty was

____

____

____

____

Was the patient made anxious? ☐ Yes ☐ No

If yes,

please give details

____

____

____

____

Where did the patient complete the questionnaire?
(Please specify whether this was in a separate room alone, in a corner of the clinic, the ward or other

____

Thank you. Please now send the questionnaire and patient information sheet and consent form back to Cardiff University in the freepost envelope provided.

57727

PID - 6 6

Ymddiriedolaeth GIG Gogledd Orllewin Cymru
North West Wales NHS Trust

NHS WALES GIG CYMRU
Ymddiriedolaeth GIG Gogledd Cymru
North Wales NHS Trust

CID - ☐ GLAN CLWYD
☐ GWYNEDD
☐ MAELOR

Confidential Questionnaire

Looking At Your Symptoms Study

This Study has been funded by Cancer Research UK

Cardiff University / Prifysgol Caerdydd
Ysgol Glinigol Gogledd Cymru / North Wales Clinical School
Cancer Research UK
sewtu — South East Wales Trials Unit / Uned Ymchwil De-ddwyrain Cymru

Dear Patient

I am very grateful to you for agreeing to help us with this study.

I hope the information you supply will help us give people a diagnosis earlier.

If you feel you would like to talk to someone about this questionnaire at a later stage, please speak to the doctor or nurse who is looking after you, or be in touch with the research team, whose contact details are on your patient information sheet.

I can assure you that anything you tell us will be treated in the utmost confidence.

Yours sincerely

Dr Richard Neal
Senior Clinical Lecturer
Cardiff University
Chief Investigator, Symptoms Study

About your symptoms

These questions are about symptoms you noticed before your diagnosis. We are interested in symptoms which you think are related to your cancer.

Please put a cross in the appropriate box, or write an answer as required.

Looking back...

1. Did you have any symptoms that you feel were due to your cancer before you were diagnosed?

Yes ☐ **Please continue with Question 2**

No ☐ **Please go to Section 2 on Page 5**

2. What was the first symptom that made you think something might be wrong?

[ ]

Please go to Question 3

The following questions are about when you first noticed a symptom and when you first told your GP or nurse about it.

Please give an exact date if you can. Otherwise give your best estimate. (For example approximately how long ago, the month or season). You wish to refer to your diary or calendar if you have it with you.

Here is an example question:

Decrease in appetite

Did you have this symptom?

Yes ☒ **Please complete A then B or C below**

No ☐ **Please go to Section 2**

A When did you first notice this? d d / m m / y y **OR** Estimate '3 months ago' or 'June'

B When did you first tell your GP or nurse? 2 3 / 1 2 / 0 7 **OR** Estimate

OR

C Put a cross here if you didn't tell your GP or nurse ☐

3. Change in bowel habit

Did you have this symptom?

Yes ☐ **Please complete A then B or C below**

No ☐ **Please go to Question 4**

A When did you first notice this? ☐☐ / ☐☐ / ☐☐ **OR** ☐

B When did you first tell your GP or nurse? ☐☐ / ☐☐ / ☐☐ **OR** ☐

OR

C Put a cross here if you didn't tell your GP or nurse ☐

4. Bleeding from back passage

Did you have this symptom?

Yes ☐ **Please complete A then B or C below**

No ☐ **Please go to Section 2**

A When did you first notice this? ☐☐ / ☐☐ / ☐☐ **OR** ☐

B When did you first tell your GP or nurse? ☐☐ / ☐☐ / ☐☐ **OR** ☐

OR

C Put a cross here if you didn't tell your GP or nurse ☐

Section 2

Please give an exact date if you can. Otherwise give your best estimate (for example approximately how long ago, the month or the season). You may wish to refer to your diary or calendar if you have it with you.

Did you have any of the following?

1. Decrease in appetite

Did you have this?

Yes ☐ **Please complete A then B or C below**

No ☐ **Please go to Question 2**

A When did you first notice this? ☐☐ / ☐☐ / ☐☐ **OR** ☐

B When did you first tell your GP or nurse? ☐☐ / ☐☐ / ☐☐ **OR** ☐

OR

C Put a cross here if you didn't tell your GP or nurse ☐

2. Unexplained weight loss

Did you have this?

Yes ☐ **Please complete A then B or C below**

No ☐ **Please go to Question 3**

A When did you first notice this? ☐☐ / ☐☐ / ☐☐ **OR** ☐

B When did you first tell your GP or nurse? ☐☐ / ☐☐ / ☐☐ **OR** ☐

OR

C Put a cross here if you didn't tell your GP or nurse ☐

3. **Fatigue or tiredness that is unusual for you**

Did you have this?

Yes ☐ **Please complete A then B or C below**

No ☐ **Please go to Question 4**

A When did you first notice this? ☐☐ / ☐☐ / ☐☐ **OR** ☐

B When did you first tell your GP or nurse? ☐☐ / ☐☐ / ☐☐ **OR** ☐

OR

C Put a cross here if you didn't tell your GP or nurse ☐

4. **Feeling different 'in yourself' from usual**

Did you have this?

Yes ☐ **Please complete A then B or C below**

No ☐ **Please go to Question 5**

A When did you first notice this? ☐☐ / ☐☐ / ☐☐ **OR** ☐

B When did you first tell your GP or nurse? ☐☐ / ☐☐ / ☐☐ **OR** ☐

OR

C Put a cross here if you didn't tell your GP or nurse ☐

Please try and describe what the feeling was

If you had any further symptoms which you feel are relevant to your cancer diagnosis, please list them below. If not, please go to Question 7.

Please describe the symptom here and complete A then B or C below

5.

**A**	When did you first notice this?	☐☐ / ☐☐ / ☐☐ **OR** ☐
**B**	When did you first tell your GP or nurse?	☐☐ / ☐☐ / ☐☐ **OR** ☐
	**OR**	
**C**	Put a cross here if you didn't tell your GP or nurse	☐

If you had another symptom you feel is relevant, please write it below, otherwise go to Question 7.

Please describe the symptom here and complete A then B or C below

6.

**A**	When did you first notice this?	☐☐ / ☐☐ / ☐☐ **OR** ☐
**B**	When did you first tell your GP or nurse?	☐☐ / ☐☐ / ☐☐ **OR** ☐
	**OR**	
**C**	Put a cross here if you didn't tell your GP or nurse	☐

If you had further symptoms you would like to tell us about, there is space at the end of the Questionnaire for you to write them.

Did your GP send you for any tests?

Thinking back to the time immediately before you diagnosis, did your GP send you for any of the following tests?

Please cross the appropriate boxes

7. Were you sent for any of these?

	Yes	Not sure	No
Blood test(s)	☐	☐	☐
CT Scan	☐	☐	☐
Ultrasound scan	☐	☐	☐
Barium Enema	☐	☐	☐
Sigmoidoscopy or colonoscopy (Looking at bowel with internal camera)	☐	☐	☐
X-ray	☐	☐	☐

Please go to Question 8

About you

The next questions relate to general information about you. It would help us to know more about the background of the people completing the questionnaire.

8. Which best describes your employment status?

Please cross one box only

- Employed full-time ☐
- Employed part-time ☐
- Self employed full-time ☐
- Self employed part-time ☐
- Unemployed (seeking work) ☐
- Unemployed (not seeking work) ☐
- Retired ☐
- Student ☐
- Permanently sick/disabled ☐
- Temporarily sick/disabled ☐
- Looking after family/home ☐
- Other, please describe ☐

9. What is your highest level of qualification?

Please cross one box only

- Degree (or equivalent) ☐
- Diploma (or equivalent) ☐
- 'A' level ☐
- GCSE / 'O' level ☐
- None ☐
- Other, please specify ☐

10. How would you describe your ethnicity

Please cross one box only

White ☐
or
White British ☐
White Irish ☐
Other White background ☐

Mixed ☐
or
White & Black Carribean ☐
White & Black African ☐
White & Asian ☐
Other mixed background ☐

Black or Black British ☐
or
Carribean ☐
African ☐
Other Black background ☐

Asian or Asian British ☐
or
Indian ☐
Pakistani ☐
Bangladeshi ☐
Other Asian background ☐

Chinese or other ethnic group ☐
or
Chinese ☐
Other ethnic group ☐

11.

Do you live alone?

Yes ☐ **Please go to Question 12**

No ☐ **Please say who you live with below**

Who do you live with?
(e.g. wife, husband, partner, family member)

12. Are you suffering from, or have you suffered from, any of the following in the past 2 years?

Please cross any that are applicable to you

- Asthma ☐
- Chronic Obstructive Pulmonary Disease (COPD) ☐
- Other lung disease (e.g. fibrosis, bronchiectatis etc) ☐
- Heart disease ☐
- Anxiety or depression ☐
- Inflammatory bowel disease ☐
- Irritable bowel syndrome ☐
- Peptic ulcer ☐
- Previous cancer ☐
- Diabetes ☐
- Arthritis ☐

13. About smoking

Please cross the appropriate statement

- Are you a current smoker? ☐
- Are you an ex-smoker? ☐
- Are you a non-smoker (never smoked)? ☐

14. Do you think you were more at risk of getting cancer because of your family history?

Yes ☐

No ☐

15. **The following questions are about how completing the questionnaire made you feel.**

A number of statements which people have used to describe themselves are given below. Read each statement and then cross the most appropriate box to indicate how you feel <u>right now, at the moment.</u> There are no right or wrong answers. Do not spend too much time on any one statement but give the answer which seems to describe your present feeling best.

		Not at all	Somewhat	Moderately	Very much
1.	I feel calm	☐	☐	☐	☐
2.	I am tense	☐	☐	☐	☐
3.	I feel upset	☐	☐	☐	☐
4.	I am relaxed	☐	☐	☐	☐
5.	I feel content	☐	☐	☐	☐
6.	I am worried	☐	☐	☐	☐

Please make sure you have answered all the questions.

If you have any other comments or there is anything else you would like to tell us about when you first noticed your symptoms, please do so using the space below.

If you feel you would like to talk to someone about this questionnaire at a later stage, please speak to the doctor or nurse who is looking after you, or be in touch with the research team, whose contact details are on your patient information sheet

Thank you for completing the questionnaire

Please give it to the research nurse

The next page is for the Researcher to complete

57727

66

Researcher - Post-completion Sheet

Researcher initials: ☐☐☐

Date questionnaire completed: Date ☐☐ / ☐☐ / ☐☐ Time ☐☐:☐☐

If not completed, give reason ☐

How long is it since the patient was told of his/her diagnosis? ☐

How long did it take the patient to complete the questionnaire?

- Less than 5 minutes ☐
- Between 5 and 10 minutes ☐
- Between 11 and 15 minutes ☐
- Longer than 15 minutes ☐

For the patient selected to complete the questionnaire on their own

Did the patient ask for any help? ☐ Yes ☐ No

If yes,

please specify what help was requested

____

____

please specify what help was given

____

____

For the patient selected for the researcher-administered questionnaire

Did the patient find any of the questions difficult? ☐ Yes ☐ No

If yes,

please specify which questions and what the difficulty was

____

____

____

____

Was the patient made anxious? ☐ Yes ☐ No

If yes,

please give details

____

____

____

____

Where did the patient complete the questionnaire?
(Please specify whether this was in a separate room alone, in a corner of the clinic, the ward or other

____

Thank you. Please now send the questionnaire and patient information sheet and consent form back to Cardiff University in the freepost envelope provided.

57727

PID - | | 6 | 7 |

Ymddiriedolaeth GIG Gogledd Orllewin Cymru
North West Wales NHS Trust

NHS WALES GIG CYMRU
Ymddiriedolaeth GIG Gogledd Cymru
North Wales NHS Trust

☐ GLAN CLWYD

CID - ☐ GWYNEDD

☐ MAELOR

Confidential Questionnaire

Looking At Your Symptoms Study

This Study has been funded by Cancer Research UK

Cardiff University / Prifysgol Caerdydd

Ysgol Glinigol Gogledd Cymru / North Wales Clinical School

Cancer Research UK

sewtu — South East Wales Trials Unit / Uned Ymchwil De-ddwyrain Cymru

Dear Patient

I am very grateful to you for agreeing to help us with this study.

I hope the information you supply will help us give people a diagnosis earlier.

If you feel you would like to talk to someone about this questionnaire at a later stage, please speak to the doctor or nurse who is looking after you, or be in touch with the research team, whose contact details are on your patient information sheet.

I can assure you that anything you tell us will be treated in the utmost confidence.

Yours sincerely

Dr Richard Neal
Senior Clinical Lecturer
Cardiff University
Chief Investigator, Symptoms Study

About your symptoms

These questions are about symptoms you noticed before your diagnosis. We are interested in symptoms which you think are related to your cancer.

Please put a cross in the appropriate box, or write an answer as required.

Looking back...

1. Did you have any symptoms that you feel were due to your cancer before you were diagnosed?

Yes ☐ **Please continue with Question 2**

No ☐ **Please go to Section 2 on Page 5**

2. What was the first symptom that made you think something might be wrong?

☐

Please go to Question 3

The following questions are about when you first noticed a symptom and when you first told your GP or nurse about it.

Please give an exact date if you can. Otherwise give your best estimate. (For example approximately how long ago, the month or season). You wish to refer to your diary or calendar if you have it with you.

Here is an example question:

Decrease in appetite

Did you have this symptom?

Yes ☒ **Please complete A then B or C below**

No ☐ **Please go to Section 2**

A When did you first notice this? d d / m m / y y **OR** Estimate '3 months ago' or 'June'

B When did you first tell your GP or nurse? 2 3 / 1 2 / 0 7 **OR** Estimate

OR

C Put a cross here if you didn't tell your GP or nurse ☐

3. Change in bowel habit

Did you have this symptom?

Yes ☐ **Please complete A then B or C below**

No ☐ **Please go to Question 4**

A When did you first notice this? ☐☐ / ☐☐ / ☐☐ **OR** ☐

B When did you first tell your GP or nurse? ☐☐ / ☐☐ / ☐☐ **OR** ☐

OR

C Put a cross here if you didn't tell your GP or nurse ☐

4. Bleeding from back passage

Did you have this symptom?

Yes ☐ **Please complete A then B or C below**

No ☐ **Please go to Section 2**

A When did you first notice this? ☐☐ / ☐☐ / ☐☐ **OR** ☐

B When did you first tell your GP or nurse? ☐☐ / ☐☐ / ☐☐ **OR** ☐

OR

C Put a cross here if you didn't tell your GP or nurse ☐

Section 2

Please give an exact date if you can. Otherwise give your best estimate (for example approximately how long ago, the month or the season). You may wish to refer to your diary or calendar if you have it with you.

Did you have any of the following?

1. Decrease in appetite

Did you have this?

Yes ☐ **Please complete A then B or C below**

No ☐ **Please go to Question 2**

A When did you first notice this? ☐☐ / ☐☐ / ☐☐ **OR** ☐

B When did you first tell your GP or nurse? ☐☐ / ☐☐ / ☐☐ **OR** ☐

OR

C Put a cross here if you didn't tell your GP or nurse ☐

2. Unexplained weight loss

Did you have this?

Yes ☐ **Please complete A then B or C below**

No ☐ **Please go to Question 3**

A When did you first notice this? ☐☐ / ☐☐ / ☐☐ **OR** ☐

B When did you first tell your GP or nurse? ☐☐ / ☐☐ / ☐☐ **OR** ☐

OR

C Put a cross here if you didn't tell your GP or nurse ☐

3. Fatigue or tiredness that is unusual for you

Did you have this?

Yes ☐ **Please complete A then B or C below**

No ☐ **Please go to Question 4**

A When did you first notice this? ☐☐ / ☐☐ / ☐☐ **OR** ☐

B When did you first tell your GP or nurse? ☐☐ / ☐☐ / ☐☐ **OR** ☐

OR

C Put a cross here if you didn't tell your GP or nurse ☐

4. Feeling different 'in yourself' from usual

Did you have this?

Yes ☐ **Please complete A then B or C below**

No ☐ **Please go to Question 5**

A When did you first notice this? ☐☐ / ☐☐ / ☐☐ **OR** ☐

B When did you first tell your GP or nurse? ☐☐ / ☐☐ / ☐☐ **OR** ☐

OR

C Put a cross here if you didn't tell your GP or nurse ☐

Please try and describe what the feeling was

If you had any further symptoms which you feel are relevant to your cancer diagnosis, please list them below. If not, please go to Question 7.

Please describe the symptom here and complete A then B or C below

5.

A When did you first notice this? ☐☐ / ☐☐ / ☐☐ **OR** ☐

B When did you first tell your GP or nurse? ☐☐ / ☐☐ / ☐☐ **OR** ☐

OR

C Put a cross here if you didn't tell your GP or nurse ☐

If you had another symptom you feel is relevant, please write it below, otherwise go to Question 7.

Please describe the symptom here and complete A then B or C below

6.

A When did you first notice this? ☐☐ / ☐☐ / ☐☐ **OR** ☐

B When did you first tell your GP or nurse? ☐☐ / ☐☐ / ☐☐ **OR** ☐

OR

C Put a cross here if you didn't tell your GP or nurse ☐

If you had further symptoms you would like to tell us about, there is space at the end of the Questionnaire for you to write them.

Did your GP send you for any tests?

Thinking back to the time immediately before you diagnosis, did your GP send you for any of the following tests?

Please cross the appropriate boxes

7. Were you sent for any of these?

	Yes	Not sure	No
Blood test(s)	☐	☐	☐
CT Scan	☐	☐	☐
Ultrasound scan	☐	☐	☐
Barium Enema	☐	☐	☐
Sigmoidoscopy or colonoscopy (Looking at bowel with internal camera)	☐	☐	☐
X-ray	☐	☐	☐

Please go to Question 8

About you

The next questions relate to general information about you. It would help us to know more about the background of the people completing the questionnaire.

8. Which best describes your employment status?

Please cross one box only

- Employed full-time ☐
- Employed part-time ☐
- Self employed full-time ☐
- Self employed part-time ☐
- Unemployed (seeking work) ☐
- Unemployed (not seeking work) ☐
- Retired ☐
- Student ☐
- Permanently sick/disabled ☐
- Temporarily sick/disabled ☐
- Looking after family/home ☐

Other, please describe ☐

9. What is your highest level of qualification?

Please cross one box only

- Degree (or equivalent) ☐
- Diploma (or equivalent) ☐
- 'A' level ☐
- GCSE / 'O' level ☐
- None ☐

Other, please specify ☐

10. How would you describe your ethnicity

Please cross one box only

White ☐
or
- White British ☐
- White Irish ☐
- Other White background ☐

Mixed ☐
or
- White & Black Carribean ☐
- White & Black African ☐
- White & Asian ☐
- Other mixed background ☐

Black or Black British ☐
or
- Carribean ☐
- African ☐
- Other Black background ☐

Asian or Asian British ☐
or
- Indian ☐
- Pakistani ☐
- Bangladeshi ☐
- Other Asian background ☐

Chinese or other ethnic group ☐
or
- Chinese ☐
- Other ethnic group ☐

11.

Do you live alone?

Yes ☐ **Please go to Question 12**

No ☐ **Please say who you live with below**

Who do you live with?
(e.g. wife, husband, partner, family member)

12. **Are you suffering from, or have you suffered from, any of the following in the past 2 years?**

Please cross any that are applicable to you

- Asthma ☐
- Chronic Obstructive Pulmonary Disease (COPD) ☐
- Other lung disease (e.g. fibrosis, bronchiectatis etc) ☐
- Heart disease ☐
- Anxiety or depression ☐
- Inflammatory bowel disease ☐
- Irritable bowel syndrome ☐
- Peptic ulcer ☐
- Previous cancer ☐
- Diabetes ☐
- Arthritis ☐

13. **About smoking**

Please cross the appropriate statement

- Are you a current smoker? ☐
- Are you an ex-smoker? ☐
- Are you a non-smoker (never smoked)? ☐

14. **Do you think you were more at risk of getting cancer because of your family history?**

Yes ☐

No ☐

15. **The following questions are about how completing the questionnaire made you feel.**

A number of statements which people have used to describe themselves are given below. Read each statement and then cross the most appropriate box to indicate how you feel right now, at the moment. There are no right or wrong answers. Do not spend too much time on any one statement but give the answer which seems to describe your present feeling best.

		Not at all	Somewhat	Moderately	Very much
1.	I feel calm	☐	☐	☐	☐
2.	I am tense	☐	☐	☐	☐
3.	I feel upset	☐	☐	☐	☐
4.	I am relaxed	☐	☐	☐	☐
5.	I feel content	☐	☐	☐	☐
6.	I am worried	☐	☐	☐	☐

Please make sure you have answered all the questions.

If you have any other comments or there is anything else you would like to tell us about when you first noticed your symptoms, please do so using the space below.

If you feel you would like to talk to someone about this questionnaire at a later stage, please speak to the doctor or nurse who is looking after you, or be in touch with the research team, whose contact details are on your patient information sheet

Thank you for completing the questionnaire

Please give it to the research nurse

The next page is for the Researcher to complete

57727

6 7

Researcher - Post-completion Sheet

Researcher initials: ☐☐☐

Date questionnaire completed: Date ☐☐ / ☐☐ / ☐☐ Time ☐☐:☐☐

If not completed, give reason ☐

How long is it since the patient was told of his/her diagnosis? ☐

How long did it take the patient to complete the questionnaire?

- Less than 5 minutes ☐
- Between 5 and 10 minutes ☐
- Between 11 and 15 minutes ☐
- Longer than 15 minutes ☐

For the patient selected to complete the questionnaire on their own

Did the patient ask for any help? ☐ Yes ☐ No

If yes,

please specify what help was requested

___

___

please specify what help was given

___

___

For the patient selected for the researcher-administered questionnaire

Did the patient find any of the questions difficult? ☐ Yes ☐ No

If yes,

please specify which questions and what the difficulty was

___

___

___

___

Was the patient made anxious? ☐ Yes ☐ No

If yes,

please give details

___

___

___

___

Where did the patient complete the questionnaire?

(Please specify whether this was in a separate room alone, in a corner of the clinic, the ward or other

___

Thank you. Please now send the questionnaire and patient information sheet and consent form back to Cardiff University in the freepost envelope provided.

57727

PID - 6 8

Ymddiriedolaeth GIG Gogledd Orllewin Cymru
North West Wales NHS Trust

NHS Wales GIG Cymru
Ymddiriedolaeth GIG Gogledd Cymru
North Wales NHS Trust

CID -
- ☐ GLAN CLWYD
- ☐ GWYNEDD
- ☐ MAELOR

Confidential Questionnaire

Looking At Your Symptoms Study

This Study has been funded by Cancer Research UK

Cardiff University / Prifysgol Caerdydd
Ysgol Glinigol Gogledd Cymru / North Wales Clinical School
Cancer Research UK
sewtu South East Wales Trials Unit / Uned Ymchwil De-ddwyrain Cymru

Dear Patient

I am very grateful to you for agreeing to help us with this study.

I hope the information you supply will help us give people a diagnosis earlier.

If you feel you would like to talk to someone about this questionnaire at a later stage, please speak to the doctor or nurse who is looking after you, or be in touch with the research team, whose contact details are on your patient information sheet.

I can assure you that anything you tell us will be treated in the utmost confidence.

Yours sincerely

Richard Neal

Dr Richard Neal
Senior Clinical Lecturer
Cardiff University
Chief Investigator, Symptoms Study

About your symptoms

These questions are about symptoms you noticed before your diagnosis. We are interested in symptoms which you think are related to your cancer.

Please put a cross in the appropriate box, or write an answer as required.

Looking back...

1. Did you have any symptoms that you feel were due to your cancer before you were diagnosed?

Yes ☐ **Please continue with Question 2**

No ☐ **Please go to Section 2 on Page 5**

2. What was the first symptom that made you think something might be wrong?

☐

Please go to Question 3

The following questions are about when you first noticed a symptom and when you first told your GP or nurse about it.

Please give an exact date if you can. Otherwise give your best estimate. (For example approximately how long ago, the month or season). You wish to refer to your diary or calendar if you have it with you.

Here is an example question:

Decrease in appetite

Did you have this symptom?

Yes ☒ **Please complete A then B or C below**

No ☐ **Please go to Section 2**

A When did you first notice this? d d / m m / y y **OR** Estimate: '3 months ago' or 'June'

B When did you first tell your GP or nurse? 2 3 / 1 2 / 0 7 **OR** Estimate

OR

C Put a cross here if you didn't tell your GP or nurse ☐

3. Change in bowel habit

Did you have this symptom?

Yes ☐ **Please complete A then B or C below**

No ☐ **Please go to Question 4**

A When did you first notice this? ☐☐ / ☐☐ / ☐☐ **OR** ☐

B When did you first tell your GP or nurse? ☐☐ / ☐☐ / ☐☐ **OR** ☐

OR

C Put a cross here if you didn't tell your GP or nurse ☐

4. Bleeding from back passage

Did you have this symptom?

Yes ☐ **Please complete A then B or C below**

No ☐ **Please go to Section 2**

A When did you first notice this? ☐☐ / ☐☐ / ☐☐ **OR** ☐

B When did you first tell your GP or nurse? ☐☐ / ☐☐ / ☐☐ **OR** ☐

OR

C Put a cross here if you didn't tell your GP or nurse ☐

Please give an exact date if you can. Otherwise give your best estimate (for example approximately how long ago, the month or the season). You may wish to refer to your diary or calendar if you have it with you.

Did you have any of the following?

1. Decrease in appetite

Did you have this?

Yes ☐ **Please complete A then B or C below**

No ☐ **Please go to Question 2**

A When did you first notice this? ☐☐ / ☐☐ / ☐☐ **OR** ☐

B When did you first tell your GP or nurse? ☐☐ / ☐☐ / ☐☐ **OR** ☐

OR

C Put a cross here if you didn't tell your GP or nurse ☐

2. Unexplained weight loss

Did you have this?

Yes ☐ **Please complete A then B or C below**

No ☐ **Please go to Question 3**

A When did you first notice this? ☐☐ / ☐☐ / ☐☐ **OR** ☐

B When did you first tell your GP or nurse? ☐☐ / ☐☐ / ☐☐ **OR** ☐

OR

C Put a cross here if you didn't tell your GP or nurse ☐

3. Fatigue or tiredness that is unusual for you

Did you have this?

Yes ☐ **Please complete A then B or C below**

No ☐ **Please go to Question 4**

A When did you first notice this? ☐☐ / ☐☐ / ☐☐ **OR** ☐

B When did you first tell your GP or nurse? ☐☐ / ☐☐ / ☐☐ **OR** ☐

OR

C Put a cross here if you didn't tell your GP or nurse ☐

4. Feeling different 'in yourself' from usual

Did you have this?

Yes ☐ **Please complete A then B or C below**

No ☐ **Please go to Question 5**

A When did you first notice this? ☐☐ / ☐☐ / ☐☐ **OR** ☐

B When did you first tell your GP or nurse? ☐☐ / ☐☐ / ☐☐ **OR** ☐

OR

C Put a cross here if you didn't tell your GP or nurse ☐

Please try and describe what the feeling was

If you had any further symptoms which you feel are relevant to your cancer diagnosis, please list them below. If not, please go to Question 7.

Please describe the symptom here and complete A then B or C below

5.

**A** When did you <u>first notice</u> this?	☐☐ / ☐☐ / ☐☐	**OR** ☐
**B** When did you <u>first tell</u> your GP or nurse?	☐☐ / ☐☐ / ☐☐	**OR** ☐
**OR**		
**C** Put a cross here if you <u>didn't tell</u> your GP or nurse	☐	

If you had another symptom you feel is relevant, please write it below, otherwise go to Question 7.

Please describe the symptom here and complete A then B or C below

6.

**A** When did you <u>first notice</u> this?	☐☐ / ☐☐ / ☐☐	**OR** ☐
**B** When did you <u>first tell</u> your GP or nurse?	☐☐ / ☐☐ / ☐☐	**OR** ☐
**OR**		
**C** Put a cross here if you <u>didn't tell</u> your GP or nurse	☐	

If you had further symptoms you would like to tell us about, there is space at the end of the Questionnaire for you to write them.

Did your GP send you for any tests?

Thinking back to the time immediately before you diagnosis, did your GP send you for any of the following tests?

Please cross the appropriate boxes

7. Were you sent for any of these?

	Yes	Not sure	No
Blood test(s)	☐	☐	☐
CT Scan	☐	☐	☐
Ultrasound scan	☐	☐	☐
Barium Enema	☐	☐	☐
Sigmoidoscopy or colonoscopy (Looking at bowel with internal camera)	☐	☐	☐
X-ray	☐	☐	☐

Please go to Question 8

About you

The next questions relate to general information about you. It would help us to know more about the background of the people completing the questionnaire.

8. Which best describes your employment status?

Please cross one box only

- Employed full-time ☐
- Employed part-time ☐
- Self employed full-time ☐
- Self employed part-time ☐
- Unemployed (seeking work) ☐
- Unemployed (not seeking work) ☐
- Retired ☐
- Student ☐
- Permanently sick/disabled ☐
- Temporarily sick/disabled ☐
- Looking after family/home ☐

Other, please describe

9. What is your highest level of qualification?

Please cross one box only

- Degree (or equivalent) ☐
- Diploma (or equivalent) ☐
- 'A' level ☐
- GCSE / 'O' level ☐
- None ☐

Other, please specify

10. **How would you describe your ethnicity**

Please cross one box only

White ☐
or
White British ☐
White Irish ☐
Other White background ☐

Mixed ☐
or
White & Black Carribean ☐
White & Black African ☐
White & Asian ☐
Other mixed background ☐

Black or Black British ☐
or
Carribean ☐
African ☐
Other Black background ☐

Asian or Asian British ☐
or
Indian ☐
Pakistani ☐
Bangladeshi ☐
Other Asian background ☐

Chinese or other ethnic group ☐
or
Chinese ☐
Other ethnic group ☐

11.

Do you live alone?

Yes ☐ **Please go to Question 12**

No ☐ **Please say who you live with below**

Who do you live with?
(e.g. wife, husband, partner, family member)

12. Are you suffering from, or have you suffered from, any of the following in the past 2 years?

Please cross any that are applicable to you

- Asthma ☐
- Chronic Obstructive Pulmonary Disease (COPD) ☐
- Other lung disease (e.g. fibrosis, bronchiectatis etc) ☐
- Heart disease ☐
- Anxiety or depression ☐
- Inflammatory bowel disease ☐
- Irritable bowel syndrome ☐
- Peptic ulcer ☐
- Previous cancer ☐
- Diabetes ☐
- Arthritis ☐

13. About smoking

Please cross the appropriate statement

- Are you a current smoker? ☐
- Are you an ex-smoker? ☐
- Are you a non-smoker (never smoked)? ☐

14. Do you think you were more at risk of getting cancer because of your family history?

Yes ☐

No ☐

15. **The following questions are about how completing the questionnaire made you feel.**

A number of statements which people have used to describe themselves are given below. Read each statement and then cross the most appropriate box to indicate how you feel right now, at the moment. There are no right or wrong answers. Do not spend too much time on any one statement but give the answer which seems to describe your present feeling best.

		Not at all	Somewhat	Moderately	Very much
1.	I feel calm	☐	☐	☐	☐
2.	I am tense	☐	☐	☐	☐
3.	I feel upset	☐	☐	☐	☐
4.	I am relaxed	☐	☐	☐	☐
5.	I feel content	☐	☐	☐	☐
6.	I am worried	☐	☐	☐	☐

Please make sure you have answered all the questions.

If you have any other comments or there is anything else you would like to tell us about when you first noticed your symptoms, please do so using the space below.

If you feel you would like to talk to someone about this questionnaire at a later stage, please speak to the doctor or nurse who is looking after you, or be in touch with the research team, whose contact details are on your patient information sheet

Thank you for completing the questionnaire

Please give it to the research nurse

The next page is for the Researcher to complete

Researcher - Post-completion Sheet

Researcher initials: ☐☐☐

Date questionnaire completed: Date ☐☐ / ☐☐ / ☐☐ Time ☐☐:☐☐

If not completed, give reason ☐

How long is it since the patient was told of his/her diagnosis? ☐

How long did it take the patient to complete the questionnaire?

- Less than 5 minutes ☐
- Between 5 and 10 minutes ☐
- Between 11 and 15 minutes ☐
- Longer than 15 minutes ☐

For the patient selected to complete the questionnaire on their own

Did the patient ask for any help? ☐ Yes ☐ No

If yes,

please specify what help was requested

____

____

please specify what help was given

____

____

For the patient selected for the researcher-administered questionnaire

Did the patient find any of the questions difficult? ☐ Yes ☐ No

If yes,

please specify which questions and what the difficulty was

____

____

____

____

Was the patient made anxious? ☐ Yes ☐ No

If yes,

please give details

____

____

____

____

Where did the patient complete the questionnaire?

(Please specify whether this was in a separate room alone, in a corner of the clinic, the ward or other

____

Thank you. Please now send the questionnaire and patient information sheet and consent form back to Cardiff University in the freepost envelope provided.

57727

PID - 6 9

Ymddiriedolaeth GIG Gogledd Orllewin Cymru
North West Wales NHS Trust

NHS WALES GIG CYMRU
Ymddiriedolaeth GIG Gogledd Cymru
North Wales NHS Trust

CID - ☐ GLAN CLWYD
☐ GWYNEDD
☐ MAELOR

Confidential Questionnaire

Looking At Your Symptoms Study

This Study has been funded by Cancer Research UK

Cardiff University / Prifysgol Caerdydd

Ysgol Glinigol Gogledd Cymru / North Wales Clinical School

Cancer Research UK

South East Wales Trials Unit / Uned Ymchwil De-ddwyrain Cymru

Dear Patient

I am very grateful to you for agreeing to help us with this study.

I hope the information you supply will help us give people a diagnosis earlier.

If you feel you would like to talk to someone about this questionnaire at a later stage, please speak to the doctor or nurse who is looking after you, or be in touch with the research team, whose contact details are on your patient information sheet.

I can assure you that anything you tell us will be treated in the utmost confidence.

Yours sincerely

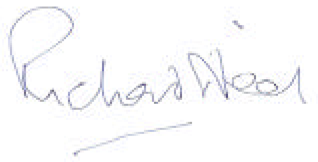

Dr Richard Neal
Senior Clinical Lecturer
Cardiff University
Chief Investigator, Symptoms Study

About your symptoms

These questions are about symptoms you noticed before your diagnosis. We are interested in symptoms which you think are related to your cancer.

Please put a cross in the appropriate box, or write an answer as required.

Looking back...

1. Did you have any symptoms that you feel were due to your cancer before you were diagnosed?

Yes ☐ **Please continue with Question 2**

No ☐ **Please go to Section 2 on Page 5**

2. What was the first symptom that made you think something might be wrong?

☐

Please go to Question 3

The following questions are about when you first noticed a symptom and when you first told your GP or nurse about it.

Please give an exact date if you can. Otherwise give your best estimate. (For example approximately how long ago, the month or season). You wish to refer to your diary or calendar if you have it with you.

Here is an example question:

Decrease in appetite

Did you have this symptom?

Yes ☒ **Please complete A then B or C below**

No ☐ **Please go to Section 2**

A When did you first notice this? d d / m m / y y **OR** Estimate '3 months ago' or 'June'

B When did you first tell your GP or nurse? 2 3 / 1 2 / 0 7 **OR** Estimate

OR

C Put a cross here if you didn't tell your GP or nurse ☐

3. Change in bowel habit

Did you have this symptom?

Yes ☐ **Please complete A then B or C below**

No ☐ **Please go to Question 4**

A When did you first notice this? ☐☐ / ☐☐ / ☐☐ **OR** ☐

B When did you first tell your GP or nurse? ☐☐ / ☐☐ / ☐☐ **OR** ☐

OR

C Put a cross here if you didn't tell your GP or nurse ☐

4. Bleeding from back passage

Did you have this symptom?

Yes ☐ **Please complete A then B or C below**

No ☐ **Please go to Section 2**

A When did you first notice this? ☐☐ / ☐☐ / ☐☐ **OR** ☐

B When did you first tell your GP or nurse? ☐☐ / ☐☐ / ☐☐ **OR** ☐

OR

C Put a cross here if you didn't tell your GP or nurse ☐

Section 2

Please give an exact date if you can. Otherwise give your best estimate (for example approximately how long ago, the month or the season). You may wish to refer to your diary or calendar if you have it with you.

Did you have any of the following?

1. Decrease in appetite

Did you have this?

Yes ☐ **Please complete A then B or C below**

No ☐ **Please go to Question 2**

A When did you first notice this? ☐☐ / ☐☐ / ☐☐ **OR** ☐

B When did you first tell your GP or nurse? ☐☐ / ☐☐ / ☐☐ **OR** ☐

OR

C Put a cross here if you didn't tell your GP or nurse ☐

2. Unexplained weight loss

Did you have this?

Yes ☐ **Please complete A then B or C below**

No ☐ **Please go to Question 3**

A When did you first notice this? ☐☐ / ☐☐ / ☐☐ **OR** ☐

B When did you first tell your GP or nurse? ☐☐ / ☐☐ / ☐☐ **OR** ☐

OR

C Put a cross here if you didn't tell your GP or nurse ☐

3. **Fatigue or tiredness that is unusual for you**

Did you have this?

Yes ☐ **Please complete A then B or C below**

No ☐ **Please go to Question 4**

A When did you first notice this? ☐☐ / ☐☐ / ☐☐ **OR** ☐

B When did you first tell your GP or nurse? ☐☐ / ☐☐ / ☐☐ **OR** ☐

OR

C Put a cross here if you didn't tell your GP or nurse ☐

4. **Feeling different 'in yourself' from usual**

Did you have this?

Yes ☐ **Please complete A then B or C below**

No ☐ **Please go to Question 5**

A When did you first notice this? ☐☐ / ☐☐ / ☐☐ **OR** ☐

B When did you first tell your GP or nurse? ☐☐ / ☐☐ / ☐☐ **OR** ☐

OR

C Put a cross here if you didn't tell your GP or nurse ☐

Please try and describe what the feeling was

If you had any further symptoms which you feel are relevant to your cancer diagnosis, please list them below. If not, please go to Question 7.

Please describe the symptom here and complete A then B or C below

5.

**A**	When did you first notice this?	☐☐ / ☐☐ / ☐☐ **OR**
**B**	When did you first tell your GP or nurse?	☐☐ / ☐☐ / ☐☐ **OR**
	**OR**	
**C**	Put a cross here if you didn't tell your GP or nurse	☐

If you had another symptom you feel is relevant, please write it below, otherwise go to Question 7.

Please describe the symptom here and complete A then B or C below

6.

**A**	When did you first notice this?	☐☐ / ☐☐ / ☐☐ **OR**
**B**	When did you first tell your GP or nurse?	☐☐ / ☐☐ / ☐☐ **OR**
	**OR**	
**C**	Put a cross here if you didn't tell your GP or nurse	☐

If you had further symptoms you would like to tell us about, there is space at the end of the Questionnaire for you to write them.

Did your GP send you for any tests?

Thinking back to the time immediately before you diagnosis, did your GP send you for any of the following tests?

Please cross the appropriate boxes

7. Were you sent for any of these?

	Yes	Not sure	No
Blood test(s)	☐	☐	☐
CT Scan	☐	☐	☐
Ultrasound scan	☐	☐	☐
Barium Enema	☐	☐	☐
Sigmoidoscopy or colonoscopy (Looking at bowel with internal camera)	☐	☐	☐
X-ray	☐	☐	☐

Please go to Question 8

About you

The next questions relate to general information about you. It would help us to know more about the background of the people completing the questionnaire.

8. Which best describes your employment status?

Please cross one box only

- Employed full-time ☐
- Employed part-time ☐
- Self employed full-time ☐
- Self employed part-time ☐
- Unemployed (seeking work) ☐
- Unemployed (not seeking work) ☐
- Retired ☐
- Student ☐
- Permanently sick/disabled ☐
- Temporarily sick/disabled ☐
- Looking after family/home ☐
- Other, please describe ☐

9. What is your highest level of qualification?

Please cross one box only

- Degree (or equivalent) ☐
- Diploma (or equivalent) ☐
- 'A' level ☐
- GCSE / 'O' level ☐
- None ☐
- Other, please specify ☐

10. **How would you describe your ethnicity**

Please cross one box only

White ☐
or
- White British ☐
- White Irish ☐
- Other White background ☐

Mixed ☐
or
- White & Black Carribean ☐
- White & Black African ☐
- White & Asian ☐
- Other mixed background ☐

Black or Black British ☐
or
- Carribean ☐
- African ☐
- Other Black background ☐

Asian or Asian British ☐
or
- Indian ☐
- Pakistani ☐
- Bangladeshi ☐
- Other Asian background ☐

Chinese or other ethnic group ☐
or
- Chinese ☐
- Other ethnic group ☐

11.

Do you live alone?

Yes ☐ **Please go to Question 12**

No ☐ **Please say who you live with below**

Who do you live with?
(e.g. wife, husband, partner, family member)

12. **Are you suffering from, or have you suffered from, any of the following in the past 2 years?**

Please cross any that are applicable to you

- Asthma ☐
- Chronic Obstructive Pulmonary Disease (COPD) ☐
- Other lung disease (e.g. fibrosis, bronchiectatis etc) ☐
- Heart disease ☐
- Anxiety or depression ☐
- Inflammatory bowel disease ☐
- Irritable bowel syndrome ☐
- Peptic ulcer ☐
- Previous cancer ☐
- Diabetes ☐
- Arthritis ☐

13. **About smoking**

Please cross the appropriate statement

- Are you a current smoker? ☐
- Are you an ex-smoker? ☐
- Are you a non-smoker (never smoked)? ☐

14. **Do you think you were more at risk of getting cancer because of your family history?**

Yes ☐

No ☐

15. The following questions are about how completing the questionnaire made you feel.

A number of statements which people have used to describe themselves are given below. Read each statement and then cross the most appropriate box to indicate how you feel right now, at the moment. There are no right or wrong answers. Do not spend too much time on any one statement but give the answer which seems to describe your present feeling best.

		Not at all	Somewhat	Moderately	Very much
1.	I feel calm	☐	☐	☐	☐
2.	I am tense	☐	☐	☐	☐
3.	I feel upset	☐	☐	☐	☐
4.	I am relaxed	☐	☐	☐	☐
5.	I feel content	☐	☐	☐	☐
6.	I am worried	☐	☐	☐	☐

Please make sure you have answered all the questions.

If you have any other comments or there is anything else you would like to tell us about when you first noticed your symptoms, please do so using the space below.

If you feel you would like to talk to someone about this questionnaire at a later stage, please speak to the doctor or nurse who is looking after you, or be in touch with the research team, whose contact details are on your patient information sheet

Thank you for completing the questionnaire

Please give it to the research nurse

The next page is for the Researcher to complete

57727

Researcher - Post-completion Sheet **Researcher initials:** ☐☐☐

Date questionnaire completed: Date ☐☐ / ☐☐ / ☐☐ Time ☐☐:☐☐

If not completed, give reason ☐

How long is it since the patient was told of his/her diagnosis? ☐

How long did it take the patient to complete the questionnaire?

- Less than 5 minutes ☐
- Between 5 and 10 minutes ☐
- Between 11 and 15 minutes ☐
- Longer than 15 minutes ☐

For the patient selected to complete the questionnaire on their own

Did the patient ask for any help? ☐ Yes ☐ No

If yes,

please specify what help was requested

____

____

please specify what help was given

____

____

For the patient selected for the researcher-administered questionnaire

Did the patient find any of the questions difficult? ☐ Yes ☐ No

If yes,

please specify which questions and what the difficulty was

____

____

____

____

Was the patient made anxious? ☐ Yes ☐ No

If yes,

please give details

____

____

____

____

Where did the patient complete the questionnaire?
(Please specify whether this was in a separate room alone, in a corner of the clinic, the ward or other

____

Thank you. Please now send the questionnaire and patient information sheet and consent form back to Cardiff University in the freepost envelope provided.

57727

PID - | | 7 | 0 |

Ymddiriedolaeth GIG Gogledd Orllewin Cymru
North West Wales NHS Trust

NHS WALES GIG CYMRU
Ymddiriedolaeth GIG Gogledd Cymru
North Wales NHS Trust

☐ GLAN CLWYD

CID - ☐ GWYNEDD

☐ MAELOR

Confidential Questionnaire

Looking At Your Symptoms Study

This Study has been funded by Cancer Research UK

Cardiff University / Prifysgol Caerdydd
Ysgol Glinigol Gogledd Cymru / North Wales Clinical School
Cancer Research UK
sewtu South East Wales Trials Unit / Uned Ymchwil De-ddwyrain Cymru

Dear Patient

I am very grateful to you for agreeing to help us with this study.

I hope the information you supply will help us give people a diagnosis earlier.

If you feel you would like to talk to someone about this questionnaire at a later stage, please speak to the doctor or nurse who is looking after you, or be in touch with the research team, whose contact details are on your patient information sheet.

I can assure you that anything you tell us will be treated in the utmost confidence.

Yours sincerely

Dr Richard Neal
Senior Clinical Lecturer
Cardiff University
Chief Investigator, Symptoms Study

About your symptoms

These questions are about symptoms you noticed before your diagnosis. We are interested in symptoms which you think are related to your cancer.

Please put a cross in the appropriate box, or write an answer as required.

Looking back...

1. Did you have any symptoms that you feel were due to your cancer before you were diagnosed?

Yes ☐ **Please continue with Question 2**

No ☐ **Please go to Section 2 on Page 5**

2. What was the first symptom that made you think something might be wrong?

☐

Please go to Question 3

The following questions are about when you first noticed a symptom and when you first told your GP or nurse about it.

Please give an exact date if you can. Otherwise give your best estimate. (For example approximately how long ago, the month or season). You wish to refer to your diary or calendar if you have it with you.

Here is an example question:

Decrease in appetite

Did you have this symptom?

Yes ☒ **Please complete A then B or C below**

No ☐ **Please go to Section 2**

A When did you first notice this? d d / m m / y y **OR** Estimate '3 months ago' or 'June'

B When did you first tell your GP or nurse? 2 3 / 1 2 / 0 7 **OR** Estimate

OR

C Put a cross here if you didn't tell your GP or nurse ☐

3. Change in bowel habit

Did you have this symptom?

Yes ☐ **Please complete A then B or C below**

No ☐ **Please go to Question 4**

A When did you first notice this? ☐☐ / ☐☐ / ☐☐ **OR** ☐

B When did you first tell your GP or nurse? ☐☐ / ☐☐ / ☐☐ **OR** ☐

OR

C Put a cross here if you didn't tell your GP or nurse ☐

4. Bleeding from back passage

Did you have this symptom?

Yes ☐ **Please complete A then B or C below**

No ☐ **Please go to Section 2**

A When did you first notice this? ☐☐ / ☐☐ / ☐☐ **OR** ☐

B When did you first tell your GP or nurse? ☐☐ / ☐☐ / ☐☐ **OR** ☐

OR

C Put a cross here if you didn't tell your GP or nurse ☐

Section 2

Please give an exact date if you can. Otherwise give your best estimate (for example approximately how long ago, the month or the season). You may wish to refer to your diary or calendar if you have it with you.

Did you have any of the following?

1. Decrease in appetite

Did you have this?

Yes ☐ **Please complete A then B or C below**

No ☐ **Please go to Question 2**

A When did you first notice this? ☐☐ / ☐☐ / ☐☐ **OR** ☐

B When did you first tell your GP or nurse? ☐☐ / ☐☐ / ☐☐ **OR** ☐

OR

C Put a cross here if you didn't tell your GP or nurse ☐

2. Unexplained weight loss

Did you have this?

Yes ☐ **Please complete A then B or C below**

No ☐ **Please go to Question 3**

A When did you first notice this? ☐☐ / ☐☐ / ☐☐ **OR** ☐

B When did you first tell your GP or nurse? ☐☐ / ☐☐ / ☐☐ **OR** ☐

OR

C Put a cross here if you didn't tell your GP or nurse ☐

3. **Fatigue or tiredness that is unusual for you**

Did you have this?

Yes ☐ **Please complete A then B or C below**

No ☐ **Please go to Question 4**

A When did you first notice this?

☐☐ / ☐☐ / ☐☐ **OR** ☐

B When did you first tell your GP or nurse?

☐☐ / ☐☐ / ☐☐ **OR** ☐

OR

C Put a cross here if you didn't tell your GP or nurse ☐

4. **Feeling different 'in yourself' from usual**

Did you have this?

Yes ☐ **Please complete A then B or C below**

No ☐ **Please go to Question 5**

A When did you first notice this?

☐☐ / ☐☐ / ☐☐ **OR** ☐

B When did you first tell your GP or nurse?

☐☐ / ☐☐ / ☐☐ **OR** ☐

OR

C Put a cross here if you didn't tell your GP or nurse ☐

Please try and describe what the feeling was

If you had any further symptoms which you feel are relevant to your cancer diagnosis, please list them below. If not, please go to Question 7.

Please describe the symptom here and complete A then B or C below

5.

**A**	When did you first notice this?	☐☐ / ☐☐ / ☐☐ **OR** ☐
**B**	When did you first tell your GP or nurse?	☐☐ / ☐☐ / ☐☐ **OR** ☐
	**OR**	
**C**	Put a cross here if you didn't tell your GP or nurse	☐

If you had another symptom you feel is relevant, please write it below, otherwise go to Question 7.

Please describe the symptom here and complete A then B or C below

6.

**A**	When did you first notice this?	☐☐ / ☐☐ / ☐☐ **OR** ☐
**B**	When did you first tell your GP or nurse?	☐☐ / ☐☐ / ☐☐ **OR** ☐
	**OR**	
**C**	Put a cross here if you didn't tell your GP or nurse	☐

If you had further symptoms you would like to tell us about, there is space at the end of the Questionnaire for you to write them.

Did your GP send you for any tests?

Thinking back to the time immediately before you diagnosis, did your GP send you for any of the following tests?

Please cross the appropriate boxes

7. **Were you sent for any of these?**

	Yes	Not sure	No
Blood test(s)	☐	☐	☐
CT Scan	☐	☐	☐
Ultrasound scan	☐	☐	☐
Barium Enema	☐	☐	☐
Sigmoidoscopy or colonoscopy (Looking at bowel with internal camera)	☐	☐	☐
X-ray	☐	☐	☐

Please go to Question 8

About you

The next questions relate to general information about you. It would help us to know more about the background of the people completing the questionnaire.

8. Which best describes your employment status?

Please cross one box only

- Employed full-time ☐
- Employed part-time ☐
- Self employed full-time ☐
- Self employed part-time ☐
- Unemployed (seeking work) ☐
- Unemployed (not seeking work) ☐
- Retired ☐
- Student ☐
- Permanently sick/disabled ☐
- Temporarily sick/disabled ☐
- Looking after family/home ☐
- Other, please describe [ ]

9. What is your highest level of qualification?

Please cross one box only

- Degree (or equivalent) ☐
- Diploma (or equivalent) ☐
- 'A' level ☐
- GCSE / 'O' level ☐
- None ☐
- Other, please specify [ ]

10. **How would you describe your ethnicity**

Please cross one box only

White ☐
or
White British ☐
White Irish ☐
Other White background ☐

Mixed ☐
or
White & Black Carribean ☐
White & Black African ☐
White & Asian ☐
Other mixed background ☐

Black or Black British ☐
or
Carribean ☐
African ☐
Other Black background ☐

Asian or Asian British ☐
or
Indian ☐
Pakistani ☐
Bangladeshi ☐
Other Asian background ☐

Chinese or other ethnic group ☐
or
Chinese ☐
Other ethnic group ☐

11.

Do you live alone?

Yes ☐ **Please go to Question 12**

No ☐ **Please say who you live with below**

Who do you live with?
(e.g. wife, husband, partner, family member)

12. **Are you suffering from, or have you suffered from, any of the following in the past 2 years?**

Please cross any that are applicable to you

- Asthma ☐
- Chronic Obstructive Pulmonary Disease (COPD) ☐
- Other lung disease (e.g. fibrosis, bronchiectatis etc) ☐
- Heart disease ☐
- Anxiety or depression ☐
- Inflammatory bowel disease ☐
- Irritable bowel syndrome ☐
- Peptic ulcer ☐
- Previous cancer ☐
- Diabetes ☐
- Arthritis ☐

13. **About smoking**

Please cross the appropriate statement

- Are you a current smoker? ☐
- Are you an ex-smoker? ☐
- Are you a non-smoker (never smoked)? ☐

14. **Do you think you were more at risk of getting cancer because of your family history?**

Yes ☐
No ☐

15. The following questions are about how completing the questionnaire made you feel.

A number of statements which people have used to describe themselves are given below. Read each statement and then cross the most appropriate box to indicate how you feel right now, at the moment. There are no right or wrong answers. Do not spend too much time on any one statement but give the answer which seems to describe your present feeling best.

		Not at all	Somewhat	Moderately	Very much
1.	I feel calm	☐	☐	☐	☐
2.	I am tense	☐	☐	☐	☐
3.	I feel upset	☐	☐	☐	☐
4.	I am relaxed	☐	☐	☐	☐
5.	I feel content	☐	☐	☐	☐
6.	I am worried	☐	☐	☐	☐

Please make sure you have answered all the questions.

If you have any other comments or there is anything else you would like to tell us about when you first noticed your symptoms, please do so using the space below.

If you feel you would like to talk to someone about this questionnaire at a later stage, please speak to the doctor or nurse who is looking after you, or be in touch with the research team, whose contact details are on your patient information sheet

Thank you for completing the questionnaire

Please give it to the research nurse

The next page is for the Researcher to complete

57727

7 0

Researcher - Post-completion Sheet

Researcher initials: ☐☐☐

Date questionnaire completed: Date ☐☐ / ☐☐ / ☐☐ Time ☐☐:☐☐

If not completed, give reason ☐

How long is it since the patient was told of his/her diagnosis? ☐

How long did it take the patient to complete the questionnaire?

- Less than 5 minutes ☐
- Between 5 and 10 minutes ☐
- Between 11 and 15 minutes ☐
- Longer than 15 minutes ☐

For the patient selected to complete the questionnaire on their own

Did the patient ask for any help? ☐ Yes ☐ No

If yes,

please specify what help was requested

____

____

please specify what help was given

____

____

For the patient selected for the researcher-administered questionnaire

Did the patient find any of the questions difficult? ☐ Yes ☐ No

If yes,

please specify which questions and what the difficulty was

____

____

____

____

Was the patient made anxious? ☐ Yes ☐ No

If yes,

please give details

____

____

____

____

Where did the patient complete the questionnaire?
(Please specify whether this was in a separate room alone, in a corner of the clinic, the ward or other

____

Thank you. Please now send the questionnaire and patient information sheet and consent form back to Cardiff University in the freepost envelope provided.

57727

PID - | 7 | 1

☐ GLAN CLWYD

CID - ☐ GWYNEDD

☐ MAELOR

Confidential Questionnaire

Looking At Your Symptoms Study

This Study has been funded by Cancer Research UK

Dear Patient

I am very grateful to you for agreeing to help us with this study.

I hope the information you supply will help us give people a diagnosis earlier.

If you feel you would like to talk to someone about this questionnaire at a later stage, please speak to the doctor or nurse who is looking after you, or be in touch with the research team, whose contact details are on your patient information sheet.

I can assure you that anything you tell us will be treated in the utmost confidence.

Yours sincerely

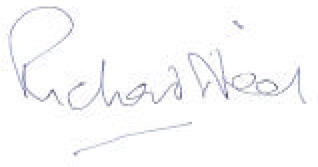

Dr Richard Neal
Senior Clinical Lecturer
Cardiff University
Chief Investigator, Symptoms Study

About your symptoms

These questions are about symptoms you noticed before your diagnosis. We are interested in symptoms which you think are related to your cancer.

Please put a cross in the appropriate box, or write an answer as required.

Looking back...

1. Did you have any symptoms that you feel were due to your cancer before you were diagnosed?

Yes ☐ **Please continue with Question 2**

No ☐ **Please go to Section 2 on Page 5**

2. What was the first symptom that made you think something might be wrong?

☐

Please go to Question 3

The following questions are about when you first noticed a symptom and when you first told your GP or nurse about it.

Please give an exact date if you can. Otherwise give your best estimate. (For example approximately how long ago, the month or season). You wish to refer to your diary or calendar if you have it with you.

Here is an example question:

Decrease in appetite

Did you have this symptom?

Yes ☒ **Please complete A then B or C below**

No ☐ **Please go to Section 2**

A When did you first notice this? d d / m m / y y **OR** Estimate '3 months ago' or 'June'

B When did you first tell your GP or nurse? 2 3 / 1 2 / 0 7 **OR** Estimate

OR

C Put a cross here if you didn't tell your GP or nurse ☐

3. Change in bowel habit

Did you have this symptom?

Yes ☐ **Please complete A then B or C below**

No ☐ **Please go to Question 4**

A When did you <u>first notice</u> this? ☐☐ / ☐☐ / ☐☐ **OR** ☐

B When did you <u>first tell</u> your GP or nurse? ☐☐ / ☐☐ / ☐☐ **OR** ☐

OR

C Put a cross here if you <u>didn't tell</u> your GP or nurse ☐

4. Bleeding from back passage

Did you have this symptom?

Yes ☐ **Please complete A then B or C below**

No ☐ **Please go to Section 2**

A When did you <u>first notice</u> this? ☐☐ / ☐☐ / ☐☐ **OR** ☐

B When did you <u>first tell</u> your GP or nurse? ☐☐ / ☐☐ / ☐☐ **OR** ☐

OR

C Put a cross here if you <u>didn't tell</u> your GP or nurse ☐

Section 2

Please give an exact date if you can. Otherwise give your best estimate (for example approximately how long ago, the month or the season). You may wish to refer to your diary or calendar if you have it with you.

Did you have any of the following?

1. Decrease in appetite

Did you have this?

Yes ☐ **Please complete A then B or C below**

No ☐ **Please go to Question 2**

A When did you first notice this? ☐☐ / ☐☐ / ☐☐ **OR** ☐

B When did you first tell your GP or nurse? ☐☐ / ☐☐ / ☐☐ **OR** ☐

OR

C Put a cross here if you didn't tell your GP or nurse ☐

2. Unexplained weight loss

Did you have this?

Yes ☐ **Please complete A then B or C below**

No ☐ **Please go to Question 3**

A When did you first notice this? ☐☐ / ☐☐ / ☐☐ **OR** ☐

B When did you first tell your GP or nurse? ☐☐ / ☐☐ / ☐☐ **OR** ☐

OR

C Put a cross here if you didn't tell your GP or nurse ☐

3. **Fatigue or tiredness that is unusual for you**

Did you have this?

Yes ☐ **Please complete A then B or C below**

No ☐ **Please go to Question 4**

A When did you <u>first notice</u> this? __ __ / __ __ / __ __ **OR** ______

B When did you <u>first tell</u> your GP or nurse? __ __ / __ __ / __ __ **OR** ______

OR

C Put a cross here if you <u>didn't tell</u> your GP or nurse ☐

4. **Feeling different 'in yourself' from usual**

Did you have this?

Yes ☐ **Please complete A then B or C below**

No ☐ **Please go to Question 5**

A When did you <u>first notice</u> this? __ __ / __ __ / __ __ **OR** ______

B When did you <u>first tell</u> your GP or nurse? __ __ / __ __ / __ __ **OR** ______

OR

C Put a cross here if you <u>didn't tell</u> your GP or nurse ☐

Please try and describe what the feeling was

If you had any further symptoms which you feel are relevant to your cancer diagnosis, please list them below. If not, please go to Question 7.

Please describe the symptom here and complete A then B or C below

5.

**A**	When did you first notice this?	__ __ / __ __ / __ __ **OR**
**B**	When did you first tell your GP or nurse?	__ __ / __ __ / __ __ **OR**
	**OR**	
**C**	Put a cross here if you didn't tell your GP or nurse	☐

If you had another symptom you feel is relevant, please write it below, otherwise go to Question 7.

Please describe the symptom here and complete A then B or C below

6.

**A**	When did you first notice this?	__ __ / __ __ / __ __ **OR**
**B**	When did you first tell your GP or nurse?	__ __ / __ __ / __ __ **OR**
	**OR**	
**C**	Put a cross here if you didn't tell your GP or nurse	☐

If you had further symptoms you would like to tell us about, there is space at the end of the Questionnaire for you to write them.

Did your GP send you for any tests?

Thinking back to the time immediately before you diagnosis, did your GP send you for any of the following tests?

Please cross the appropriate boxes

7. Were you sent for any of these?

	Yes	Not sure	No
Blood test(s)	☐	☐	☐
CT Scan	☐	☐	☐
Ultrasound scan	☐	☐	☐
Barium Enema	☐	☐	☐
Sigmoidoscopy or colonoscopy (Looking at bowel with internal camera)	☐	☐	☐
X-ray	☐	☐	☐

Please go to Question 8

About you

The next questions relate to general information about you. It would help us to know more about the background of the people completing the questionnaire.

8. Which best describes your employment status?

Please cross one box only

- Employed full-time ☐
- Employed part-time ☐
- Self employed full-time ☐
- Self employed part-time ☐
- Unemployed (seeking work) ☐
- Unemployed (not seeking work) ☐
- Retired ☐
- Student ☐
- Permanently sick/disabled ☐
- Temporarily sick/disabled ☐
- Looking after family/home ☐
- Other, please describe ________

9. What is your highest level of qualification?

Please cross one box only

- Degree (or equivalent) ☐
- Diploma (or equivalent) ☐
- 'A' level ☐
- GCSE / 'O' level ☐
- None ☐
- Other, please specify ________

10. **How would you describe your ethnicity**

Please cross one box only

White ☐
or
- White British ☐
- White Irish ☐
- Other White background ☐

Mixed ☐
or
- White & Black Carribean ☐
- White & Black African ☐
- White & Asian ☐
- Other mixed background ☐

Black or Black British ☐
or
- Carribean ☐
- African ☐
- Other Black background ☐

Asian or Asian British ☐
or
- Indian ☐
- Pakistani ☐
- Bangladeshi ☐
- Other Asian background ☐

Chinese or other ethnic group ☐
or
- Chinese ☐
- Other ethnic group ☐

11.

Do you live alone?

Yes ☐ **Please go to Question 12**

No ☐ **Please say who you live with below**

Who do you live with?
(e.g. wife, husband, partner, family member)

12. **Are you suffering from, or have you suffered from, any of the following in the past 2 years?**

Please cross any that are applicable to you

Asthma	☐
Chronic Obstructive Pulmonary Disease (COPD)	☐
Other lung disease (e.g. fibrosis, bronchiectatis etc)	☐
Heart disease	☐
Anxiety or depression	☐
Inflammatory bowel disease	☐
Irritable bowel syndrome	☐
Peptic ulcer	☐
Previous cancer	☐
Diabetes	☐
Arthritis	☐

13. **About smoking**

Please cross the appropriate statement

Are you a current smoker?	☐
Are you an ex-smoker?	☐
Are you a non-smoker (never smoked)?	☐

14. **Do you think you were more at risk of getting cancer because of your family history?**

Yes ☐

No ☐

15. **The following questions are about how completing the questionnaire made you feel.**

A number of statements which people have used to describe themselves are given below. Read each statement and then cross the most appropriate box to indicate how you feel right now, at the moment. There are no right or wrong answers. Do not spend too much time on any one statement but give the answer which seems to describe your present feeling best.

		Not at all	Somewhat	Moderately	Very much
1.	I feel calm	☐	☐	☐	☐
2.	I am tense	☐	☐	☐	☐
3.	I feel upset	☐	☐	☐	☐
4.	I am relaxed	☐	☐	☐	☐
5.	I feel content	☐	☐	☐	☐
6.	I am worried	☐	☐	☐	☐

Please make sure you have answered all the questions.

If you have any other comments or there is anything else you would like to tell us about when you first noticed your symptoms, please do so using the space below.

If you feel you would like to talk to someone about this questionnaire at a later stage, please speak to the doctor or nurse who is looking after you, or be in touch with the research team, whose contact details are on your patient information sheet

Thank you for completing the questionnaire

Please give it to the research nurse

The next page is for the Researcher to complete

Researcher - Post-completion Sheet **Researcher initials:** ☐☐☐

Date questionnaire completed: Date ☐☐ / ☐☐ / ☐☐ Time ☐☐:☐☐

If not completed, give reason ☐

How long is it since the patient was told of his/her diagnosis? ☐

How long did it take the patient to complete the questionnaire?

- Less than 5 minutes ☐
- Between 5 and 10 minutes ☐
- Between 11 and 15 minutes ☐
- Longer than 15 minutes ☐

For the patient selected to complete the questionnaire on their own

Did the patient ask for any help? ☐ Yes ☐ No

If yes,

please specify what help was requested

____

____

please specify what help was given

____

____

For the patient selected for the researcher-administered questionnaire

Did the patient find any of the questions difficult? ☐ Yes ☐ No

If yes,

please specify which questions and what the difficulty was

____

____

____

____

Was the patient made anxious? ☐ Yes ☐ No

If yes,

please give details

____

____

____

____

Where did the patient complete the questionnaire?

(Please specify whether this was in a separate room alone, in a corner of the clinic, the ward or other

____

Thank you. Please now send the questionnaire and patient information sheet and consent form back to Cardiff University in the freepost envelope provided.

57727

PID - | 7 | 2

Ymddiriedolaeth GIG Gogledd Orllewin Cymru
North West Wales NHS Trust

NHS WALES GIG CYMRU
Ymddiriedolaeth GIG Gogledd Cymru
North Wales NHS Trust

CID - ☐ GLAN CLWYD
☐ GWYNEDD
☐ MAELOR

Confidential Questionnaire

Looking At Your Symptoms Study

This Study has been funded by Cancer Research UK

CARDIFF UNIVERSITY PRIFYSGOL CAERDYDD
Ysgol Glinigol Gogledd Cymru North Wales Clinical School
CANCER RESEARCH UK
sewtu South East Wales Trials Unit Uned Ymchwil De-ddwyrain Cymru

Dear Patient

I am very grateful to you for agreeing to help us with this study.

I hope the information you supply will help us give people a diagnosis earlier.

If you feel you would like to talk to someone about this questionnaire at a later stage, please speak to the doctor or nurse who is looking after you, or be in touch with the research team, whose contact details are on your patient information sheet.

I can assure you that anything you tell us will be treated in the utmost confidence.

Yours sincerely

Richard Neal

Dr Richard Neal
Senior Clinical Lecturer
Cardiff University
Chief Investigator, Symptoms Study

About your symptoms

These questions are about symptoms you noticed before your diagnosis. We are interested in symptoms which you think are related to your cancer.

Please put a cross in the appropriate box, or write an answer as required.

Looking back...

1. Did you have any symptoms that you feel were due to your cancer before you were diagnosed?

Yes ☐ **Please continue with Question 2**

No ☐ **Please go to Section 2 on Page 5**

2. What was the first symptom that made you think something might be wrong?

☐

Please go to Question 3

The following questions are about when you first noticed a symptom and when you first told your GP or nurse about it.

Please give an exact date if you can. Otherwise give your best estimate. (For example approximately how long ago, the month or season). You wish to refer to your diary or calendar if you have it with you.

Here is an example question:

Decrease in appetite

Did you have this symptom?

Yes ☒ **Please complete A then B or C below**

No ☐ **Please go to Section 2**

A When did you first notice this? d d / m m / y y **OR** Estimate '3 months ago' or 'June'

B When did you first tell your GP or nurse? 2 3 / 1 2 / 0 7 **OR** Estimate

OR

C Put a cross here if you didn't tell your GP or nurse ☐

3. Change in bowel habit

Did you have this symptom?

Yes ☐ **Please complete A then B or C below**

No ☐ **Please go to Question 4**

A When did you first notice this? ☐☐ / ☐☐ / ☐☐ **OR** ☐

B When did you first tell your GP or nurse? ☐☐ / ☐☐ / ☐☐ **OR** ☐

OR

C Put a cross here if you didn't tell your GP or nurse ☐

4. Bleeding from back passage

Did you have this symptom?

Yes ☐ **Please complete A then B or C below**

No ☐ **Please go to Section 2**

A When did you first notice this? ☐☐ / ☐☐ / ☐☐ **OR** ☐

B When did you first tell your GP or nurse? ☐☐ / ☐☐ / ☐☐ **OR** ☐

OR

C Put a cross here if you didn't tell your GP or nurse ☐

Section 2

Please give an exact date if you can. Otherwise give your best estimate (for example approximately how long ago, the month or the season). You may wish to refer to your diary or calendar if you have it with you.

Did you have any of the following?

1. Decrease in appetite

Did you have this?

Yes ☐ **Please complete A then B or C below**

No ☐ **Please go to Question 2**

A When did you first notice this? ☐☐ / ☐☐ / ☐☐ **OR** ☐

B When did you first tell your GP or nurse? ☐☐ / ☐☐ / ☐☐ **OR** ☐

OR

C Put a cross here if you didn't tell your GP or nurse ☐

2. Unexplained weight loss

Did you have this?

Yes ☐ **Please complete A then B or C below**

No ☐ **Please go to Question 3**

A When did you first notice this? ☐☐ / ☐☐ / ☐☐ **OR** ☐

B When did you first tell your GP or nurse? ☐☐ / ☐☐ / ☐☐ **OR** ☐

OR

C Put a cross here if you didn't tell your GP or nurse ☐

3. Fatigue or tiredness that is unusual for you

Did you have this?

Yes ☐ **Please complete A then B or C below**

No ☐ **Please go to Question 4**

A When did you <u>first notice</u> this? ☐☐ / ☐☐ / ☐☐ **OR** ☐

B When did you <u>first tell</u> your GP or nurse? ☐☐ / ☐☐ / ☐☐ **OR** ☐

OR

C Put a cross here if you <u>didn't tell</u> your GP or nurse ☐

4. Feeling different 'in yourself' from usual

Did you have this?

Yes ☐ **Please complete A then B or C below**

No ☐ **Please go to Question 5**

A When did you <u>first notice</u> this? ☐☐ / ☐☐ / ☐☐ **OR** ☐

B When did you <u>first tell</u> your GP or nurse? ☐☐ / ☐☐ / ☐☐ **OR** ☐

OR

C Put a cross here if you <u>didn't tell</u> your GP or nurse ☐

Please try and describe what the feeling was

If you had any further symptoms which you feel are relevant to your cancer diagnosis, please list them below. If not, please go to Question 7.

Please describe the symptom here and complete A then B or C below

5.

**A**	When did you first notice this?	☐☐ / ☐☐ / ☐☐ **OR** ☐
**B**	When did you first tell your GP or nurse?	☐☐ / ☐☐ / ☐☐ **OR** ☐
	**OR**	
**C**	Put a cross here if you didn't tell your GP or nurse	☐

If you had another symptom you feel is relevant, please write it below, otherwise go to Question 7.

Please describe the symptom here and complete A then B or C below

6.

**A**	When did you first notice this?	☐☐ / ☐☐ / ☐☐ **OR** ☐
**B**	When did you first tell your GP or nurse?	☐☐ / ☐☐ / ☐☐ **OR** ☐
	**OR**	
**C**	Put a cross here if you didn't tell your GP or nurse	☐

If you had further symptoms you would like to tell us about, there is space at the end of the Questionnaire for you to write them.

Did your GP send you for any tests?

Thinking back to the time immediately before you diagnosis, did your GP send you for any of the following tests?

Please cross the appropriate boxes

7. Were you sent for any of these?

	Yes	Not sure	No
Blood test(s)	☐	☐	☐
CT Scan	☐	☐	☐
Ultrasound scan	☐	☐	☐
Barium Enema	☐	☐	☐
Sigmoidoscopy or colonoscopy (Looking at bowel with internal camera)	☐	☐	☐
X-ray	☐	☐	☐

Please go to Question 8

About you

The next questions relate to general information about you. It would help us to know more about the background of the people completing the questionnaire.

8. Which best describes your employment status?

Please cross one box only

- Employed full-time ☐
- Employed part-time ☐
- Self employed full-time ☐
- Self employed part-time ☐
- Unemployed (seeking work) ☐
- Unemployed (not seeking work) ☐
- Retired ☐
- Student ☐
- Permanently sick/disabled ☐
- Temporarily sick/disabled ☐
- Looking after family/home ☐
- Other, please describe ____________

9. What is your highest level of qualification?

Please cross one box only

- Degree (or equivalent) ☐
- Diploma (or equivalent) ☐
- 'A' level ☐
- GCSE / 'O' level ☐
- None ☐
- Other, please specify ____________

10. How would you describe your ethnicity

Please cross one box only

White ☐
or
- White British ☐
- White Irish ☐
- Other White background ☐

Mixed ☐
or
- White & Black Carribean ☐
- White & Black African ☐
- White & Asian ☐
- Other mixed background ☐

Black or Black British ☐
or
- Carribean ☐
- African ☐
- Other Black background ☐

Asian or Asian British ☐
or
- Indian ☐
- Pakistani ☐
- Bangladeshi ☐
- Other Asian background ☐

Chinese or other ethnic group ☐
or
- Chinese ☐
- Other ethnic group ☐

11.

Do you live alone?

Yes ☐ **Please go to Question 12**

No ☐ **Please say who you live with below**

Who do you live with?
(e.g. wife, husband, partner, family member)

12. **Are you suffering from, or have you suffered from, any of the following in the past 2 years?**

Please cross any that are applicable to you

- Asthma ☐
- Chronic Obstructive Pulmonary Disease (COPD) ☐
- Other lung disease (e.g. fibrosis, bronchiectatis etc) ☐
- Heart disease ☐
- Anxiety or depression ☐
- Inflammatory bowel disease ☐
- Irritable bowel syndrome ☐
- Peptic ulcer ☐
- Previous cancer ☐
- Diabetes ☐
- Arthritis ☐

13. **About smoking**

Please cross the appropriate statement

- Are you a current smoker? ☐
- Are you an ex-smoker? ☐
- Are you a non-smoker (never smoked)? ☐

14. **Do you think you were more at risk of getting cancer because of your family history?**

Yes ☐
No ☐

15. The following questions are about how completing the questionnaire made you feel.

A number of statements which people have used to describe themselves are given below. Read each statement and then cross the most appropriate box to indicate how you feel right now, at the moment. There are no right or wrong answers. Do not spend too much time on any one statement but give the answer which seems to describe your present feeling best.

		Not at all	Somewhat	Moderately	Very much
1.	I feel calm	☐	☐	☐	☐
2.	I am tense	☐	☐	☐	☐
3.	I feel upset	☐	☐	☐	☐
4.	I am relaxed	☐	☐	☐	☐
5.	I feel content	☐	☐	☐	☐
6.	I am worried	☐	☐	☐	☐

Please make sure you have answered all the questions.

If you have any other comments or there is anything else you would like to tell us about when you first noticed your symptoms, please do so using the space below.

If you feel you would like to talk to someone about this questionnaire at a later stage, please speak to the doctor or nurse who is looking after you, or be in touch with the research team, whose contact details are on your patient information sheet

Thank you for completing the questionnaire

Please give it to the research nurse

The next page is for the Researcher to complete

57727

7 2

Researcher - Post-completion Sheet

Researcher initials: ☐☐☐

Date questionnaire completed: Date ☐☐ / ☐☐ / ☐☐ Time ☐☐:☐☐

If not completed, give reason ☐

How long is it since the patient was told of his/her diagnosis? ☐

How long did it take the patient to complete the questionnaire?

- Less than 5 minutes ☐
- Between 5 and 10 minutes ☐
- Between 11 and 15 minutes ☐
- Longer than 15 minutes ☐

For the patient selected to complete the questionnaire on their own

Did the patient ask for any help? ☐ Yes ☐ No

If yes,

please specify what help was requested

____

____

please specify what help was given

____

____

For the patient selected for the researcher-administered questionnaire

Did the patient find any of the questions difficult? ☐ Yes ☐ No

If yes,

please specify which questions and what the difficulty was

____

____

____

____

Was the patient made anxious? ☐ Yes ☐ No

If yes,

please give details

____

____

____

____

Where did the patient complete the questionnaire?

(Please specify whether this was in a separate room alone, in a corner of the clinic, the ward or other

____

Thank you. Please now send the questionnaire and patient information sheet and consent form back to Cardiff University in the freepost envelope provided.

57727

PID - 7 3

☐ GLAN CLWYD

CID - ☐ GWYNEDD

☐ MAELOR

Confidential Questionnaire

Looking At Your Symptoms Study

This Study has been funded by Cancer Research UK

Dear Patient

I am very grateful to you for agreeing to help us with this study.

I hope the information you supply will help us give people a diagnosis earlier.

If you feel you would like to talk to someone about this questionnaire at a later stage, please speak to the doctor or nurse who is looking after you, or be in touch with the research team, whose contact details are on your patient information sheet.

I can assure you that anything you tell us will be treated in the utmost confidence.

Yours sincerely

Richard Neal

Dr Richard Neal
Senior Clinical Lecturer
Cardiff University
Chief Investigator, Symptoms Study

About your symptoms

These questions are about symptoms you noticed before your diagnosis. We are interested in symptoms which you think are related to your cancer.

Please put a cross in the appropriate box, or write an answer as required.

Looking back...

1. Did you have any symptoms that you feel were due to your cancer before you were diagnosed?

Yes ☐ **Please continue with Question 2**

No ☐ **Please go to Section 2 on Page 5**

2. What was the first symptom that made you think something might be wrong?

[ ]

Please go to Question 3

The following questions are about when you first noticed a symptom and when you first told your GP or nurse about it.

Please give an exact date if you can. Otherwise give your best estimate. (For example approximately how long ago, the month or season). You wish to refer to your diary or calendar if you have it with you.

Here is an example question:

Decrease in appetite

Did you have this symptom?

Yes ☒ **Please complete A then B or C below**

No ☐ **Please go to Section 2**

A When did you first notice this? d d / m m / y y **OR** Estimate '3 months ago' or 'June'

B When did you first tell your GP or nurse? 2 3 / 1 2 / 0 7 **OR** Estimate

OR

C Put a cross here if you didn't tell your GP or nurse ☐

3. Change in bowel habit

Did you have this symptom?

Yes ☐ **Please complete A then B or C below**

No ☐ **Please go to Question 4**

A When did you first notice this? ☐☐ / ☐☐ / ☐☐ **OR** ☐

B When did you first tell your GP or nurse? ☐☐ / ☐☐ / ☐☐ **OR** ☐

OR

C Put a cross here if you didn't tell your GP or nurse ☐

4. Bleeding from back passage

Did you have this symptom?

Yes ☐ **Please complete A then B or C below**

No ☐ **Please go to Section 2**

A When did you first notice this? ☐☐ / ☐☐ / ☐☐ **OR** ☐

B When did you first tell your GP or nurse? ☐☐ / ☐☐ / ☐☐ **OR** ☐

OR

C Put a cross here if you didn't tell your GP or nurse ☐

Section 2

Please give an exact date if you can. Otherwise give your best estimate (for example approximately how long ago, the month or the season). You may wish to refer to your diary or calendar if you have it with you.

Did you have any of the following?

1. Decrease in appetite

Did you have this?

Yes ☐ **Please complete A then B or C below**

No ☐ **Please go to Question 2**

A When did you first notice this? ☐☐ / ☐☐ / ☐☐ **OR** ☐

B When did you first tell your GP or nurse? ☐☐ / ☐☐ / ☐☐ **OR** ☐

OR

C Put a cross here if you didn't tell your GP or nurse ☐

2. Unexplained weight loss

Did you have this?

Yes ☐ **Please complete A then B or C below**

No ☐ **Please go to Question 3**

A When did you first notice this? ☐☐ / ☐☐ / ☐☐ **OR** ☐

B When did you first tell your GP or nurse? ☐☐ / ☐☐ / ☐☐ **OR** ☐

OR

C Put a cross here if you didn't tell your GP or nurse ☐

3. **Fatigue or tiredness that is unusual for you**

Did you have this?

Yes ☐ **Please complete A then B or C below**

No ☐ **Please go to Question 4**

A When did you first notice this? ☐☐ / ☐☐ / ☐☐ **OR** ☐

B When did you first tell your GP or nurse? ☐☐ / ☐☐ / ☐☐ **OR** ☐

OR

C Put a cross here if you didn't tell your GP or nurse ☐

4. **Feeling different 'in yourself' from usual**

Did you have this?

Yes ☐ **Please complete A then B or C below**

No ☐ **Please go to Question 5**

A When did you first notice this? ☐☐ / ☐☐ / ☐☐ **OR** ☐

B When did you first tell your GP or nurse? ☐☐ / ☐☐ / ☐☐ **OR** ☐

OR

C Put a cross here if you didn't tell your GP or nurse ☐

Please try and describe what the feeling was

If you had any further symptoms which you feel are relevant to your cancer diagnosis, please list them below. If not, please go to Question 7.

Please describe the symptom here and complete A then B or C below

5.

**A**	When did you first notice this?	☐☐ / ☐☐ / ☐☐ **OR** ☐
**B**	When did you first tell your GP or nurse?	☐☐ / ☐☐ / ☐☐ **OR** ☐
	**OR**	
**C**	Put a cross here if you didn't tell your GP or nurse	☐

If you had another symptom you feel is relevant, please write it below, otherwise go to Question 7.

Please describe the symptom here and complete A then B or C below

6.

**A**	When did you first notice this?	☐☐ / ☐☐ / ☐☐ **OR** ☐
**B**	When did you first tell your GP or nurse?	☐☐ / ☐☐ / ☐☐ **OR** ☐
	**OR**	
**C**	Put a cross here if you didn't tell your GP or nurse	☐

If you had further symptoms you would like to tell us about, there is space at the end of the Questionnaire for you to write them.

Did your GP send you for any tests?

Thinking back to the time immediately before you diagnosis, did your GP send you for any of the following tests?

Please cross the appropriate boxes

7. Were you sent for any of these?

	Yes	Not sure	No
Blood test(s)	☐	☐	☐
CT Scan	☐	☐	☐
Ultrasound scan	☐	☐	☐
Barium Enema	☐	☐	☐
Sigmoidoscopy or colonoscopy (Looking at bowel with internal camera)	☐	☐	☐
X-ray	☐	☐	☐

Please go to Question 8

About you

The next questions relate to general information about you. It would help us to know more about the background of the people completing the questionnaire.

8. Which best describes your employment status?

Please cross one box only

- Employed full-time ☐
- Employed part-time ☐
- Self employed full-time ☐
- Self employed part-time ☐
- Unemployed (seeking work) ☐
- Unemployed (not seeking work) ☐
- Retired ☐
- Student ☐
- Permanently sick/disabled ☐
- Temporarily sick/disabled ☐
- Looking after family/home ☐
- Other, please describe ______

9. What is your highest level of qualification?

Please cross one box only

- Degree (or equivalent) ☐
- Diploma (or equivalent) ☐
- 'A' level ☐
- GCSE / 'O' level ☐
- None ☐
- Other, please specify ______

10. How would you describe your ethnicity

Please cross one box only

White ☐
or
- White British ☐
- White Irish ☐
- Other White background ☐

Mixed ☐
or
- White & Black Carribean ☐
- White & Black African ☐
- White & Asian ☐
- Other mixed background ☐

Black or Black British ☐
or
- Carribean ☐
- African ☐
- Other Black background ☐

Asian or Asian British ☐
or
- Indian ☐
- Pakistani ☐
- Bangladeshi ☐
- Other Asian background ☐

Chinese or other ethnic group ☐
or
- Chinese ☐
- Other ethnic group ☐

11.

Do you live alone?

Yes ☐ **Please go to Question 12**

No ☐ **Please say who you live with below**

Who do you live with?
(e.g. wife, husband, partner, family member)

12. **Are you suffering from, or have you suffered from, any of the following in the past 2 years?**

Please cross any that are applicable to you

- Asthma ☐
- Chronic Obstructive Pulmonary Disease (COPD) ☐
- Other lung disease (e.g. fibrosis, bronchiectatis etc) ☐
- Heart disease ☐
- Anxiety or depression ☐
- Inflammatory bowel disease ☐
- Irritable bowel syndrome ☐
- Peptic ulcer ☐
- Previous cancer ☐
- Diabetes ☐
- Arthritis ☐

13. **About smoking**

Please cross the appropriate statement

- Are you a current smoker? ☐
- Are you an ex-smoker? ☐
- Are you a non-smoker (never smoked)? ☐

14. **Do you think you were more at risk of getting cancer because of your family history?**

Yes ☐
No ☐

15. The following questions are about how completing the questionnaire made you feel.

A number of statements which people have used to describe themselves are given below. Read each statement and then cross the most appropriate box to indicate how you feel right now, at the moment. There are no right or wrong answers. Do not spend too much time on any one statement but give the answer which seems to describe your present feeling best.

		Not at all	Somewhat	Moderately	Very much
1.	I feel calm	☐	☐	☐	☐
2.	I am tense	☐	☐	☐	☐
3.	I feel upset	☐	☐	☐	☐
4.	I am relaxed	☐	☐	☐	☐
5.	I feel content	☐	☐	☐	☐
6.	I am worried	☐	☐	☐	☐

Please make sure you have answered all the questions.

If you have any other comments or there is anything else you would like to tell us about when you first noticed your symptoms, please do so using the space below.

If you feel you would like to talk to someone about this questionnaire at a later stage, please speak to the doctor or nurse who is looking after you, or be in touch with the research team, whose contact details are on your patient information sheet

Thank you for completing the questionnaire

Please give it to the research nurse

The next page is for the Researcher to complete

Researcher - Post-completion Sheet

Researcher initials: ☐☐☐

Date questionnaire completed: Date ☐☐ / ☐☐ / ☐☐ Time ☐☐:☐☐

If not completed, give reason ☐

How long is it since the patient was told of his/her diagnosis? ☐

How long did it take the patient to complete the questionnaire?

- Less than 5 minutes ☐
- Between 5 and 10 minutes ☐
- Between 11 and 15 minutes ☐
- Longer than 15 minutes ☐

For the patient selected to <u>complete the questionnaire on their own</u>

Did the patient ask for any help? ☐ Yes ☐ No

If yes,

please specify what help was requested

____

____

please specify what help was given

____

____

For the patient selected for <u>the researcher-administered questionnaire</u>

Did the patient find any of the questions difficult? ☐ Yes ☐ No

If yes,

please specify which questions and what the difficulty was

____

____

____

____

Was the patient made anxious? ☐ Yes ☐ No

If yes,

please give details

____

____

____

____

Where did the patient complete the questionnaire?

(Please specify whether this was in a separate room alone, in a corner of the clinic, the ward or other

____

Thank you. Please now send the questionnaire and patient information sheet and consent form back to Cardiff University in the freepost envelope provided.

57727

PID - | | 7 | 4 |

Ymddiriedolaeth GIG Gogledd Orllewin Cymru
North West Wales NHS Trust

NHS WALES GIG CYMRU
Ymddiriedolaeth GIG Gogledd Cymru
North Wales NHS Trust

☐ GLAN CLWYD

CID - ☐ GWYNEDD

☐ MAELOR

Confidential Questionnaire

Looking At Your Symptoms Study

This Study has been funded by Cancer Research UK

CARDIFF UNIVERSITY PRIFYSGOL CAERDYDD

Ysgol Glinigol Gogledd Cymru North Wales Clinical School

CANCER RESEARCH UK

sewtu South East Wales Trials Unit Uned Ymchwil De-ddwyrain Cymru

Dear Patient

I am very grateful to you for agreeing to help us with this study.

I hope the information you supply will help us give people a diagnosis earlier.

If you feel you would like to talk to someone about this questionnaire at a later stage, please speak to the doctor or nurse who is looking after you, or be in touch with the research team, whose contact details are on your patient information sheet.

I can assure you that anything you tell us will be treated in the utmost confidence.

Yours sincerely

Dr Richard Neal
Senior Clinical Lecturer
Cardiff University
Chief Investigator, Symptoms Study

About your symptoms

These questions are about symptoms you noticed before your diagnosis. We are interested in symptoms which you think are related to your cancer.

Please put a cross in the appropriate box, or write an answer as required.

Looking back...

1. Did you have any symptoms that you feel were due to your cancer before you were diagnosed?

Yes ☐ **Please continue with Question 2**

No ☐ **Please go to Section 2 on Page 5**

2. What was the first symptom that made you think something might be wrong?

[ ]

Please go to Question 3

The following questions are about when you first noticed a symptom and when you first told your GP or nurse about it.

Please give an exact date if you can. Otherwise give your best estimate. (For example approximately how long ago, the month or season). You wish to refer to your diary or calendar if you have it with you.

Here is an example question:

Decrease in appetite

Did you have this symptom?

Yes ☒ **Please complete A then B or C below**

No ☐ **Please go to Section 2**

A When did you first notice this? d d / m m / y y **OR** Estimate '3 months ago' or 'June'

B When did you first tell your GP or nurse? 2 3 / 1 2 / 0 7 **OR** Estimate

OR

C Put a cross here if you didn't tell your GP or nurse ☐

3. Change in bowel habit

Did you have this symptom?

Yes ☐ **Please complete A then B or C below**

No ☐ **Please go to Question 4**

A When did you first notice this? ☐☐ / ☐☐ / ☐☐ **OR** ☐

B When did you first tell your GP or nurse? ☐☐ / ☐☐ / ☐☐ **OR** ☐

OR

C Put a cross here if you didn't tell your GP or nurse ☐

4. Bleeding from back passage

Did you have this symptom?

Yes ☐ **Please complete A then B or C below**

No ☐ **Please go to Section 2**

A When did you first notice this? ☐☐ / ☐☐ / ☐☐ **OR** ☐

B When did you first tell your GP or nurse? ☐☐ / ☐☐ / ☐☐ **OR** ☐

OR

C Put a cross here if you didn't tell your GP or nurse ☐

Section 2

Please give an exact date if you can. Otherwise give your best estimate (for example approximately how long ago, the month or the season). You may wish to refer to your diary or calendar if you have it with you.

Did you have any of the following?

1. Decrease in appetite

Did you have this?

Yes ☐ **Please complete A then B or C below**

No ☐ **Please go to Question 2**

A When did you first notice this? ☐☐ / ☐☐ / ☐☐ **OR** ☐

B When did you first tell your GP or nurse? ☐☐ / ☐☐ / ☐☐ **OR** ☐

OR

C Put a cross here if you didn't tell your GP or nurse ☐

2. Unexplained weight loss

Did you have this?

Yes ☐ **Please complete A then B or C below**

No ☐ **Please go to Question 3**

A When did you first notice this? ☐☐ / ☐☐ / ☐☐ **OR** ☐

B When did you first tell your GP or nurse? ☐☐ / ☐☐ / ☐☐ **OR** ☐

OR

C Put a cross here if you didn't tell your GP or nurse ☐

3. Fatigue or tiredness that is unusual for you

Did you have this?

Yes ☐ **Please complete A then B or C below**

No ☐ **Please go to Question 4**

A When did you first notice this? ☐☐ / ☐☐ / ☐☐ **OR** ☐

B When did you first tell your GP or nurse? ☐☐ / ☐☐ / ☐☐ **OR** ☐

OR

C Put a cross here if you didn't tell your GP or nurse ☐

4. Feeling different 'in yourself' from usual

Did you have this?

Yes ☐ **Please complete A then B or C below**

No ☐ **Please go to Question 5**

A When did you first notice this? ☐☐ / ☐☐ / ☐☐ **OR** ☐

B When did you first tell your GP or nurse? ☐☐ / ☐☐ / ☐☐ **OR** ☐

OR

C Put a cross here if you didn't tell your GP or nurse ☐

Please try and describe what the feeling was

If you had any further symptoms which you feel are relevant to your cancer diagnosis, please list them below. If not, please go to Question 7.

Please describe the symptom here and complete A then B or C below

5.

A When did you first notice this? ☐☐ / ☐☐ / ☐☐ **OR** ☐

B When did you first tell your GP or nurse? ☐☐ / ☐☐ / ☐☐ **OR** ☐

OR

C Put a cross here if you didn't tell your GP or nurse ☐

If you had another symptom you feel is relevant, please write it below, otherwise go to Question 7.

Please describe the symptom here and complete A then B or C below

6.

A When did you first notice this? ☐☐ / ☐☐ / ☐☐ **OR** ☐

B When did you first tell your GP or nurse? ☐☐ / ☐☐ / ☐☐ **OR** ☐

OR

C Put a cross here if you didn't tell your GP or nurse ☐

If you had further symptoms you would like to tell us about, there is space at the end of the Questionnaire for you to write them.

Did your GP send you for any tests?

Thinking back to the time immediately before you diagnosis, did your GP send you for any of the following tests?

Please cross the appropriate boxes

7. Were you sent for any of these?

	Yes	Not sure	No
Blood test(s)	☐	☐	☐
CT Scan	☐	☐	☐
Ultrasound scan	☐	☐	☐
Barium Enema	☐	☐	☐
Sigmoidoscopy or colonoscopy (Looking at bowel with internal camera)	☐	☐	☐
X-ray	☐	☐	☐

Please go to Question 8

About you

The next questions relate to general information about you. It would help us to know more about the background of the people completing the questionnaire.

8. Which best describes your employment status?

Please cross one box only

- Employed full-time ☐
- Employed part-time ☐
- Self employed full-time ☐
- Self employed part-time ☐
- Unemployed (seeking work) ☐
- Unemployed (not seeking work) ☐
- Retired ☐
- Student ☐
- Permanently sick/disabled ☐
- Temporarily sick/disabled ☐
- Looking after family/home ☐

Other, please describe ____________

9. What is your highest level of qualification?

Please cross one box only

- Degree (or equivalent) ☐
- Diploma (or equivalent) ☐
- 'A' level ☐
- GCSE / 'O' level ☐
- None ☐

Other, please specify ____________

10. **How would you describe your ethnicity**

Please cross one box only

White ☐
or
- White British ☐
- White Irish ☐
- Other White background ☐

Mixed ☐
or
- White & Black Carribean ☐
- White & Black African ☐
- White & Asian ☐
- Other mixed background ☐

Black or Black British ☐
or
- Carribean ☐
- African ☐
- Other Black background ☐

Asian or Asian British ☐
or
- Indian ☐
- Pakistani ☐
- Bangladeshi ☐
- Other Asian background ☐

Chinese or other ethnic group ☐
or
- Chinese ☐
- Other ethnic group ☐

11.

Do you live alone?

Yes ☐ **Please go to Question 12**

No ☐ **Please say who you live with below**

Who do you live with?
(e.g. wife, husband, partner, family member)

12. **Are you suffering from, or have you suffered from, any of the following in the past 2 years?**

Please cross any that are applicable to you

- Asthma ☐
- Chronic Obstructive Pulmonary Disease (COPD) ☐
- Other lung disease (e.g. fibrosis, bronchiectatis etc) ☐
- Heart disease ☐
- Anxiety or depression ☐
- Inflammatory bowel disease ☐
- Irritable bowel syndrome ☐
- Peptic ulcer ☐
- Previous cancer ☐
- Diabetes ☐
- Arthritis ☐

13. **About smoking**

Please cross the appropriate statement

- Are you a current smoker? ☐
- Are you an ex-smoker? ☐
- Are you a non-smoker (never smoked)? ☐

14. **Do you think you were more at risk of getting cancer because of your family history?**

Yes ☐
No ☐

15. **The following questions are about how completing the questionnaire made you feel.**

A number of statements which people have used to describe themselves are given below. Read each statement and then cross the most appropriate box to indicate how you feel right now, at the moment. There are no right or wrong answers. Do not spend too much time on any one statement but give the answer which seems to describe your present feeling best.

		Not at all	Somewhat	Moderately	Very much
1.	I feel calm	☐	☐	☐	☐
2.	I am tense	☐	☐	☐	☐
3.	I feel upset	☐	☐	☐	☐
4.	I am relaxed	☐	☐	☐	☐
5.	I feel content	☐	☐	☐	☐
6.	I am worried	☐	☐	☐	☐

Please make sure you have answered all the questions.

If you have any other comments or there is anything else you would like to tell us about when you first noticed your symptoms, please do so using the space below.

If you feel you would like to talk to someone about this questionnaire at a later stage, please speak to the doctor or nurse who is looking after you, or be in touch with the research team, whose contact details are on your patient information sheet

Thank you for completing the questionnaire

Please give it to the research nurse

The next page is for the Researcher to complete

57727

7 4

Researcher - Post-completion Sheet

Researcher initials: [ ][ ][ ]

Date questionnaire completed: Date [ ][ ] / [ ][ ] / [ ][ ] Time [ ][ ] : [ ][ ]

If not completed, give reason

How long is it since the patient was told of his/her diagnosis?

How long did it take the patient to complete the questionnaire?

- Less than 5 minutes ☐
- Between 5 and 10 minutes ☐
- Between 11 and 15 minutes ☐
- Longer than 15 minutes ☐

For the patient selected to <u>complete the questionnaire on their own</u>

Did the patient ask for any help? ☐ Yes ☐ No

If yes,

please specify what help was requested

____

____

please specify what help was given

____

____

For the patient selected for <u>the researcher-administered questionnaire</u>

Did the patient find any of the questions difficult? ☐ Yes ☐ No

If yes,

please specify which questions and what the difficulty was

____

____

____

____

Was the patient made anxious? ☐ Yes ☐ No

If yes,

please give details

____

____

____

____

Where did the patient complete the questionnaire?

(Please specify whether this was in a separate room alone, in a corner of the clinic, the ward or other

____

Thank you. Please now send the questionnaire and patient information sheet and consent form back to Cardiff University in the freepost envelope provided.

57727

PID - | | 7 | 5 |

Ymddiriedolaeth GIG Gogledd Orllewin Cymru
North West Wales NHS Trust

NHS WALES GIG CYMRU
Ymddiriedolaeth GIG Gogledd Cymru
North Wales NHS Trust

☐ GLAN CLWYD

CID - ☐ GWYNEDD

☐ MAELOR

Confidential Questionnaire

Looking At Your Symptoms Study

This Study has been funded by Cancer Research UK

Cardiff University / Prifysgol Caerdydd
Ysgol Glinigol Gogledd Cymru / North Wales Clinical School
Cancer Research UK
sewtu — South East Wales Trials Unit / Uned Ymchwil De-ddwyrain Cymru

Dear Patient

I am very grateful to you for agreeing to help us with this study.

I hope the information you supply will help us give people a diagnosis earlier.

If you feel you would like to talk to someone about this questionnaire at a later stage, please speak to the doctor or nurse who is looking after you, or be in touch with the research team, whose contact details are on your patient information sheet.

I can assure you that anything you tell us will be treated in the utmost confidence.

Yours sincerely

Dr Richard Neal
Senior Clinical Lecturer
Cardiff University
Chief Investigator, Symptoms Study

About your symptoms

These questions are about symptoms you noticed before your diagnosis. We are interested in symptoms which you think are related to your cancer.

Please put a cross in the appropriate box, or write an answer as required.

Looking back...

1. Did you have any symptoms that you feel were due to your cancer before you were diagnosed?

- Yes ☐ **Please continue with Question 2**
- No ☐ **Please go to Section 2 on Page 5**

2. What was the first symptom that made you think something might be wrong?

[ ]

Please go to Question 3

The following questions are about when you first noticed a symptom and when you first told your GP or nurse about it.

Please give an exact date if you can. Otherwise give your best estimate. (For example approximately how long ago, the month or season). You wish to refer to your diary or calendar if you have it with you.

Here is an example question:

Decrease in appetite

Did you have this symptom?

- Yes ☒ **Please complete A then B or C below**
- No ☐ **Please go to Section 2**

A When did you first notice this? d d / m m / y y **OR** Estimate: '3 months ago' or 'June'

B When did you first tell your GP or nurse? 2 3 / 1 2 / 0 7 **OR** Estimate

OR

C Put a cross here if you didn't tell your GP or nurse ☐

3. Change in bowel habit

Did you have this symptom?

Yes ☐ **Please complete A then B or C below**

No ☐ **Please go to Question 4**

A When did you first notice this? ☐☐ / ☐☐ / ☐☐ **OR** ☐

B When did you first tell your GP or nurse? ☐☐ / ☐☐ / ☐☐ **OR** ☐

OR

C Put a cross here if you didn't tell your GP or nurse ☐

4. Bleeding from back passage

Did you have this symptom?

Yes ☐ **Please complete A then B or C below**

No ☐ **Please go to Section 2**

A When did you first notice this? ☐☐ / ☐☐ / ☐☐ **OR** ☐

B When did you first tell your GP or nurse? ☐☐ / ☐☐ / ☐☐ **OR** ☐

OR

C Put a cross here if you didn't tell your GP or nurse ☐

Section 2

Please give an exact date if you can. Otherwise give your best estimate (for example approximately how long ago, the month or the season). You may wish to refer to your diary or calendar if you have it with you.

Did you have any of the following?

1. Decrease in appetite

Did you have this?

Yes ☐ **Please complete A then B or C below**

No ☐ **Please go to Question 2**

A When did you first notice this? ☐☐ / ☐☐ / ☐☐ **OR** ☐

B When did you first tell your GP or nurse? ☐☐ / ☐☐ / ☐☐ **OR** ☐

OR

C Put a cross here if you didn't tell your GP or nurse ☐

2. Unexplained weight loss

Did you have this?

Yes ☐ **Please complete A then B or C below**

No ☐ **Please go to Question 3**

A When did you first notice this? ☐☐ / ☐☐ / ☐☐ **OR** ☐

B When did you first tell your GP or nurse? ☐☐ / ☐☐ / ☐☐ **OR** ☐

OR

C Put a cross here if you didn't tell your GP or nurse ☐

3. **Fatigue or tiredness that is unusual for you**

Did you have this?

Yes ☐ **Please complete A then B or C below**

No ☐ **Please go to Question 4**

A When did you first notice this? ☐☐ / ☐☐ / ☐☐ **OR** ☐

B When did you first tell your GP or nurse? ☐☐ / ☐☐ / ☐☐ **OR** ☐

OR

C Put a cross here if you didn't tell your GP or nurse ☐

4. **Feeling different 'in yourself' from usual**

Did you have this?

Yes ☐ **Please complete A then B or C below**

No ☐ **Please go to Question 5**

A When did you first notice this? ☐☐ / ☐☐ / ☐☐ **OR** ☐

B When did you first tell your GP or nurse? ☐☐ / ☐☐ / ☐☐ **OR** ☐

OR

C Put a cross here if you didn't tell your GP or nurse ☐

Please try and describe what the feeling was

If you had any further symptoms which you feel are relevant to your cancer diagnosis, please list them below. If not, please go to Question 7.

Please describe the symptom here and complete A then B or C below

5.

A When did you first notice this? __/__/__ OR

B When did you first tell your GP or nurse? __/__/__ OR

OR

C Put a cross here if you didn't tell your GP or nurse ☐

If you had another symptom you feel is relevant, please write it below, otherwise go to Question 7.

Please describe the symptom here and complete A then B or C below

6.

A When did you first notice this? __/__/__ OR

B When did you first tell your GP or nurse? __/__/__ OR

OR

C Put a cross here if you didn't tell your GP or nurse ☐

If you had further symptoms you would like to tell us about, there is space at the end of the Questionnaire for you to write them.

Did your GP send you for any tests?

Thinking back to the time immediately before you diagnosis, did your GP send you for any of the following tests?

Please cross the appropriate boxes

7. Were you sent for any of these?

	Yes	Not sure	No
Blood test(s)	☐	☐	☐
CT Scan	☐	☐	☐
Ultrasound scan	☐	☐	☐
Barium Enema	☐	☐	☐
Sigmoidoscopy or colonoscopy (Looking at bowel with internal camera)	☐	☐	☐
X-ray	☐	☐	☐

Please go to Question 8

About you

The next questions relate to general information about you. It would help us to know more about the background of the people completing the questionnaire.

8. Which best describes your employment status?

Please cross one box only

- Employed full-time ☐
- Employed part-time ☐
- Self employed full-time ☐
- Self employed part-time ☐
- Unemployed (seeking work) ☐
- Unemployed (not seeking work) ☐
- Retired ☐
- Student ☐
- Permanently sick/disabled ☐
- Temporarily sick/disabled ☐
- Looking after family/home ☐
- Other, please describe ______

9. What is your highest level of qualification?

Please cross one box only

- Degree (or equivalent) ☐
- Diploma (or equivalent) ☐
- 'A' level ☐
- GCSE / 'O' level ☐
- None ☐
- Other, please specify ______

10. How would you describe your ethnicity

Please cross one box only

White ☐
or
- White British ☐
- White Irish ☐
- Other White background ☐

Mixed ☐
or
- White & Black Carribean ☐
- White & Black African ☐
- White & Asian ☐
- Other mixed background ☐

Black or Black British ☐
or
- Carribean ☐
- African ☐
- Other Black background ☐

Asian or Asian British ☐
or
- Indian ☐
- Pakistani ☐
- Bangladeshi ☐
- Other Asian background ☐

Chinese or other ethnic group ☐
or
- Chinese ☐
- Other ethnic group ☐

11.

Do you live alone?

Yes ☐ **Please go to Question 12**

No ☐ **Please say who you live with below**

Who do you live with?
(e.g. wife, husband, partner, family member)

☐

12. Are you suffering from, or have you suffered from, any of the following in the past 2 years?

Please cross any that are applicable to you

- Asthma ☐
- Chronic Obstructive Pulmonary Disease (COPD) ☐
- Other lung disease (e.g. fibrosis, bronchiectatis etc) ☐
- Heart disease ☐
- Anxiety or depression ☐
- Inflammatory bowel disease ☐
- Irritable bowel syndrome ☐
- Peptic ulcer ☐
- Previous cancer ☐
- Diabetes ☐
- Arthritis ☐

13. About smoking

Please cross the appropriate statement

- Are you a current smoker? ☐
- Are you an ex-smoker? ☐
- Are you a non-smoker (never smoked)? ☐

14. Do you think you were more at risk of getting cancer because of your family history?

Yes ☐
No ☐

15. **The following questions are about how completing the questionnaire made you feel.**

A number of statements which people have used to describe themselves are given below. Read each statement and then cross the most appropriate box to indicate how you feel right now, at the moment. There are no right or wrong answers. Do not spend too much time on any one statement but give the answer which seems to describe your present feeling best.

		Not at all	Somewhat	Moderately	Very much
1.	I feel calm	☐	☐	☐	☐
2.	I am tense	☐	☐	☐	☐
3.	I feel upset	☐	☐	☐	☐
4.	I am relaxed	☐	☐	☐	☐
5.	I feel content	☐	☐	☐	☐
6.	I am worried	☐	☐	☐	☐

Please make sure you have answered all the questions.

If you have any other comments or there is anything else you would like to tell us about when you first noticed your symptoms, please do so using the space below.

If you feel you would like to talk to someone about this questionnaire at a later stage, please speak to the doctor or nurse who is looking after you, or be in touch with the research team, whose contact details are on your patient information sheet

Thank you for completing the questionnaire

Please give it to the research nurse

The next page is for the Researcher to complete

57727

Researcher - Post-completion Sheet

Researcher initials: ☐☐☐

Date questionnaire completed: Date ☐☐ / ☐☐ / ☐☐ Time ☐☐:☐☐

If not completed, give reason ☐

How long is it since the patient was told of his/her diagnosis? ☐

How long did it take the patient to complete the questionnaire?

- Less than 5 minutes ☐
- Between 5 and 10 minutes ☐
- Between 11 and 15 minutes ☐
- Longer than 15 minutes ☐

For the patient selected to complete the questionnaire on their own

Did the patient ask for any help? ☐ Yes ☐ No

If yes,

please specify what help was requested

____

____

please specify what help was given

____

____

For the patient selected for the researcher-administered questionnaire

Did the patient find any of the questions difficult? ☐ Yes ☐ No

If yes,

please specify which questions and what the difficulty was

____

____

____

____

Was the patient made anxious? ☐ Yes ☐ No

If yes,

please give details

____

____

____

____

Where did the patient complete the questionnaire?

(Please specify whether this was in a separate room alone, in a corner of the clinic, the ward or other

____

Thank you. Please now send the questionnaire and patient information sheet and consent form back to Cardiff University in the freepost envelope provided.

57727

PID - | | 7 | 6 |

Ymddiriedolaeth GIG Gogledd Orllewin Cymru
North West Wales NHS Trust

NHS WALES GIG CYMRU
Ymddiriedolaeth GIG Gogledd Cymru
North Wales NHS Trust

CID - ☐ GLAN CLWYD
☐ GWYNEDD
☐ MAELOR

Confidential Questionnaire

Looking At Your Symptoms Study

This Study has been funded by Cancer Research UK

CARDIFF UNIVERSITY PRIFYSGOL CAERDYDD
Ysgol Glinigol Gogledd Cymru North Wales Clinical School
CANCER RESEARCH UK
sewtu South East Wales Trials Unit Uned Ymchwil De-ddwyrain Cymru

Dear Patient

I am very grateful to you for agreeing to help us with this study.

I hope the information you supply will help us give people a diagnosis earlier.

If you feel you would like to talk to someone about this questionnaire at a later stage, please speak to the doctor or nurse who is looking after you, or be in touch with the research team, whose contact details are on your patient information sheet.

I can assure you that anything you tell us will be treated in the utmost confidence.

Yours sincerely

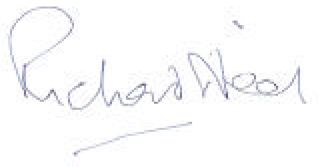

Dr Richard Neal
Senior Clinical Lecturer
Cardiff University
Chief Investigator, Symptoms Study

About your symptoms

These questions are about symptoms you noticed before your diagnosis. We are interested in symptoms which you think are related to your cancer.

Please put a cross in the appropriate box, or write an answer as required.

Looking back...

1. Did you have any symptoms that you feel were due to your cancer before you were diagnosed?

Yes ☐ **Please continue with Question 2**

No ☐ **Please go to Section 2 on Page 5**

2. What was the first symptom that made you think something might be wrong?

☐

Please go to Question 3

The following questions are about when you first noticed a symptom and when you first told your GP or nurse about it.

Please give an exact date if you can. Otherwise give your best estimate. (For example approximately how long ago, the month or season). You wish to refer to your diary or calendar if you have it with you.

Here is an example question:

Decrease in appetite

Did you have this symptom?

Yes ☒ **Please complete A then B or C below**

No ☐ **Please go to Section 2**

A When did you first notice this? d d / m m / y y **OR** Estimate '3 months ago' or 'June'

B When did you first tell your GP or nurse? 2 3 / 1 2 / 0 7 **OR** Estimate

OR

C Put a cross here if you didn't tell your GP or nurse ☐

3. Change in bowel habit

Did you have this symptom?

Yes ☐ **Please complete A then B or C below**

No ☐ **Please go to Question 4**

A When did you first notice this? ☐☐ / ☐☐ / ☐☐ **OR** ☐

B When did you first tell your GP or nurse? ☐☐ / ☐☐ / ☐☐ **OR** ☐

OR

C Put a cross here if you didn't tell your GP or nurse ☐

4. Bleeding from back passage

Did you have this symptom?

Yes ☐ **Please complete A then B or C below**

No ☐ **Please go to Section 2**

A When did you first notice this? ☐☐ / ☐☐ / ☐☐ **OR** ☐

B When did you first tell your GP or nurse? ☐☐ / ☐☐ / ☐☐ **OR** ☐

OR

C Put a cross here if you didn't tell your GP or nurse ☐

Section 2

Please give an exact date if you can. Otherwise give your best estimate (for example approximately how long ago, the month or the season). You may wish to refer to your diary or calendar if you have it with you.

Did you have any of the following?

1. Decrease in appetite

Did you have this?

Yes ☐ **Please complete A then B or C below**

No ☐ **Please go to Question 2**

A When did you first notice this? ☐☐ / ☐☐ / ☐☐ **OR** ☐

B When did you first tell your GP or nurse? ☐☐ / ☐☐ / ☐☐ **OR** ☐

OR

C Put a cross here if you didn't tell your GP or nurse ☐

2. Unexplained weight loss

Did you have this?

Yes ☐ **Please complete A then B or C below**

No ☐ **Please go to Question 3**

A When did you first notice this? ☐☐ / ☐☐ / ☐☐ **OR** ☐

B When did you first tell your GP or nurse? ☐☐ / ☐☐ / ☐☐ **OR** ☐

OR

C Put a cross here if you didn't tell your GP or nurse ☐

3. Fatigue or tiredness that is unusual for you

Did you have this?

Yes ☐ **Please complete A then B or C below**

No ☐ **Please go to Question 4**

A When did you first notice this? ☐☐ / ☐☐ / ☐☐ **OR** ☐

B When did you first tell your GP or nurse? ☐☐ / ☐☐ / ☐☐ **OR** ☐

OR

C Put a cross here if you didn't tell your GP or nurse ☐

4. Feeling different 'in yourself' from usual

Did you have this?

Yes ☐ **Please complete A then B or C below**

No ☐ **Please go to Question 5**

A When did you first notice this? ☐☐ / ☐☐ / ☐☐ **OR** ☐

B When did you first tell your GP or nurse? ☐☐ / ☐☐ / ☐☐ **OR** ☐

OR

C Put a cross here if you didn't tell your GP or nurse ☐

Please try and describe what the feeling was

If you had any further symptoms which you feel are relevant to your cancer diagnosis, please list them below. If not, please go to Question 7.

Please describe the symptom here and complete A then B or C below

5.

A When did you <u>first notice</u> this? ☐☐ / ☐☐ / ☐☐ **OR** ☐

B When did you <u>first tell</u> your GP or nurse? ☐☐ / ☐☐ / ☐☐ **OR** ☐

OR

C Put a cross here if you <u>didn't tell</u> your GP or nurse ☐

If you had another symptom you feel is relevant, please write it below, otherwise go to Question 7.

Please describe the symptom here and complete A then B or C below

6.

A When did you <u>first notice</u> this? ☐☐ / ☐☐ / ☐☐ **OR** ☐

B When did you <u>first tell</u> your GP or nurse? ☐☐ / ☐☐ / ☐☐ **OR** ☐

OR

C Put a cross here if you <u>didn't tell</u> your GP or nurse ☐

If you had further symptoms you would like to tell us about, there is space at the end of the Questionnaire for you to write them.

Did your GP send you for any tests?

Thinking back to the time immediately before you diagnosis, did your GP send you for any of the following tests?

Please cross the appropriate boxes

7. Were you sent for any of these?

	Yes	Not sure	No
Blood test(s)	☐	☐	☐
CT Scan	☐	☐	☐
Ultrasound scan	☐	☐	☐
Barium Enema	☐	☐	☐
Sigmoidoscopy or colonoscopy (Looking at bowel with internal camera)	☐	☐	☐
X-ray	☐	☐	☐

Please go to Question 8

About you

The next questions relate to general information about you. It would help us to know more about the background of the people completing the questionnaire.

8. Which best describes your employment status?

Please cross one box only

- Employed full-time ☐
- Employed part-time ☐
- Self employed full-time ☐
- Self employed part-time ☐
- Unemployed (seeking work) ☐
- Unemployed (not seeking work) ☐
- Retired ☐
- Student ☐
- Permanently sick/disabled ☐
- Temporarily sick/disabled ☐
- Looking after family/home ☐
- Other, please describe ________

9. What is your highest level of qualification?

Please cross one box only

- Degree (or equivalent) ☐
- Diploma (or equivalent) ☐
- 'A' level ☐
- GCSE / 'O' level ☐
- None ☐
- Other, please specify ________

10. How would you describe your ethnicity

Please cross one box only

White ☐
or
- White British ☐
- White Irish ☐
- Other White background ☐

Mixed ☐
or
- White & Black Carribean ☐
- White & Black African ☐
- White & Asian ☐
- Other mixed background ☐

Black or Black British ☐
or
- Carribean ☐
- African ☐
- Other Black background ☐

Asian or Asian British ☐
or
- Indian ☐
- Pakistani ☐
- Bangladeshi ☐
- Other Asian background ☐

Chinese or other ethnic group ☐
or
- Chinese ☐
- Other ethnic group ☐

11.

Do you live alone?

Yes ☐ **Please go to Question 12**

No ☐ **Please say who you live with below**

Who do you live with?
(e.g. wife, husband, partner, family member)

12. Are you suffering from, or have you suffered from, any of the following in the past 2 years?

Please cross any that are applicable to you

- Asthma ☐
- Chronic Obstructive Pulmonary Disease (COPD) ☐
- Other lung disease (e.g. fibrosis, bronchiectatis etc) ☐
- Heart disease ☐
- Anxiety or depression ☐
- Inflammatory bowel disease ☐
- Irritable bowel syndrome ☐
- Peptic ulcer ☐
- Previous cancer ☐
- Diabetes ☐
- Arthritis ☐

13. About smoking

Please cross the appropriate statement

- Are you a current smoker? ☐
- Are you an ex-smoker? ☐
- Are you a non-smoker (never smoked)? ☐

14. Do you think you were more at risk of getting cancer because of your family history?

Yes ☐
No ☐

15. **The following questions are about how completing the questionnaire made you feel.**

A number of statements which people have used to describe themselves are given below. Read each statement and then cross the most appropriate box to indicate how you feel right now, at the moment. There are no right or wrong answers. Do not spend too much time on any one statement but give the answer which seems to describe your present feeling best.

		Not at all	Somewhat	Moderately	Very much
1.	I feel calm	☐	☐	☐	☐
2.	I am tense	☐	☐	☐	☐
3.	I feel upset	☐	☐	☐	☐
4.	I am relaxed	☐	☐	☐	☐
5.	I feel content	☐	☐	☐	☐
6.	I am worried	☐	☐	☐	☐

Please make sure you have answered all the questions.

If you have any other comments or there is anything else you would like to tell us about when you first noticed your symptoms, please do so using the space below.

If you feel you would like to talk to someone about this questionnaire at a later stage, please speak to the doctor or nurse who is looking after you, or be in touch with the research team, whose contact details are on your patient information sheet

Thank you for completing the questionnaire

Please give it to the research nurse

The next page is for the Researcher to complete

Researcher - Post-completion Sheet **Researcher initials:** ☐☐☐

Date questionnaire completed: Date ☐☐ / ☐☐ / ☐☐ Time ☐☐:☐☐

If not completed, give reason ☐

How long is it since the patient was told of his/her diagnosis? ☐

How long did it take the patient to complete the questionnaire?

- Less than 5 minutes ☐
- Between 5 and 10 minutes ☐
- Between 11 and 15 minutes ☐
- Longer than 15 minutes ☐

For the patient selected to complete the questionnaire on their own

Did the patient ask for any help? ☐ Yes ☐ No

If yes,

please specify what help was requested

____

____

please specify what help was given

____

____

For the patient selected for the researcher-administered questionnaire

Did the patient find any of the questions difficult? ☐ Yes ☐ No

If yes,

please specify which questions and what the difficulty was

____

____

____

____

Was the patient made anxious? ☐ Yes ☐ No

If yes,

please give details

____

____

____

____

Where did the patient complete the questionnaire?
(Please specify whether this was in a separate room alone, in a corner of the clinic, the ward or other

____

Thank you. Please now send the questionnaire and patient information sheet and consent form back to Cardiff University in the freepost envelope provided.

57727

PID - | | 7 | 7 |

Ymddiriedolaeth GIG Gogledd Orllewin Cymru
North West Wales NHS Trust

NHS WALES GIG CYMRU
Ymddiriedolaeth GIG Gogledd Cymru
North Wales NHS Trust

CID -
- ☐ GLAN CLWYD
- ☐ GWYNEDD
- ☐ MAELOR

Confidential Questionnaire

Looking At Your Symptoms Study

This Study has been funded by Cancer Research UK

Cardiff University / Prifysgol Caerdydd
Ysgol Glinigol Gogledd Cymru / North Wales Clinical School
Cancer Research UK
sewtu South East Wales Trials Unit / Uned Ymchwil De-ddwyrain Cymru

Dear Patient

I am very grateful to you for agreeing to help us with this study.

I hope the information you supply will help us give people a diagnosis earlier.

If you feel you would like to talk to someone about this questionnaire at a later stage, please speak to the doctor or nurse who is looking after you, or be in touch with the research team, whose contact details are on your patient information sheet.

I can assure you that anything you tell us will be treated in the utmost confidence.

Yours sincerely

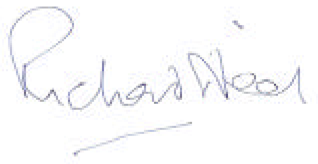

Dr Richard Neal
Senior Clinical Lecturer
Cardiff University
Chief Investigator, Symptoms Study

About your symptoms

These questions are about symptoms you noticed before your diagnosis. We are interested in symptoms which you think are related to your cancer.

Please put a cross in the appropriate box, or write an answer as required.

Looking back...

1. Did you have any symptoms that you feel were due to your cancer before you were diagnosed?

Yes ☐ **Please continue with Question 2**

No ☐ **Please go to Section 2 on Page 5**

2. What was the first symptom that made you think something might be wrong?

☐

Please go to Question 3

The following questions are about when you first noticed a symptom and when you first told your GP or nurse about it.

Please give an exact date if you can. Otherwise give your best estimate. (For example approximately how long ago, the month or season). You wish to refer to your diary or calendar if you have it with you.

Here is an example question:

Decrease in appetite

Did you have this symptom?

Yes ☒ **Please complete A then B or C below**

No ☐ **Please go to Section 2**

A When did you first notice this? d d / m m / y y **OR** Estimate '3 months ago' or 'June'

B When did you first tell your GP or nurse? 2 3 / 1 2 / 0 7 **OR** Estimate

OR

C Put a cross here if you didn't tell your GP or nurse ☐

3. Change in bowel habit

Did you have this symptom?

Yes ☐ **Please complete A then B or C below**

No ☐ **Please go to Question 4**

A When did you first notice this? ☐☐ / ☐☐ / ☐☐ **OR** ☐

B When did you first tell your GP or nurse? ☐☐ / ☐☐ / ☐☐ **OR** ☐

OR

C Put a cross here if you didn't tell your GP or nurse ☐

4. Bleeding from back passage

Did you have this symptom?

Yes ☐ **Please complete A then B or C below**

No ☐ **Please go to Section 2**

A When did you first notice this? ☐☐ / ☐☐ / ☐☐ **OR** ☐

B When did you first tell your GP or nurse? ☐☐ / ☐☐ / ☐☐ **OR** ☐

OR

C Put a cross here if you didn't tell your GP or nurse ☐

Section 2

Please give an exact date if you can. Otherwise give your best estimate (for example approximately how long ago, the month or the season). You may wish to refer to your diary or calendar if you have it with you.

Did you have any of the following?

1. Decrease in appetite

Did you have this?

Yes ☐ **Please complete A then B or C below**

No ☐ **Please go to Question 2**

A When did you first notice this? ☐☐ / ☐☐ / ☐☐ **OR** ☐

B When did you first tell your GP or nurse? ☐☐ / ☐☐ / ☐☐ **OR** ☐

OR

C Put a cross here if you didn't tell your GP or nurse ☐

2. Unexplained weight loss

Did you have this?

Yes ☐ **Please complete A then B or C below**

No ☐ **Please go to Question 3**

A When did you first notice this? ☐☐ / ☐☐ / ☐☐ **OR** ☐

B When did you first tell your GP or nurse? ☐☐ / ☐☐ / ☐☐ **OR** ☐

OR

C Put a cross here if you didn't tell your GP or nurse ☐

3. **Fatigue or tiredness that is unusual for you**

Did you have this?

Yes ☐ **Please complete A then B or C below**

No ☐ **Please go to Question 4**

A When did you first notice this? ☐☐ / ☐☐ / ☐☐ **OR** ☐

B When did you first tell your GP or nurse? ☐☐ / ☐☐ / ☐☐ **OR** ☐

OR

C Put a cross here if you didn't tell your GP or nurse ☐

4. **Feeling different 'in yourself' from usual**

Did you have this?

Yes ☐ **Please complete A then B or C below**

No ☐ **Please go to Question 5**

A When did you first notice this? ☐☐ / ☐☐ / ☐☐ **OR** ☐

B When did you first tell your GP or nurse? ☐☐ / ☐☐ / ☐☐ **OR** ☐

OR

C Put a cross here if you didn't tell your GP or nurse ☐

Please try and describe what the feeling was

If you had any further symptoms which you feel are relevant to your cancer diagnosis, please list them below. If not, please go to Question 7.

Please describe the symptom here and complete A then B or C below

5. ____________________

A When did you first notice this? ☐☐ / ☐☐ / ☐☐ **OR** ☐

B When did you first tell your GP or nurse? ☐☐ / ☐☐ / ☐☐ **OR** ☐

OR

C Put a cross here if you didn't tell your GP or nurse ☐

If you had another symptom you feel is relevant, please write it below, otherwise go to Question 7.

Please describe the symptom here and complete A then B or C below

6. ____________________

A When did you first notice this? ☐☐ / ☐☐ / ☐☐ **OR** ☐

B When did you first tell your GP or nurse? ☐☐ / ☐☐ / ☐☐ **OR** ☐

OR

C Put a cross here if you didn't tell your GP or nurse ☐

If you had further symptoms you would like to tell us about, there is space at the end of the Questionnaire for you to write them.

Did your GP send you for any tests?

Thinking back to the time immediately before you diagnosis, did your GP send you for any of the following tests?

Please cross the appropriate boxes

7. Were you sent for any of these?

	Yes	Not sure	No
Blood test(s)	☐	☐	☐
CT Scan	☐	☐	☐
Ultrasound scan	☐	☐	☐
Barium Enema	☐	☐	☐
Sigmoidoscopy or colonoscopy (Looking at bowel with internal camera)	☐	☐	☐
X-ray	☐	☐	☐

Please go to Question 8

About you

The next questions relate to general information about you. It would help us to know more about the background of the people completing the questionnaire.

8. Which best describes your employment status?

Please cross one box only

- Employed full-time ☐
- Employed part-time ☐
- Self employed full-time ☐
- Self employed part-time ☐
- Unemployed (seeking work) ☐
- Unemployed (not seeking work) ☐
- Retired ☐
- Student ☐
- Permanently sick/disabled ☐
- Temporarily sick/disabled ☐
- Looking after family/home ☐
- Other, please describe ____________

9. What is your highest level of qualification?

Please cross one box only

- Degree (or equivalent) ☐
- Diploma (or equivalent) ☐
- 'A' level ☐
- GCSE / 'O' level ☐
- None ☐
- Other, please specify ____________

10. How would you describe your ethnicity

Please cross one box only

White ☐
or
- White British ☐
- White Irish ☐
- Other White background ☐

Mixed ☐
or
- White & Black Carribean ☐
- White & Black African ☐
- White & Asian ☐
- Other mixed background ☐

Black or Black British ☐
or
- Carribean ☐
- African ☐
- Other Black background ☐

Asian or Asian British ☐
or
- Indian ☐
- Pakistani ☐
- Bangladeshi ☐
- Other Asian background ☐

Chinese or other ethnic group ☐
or
- Chinese ☐
- Other ethnic group ☐

11.

Do you live alone?

Yes ☐ **Please go to Question 12**

No ☐ **Please say who you live with below**

Who do you live with?
(e.g. wife, husband, partner, family member)

12. **Are you suffering from, or have you suffered from, any of the following in the past 2 years?**

Please cross any that are applicable to you

- Asthma ☐
- Chronic Obstructive Pulmonary Disease (COPD) ☐
- Other lung disease (e.g. fibrosis, bronchiectatis etc) ☐
- Heart disease ☐
- Anxiety or depression ☐
- Inflammatory bowel disease ☐
- Irritable bowel syndrome ☐
- Peptic ulcer ☐
- Previous cancer ☐
- Diabetes ☐
- Arthritis ☐

13. **About smoking**

Please cross the appropriate statement

- Are you a current smoker? ☐
- Are you an ex-smoker? ☐
- Are you a non-smoker (never smoked)? ☐

14. **Do you think you were more at risk of getting cancer because of your family history?**

Yes ☐

No ☐

15. **The following questions are about how completing the questionnaire made you feel.**

A number of statements which people have used to describe themselves are given below. Read each statement and then cross the most appropriate box to indicate how you feel right now, at the moment. There are no right or wrong answers. Do not spend too much time on any one statement but give the answer which seems to describe your present feeling best.

		Not at all	Somewhat	Moderately	Very much
1.	I feel calm	☐	☐	☐	☐
2.	I am tense	☐	☐	☐	☐
3.	I feel upset	☐	☐	☐	☐
4.	I am relaxed	☐	☐	☐	☐
5.	I feel content	☐	☐	☐	☐
6.	I am worried	☐	☐	☐	☐

Please make sure you have answered all the questions.

If you have any other comments or there is anything else you would like to tell us about when you first noticed your symptoms, please do so using the space below.

If you feel you would like to talk to someone about this questionnaire at a later stage, please speak to the doctor or nurse who is looking after you, or be in touch with the research team, whose contact details are on your patient information sheet

Thank you for completing the questionnaire

Please give it to the research nurse

The next page is for the Researcher to complete

Researcher - Post-completion Sheet

Researcher initials: ☐☐☐

Date questionnaire completed: Date ☐☐ / ☐☐ / ☐☐ Time ☐☐:☐☐

If not completed, give reason ☐

How long is it since the patient was told of his/her diagnosis? ☐

How long did it take the patient to complete the questionnaire?

- Less than 5 minutes ☐
- Between 5 and 10 minutes ☐
- Between 11 and 15 minutes ☐
- Longer than 15 minutes ☐

For the patient selected to <u>complete the questionnaire on their own</u>

Did the patient ask for any help? ☐ Yes ☐ No

If yes,

please specify what help was requested

___

___

please specify what help was given

___

___

For the patient selected for <u>the researcher-administered questionnaire</u>

Did the patient find any of the questions difficult? ☐ Yes ☐ No

If yes,

please specify which questions and what the difficulty was

___

___

___

___

Was the patient made anxious? ☐ Yes ☐ No

If yes,

please give details

___

___

___

___

Where did the patient complete the questionnaire?

(Please specify whether this was in a separate room alone, in a corner of the clinic, the ward or other

___

Thank you. Please now send the questionnaire and patient information sheet and consent form back to Cardiff University in the freepost envelope provided.

57727

PID - 78

Ymddiriedolaeth GIG Gogledd Orllewin Cymru
North West Wales NHS Trust

NHS WALES GIG CYMRU
Ymddiriedolaeth GIG Gogledd Cymru
North Wales NHS Trust

☐ GLAN CLWYD

CID - ☐ GWYNEDD

☐ MAELOR

Confidential Questionnaire

Looking At Your Symptoms Study

This Study has been funded by Cancer Research UK

CARDIFF UNIVERSITY PRIFYSGOL CAERDYDD

Ysgol Glinigol Gogledd Cymru North Wales Clinical School

CANCER RESEARCH UK

sewtu South East Wales Trials Unit Uned Ymchwil De-ddwyrain Cymru

Dear Patient

I am very grateful to you for agreeing to help us with this study.

I hope the information you supply will help us give people a diagnosis earlier.

If you feel you would like to talk to someone about this questionnaire at a later stage, please speak to the doctor or nurse who is looking after you, or be in touch with the research team, whose contact details are on your patient information sheet.

I can assure you that anything you tell us will be treated in the utmost confidence.

Yours sincerely

Dr Richard Neal
Senior Clinical Lecturer
Cardiff University
Chief Investigator, Symptoms Study

About your symptoms

These questions are about symptoms you noticed before your diagnosis. We are interested in symptoms which you think are related to your cancer.

Please put a cross in the appropriate box, or write an answer as required.

Looking back...

1. Did you have any symptoms that you feel were due to your cancer before you were diagnosed?

Yes ☐ **Please continue with Question 2**

No ☐ **Please go to Section 2 on Page 5**

2. What was the first symptom that made you think something might be wrong?

[ ]

Please go to Question 3

The following questions are about when you first noticed a symptom and when you first told your GP or nurse about it.

Please give an exact date if you can. Otherwise give your best estimate. (For example approximately how long ago, the month or season). You wish to refer to your diary or calendar if you have it with you.

Here is an example question:

Decrease in appetite

Did you have this symptom?

Yes ☒ **Please complete A then B or C below**

No ☐ **Please go to Section 2**

A When did you first notice this? d d / m m / y y **OR** Estimate '3 months ago' or 'June'

B When did you first tell your GP or nurse? 2 3 / 1 2 / 0 7 **OR** Estimate

OR

C Put a cross here if you didn't tell your GP or nurse ☐

3. Change in bowel habit

Did you have this symptom?

Yes ☐ **Please complete A then B or C below**

No ☐ **Please go to Question 4**

A When did you first notice this?

☐☐ / ☐☐ / ☐☐ **OR** ☐

B When did you first tell your GP or nurse?

☐☐ / ☐☐ / ☐☐ **OR** ☐

OR

C Put a cross here if you didn't tell your GP or nurse ☐

4. Bleeding from back passage

Did you have this symptom?

Yes ☐ **Please complete A then B or C below**

No ☐ **Please go to Section 2**

A When did you first notice this?

☐☐ / ☐☐ / ☐☐ **OR** ☐

B When did you first tell your GP or nurse?

☐☐ / ☐☐ / ☐☐ **OR** ☐

OR

C Put a cross here if you didn't tell your GP or nurse ☐

Section 2

Please give an exact date if you can. Otherwise give your best estimate (for example approximately how long ago, the month or the season). You may wish to refer to your diary or calendar if you have it with you.

Did you have any of the following?

1. Decrease in appetite

Did you have this?

Yes ☐ **Please complete A then B or C below**

No ☐ **Please go to Question 2**

A When did you first notice this? ☐☐ / ☐☐ / ☐☐ **OR** ☐

B When did you first tell your GP or nurse? ☐☐ / ☐☐ / ☐☐ **OR** ☐

OR

C Put a cross here if you didn't tell your GP or nurse ☐

2. Unexplained weight loss

Did you have this?

Yes ☐ **Please complete A then B or C below**

No ☐ **Please go to Question 3**

A When did you first notice this? ☐☐ / ☐☐ / ☐☐ **OR** ☐

B When did you first tell your GP or nurse? ☐☐ / ☐☐ / ☐☐ **OR** ☐

OR

C Put a cross here if you didn't tell your GP or nurse ☐

3. Fatigue or tiredness that is unusual for you

Did you have this?

Yes ☐ **Please complete A then B or C below**

No ☐ **Please go to Question 4**

A When did you first notice this? ☐☐ / ☐☐ / ☐☐ **OR** ☐

B When did you first tell your GP or nurse? ☐☐ / ☐☐ / ☐☐ **OR** ☐

OR

C Put a cross here if you didn't tell your GP or nurse ☐

4. Feeling different 'in yourself' from usual

Did you have this?

Yes ☐ **Please complete A then B or C below**

No ☐ **Please go to Question 5**

A When did you first notice this? ☐☐ / ☐☐ / ☐☐ **OR** ☐

B When did you first tell your GP or nurse? ☐☐ / ☐☐ / ☐☐ **OR** ☐

OR

C Put a cross here if you didn't tell your GP or nurse ☐

Please try and describe what the feeling was

If you had any further symptoms which you feel are relevant to your cancer diagnosis, please list them below. If not, please go to Question 7.

Please describe the symptom here and complete A then B or C below

5.

**A**	When did you first notice this?	☐☐ / ☐☐ / ☐☐ **OR** ☐
**B**	When did you first tell your GP or nurse?	☐☐ / ☐☐ / ☐☐ **OR** ☐
	**OR**	
**C**	Put a cross here if you didn't tell your GP or nurse	☐

If you had another symptom you feel is relevant, please write it below, otherwise go to Question 7.

Please describe the symptom here and complete A then B or C below

6.

**A**	When did you first notice this?	☐☐ / ☐☐ / ☐☐ **OR** ☐
**B**	When did you first tell your GP or nurse?	☐☐ / ☐☐ / ☐☐ **OR** ☐
	**OR**	
**C**	Put a cross here if you didn't tell your GP or nurse	☐

If you had further symptoms you would like to tell us about, there is space at the end of the Questionnaire for you to write them.

Did your GP send you for any tests?

Thinking back to the time immediately before you diagnosis, did your GP send you for any of the following tests?

Please cross the appropriate boxes

7. Were you sent for any of these?

	Yes	Not sure	No
Blood test(s)	☐	☐	☐
CT Scan	☐	☐	☐
Ultrasound scan	☐	☐	☐
Barium Enema	☐	☐	☐
Sigmoidoscopy or colonoscopy (Looking at bowel with internal camera)	☐	☐	☐
X-ray	☐	☐	☐

Please go to Question 8

About you

The next questions relate to general information about you. It would help us to know more about the background of the people completing the questionnaire.

8. **Which best describes your employment status?**

Please cross one box only

- Employed full-time ☐
- Employed part-time ☐
- Self employed full-time ☐
- Self employed part-time ☐
- Unemployed (seeking work) ☐
- Unemployed (not seeking work) ☐
- Retired ☐
- Student ☐
- Permanently sick/disabled ☐
- Temporarily sick/disabled ☐
- Looking after family/home ☐
- Other, please describe [ ]

9. **What is your highest level of qualification?**

Please cross one box only

- Degree (or equivalent) ☐
- Diploma (or equivalent) ☐
- 'A' level ☐
- GCSE / 'O' level ☐
- None ☐
- Other, please specify [ ]

10. **How would you describe your ethnicity**

Please cross one box only

White ☐
or
- White British ☐
- White Irish ☐
- Other White background ☐

Mixed ☐
or
- White & Black Carribean ☐
- White & Black African ☐
- White & Asian ☐
- Other mixed background ☐

Black or Black British ☐
or
- Carribean ☐
- African ☐
- Other Black background ☐

Asian or Asian British ☐
or
- Indian ☐
- Pakistani ☐
- Bangladeshi ☐
- Other Asian background ☐

Chinese or other ethnic group ☐
or
- Chinese ☐
- Other ethnic group ☐

11.

Do you live alone?

Yes ☐ **Please go to Question 12**

No ☐ **Please say who you live with below**

Who do you live with?
(e.g. wife, husband, partner, family member)

12. Are you suffering from, or have you suffered from, any of the following in the past 2 years?

Please cross any that are applicable to you

- Asthma ☐
- Chronic Obstructive Pulmonary Disease (COPD) ☐
- Other lung disease (e.g. fibrosis, bronchiectatis etc) ☐
- Heart disease ☐
- Anxiety or depression ☐
- Inflammatory bowel disease ☐
- Irritable bowel syndrome ☐
- Peptic ulcer ☐
- Previous cancer ☐
- Diabetes ☐
- Arthritis ☐

13. About smoking

Please cross the appropriate statement

- Are you a current smoker? ☐
- Are you an ex-smoker? ☐
- Are you a non-smoker (never smoked)? ☐

14. Do you think you were more at risk of getting cancer because of your family history?

Yes ☐

No ☐

15. **The following questions are about how completing the questionnaire made you feel.**

A number of statements which people have used to describe themselves are given below. Read each statement and then cross the most appropriate box to indicate how you feel right now, at the moment. There are no right or wrong answers. Do not spend too much time on any one statement but give the answer which seems to describe your present feeling best.

		Not at all	Somewhat	Moderately	Very much
1.	I feel calm	☐	☐	☐	☐
2.	I am tense	☐	☐	☐	☐
3.	I feel upset	☐	☐	☐	☐
4.	I am relaxed	☐	☐	☐	☐
5.	I feel content	☐	☐	☐	☐
6.	I am worried	☐	☐	☐	☐

Please make sure you have answered all the questions.

If you have any other comments or there is anything else you would like to tell us about when you first noticed your symptoms, please do so using the space below.

If you feel you would like to talk to someone about this questionnaire at a later stage, please speak to the doctor or nurse who is looking after you, or be in touch with the research team, whose contact details are on your patient information sheet

Thank you for completing the questionnaire

Please give it to the research nurse

The next page is for the Researcher to complete

Researcher - Post-completion Sheet

Researcher initials: ☐☐☐

Date questionnaire completed: Date ☐☐ / ☐☐ / ☐☐ Time ☐☐:☐☐

If not completed, give reason ☐

How long is it since the patient was told of his/her diagnosis? ☐

How long did it take the patient to complete the questionnaire?

- Less than 5 minutes ☐
- Between 5 and 10 minutes ☐
- Between 11 and 15 minutes ☐
- Longer than 15 minutes ☐

For the patient selected to <u>complete the questionnaire on their own</u>

Did the patient ask for any help? ☐ Yes ☐ No

If yes,

please specify what help was requested

____

____

please specify what help was given

____

____

For the patient selected for <u>the researcher-administered questionnaire</u>

Did the patient find any of the questions difficult? ☐ Yes ☐ No

If yes,

please specify which questions and what the difficulty was

____

____

____

____

Was the patient made anxious? ☐ Yes ☐ No

If yes,

please give details

____

____

____

____

Where did the patient complete the questionnaire?
(Please specify whether this was in a separate room alone, in a corner of the clinic, the ward or other

____

Thank you. Please now send the questionnaire and patient information sheet and consent form back to Cardiff University in the freepost envelope provided.

57727

PID - | 7 | 9

☐ GLAN CLWYD

CID - ☐ GWYNEDD

☐ MAELOR

Confidential Questionnaire

Looking At Your Symptoms Study

This Study has been funded by Cancer Research UK

Dear Patient

I am very grateful to you for agreeing to help us with this study.

I hope the information you supply will help us give people a diagnosis earlier.

If you feel you would like to talk to someone about this questionnaire at a later stage, please speak to the doctor or nurse who is looking after you, or be in touch with the research team, whose contact details are on your patient information sheet.

I can assure you that anything you tell us will be treated in the utmost confidence.

Yours sincerely

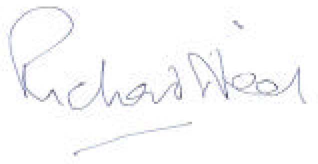

Dr Richard Neal
Senior Clinical Lecturer
Cardiff University
Chief Investigator, Symptoms Study

About your symptoms

These questions are about symptoms you noticed before your diagnosis. We are interested in symptoms which you think are related to your cancer.

Please put a cross in the appropriate box, or write an answer as required.

Looking back...

1. Did you have any symptoms that you feel were due to your cancer before you were diagnosed?

Yes ☐ **Please continue with Question 2**

No ☐ **Please go to Section 2 on Page 5**

2. What was the first symptom that made you think something might be wrong?

☐

Please go to Question 3

The following questions are about when you first noticed a symptom and when you first told your GP or nurse about it.

Please give an exact date if you can. Otherwise give your best estimate. (For example approximately how long ago, the month or season). You wish to refer to your diary or calendar if you have it with you.

Here is an example question:

Decrease in appetite

Did you have this symptom?

Yes ☒ **Please complete A then B or C below**

No ☐ **Please go to Section 2**

A When did you first notice this? d d / m m / y y **OR** Estimate '3 months ago' or 'June'

B When did you first tell your GP or nurse? 2 3 / 1 2 / 0 7 **OR** Estimate

OR

C Put a cross here if you didn't tell your GP or nurse ☐

3. Change in bowel habit

Did you have this symptom?

Yes ☐ **Please complete A then B or C below**

No ☐ **Please go to Question 4**

A When did you first notice this? ☐☐ / ☐☐ / ☐☐ **OR** ☐

B When did you first tell your GP or nurse? ☐☐ / ☐☐ / ☐☐ **OR** ☐

OR

C Put a cross here if you didn't tell your GP or nurse ☐

4. Bleeding from back passage

Did you have this symptom?

Yes ☐ **Please complete A then B or C below**

No ☐ **Please go to Section 2**

A When did you first notice this? ☐☐ / ☐☐ / ☐☐ **OR** ☐

B When did you first tell your GP or nurse? ☐☐ / ☐☐ / ☐☐ **OR** ☐

OR

C Put a cross here if you didn't tell your GP or nurse ☐

Section 2

Please give an exact date if you can. Otherwise give your best estimate (for example approximately how long ago, the month or the season). You may wish to refer to your diary or calendar if you have it with you.

Did you have any of the following?

1. Decrease in appetite

Did you have this?

Yes ☐ **Please complete A then B or C below**

No ☐ **Please go to Question 2**

A When did you first notice this? ☐☐ / ☐☐ / ☐☐ **OR** ☐

B When did you first tell your GP or nurse? ☐☐ / ☐☐ / ☐☐ **OR** ☐

OR

C Put a cross here if you didn't tell your GP or nurse ☐

2. Unexplained weight loss

Did you have this?

Yes ☐ **Please complete A then B or C below**

No ☐ **Please go to Question 3**

A When did you first notice this? ☐☐ / ☐☐ / ☐☐ **OR** ☐

B When did you first tell your GP or nurse? ☐☐ / ☐☐ / ☐☐ **OR** ☐

OR

C Put a cross here if you didn't tell your GP or nurse ☐

3. Fatigue or tiredness that is unusual for you

Did you have this?

Yes ☐ **Please complete A then B or C below**

No ☐ **Please go to Question 4**

A When did you first notice this? ☐☐ / ☐☐ / ☐☐ **OR** ☐

B When did you first tell your GP or nurse? ☐☐ / ☐☐ / ☐☐ **OR** ☐

OR

C Put a cross here if you didn't tell your GP or nurse ☐

4. Feeling different 'in yourself' from usual

Did you have this?

Yes ☐ **Please complete A then B or C below**

No ☐ **Please go to Question 5**

A When did you first notice this? ☐☐ / ☐☐ / ☐☐ **OR** ☐

B When did you first tell your GP or nurse? ☐☐ / ☐☐ / ☐☐ **OR** ☐

OR

C Put a cross here if you didn't tell your GP or nurse ☐

Please try and describe what the feeling was

If you had any further symptoms which you feel are relevant to your cancer diagnosis, please list them below. If not, please go to Question 7.

Please describe the symptom here and complete A then B or C below

5.

**A** When did you first notice this?	__ __ / __ __ / __ __	**OR**
**B** When did you first tell your GP or nurse?	__ __ / __ __ / __ __	**OR**
**OR** **C** Put a cross here if you didn't tell your GP or nurse	☐	

If you had another symptom you feel is relevant, please write it below, otherwise go to Question 7.

Please describe the symptom here and complete A then B or C below

6.

**A** When did you first notice this?	__ __ / __ __ / __ __	**OR**
**B** When did you first tell your GP or nurse?	__ __ / __ __ / __ __	**OR**
**OR** **C** Put a cross here if you didn't tell your GP or nurse	☐	

If you had further symptoms you would like to tell us about, there is space at the end of the Questionnaire for you to write them.

Did your GP send you for any tests?

Thinking back to the time immediately before you diagnosis, did your GP send you for any of the following tests?

Please cross the appropriate boxes

7. Were you sent for any of these?

	Yes	Not sure	No
Blood test(s)	☐	☐	☐
CT Scan	☐	☐	☐
Ultrasound scan	☐	☐	☐
Barium Enema	☐	☐	☐
Sigmoidoscopy or colonoscopy (Looking at bowel with internal camera)	☐	☐	☐
X-ray	☐	☐	☐

Please go to Question 8

About you

The next questions relate to general information about you. It would help us to know more about the background of the people completing the questionnaire.

8. Which best describes your employment status?

Please cross one box only

- Employed full-time ☐
- Employed part-time ☐
- Self employed full-time ☐
- Self employed part-time ☐
- Unemployed (seeking work) ☐
- Unemployed (not seeking work) ☐
- Retired ☐
- Student ☐
- Permanently sick/disabled ☐
- Temporarily sick/disabled ☐
- Looking after family/home ☐
- Other, please describe ______

9. What is your highest level of qualification?

Please cross one box only

- Degree (or equivalent) ☐
- Diploma (or equivalent) ☐
- 'A' level ☐
- GCSE / 'O' level ☐
- None ☐
- Other, please specify ______

10. **How would you describe your ethnicity**

Please cross one box only

White ☐
or
White British ☐
White Irish ☐
Other White background ☐

Mixed ☐
or
White & Black Carribean ☐
White & Black African ☐
White & Asian ☐
Other mixed background ☐

Black or Black British ☐
or
Carribean ☐
African ☐
Other Black background ☐

Asian or Asian British ☐
or
Indian ☐
Pakistani ☐
Bangladeshi ☐
Other Asian background ☐

Chinese or other ethnic group ☐
or
Chinese ☐
Other ethnic group ☐

11.

Do you live alone?

Yes ☐ **Please go to Question 12**

No ☐ **Please say who you live with below**

Who do you live with?
(e.g. wife, husband, partner, family member)

12. **Are you suffering from, or have you suffered from, any of the following in the past 2 years?**

Please cross any that are applicable to you

- Asthma ☐
- Chronic Obstructive Pulmonary Disease (COPD) ☐
- Other lung disease (e.g. fibrosis, bronchiectatis etc) ☐
- Heart disease ☐
- Anxiety or depression ☐
- Inflammatory bowel disease ☐
- Irritable bowel syndrome ☐
- Peptic ulcer ☐
- Previous cancer ☐
- Diabetes ☐
- Arthritis ☐

13. **About smoking**

Please cross the appropriate statement

- Are you a current smoker? ☐
- Are you an ex-smoker? ☐
- Are you a non-smoker (never smoked)? ☐

14. **Do you think you were more at risk of getting cancer because of your family history?**

Yes ☐
No ☐

15. **The following questions are about how completing the questionnaire made you feel.**

A number of statements which people have used to describe themselves are given below. Read each statement and then cross the most appropriate box to indicate how you feel right now, at the moment. There are no right or wrong answers. Do not spend too much time on any one statement but give the answer which seems to describe your present feeling best.

		Not at all	Somewhat	Moderately	Very much
1.	I feel calm	☐	☐	☐	☐
2.	I am tense	☐	☐	☐	☐
3.	I feel upset	☐	☐	☐	☐
4.	I am relaxed	☐	☐	☐	☐
5.	I feel content	☐	☐	☐	☐
6.	I am worried	☐	☐	☐	☐

Please make sure you have answered all the questions.

If you have any other comments or there is anything else you would like to tell us about when you first noticed your symptoms, please do so using the space below.

If you feel you would like to talk to someone about this questionnaire at a later stage, please speak to the doctor or nurse who is looking after you, or be in touch with the research team, whose contact details are on your patient information sheet

Thank you for completing the questionnaire

Please give it to the research nurse

The next page is for the Researcher to complete

57727

7 9

Researcher - Post-completion Sheet

Researcher initials: ☐☐☐

Date questionnaire completed: Date ☐☐ / ☐☐ / ☐☐ Time ☐☐:☐☐

If not completed, give reason ☐

How long is it since the patient was told of his/her diagnosis? ☐

How long did it take the patient to complete the questionnaire?

- Less than 5 minutes ☐
- Between 5 and 10 minutes ☐
- Between 11 and 15 minutes ☐
- Longer than 15 minutes ☐

For the patient selected to complete the questionnaire on their own

Did the patient ask for any help? ☐ Yes ☐ No

If yes,

please specify what help was requested

____

____

please specify what help was given

____

____

For the patient selected for the researcher-administered questionnaire

Did the patient find any of the questions difficult? ☐ Yes ☐ No

If yes,

please specify which questions and what the difficulty was

____

____

____

____

Was the patient made anxious? ☐ Yes ☐ No

If yes,

please give details

____

____

____

____

Where did the patient complete the questionnaire?
(Please specify whether this was in a separate room alone, in a corner of the clinic, the ward or other

____

Thank you. Please now send the questionnaire and patient information sheet and consent form back to Cardiff University in the freepost envelope provided.

57727

PID - 8 0

Ymddiriedolaeth GIG Gogledd Orllewin Cymru
North West Wales NHS Trust

NHS Wales GIG Cymru
Ymddiriedolaeth GIG Gogledd Cymru
North Wales NHS Trust

☐ GLAN CLWYD

CID - ☐ GWYNEDD

☐ MAELOR

Confidential Questionnaire

Looking At Your Symptoms Study

This Study has been funded by Cancer Research UK

Cardiff University / Prifysgol Caerdydd

Ysgol Glinigol Gogledd Cymru / North Wales Clinical School

Cancer Research UK

sewtu — South East Wales Trials Unit / Uned Ymchwil De-ddwyrain Cymru

Dear Patient

I am very grateful to you for agreeing to help us with this study.

I hope the information you supply will help us give people a diagnosis earlier.

If you feel you would like to talk to someone about this questionnaire at a later stage, please speak to the doctor or nurse who is looking after you, or be in touch with the research team, whose contact details are on your patient information sheet.

I can assure you that anything you tell us will be treated in the utmost confidence.

Yours sincerely

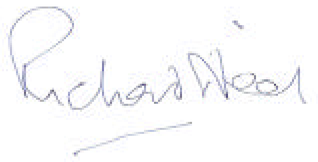

Dr Richard Neal
Senior Clinical Lecturer
Cardiff University
Chief Investigator, Symptoms Study

About your symptoms

These questions are about symptoms you noticed before your diagnosis. We are interested in symptoms which you think are related to your cancer.

Please put a cross in the appropriate box, or write an answer as required.

Looking back...

1. Did you have any symptoms that you feel were due to your cancer before you were diagnosed?

Yes ☐ **Please continue with Question 2**

No ☐ **Please go to Section 2 on Page 5**

2. What was the first symptom that made you think something might be wrong?

☐

Please go to Question 3

The following questions are about when you first noticed a symptom and when you first told your GP or nurse about it.

Please give an exact date if you can. Otherwise give your best estimate. (For example approximately how long ago, the month or season). You wish to refer to your diary or calendar if you have it with you.

Here is an example question:

Decrease in appetite

Did you have this symptom?

Yes ☒ **Please complete A then B or C below**

No ☐ **Please go to Section 2**

A When did you first notice this? d d / m m / y y **OR** Estimate '3 months ago' or 'June'

B When did you first tell your GP or nurse? 2 3 / 1 2 / 0 7 **OR** Estimate

OR

C Put a cross here if you didn't tell your GP or nurse ☐

3. Change in bowel habit

Did you have this symptom?

Yes ☐ **Please complete A then B or C below**

No ☐ **Please go to Question 4**

A When did you first notice this? ☐☐ / ☐☐ / ☐☐ **OR** ☐

B When did you first tell your GP or nurse? ☐☐ / ☐☐ / ☐☐ **OR** ☐

OR

C Put a cross here if you didn't tell your GP or nurse ☐

4. Bleeding from back passage

Did you have this symptom?

Yes ☐ **Please complete A then B or C below**

No ☐ **Please go to Section 2**

A When did you first notice this? ☐☐ / ☐☐ / ☐☐ **OR** ☐

B When did you first tell your GP or nurse? ☐☐ / ☐☐ / ☐☐ **OR** ☐

OR

C Put a cross here if you didn't tell your GP or nurse ☐

Section 2

Please give an exact date if you can. Otherwise give your best estimate (for example approximately how long ago, the month or the season). You may wish to refer to your diary or calendar if you have it with you.

Did you have any of the following?

1. Decrease in appetite

Did you have this?

Yes ☐ **Please complete A then B or C below**

No ☐ **Please go to Question 2**

A When did you first notice this? ☐☐ / ☐☐ / ☐☐ **OR** ☐

B When did you first tell your GP or nurse? ☐☐ / ☐☐ / ☐☐ **OR** ☐

OR

C Put a cross here if you didn't tell your GP or nurse ☐

2. Unexplained weight loss

Did you have this?

Yes ☐ **Please complete A then B or C below**

No ☐ **Please go to Question 3**

A When did you first notice this? ☐☐ / ☐☐ / ☐☐ **OR** ☐

B When did you first tell your GP or nurse? ☐☐ / ☐☐ / ☐☐ **OR** ☐

OR

C Put a cross here if you didn't tell your GP or nurse ☐

3. Fatigue or tiredness that is unusual for you

Did you have this?

Yes ☐ **Please complete A then B or C below**

No ☐ **Please go to Question 4**

A When did you <u>first notice</u> this? ☐☐ / ☐☐ / ☐☐ **OR** ☐

B When did you <u>first tell</u> your GP or nurse? ☐☐ / ☐☐ / ☐☐ **OR** ☐

OR

C Put a cross here if you <u>didn't tell</u> your GP or nurse ☐

4. Feeling different 'in yourself' from usual

Did you have this?

Yes ☐ **Please complete A then B or C below**

No ☐ **Please go to Question 5**

A When did you <u>first notice</u> this? ☐☐ / ☐☐ / ☐☐ **OR** ☐

B When did you <u>first tell</u> your GP or nurse? ☐☐ / ☐☐ / ☐☐ **OR** ☐

OR

C Put a cross here if you <u>didn't tell</u> your GP or nurse ☐

Please try and describe what the feeling was

If you had any further symptoms which you feel are relevant to your cancer diagnosis, please list them below. If not, please go to Question 7.

Please describe the symptom here and complete A then B or C below

5.

**A**	When did you first notice this?	☐☐ / ☐☐ / ☐☐ **OR** ☐
**B**	When did you first tell your GP or nurse?	☐☐ / ☐☐ / ☐☐ **OR** ☐
	**OR**	
**C**	Put a cross here if you didn't tell your GP or nurse	☐

If you had another symptom you feel is relevant, please write it below, otherwise go to Question 7.

Please describe the symptom here and complete A then B or C below

6.

**A**	When did you first notice this?	☐☐ / ☐☐ / ☐☐ **OR** ☐
**B**	When did you first tell your GP or nurse?	☐☐ / ☐☐ / ☐☐ **OR** ☐
	**OR**	
**C**	Put a cross here if you didn't tell your GP or nurse	☐

If you had further symptoms you would like to tell us about, there is space at the end of the Questionnaire for you to write them.

Did your GP send you for any tests?

Thinking back to the time immediately before you diagnosis, did your GP send you for any of the following tests?

Please cross the appropriate boxes

7. Were you sent for any of these?

	Yes	Not sure	No
Blood test(s)	☐	☐	☐
CT Scan	☐	☐	☐
Ultrasound scan	☐	☐	☐
Barium Enema	☐	☐	☐
Sigmoidoscopy or colonoscopy (Looking at bowel with internal camera)	☐	☐	☐
X-ray	☐	☐	☐

Please go to Question 8

About you

The next questions relate to general information about you. It would help us to know more about the background of the people completing the questionnaire.

8. Which best describes your employment status?

Please cross one box only

- Employed full-time ☐
- Employed part-time ☐
- Self employed full-time ☐
- Self employed part-time ☐
- Unemployed (seeking work) ☐
- Unemployed (not seeking work) ☐
- Retired ☐
- Student ☐
- Permanently sick/disabled ☐
- Temporarily sick/disabled ☐
- Looking after family/home ☐
- Other, please describe ____________

9. What is your highest level of qualification?

Please cross one box only

- Degree (or equivalent) ☐
- Diploma (or equivalent) ☐
- 'A' level ☐
- GCSE / 'O' level ☐
- None ☐
- Other, please specify ____________

10. How would you describe your ethnicity

Please cross one box only

White ☐
or
- White British ☐
- White Irish ☐
- Other White background ☐

Mixed ☐
or
- White & Black Carribean ☐
- White & Black African ☐
- White & Asian ☐
- Other mixed background ☐

Black or Black British ☐
or
- Carribean ☐
- African ☐
- Other Black background ☐

Asian or Asian British ☐
or
- Indian ☐
- Pakistani ☐
- Bangladeshi ☐
- Other Asian background ☐

Chinese or other ethnic group ☐
or
- Chinese ☐
- Other ethnic group ☐

11.

Do you live alone?

Yes ☐ **Please go to Question 12**

No ☐ **Please say who you live with below**

Who do you live with?
(e.g. wife, husband, partner, family member)

12. **Are you suffering from, or have you suffered from, any of the following in the past 2 years?**

Please cross any that are applicable to you

- Asthma ☐
- Chronic Obstructive Pulmonary Disease (COPD) ☐
- Other lung disease (e.g. fibrosis, bronchiectatis etc) ☐
- Heart disease ☐
- Anxiety or depression ☐
- Inflammatory bowel disease ☐
- Irritable bowel syndrome ☐
- Peptic ulcer ☐
- Previous cancer ☐
- Diabetes ☐
- Arthritis ☐

13. **About smoking**

Please cross the appropriate statement

- Are you a current smoker? ☐
- Are you an ex-smoker? ☐
- Are you a non-smoker (never smoked)? ☐

14. **Do you think you were more at risk of getting cancer because of your family history?**

Yes ☐
No ☐

15. The following questions are about how completing the questionnaire made you feel.

A number of statements which people have used to describe themselves are given below. Read each statement and then cross the most appropriate box to indicate how you feel right now, at the moment. There are no right or wrong answers. Do not spend too much time on any one statement but give the answer which seems to describe your present feeling best.

		Not at all	Somewhat	Moderately	Very much
1.	I feel calm	☐	☐	☐	☐
2.	I am tense	☐	☐	☐	☐
3.	I feel upset	☐	☐	☐	☐
4.	I am relaxed	☐	☐	☐	☐
5.	I feel content	☐	☐	☐	☐
6.	I am worried	☐	☐	☐	☐

Please make sure you have answered all the questions.

If you have any other comments or there is anything else you would like to tell us about when you first noticed your symptoms, please do so using the space below.

If you feel you would like to talk to someone about this questionnaire at a later stage, please speak to the doctor or nurse who is looking after you, or be in touch with the research team, whose contact details are on your patient information sheet

Thank you for completing the questionnaire

Please give it to the research nurse

The next page is for the Researcher to complete

57727

80

Researcher - Post-completion Sheet

Researcher initials: ☐☐☐

Date questionnaire completed: Date ☐☐ / ☐☐ / ☐☐ Time ☐☐:☐☐

If not completed, give reason ☐

How long is it since the patient was told of his/her diagnosis? ☐

How long did it take the patient to complete the questionnaire?

- Less than 5 minutes ☐
- Between 5 and 10 minutes ☐
- Between 11 and 15 minutes ☐
- Longer than 15 minutes ☐

For the patient selected to complete the questionnaire on their own

Did the patient ask for any help? ☐ Yes ☐ No

If yes,

please specify what help was requested

____

____

please specify what help was given

____

____

For the patient selected for the researcher-administered questionnaire

Did the patient find any of the questions difficult? ☐ Yes ☐ No

If yes,

please specify which questions and what the difficulty was

____

____

____

____

Was the patient made anxious? ☐ Yes ☐ No

If yes,

please give details

____

____

____

____

Where did the patient complete the questionnaire?

(Please specify whether this was in a separate room alone, in a corner of the clinic, the ward or other

____

Thank you. Please now send the questionnaire and patient information sheet and consent form back to Cardiff University in the freepost envelope provided.

57727

PID - 81

Ymddiriedolaeth GIG Gogledd Orllewin Cymru
North West Wales NHS Trust

NHS WALES GIG CYMRU
Ymddiriedolaeth GIG Gogledd Cymru
North Wales NHS Trust

☐ GLAN CLWYD

CID - ☐ GWYNEDD

☐ MAELOR

Confidential Questionnaire

Looking At Your Symptoms Study

This Study has been funded by Cancer Research UK

Cardiff University / Prifysgol Caerdydd

Ysgol Glinigol Gogledd Cymru
North Wales Clinical School

Cancer Research UK

sewtu
South East Wales Trials Unit
Uned Ymchwil De-ddwyrain Cymru

Dear Patient

I am very grateful to you for agreeing to help us with this study.

I hope the information you supply will help us give people a diagnosis earlier.

If you feel you would like to talk to someone about this questionnaire at a later stage, please speak to the doctor or nurse who is looking after you, or be in touch with the research team, whose contact details are on your patient information sheet.

I can assure you that anything you tell us will be treated in the utmost confidence.

Yours sincerely

Dr Richard Neal
Senior Clinical Lecturer
Cardiff University
Chief Investigator, Symptoms Study

About your symptoms

These questions are about symptoms you noticed before your diagnosis. We are interested in symptoms which you think are related to your cancer.

Please put a cross in the appropriate box, or write an answer as required.

Looking back...

1. Did you have any symptoms that you feel were due to your cancer before you were diagnosed?

Yes ☐ **Please continue with Question 2**

No ☐ **Please go to Section 2 on Page 5**

2. What was the first symptom that made you think something might be wrong?

☐

Please go to Question 3

The following questions are about when you first noticed a symptom and when you first told your GP or nurse about it.

Please give an exact date if you can. Otherwise give your best estimate. (For example approximately how long ago, the month or season). You wish to refer to your diary or calendar if you have it with you.

Here is an example question:

Decrease in appetite

Did you have this symptom?

Yes ☒ **Please complete A then B or C below**

No ☐ **Please go to Section 2**

A When did you first notice this? d d / m m / y y **OR** Estimate '3 months ago' or 'June'

B When did you first tell your GP or nurse? 2 3 / 1 2 / 0 7 **OR** Estimate

OR

C Put a cross here if you didn't tell your GP or nurse ☐

3. Change in bowel habit

Did you have this symptom?

Yes ☐ **Please complete A then B or C below**

No ☐ **Please go to Question 4**

A When did you <u>first notice</u> this? ☐☐ / ☐☐ / ☐☐ **OR** ☐

B When did you <u>first tell</u> your GP or nurse? ☐☐ / ☐☐ / ☐☐ **OR** ☐

OR

C Put a cross here if you <u>didn't tell</u> your GP or nurse ☐

4. Bleeding from back passage

Did you have this symptom?

Yes ☐ **Please complete A then B or C below**

No ☐ **Please go to Section 2**

A When did you <u>first notice</u> this? ☐☐ / ☐☐ / ☐☐ **OR** ☐

B When did you <u>first tell</u> your GP or nurse? ☐☐ / ☐☐ / ☐☐ **OR** ☐

OR

C Put a cross here if you <u>didn't tell</u> your GP or nurse ☐

Section 2

Please give an exact date if you can. Otherwise give your best estimate (for example approximately how long ago, the month or the season). You may wish to refer to your diary or calendar if you have it with you.

Did you have any of the following?

1. Decrease in appetite

Did you have this?

Yes ☐ **Please complete A then B or C below**

No ☐ **Please go to Question 2**

A When did you first notice this? ☐☐ / ☐☐ / ☐☐ **OR** ☐

B When did you first tell your GP or nurse? ☐☐ / ☐☐ / ☐☐ **OR** ☐

OR

C Put a cross here if you didn't tell your GP or nurse ☐

2. Unexplained weight loss

Did you have this?

Yes ☐ **Please complete A then B or C below**

No ☐ **Please go to Question 3**

A When did you first notice this? ☐☐ / ☐☐ / ☐☐ **OR** ☐

B When did you first tell your GP or nurse? ☐☐ / ☐☐ / ☐☐ **OR** ☐

OR

C Put a cross here if you didn't tell your GP or nurse ☐

3. Fatigue or tiredness that is unusual for you

Did you have this?

Yes ☐ **Please complete A then B or C below**

No ☐ **Please go to Question 4**

A When did you first notice this? ☐☐ / ☐☐ / ☐☐ **OR** ☐

B When did you first tell your GP or nurse? ☐☐ / ☐☐ / ☐☐ **OR** ☐

OR

C Put a cross here if you didn't tell your GP or nurse ☐

4. Feeling different 'in yourself' from usual

Did you have this?

Yes ☐ **Please complete A then B or C below**

No ☐ **Please go to Question 5**

A When did you first notice this? ☐☐ / ☐☐ / ☐☐ **OR** ☐

B When did you first tell your GP or nurse? ☐☐ / ☐☐ / ☐☐ **OR** ☐

OR

C Put a cross here if you didn't tell your GP or nurse ☐

Please try and describe what the feeling was

If you had any further symptoms which you feel are relevant to your cancer diagnosis, please list them below. If not, please go to Question 7.

Please describe the symptom here and complete A then B or C below

5.

**A** When did you <u>first notice</u> this?	☐☐ / ☐☐ / ☐☐ **OR**
**B** When did you <u>first tell</u> your GP or nurse?	☐☐ / ☐☐ / ☐☐ **OR**
**OR**	
**C** Put a cross here if you <u>didn't tell</u> your GP or nurse	☐

If you had another symptom you feel is relevant, please write it below, otherwise go to Question 7.

Please describe the symptom here and complete A then B or C below

6.

**A** When did you <u>first notice</u> this?	☐☐ / ☐☐ / ☐☐ **OR**
**B** When did you <u>first tell</u> your GP or nurse?	☐☐ / ☐☐ / ☐☐ **OR**
**OR**	
**C** Put a cross here if you <u>didn't tell</u> your GP or nurse	☐

If you had further symptoms you would like to tell us about, there is space at the end of the Questionnaire for you to write them.

Did your GP send you for any tests?

Thinking back to the time immediately before you diagnosis, did your GP send you for any of the following tests?

Please cross the appropriate boxes

7. Were you sent for any of these?

	Yes	Not sure	No
Blood test(s)	☐	☐	☐
CT Scan	☐	☐	☐
Ultrasound scan	☐	☐	☐
Barium Enema	☐	☐	☐
Sigmoidoscopy or colonoscopy (Looking at bowel with internal camera)	☐	☐	☐
X-ray	☐	☐	☐

Please go to Question 8

About you

The next questions relate to general information about you. It would help us to know more about the background of the people completing the questionnaire.

8. **Which best describes your employment status?**

Please cross one box only

- Employed full-time ☐
- Employed part-time ☐
- Self employed full-time ☐
- Self employed part-time ☐
- Unemployed (seeking work) ☐
- Unemployed (not seeking work) ☐
- Retired ☐
- Student ☐
- Permanently sick/disabled ☐
- Temporarily sick/disabled ☐
- Looking after family/home ☐
- Other, please describe ______

9. **What is your highest level of qualification?**

Please cross one box only

- Degree (or equivalent) ☐
- Diploma (or equivalent) ☐
- 'A' level ☐
- GCSE / 'O' level ☐
- None ☐
- Other, please specify ______

10. **How would you describe your ethnicity**

Please cross one box only

White ☐
or
White British ☐
White Irish ☐
Other White background ☐

Mixed ☐
or
White & Black Carribean ☐
White & Black African ☐
White & Asian ☐
Other mixed background ☐

Black or Black British ☐
or
Carribean ☐
African ☐
Other Black background ☐

Asian or Asian British ☐
or
Indian ☐
Pakistani ☐
Bangladeshi ☐
Other Asian background ☐

Chinese or other ethnic group ☐
or
Chinese ☐
Other ethnic group ☐

11.

Do you live alone?

Yes ☐ **Please go to Question 12**

No ☐ **Please say who you live with below**

Who do you live with?
(e.g. wife, husband, partner, family member)

12. Are you suffering from, or have you suffered from, any of the following in the past 2 years?

Please cross any that are applicable to you

- Asthma ☐
- Chronic Obstructive Pulmonary Disease (COPD) ☐
- Other lung disease (e.g. fibrosis, bronchiectatis etc) ☐
- Heart disease ☐
- Anxiety or depression ☐
- Inflammatory bowel disease ☐
- Irritable bowel syndrome ☐
- Peptic ulcer ☐
- Previous cancer ☐
- Diabetes ☐
- Arthritis ☐

13. About smoking

Please cross the appropriate statement

- Are you a current smoker? ☐
- Are you an ex-smoker? ☐
- Are you a non-smoker (never smoked)? ☐

14. Do you think you were more at risk of getting cancer because of your family history?

Yes ☐

No ☐

15. **The following questions are about how completing the questionnaire made you feel.**

A number of statements which people have used to describe themselves are given below. Read each statement and then cross the most appropriate box to indicate how you feel right now, at the moment. There are no right or wrong answers. Do not spend too much time on any one statement but give the answer which seems to describe your present feeling best.

		Not at all	Somewhat	Moderately	Very much
1.	I feel calm	☐	☐	☐	☐
2.	I am tense	☐	☐	☐	☐
3.	I feel upset	☐	☐	☐	☐
4.	I am relaxed	☐	☐	☐	☐
5.	I feel content	☐	☐	☐	☐
6.	I am worried	☐	☐	☐	☐

Please make sure you have answered all the questions.

If you have any other comments or there is anything else you would like to tell us about when you first noticed your symptoms, please do so using the space below.

If you feel you would like to talk to someone about this questionnaire at a later stage, please speak to the doctor or nurse who is looking after you, or be in touch with the research team, whose contact details are on your patient information sheet

Thank you for completing the questionnaire

Please give it to the research nurse

The next page is for the Researcher to complete

Researcher - Post-completion Sheet

Researcher initials: ☐☐☐

Date questionnaire completed: Date ☐☐ / ☐☐ / ☐☐ Time ☐☐ : ☐☐

If not completed, give reason ☐

How long is it since the patient was told of his/her diagnosis? ☐

How long did it take the patient to complete the questionnaire?

- Less than 5 minutes ☐
- Between 5 and 10 minutes ☐
- Between 11 and 15 minutes ☐
- Longer than 15 minutes ☐

For the patient selected to <u>complete the questionnaire on their own</u>

Did the patient ask for any help? ☐ Yes ☐ No

If yes,

please specify what help was requested

___

___

please specify what help was given

___

___

For the patient selected for <u>the researcher-administered questionnaire</u>

Did the patient find any of the questions difficult? ☐ Yes ☐ No

If yes,

please specify which questions and what the difficulty was

___

___

___

___

Was the patient made anxious? ☐ Yes ☐ No

If yes,

please give details

___

___

___

___

Where did the patient complete the questionnaire?

(Please specify whether this was in a separate room alone, in a corner of the clinic, the ward or other

___

Thank you. Please now send the questionnaire and patient information sheet and consent form back to Cardiff University in the freepost envelope provided.

57727

PID - 82

Ymddiriedolaeth GIG Gogledd Orllewin Cymru
North West Wales NHS Trust

NHS WALES GIG CYMRU
Ymddiriedolaeth GIG Gogledd Cymru
North Wales NHS Trust

CID - ☐ GLAN CLWYD
☐ GWYNEDD
☐ MAELOR

Confidential Questionnaire

Looking At Your Symptoms Study

This Study has been funded by Cancer Research UK

Cardiff University / Prifysgol Caerdydd
Ysgol Glinigol Gogledd Cymru / North Wales Clinical School
Cancer Research UK
sewtu — South East Wales Trials Unit / Uned Ymchwil De-ddwyrain Cymru

Dear Patient

I am very grateful to you for agreeing to help us with this study.

I hope the information you supply will help us give people a diagnosis earlier.

If you feel you would like to talk to someone about this questionnaire at a later stage, please speak to the doctor or nurse who is looking after you, or be in touch with the research team, whose contact details are on your patient information sheet.

I can assure you that anything you tell us will be treated in the utmost confidence.

Yours sincerely

Richard Neal

Dr Richard Neal
Senior Clinical Lecturer
Cardiff University
Chief Investigator, Symptoms Study

About your symptoms

These questions are about symptoms you noticed before your diagnosis. We are interested in symptoms which you think are related to your cancer.

Please put a cross in the appropriate box, or write an answer as required.

Looking back...

1. Did you have any symptoms that you feel were due to your cancer before you were diagnosed?

Yes ☐ **Please continue with Question 2**

No ☐ **Please go to Section 2 on Page 5**

2. What was the first symptom that made you think something might be wrong?

[ ]

Please go to Question 3

The following questions are about when you first noticed a symptom and when you first told your GP or nurse about it.

Please give an exact date if you can. Otherwise give your best estimate. (For example approximately how long ago, the month or season). You wish to refer to your diary or calendar if you have it with you.

Here is an example question:

Decrease in appetite

Did you have this symptom?

Yes ☒ **Please complete A then B or C below**

No ☐ **Please go to Section 2**

A When did you first notice this? d d / m m / y y **OR** Estimate '3 months ago' or 'June'

B When did you first tell your GP or nurse? 2 3 / 1 2 / 0 7 **OR** Estimate

OR

C Put a cross here if you didn't tell your GP or nurse ☐

3. Change in bowel habit

Did you have this symptom?

Yes ☐ **Please complete A then B or C below**

No ☐ **Please go to Question 4**

A When did you first notice this? ☐☐ / ☐☐ / ☐☐ **OR** ☐

B When did you first tell your GP or nurse? ☐☐ / ☐☐ / ☐☐ **OR** ☐

OR

C Put a cross here if you didn't tell your GP or nurse ☐

4. Bleeding from back passage

Did you have this symptom?

Yes ☐ **Please complete A then B or C below**

No ☐ **Please go to Section 2**

A When did you first notice this? ☐☐ / ☐☐ / ☐☐ **OR** ☐

B When did you first tell your GP or nurse? ☐☐ / ☐☐ / ☐☐ **OR** ☐

OR

C Put a cross here if you didn't tell your GP or nurse ☐

Section 2

Please give an exact date if you can. Otherwise give your best estimate (for example approximately how long ago, the month or the season). You may wish to refer to your diary or calendar if you have it with you.

Did you have any of the following?

1. Decrease in appetite

Did you have this?

Yes ☐ **Please complete A then B or C below**

No ☐ **Please go to Question 2**

A When did you first notice this? ☐☐ / ☐☐ / ☐☐ **OR** ☐

B When did you first tell your GP or nurse? ☐☐ / ☐☐ / ☐☐ **OR** ☐

OR

C Put a cross here if you didn't tell your GP or nurse ☐

2. Unexplained weight loss

Did you have this?

Yes ☐ **Please complete A then B or C below**

No ☐ **Please go to Question 3**

A When did you first notice this? ☐☐ / ☐☐ / ☐☐ **OR** ☐

B When did you first tell your GP or nurse? ☐☐ / ☐☐ / ☐☐ **OR** ☐

OR

C Put a cross here if you didn't tell your GP or nurse ☐

3. Fatigue or tiredness that is unusual for you

Did you have this?

Yes ☐ **Please complete A then B or C below**

No ☐ **Please go to Question 4**

A When did you first notice this? ☐☐ / ☐☐ / ☐☐ **OR** ☐

B When did you first tell your GP or nurse? ☐☐ / ☐☐ / ☐☐ **OR** ☐

OR

C Put a cross here if you didn't tell your GP or nurse ☐

4. Feeling different 'in yourself' from usual

Did you have this?

Yes ☐ **Please complete A then B or C below**

No ☐ **Please go to Question 5**

A When did you first notice this? ☐☐ / ☐☐ / ☐☐ **OR** ☐

B When did you first tell your GP or nurse? ☐☐ / ☐☐ / ☐☐ **OR** ☐

OR

C Put a cross here if you didn't tell your GP or nurse ☐

Please try and describe what the feeling was

If you had any further symptoms which you feel are relevant to your cancer diagnosis, please list them below. If not, please go to Question 7.

Please describe the symptom here and complete A then B or C below

5.

A When did you first notice this? ☐☐ / ☐☐ / ☐☐ **OR** ☐

B When did you first tell your GP or nurse? ☐☐ / ☐☐ / ☐☐ **OR** ☐

OR

C Put a cross here if you didn't tell your GP or nurse ☐

If you had another symptom you feel is relevant, please write it below, otherwise go to Question 7.

Please describe the symptom here and complete A then B or C below

6.

A When did you first notice this? ☐☐ / ☐☐ / ☐☐ **OR** ☐

B When did you first tell your GP or nurse? ☐☐ / ☐☐ / ☐☐ **OR** ☐

OR

C Put a cross here if you didn't tell your GP or nurse ☐

If you had further symptoms you would like to tell us about, there is space at the end of the Questionnaire for you to write them.

Did your GP send you for any tests?

Thinking back to the time immediately before you diagnosis, did your GP send you for any of the following tests?

Please cross the appropriate boxes

7. Were you sent for any of these?

	Yes	Not sure	No
Blood test(s)	☐	☐	☐
CT Scan	☐	☐	☐
Ultrasound scan	☐	☐	☐
Barium Enema	☐	☐	☐
Sigmoidoscopy or colonoscopy (Looking at bowel with internal camera)	☐	☐	☐
X-ray	☐	☐	☐

Please go to Question 8

About you

The next questions relate to general information about you. It would help us to know more about the background of the people completing the questionnaire.

8. Which best describes your employment status?

Please cross one box only

- Employed full-time ☐
- Employed part-time ☐
- Self employed full-time ☐
- Self employed part-time ☐
- Unemployed (seeking work) ☐
- Unemployed (not seeking work) ☐
- Retired ☐
- Student ☐
- Permanently sick/disabled ☐
- Temporarily sick/disabled ☐
- Looking after family/home ☐

Other, please describe ____________

9. What is your highest level of qualification?

Please cross one box only

- Degree (or equivalent) ☐
- Diploma (or equivalent) ☐
- 'A' level ☐
- GCSE / 'O' level ☐
- None ☐

Other, please specify ____________

10. How would you describe your ethnicity

Please cross one box only

White ☐
or
- White British ☐
- White Irish ☐
- Other White background ☐

Mixed ☐
or
- White & Black Carribean ☐
- White & Black African ☐
- White & Asian ☐
- Other mixed background ☐

Black or Black British ☐
or
- Carribean ☐
- African ☐
- Other Black background ☐

Asian or Asian British ☐
or
- Indian ☐
- Pakistani ☐
- Bangladeshi ☐
- Other Asian background ☐

Chinese or other ethnic group ☐
or
- Chinese ☐
- Other ethnic group ☐

11.

Do you live alone?

Yes ☐ **Please go to Question 12**

No ☐ **Please say who you live with below**

Who do you live with?
(e.g. wife, husband, partner, family member)

12. **Are you suffering from, or have you suffered from, any of the following in the past 2 years?**

Please cross any that are applicable to you

- Asthma ☐
- Chronic Obstructive Pulmonary Disease (COPD) ☐
- Other lung disease (e.g. fibrosis, bronchiectatis etc) ☐
- Heart disease ☐
- Anxiety or depression ☐
- Inflammatory bowel disease ☐
- Irritable bowel syndrome ☐
- Peptic ulcer ☐
- Previous cancer ☐
- Diabetes ☐
- Arthritis ☐

13. **About smoking**

Please cross the appropriate statement

- Are you a current smoker? ☐
- Are you an ex-smoker? ☐
- Are you a non-smoker (never smoked)? ☐

14. **Do you think you were more at risk of getting cancer because of your family history?**

Yes ☐
No ☐

15. **The following questions are about how completing the questionnaire made you feel.**

A number of statements which people have used to describe themselves are given below. Read each statement and then cross the most appropriate box to indicate how you feel right now, at the moment. There are no right or wrong answers. Do not spend too much time on any one statement but give the answer which seems to describe your present feeling best.

		Not at all	Somewhat	Moderately	Very much
1.	I feel calm	☐	☐	☐	☐
2.	I am tense	☐	☐	☐	☐
3.	I feel upset	☐	☐	☐	☐
4.	I am relaxed	☐	☐	☐	☐
5.	I feel content	☐	☐	☐	☐
6.	I am worried	☐	☐	☐	☐

Please make sure you have answered all the questions.

If you have any other comments or there is anything else you would like to tell us about when you first noticed your symptoms, please do so using the space below.

If you feel you would like to talk to someone about this questionnaire at a later stage, please speak to the doctor or nurse who is looking after you, or be in touch with the research team, whose contact details are on your patient information sheet

Thank you for completing the questionnaire

Please give it to the research nurse

The next page is for the Researcher to complete

Researcher - Post-completion Sheet

Researcher initials: ☐☐☐

Date questionnaire completed: Date ☐☐ / ☐☐ / ☐☐ Time ☐☐:☐☐

If not completed, give reason ☐

How long is it since the patient was told of his/her diagnosis? ☐

How long did it take the patient to complete the questionnaire?

- Less than 5 minutes ☐
- Between 5 and 10 minutes ☐
- Between 11 and 15 minutes ☐
- Longer than 15 minutes ☐

For the patient selected to complete the questionnaire on their own

Did the patient ask for any help? ☐ Yes ☐ No

If yes,

please specify what help was requested

____

____

please specify what help was given

____

____

For the patient selected for the researcher-administered questionnaire

Did the patient find any of the questions difficult? ☐ Yes ☐ No

If yes,

please specify which questions and what the difficulty was

____

____

____

____

Was the patient made anxious? ☐ Yes ☐ No

If yes,

please give details

____

____

____

____

Where did the patient complete the questionnaire?

(Please specify whether this was in a separate room alone, in a corner of the clinic, the ward or other

____

Thank you. Please now send the questionnaire and patient information sheet and consent form back to Cardiff University in the freepost envelope provided.

57727

PID - 83

Ymddiriedolaeth GIG Gogledd Orllewin Cymru
North West Wales NHS Trust

NHS WALES GIG CYMRU
Ymddiriedolaeth GIG Gogledd Cymru
North Wales NHS Trust

☐ GLAN CLWYD

CID - ☐ GWYNEDD

☐ MAELOR

Confidential Questionnaire

Looking At Your Symptoms Study

This Study has been funded by Cancer Research UK

Cardiff University / Prifysgol Caerdydd
Ysgol Glinigol Gogledd Cymru / North Wales Clinical School
Cancer Research UK
sewtu South East Wales Trials Unit / Uned Ymchwil De-ddwyrain Cymru

Dear Patient

I am very grateful to you for agreeing to help us with this study.

I hope the information you supply will help us give people a diagnosis earlier.

If you feel you would like to talk to someone about this questionnaire at a later stage, please speak to the doctor or nurse who is looking after you, or be in touch with the research team, whose contact details are on your patient information sheet.

I can assure you that anything you tell us will be treated in the utmost confidence.

Yours sincerely

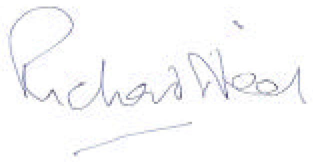

Dr Richard Neal
Senior Clinical Lecturer
Cardiff University
Chief Investigator, Symptoms Study

About your symptoms

These questions are about symptoms you noticed before your diagnosis. We are interested in symptoms which you think are related to your cancer.

Please put a cross in the appropriate box, or write an answer as required.

Looking back...

1. Did you have any symptoms that you feel were due to your cancer before you were diagnosed?

Yes ☐ **Please continue with Question 2**

No ☐ **Please go to Section 2 on Page 5**

2. What was the first symptom that made you think something might be wrong?

Please go to Question 3

The following questions are about when you first noticed a symptom and when you first told your GP or nurse about it.

Please give an exact date if you can. Otherwise give your best estimate. (For example approximately how long ago, the month or season). You wish to refer to your diary or calendar if you have it with you.

Here is an example question:

Decrease in appetite

Did you have this symptom?

Yes ☒ **Please complete A then B or C below**

No ☐ **Please go to Section 2**

A When did you first notice this? d d / m m / y y **OR** Estimate '3 months ago' or 'June'

B When did you first tell your GP or nurse? 2 3 / 1 2 / 0 7 **OR** Estimate

OR

C Put a cross here if you didn't tell your GP or nurse ☐

3. Change in bowel habit

Did you have this symptom?

Yes ☐ **Please complete A then B or C below**

No ☐ **Please go to Question 4**

A When did you first notice this? ☐☐ / ☐☐ / ☐☐ **OR** ☐

B When did you first tell your GP or nurse? ☐☐ / ☐☐ / ☐☐ **OR** ☐

OR

C Put a cross here if you didn't tell your GP or nurse ☐

4. Bleeding from back passage

Did you have this symptom?

Yes ☐ **Please complete A then B or C below**

No ☐ **Please go to Section 2**

A When did you first notice this? ☐☐ / ☐☐ / ☐☐ **OR** ☐

B When did you first tell your GP or nurse? ☐☐ / ☐☐ / ☐☐ **OR** ☐

OR

C Put a cross here if you didn't tell your GP or nurse ☐

Section 2

Please give an exact date if you can. Otherwise give your best estimate (for example approximately how long ago, the month or the season). You may wish to refer to your diary or calendar if you have it with you.

Did you have any of the following?

1. Decrease in appetite

Did you have this?

Yes ☐ **Please complete A then B or C below**

No ☐ **Please go to Question 2**

A When did you first notice this? ☐☐ / ☐☐ / ☐☐ **OR** ☐

B When did you first tell your GP or nurse? ☐☐ / ☐☐ / ☐☐ **OR** ☐

OR

C Put a cross here if you didn't tell your GP or nurse ☐

2. Unexplained weight loss

Did you have this?

Yes ☐ **Please complete A then B or C below**

No ☐ **Please go to Question 3**

A When did you first notice this? ☐☐ / ☐☐ / ☐☐ **OR** ☐

B When did you first tell your GP or nurse? ☐☐ / ☐☐ / ☐☐ **OR** ☐

OR

C Put a cross here if you didn't tell your GP or nurse ☐

3. **Fatigue or tiredness that is unusual for you**

Did you have this?

Yes ☐ **Please complete A then B or C below**

No ☐ **Please go to Question 4**

A When did you first notice this? ☐☐ / ☐☐ / ☐☐ **OR** ☐

B When did you first tell your GP or nurse? ☐☐ / ☐☐ / ☐☐ **OR** ☐

OR

C Put a cross here if you didn't tell your GP or nurse ☐

4. **Feeling different 'in yourself' from usual**

Did you have this?

Yes ☐ **Please complete A then B or C below**

No ☐ **Please go to Question 5**

A When did you first notice this? ☐☐ / ☐☐ / ☐☐ **OR** ☐

B When did you first tell your GP or nurse? ☐☐ / ☐☐ / ☐☐ **OR** ☐

OR

C Put a cross here if you didn't tell your GP or nurse ☐

Please try and describe what the feeling was

If you had any further symptoms which you feel are relevant to your cancer diagnosis, please list them below. If not, please go to Question 7.

Please describe the symptom here and complete A then B or C below

5. ______________________

A When did you first notice this? ☐☐ / ☐☐ / ☐☐ **OR** ☐

B When did you first tell your GP or nurse? ☐☐ / ☐☐ / ☐☐ **OR** ☐

OR

C Put a cross here if you didn't tell your GP or nurse ☐

If you had another symptom you feel is relevant, please write it below, otherwise go to Question 7.

Please describe the symptom here and complete A then B or C below

6. ______________________

A When did you first notice this? ☐☐ / ☐☐ / ☐☐ **OR** ☐

B When did you first tell your GP or nurse? ☐☐ / ☐☐ / ☐☐ **OR** ☐

OR

C Put a cross here if you didn't tell your GP or nurse ☐

If you had further symptoms you would like to tell us about, there is space at the end of the Questionnaire for you to write them.

Did your GP send you for any tests?

Thinking back to the time immediately before you diagnosis, did your GP send you for any of the following tests?

Please cross the appropriate boxes

7. Were you sent for any of these?

	Yes	Not sure	No
Blood test(s)	☐	☐	☐
CT Scan	☐	☐	☐
Ultrasound scan	☐	☐	☐
Barium Enema	☐	☐	☐
Sigmoidoscopy or colonoscopy (Looking at bowel with internal camera)	☐	☐	☐
X-ray	☐	☐	☐

Please go to Question 8

About you

The next questions relate to general information about you. It would help us to know more about the background of the people completing the questionnaire.

8. Which best describes your employment status?

Please cross one box only

- Employed full-time ☐
- Employed part-time ☐
- Self employed full-time ☐
- Self employed part-time ☐
- Unemployed (seeking work) ☐
- Unemployed (not seeking work) ☐
- Retired ☐
- Student ☐
- Permanently sick/disabled ☐
- Temporarily sick/disabled ☐
- Looking after family/home ☐
- Other, please describe ____________

9. What is your highest level of qualification?

Please cross one box only

- Degree (or equivalent) ☐
- Diploma (or equivalent) ☐
- 'A' level ☐
- GCSE / 'O' level ☐
- None ☐
- Other, please specify ____________

10. **How would you describe your ethnicity**

Please cross one box only

White ☐
or
- White British ☐
- White Irish ☐
- Other White background ☐

Mixed ☐
or
- White & Black Carribean ☐
- White & Black African ☐
- White & Asian ☐
- Other mixed background ☐

Black or Black British ☐
or
- Carribean ☐
- African ☐
- Other Black background ☐

Asian or Asian British ☐
or
- Indian ☐
- Pakistani ☐
- Bangladeshi ☐
- Other Asian background ☐

Chinese or other ethnic group ☐
or
- Chinese ☐
- Other ethnic group ☐

11.

Do you live alone?

Yes ☐ **Please go to Question 12**

No ☐ **Please say who you live with below**

Who do you live with?
(e.g. wife, husband, partner, family member)

12. **Are you suffering from, or have you suffered from, any of the following in the past 2 years?**

Please cross any that are applicable to you

- Asthma ☐
- Chronic Obstructive Pulmonary Disease (COPD) ☐
- Other lung disease (e.g. fibrosis, bronchiectatis etc) ☐
- Heart disease ☐
- Anxiety or depression ☐
- Inflammatory bowel disease ☐
- Irritable bowel syndrome ☐
- Peptic ulcer ☐
- Previous cancer ☐
- Diabetes ☐
- Arthritis ☐

13. **About smoking**

Please cross the appropriate statement

- Are you a current smoker? ☐
- Are you an ex-smoker? ☐
- Are you a non-smoker (never smoked)? ☐

14. **Do you think you were more at risk of getting cancer because of your family history?**

Yes ☐

No ☐

15. **The following questions are about how completing the questionnaire made you feel.**

A number of statements which people have used to describe themselves are given below. Read each statement and then cross the most appropriate box to indicate how you feel right now, at the moment. There are no right or wrong answers. Do not spend too much time on any one statement but give the answer which seems to describe your present feeling best.

		Not at all	Somewhat	Moderately	Very much
1.	I feel calm	☐	☐	☐	☐
2.	I am tense	☐	☐	☐	☐
3.	I feel upset	☐	☐	☐	☐
4.	I am relaxed	☐	☐	☐	☐
5.	I feel content	☐	☐	☐	☐
6.	I am worried	☐	☐	☐	☐

Please make sure you have answered all the questions.

If you have any other comments or there is anything else you would like to tell us about when you first noticed your symptoms, please do so using the space below.

If you feel you would like to talk to someone about this questionnaire at a later stage, please speak to the doctor or nurse who is looking after you, or be in touch with the research team, whose contact details are on your patient information sheet

Thank you for completing the questionnaire

Please give it to the research nurse

The next page is for the Researcher to complete

Researcher - Post-completion Sheet

Researcher initials: ☐☐☐

Date questionnaire completed: Date ☐☐ / ☐☐ / ☐☐ Time ☐☐:☐☐

If not completed, give reason ☐

How long is it since the patient was told of his/her diagnosis? ☐

How long did it take the patient to complete the questionnaire?

- Less than 5 minutes ☐
- Between 5 and 10 minutes ☐
- Between 11 and 15 minutes ☐
- Longer than 15 minutes ☐

For the patient selected to <u>complete the questionnaire on their own</u>

Did the patient ask for any help? ☐ Yes ☐ No

If yes,

please specify what help was requested

____

____

please specify what help was given

____

____

For the patient selected for <u>the researcher-administered questionnaire</u>

Did the patient find any of the questions difficult? ☐ Yes ☐ No

If yes,

please specify which questions and what the difficulty was

____

____

____

____

Was the patient made anxious? ☐ Yes ☐ No

If yes,

please give details

____

____

____

____

Where did the patient complete the questionnaire?
(Please specify whether this was in a separate room alone, in a corner of the clinic, the ward or other

____

Thank you. Please now send the questionnaire and patient information sheet and consent form back to Cardiff University in the freepost envelope provided.

57727

PID - | 8 | 4

Ymddiriedolaeth GIG Gogledd Orllewin Cymru
North West Wales NHS Trust

NHS WALES GIG CYMRU
Ymddiriedolaeth GIG Gogledd Cymru
North Wales NHS Trust

CID -
☐ GLAN CLWYD
☐ GWYNEDD
☐ MAELOR

Confidential Questionnaire

Looking At Your Symptoms Study

This Study has been funded by Cancer Research UK

Cardiff University / Prifysgol Caerdydd
Ysgol Glinigol Gogledd Cymru / North Wales Clinical School
Cancer Research UK
sewtu South East Wales Trials Unit / Uned Ymchwil De-ddwyrain Cymru

Dear Patient

I am very grateful to you for agreeing to help us with this study.

I hope the information you supply will help us give people a diagnosis earlier.

If you feel you would like to talk to someone about this questionnaire at a later stage, please speak to the doctor or nurse who is looking after you, or be in touch with the research team, whose contact details are on your patient information sheet.

I can assure you that anything you tell us will be treated in the utmost confidence.

Yours sincerely

Dr Richard Neal
Senior Clinical Lecturer
Cardiff University
Chief Investigator, Symptoms Study

About your symptoms

These questions are about symptoms you noticed before your diagnosis. We are interested in symptoms which you think are related to your cancer.

Please put a cross in the appropriate box, or write an answer as required.

Looking back...

1. Did you have any symptoms that you feel were due to your cancer before you were diagnosed?

Yes ☐ **Please continue with Question 2**

No ☐ **Please go to Section 2 on Page 5**

2. What was the first symptom that made you think something might be wrong?

[ ]

Please go to Question 3

The following questions are about when you first noticed a symptom and when you first told your GP or nurse about it.

Please give an exact date if you can. Otherwise give your best estimate. (For example approximately how long ago, the month or season). You wish to refer to your diary or calendar if you have it with you.

Here is an example question:

Decrease in appetite

Did you have this symptom?

Yes ☒ **Please complete A then B or C below**

No ☐ **Please go to Section 2**

A When did you first notice this? d d / m m / y y **OR** Estimate '3 months ago' or 'June'

B When did you first tell your GP or nurse? 2 3 / 1 2 / 0 7 **OR** Estimate

OR

C Put a cross here if you didn't tell your GP or nurse ☐

3. Change in bowel habit

Did you have this symptom?

Yes ☐ **Please complete A then B or C below**

No ☐ **Please go to Question 4**

A When did you first notice this? ☐☐ / ☐☐ / ☐☐ **OR** ☐

B When did you first tell your GP or nurse? ☐☐ / ☐☐ / ☐☐ **OR** ☐

OR

C Put a cross here if you didn't tell your GP or nurse ☐

4. Bleeding from back passage

Did you have this symptom?

Yes ☐ **Please complete A then B or C below**

No ☐ **Please go to Section 2**

A When did you first notice this? ☐☐ / ☐☐ / ☐☐ **OR** ☐

B When did you first tell your GP or nurse? ☐☐ / ☐☐ / ☐☐ **OR** ☐

OR

C Put a cross here if you didn't tell your GP or nurse ☐

Section 2

Please give an exact date if you can. Otherwise give your best estimate (for example approximately how long ago, the month or the season). You may wish to refer to your diary or calendar if you have it with you.

Did you have any of the following?

1. Decrease in appetite

Did you have this?

Yes ☐ **Please complete A then B or C below**

No ☐ **Please go to Question 2**

A When did you first notice this? ☐☐ / ☐☐ / ☐☐ **OR** ☐

B When did you first tell your GP or nurse? ☐☐ / ☐☐ / ☐☐ **OR** ☐

OR

C Put a cross here if you didn't tell your GP or nurse ☐

2. Unexplained weight loss

Did you have this?

Yes ☐ **Please complete A then B or C below**

No ☐ **Please go to Question 3**

A When did you first notice this? ☐☐ / ☐☐ / ☐☐ **OR** ☐

B When did you first tell your GP or nurse? ☐☐ / ☐☐ / ☐☐ **OR** ☐

OR

C Put a cross here if you didn't tell your GP or nurse ☐

3. Fatigue or tiredness that is unusual for you

Did you have this?

Yes ☐ **Please complete A then B or C below**

No ☐ **Please go to Question 4**

A When did you first notice this? __/__/__ **OR** ____

B When did you first tell your GP or nurse? __/__/__ **OR** ____

OR

C Put a cross here if you didn't tell your GP or nurse ☐

4. Feeling different 'in yourself' from usual

Did you have this?

Yes ☐ **Please complete A then B or C below**

No ☐ **Please go to Question 5**

A When did you first notice this? __/__/__ **OR** ____

B When did you first tell your GP or nurse? __/__/__ **OR** ____

OR

C Put a cross here if you didn't tell your GP or nurse ☐

Please try and describe what the feeling was

If you had any further symptoms which you feel are relevant to your cancer diagnosis, please list them below. If not, please go to Question 7.

Please describe the symptom here and complete A then B or C below

5.

**A**	When did you first notice this?	__/__/__ **OR**
**B**	When did you first tell your GP or nurse?	__/__/__ **OR**
	**OR**	
**C**	Put a cross here if you didn't tell your GP or nurse	☐

If you had another symptom you feel is relevant, please write it below, otherwise go to Question 7.

Please describe the symptom here and complete A then B or C below

6.

**A**	When did you first notice this?	__/__/__ **OR**
**B**	When did you first tell your GP or nurse?	__/__/__ **OR**
	**OR**	
**C**	Put a cross here if you didn't tell your GP or nurse	☐

If you had further symptoms you would like to tell us about, there is space at the end of the Questionnaire for you to write them.

Did your GP send you for any tests?

Thinking back to the time immediately before you diagnosis, did your GP send you for any of the following tests?

Please cross the appropriate boxes

7. Were you sent for any of these?

	Yes	Not sure	No
Blood test(s)	☐	☐	☐
CT Scan	☐	☐	☐
Ultrasound scan	☐	☐	☐
Barium Enema	☐	☐	☐
Sigmoidoscopy or colonoscopy (Looking at bowel with internal camera)	☐	☐	☐
X-ray	☐	☐	☐

Please go to Question 8

About you

The next questions relate to general information about you. It would help us to know more about the background of the people completing the questionnaire.

8. **Which best describes your employment status?**

Please cross one box only

- Employed full-time ☐
- Employed part-time ☐
- Self employed full-time ☐
- Self employed part-time ☐
- Unemployed (seeking work) ☐
- Unemployed (not seeking work) ☐
- Retired ☐
- Student ☐
- Permanently sick/disabled ☐
- Temporarily sick/disabled ☐
- Looking after family/home ☐
- Other, please describe ________

9. **What is your highest level of qualification?**

Please cross one box only

- Degree (or equivalent) ☐
- Diploma (or equivalent) ☐
- 'A' level ☐
- GCSE / 'O' level ☐
- None ☐
- Other, please specify ________

10. **How would you describe your ethnicity**

Please cross one box only

White ☐
or
White British ☐
White Irish ☐
Other White background ☐

Mixed ☐
or
White & Black Carribean ☐
White & Black African ☐
White & Asian ☐
Other mixed background ☐

Black or Black British ☐
or
Carribean ☐
African ☐
Other Black background ☐

Asian or Asian British ☐
or
Indian ☐
Pakistani ☐
Bangladeshi ☐
Other Asian background ☐

Chinese or other ethnic group ☐
or
Chinese ☐
Other ethnic group ☐

11.

Do you live alone?

Yes ☐ **Please go to Question 12**

No ☐ **Please say who you live with below**

Who do you live with?
(e.g. wife, husband, partner, family member)

12. Are you suffering from, or have you suffered from, any of the following in the past 2 years?

Please cross any that are applicable to you

Asthma	☐
Chronic Obstructive Pulmonary Disease (COPD)	☐
Other lung disease (e.g. fibrosis, bronchiectatis etc)	☐
Heart disease	☐
Anxiety or depression	☐
Inflammatory bowel disease	☐
Irritable bowel syndrome	☐
Peptic ulcer	☐
Previous cancer	☐
Diabetes	☐
Arthritis	☐

13. About smoking

Please cross the appropriate statement

Are you a current smoker?	☐
Are you an ex-smoker?	☐
Are you a non-smoker (never smoked)?	☐

14. Do you think you were more at risk of getting cancer because of your family history?

Yes ☐

No ☐

15. **The following questions are about how completing the questionnaire made you feel.**

A number of statements which people have used to describe themselves are given below. Read each statement and then cross the most appropriate box to indicate how you feel right now, at the moment. There are no right or wrong answers. Do not spend too much time on any one statement but give the answer which seems to describe your present feeling best.

		Not at all	Somewhat	Moderately	Very much
1.	I feel calm	☐	☐	☐	☐
2.	I am tense	☐	☐	☐	☐
3.	I feel upset	☐	☐	☐	☐
4.	I am relaxed	☐	☐	☐	☐
5.	I feel content	☐	☐	☐	☐
6.	I am worried	☐	☐	☐	☐

Please make sure you have answered all the questions.

If you have any other comments or there is anything else you would like to tell us about when you first noticed your symptoms, please do so using the space below.

If you feel you would like to talk to someone about this questionnaire at a later stage, please speak to the doctor or nurse who is looking after you, or be in touch with the research team, whose contact details are on your patient information sheet

Thank you for completing the questionnaire

Please give it to the research nurse

The next page is for the Researcher to complete

57727

84

Researcher - Post-completion Sheet

Researcher initials: ☐☐☐

Date questionnaire completed: Date ☐☐ / ☐☐ / ☐☐ Time ☐☐:☐☐

If not completed, give reason ☐

How long is it since the patient was told of his/her diagnosis? ☐

How long did it take the patient to complete the questionnaire?

- Less than 5 minutes ☐
- Between 5 and 10 minutes ☐
- Between 11 and 15 minutes ☐
- Longer than 15 minutes ☐

For the patient selected to complete the questionnaire on their own

Did the patient ask for any help? ☐ Yes ☐ No

If yes,

please specify what help was requested

____

____

please specify what help was given

____

____

For the patient selected for the researcher-administered questionnaire

Did the patient find any of the questions difficult? ☐ Yes ☐ No

If yes,

please specify which questions and what the difficulty was

____

____

____

____

Was the patient made anxious? ☐ Yes ☐ No

If yes,

please give details

____

____

____

____

Where did the patient complete the questionnaire?
(Please specify whether this was in a separate room alone, in a corner of the clinic, the ward or other

____

Thank you. Please now send the questionnaire and patient information sheet and consent form back to Cardiff University in the freepost envelope provided.

57727

PID - 8 5

Ymddiriedolaeth GIG Gogledd Orllewin Cymru
North West Wales NHS Trust

NHS WALES GIG CYMRU
Ymddiriedolaeth GIG Gogledd Cymru
North Wales NHS Trust

CID -
- [ ] GLAN CLWYD
- [ ] GWYNEDD
- [ ] MAELOR

Confidential Questionnaire

Looking At Your Symptoms Study

This Study has been funded by Cancer Research UK

Cardiff University / Prifysgol Caerdydd — Ysgol Glinigol Gogledd Cymru / North Wales Clinical School — Cancer Research UK — sewtu South East Wales Trials Unit / Uned Ymchwil De-ddwyrain Cymru

Dear Patient

I am very grateful to you for agreeing to help us with this study.

I hope the information you supply will help us give people a diagnosis earlier.

If you feel you would like to talk to someone about this questionnaire at a later stage, please speak to the doctor or nurse who is looking after you, or be in touch with the research team, whose contact details are on your patient information sheet.

I can assure you that anything you tell us will be treated in the utmost confidence.

Yours sincerely

Dr Richard Neal
Senior Clinical Lecturer
Cardiff University
Chief Investigator, Symptoms Study

About your symptoms

These questions are about symptoms you noticed before your diagnosis. We are interested in symptoms which you think are related to your cancer.

Please put a cross in the appropriate box, or write an answer as required.

Looking back...

1. Did you have any symptoms that you feel were due to your cancer before you were diagnosed?

Yes ☐ **Please continue with Question 2**

No ☐ **Please go to Section 2 on Page 5**

2. What was the first symptom that made you think something might be wrong?

[ ]

Please go to Question 3

The following questions are about when you first noticed a symptom and when you first told your GP or nurse about it.

Please give an exact date if you can. Otherwise give your best estimate. (For example approximately how long ago, the month or season). You wish to refer to your diary or calendar if you have it with you.

Here is an example question:

Decrease in appetite

Did you have this symptom?

Yes ☒ **Please complete A then B or C below**

No ☐ **Please go to Section 2**

A When did you first notice this? d d / m m / y y **OR** Estimate '3 months ago' or 'June'

B When did you first tell your GP or nurse? 2 3 / 1 2 / 0 7 **OR** Estimate

OR

C Put a cross here if you didn't tell your GP or nurse ☐

3. Change in bowel habit

Did you have this symptom?

Yes ☐ **Please complete A then B or C below**

No ☐ **Please go to Question 4**

A When did you first notice this? ☐☐ / ☐☐ / ☐☐ **OR** ☐

B When did you first tell your GP or nurse? ☐☐ / ☐☐ / ☐☐ **OR** ☐

OR

C Put a cross here if you didn't tell your GP or nurse ☐

4. Bleeding from back passage

Did you have this symptom?

Yes ☐ **Please complete A then B or C below**

No ☐ **Please go to Section 2**

A When did you first notice this? ☐☐ / ☐☐ / ☐☐ **OR** ☐

B When did you first tell your GP or nurse? ☐☐ / ☐☐ / ☐☐ **OR** ☐

OR

C Put a cross here if you didn't tell your GP or nurse ☐

Section 2

Please give an exact date if you can. Otherwise give your best estimate (for example approximately how long ago, the month or the season). You may wish to refer to your diary or calendar if you have it with you.

Did you have any of the following?

1. Decrease in appetite

Did you have this?

Yes ☐ **Please complete A then B or C below**

No ☐ **Please go to Question 2**

A When did you first notice this? ☐☐ / ☐☐ / ☐☐ **OR** ☐

B When did you first tell your GP or nurse? ☐☐ / ☐☐ / ☐☐ **OR** ☐

OR

C Put a cross here if you didn't tell your GP or nurse ☐

2. Unexplained weight loss

Did you have this?

Yes ☐ **Please complete A then B or C below**

No ☐ **Please go to Question 3**

A When did you first notice this? ☐☐ / ☐☐ / ☐☐ **OR** ☐

B When did you first tell your GP or nurse? ☐☐ / ☐☐ / ☐☐ **OR** ☐

OR

C Put a cross here if you didn't tell your GP or nurse ☐

3. Fatigue or tiredness that is unusual for you

Did you have this?

Yes ☐ **Please complete A then B or C below**

No ☐ **Please go to Question 4**

A When did you first notice this? ☐☐ / ☐☐ / ☐☐ **OR** ☐

B When did you first tell your GP or nurse? ☐☐ / ☐☐ / ☐☐ **OR** ☐

OR

C Put a cross here if you didn't tell your GP or nurse ☐

4. Feeling different 'in yourself' from usual

Did you have this?

Yes ☐ **Please complete A then B or C below**

No ☐ **Please go to Question 5**

A When did you first notice this? ☐☐ / ☐☐ / ☐☐ **OR** ☐

B When did you first tell your GP or nurse? ☐☐ / ☐☐ / ☐☐ **OR** ☐

OR

C Put a cross here if you didn't tell your GP or nurse ☐

Please try and describe what the feeling was

If you had any further symptoms which you feel are relevant to your cancer diagnosis, please list them below. If not, please go to Question 7.

Please describe the symptom here and complete A then B or C below

5.

**A**	When did you first notice this?	☐☐ / ☐☐ / ☐☐ **OR** ☐
**B**	When did you first tell your GP or nurse?	☐☐ / ☐☐ / ☐☐ **OR** ☐
	**OR**	
**C**	Put a cross here if you didn't tell your GP or nurse	☐

If you had another symptom you feel is relevant, please write it below, otherwise go to Question 7.

Please describe the symptom here and complete A then B or C below

6.

**A**	When did you first notice this?	☐☐ / ☐☐ / ☐☐ **OR** ☐
**B**	When did you first tell your GP or nurse?	☐☐ / ☐☐ / ☐☐ **OR** ☐
	**OR**	
**C**	Put a cross here if you didn't tell your GP or nurse	☐

If you had further symptoms you would like to tell us about, there is space at the end of the Questionnaire for you to write them.

Did your GP send you for any tests?

Thinking back to the time immediately before you diagnosis, did your GP send you for any of the following tests?

Please cross the appropriate boxes

7. Were you sent for any of these?

	Yes	Not sure	No
Blood test(s)	☐	☐	☐
CT Scan	☐	☐	☐
Ultrasound scan	☐	☐	☐
Barium Enema	☐	☐	☐
Sigmoidoscopy or colonoscopy (Looking at bowel with internal camera)	☐	☐	☐
X-ray	☐	☐	☐

Please go to Question 8

About you

The next questions relate to general information about you. It would help us to know more about the background of the people completing the questionnaire.

8. Which best describes your employment status?

Please cross one box only

- Employed full-time ☐
- Employed part-time ☐
- Self employed full-time ☐
- Self employed part-time ☐
- Unemployed (seeking work) ☐
- Unemployed (not seeking work) ☐
- Retired ☐
- Student ☐
- Permanently sick/disabled ☐
- Temporarily sick/disabled ☐
- Looking after family/home ☐
- Other, please describe ____

9. What is your highest level of qualification?

Please cross one box only

- Degree (or equivalent) ☐
- Diploma (or equivalent) ☐
- 'A' level ☐
- GCSE / 'O' level ☐
- None ☐
- Other, please specify ____

10. How would you describe your ethnicity

Please cross one box only

White ☐
or
- White British ☐
- White Irish ☐
- Other White background ☐

Mixed ☐
or
- White & Black Carribean ☐
- White & Black African ☐
- White & Asian ☐
- Other mixed background ☐

Black or Black British ☐
or
- Carribean ☐
- African ☐
- Other Black background ☐

Asian or Asian British ☐
or
- Indian ☐
- Pakistani ☐
- Bangladeshi ☐
- Other Asian background ☐

Chinese or other ethnic group ☐
or
- Chinese ☐
- Other ethnic group ☐

11.

Do you live alone?

Yes ☐ **Please go to Question 12**

No ☐ **Please say who you live with below**

Who do you live with?
(e.g. wife, husband, partner, family member)

12. Are you suffering from, or have you suffered from, any of the following in the past 2 years?

Please cross any that are applicable to you

- Asthma ☐
- Chronic Obstructive Pulmonary Disease (COPD) ☐
- Other lung disease (e.g. fibrosis, bronchiectatis etc) ☐
- Heart disease ☐
- Anxiety or depression ☐
- Inflammatory bowel disease ☐
- Irritable bowel syndrome ☐
- Peptic ulcer ☐
- Previous cancer ☐
- Diabetes ☐
- Arthritis ☐

13. About smoking

Please cross the appropriate statement

- Are you a current smoker? ☐
- Are you an ex-smoker? ☐
- Are you a non-smoker (never smoked)? ☐

14. Do you think you were more at risk of getting cancer because of your family history?

Yes ☐

No ☐

15. The following questions are about how completing the questionnaire made you feel.

A number of statements which people have used to describe themselves are given below. Read each statement and then cross the most appropriate box to indicate how you feel right now, at the moment. There are no right or wrong answers. Do not spend too much time on any one statement but give the answer which seems to describe your present feeling best.

		Not at all	Somewhat	Moderately	Very much
1.	I feel calm	☐	☐	☐	☐
2.	I am tense	☐	☐	☐	☐
3.	I feel upset	☐	☐	☐	☐
4.	I am relaxed	☐	☐	☐	☐
5.	I feel content	☐	☐	☐	☐
6.	I am worried	☐	☐	☐	☐

Please make sure you have answered all the questions.

If you have any other comments or there is anything else you would like to tell us about when you first noticed your symptoms, please do so using the space below.

If you feel you would like to talk to someone about this questionnaire at a later stage, please speak to the doctor or nurse who is looking after you, or be in touch with the research team, whose contact details are on your patient information sheet

Thank you for completing the questionnaire

Please give it to the research nurse

The next page is for the Researcher to complete

Researcher - Post-completion Sheet **Researcher initials:** ☐☐☐

Date questionnaire completed: Date ☐☐ / ☐☐ / ☐☐ Time ☐☐:☐☐

If not completed, give reason ☐

How long is it since the patient was told of his/her diagnosis? ☐

How long did it take the patient to complete the questionnaire?

- Less than 5 minutes ☐
- Between 5 and 10 minutes ☐
- Between 11 and 15 minutes ☐
- Longer than 15 minutes ☐

For the patient selected to <u>complete the questionnaire on their own</u>

Did the patient ask for any help? ☐ Yes ☐ No

If yes,

please specify what help was requested

____

____

please specify what help was given

____

____

For the patient selected for <u>the researcher-administered questionnaire</u>

Did the patient find any of the questions difficult? ☐ Yes ☐ No

If yes,

please specify which questions and what the difficulty was

____

____

____

____

Was the patient made anxious? ☐ Yes ☐ No

If yes,

please give details

____

____

____

____

Where did the patient complete the questionnaire?

(Please specify whether this was in a separate room alone, in a corner of the clinic, the ward or other

____

Thank you. Please now send the questionnaire and patient information sheet and consent form back to Cardiff University in the freepost envelope provided.

57727

PID - | 8 | 6 |

Ymddiriedolaeth GIG Gogledd Orllewin Cymru
North West Wales NHS Trust

NHS WALES GIG CYMRU
Ymddiriedolaeth GIG Gogledd Cymru
North Wales NHS Trust

CID -
☐ GLAN CLWYD
☐ GWYNEDD
☐ MAELOR

Confidential Questionnaire

Looking At Your Symptoms Study

This Study has been funded by Cancer Research UK

Cardiff University / Prifysgol Caerdydd
Ysgol Glinigol Gogledd Cymru / North Wales Clinical School
Cancer Research UK
sewtu South East Wales Trials Unit / Uned Ymchwil De-ddwyrain Cymru

Dear Patient

I am very grateful to you for agreeing to help us with this study.

I hope the information you supply will help us give people a diagnosis earlier.

If you feel you would like to talk to someone about this questionnaire at a later stage, please speak to the doctor or nurse who is looking after you, or be in touch with the research team, whose contact details are on your patient information sheet.

I can assure you that anything you tell us will be treated in the utmost confidence.

Yours sincerely

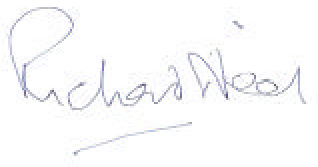

Dr Richard Neal
Senior Clinical Lecturer
Cardiff University
Chief Investigator, Symptoms Study

About your symptoms

These questions are about symptoms you noticed before your diagnosis. We are interested in symptoms which you think are related to your cancer.

Please put a cross in the appropriate box, or write an answer as required.

Looking back...

1. Did you have any symptoms that you feel were due to your cancer before you were diagnosed?

- Yes ☐ **Please continue with Question 2**
- No ☐ **Please go to Section 2 on Page 5**

2. What was the first symptom that made you think something might be wrong?

☐

Please go to Question 3

The following questions are about when you first noticed a symptom and when you first told your GP or nurse about it.

Please give an exact date if you can. Otherwise give your best estimate. (For example approximately how long ago, the month or season). You wish to refer to your diary or calendar if you have it with you.

Here is an example question:

Decrease in appetite

Did you have this symptom?

- Yes ☒ **Please complete A then B or C below**
- No ☐ **Please go to Section 2**

A When did you first notice this? d d / m m / y y **OR** Estimate '3 months ago' or 'June'

B When did you first tell your GP or nurse? 2 3 / 1 2 / 0 7 **OR** Estimate

OR

C Put a cross here if you didn't tell your GP or nurse ☐

3. Change in bowel habit

Did you have this symptom?

Yes ☐ **Please complete A then B or C below**

No ☐ **Please go to Question 4**

A When did you first notice this? ☐☐ / ☐☐ / ☐☐ **OR** ☐

B When did you first tell your GP or nurse? ☐☐ / ☐☐ / ☐☐ **OR** ☐

OR

C Put a cross here if you didn't tell your GP or nurse ☐

4. Bleeding from back passage

Did you have this symptom?

Yes ☐ **Please complete A then B or C below**

No ☐ **Please go to Section 2**

A When did you first notice this? ☐☐ / ☐☐ / ☐☐ **OR** ☐

B When did you first tell your GP or nurse? ☐☐ / ☐☐ / ☐☐ **OR** ☐

OR

C Put a cross here if you didn't tell your GP or nurse ☐

Section 2

Please give an exact date if you can. Otherwise give your best estimate (for example approximately how long ago, the month or the season). You may wish to refer to your diary or calendar if you have it with you.

Did you have any of the following?

1. Decrease in appetite

Did you have this?

Yes ☐ **Please complete A then B or C below**

No ☐ **Please go to Question 2**

A When did you first notice this? ☐☐ / ☐☐ / ☐☐ **OR** ☐

B When did you first tell your GP or nurse? ☐☐ / ☐☐ / ☐☐ **OR** ☐

OR

C Put a cross here if you didn't tell your GP or nurse ☐

2. Unexplained weight loss

Did you have this?

Yes ☐ **Please complete A then B or C below**

No ☐ **Please go to Question 3**

A When did you first notice this? ☐☐ / ☐☐ / ☐☐ **OR** ☐

B When did you first tell your GP or nurse? ☐☐ / ☐☐ / ☐☐ **OR** ☐

OR

C Put a cross here if you didn't tell your GP or nurse ☐

3. **Fatigue or tiredness that is unusual for you**

Did you have this?

Yes ☐ **Please complete A then B or C below**

No ☐ **Please go to Question 4**

A When did you first notice this? ☐☐ / ☐☐ / ☐☐ **OR** ☐

B When did you first tell your GP or nurse? ☐☐ / ☐☐ / ☐☐ **OR** ☐

OR

C Put a cross here if you didn't tell your GP or nurse ☐

4. **Feeling different 'in yourself' from usual**

Did you have this?

Yes ☐ **Please complete A then B or C below**

No ☐ **Please go to Question 5**

A When did you first notice this? ☐☐ / ☐☐ / ☐☐ **OR** ☐

B When did you first tell your GP or nurse? ☐☐ / ☐☐ / ☐☐ **OR** ☐

OR

C Put a cross here if you didn't tell your GP or nurse ☐

Please try and describe what the feeling was

If you had any further symptoms which you feel are relevant to your cancer diagnosis, please list them below. If not, please go to Question 7.

Please describe the symptom here and complete A then B or C below

5.

**A** When did you <u>first notice</u> this?	☐☐ / ☐☐ / ☐☐ **OR** ☐
**B** When did you <u>first tell</u> your GP or nurse?	☐☐ / ☐☐ / ☐☐ **OR** ☐
**OR**	
**C** Put a cross here if you <u>didn't tell</u> your GP or nurse	☐

If you had another symptom you feel is relevant, please write it below, otherwise go to Question 7.

Please describe the symptom here and complete A then B or C below

6.

**A** When did you <u>first notice</u> this?	☐☐ / ☐☐ / ☐☐ **OR** ☐
**B** When did you <u>first tell</u> your GP or nurse?	☐☐ / ☐☐ / ☐☐ **OR** ☐
**OR**	
**C** Put a cross here if you <u>didn't tell</u> your GP or nurse	☐

If you had further symptoms you would like to tell us about, there is space at the end of the Questionnaire for you to write them.

Did your GP send you for any tests?

Thinking back to the time immediately before you diagnosis, did your GP send you for any of the following tests?

Please cross the appropriate boxes

7. Were you sent for any of these?

	Yes	Not sure	No
Blood test(s)	☐	☐	☐
CT Scan	☐	☐	☐
Ultrasound scan	☐	☐	☐
Barium Enema	☐	☐	☐
Sigmoidoscopy or colonoscopy (Looking at bowel with internal camera)	☐	☐	☐
X-ray	☐	☐	☐

Please go to Question 8

About you

The next questions relate to general information about you. It would help us to know more about the background of the people completing the questionnaire.

8. **Which best describes your employment status?**

Please cross one box only

- Employed full-time ☐
- Employed part-time ☐
- Self employed full-time ☐
- Self employed part-time ☐
- Unemployed (seeking work) ☐
- Unemployed (not seeking work) ☐
- Retired ☐
- Student ☐
- Permanently sick/disabled ☐
- Temporarily sick/disabled ☐
- Looking after family/home ☐
- Other, please describe ______

9. **What is your highest level of qualification?**

Please cross one box only

- Degree (or equivalent) ☐
- Diploma (or equivalent) ☐
- 'A' level ☐
- GCSE / 'O' level ☐
- None ☐
- Other, please specify ______

10. How would you describe your ethnicity

Please cross one box only

White ☐
or
- White British ☐
- White Irish ☐
- Other White background ☐

Mixed ☐
or
- White & Black Carribean ☐
- White & Black African ☐
- White & Asian ☐
- Other mixed background ☐

Black or Black British ☐
or
- Carribean ☐
- African ☐
- Other Black background ☐

Asian or Asian British ☐
or
- Indian ☐
- Pakistani ☐
- Bangladeshi ☐
- Other Asian background ☐

Chinese or other ethnic group ☐
or
- Chinese ☐
- Other ethnic group ☐

11.

Do you live alone?

Yes ☐ **Please go to Question 12**

No ☐ **Please say who you live with below**

Who do you live with?
(e.g. wife, husband, partner, family member)

12. Are you suffering from, or have you suffered from, any of the following in the past 2 years?

Please cross any that are applicable to you

- Asthma ☐
- Chronic Obstructive Pulmonary Disease (COPD) ☐
- Other lung disease (e.g. fibrosis, bronchiectatis etc) ☐
- Heart disease ☐
- Anxiety or depression ☐
- Inflammatory bowel disease ☐
- Irritable bowel syndrome ☐
- Peptic ulcer ☐
- Previous cancer ☐
- Diabetes ☐
- Arthritis ☐

13. About smoking

Please cross the appropriate statement

- Are you a current smoker? ☐
- Are you an ex-smoker? ☐
- Are you a non-smoker (never smoked)? ☐

14. Do you think you were more at risk of getting cancer because of your family history?

Yes ☐

No ☐

15. The following questions are about how completing the questionnaire made you feel.

A number of statements which people have used to describe themselves are given below. Read each statement and then cross the most appropriate box to indicate how you feel right now, at the moment. There are no right or wrong answers. Do not spend too much time on any one statement but give the answer which seems to describe your present feeling best.

		Not at all	Somewhat	Moderately	Very much
1.	I feel calm	☐	☐	☐	☐
2.	I am tense	☐	☐	☐	☐
3.	I feel upset	☐	☐	☐	☐
4.	I am relaxed	☐	☐	☐	☐
5.	I feel content	☐	☐	☐	☐
6.	I am worried	☐	☐	☐	☐

Please make sure you have answered all the questions.

If you have any other comments or there is anything else you would like to tell us about when you first noticed your symptoms, please do so using the space below.

If you feel you would like to talk to someone about this questionnaire at a later stage, please speak to the doctor or nurse who is looking after you, or be in touch with the research team, whose contact details are on your patient information sheet

Thank you for completing the questionnaire

Please give it to the research nurse

The next page is for the Researcher to complete

57727

86

Researcher - Post-completion Sheet

Researcher initials: ☐☐☐

Date questionnaire completed: Date ☐☐ / ☐☐ / ☐☐ Time ☐☐:☐☐

If not completed, give reason ☐

How long is it since the patient was told of his/her diagnosis? ☐

How long did it take the patient to complete the questionnaire?

- Less than 5 minutes ☐
- Between 5 and 10 minutes ☐
- Between 11 and 15 minutes ☐
- Longer than 15 minutes ☐

For the patient selected to <u>complete the questionnaire on their own</u>

Did the patient ask for any help? ☐ Yes ☐ No

If yes,

please specify what help was requested

____

____

please specify what help was given

____

____

For the patient selected for <u>the researcher-administered questionnaire</u>

Did the patient find any of the questions difficult? ☐ Yes ☐ No

If yes,

please specify which questions and what the difficulty was

____

____

____

____

Was the patient made anxious? ☐ Yes ☐ No

If yes,

please give details

____

____

____

____

Where did the patient complete the questionnaire?
(Please specify whether this was in a separate room alone, in a corner of the clinic, the ward or other

____

Thank you. Please now send the questionnaire and patient information sheet and consent form back to Cardiff University in the freepost envelope provided.

57727

PID - 87

Ymddiriedolaeth GIG Gogledd Orllewin Cymru
North West Wales NHS Trust

NHS WALES GIG CYMRU
Ymddiriedolaeth GIG Gogledd Cymru
North Wales NHS Trust

CID - ☐ GLAN CLWYD
☐ GWYNEDD
☐ MAELOR

Confidential Questionnaire

Looking At Your Symptoms Study

This Study has been funded by Cancer Research UK

Cardiff University / Prifysgol Caerdydd
Ysgol Glinigol Gogledd Cymru / North Wales Clinical School
Cancer Research UK
sewtu South East Wales Trials Unit / Uned Ymchwil De-ddwyrain Cymru

Dear Patient

I am very grateful to you for agreeing to help us with this study.

I hope the information you supply will help us give people a diagnosis earlier.

If you feel you would like to talk to someone about this questionnaire at a later stage, please speak to the doctor or nurse who is looking after you, or be in touch with the research team, whose contact details are on your patient information sheet.

I can assure you that anything you tell us will be treated in the utmost confidence.

Yours sincerely

Dr Richard Neal
Senior Clinical Lecturer
Cardiff University
Chief Investigator, Symptoms Study

About your symptoms

These questions are about symptoms you noticed before your diagnosis. We are interested in symptoms which you think are related to your cancer.

Please put a cross in the appropriate box, or write an answer as required.

Looking back...

1. Did you have any symptoms that you feel were due to your cancer before you were diagnosed?

Yes ☐ **Please continue with Question 2**

No ☐ **Please go to Section 2 on Page 5**

2. What was the first symptom that made you think something might be wrong?

☐ (answer box)

Please go to Question 3

The following questions are about when you first noticed a symptom and when you first told your GP or nurse about it.

Please give an exact date if you can. Otherwise give your best estimate. (For example approximately how long ago, the month or season). You wish to refer to your diary or calendar if you have it with you.

Here is an example question:

Decrease in appetite

Did you have this symptom?

Yes ☒ **Please complete A then B or C below**

No ☐ **Please go to Section 2**

A When did you first notice this? d d / m m / y y **OR** Estimate '3 months ago' or 'June'

B When did you first tell your GP or nurse? 2 3 / 1 2 / 0 7 **OR** Estimate

OR

C Put a cross here if you didn't tell your GP or nurse ☐

3. Change in bowel habit

Did you have this symptom?

Yes ☐ **Please complete A then B or C below**

No ☐ **Please go to Question 4**

A When did you first notice this?

☐☐ / ☐☐ / ☐☐ **OR** ☐

B When did you first tell your GP or nurse?

☐☐ / ☐☐ / ☐☐ **OR** ☐

OR

C Put a cross here if you didn't tell your GP or nurse ☐

4. Bleeding from back passage

Did you have this symptom?

Yes ☐ **Please complete A then B or C below**

No ☐ **Please go to Section 2**

A When did you first notice this?

☐☐ / ☐☐ / ☐☐ **OR** ☐

B When did you first tell your GP or nurse?

☐☐ / ☐☐ / ☐☐ **OR** ☐

OR

C Put a cross here if you didn't tell your GP or nurse ☐

Section 2

Please give an exact date if you can. Otherwise give your best estimate (for example approximately how long ago, the month or the season). You may wish to refer to your diary or calendar if you have it with you.

Did you have any of the following?

1. Decrease in appetite

Did you have this?

Yes ☐ **Please complete A then B or C below**

No ☐ **Please go to Question 2**

A When did you first notice this? ☐☐ / ☐☐ / ☐☐ **OR** ☐

B When did you first tell your GP or nurse? ☐☐ / ☐☐ / ☐☐ **OR** ☐

OR

C Put a cross here if you didn't tell your GP or nurse ☐

2. Unexplained weight loss

Did you have this?

Yes ☐ **Please complete A then B or C below**

No ☐ **Please go to Question 3**

A When did you first notice this? ☐☐ / ☐☐ / ☐☐ **OR** ☐

B When did you first tell your GP or nurse? ☐☐ / ☐☐ / ☐☐ **OR** ☐

OR

C Put a cross here if you didn't tell your GP or nurse ☐

3. Fatigue or tiredness that is unusual for you

Did you have this?

Yes ☐ **Please complete A then B or C below**

No ☐ **Please go to Question 4**

A When did you first notice this? ☐☐ / ☐☐ / ☐☐ **OR** ☐

B When did you first tell your GP or nurse? ☐☐ / ☐☐ / ☐☐ **OR** ☐

OR

C Put a cross here if you didn't tell your GP or nurse ☐

4. Feeling different 'in yourself' from usual

Did you have this?

Yes ☐ **Please complete A then B or C below**

No ☐ **Please go to Question 5**

A When did you first notice this? ☐☐ / ☐☐ / ☐☐ **OR** ☐

B When did you first tell your GP or nurse? ☐☐ / ☐☐ / ☐☐ **OR** ☐

OR

C Put a cross here if you didn't tell your GP or nurse ☐

Please try and describe what the feeling was

If you had any further symptoms which you feel are relevant to your cancer diagnosis, please list them below. If not, please go to Question 7.

Please describe the symptom here and complete A then B or C below

5.

**A**	When did you first notice this?	☐☐ / ☐☐ / ☐☐ **OR** ☐
**B**	When did you first tell your GP or nurse?	☐☐ / ☐☐ / ☐☐ **OR** ☐
	**OR**	
**C**	Put a cross here if you didn't tell your GP or nurse	☐

If you had another symptom you feel is relevant, please write it below, otherwise go to Question 7.

Please describe the symptom here and complete A then B or C below

6.

**A**	When did you first notice this?	☐☐ / ☐☐ / ☐☐ **OR** ☐
**B**	When did you first tell your GP or nurse?	☐☐ / ☐☐ / ☐☐ **OR** ☐
	**OR**	
**C**	Put a cross here if you didn't tell your GP or nurse	☐

If you had further symptoms you would like to tell us about, there is space at the end of the Questionnaire for you to write them.

Did your GP send you for any tests?

Thinking back to the time immediately before you diagnosis, did your GP send you for any of the following tests?

Please cross the appropriate boxes

7. Were you sent for any of these?

	Yes	Not sure	No
Blood test(s)	☐	☐	☐
CT Scan	☐	☐	☐
Ultrasound scan	☐	☐	☐
Barium Enema	☐	☐	☐
Sigmoidoscopy or colonoscopy (Looking at bowel with internal camera)	☐	☐	☐
X-ray	☐	☐	☐

Please go to Question 8

About you

The next questions relate to general information about you. It would help us to know more about the background of the people completing the questionnaire.

8. **Which best describes your employment status?**

Please cross one box only

- Employed full-time ☐
- Employed part-time ☐
- Self employed full-time ☐
- Self employed part-time ☐
- Unemployed (seeking work) ☐
- Unemployed (not seeking work) ☐
- Retired ☐
- Student ☐
- Permanently sick/disabled ☐
- Temporarily sick/disabled ☐
- Looking after family/home ☐
- Other, please describe ______

9. **What is your highest level of qualification?**

Please cross one box only

- Degree (or equivalent) ☐
- Diploma (or equivalent) ☐
- 'A' level ☐
- GCSE / 'O' level ☐
- None ☐
- Other, please specify ______

10. **How would you describe your ethnicity**

Please cross one box only

White ☐
or
White British ☐
White Irish ☐
Other White background ☐

Mixed ☐
or
White & Black Carribean ☐
White & Black African ☐
White & Asian ☐
Other mixed background ☐

Black or Black British ☐
or
Carribean ☐
African ☐
Other Black background ☐

Asian or Asian British ☐
or
Indian ☐
Pakistani ☐
Bangladeshi ☐
Other Asian background ☐

Chinese or other ethnic group ☐
or
Chinese ☐
Other ethnic group ☐

11.

Do you live alone?

Yes ☐ **Please go to Question 12**

No ☐ **Please say who you live with below**

Who do you live with?
(e.g. wife, husband, partner, family member)

12. **Are you suffering from, or have you suffered from, any of the following in the past 2 years?**

Please cross any that are applicable to you

- Asthma ☐
- Chronic Obstructive Pulmonary Disease (COPD) ☐
- Other lung disease (e.g. fibrosis, bronchiectatis etc) ☐
- Heart disease ☐
- Anxiety or depression ☐
- Inflammatory bowel disease ☐
- Irritable bowel syndrome ☐
- Peptic ulcer ☐
- Previous cancer ☐
- Diabetes ☐
- Arthritis ☐

13. **About smoking**

Please cross the appropriate statement

- Are you a current smoker? ☐
- Are you an ex-smoker? ☐
- Are you a non-smoker (never smoked)? ☐

14. **Do you think you were more at risk of getting cancer because of your family history?**

Yes ☐

No ☐

15. **The following questions are about how completing the questionnaire made you feel.**

A number of statements which people have used to describe themselves are given below. Read each statement and then cross the most appropriate box to indicate how you feel right now, at the moment. There are no right or wrong answers. Do not spend too much time on any one statement but give the answer which seems to describe your present feeling best.

		Not at all	Somewhat	Moderately	Very much
1.	I feel calm	☐	☐	☐	☐
2.	I am tense	☐	☐	☐	☐
3.	I feel upset	☐	☐	☐	☐
4.	I am relaxed	☐	☐	☐	☐
5.	I feel content	☐	☐	☐	☐
6.	I am worried	☐	☐	☐	☐

Please make sure you have answered all the questions.

If you have any other comments or there is anything else you would like to tell us about when you first noticed your symptoms, please do so using the space below.

If you feel you would like to talk to someone about this questionnaire at a later stage, please speak to the doctor or nurse who is looking after you, or be in touch with the research team, whose contact details are on your patient information sheet

Thank you for completing the questionnaire

Please give it to the research nurse

The next page is for the Researcher to complete

Researcher - Post-completion Sheet

Researcher initials: ☐☐☐

Date questionnaire completed: Date ☐☐ / ☐☐ / ☐☐ Time ☐☐:☐☐

If not completed, give reason ☐

How long is it since the patient was told of his/her diagnosis? ☐

How long did it take the patient to complete the questionnaire?

- Less than 5 minutes ☐
- Between 5 and 10 minutes ☐
- Between 11 and 15 minutes ☐
- Longer than 15 minutes ☐

For the patient selected to <u>complete the questionnaire on their own</u>

Did the patient ask for any help? ☐ Yes ☐ No

If yes,

please specify what help was requested

____

____

please specify what help was given

____

____

For the patient selected for <u>the researcher-administered questionnaire</u>

Did the patient find any of the questions difficult? ☐ Yes ☐ No

If yes,

please specify which questions and what the difficulty was

____

____

____

____

Was the patient made anxious? ☐ Yes ☐ No

If yes,

please give details

____

____

____

____

Where did the patient complete the questionnaire?
(Please specify whether this was in a separate room alone, in a corner of the clinic, the ward or other

____

Thank you. Please now send the questionnaire and patient information sheet and consent form back to Cardiff University in the freepost envelope provided.
